# Transposon fingerprinting using low coverage whole genome shotgun sequencing in Cacao (*Theobroma cacao* L.) and related species

**DOI:** 10.1186/1471-2164-14-502

**Published:** 2013-07-24

**Authors:** Saemundur Sveinsson, Navdeep Gill, Nolan C Kane, Quentin Cronk

**Affiliations:** 1 Department of Botany and Biodiversity Research Centre, University of British Columbia, 6270 University Boulevard, Vancouver, BC V6T 1Z4, Canada; 2 Department of Ecology & Evolutionary Biology, University of Colorado Boulder, 1800 Colorado Ave, Boulder, CO 80309, USA

**Keywords:** *Theobroma cacao*, Transposable elements, Next generation sequencing, Graph based clustering, Retrotransposon

## Abstract

**Background:**

Transposable elements (TEs) and other repetitive elements are a large and dynamically evolving part of eukaryotic genomes, especially in plants where they can account for a significant proportion of genome size. Their dynamic nature gives them the potential for use in identifying and characterizing crop germplasm. However, their repetitive nature makes them challenging to study using conventional methods of molecular biology. Next generation sequencing and new computational tools have greatly facilitated the investigation of TE variation within species and among closely related species.

**Results:**

(i) We generated low-coverage Illumina whole genome shotgun sequencing reads for multiple individuals of cacao (*Theobroma cacao*) and related species. These reads were analysed using both an alignment/mapping approach and a *de novo* (graph based clustering) approach. (ii) A standard set of ultra-conserved orthologous sequences (UCOS) standardized TE data between samples and provided phylogenetic information on the relatedness of samples. (iii) The mapping approach proved highly effective within the reference species but underestimated TE abundance in interspecific comparisons relative to the de novo methods. (iv) Individual *T. cacao* accessions have unique patterns of TE abundance indicating that the TE composition of the genome is evolving actively within this species. (v) LTR/Gypsy elements are the most abundant, comprising c.10% of the genome. (vi) Within *T. cacao* the retroelement families show an order of magnitude greater sequence variability than the DNA transposon families. (vii) *Theobroma grandiflorum* has a similar TE composition to *T. cacao*, but the related genus *Herrania* is rather different, with LTRs making up a lower proportion of the genome, perhaps because of a massive presence (c. 20%) of distinctive low complexity satellite-like repeats in this genome.

**Conclusions:**

(i) Short read alignment/mapping to reference TE contigs provides a simple and effective method of investigating intraspecific differences in TE composition. It is not appropriate for comparing repetitive elements across the species boundaries, for which *de novo* methods are more appropriate. (ii) Individual *T. cacao* accessions have unique spectra of TE composition indicating active evolution of TE abundance within this species. TE patterns could potentially be used as a “fingerprint” to identify and characterize cacao accessions.

## Background

Transposable elements (TEs) are a large and dynamically evolving part of plant genomes [1,2]. They occupy between 15% - 84% of plant genomes [3] and TE expansion is known to cause a significant increase in genome size in many cases [4]. Transposable elements are a major force in plant evolution, not only by causing genome expansions but also by altering gene function either through disruption [5] or acting as a raw material for new genes and novel functions [6,7].

Transposable elements are usually classified into two major classes based on their transposition mechanisms. Class I retrotransposons move about in a ‘copy-and-paste’ fashion, through a RNA intermediate, which is encoded back into DNA by an endogenous Reverse Transcriptase (RT) enzyme [8]. The two largest super-families of retrotransposons in plants, the LTR/Copia and LTR/Gypsy, have several other open reading frames, which play a role in the transposition, located between two regions of long terminal repeats (LTR) [1]. Class II DNA elements move about in genomes through a DNA intermediate. The most extensively studied group of class II elements transpose by a ‘cut-and-paste’ mechanism and are classified into several super-families based on sequence similarity [9]. Cut-and-paste DNA transposons are characterized by a transposase gene and a pair of flanking terminal inverted repeats (TIRS) [7].

Transposable elements are known to vary extensively in copy-number and nucleotide sequence among closely related species [4,10] and even within the same species [11]. Plant LTR retrotransposons are well known to have intraspecific variation in copy-number [12,13]. This, in combination with the easily amplifiable LTR domain, has been used in the development of molecular markers for several crop species [14-16]. In addition to the extensive presence/absence variability of the LTR elements, sequence heterogeneity is also known to be quite extensive [17]. The reverse transcriptase domain is the most extensively studied retrotransposon gene and it is known to show levels of heterogeneity from about 5% to 75% at the amino acid level [17]. Heterogeneity and sequence evolution of class II DNA transposons is relatively less studied, but a recent study shows that they can be quite heterogeneous [10].

Cacao (*Theobroma cacao L.*) is an economically important tree in the mallow family (*Malvaceae*) [18]*.* It is widely grown in tropical regions as the source of cocoa beans for the manufacture of chocolate [18]. Cacao has long been known to be genetically diverse [19] and traditionally three major lineages of Cacao varieties have been recognized: Trinitario, Criollo, and Forastero [20]. Recent work based on a variety of markers, including microsatellites and whole chloroplast genome sequences of several cacao varieties, has confirmed that the Criollo and Forastero groups are two distinct genetic lineages while the Trinitario group is of hybrid origin [21,22]. Cacao has a relatively small genome, estimated to be around 430 Mb and it has a published genome assembly of about 75% of its estimated genome size [23]. This small genome size can be partly explained by the relatively low abundance of transposable elements, compared to other angiosperms. TEs comprise only approximately a quarter of the cacao genome [23].

In this study we use low-coverage Illumina whole genome shotgun sequencing to investigate the evolutionary dynamics and comparative analysis of 3,500 TE families in nine *T. cacao* varieties and two related species, *Theobroma grandiflorum* and *Herrania balaensis*.

## Results

### Sequence coverage estimates and phylogenetic analysis using the UCOS contigs

The Illumina sequencing yielded 1.7 – 5.9 Gbp of high quality sequence per sample (Table 1). Average coverage of the ultra-conserved orthologous sequences (UCOS), estimated with BWA mapping, varied between 1.8 and 9.4X per sample (see Table 1). This value represents a relative measure of per single copy locus sequencing depth for our libraries. However it is important to note that this method may slightly underestimate the sequencing coverage of *H. balaensis* and *T. grandiflorum* due to sequence divergence in the UCOS among the three species. Furthermore the results using UCOS are consistent with results using flow cytometry genome size estimates (see Table 1 in [23]) The UCOS data was used to standardize the TE data between samples to provide relative TE abundance data. It was also used to estimate the relatedness of the accessions in order to provide an evolutionary framework for TE variation.

**Table 1:** Sequence summary statistics

**Name**	**Chloroplast haplotype**^**1**^	**Read length (bp)**	**No. reads**	**No. reads after trimming**	**UCOS coverage**
EET-64 (*T. cacao*)	Criollo	60	6.6E + 07	6.5E + 07	5.4
Criollo-22 (*T. cacao*)	Criollo	60	4.6E + 07	4.2E + 07	4.4
Stahel (*T. cacao*)	Criollo	60	6.0E + 07	5.7e + 07	5.3
Pentagonum (*T. cacao*)	Criollo	80	5.8E + 07	5.5E + 07	5.2
ICS39 (*T. cacao*)	Criollo	80	6.4E + 07	6.0E + 07	5.9
Amelonado (*T. cacao*)	Forastero	60	7.0E + 07	6.8E + 07	5.9
ICS06 (*T. cacao*)	Forastero	80	6.4E + 07	6.1E + 07	6.3
ICS01 (*T. cacao*)	Forastero	60	5.0E + 07	4.9E + 07	4.4
Scavina-6 (*T. cacao*)	Forastero	60	3.8E + 07	2.9E + 07	1.8
*T. grandiflorum* (Cupuaçu)	na	60	7.2E + 07	6.8E + 07	5.8
*H. balaensis*	na	80	7.7E + 07	7.4E + 07	9.4

Illumina sequence summary statistics and observed average coverage of the UCOS contigs for *Theobroma cacao, T. grandiflorum* and *Herrania balaensis* based on Burrows-Wheeler Aligner (BWA) alignments.

**Figure 1:**
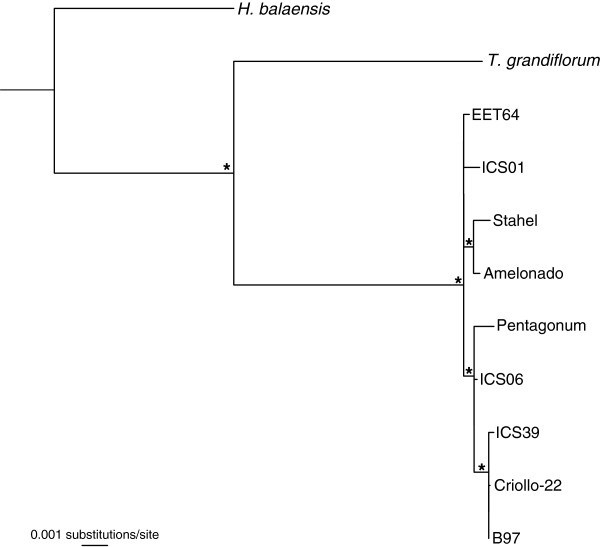
**Phylogeny of *Herrania balaensis, Theobroma grandiflorum *and nine of the *T. cacao *varieties.** The phylogenetic tree was constructed using partial sequence data of 97 ultra conserved orthologus sequences (UCOS). *Theobroma cacao* cv. Scavina-6 was excluded from the phylogenetic analysis due to low sequencing coverage. Nodes marked with asterisk have high bootstrap support (>90%).

The UCOS contigs were informative for the phylogenetic analysis of *H. balaensis*, *T. grandiflorum* and nine of the *T. cacao* varieties (Figure 1). Scavina-6 was excluded from the phylogenetic analysis due to low sequencing depth. The matrix used to construct the phylogeny consisted of 97 UCOS contigs with combined length of 20,438 nucleotides. Individual UCOS alignments varied in length, with the shortest alignment being 54 nucleotides and the longest 1,473 nucleotides. *Herrania balaensis* was set as the outgroup in the analysis which resulted in two main *Theobroma* clades (Figure 1). The first clade consists of only *T. grandiflorum*, with a 100% bootstrap support, and the second consisting of all *T. cacao* individuals. This is as expected given that *T. grandiflorum* and *T. cacao* are biologically distinct species. Within *T. cacao* there are two well-supported clades with *T. cacao* cv. Stahel and Amelonado grouping together and another well-supported grouping of *T. cacao* cv. Pentagonum, ICS06, ICS39, Criollo-22 and B97 (Figure 1). B97 is the variety used in the whole genome sequencing project of *T. cacao*[23]. EET-64 and ICS01 are unresolved on a polytomy.

### Variation in TE abundance using short read mapping

**Figure 2:**
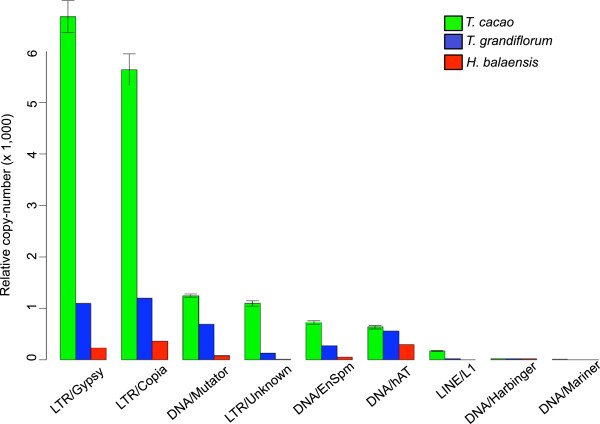
**Relative copy-number of transposable elements using reference based mapping.** Relative copy-numbers of the TE super-families in the three species represented with bar plots. Relative copy-number was calculated by dividing the total coverage of each super-family, within a sample, by the sample’s mean UCOS coverage. The much lower recovery of transposable elements in the other species is apparently due to mapping failure as the graph based clustering indicates that TE copy numbers are comparable in all species. Error bars represent standard deviation and correspond to intraspecific variation.

The coverage of the single copy UCOS genes was used as a baseline for standardization of TE coverage. Using this method, copy-numbers of TE superfamilies relative to the UCOS coverage in the three species were calculated (Figure 2). The intraspecific variation of copy-number in *Theobroma cacao* is represented by the error bars. The LTR super-families are the most numerous elements in the genomes of *H. balaensis*, *T. grandiflorum* and *T. cacao*, as previously shown. The difference in relative copy-number of class I LTR retroelements between the three species is quite striking. Using this method and the estimated genome sizes for the species [23], it can be calculated that LTR/Gypsy and LTR/Copia elements make up 9% and 7% of the genome respectively in *T. cacao*. In contrast these make up just 2% and 2% in *T. grandiflorum* and 0.6% and 0.5% in *Herrania* (Table 2). *De novo* approaches show the low values in the latter species to be artefacts (see next section). The apparently lower numbers in species distant from the reference are therefore likely due to mapping incompatibility, and this method is therefore not suitable for interspecies studies. The same pattern is also observed even when reads are mapped to the conserved regions of the LTR retrotransposons using less stringent settings in the short read aligner (Additional file 1). Mapping incompatibility is most likely attributable to retroelement divergence between distant species. This divergence is an important part of genome differentiation between species and potentially has implications for speciation and species divergence. On the other hand *de novo* methods are efficient in identifying any repetitive sequence that is either specific to a given species or has mutated beyond recognition that could render it unidentifiable using the mapping approach and therefore likely to be much more accurate for calculating copy numbers in species outside the reference species (see below).

**Table 2:** LTR retrotransposon frequencies in the three species estimated with two different methods

**Reference based mapping**			
	*T. cacao*	*T. grandiflorum*	*H. balanensis*
LTR/Gypsy	9%	2%	0.6%
LTR/Copia	7%	2%	0.5%
Graph based clustering			
	*T. cacao*	*T. grandiflorum*	*H. balanensis*
LTR/Gypsy	10%	10%	4%
LTR/Copia	5%	9%	7%

Comparison of estimated LTR retrotransposon frequencies as percentages of the genome, calculated with reference based mapping (upper half) and graph based clustering (lower half). Within *T. cacao* there is little discrepancy between the methods. Heterologous mapping between species produces different results suggesting that graph-based clustering may be more appropriate for inter-species comparisons (see Discussion).

### Variation of TE copy number using *de novo* approaches

**Figure 3:**
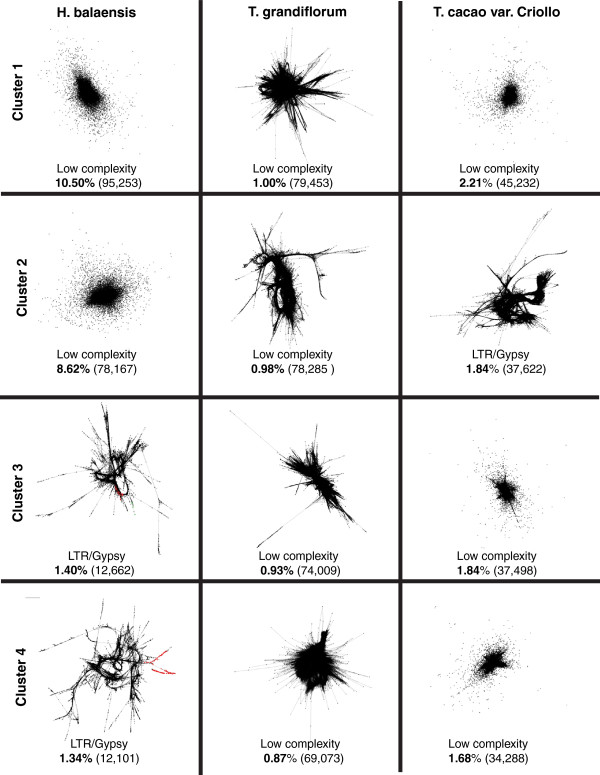
**Graph based clustering analysis of repetitive elements in the three species.** Graph layouts of the four largest clusters of repetitive elements detected in the graph based clustering analysis. *Herrania balaensis* is shown on the left, *T. grandiflorum* in the middle and *T. cacao* cv. Criollo on the right. Clusters are ordered by size, with largest at the top and fourth largest at the bottom. Below each graph layout is the class of the repetitive element, the genome percentage of each cluster and number of paired reads belonging to it in parentheses. Coloured regions in the some graphs represent conserved domains identified by RepeatExplorer. A total of 11,243,224 reads were used in the graph based clustering.

A major potential problem with studying interspecific variation using a mapping approach is that the reads from *Herrania* and *T. grandiflorum* are heterologously mapped to the *T. cacao* genome. Sequence variation between species affecting mapping quality could potentially have a considerable effect on the apparent frequency of TEs. We therefore employed a *de novo* approach in addition, using graph-based clustering. The graph based clustering analysis (Figure 3) of the short reads shows considerable differences in the representation of the major families of repetitive elements in the genomes of the three species studied here. Figure 3 shows the four largest clusters generated by RepeatExplorer, their identity and genome percentage. The most striking difference is between the two genera, where *H. balaensis* has two extremely large low complexity clusters with combined genome percentage of 19.4%. The two *Theobroma* species are more similar, with their largest clusters containing about 1-2% of the reads used in the graph based clustering. However, cluster 2 in *T. cacao* is largely composed of LTR/Gypsy elements, whereas the top four clusters in *T. grandiflorum* are all identified as low complexity elements. Using graph based clustering it can be calculated that LTR/Gypsy and LTR/Copia elements make up 10% and 5% of the genome respectively in *T. cacao* (comparing well with figures derived from a mapping approach, Table 2), whereas in *Herrania* they are 4% and 7% and in *Theobroma grandiflorum* 10% and 9%. This contrasts with the different figures arrived at for the other species using the mapping approach (see above). We conclude from comparing the results of mapping with the *de novo* approach that mapping quality in interspecific comparison has an important effect on the results. For a complete list of graph layouts of all clusters, see Additional file 2.

### Intraspecific variation of TE abundance in *T. cacao* using short read mapping and PCA

**Figure 4:**
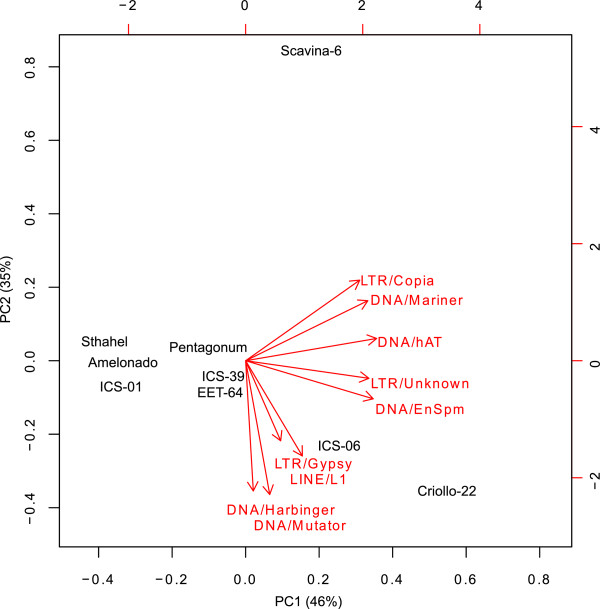
**PCA of the transposable element composition in the *Theobroma cacao *genotypes.** A biplot from a principal component analysis (PCA) using the standardized abundance of each TE super-family as explanatory variables. Percentage of the explained variance is shown in parentheses in the legend of the x- and y-axis.

The data on intraspecific relative abundance of TEs from short read mapping of the eight *T. cacao* accessions was analysed using principal coordinates analysis (PCA) (Figure 4). The first two axes include 81% of the variance. Axis 1 (46%) separates most strongly the Stahel and Amelonado varieties from the Criollo22 variety. TEs with the highest loadings on this axis are DNA transposons such as DNA/hAT and LTR retrotransposon such as Copia. Axis 2 (35%) separates most strongly Scavina-6 from Criollo22. TEs that load most strongly onto this axis are DNA transposons such as Mutator and Harbinger and Gypsy retrotransposons. This analysis clearly separates most of the *T. cacao* samples and shows several clusters of cacao accessions with the first two axes, some of which accord well with phylogenetic relatedness. Due to concerns that these results might be due to a sampling artefact, particularly given that Scavina-6 has the lowest coverage, the PCA analysis was repeated individually on 49 sub-sampled datasets that were equal in size. However, the same general pattern described above was always observed (Additional file 3).

### Sequence conservation of transposable elements in *T. cacao*

**Figure 5:**
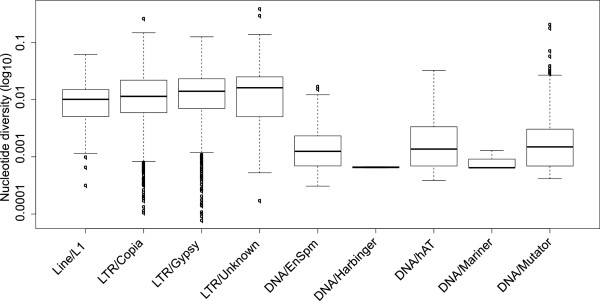
**Nucleotide variability of transposable elements in *Theobroma cacao*.** Box plot showing the nucleotide diversity across the super-families in *T. cacao*. This shows that DNA transposons have less variation at the superfamily level (see Discussion). Analyses were performed on standardized data sets (Methods) and values are presented transformed to a log10 scale.

Mapping of the short reads of the nine *T. cacao* libraries to the TE reference contigs revealed considerable levels of within species nucleotide variability, as calculated by number of nucleotide variants detected, divided by the length of the reference contigs. The nucleotide variability in the class II DNA transposons was around 10 times less than in the class I retroelements (Figure 5). These results illustrate different modes of sequence evolution in class I retroelements and class II DNA elements in the *T. cacao* studied here.

**Figure 6:**
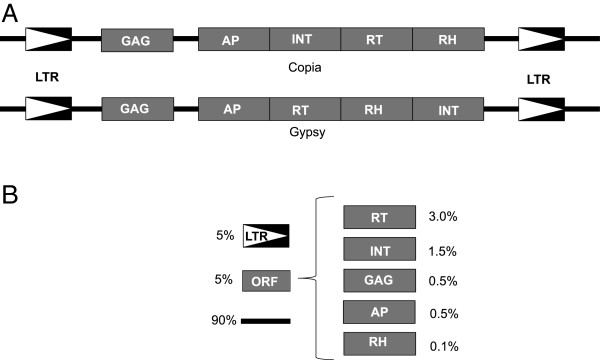
**Nucleotide diversity of LTR/Copia and LTR/Gypsy elements in *Theobroma cacao*. (A)** Schematic diagram of the structure of the two most common LTR retrotransposons super-families in the *T. cacao* genome. **(B)** Partitioning of nucleotide variation is shown as percentage values next to each of the retrotransposon components. The white arrows with black background represents the long terminal repeat (LTR), black line regions in between open reading frames (ORFs) and LTRs and grey boxes represent the following open reading frames: Reverse transcriptase (RT), integrase (IT), capsid protein (GAG), aspartic proteinase (AP) and Rnase H (RH).

Comparing the nucleotide variability in two classes of TEs is informative with regard to how these elements evolve on the whole but it sheds no light on what parts of individual elements are causing these differences. LTRDigest was able to identify characteristic features of LTR elements in 355 of the 650 class I families identified in [23]. In those 355 families, 90% of the nucleotide variability lay outside of protein coding genes and the long terminal repeats (LTRs), while 5% was in situated within the LTRs and 5% in genes (Figure 6). Reverse transcriptase (RT) was the largest contributor, containing about 3% of total nucleotide variability followed by integrase with about 1.5% and the three remaining genes contributed all less than 1% (see Figure 6). However these values are only informative of total variation not rates of variation, because they differ both in length and representation. LTRDigest does not identify all features in all the elements it interrogated. A better representation of the variability of the LTR genes and the long terminal repeat is to divide the number of nucleotide variants by the length of each feature, which yields a comparable estimate to the previously calculated nucleotide diversity. Those calculations show that the genes and the long terminal repeat all share a similar value, ranging from about 0.002 to 0.09, which are similar values to the average nucleotide diversity of the class I LTR retroelements shown in Figure 5.

## Discussion

The study of transposable elements (TEs) has been revolutionized by the increased availability and lowered costs of next generation sequencing (NGS) technologies [24]. NGS methods have not only been applied in TE studies of plants with high quality whole genomic sequences available such as *Zea luxurians*[25] and rice (*Oryza sativa*) [26] but also in organisms with limited genomic resources available such as barley (*Hordum vulgare*), pea (*Pisum sativum*) and banana (*Musa acuminata*) [27-29]. These studies demonstrate a strong correlation between copy-number estimation of TEs by traditional molecular methods and methods that count short reads from NGS experiments [25,27]. It was therefore not surprising that the copy-number estimation of TEs in this study fitted very well with previously published estimates in *T. cacao*, both in regard to the overall TE abundance in the genome, around 23%, and in the copy-number of the most abundant class I retroelement [23]. Our study therefore confirms the utility and reliability of studying genomic repeats using short reads directly.

### Different levels of nucleotide conservation in class I and class II TEs

The two major classes of TE, class I retroelements and class II DNA transposons, have been recognized for a long time as two fundamentally different groups of mobile elements probably present in all eukaryotic genomes [30]. The results presented in this study illustrate a considerable difference in the apparent conservation of the TEs in the genome of *T. cacao*, where the class I retrotransposons show significantly higher levels of heterogeneity, represented by an order of magnitude higher level of nucleotide diversity (Figure 5). This may be simply because DNA transposons are more narrowly defined at the superfamily level. However, one possible biological explanation of the high levels of heterogeneity in class I retroelements results from their transposition mechanism, as described in detail in [7]. Class I retrotransposons move about as a RNA intermediate, which is encoded into DNA before re-entry into the host genome by their endogenous reverse transcriptase enzyme, which is known to be low-fidelity, causing a high mutation rate [7,31].

### Inter- and intraspecific differences in TE abundance in *H. balaensis*, *T. grandiflorum* and *T. cacao*

Transposable elements are known to cause large inter- and intraspecific differences in the size and composition of plant genomes, demonstrated in barley (*Hordeum vulgare*) [11,32] and rice (*Oryza sativa*) [13]. However, our study only found relatively subtle intraspecific differences of the overall TE abundance in *T. cacao*. Nevertheless this slight intraspecific variation in TE copy number does potentially contribute to the variable genome sizes of different Cacao accessions reported in the supplementary material in the *T. cacao* genome paper and other sources [23,33,34]*.* Furthermore using a PCA approach to differentiate accessions based on TE abundance, wide separations do occur (Figure 4). The ability to separate cacao accessions according to TE composition is despite the fact that they are all closely related, some being of recent hybrid origin [23]. As massive parallel sequencing (MPS) costs fall, there is interest in using MPS to identify accessions, and such use has been called “ultra-barcoding” [23]. This paper shows that data generated for ultra-barcoding could also be used for “transposon composition fingerprinting” of cacao accessions (i.e. identification based on a unique spectrum of transposon composition).

### Mapping vs. de novo approaches to studying TEs from short reads

Our results (Table 2) suggest that the mapping approach, while reliable within the reference species (*T. cacao*), is unreliable in interspecific comparisons, at least for some TE families. The mapping approach reports considerable differences in the composition of repetitive elements in the three species studied (Figure 2). Apparently the genomes of *T. grandiflorum* and *H. balaensis* are significantly deficient in many LTR retrotransposons families that are very abundant in *T. cacao* (Figure 3). However this difference may be at least partly caused by low interspecific mapping quality of the short reads, since our reference contigs originate from the genome of *T. cacao*. The LTR retrotransposon families in particular have high nucleotide diversity (Figure 5), which is likely to cause problems in the mapping of the short reads.

The evidence for the failure of the mapping approach in interspecific comparisons comes from the *de novo* approach of graph based clustering using RepeatExplorer. This demonstrates that in both *T. grandiflorum* and *H. balaensis* the LTR TE families are more abundant than the mapping approach suggested (Table 2, Figure 3 and Additional file 2). More importantly the graph based clustering showed that the composition of *H. balaensis* and *T. grandiflorum* is quite different from *T. cacao.* Therefore we conclude that mapping based approaches are well suited to look at TE evolution in an intraspecific manner whereas *de novo* methods, such as graph based clustering, are much more useful in the exploration of differences in repetitive elements across species boundaries.

## Conclusions

The present study demonstrates considerable differences in transposable element composition among and even within species, highlighting their dynamic role in plant genome evolution. Variation of transposable elements in plants is important especially given the great abundance of transposable elements in plant genomes and their potential impact on the genespace. We used two different methods of looking at transposable element variation from Illumina short read data: reference-based mapping and graph-based clustering. Both are effective at capturing variation, although each is appropriate at different levels of taxonomic comparison. Reference based mapping works well within a species while graph-based clustering is preferred for between species comparisons.

## Methods

### Plant material and Illumina sequencing

Total genomic DNA was extracted from leaf tissue from 11 individuals belonging to three species in the Malvaceae: one *Herrania balaensis*, one *Theobroma grandiflorum* and nine *T. cacao.* Each *T. cacao* individual represented a different cultivated variety (see Table 1). DNA extraction was performed using the DNeasy Plant Mini Kit (Qiagen, Valencia, California, USA) according to the manufacturer’s protocol. Sequencing libraries were constructed using standard protocols and chemistry for the Illumina platform. Each library was sequenced on a single lane and generated either 60- or 80-bp paired-end sequences (see Table 1) on the Illumina GAII platform by Cofactor Genomics of St. Louis, MO (http://www.cofactorgenomics.com/). The raw reads are available on NCBI’s Short Read Archive [SRA048198].

### Mapping of reads, coverage estimates and SNP calling

The reference sequences of the transposable element (TE) families used in this study were extracted and characterized by the authors of the publication describing the *T. cacao* genome [23], who graciously made their data available for this study (Additional file 4). Briefly they identified class I retro-transposons using LTR_finder [35], LTRharvest [36] and in-house software that looked for signatures of class I retroelements, such as the long terminal repeat (LTR) and reverse transcriptase (RT). Class II elements were discovered using a blastX search of the transposase gene against the Repbase database proteins [37]. In all they identified 650 class I - and 2860 class II families. For more details see the supplementary methods in [23].

In order to estimate copy-number and sequence evolution of the TE families using our sequenced libraries of three species and nine *T. cacao* varieties, we mapped reads from each sequenced library to the TE reference contigs. Firstly, the reads were trimmed for quality, with bases below quality of 20 trimmed from the ends of each read. Quality trimmed reads were treated as single-end sequences and mapped to the TE reference contigs using BWA v0.6.1 [38] with the program’s default settings. The rationale behind treating the paired-end sequences as single-end was that TE copy-number estimation from coverage of the latter was believed to be more accurate, as paired-end information often links the repeat to different single-copy portions of the genome, preventing pairs from mapping near the boundaries of the repeated segment. Coverage estimates for each nucleotide position in the reference contigs were extracted from the sorted BAM file output of BWA using the genomeCoverageBed tool in the bedTools package v2.15.0 (genomeCoverageBed flags: -d -ibam) [39]. Relative copy-number of each TE family was estimated by counting the number of reads covering each position of the reference contig and dividing by the length of the contig. Proportional abundance was calculated for each species, by dividing the abundance of each TE super-family by the abundance of all TEs. Information on nucleotide variants detected in the reads, compared to the TE reference contigs, was extracted using samtools v0.1.7a [40]. Nucleotide diversity was estimated for each TE reference contig by counting the number of variable sites, with read-depth higher than 6 and base qualities higher than 20 (column 6 from samtools pileup –vcf output), and dividing by the length of the contig. To control for the effect of different read depths between different libraries, subsampling was used to ensure equality of total reads. Due to the repetitive nature of TEs, a variable site could represent a single nucleotide polymorphisms in a homologous copy, i.e. a heterozygote, or could stem from sequence divergence between different copies of a transposable element.

To account for differences in sequencing depth and read length of different libraries, reduced equalized data sets were used for some of the analyses presented here (Figures 4 and 5). The reduced data sets were generated by trimming the read length of all libraries to 60-bp and randomly extracting reads from all but the smallest sequenced library (Scavina-6). The purpose of this step was to make sure that variable read lengths and sequencing depths were not the cause of observed differences in TE coverage and nucleotide diversity. However any observed differences in UCOS coverage could be due to differences in genome sizes among the three species and the *T. cacao* varieties. Furthermore 49 sampling replicates were generated in order to test the effect of data sub-sampling on TE coverage estimates.

The class I LTR retrotransposons reference contigs were annotated using LTRHarvest [36] and LTRDigest [41]. These programs use similarity searches of conserved regions of LTR elements, such as the long terminal repeat and protein coding genes, to estimate the coordinates of the various features of the elements. That information was then used to estimate the variability of each of the LTR element feature, by combining the feature file output of LTRDigest with the nucleotide variant output of samtools.

In order to test whether we could better account for sequence divergence in the class I TE among the three species, we tried mapping the reads exclusively to conserved regions of the LTR retrotransposons and with relaxed BWA alignment stringency. Protein coding regions and the LTR were extracted from the reference contigs, based on the annotations from LTRHarvest [36] and LTRDigest [41] and the BWA alignment step was preformed with more relaxed settings (bwa flags: -l 1024 -i 0 -o 3). These settings allowed for more gaps and BWA used longer seed length for its short read alignments. These relaxed settings and the conserved regions of the LTR elements were only used to generate Additional file 1.

### Identification of, and mapping to UCOS contigs

A set of 357 Ultra Conserved Orthologous sequences (UCOS, http://compgenomics.ucdavis.edu/compositae_reference.php) was used to estimate the sequencing coverage of individual libraries as well as to estimate phylogenetic relationships between the three species and among the nine *T. cacao* varieties. These sequences represent single copy genes in *Arabidopsis thaliana* and tend to be conserved as single copy genes across Eukaryotes [42]. Since these genes are highly conserved and often present in a single copy in the genome, they are useful for estimating the sequencing coverage of each library and estimating copy number of the TE families. The 357 putative UCOS homologs in *T. cacao* were identified using blastx with an e-value threshold of 1E-34 [43]. The single copy status of the UCOS was verified by removing all contigs that had multiple hits to the *T. cacao* genome with a e-value lower than 1E-06. This left 245 UCOS to which the reads were mapped to using BWA, coverage of each UCOS contig was estimated using bedTools and single nucleotide polymorphisms (SNPs) called using samtools (see Additional file 5). Finally an average coverage was calculated for each library, by calculating the mean coverage of the 245 UCOS contigs. Coverage of each TE reference contig was divided by the mean UCOS coverage, in order to estimate a relative copy-number of TEs to single copy nuclear genes.

### Phylogenetic analysis using the UCOS contigs

A phylogenetic matrix was constructed by using the 245 UCOS contigs identified above as reference for short read mapping and by calling SNPs using previously described methods. *Theobroma cacao* cv. Scavina-6 was excluded from the phylogenetic analysis due to low sequencing coverage. For the construction of the matrix, only positions that were covered by 6 or more high quality reads in a given sample, with base quality equal or larger to 20 (column 6 in samtools pileup -vcf output) were used. Positions containing any ambiguous nucleotides, i.e. heterozygotes, were converted to Ns as were all other positions that did not meet previously mentioned criteria. Finally Ns were converted to gaps, trimAl v.1.2 [44] used to remove all gaps and to convert the alignments to nexus format (trimAl flags: -nogap -nexus). All alignments shorter than 50 nucleotides were excluded from further analysis, leaving 97 UCOS for further analysis. A matrix with positional information of each of the UCOS contigs was constructed using phyutility v.2.2.4 [45] (phyutility flags: -concat) (Additional file 6), for a combined analysis that includes separate analyses of each contig using a coalescence-based program (see below). Gene trees of individual UCOS alignments longer than 50 nucleotides were estimated with RAxML v7.2.6 [46], using 10 independent runs and the GTRGAMMA sequence substitution model (raxml flags: -m GTRGAMMA -N 10). In order to estimate a single phylogeny of the three species and nine remaining *T. cacao* varieties (Scavina-6 excluded), a STAR (species trees based on average ranks of coalescences) phylogeny [47] was constructed using the phybase R package (v.1.3) [48]. STAR uses the mean ranks of coalescent occurrences in a set of gene trees to construct a species tree topology [47]. In order to estimate branch lengths on the STAR tree, model parameters of the entire matrix were estimated using jModelTest v2.0.2 [49,50] and GARLI v2.0 [51] used to optimize model parameters and to add branch lengths to the STAR tree. Support values for the STAR phylogeny were estimated using a multi locus bootstrap [52] method implemented in the phybase package [48]. One thousand multi locus bootstrap replicates were analyzed using Phyml v3.0 [53], STAR trees estimated for each set of bootstrap replicates and a consensus tree constructed from all the STAR trees using the consense program in the phylip package v3.69 [54].

### Graph based clustering of the Illumina reads

The repetitive elements of three species studied here were also investigated in a *de novo* fashion using RepeatExplorer [55], which is a graph based clustering method of characterizing repetitive elements described in [56], with the program’s default settings. The Criollo-22 individual was chosen as the *T. cacao* representative. Briefly, RepeatExplorer uses information from sequence similarity among the reads and their partial overlap to construct graphs. Graphs are constructed using a Louvain method [57], where sequence reads are represented by vertices, edges are connected with overlapping reads and edge weights correspond to the similarity score among reads. The graph layouts are then examined in order to find separate clusters of reads that are often connected and correspond to distinct families of genomic repeats. These clusters are analyzed in regards to their size, determined by the number of reads comprising each cluster as well as their graph topology which gives information about their structure and variability. RepeatExplorer also performs a sequence similarity search of each cluster against RepBase [37] in order to identify the type of the repetitive elements present in the cluster. If the predicted number of nodes exceeds the capacity of the available RAM, RepeatExplorer randomly subsamples the reads. RepeatExplorer outputs a comma separated value (csv) file, containing relevant information of the clusters it identified and that consist of 0.01% or more of the reads used in the analysis (default cut-off). The program calculates the genome percentage, which is the number of reads in each cluster divided by the all the reads used in the graph based clustering (11,243,224 reads in total). An in house python script (available on request) was used parse the csv output file and combine parts of it with the figures of graph layouts. The output of that script is a panel of graph layouts, with each cluster’s most abundant class of element, in addition to the genome percentage and number of paired-end reads belonging to the cluster.

### Statistical analysis

Similarity of TE composition among sequenced individuals was investigated with a principal component analysis (PCA) using coverage of each TE super-family in the genomes of *H. balaensis, T. grandiflorum* and the nine *T. cacao* varieties. The PCA was performed using the *prcomp* function in R v2.14.1 [58], using the abundance of each super-family as explanatory variables with a natural logarithmic transformation and *scale = TRUE*. An in-house R script was used to run the PCA analysis on all sub-sampled data sets. The reduced data set ensured that differences in sequencing depth and read length did not affect the results.

## Competing interests

The authors declared no competing interest concerning the work in this paper.

## Authors’ contributions

SS planned the work, performed the data analysis and wrote the manuscript. NG and NCK assisted in data analysis and with writing the manuscript. QC jointly planned the work, co-wrote the manuscript and provided partial funding for the study. All authors read and approved the final manuscript.

## Supplementary Material

Additional file 1 **Relative copy-number of transposable elements using reference based mapping to conserved regions of the class I LTR elements.** Relative copy-numbers of the TE super-families in the three species represented with bar plots. Relative copy-number was calculated by dividing the total coverage of each super-family, within a sample, by the sample’s mean UCOS coverage. The mapping was preformed with relaxed settings in the short read aligner and the reads were mapped to conserved regions of class I LTR elements. Click here for file

Additional file 2 **Graph layouts of all the clusters generated in the graph based clustering analysis.** Graph layouts of clusters that contained 0.01% or more of the short reads used in the graph based clustering. *Herrania balaensis* is shown on the left, *T. grandiflorum* in the middle and *T. cacao* cv. Criollo on the right. Clusters are ordered by size, with largest at the top. Below each graph layout is the class of the repetitive element, the genome percentage of each cluster and number of paired reads belonging to it in parentheses. Coloured regions in some graphs represent conserved domains identified by RepeatExplorer. It should be noted that a few clusters annotated as “low complexity” may actually be referable to plastid sequence (e.g. CL19,CL26 & CL78). Click here for file

Additional file 3 **Biplots from PCAs on all of the sub-sampled datasets.** Biplots from principal component analysis on every sub-sampled dataset generated, 49 in total. The abundance of each TE super-family was used as explanatory variables and the percentage of the explained variance is shown in parentheses in the legend of the x- and y-axis of the biplot. Click here for file

Additional file 4 **The *Theobroma cacao *TE reference contigs used in this study.** A compressed file in a zip format that contains two text files with the reference contigs of the transposable elements used in this study in a fasta format. These contigs were constructed by the authors of the *T. cacao* genome paper [23] who graciously shared them with the authors of this study. Click here for file

Additional file 5 **UCOS contigs used.** A text file with containing the 245 *Theobroma cacao* UCOS contigs used in this study. The contigs were identified by blasting 357 *Arabidopsis thaliana* UCOS contigs to the *T. cacao* genome. One hundred and twelve contigs with more than one significant hits (e-value lower that 1E-06) were excluded. Click here for file

Additional file 6 **Phylogenetic matrix.** The phylogenetic matrix which was used to construct Figure 1. The matrix in nexus format that consists of a concatenated sequence of 97 partially sequenced UCOS genes and the positional information of individual gene in a Mr.Bayes nexus block. Click here for file
