# Supplementary figures and images for: Transposon fingerprinting using low coverage whole genome shotgun sequencing in Cacao (Theobroma cacao L.) and related species
Source: BMC Genomics. 2013 Jul 24;14:502. doi: 10.1186/1471-2164-14-502 (PMC3726317; doi:10.1186/1471-2164-14-502)

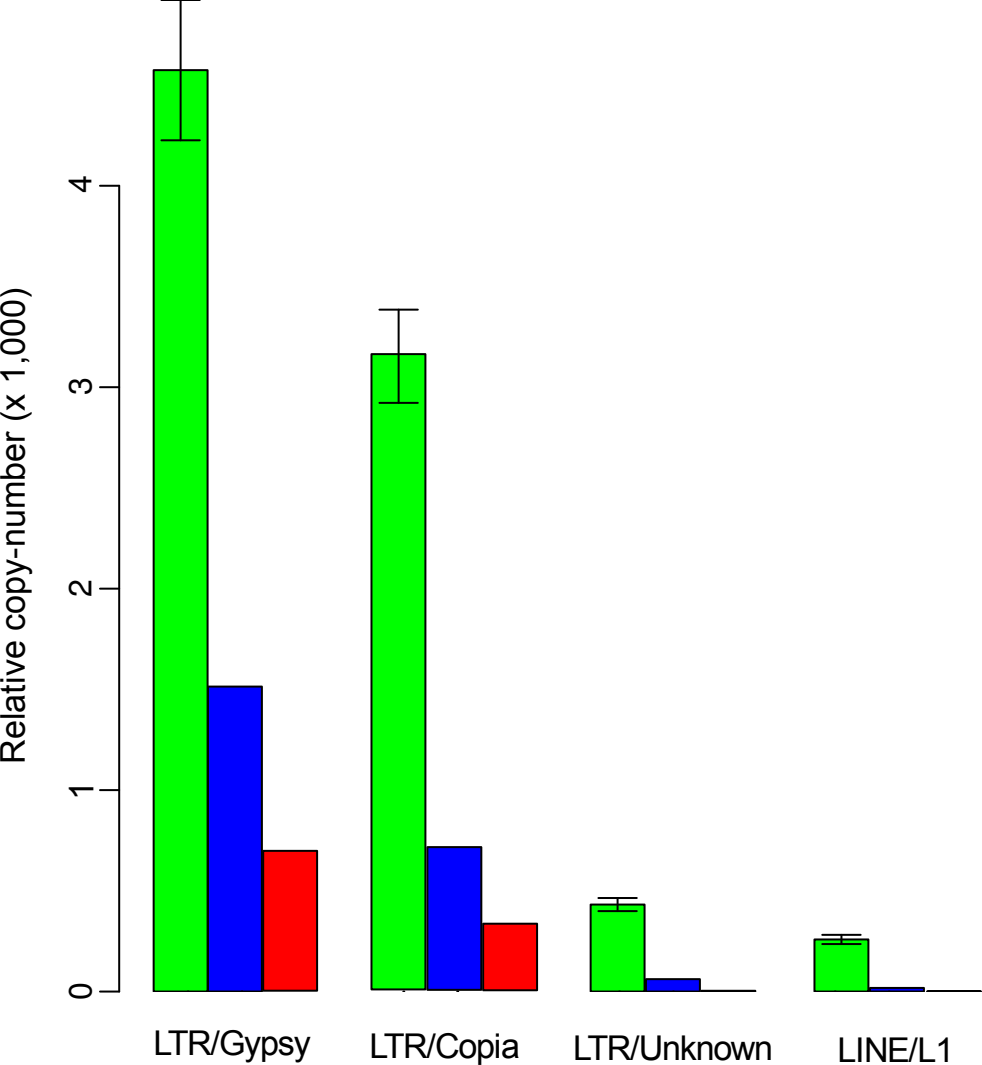

Supplement: Additional file 1 — Relative copy-number of transposable elements using reference based mapping to conserved regions of the class I LTR elements. Relative copy-numbers of the TE super-families in the three species represented with bar plots. Relative copy-number was calculated by dividing the total coverage of each super-family, within a sample, by the sample’s mean UCOS coverage. The mapping was preformed with relaxed settings in the short read aligner and the reads were mapped to conserved regions of class I LTR elements. [file 1471-2164-14-502-S1.pdf]
