# Supplementary material for: Transposon fingerprinting using low coverage whole genome shotgun sequencing in Cacao (Theobroma cacao L.) and related species
Source: BMC Genomics. 2013 Jul 24;14:502. doi: 10.1186/1471-2164-14-502 (PMC3726317; doi:10.1186/1471-2164-14-502)
Supplement: Additional file 2 — Graph layouts of all the clusters generated in the graph based clustering analysis. Graph layouts of clusters that contained 0.01% or more of the short reads used in the graph based clustering. Herrania balaensis is shown on the left, T. grandiflorum in the middle and T. cacao cv. Criollo on the right. Clusters are ordered by size, with largest at the top. Below each graph layout is the class of the repetitive element, the genome percentage of each cluster and number of paired reads belonging to it in parentheses. Coloured regions in some graphs represent conserved domains identified by RepeatExplorer. It should be noted that a few clusters annotated as “low complexity” may actually be referable to plastid sequence (e.g. CL19,CL26 & CL78). [file 1471-2164-14-502-S2.pdf]

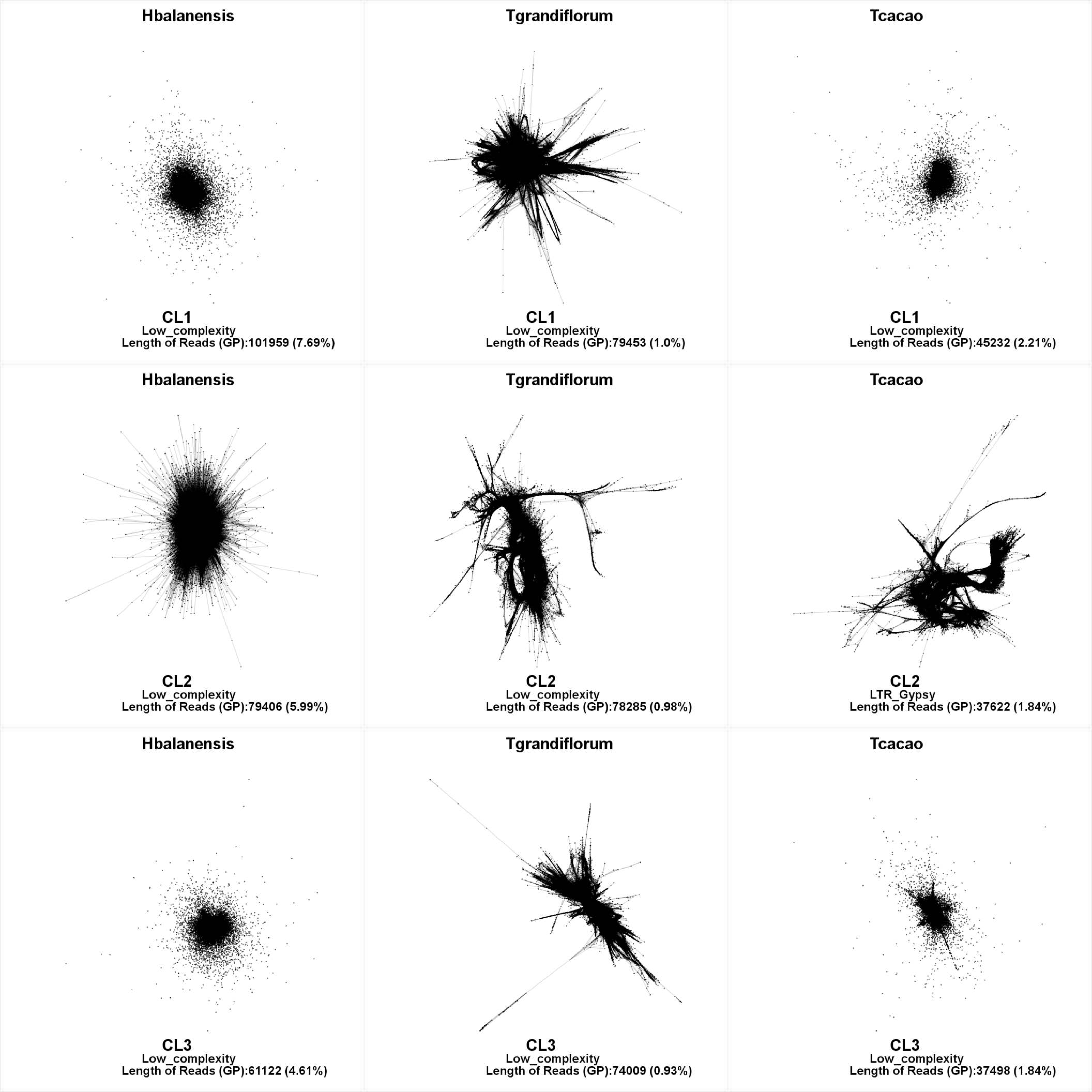
Hbalanensis
Tgrandiflorum
Tcacao
CL1
Low_complexity
Length of Reads (GP):101959 (7.69%)
CL1
Low_complexity
Length of Reads (GP):79453 (1.0%)
CL1
Low_complexity
Length of Reads (GP):45232 (2.21%)
Hbalanensis
Tgrandiflorum
Tcacao
CL2
Low_complexity
Length of Reads (GP):79406 (5.99%)
CL2
Low_complexity
Length of Reads (GP):78285 (0.98%)
CL2
LTR_Gypsy
Length of Reads (GP):37622 (1.84%)
Hbalanensis
Tgrandiflorum
Tcacao
CL3
Low_complexity
Length of Reads (GP):61122 (4.61%)
CL3
Low_complexity
Length of Reads (GP):74009 (0.93%)
CL3
Low_complexity
Length of Reads (GP):37498 (1.84%)

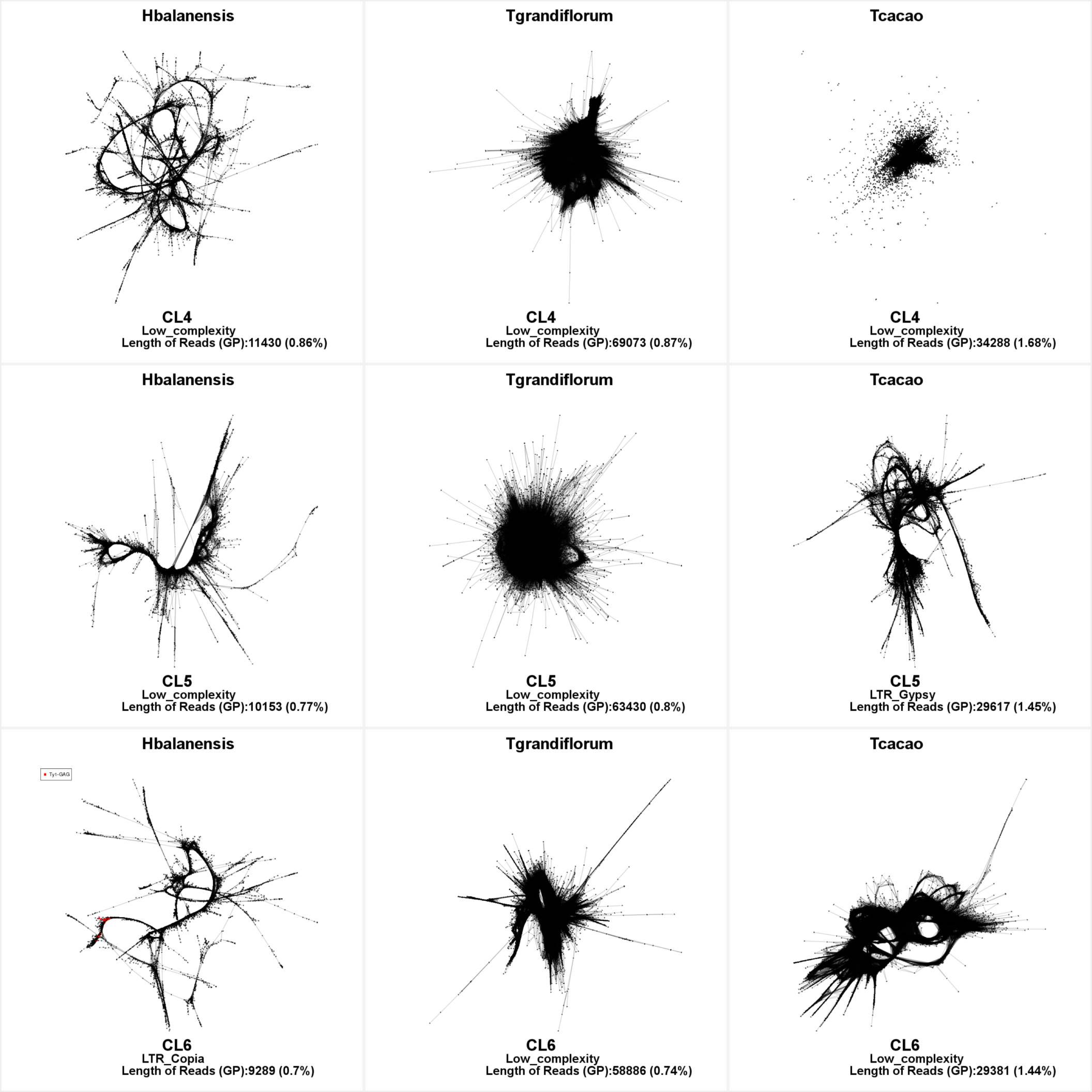
Hbalanensis
CL4
Low_complexity
Length of Reads (GP):11430 (0.86%)
Tgrandiflorum
CL4
Low_complexity
Length of Reads (GP):69073 (0.87%)
Tcacao
CL4
Low_complexity
Length of Reads (GP):34288 (1.68%)
Hbalanensis
CL5
Low_complexity
Length of Reads (GP):10153 (0.77%)
Tgrandiflorum
CL5
Low_complexity
Length of Reads (GP):63430 (0.8%)
Tcacao
CL5
LTR_Gypsy
Length of Reads (GP):29617 (1.45%)
Hbalanensis
Ty1-GAG
CL6
LTR_Copia
Length of Reads (GP):9289 (0.7%)
Tgrandiflorum
CL6
Low_complexity
Length of Reads (GP):58886 (0.74%)
Tcacao
CL6
Low_complexity
Length of Reads (GP):29381 (1.44%)

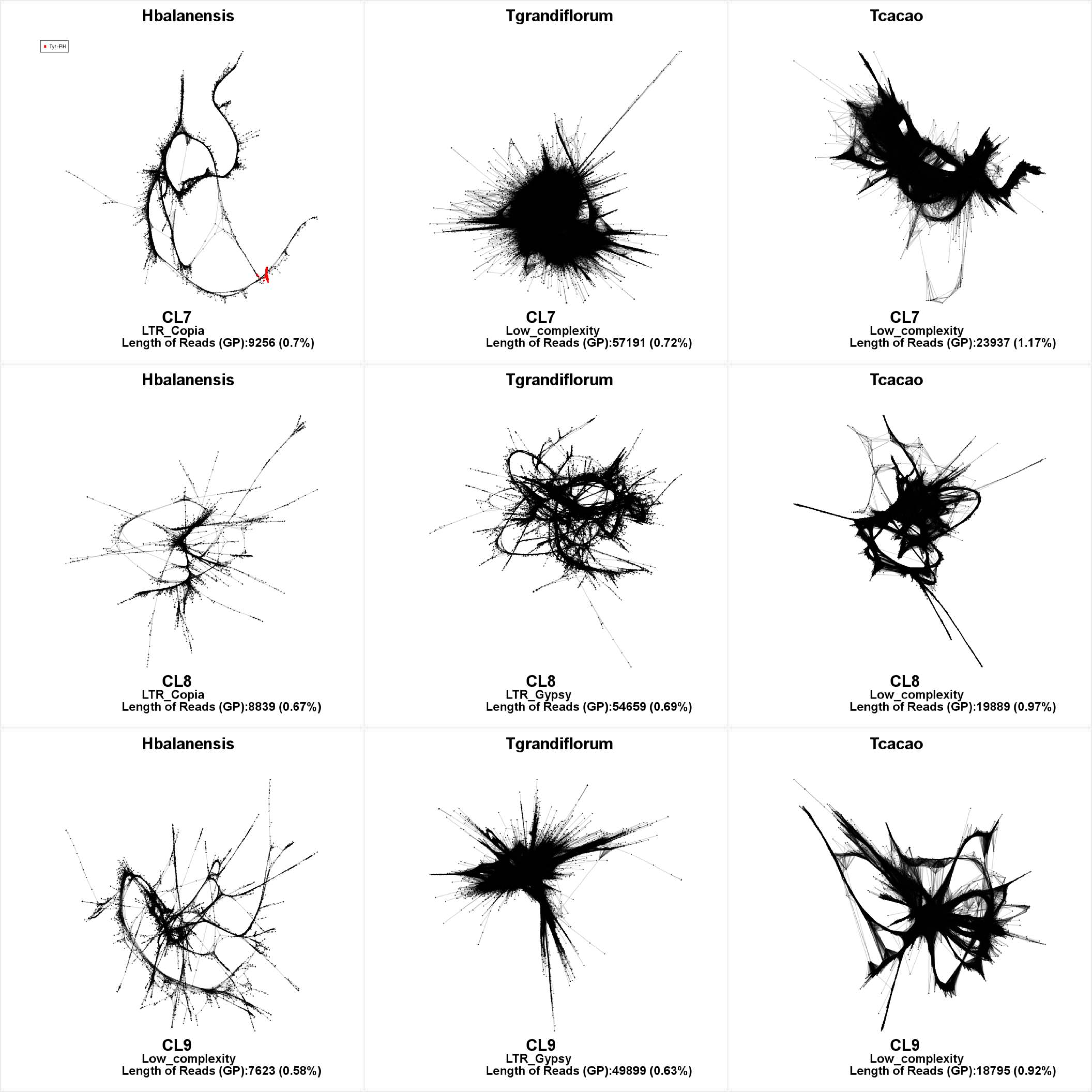
Hbalanensis
Ty1-RH
CL7
LTR_Copia
Length of Reads (GP):9256 (0.7%)
Tgrandiflorum
CL7
Low_complexity
Length of Reads (GP):57191 (0.72%)
Tcacao
CL7
Low_complexity
Length of Reads (GP):23937 (1.17%)
Hbalanensis
CL8
LTR_Copia
Length of Reads (GP):8839 (0.67%)
Tgrandiflorum
CL8
LTR_Gypsy
Length of Reads (GP):54659 (0.69%)
Tcacao
CL8
Low_complexity
Length of Reads (GP):19889 (0.97%)
Hbalanensis
CL9
Low_complexity
Length of Reads (GP):7623 (0.58%)
Tgrandiflorum
CL9
LTR_Gypsy
Length of Reads (GP):49899 (0.63%)
Tcacao
CL9
Low_complexity
Length of Reads (GP):18795 (0.92%)

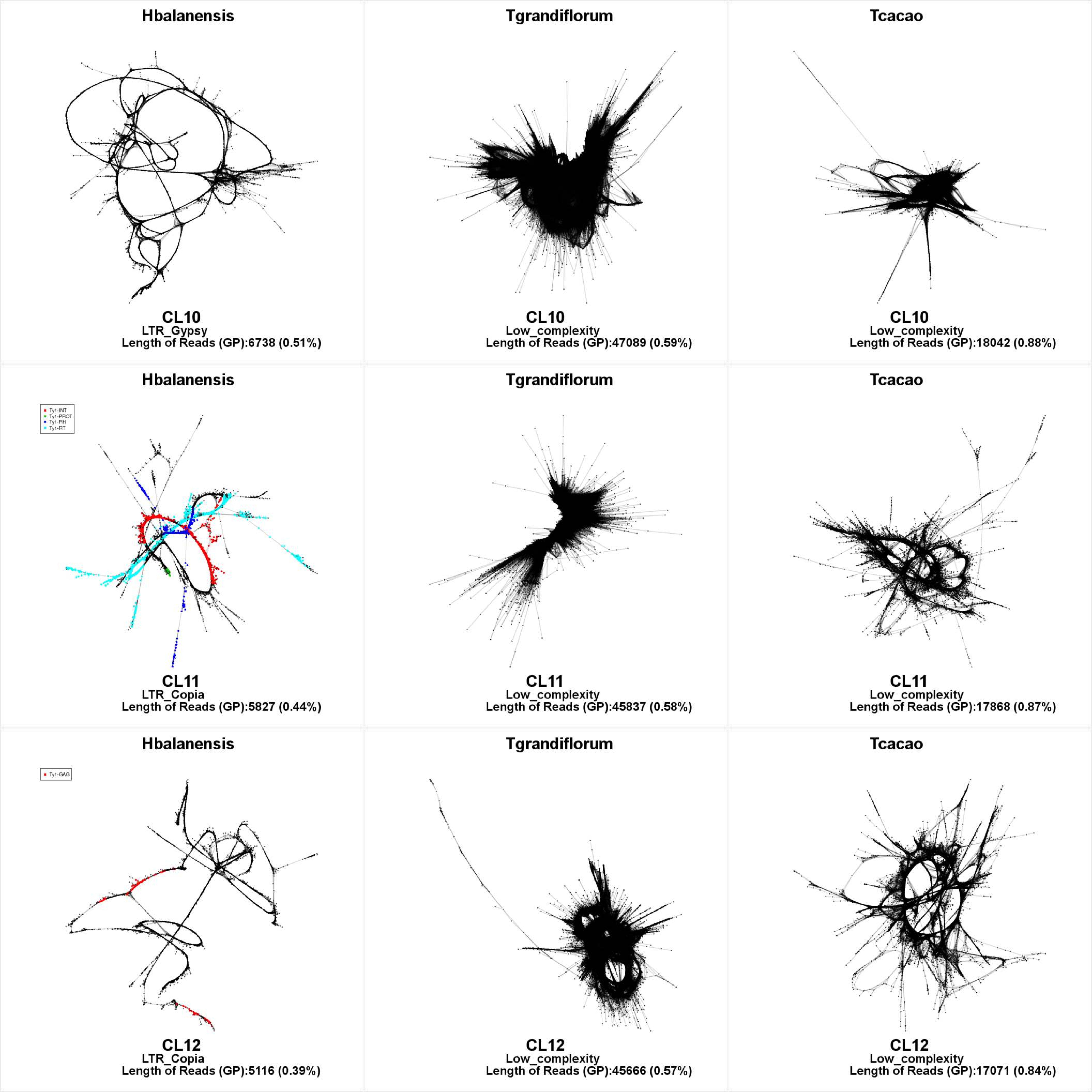
Hbalanensis
CL10
LTR_Gypsy
Length of Reads (GP):6738 (0.51%)
Tgrandiflorum
CL10
Low_complexity
Length of Reads (GP):47089 (0.59%)
Tcacao
CL10
Low_complexity
Length of Reads (GP):18042 (0.88%)
Hbalanensis
Ty1-INT
Ty1-PROT
Ty1-RH
Ty1-RT
CL11
LTR_Copia
Length of Reads (GP):5827 (0.44%)
Tgrandiflorum
CL11
Low_complexity
Length of Reads (GP):45837 (0.58%)
Tcacao
CL11
Low_complexity
Length of Reads (GP):17868 (0.87%)
Hbalanensis
Ty1-GAG
CL12
LTR_Copia
Length of Reads (GP):5116 (0.39%)
Tgrandiflorum
CL12
Low_complexity
Length of Reads (GP):45666 (0.57%)
Tcacao
CL12
Low_complexity
Length of Reads (GP):17071 (0.84%)

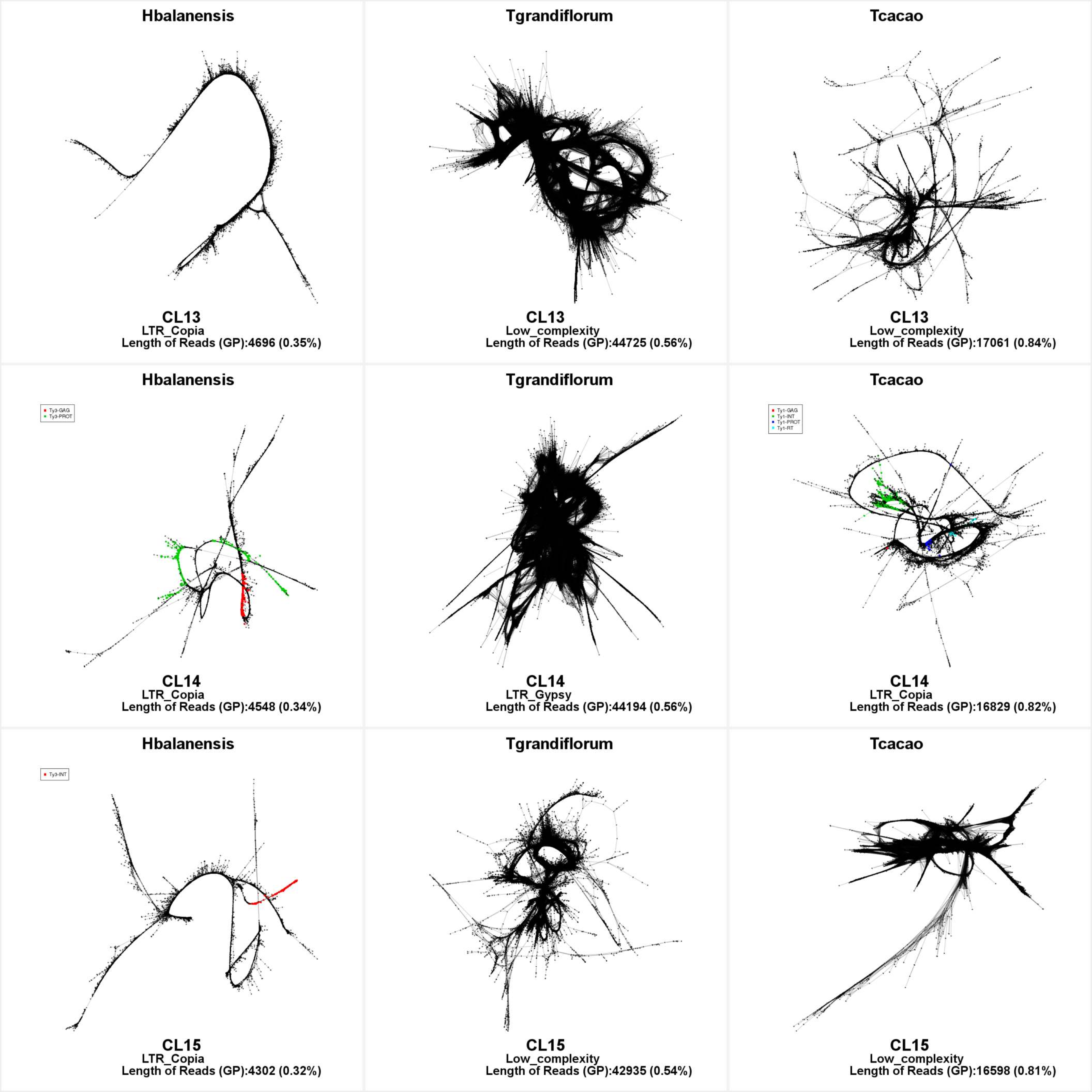
Hbalanensis
CL13
LTR_Copia
Length of Reads (GP):4696 (0.35%)
Tgrandiflorum
CL13
Low_complexity
Length of Reads (GP):44725 (0.56%)
Tcacao
CL13
Low_complexity
Length of Reads (GP):17061 (0.84%)
Hbalanensis
Ty3-GAG
Ty3-PROT
CL14
LTR_Copia
Length of Reads (GP):4548 (0.34%)
Tgrandiflorum
CL14
LTR_Gypsy
Length of Reads (GP):44194 (0.56%)
Tcacao
Ty1-GAG
Ty1-INT
Ty1-PROT
Ty1-RT
CL14
LTR_Copia
Length of Reads (GP):16829 (0.82%)
Hbalanensis
Ty3-INT
CL15
LTR_Copia
Length of Reads (GP):4302 (0.32%)
Tgrandiflorum
CL15
Low_complexity
Length of Reads (GP):42935 (0.54%)
Tcacao
CL15
Low_complexity
Length of Reads (GP):16598 (0.81%)

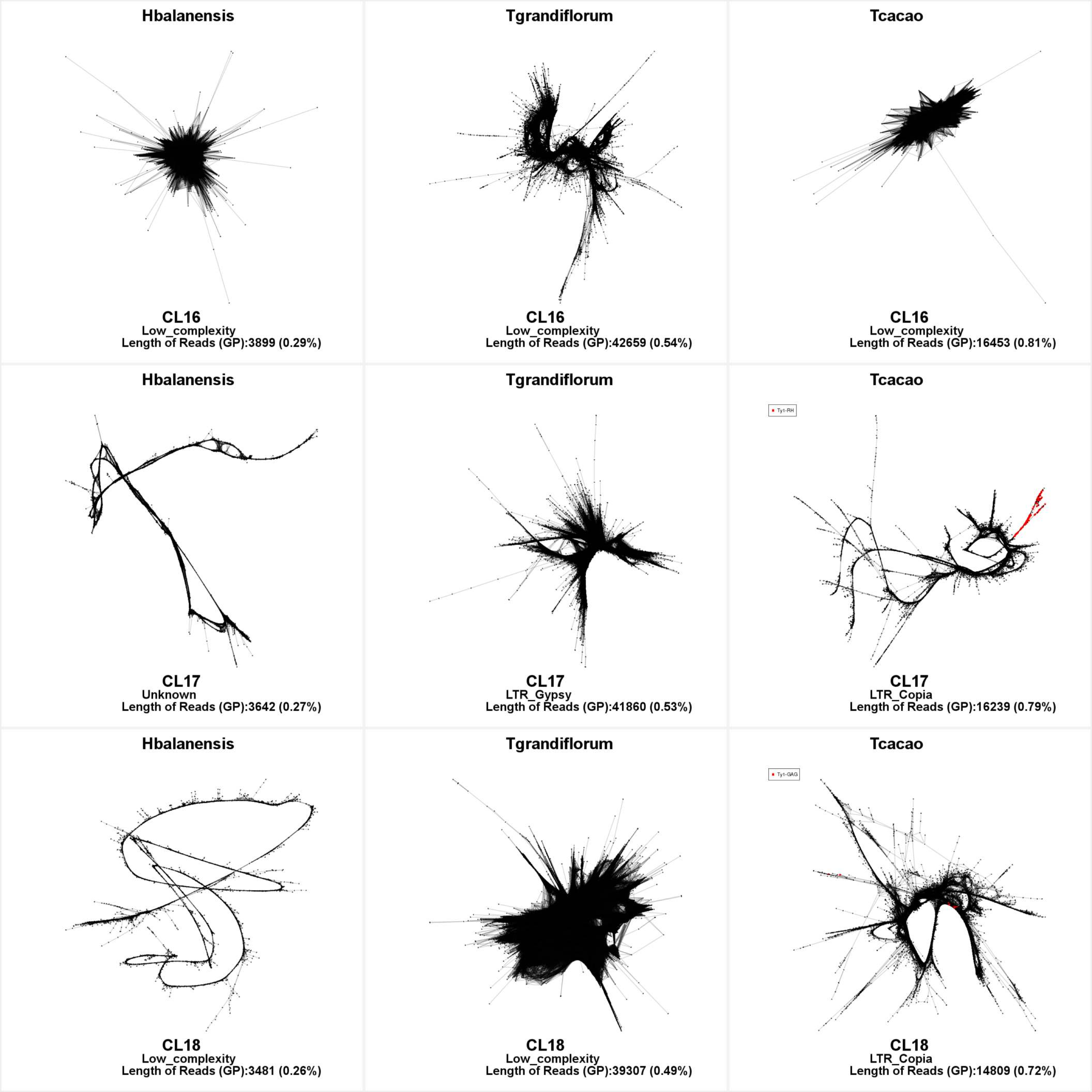
Hbalanensis
CL16
Low_complexity
Length of Reads (GP):3899 (0.29%)
Tgrandiflorum
CL16
Low_complexity
Length of Reads (GP):42659 (0.54%)
Tcacao
CL16
Low_complexity
Length of Reads (GP):16453 (0.81%)
Hbalanensis
CL17
Unknown
Length of Reads (GP):3642 (0.27%)
Tgrandiflorum
CL17
LTR_Gypsy
Length of Reads (GP):41860 (0.53%)
Tcacao
Ty1-RH
CL17
LTR_Copia
Length of Reads (GP):16239 (0.79%)
Hbalanensis
CL18
Low_complexity
Length of Reads (GP):3481 (0.26%)
Tgrandiflorum
CL18
Low_complexity
Length of Reads (GP):39307 (0.49%)
Tcacao
Ty1-GAG
CL18
LTR_Copia
Length of Reads (GP):14809 (0.72%)

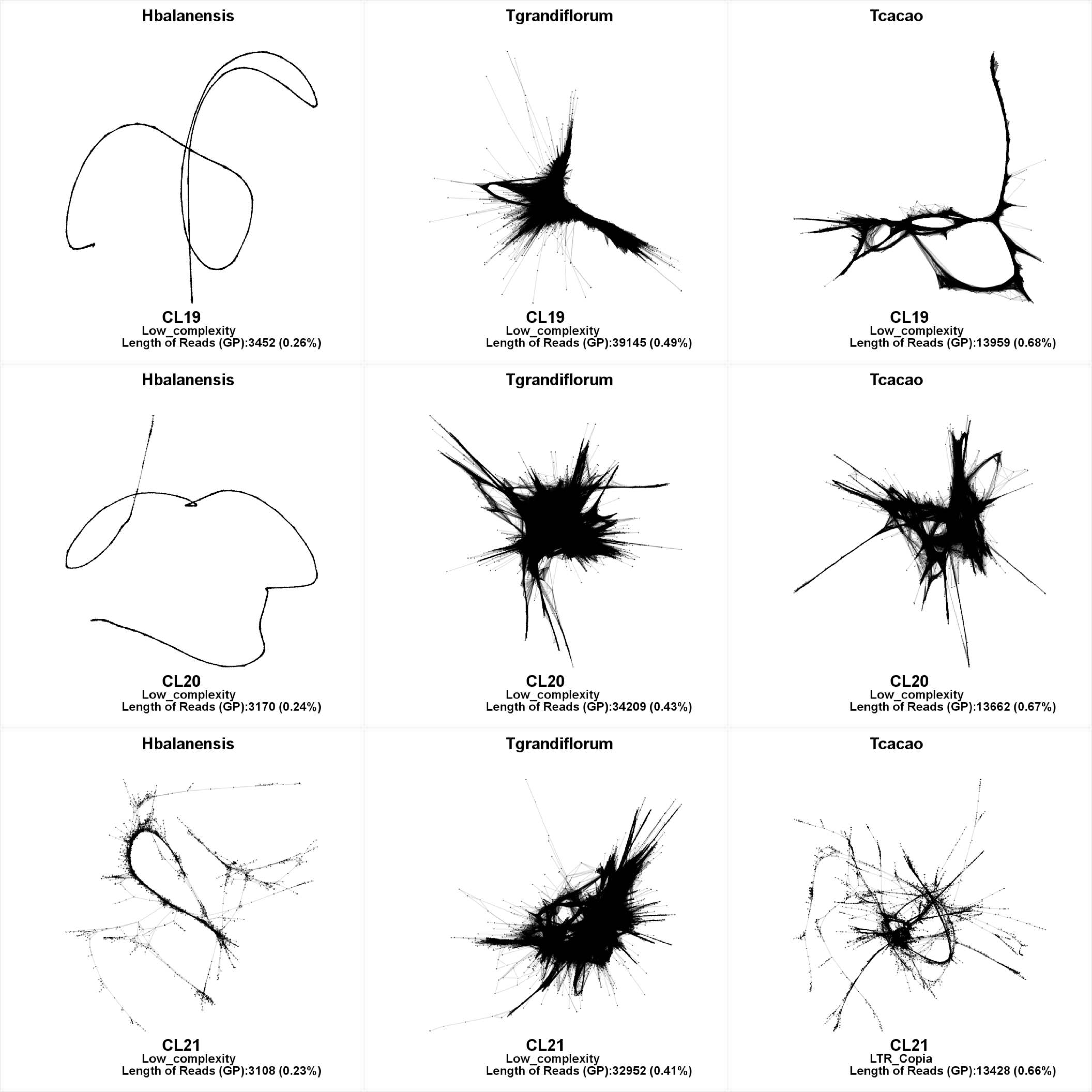
Hbalanensis
CL19
Low_complexity
Length of Reads (GP):3452 (0.26%)
Tgrandiflorum
CL19
Low_complexity
Length of Reads (GP):39145 (0.49%)
Tcacao
CL19
Low_complexity
Length of Reads (GP):13959 (0.68%)
Hbalanensis
CL20
Low_complexity
Length of Reads (GP):3170 (0.24%)
Tgrandiflorum
CL20
Low_complexity
Length of Reads (GP):34209 (0.43%)
Tcacao
CL20
Low_complexity
Length of Reads (GP):13662 (0.67%)
Hbalanensis
CL21
Low_complexity
Length of Reads (GP):3108 (0.23%)
Tgrandiflorum
CL21
Low_complexity
Length of Reads (GP):32952 (0.41%)
Tcacao
CL21
LTR_Copia
Length of Reads (GP):13428 (0.66%)

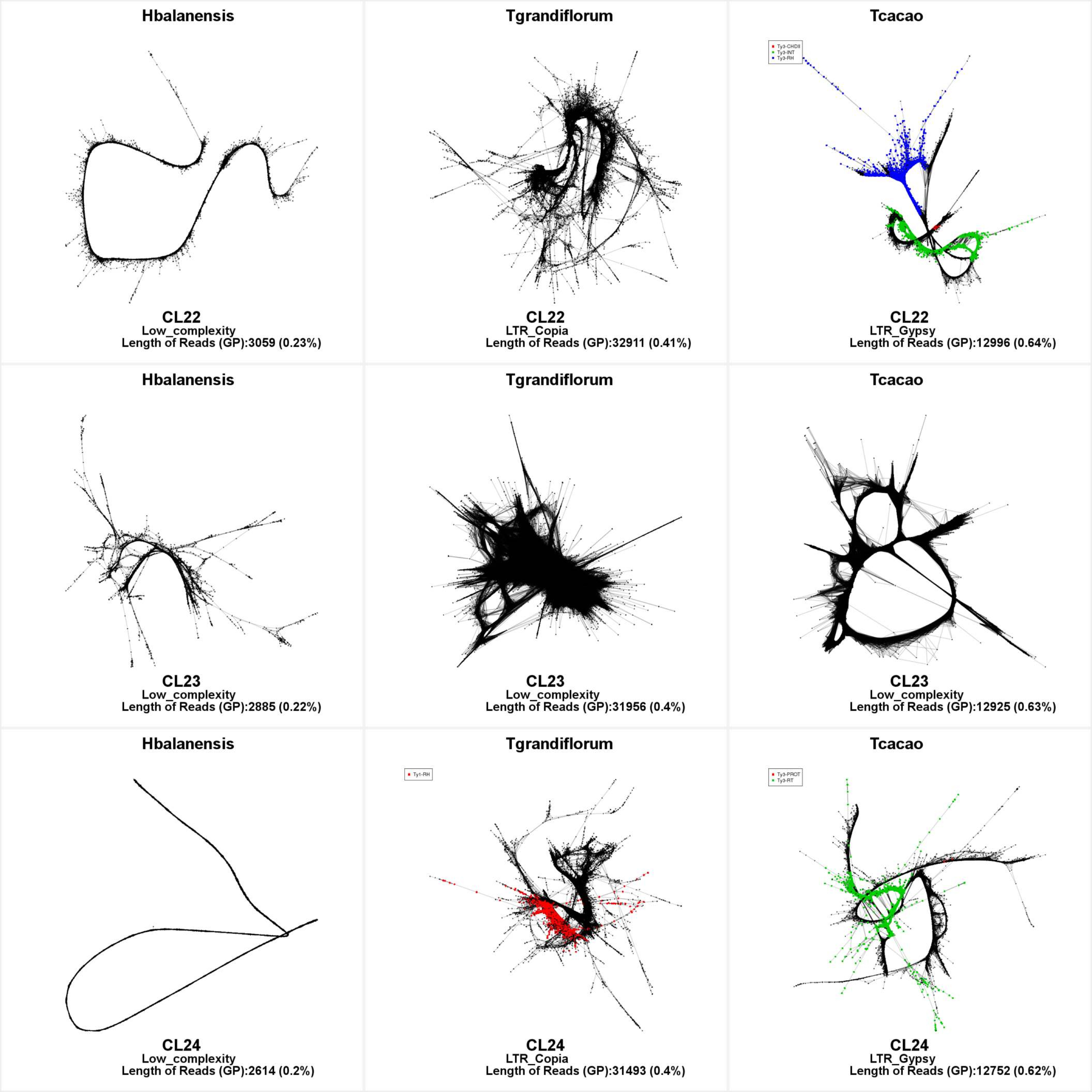
Hbalanensis
CL22
Low_complexity
Length of Reads (GP):3059 (0.23%)
Tgrandiflorum
CL22
LTR_Copia
Length of Reads (GP):32911 (0.41%)
Tcacao
Ty3-CHDII
Ty3-INT
Ty3-RH
CL22
LTR_Gypsy
Length of Reads (GP):12996 (0.64%)
Hbalanensis
CL23
Low_complexity
Length of Reads (GP):2885 (0.22%)
Tgrandiflorum
CL23
Low_complexity
Length of Reads (GP):31956 (0.4%)
Tcacao
CL23
Low_complexity
Length of Reads (GP):12925 (0.63%)
Hbalanensis
CL24
Low_complexity
Length of Reads (GP):2614 (0.2%)
Tgrandiflorum
Ty1-RH
CL24
LTR_Copia
Length of Reads (GP):31493 (0.4%)
Tcacao
Ty3-PROT
Ty3-RT
CL24
LTR_Gypsy
Length of Reads (GP):12752 (0.62%)

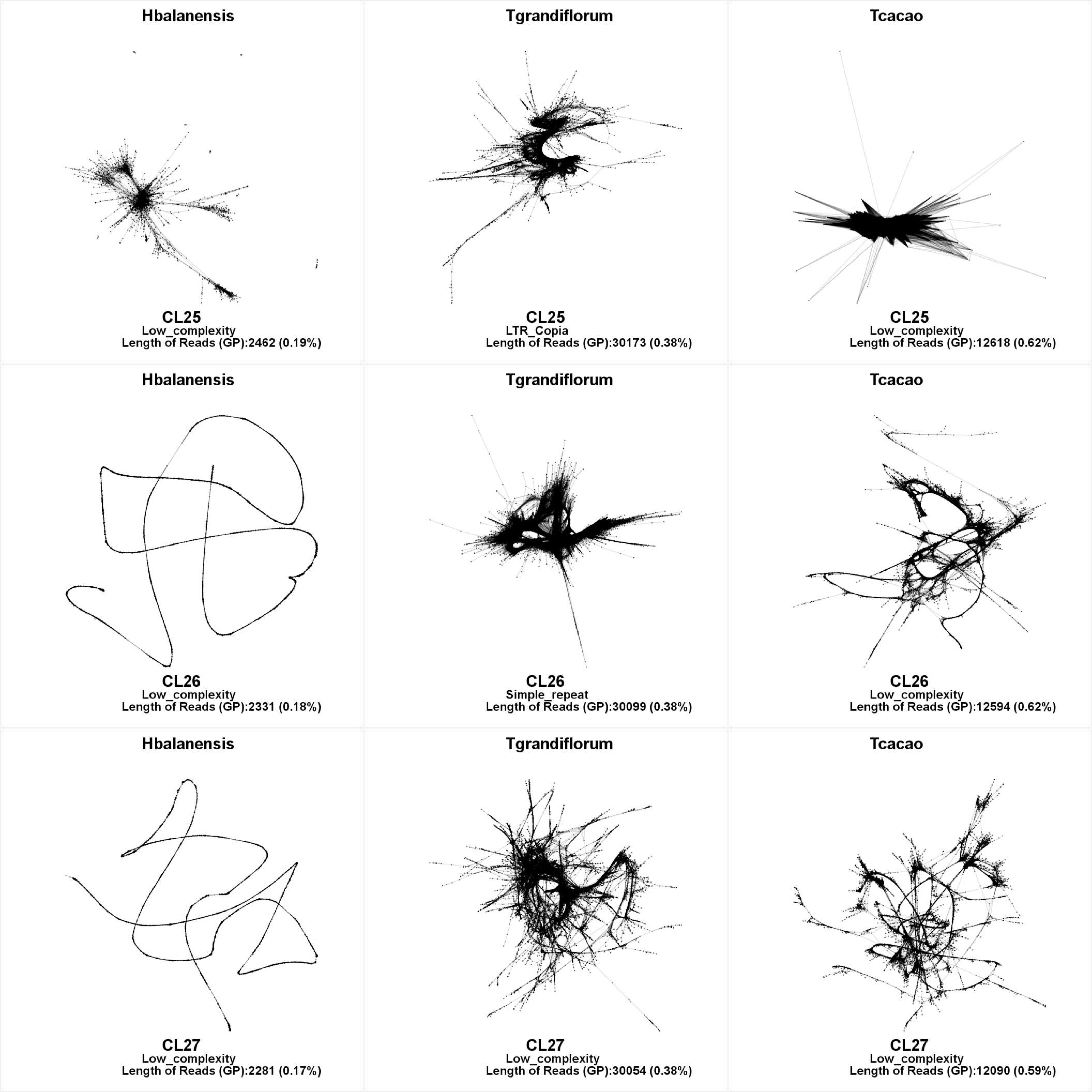
Hbalanensis
CL25
Low_complexity
Length of Reads (GP):2462 (0.19%)
Tgrandiflorum
CL25
LTR_Copia
Length of Reads (GP):30173 (0.38%)
Tcacao
CL25
Low_complexity
Length of Reads (GP):12618 (0.62%)
Hbalanensis
CL26
Low_complexity
Length of Reads (GP):2331 (0.18%)
Tgrandiflorum
CL26
Simple_repeat
Length of Reads (GP):30099 (0.38%)
Tcacao
CL26
Low_complexity
Length of Reads (GP):12594 (0.62%)
Hbalanensis
CL27
Low_complexity
Length of Reads (GP):2281 (0.17%)
Tgrandiflorum
CL27
Low_complexity
Length of Reads (GP):30054 (0.38%)
Tcacao
CL27
Low_complexity
Length of Reads (GP):12090 (0.59%)

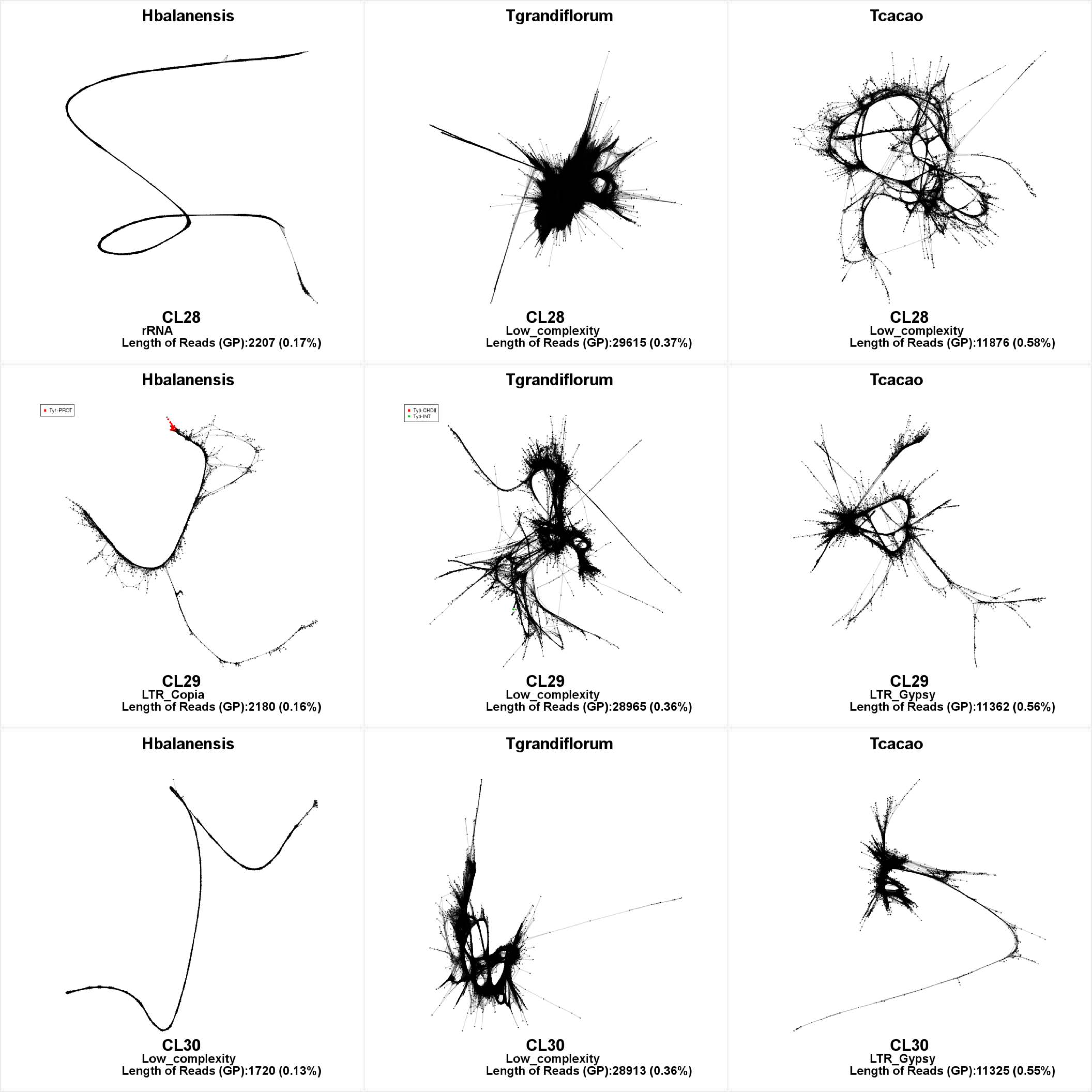
Hbalanensis
CL28
rRNA
Length of Reads (GP):2207 (0.17%)
Tgrandiflorum
CL28
Low_complexity
Length of Reads (GP):29615 (0.37%)
Tcacao
CL28
Low_complexity
Length of Reads (GP):11876 (0.58%)
Hbalanensis
Ty1-PROT
CL29
LTR_Copia
Length of Reads (GP):2180 (0.16%)
Tgrandiflorum
Ty3-CHDII
Ty3-INT
CL29
Low_complexity
Length of Reads (GP):28965 (0.36%)
Tcacao
CL29
LTR_Gypsy
Length of Reads (GP):11362 (0.56%)
Hbalanensis
CL30
Low_complexity
Length of Reads (GP):1720 (0.13%)
Tgrandiflorum
CL30
Low_complexity
Length of Reads (GP):28913 (0.36%)
Tcacao
CL30
LTR_Gypsy
Length of Reads (GP):11325 (0.55%)

Hbalanensis

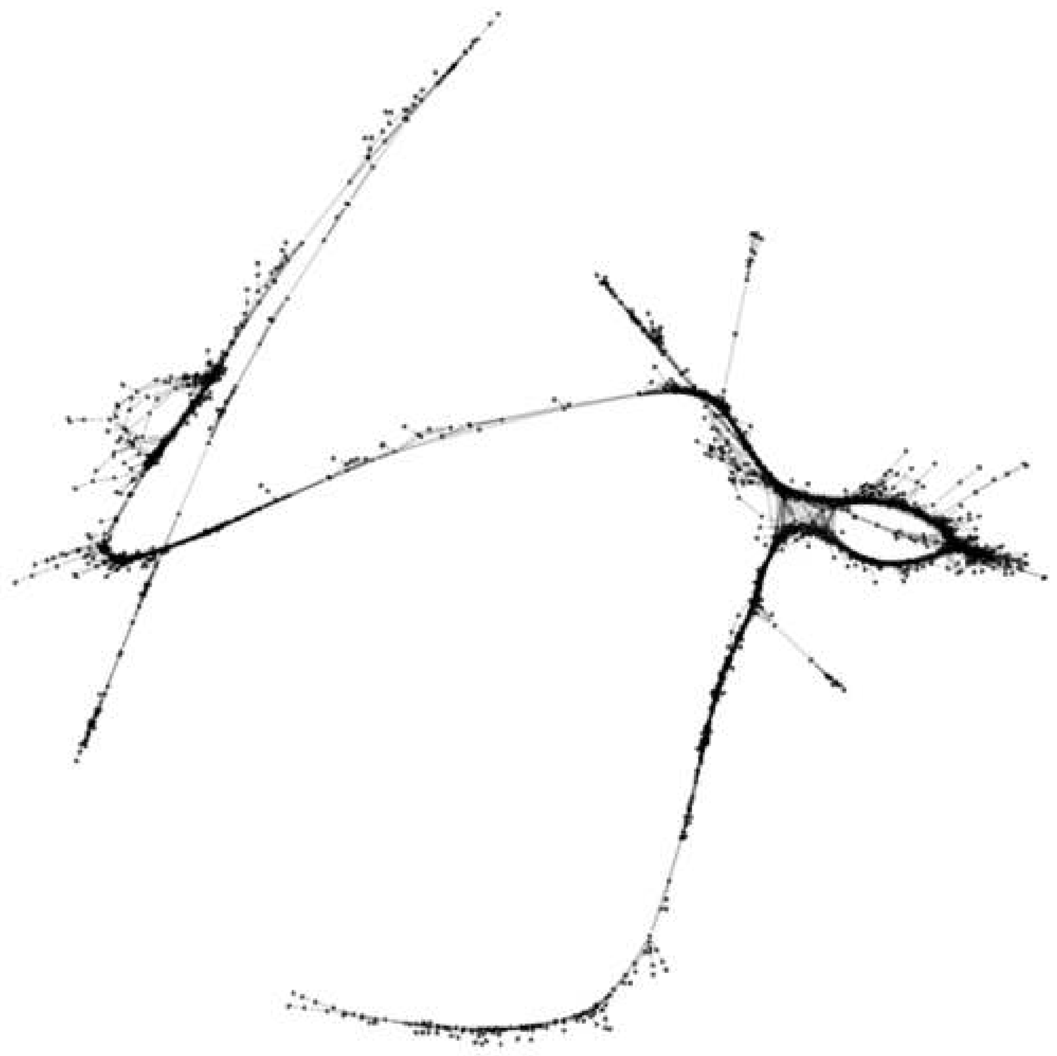

CL31
LTR_Copia
Length of Reads (GP):1684 (0.13%)

Tgrandiflorum

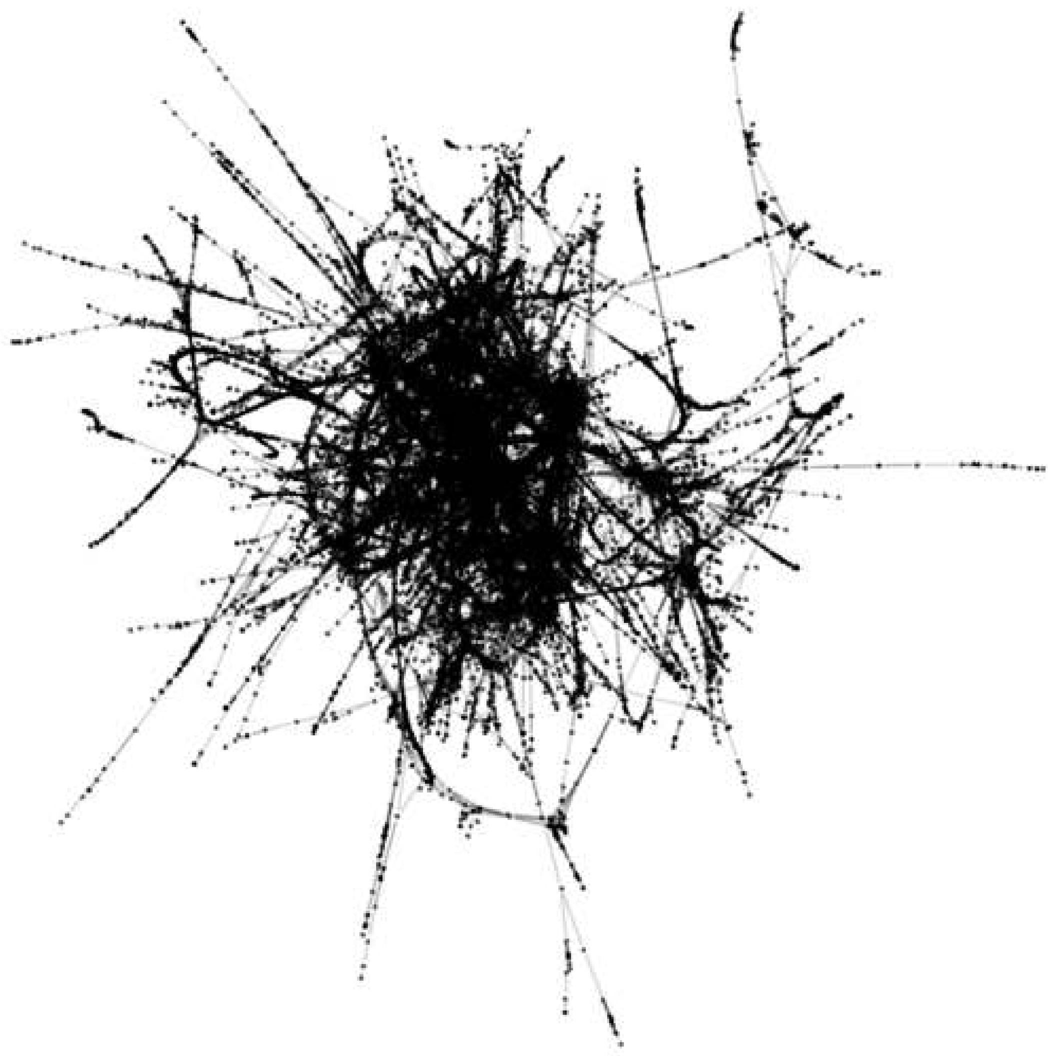

CL31
Low_complexity
Length of Reads (GP):28700 (0.36%)

Tcacao

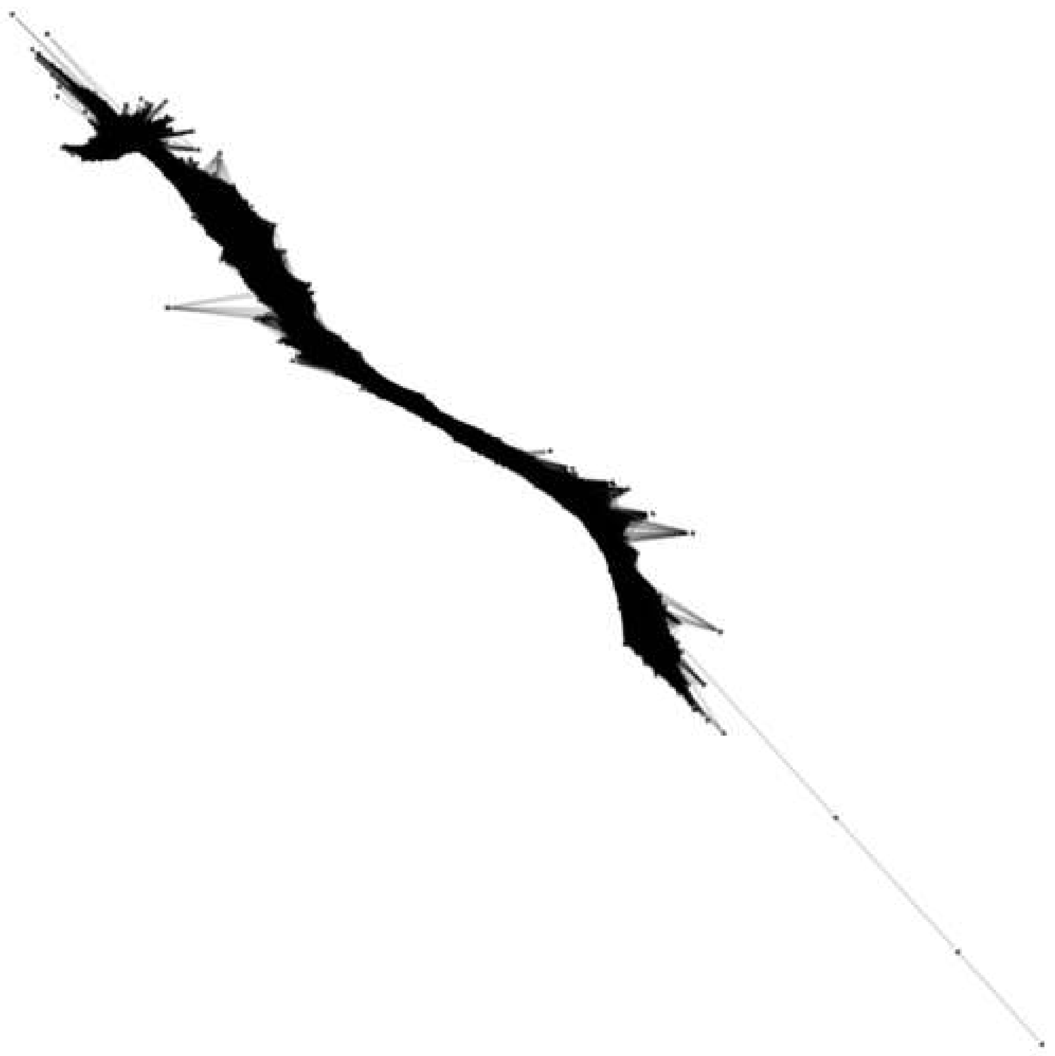

CL31
Low_complexity
Length of Reads (GP):11023 (0.54%)

Hbalanensis

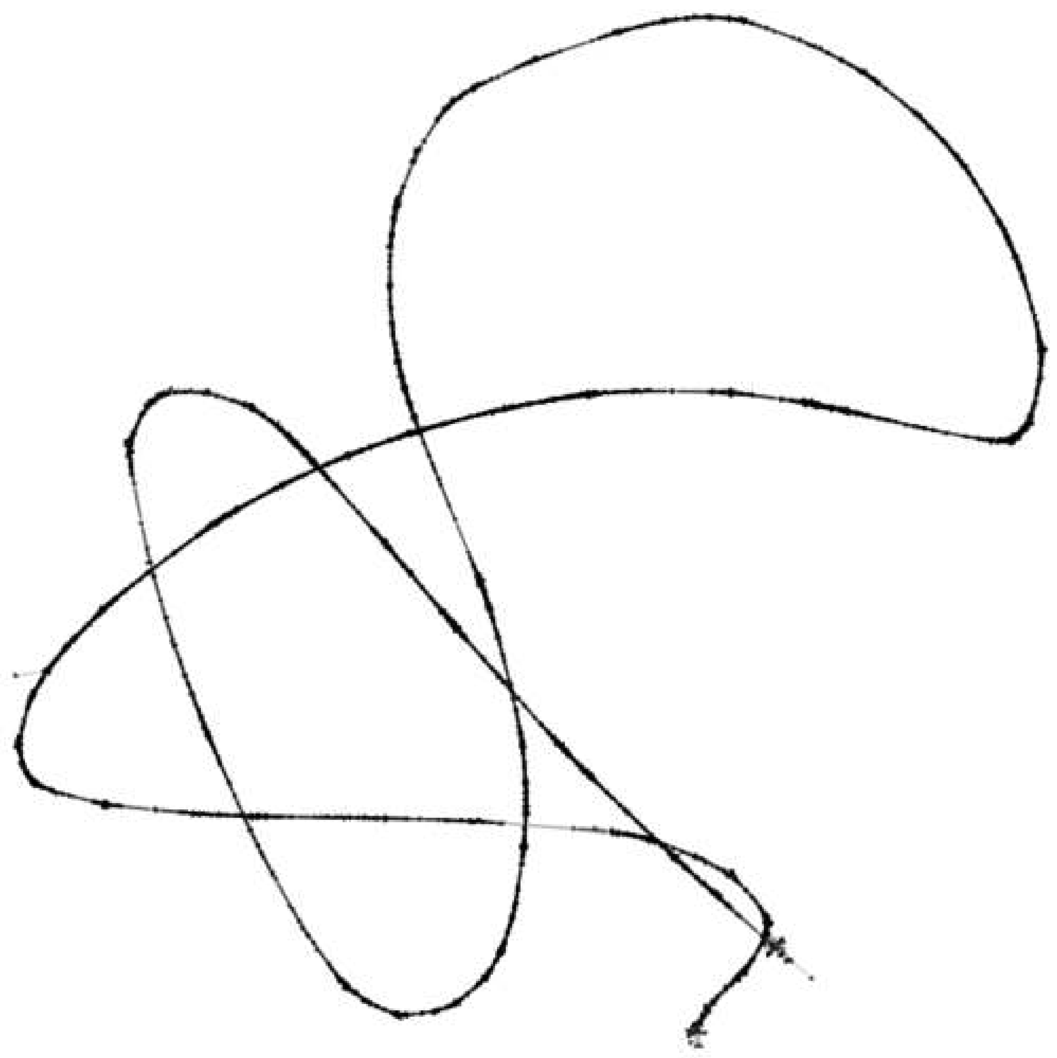

CL32
Low_complexity
Length of Reads (GP):1643 (0.12%)

Tgrandiflorum

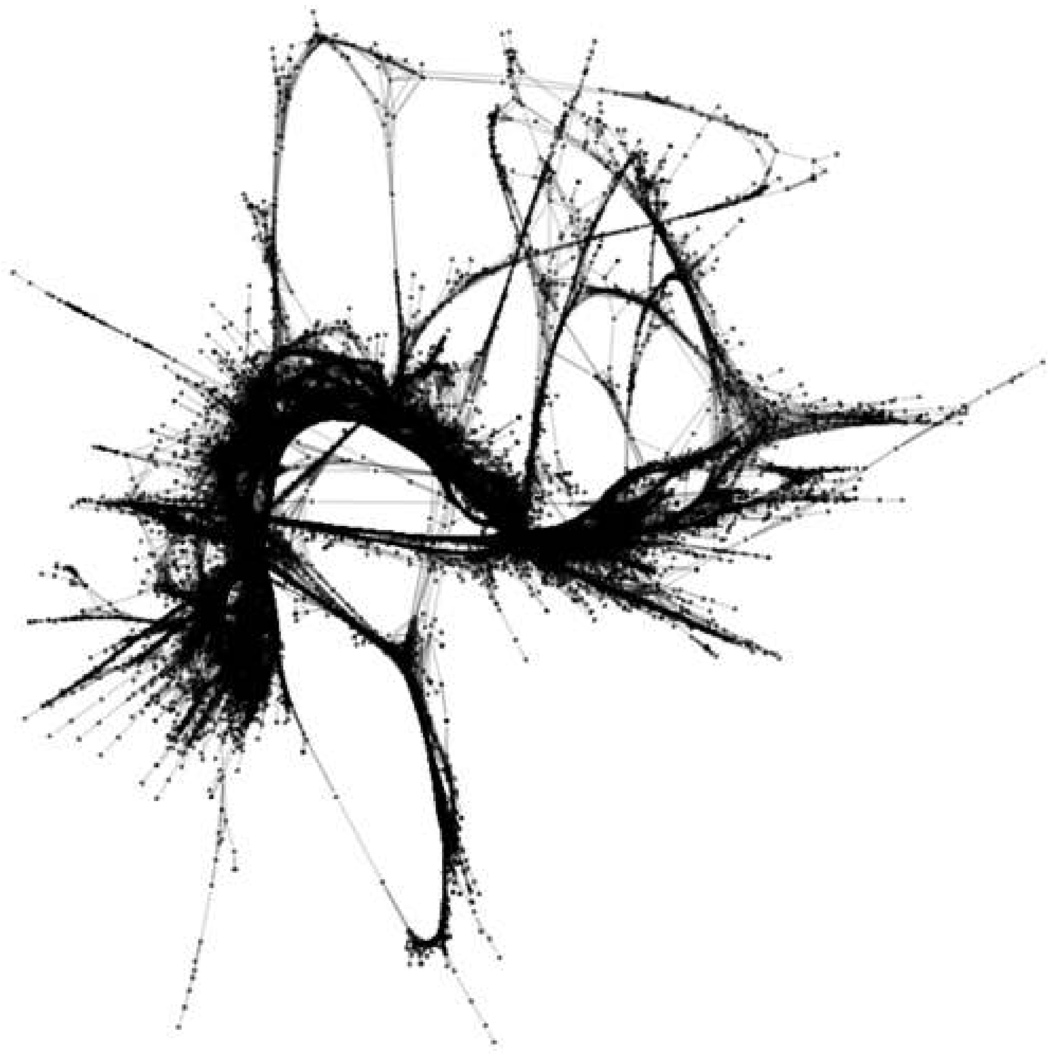

CL32
LTR_Gypsy
Length of Reads (GP):28620 (0.36%)

Tcacao

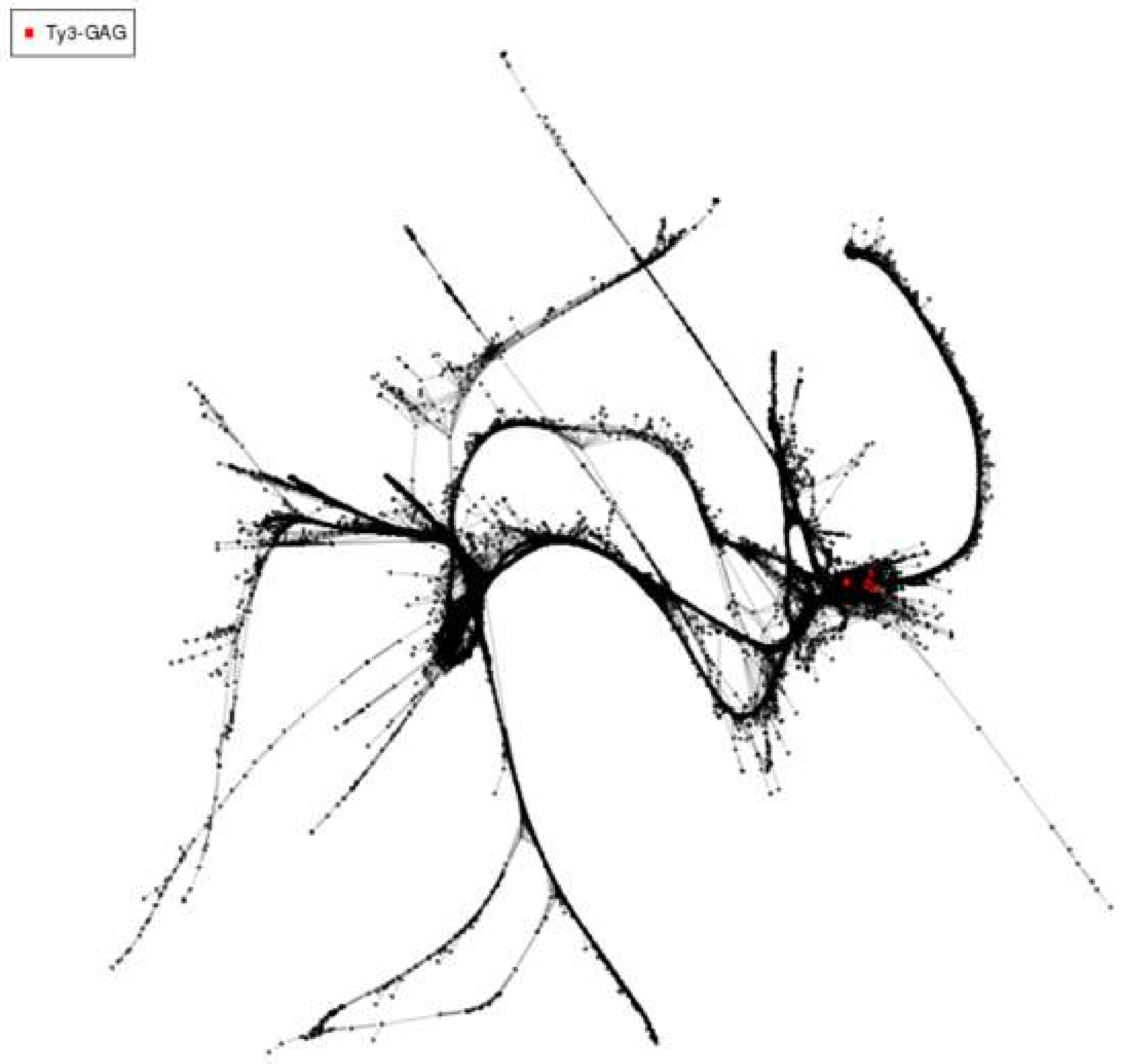

CL32
Simple_repeat
Length of Reads (GP):10719 (0.52%)

Hbalanensis

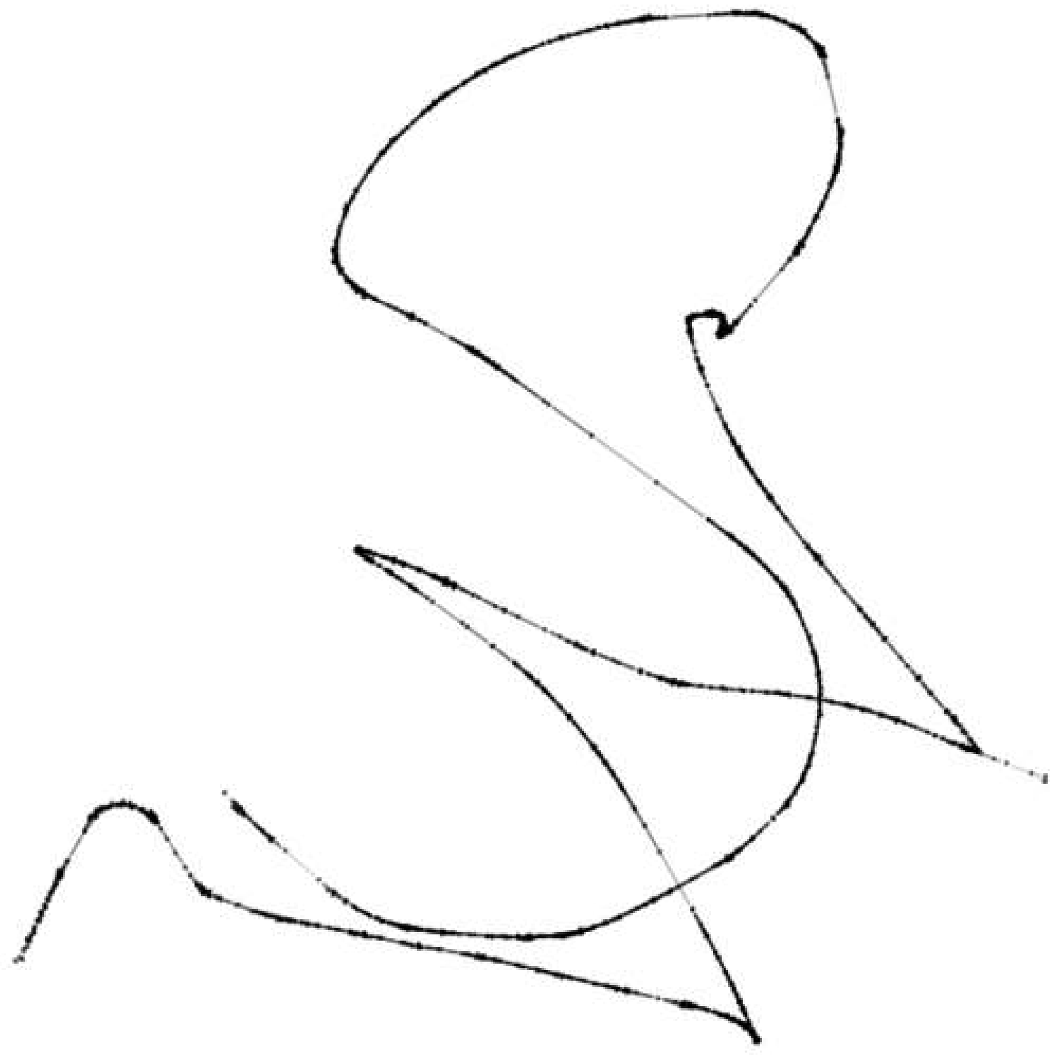

CL33
Low_complexity
Length of Reads (GP):1597 (0.12%)

Tgrandiflorum

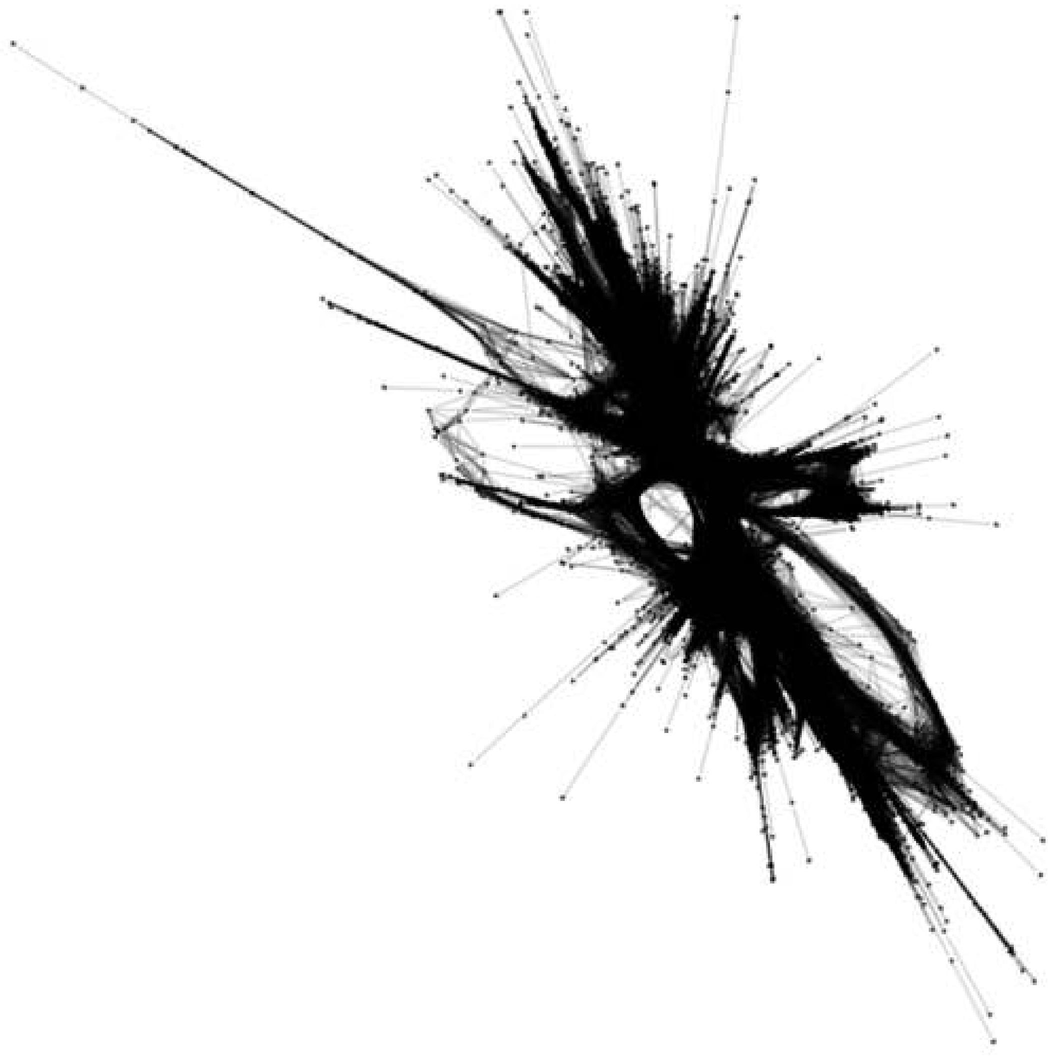

CL33
Low_complexity
Length of Reads (GP):28043 (0.35%)

Tcacao

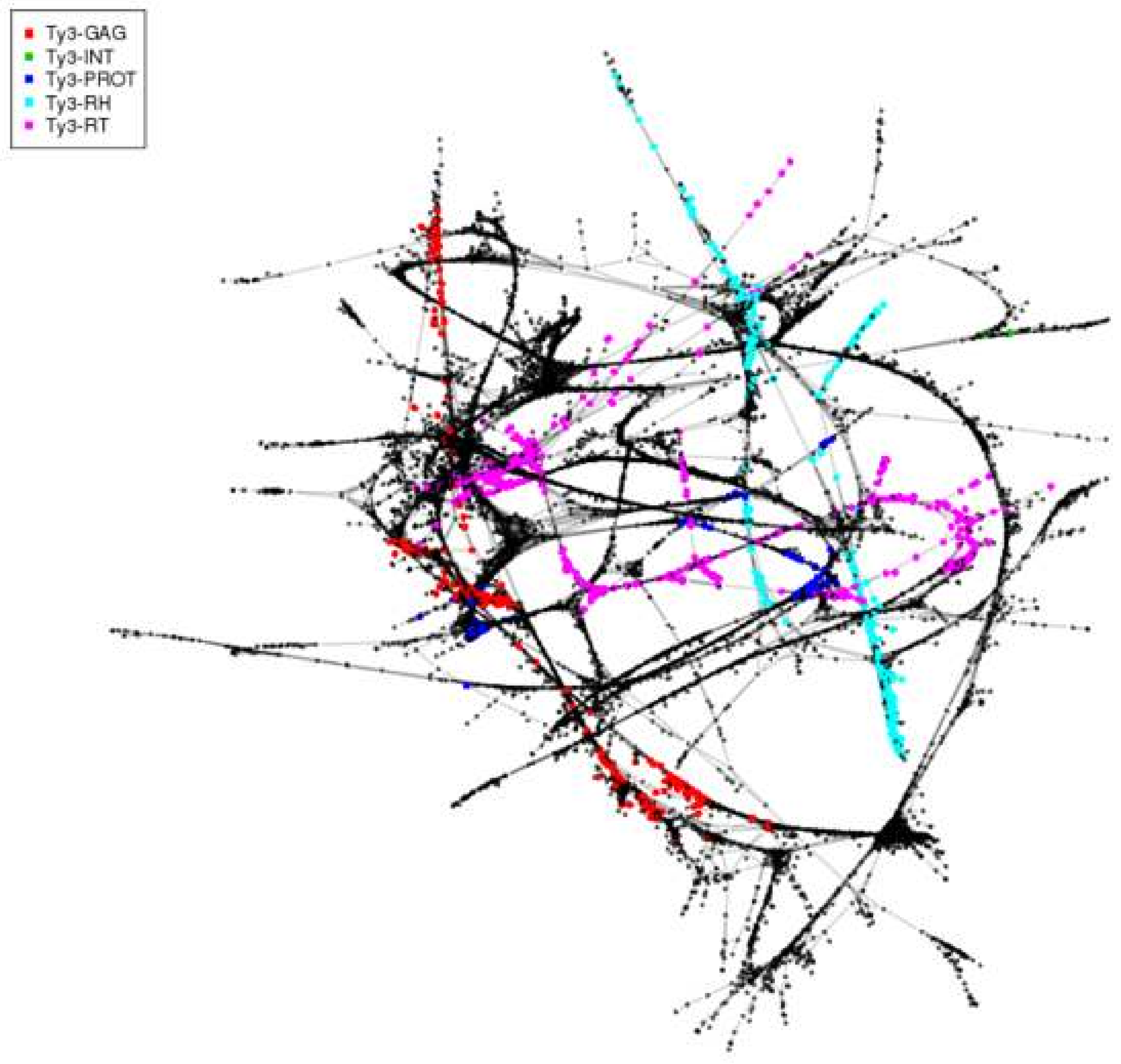

CL33
LTR_Gypsy
Length of Reads (GP):9903 (0.48%)

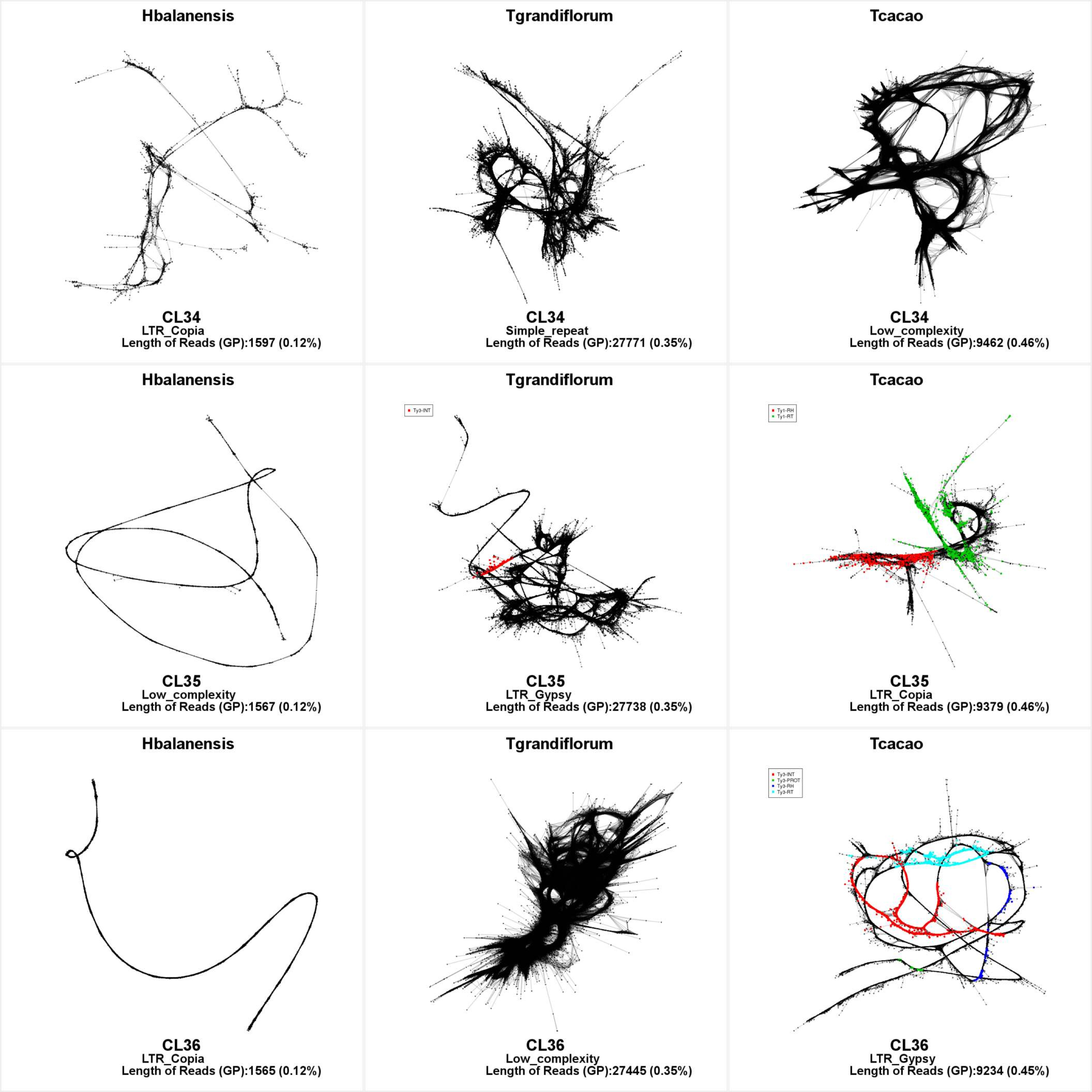
Hbalanensis
CL34
LTR_Copia
Length of Reads (GP):1597 (0.12%)
Tgrandiflorum
CL34
Simple_repeat
Length of Reads (GP):27771 (0.35%)
Tcacao
CL34
Low_complexity
Length of Reads (GP):9462 (0.46%)
Hbalanensis
CL35
Low_complexity
Length of Reads (GP):1567 (0.12%)
Tgrandiflorum
Ty3-INT
CL35
LTR_Gypsy
Length of Reads (GP):27738 (0.35%)
Tcacao
Ty1-RH
Ty1-RT
CL35
LTR_Copia
Length of Reads (GP):9379 (0.46%)
Hbalanensis
CL36
LTR_Copia
Length of Reads (GP):1565 (0.12%)
Tgrandiflorum
CL36
Low_complexity
Length of Reads (GP):27445 (0.35%)
Tcacao
Ty3-INT
Ty3-PROT
Ty3-RH
Ty3-RT
CL36
LTR_Gypsy
Length of Reads (GP):9234 (0.45%)

Hbalanensis

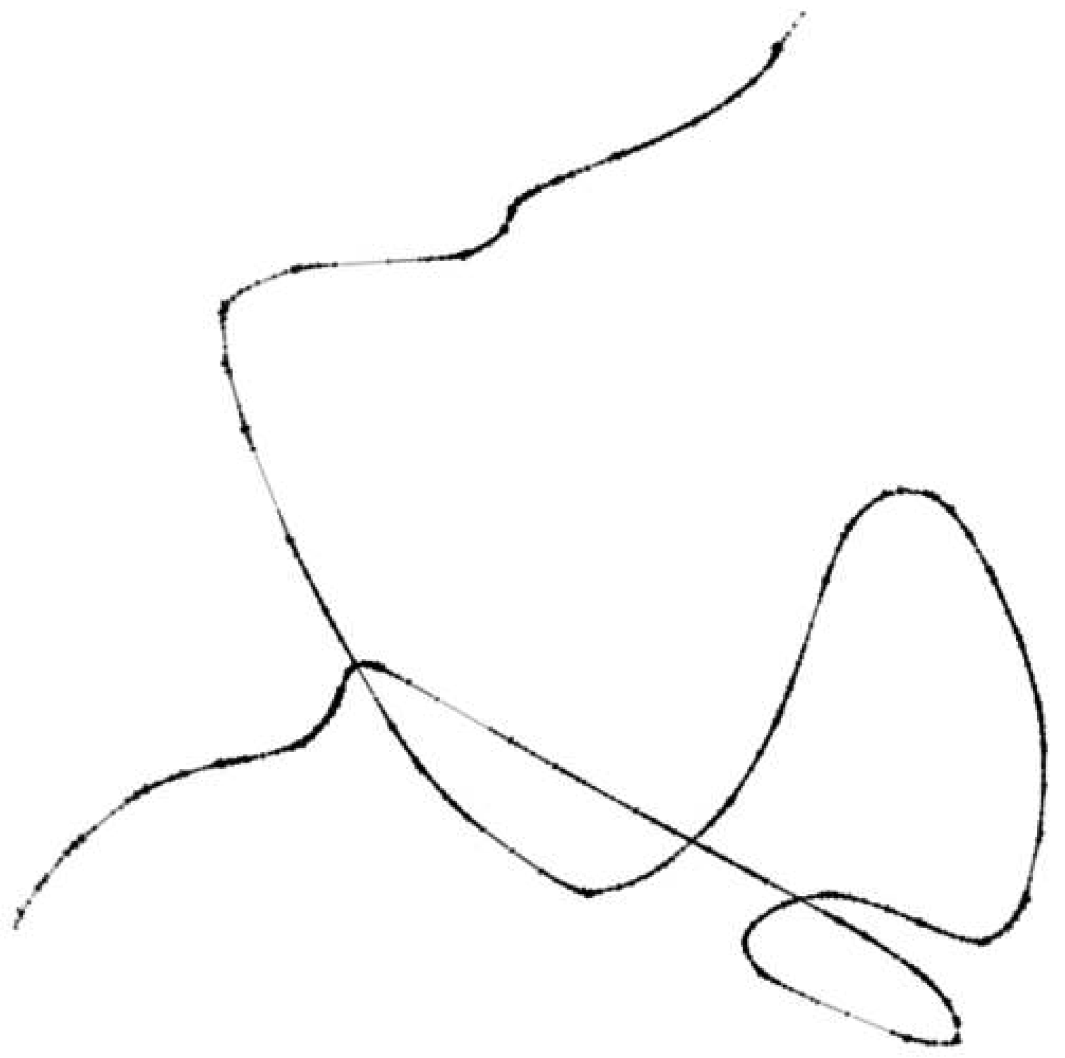

CL37
LTR_Copia
Length of Reads (GP):1541 (0.12%)

Tgrandiflorum

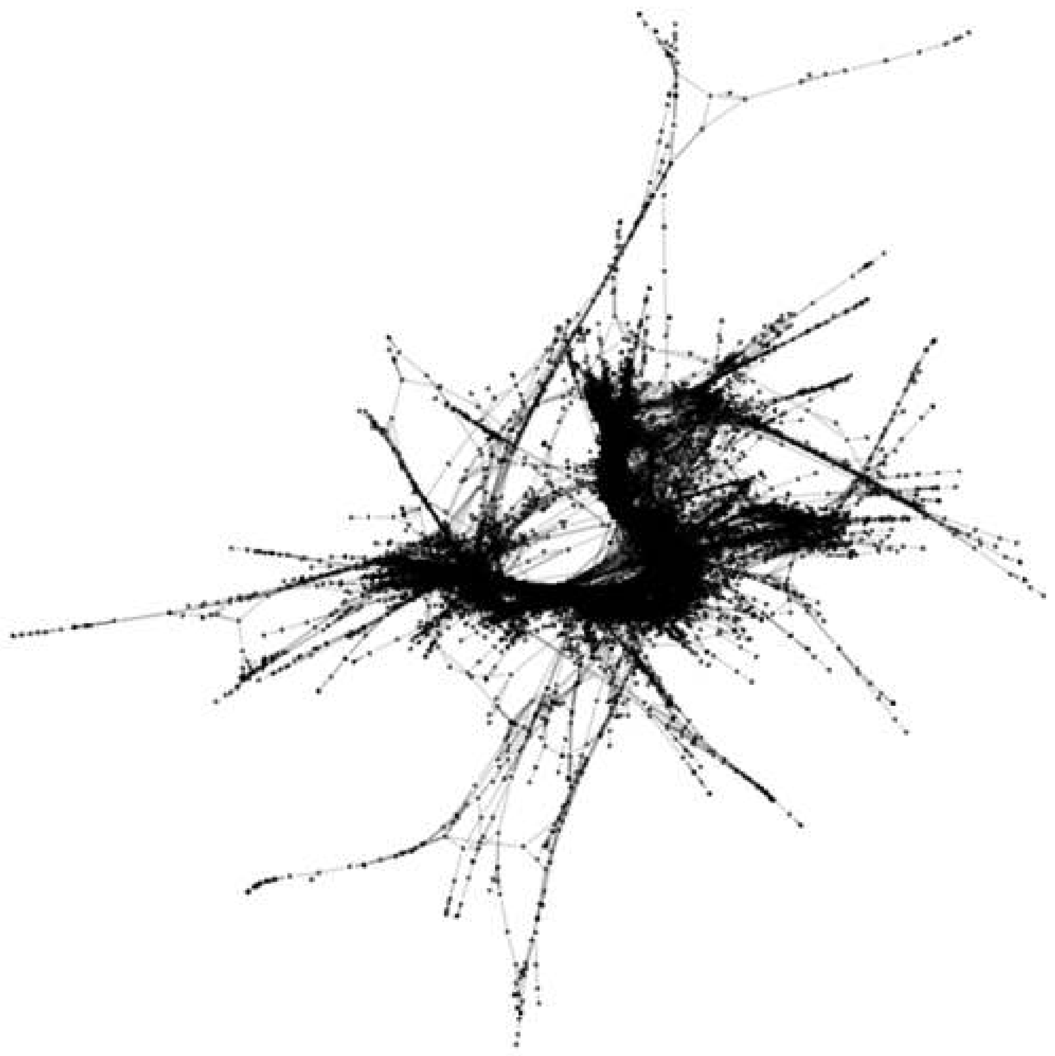

CL37
LTR_Copia
Length of Reads (GP):27364 (0.34%)

Tcacao

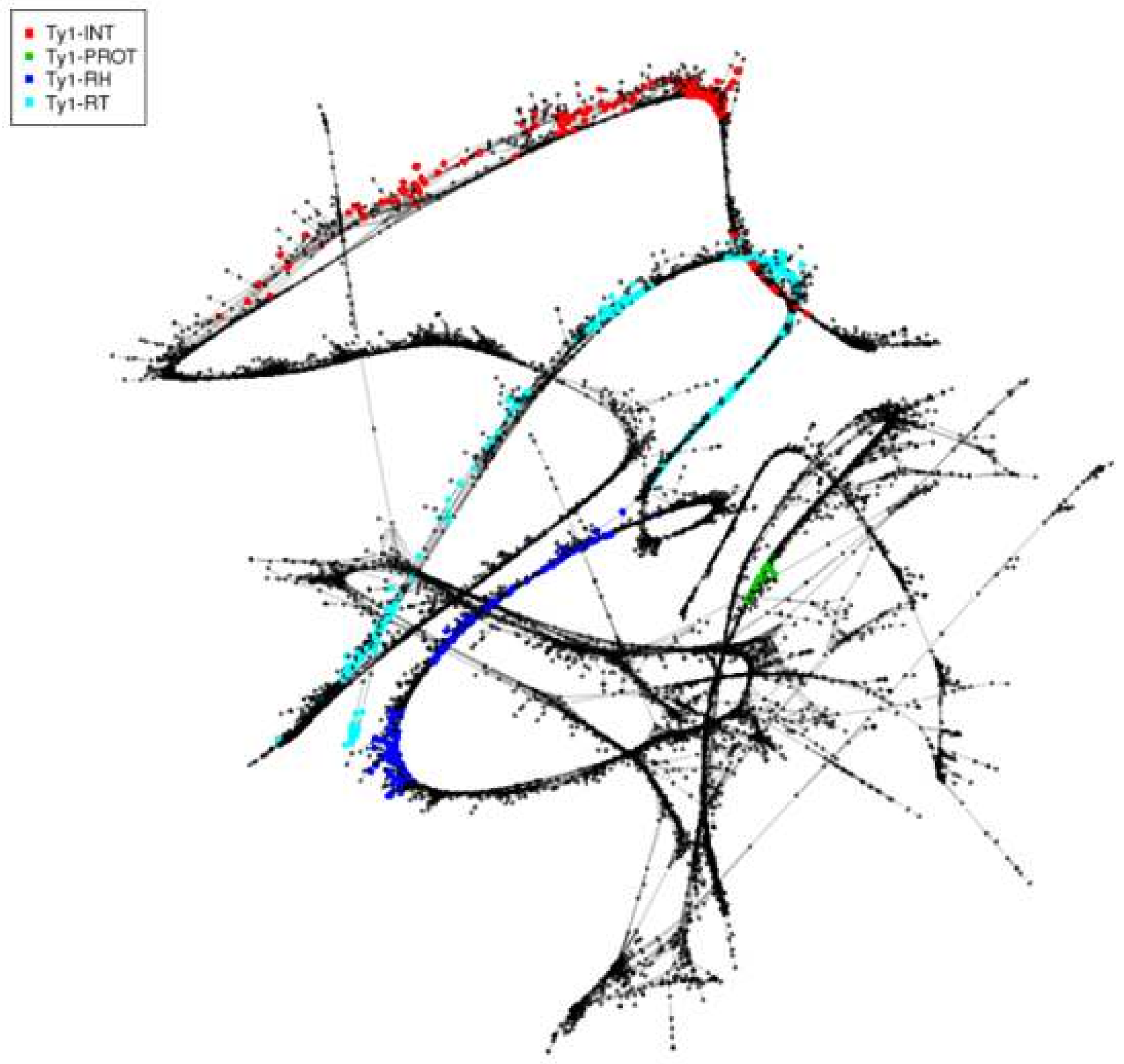

CL37
LTR_Copia
Length of Reads (GP):8710 (0.43%)

Hbalanensis

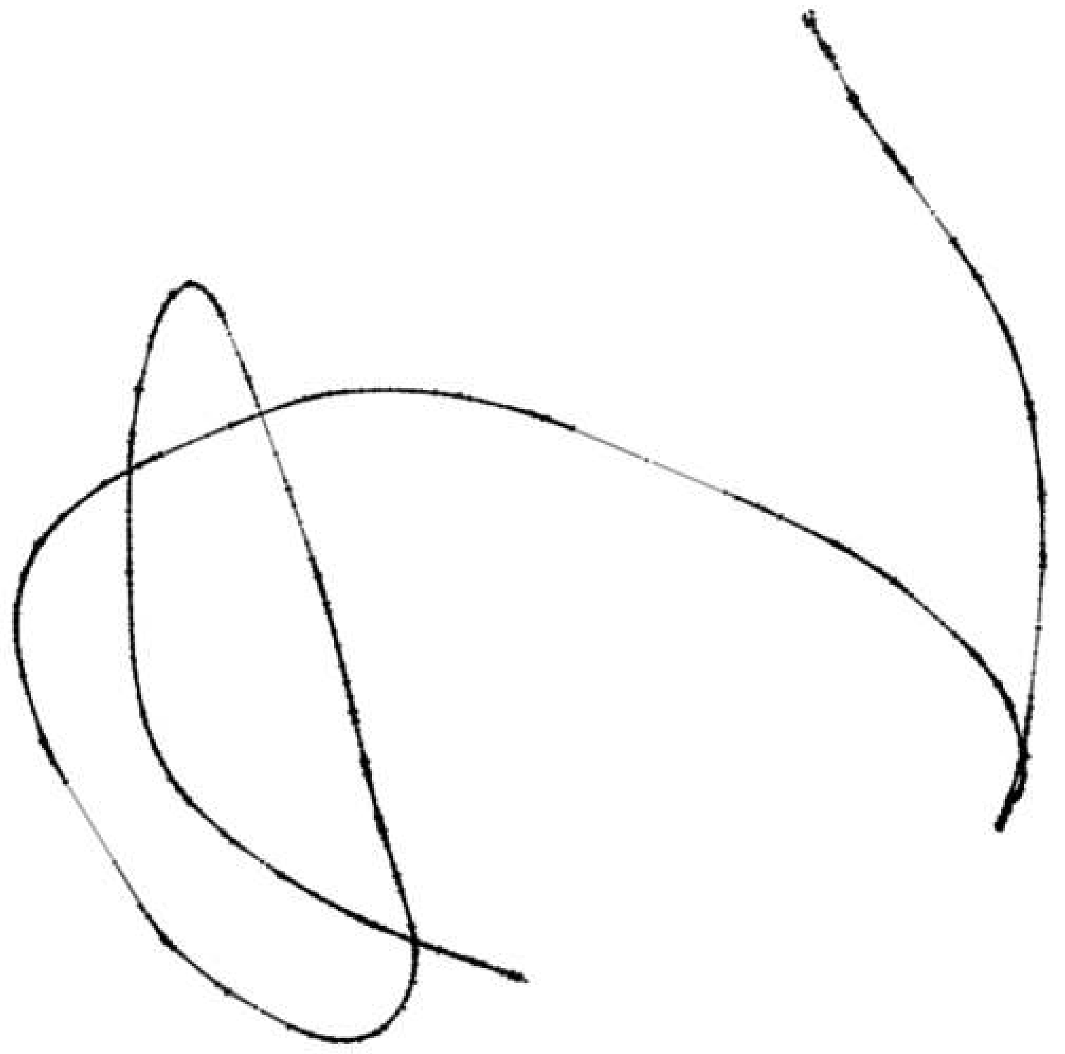

CL38
Low_complexity
Length of Reads (GP):1508 (0.11%)

Tgrandiflorum

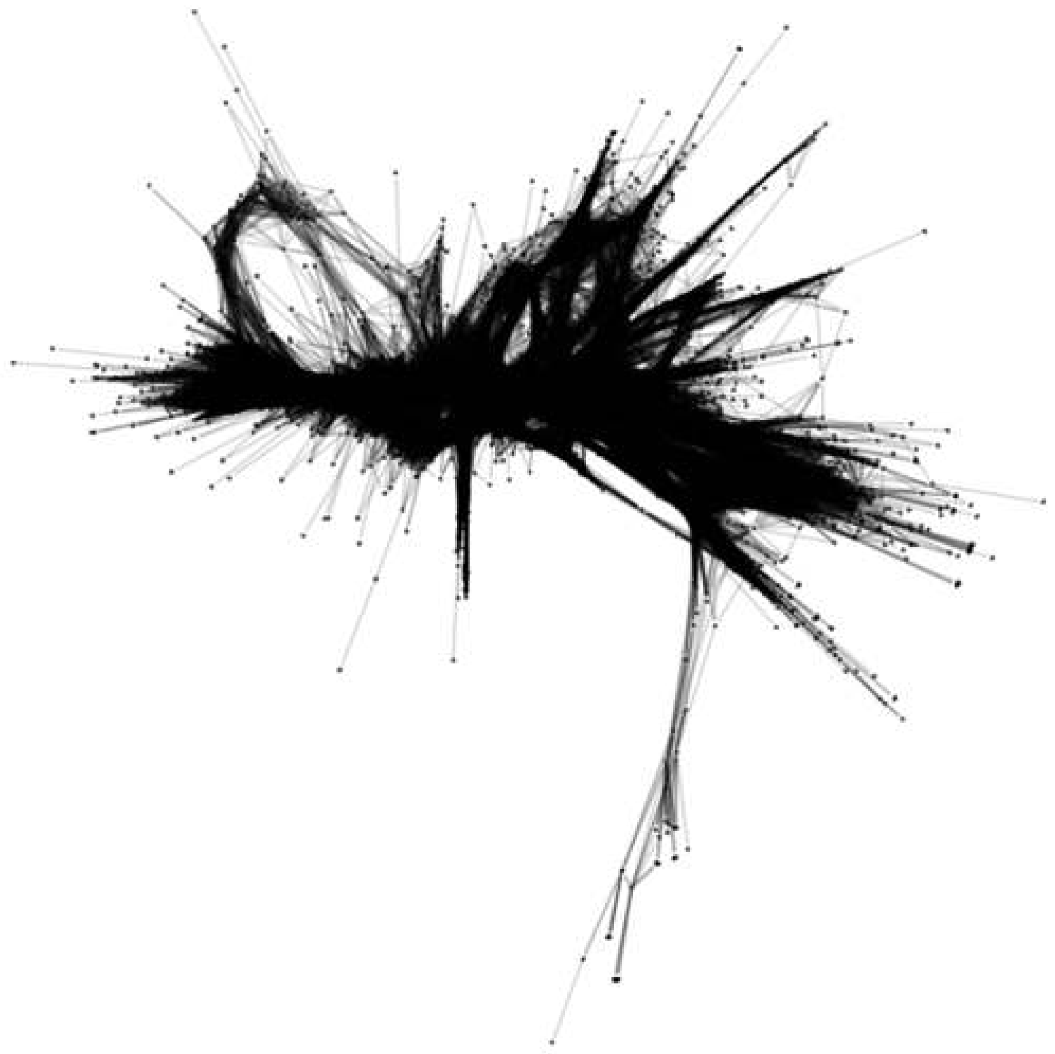

CL38
Low_complexity
Length of Reads (GP):27300 (0.34%)

Tcacao

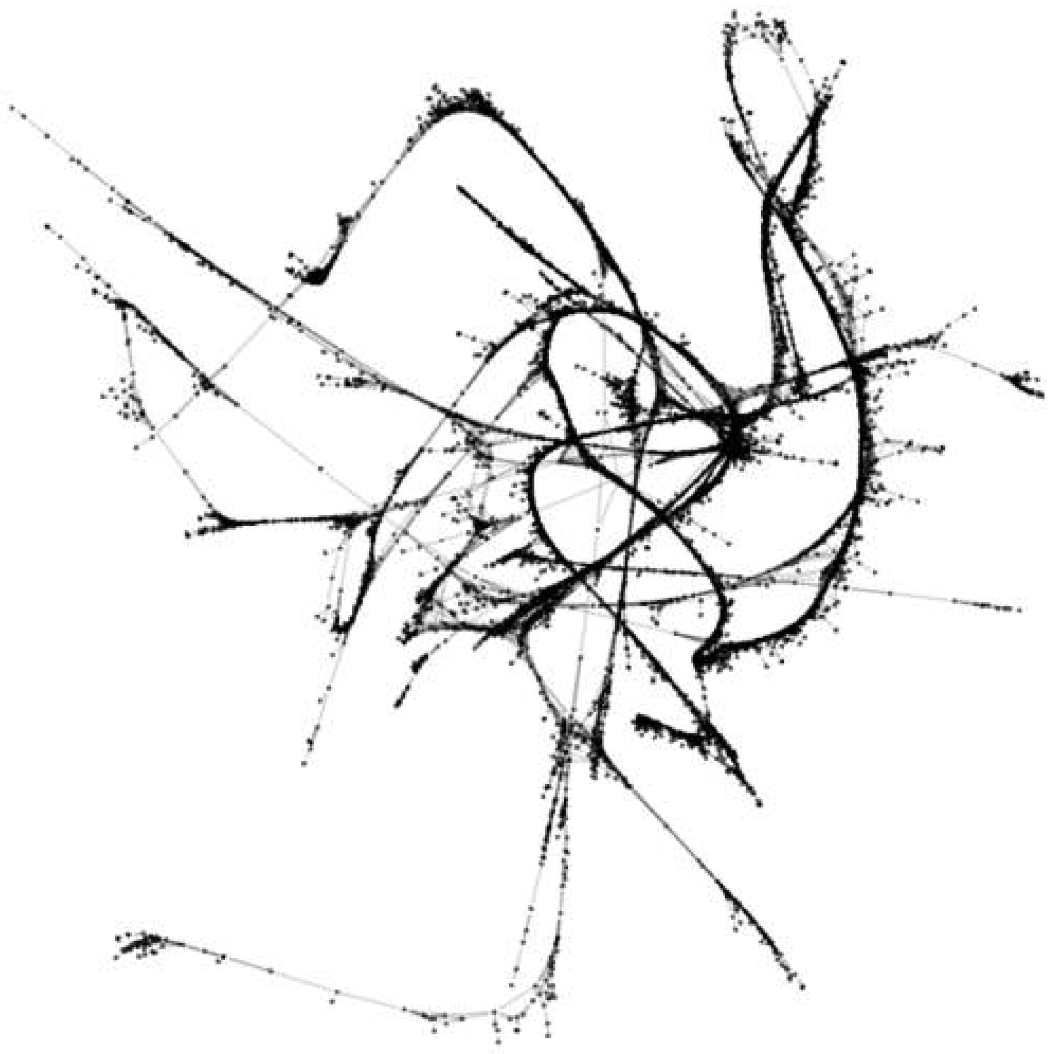

CL38
DNA_MULE_MuDR
Length of Reads (GP):8456 (0.41%)

Hbalanensis

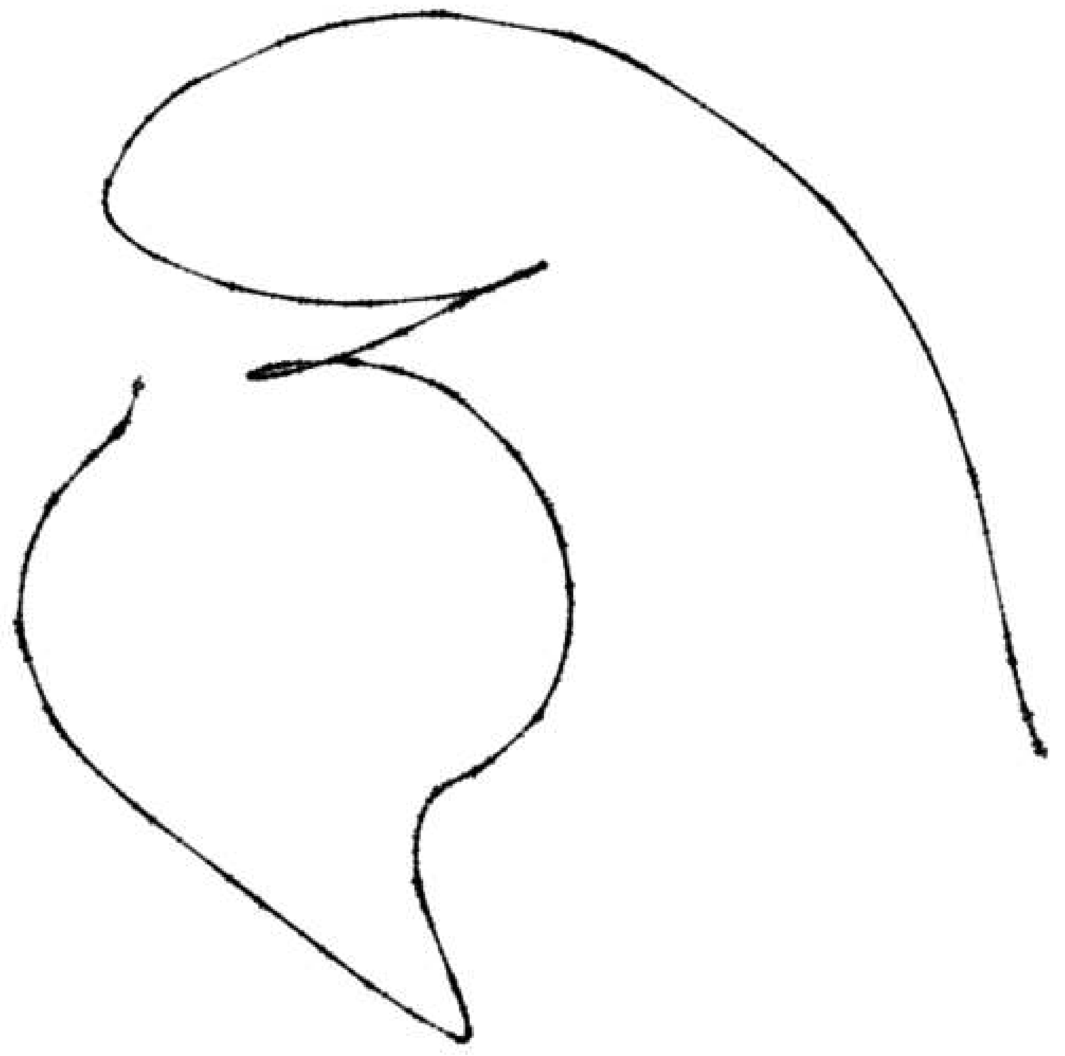

CL39
LTR_Copia
Length of Reads (GP):1401 (0.11%)

Tgrandiflorum

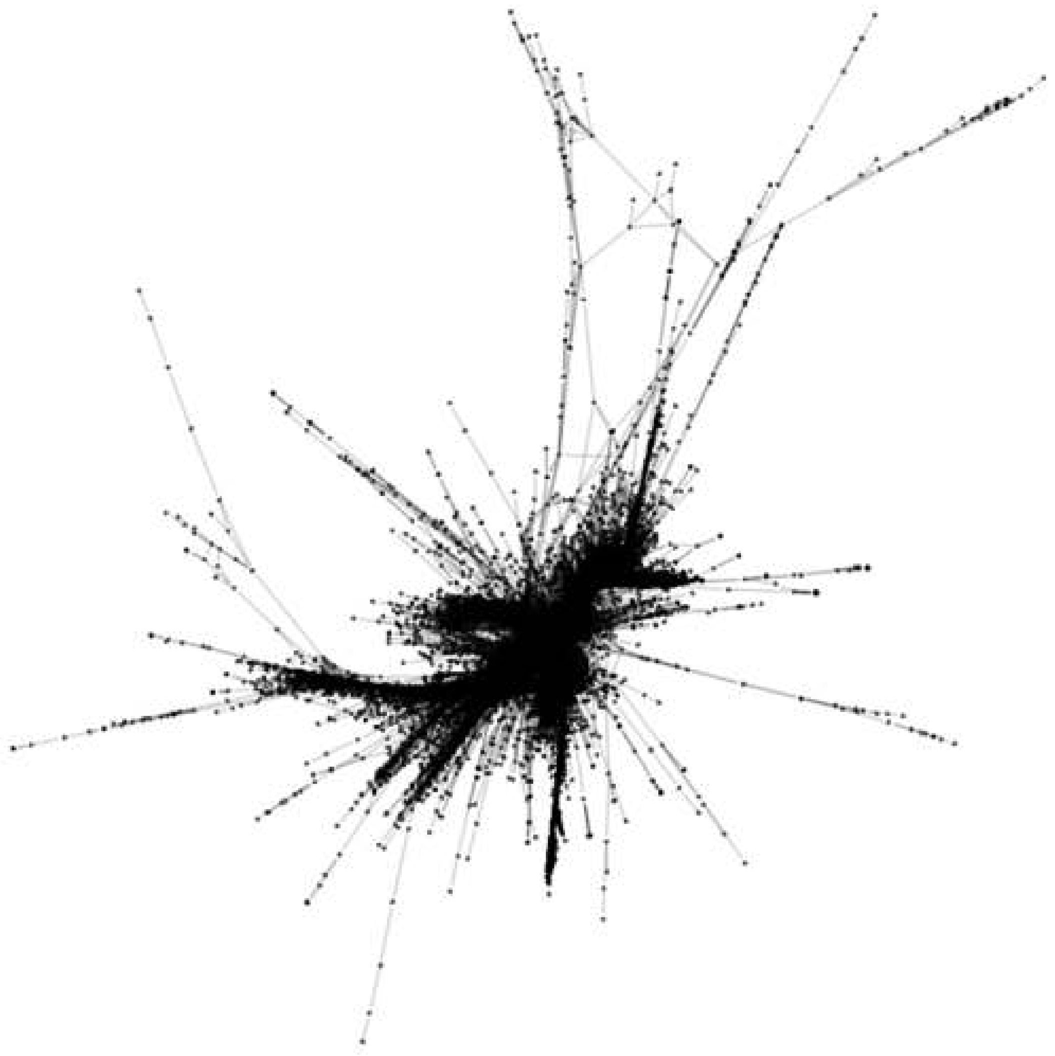

CL39
LTR_Gypsy
Length of Reads (GP):27092 (0.34%)

Tcacao

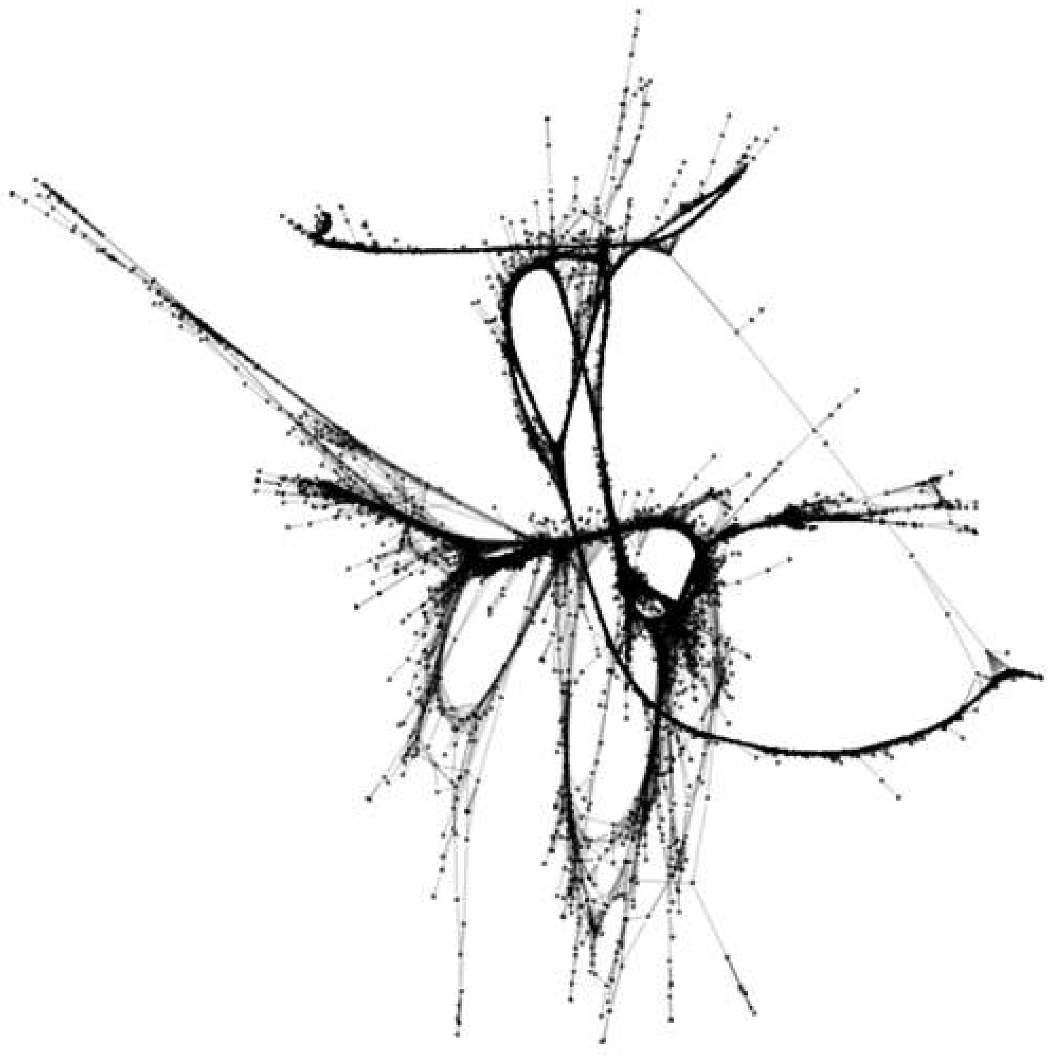

CL39
Low_complexity
Length of Reads (GP):8440 (0.41%)

Hbalanensis

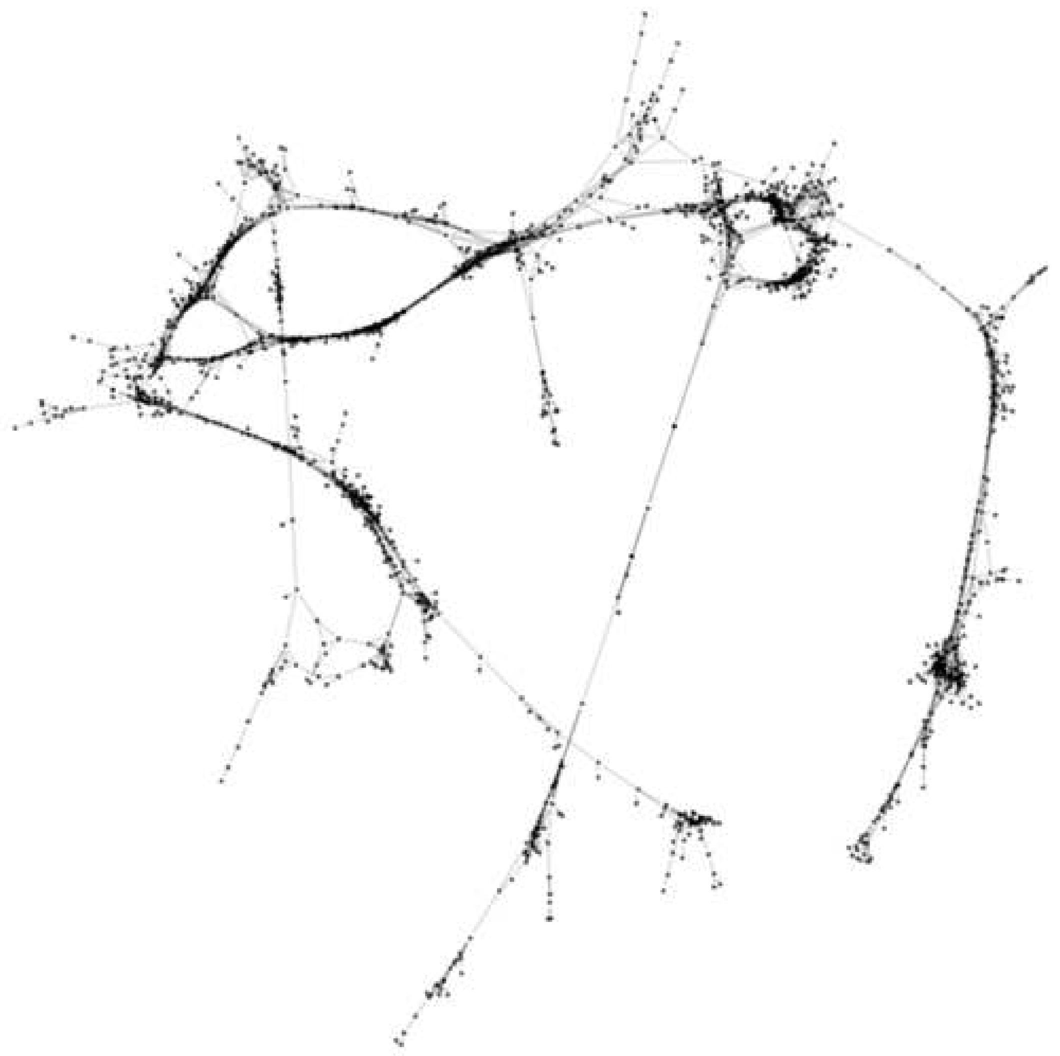

CL40
Low_complexity
Length of Reads (GP):1393 (0.11%)

Tgrandiflorum

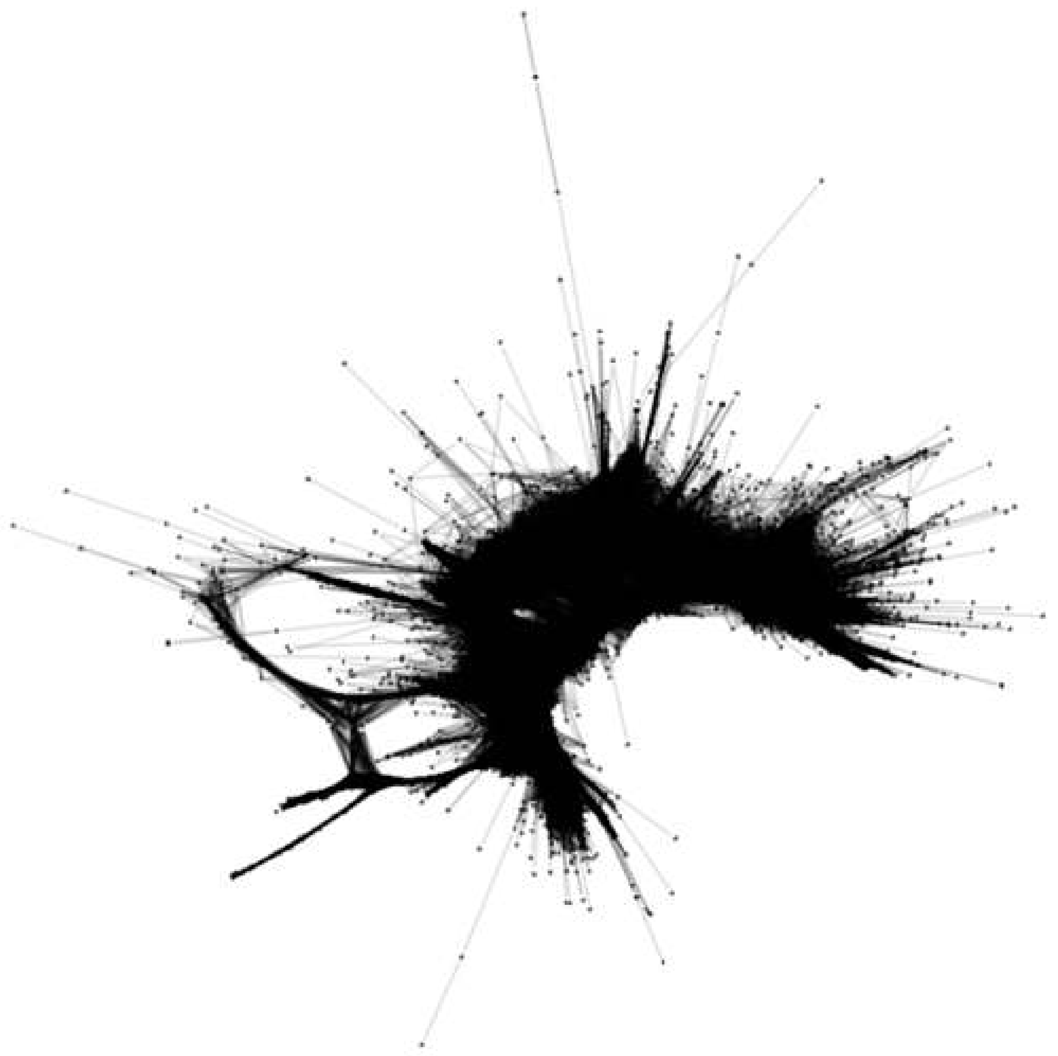

CL40
LTR_Gypsy
Length of Reads (GP):26166 (0.33%)

Tcacao

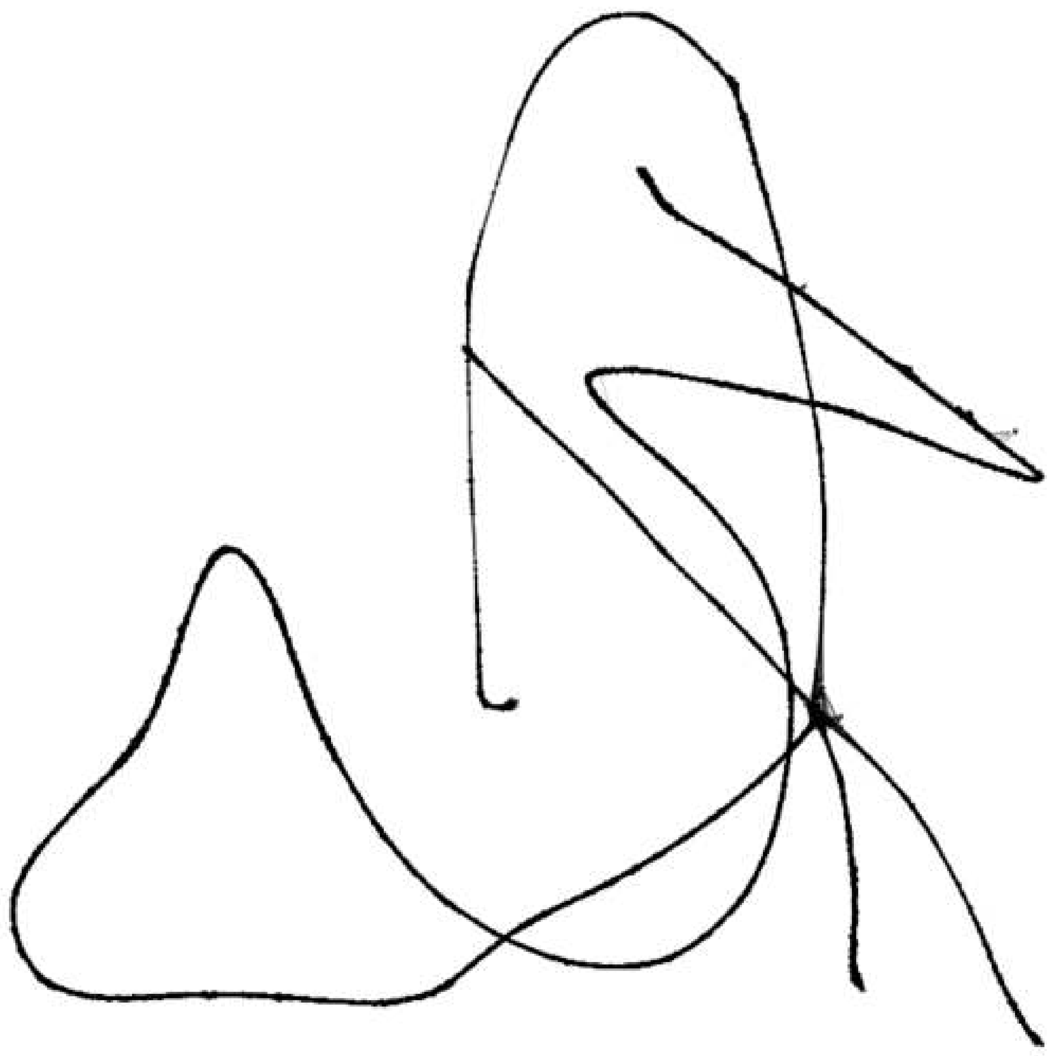

CL40
Low_complexity
Length of Reads (GP):8218 (0.4%)

Hbalanensis

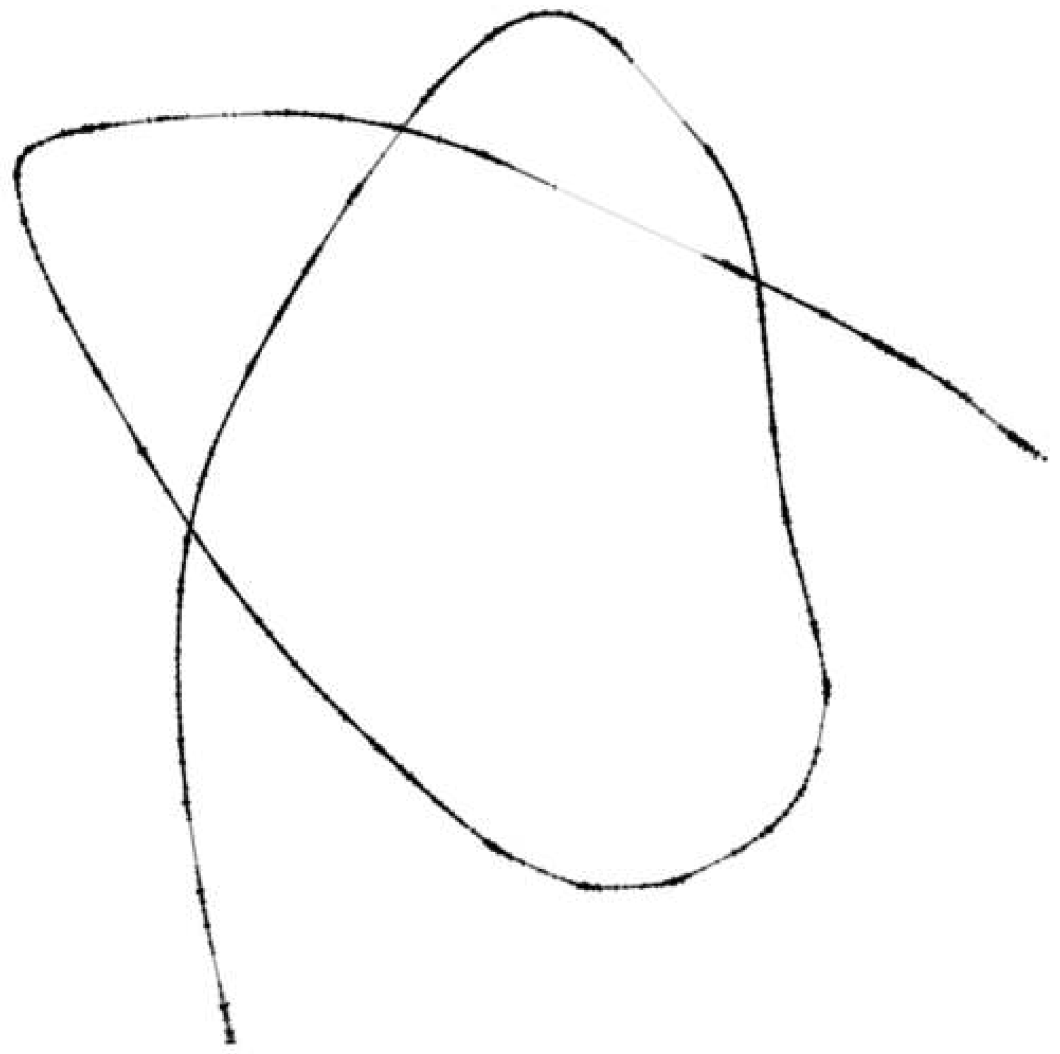

CL41
Low_complexity
Length of Reads (GP):1375 (0.1%)

Tgrandiflorum

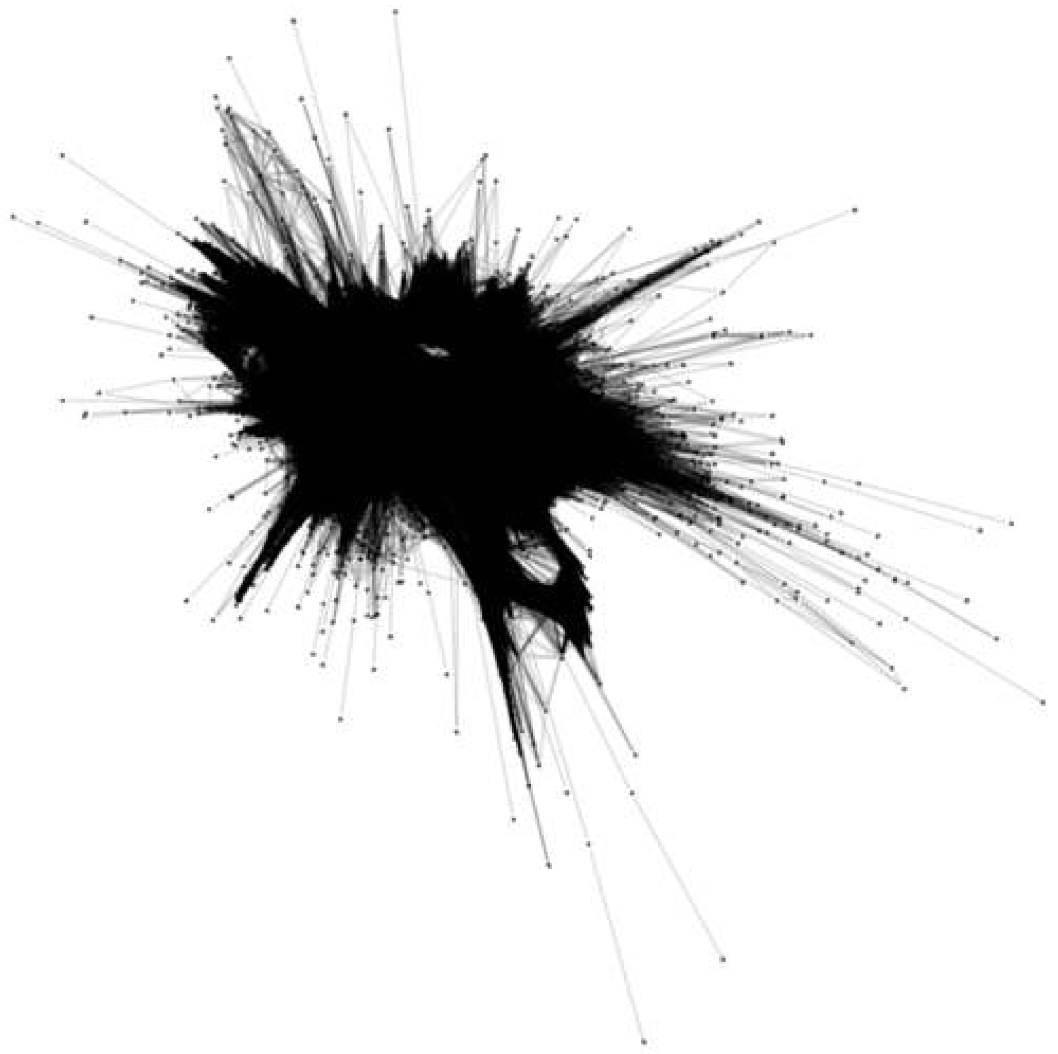

CL41
LTR_Gypsy
Length of Reads (GP):26090 (0.33%)

Tcacao

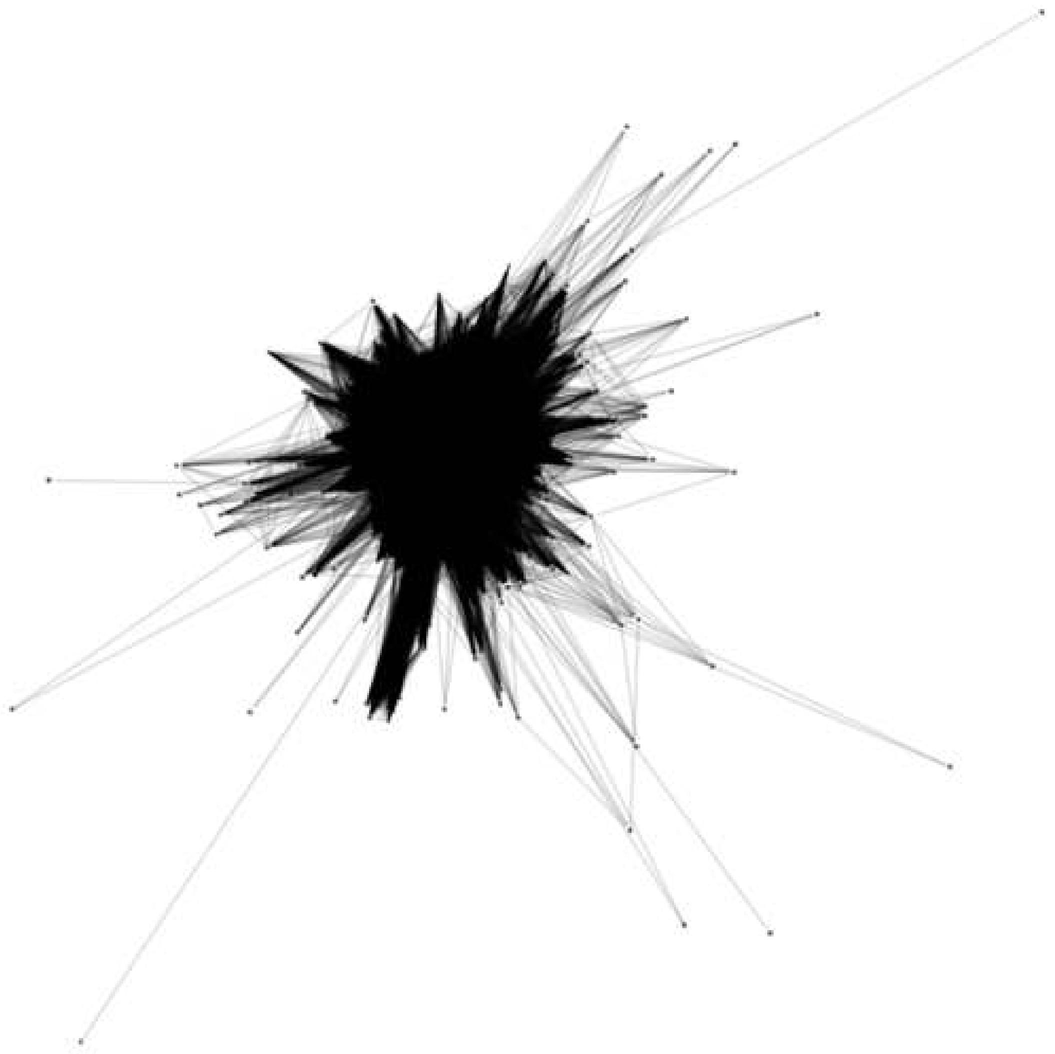

CL41
Low_complexity
Length of Reads (GP):7799 (0.38%)

Hbalanensis

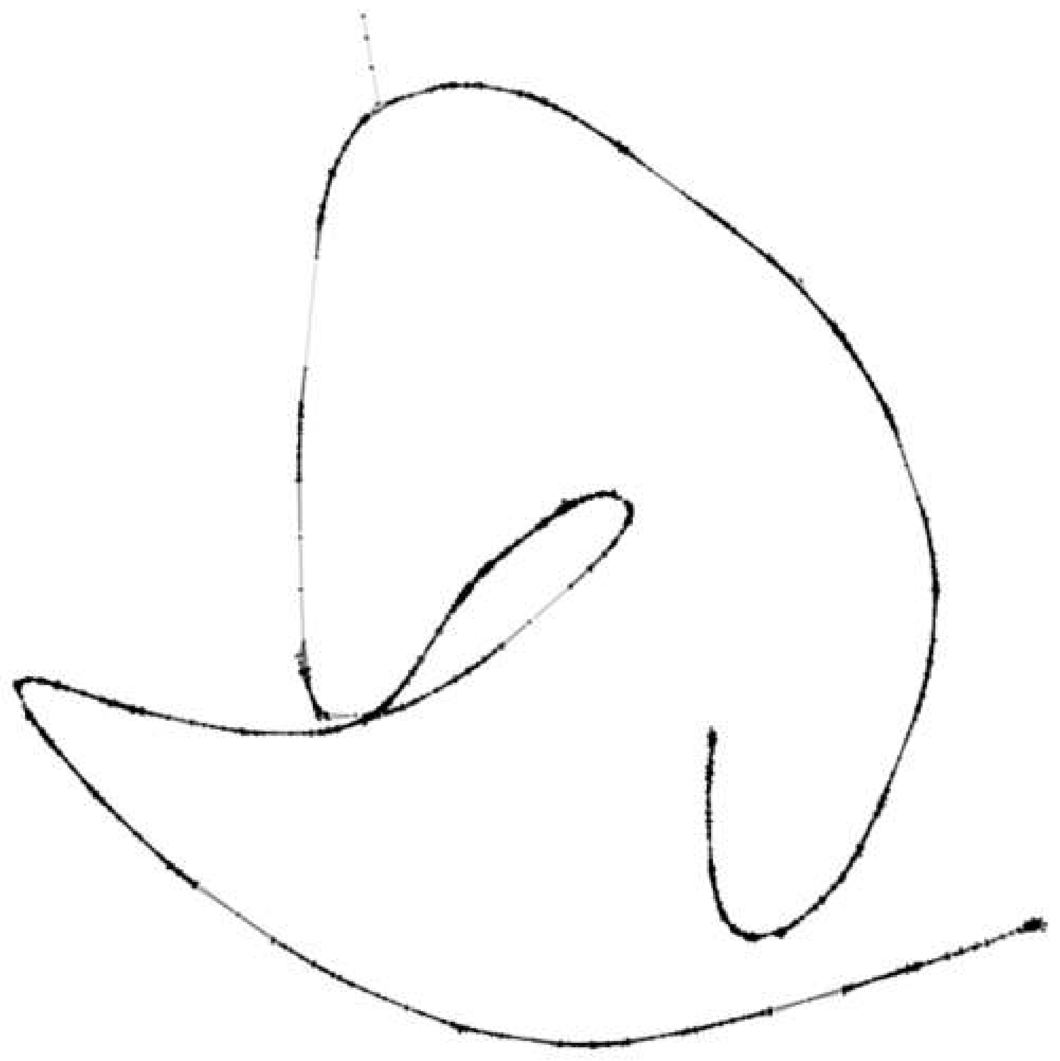

CL42
Low_complexity
Length of Reads (GP):1339 (0.1%)

Tgrandiflorum

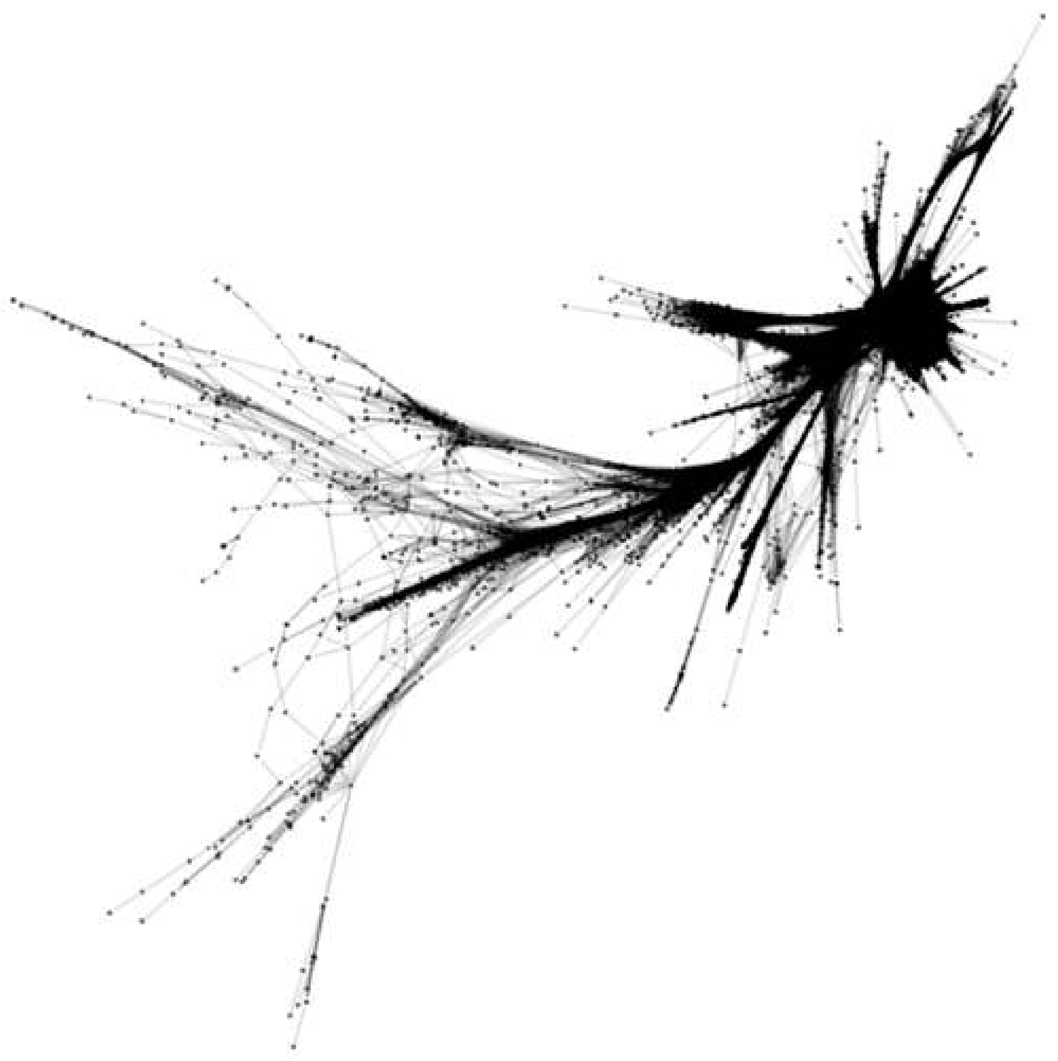

CL42
Low_complexity
Length of Reads (GP):26061 (0.33%)

Tcacao

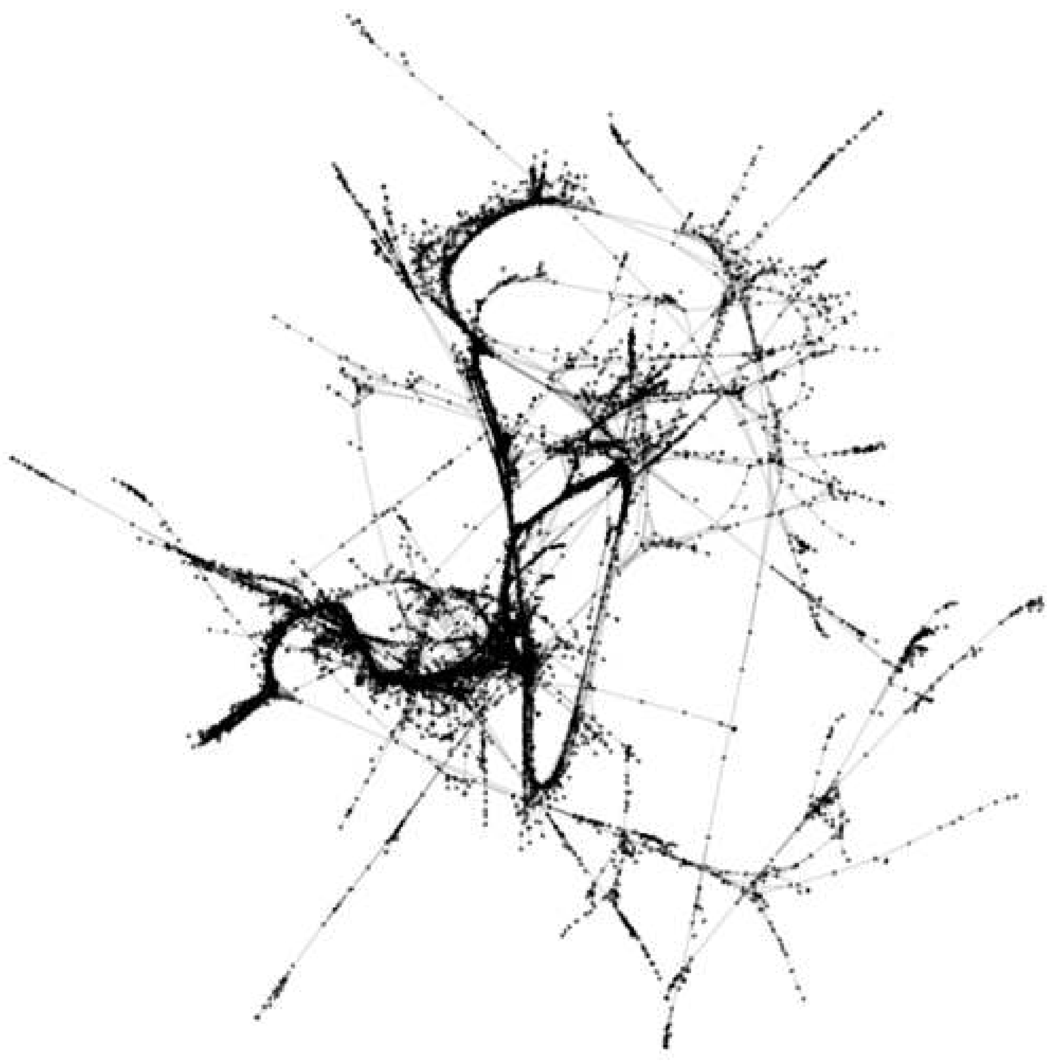

CL42
LTR_Copia
Length of Reads (GP):7719 (0.38%)

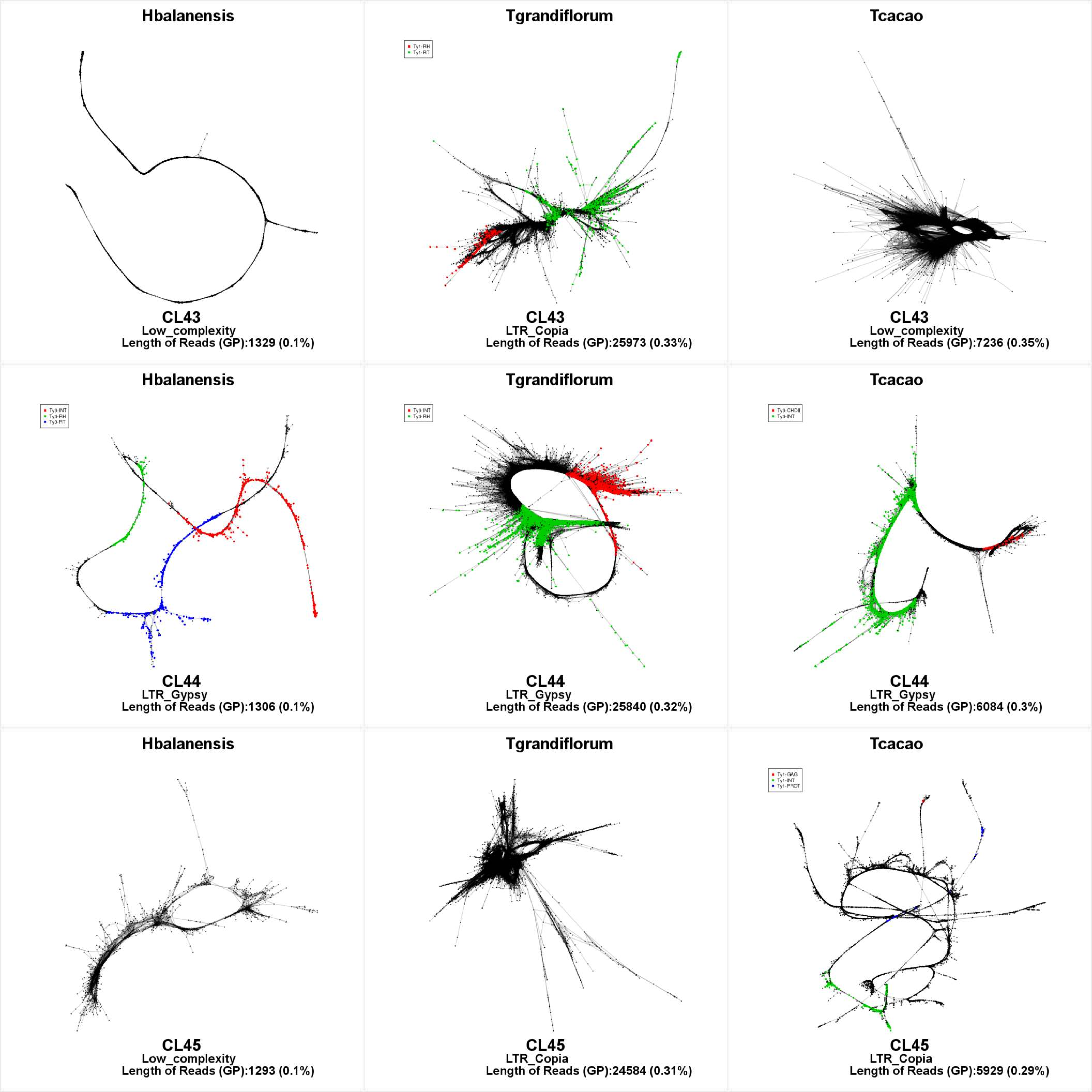
Hbalanensis
CL43
Low_complexity
Length of Reads (GP):1329 (0.1%)
Tgrandiflorum
Ty1-RH
Ty1-RT
CL43
LTR_Copia
Length of Reads (GP):25973 (0.33%)
Tcacao
CL43
Low_complexity
Length of Reads (GP):7236 (0.35%)
Hbalanensis
Ty3-INT
Ty3-RH
Ty3-RT
CL44
LTR_Gypsy
Length of Reads (GP):1306 (0.1%)
Tgrandiflorum
Ty3-INT
Ty3-RH
CL44
LTR_Gypsy
Length of Reads (GP):25840 (0.32%)
Tcacao
Ty3-CHDII
Ty3-INT
CL44
LTR_Gypsy
Length of Reads (GP):6084 (0.3%)
Hbalanensis
CL45
Low_complexity
Length of Reads (GP):1293 (0.1%)
Tgrandiflorum
CL45
LTR_Copia
Length of Reads (GP):24584 (0.31%)
Tcacao
Ty1-GAG
Ty1-INT
Ty1-PROT
CL45
LTR_Copia
Length of Reads (GP):5929 (0.29%)

Hbalanensis

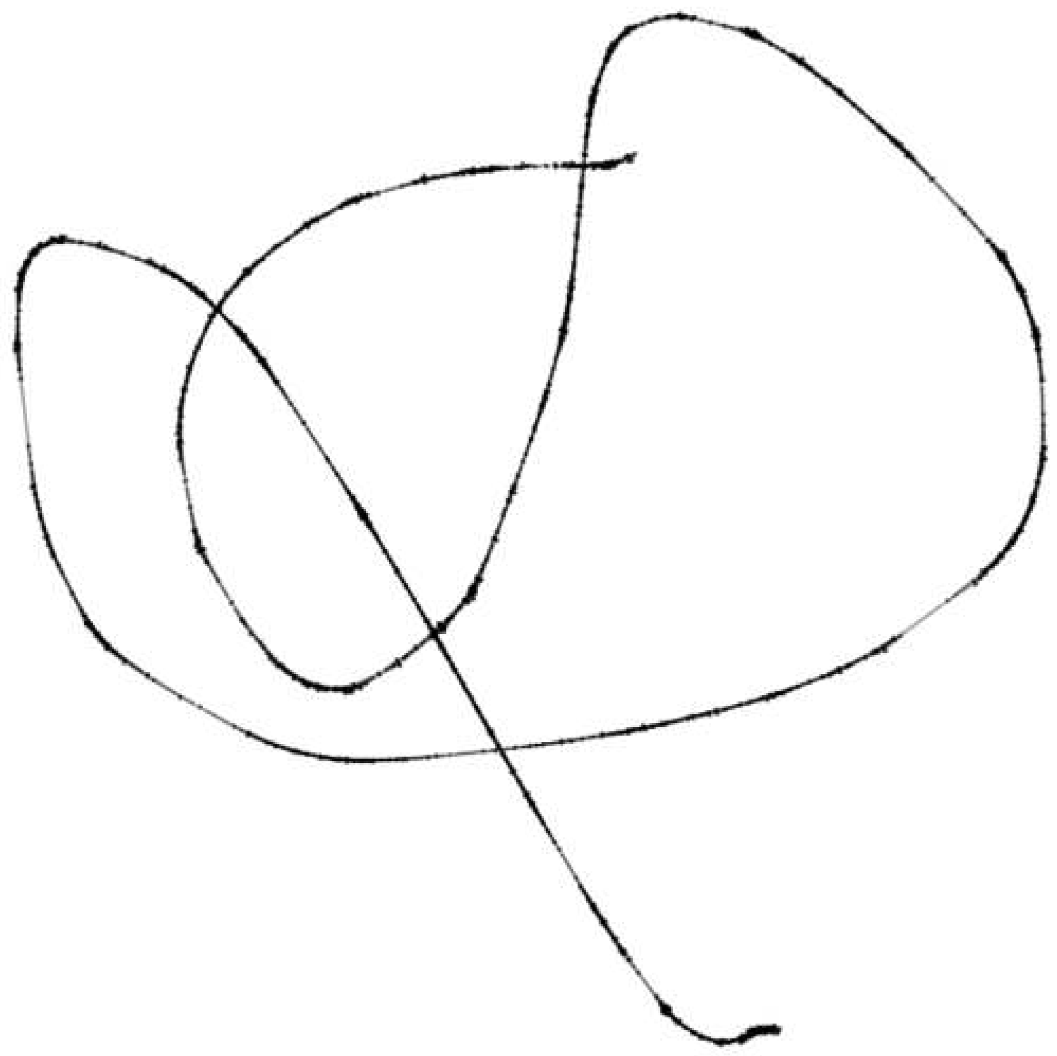

CL46
DNA_MULE_MuDR
Length of Reads (GP):1249 (0.09%)

Tgrandiflorum

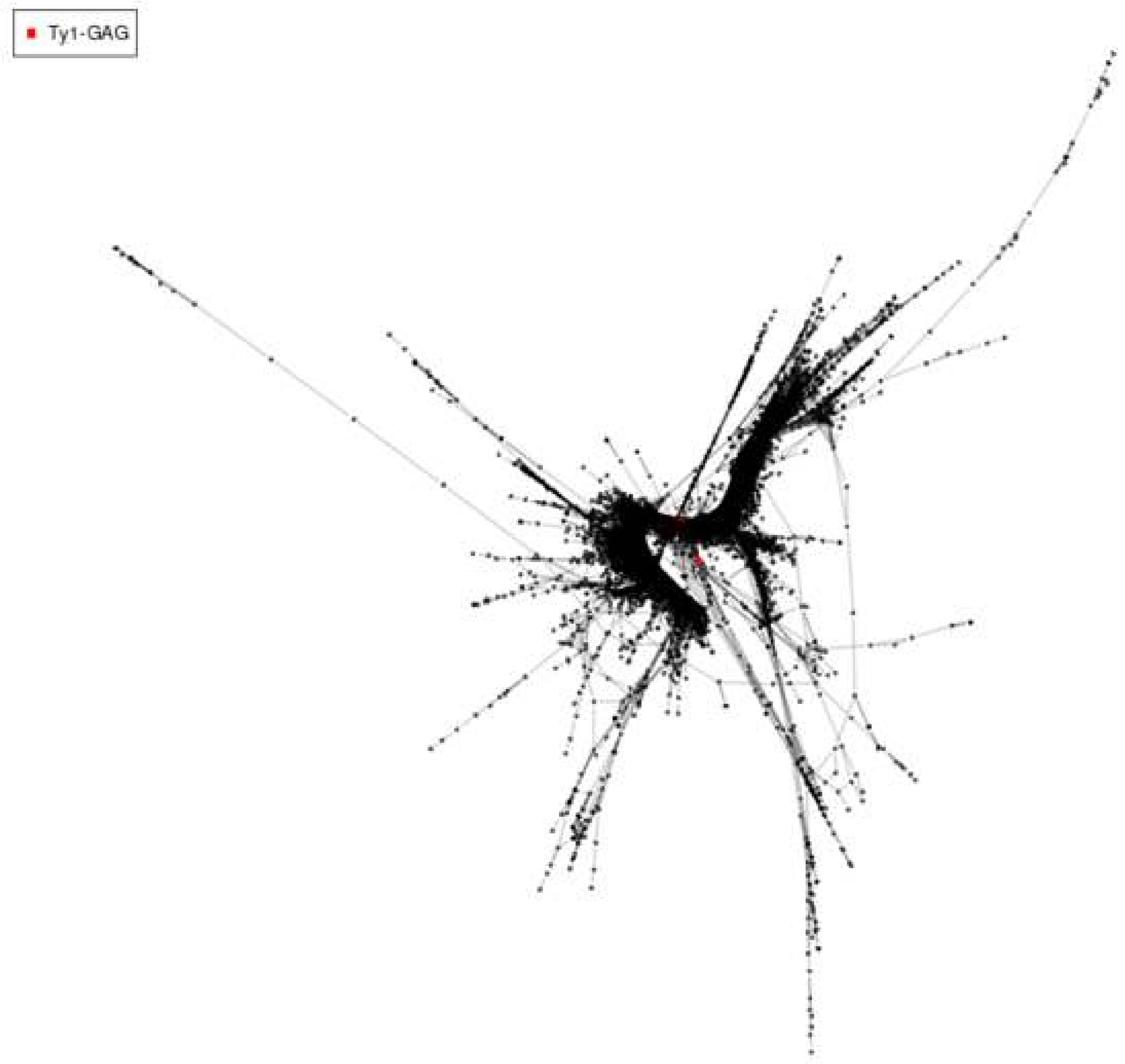

CL46
LTR_Copia
Length of Reads (GP):24505 (0.31%)

Tcacao

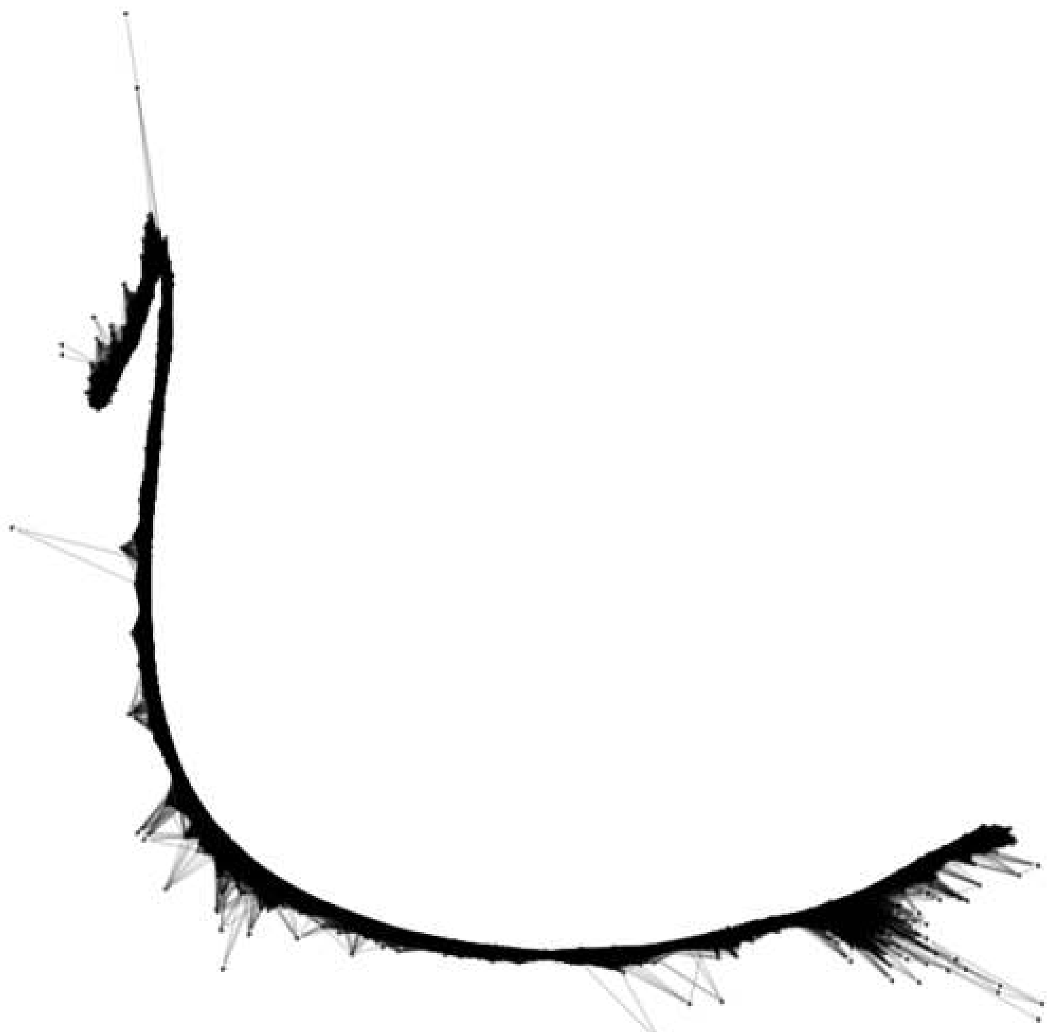

CL46
Low_complexity
Length of Reads (GP):5904 (0.29%)

Hbalanensis

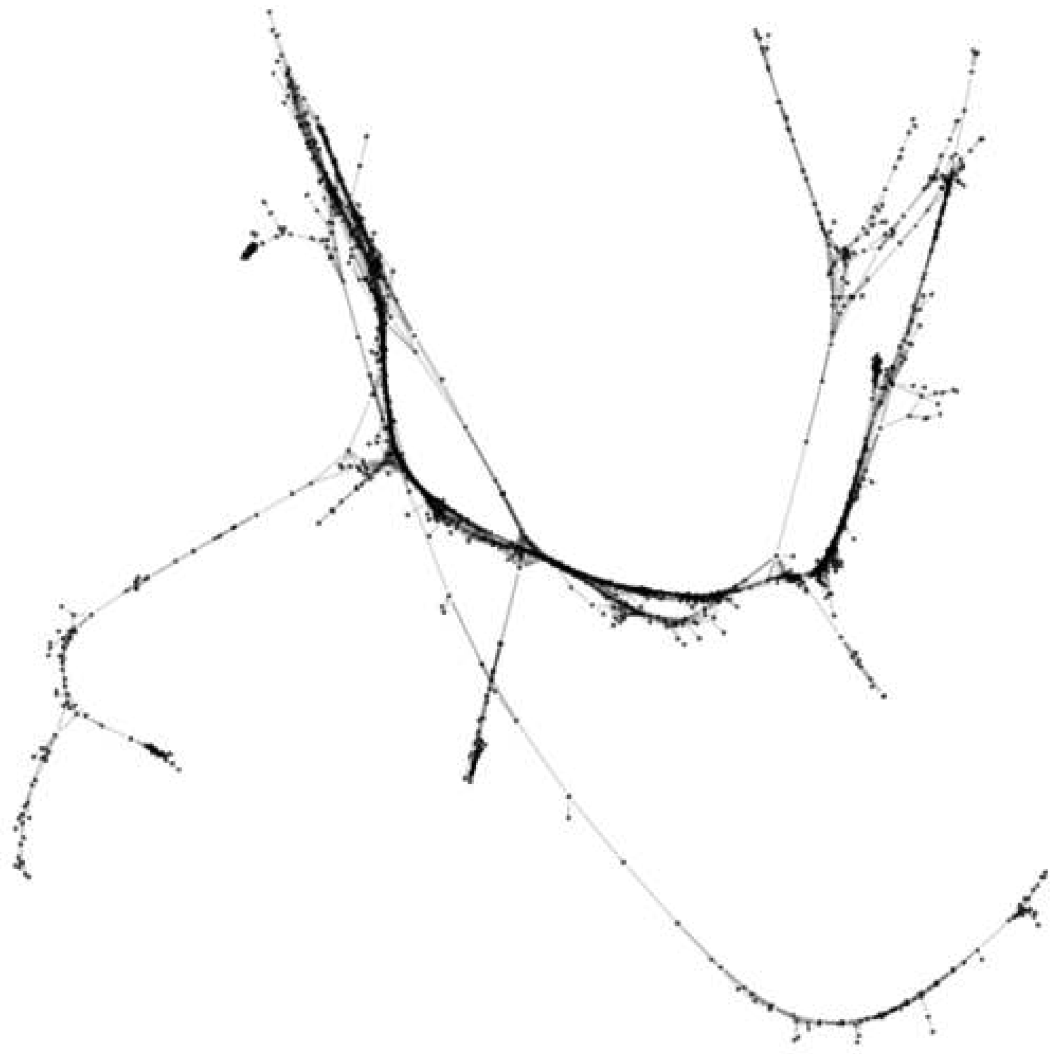

CL47
Low_complexity
Length of Reads (GP):1232 (0.09%)

Tgrandiflorum

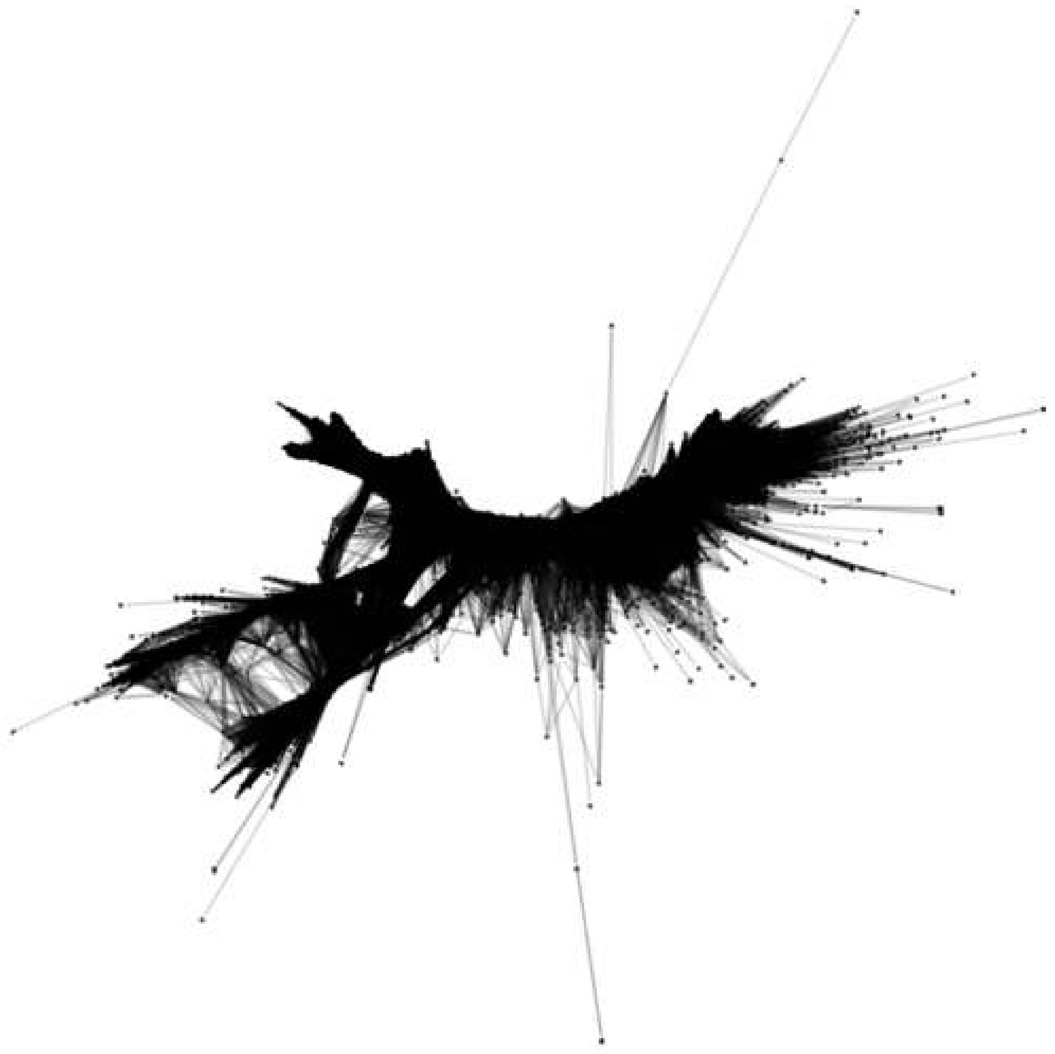

CL47
LTR_Gypsy
Length of Reads (GP):23827 (0.3%)

Tcacao

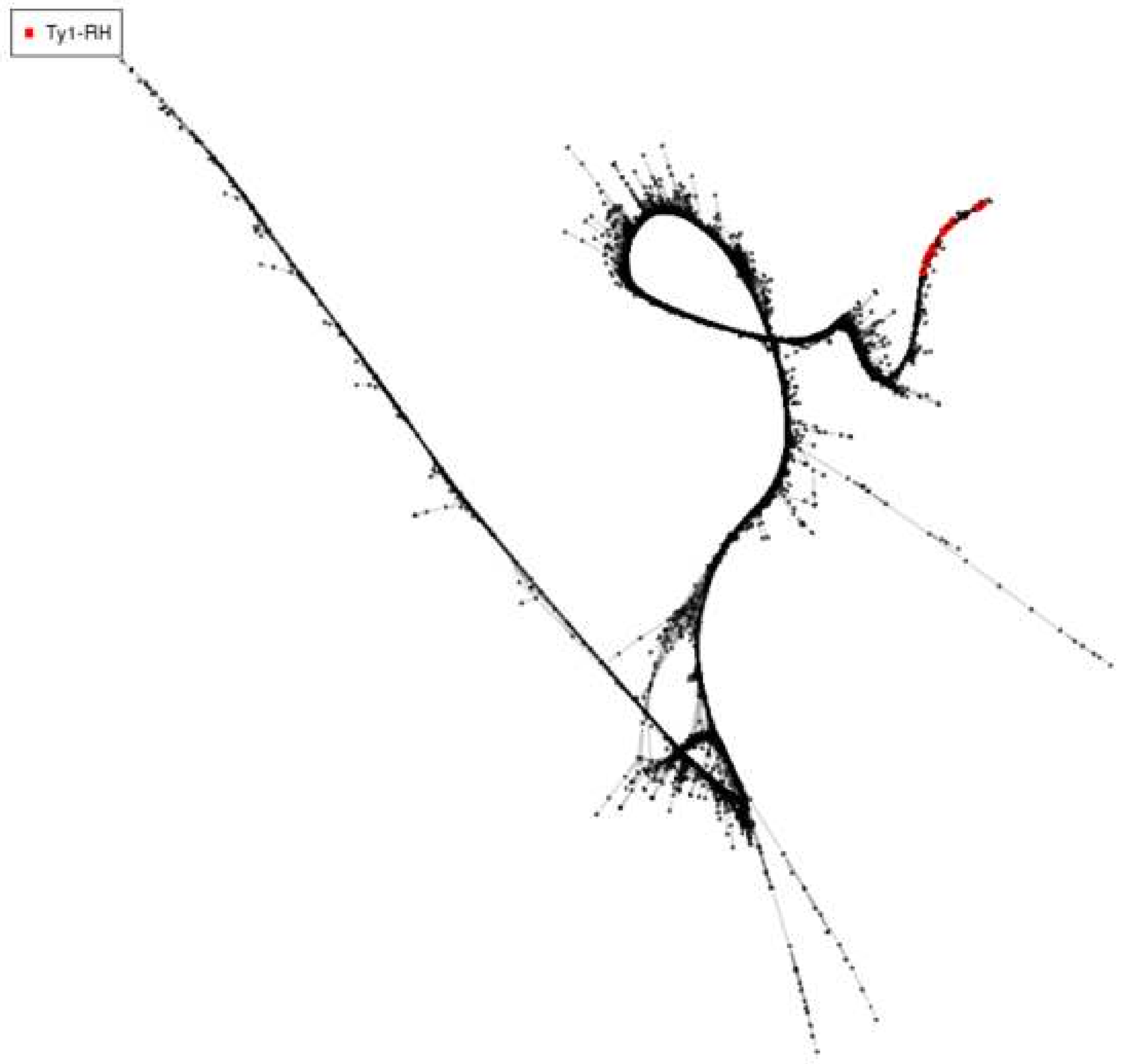

CL47
Low_complexity
Length of Reads (GP):5896 (0.29%)

Hbalanensis

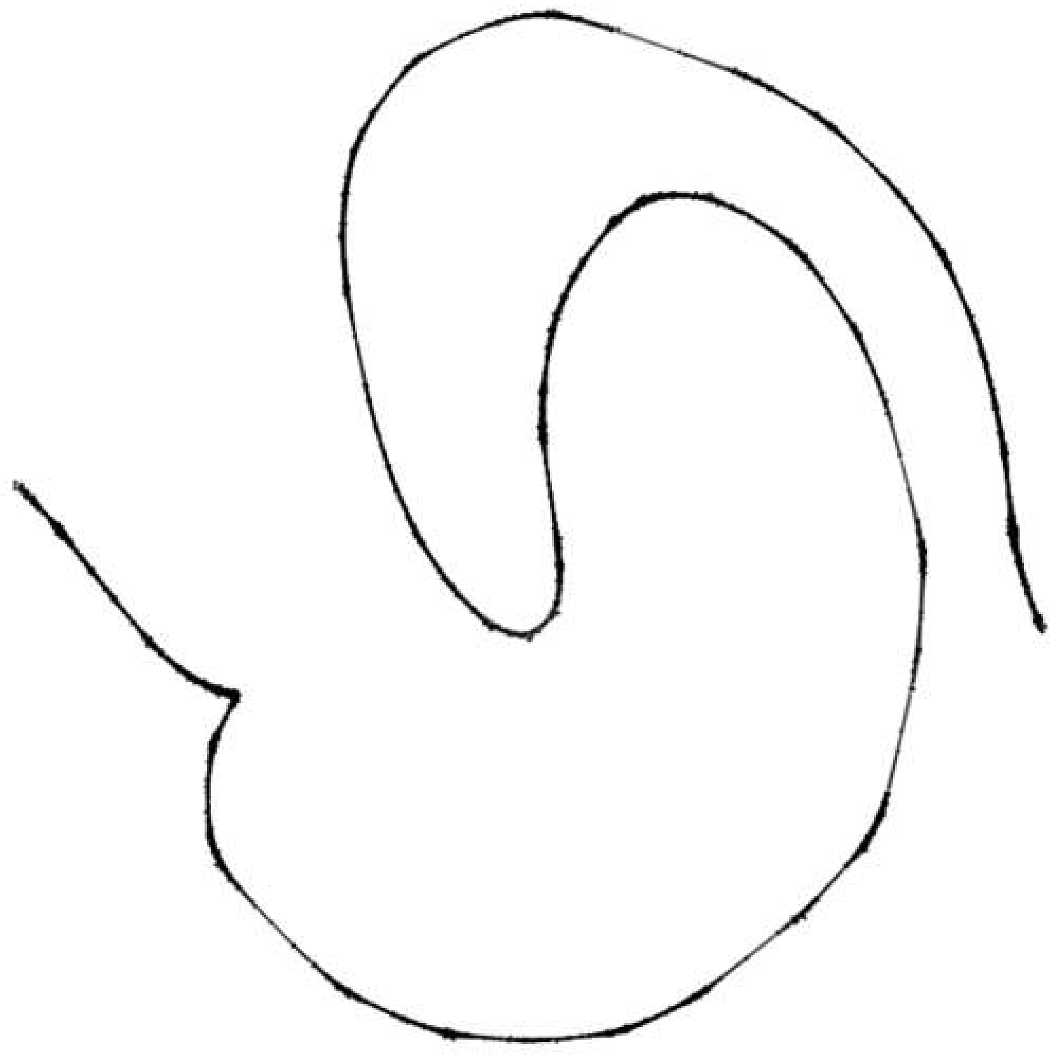

CL48
DNA_hAT_Ac
Length of Reads (GP):1197 (0.09%)

Tgrandiflorum

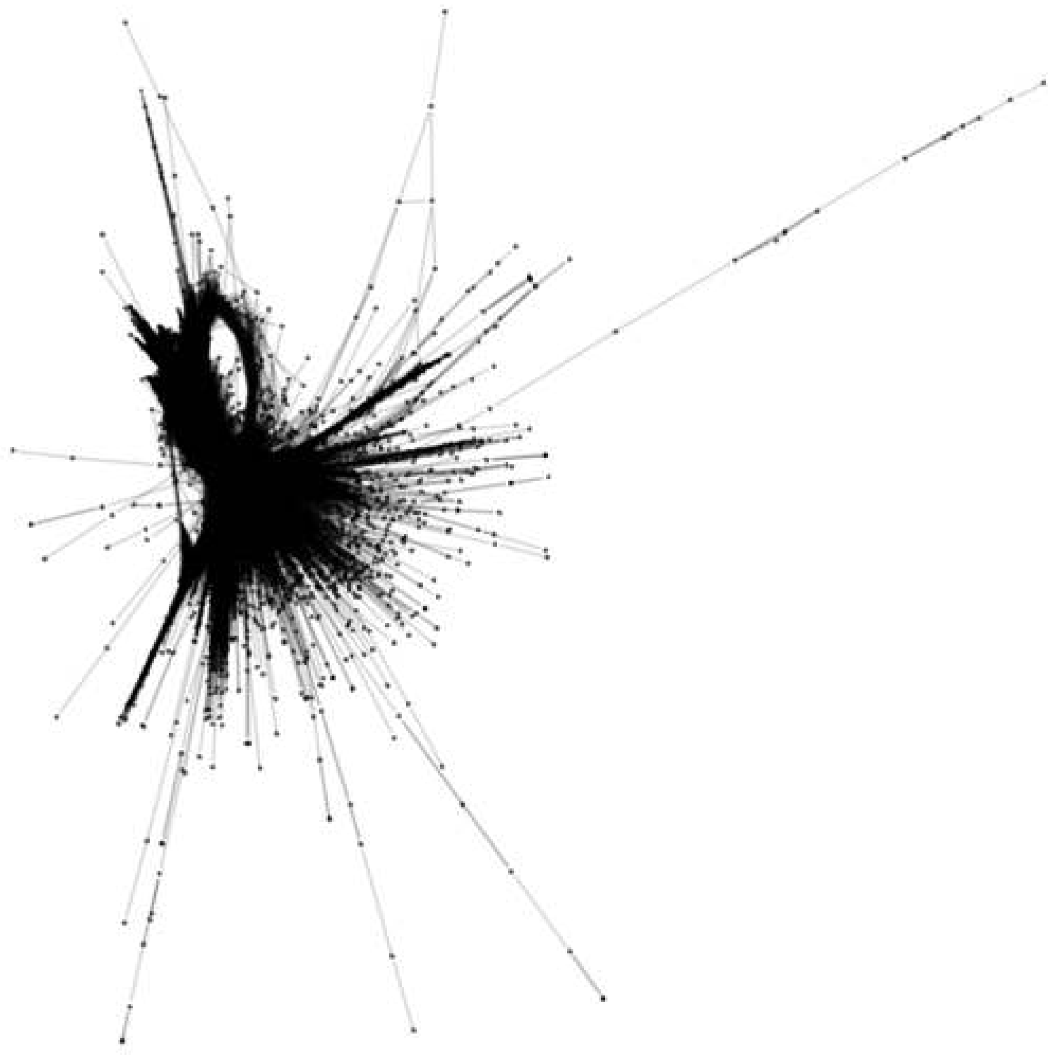

CL48
LTR_Gypsy
Length of Reads (GP):23704 (0.3%)

Tcacao

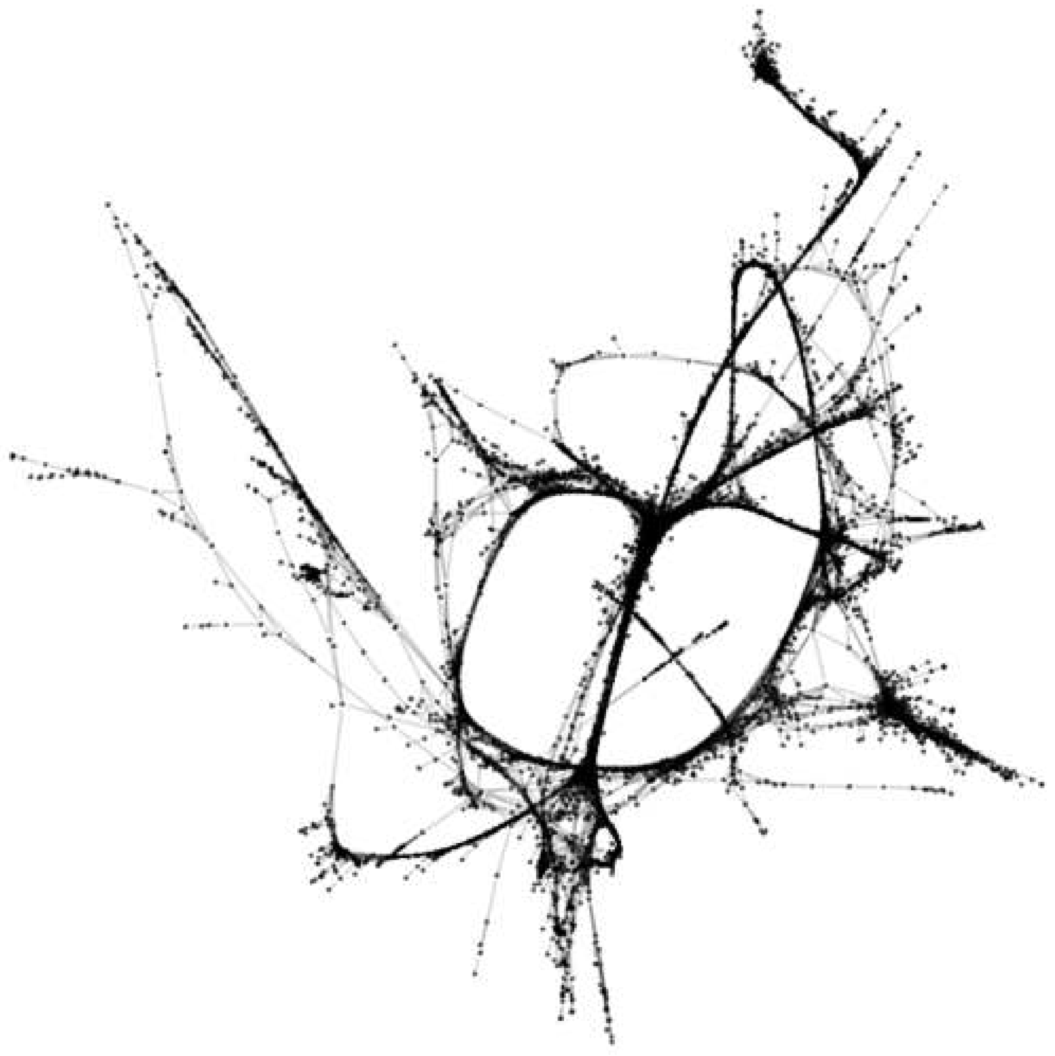

CL48
Low_complexity
Length of Reads (GP):5817 (0.28%)

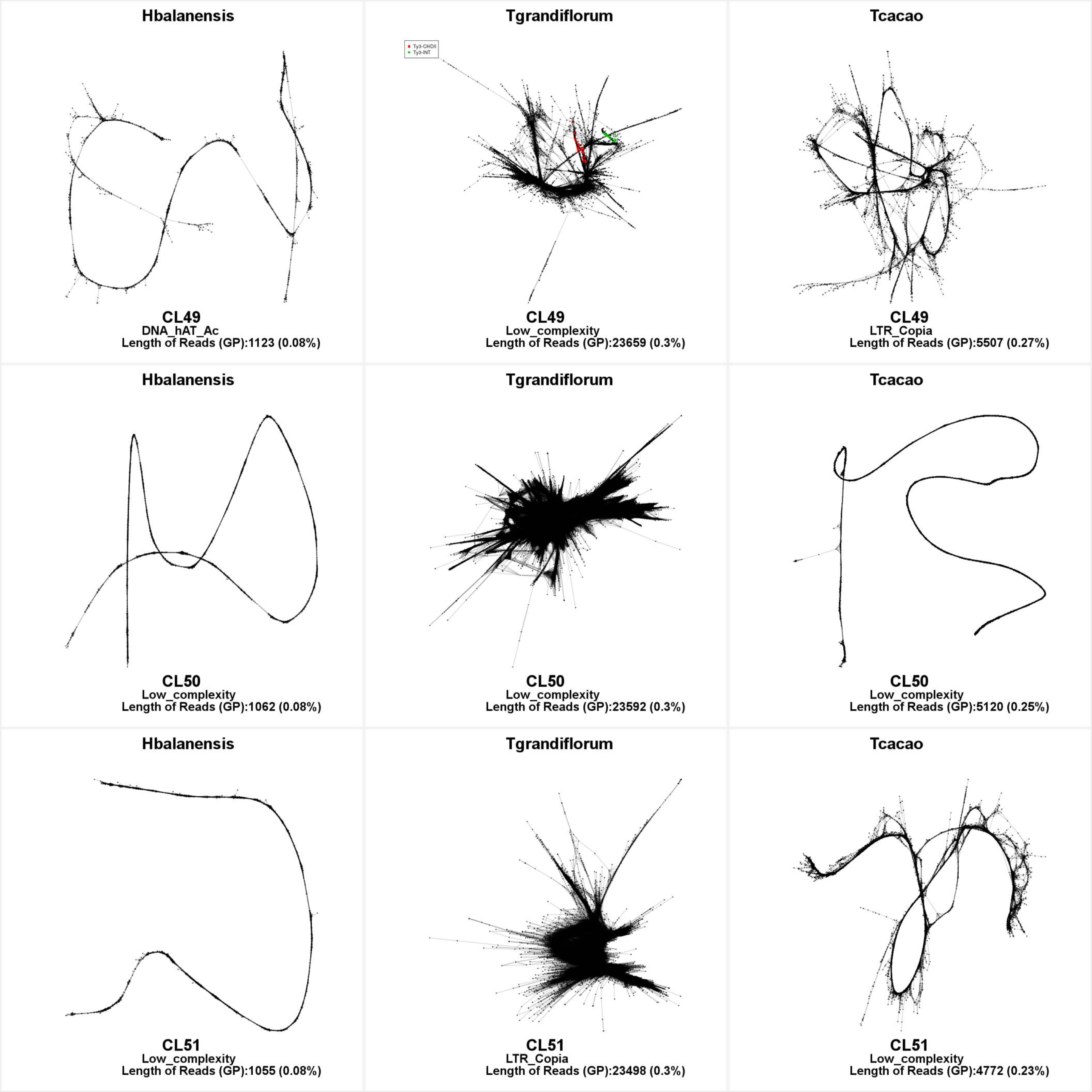
Hbalanensis
CL49
DNA_hAT_Ac
Length of Reads (GP):1123 (0.08%)
Tgrandiflorum
Ty3-CHDII
Ty3-INT
CL49
Low_complexity
Length of Reads (GP):23659 (0.3%)
Tcacao
CL49
LTR_Copia
Length of Reads (GP):5507 (0.27%)
Hbalanensis
CL50
Low_complexity
Length of Reads (GP):1062 (0.08%)
Tgrandiflorum
CL50
Low_complexity
Length of Reads (GP):23592 (0.3%)
Tcacao
CL50
Low_complexity
Length of Reads (GP):5120 (0.25%)
Hbalanensis
CL51
Low_complexity
Length of Reads (GP):1055 (0.08%)
Tgrandiflorum
CL51
LTR_Copia
Length of Reads (GP):23498 (0.3%)
Tcacao
CL51
Low_complexity
Length of Reads (GP):4772 (0.23%)

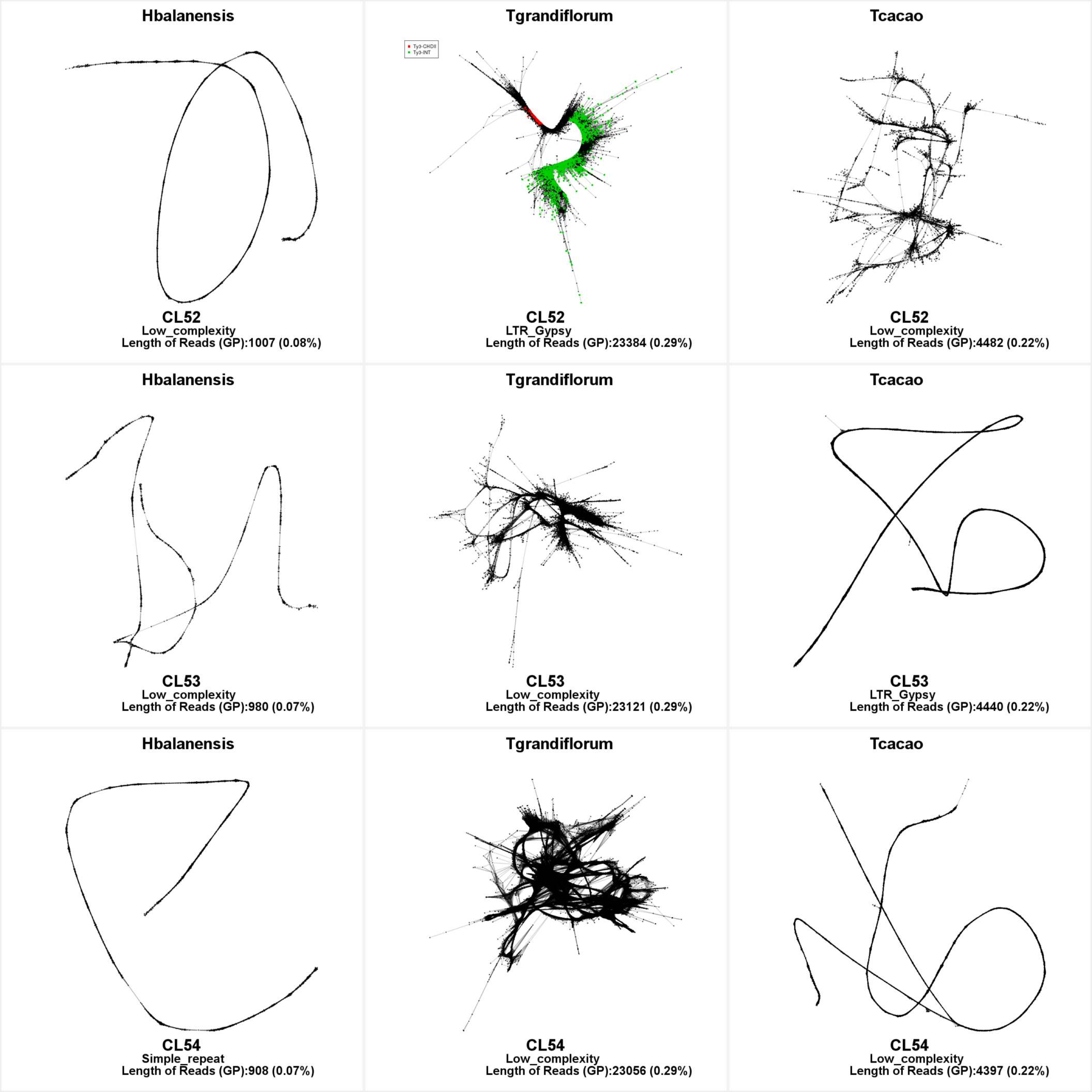
Hbalanensis
CL52
Low_complexity
Length of Reads (GP):1007 (0.08%)
Tgrandiflorum
Ty3-CHDII
Ty3-INT
CL52
LTR_Gypsy
Length of Reads (GP):23384 (0.29%)
Tcacao
CL52
Low_complexity
Length of Reads (GP):4482 (0.22%)
Hbalanensis
CL53
Low_complexity
Length of Reads (GP):980 (0.07%)
Tgrandiflorum
CL53
Low_complexity
Length of Reads (GP):23121 (0.29%)
Tcacao
CL53
LTR_Gypsy
Length of Reads (GP):4440 (0.22%)
Hbalanensis
CL54
Simple_repeat
Length of Reads (GP):908 (0.07%)
Tgrandiflorum
CL54
Low_complexity
Length of Reads (GP):23056 (0.29%)
Tcacao
CL54
Low_complexity
Length of Reads (GP):4397 (0.22%)

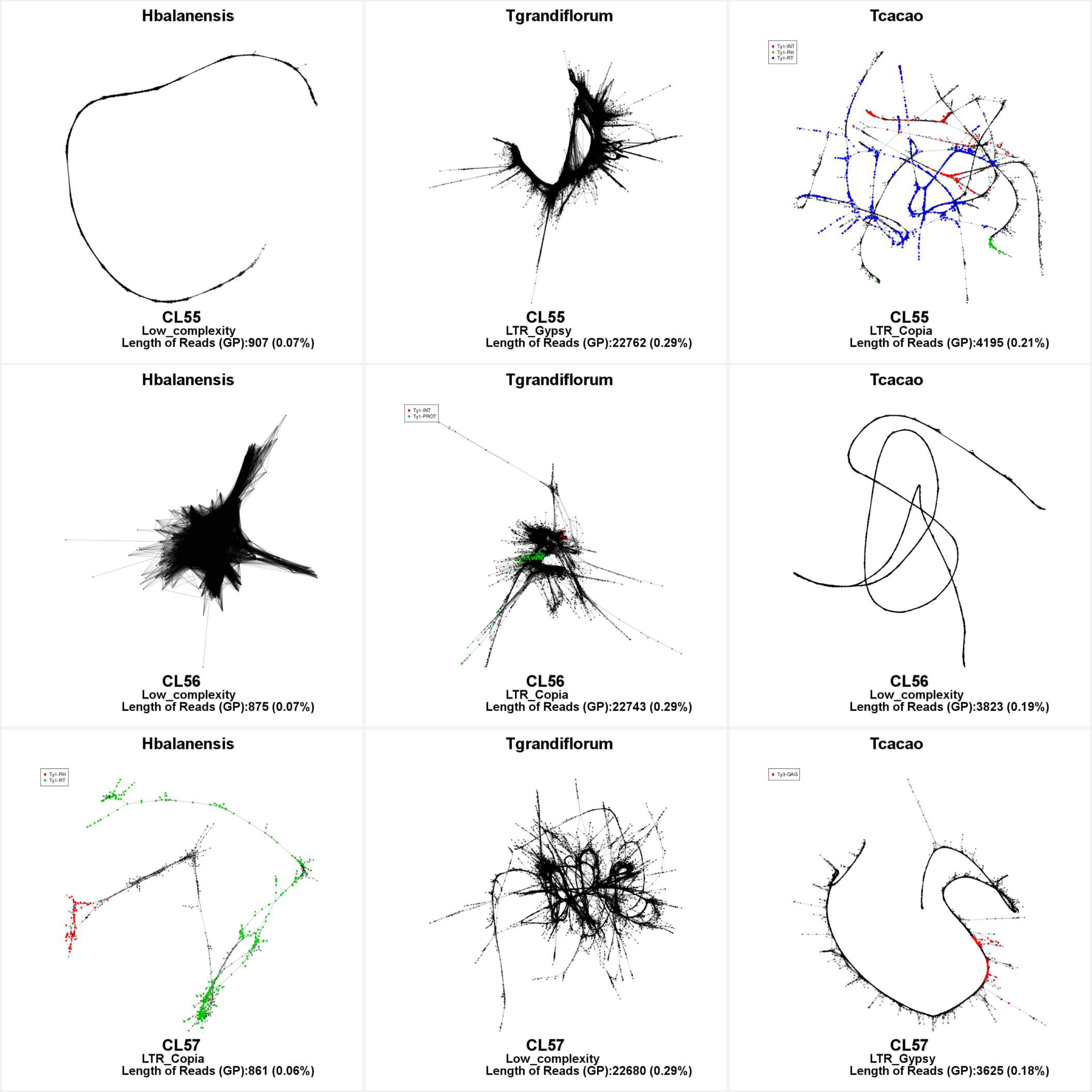

Hbalanensis
CL55
Low_complexity
Length of Reads (GP):907 (0.07%)
Tgrandiflorum
CL55
LTR_Gypsy
Length of Reads (GP):22762 (0.29%)
Tcacao
Ty1-INT
Ty1-RH
Ty1-RT
CL55
LTR_Copia
Length of Reads (GP):4195 (0.21%)
Hbalanensis
CL56
Low_complexity
Length of Reads (GP):875 (0.07%)
Tgrandiflorum
Ty1-INT
Ty1-PROT
CL56
LTR_Copia
Length of Reads (GP):22743 (0.29%)
Tcacao
CL56
Low_complexity
Length of Reads (GP):3823 (0.19%)
Hbalanensis
Ty1-RH
Ty1-RT
CL57
LTR_Copia
Length of Reads (GP):861 (0.06%)
Tgrandiflorum
CL57
Low_complexity
Length of Reads (GP):22680 (0.29%)
Tcacao
Ty3-GAG
CL57
LTR_Gypsy
Length of Reads (GP):3625 (0.18%)

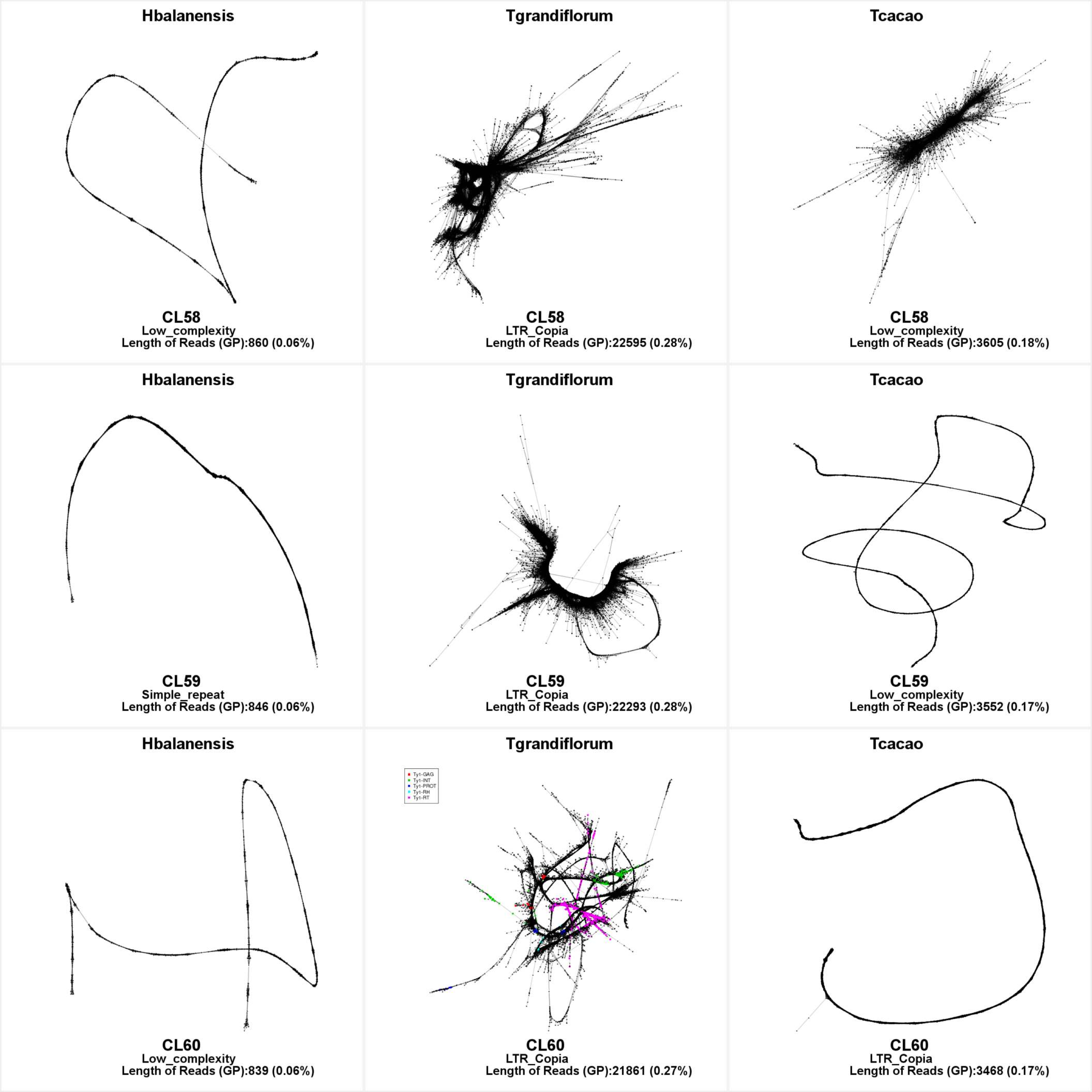
Hbalanensis
CL58
Low_complexity
Length of Reads (GP):860 (0.06%)
Tgrandiflorum
CL58
LTR_Copia
Length of Reads (GP):22595 (0.28%)
Tcacao
CL58
Low_complexity
Length of Reads (GP):3605 (0.18%)
Hbalanensis
CL59
Simple_repeat
Length of Reads (GP):846 (0.06%)
Tgrandiflorum
CL59
LTR_Copia
Length of Reads (GP):22293 (0.28%)
Tcacao
CL59
Low_complexity
Length of Reads (GP):3552 (0.17%)
Hbalanensis
CL60
Low_complexity
Length of Reads (GP):839 (0.06%)
Tgrandiflorum
Ty1-GAG
Ty1-INT
Ty1-PROT
Ty1-RH
Ty1-RT
CL60
LTR_Copia
Length of Reads (GP):21861 (0.27%)
Tcacao
CL60
LTR_Copia
Length of Reads (GP):3468 (0.17%)

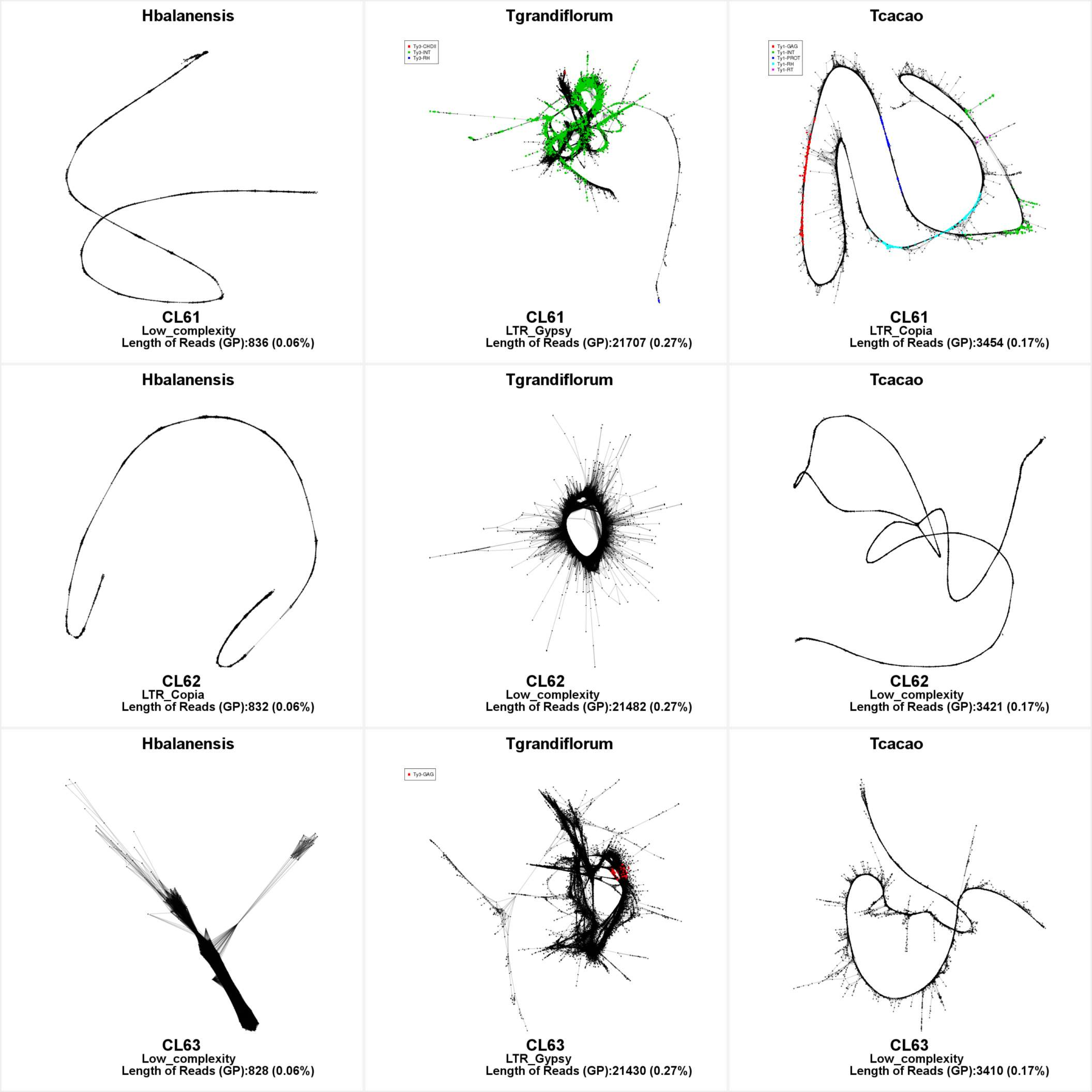
Hbalanensis
CL61
Low_complexity
Length of Reads (GP):836 (0.06%)
Tgrandiflorum
Ty3-CHDII
Ty3-INT
Ty3-RH
CL61
LTR_Gypsy
Length of Reads (GP):21707 (0.27%)
Tcacao
Ty1-GAG
Ty1-INT
Ty1-PROT
Ty1-RH
Ty1-RT
CL61
LTR_Copia
Length of Reads (GP):3454 (0.17%)
Hbalanensis
CL62
LTR_Copia
Length of Reads (GP):832 (0.06%)
Tgrandiflorum
CL62
Low_complexity
Length of Reads (GP):21482 (0.27%)
Tcacao
CL62
Low_complexity
Length of Reads (GP):3421 (0.17%)
Hbalanensis
CL63
Low_complexity
Length of Reads (GP):828 (0.06%)
Tgrandiflorum
Ty3-GAG
CL63
LTR_Gypsy
Length of Reads (GP):21430 (0.27%)
Tcacao
CL63
Low_complexity
Length of Reads (GP):3410 (0.17%)

Hbalanensis

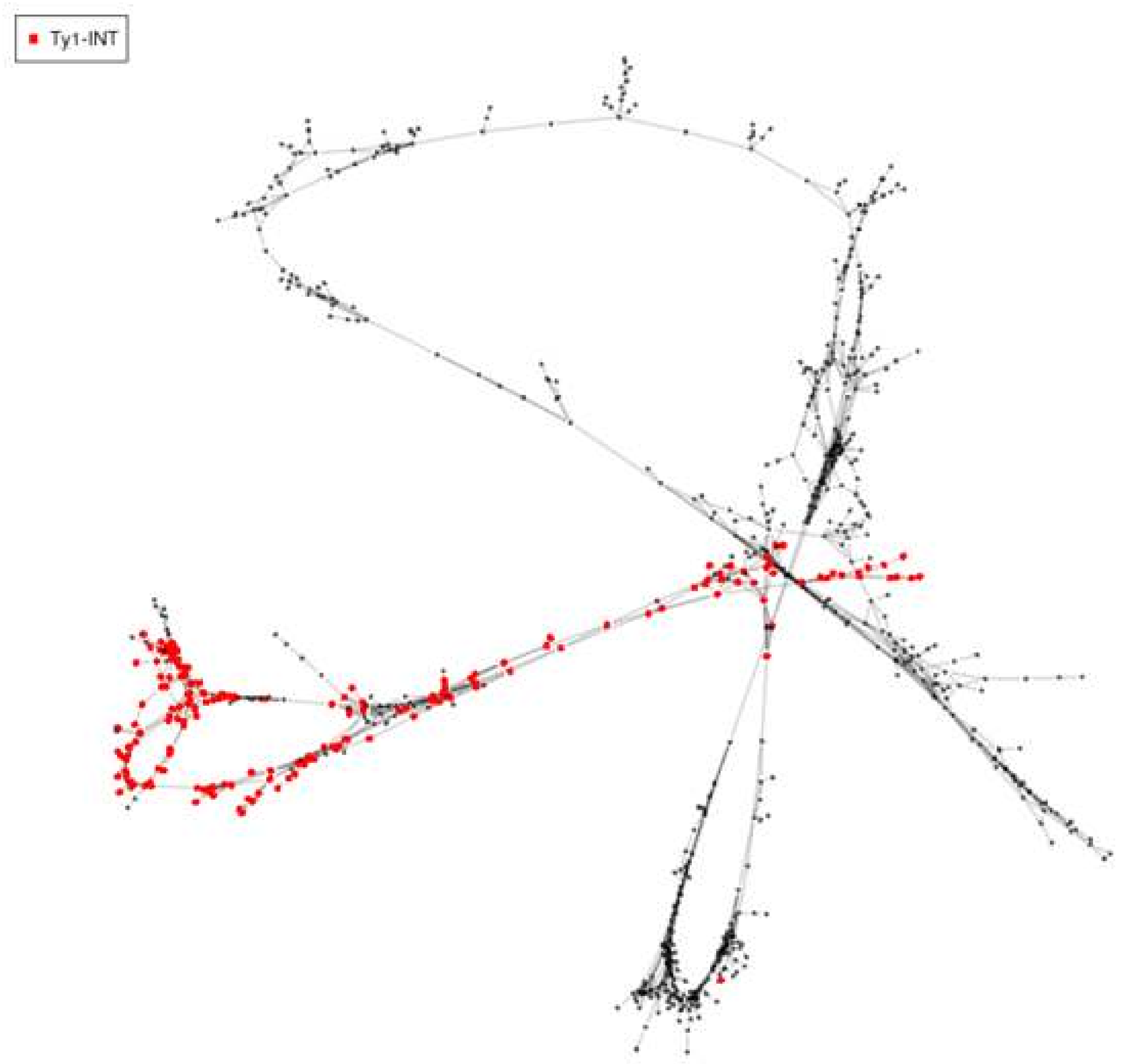

CL64
LTR_Copia
Length of Reads (GP):812 (0.06%)

Tgrandiflorum

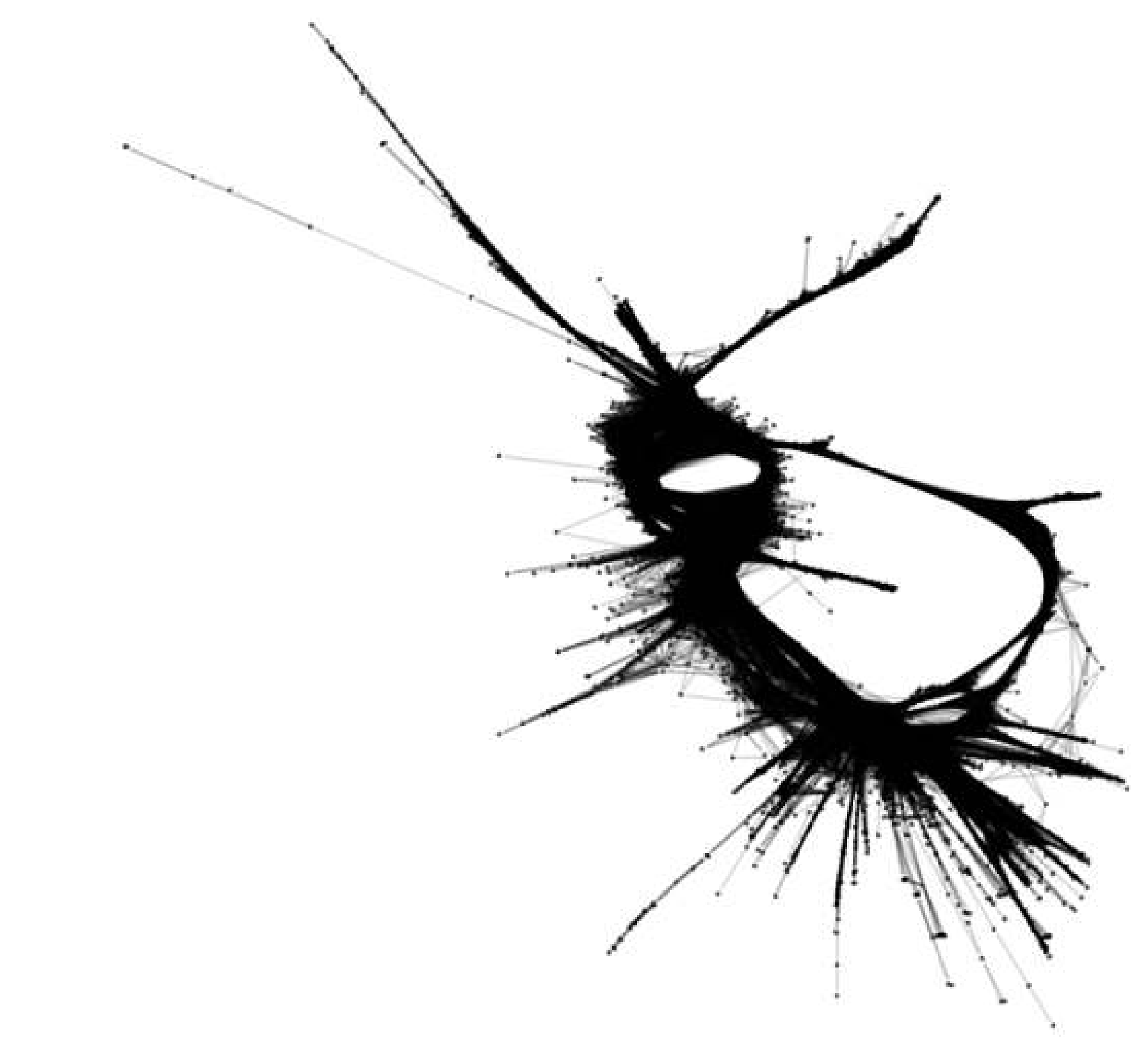

CL64
Low_complexity
Length of Reads (GP):21332 (0.27%)

Tcacao

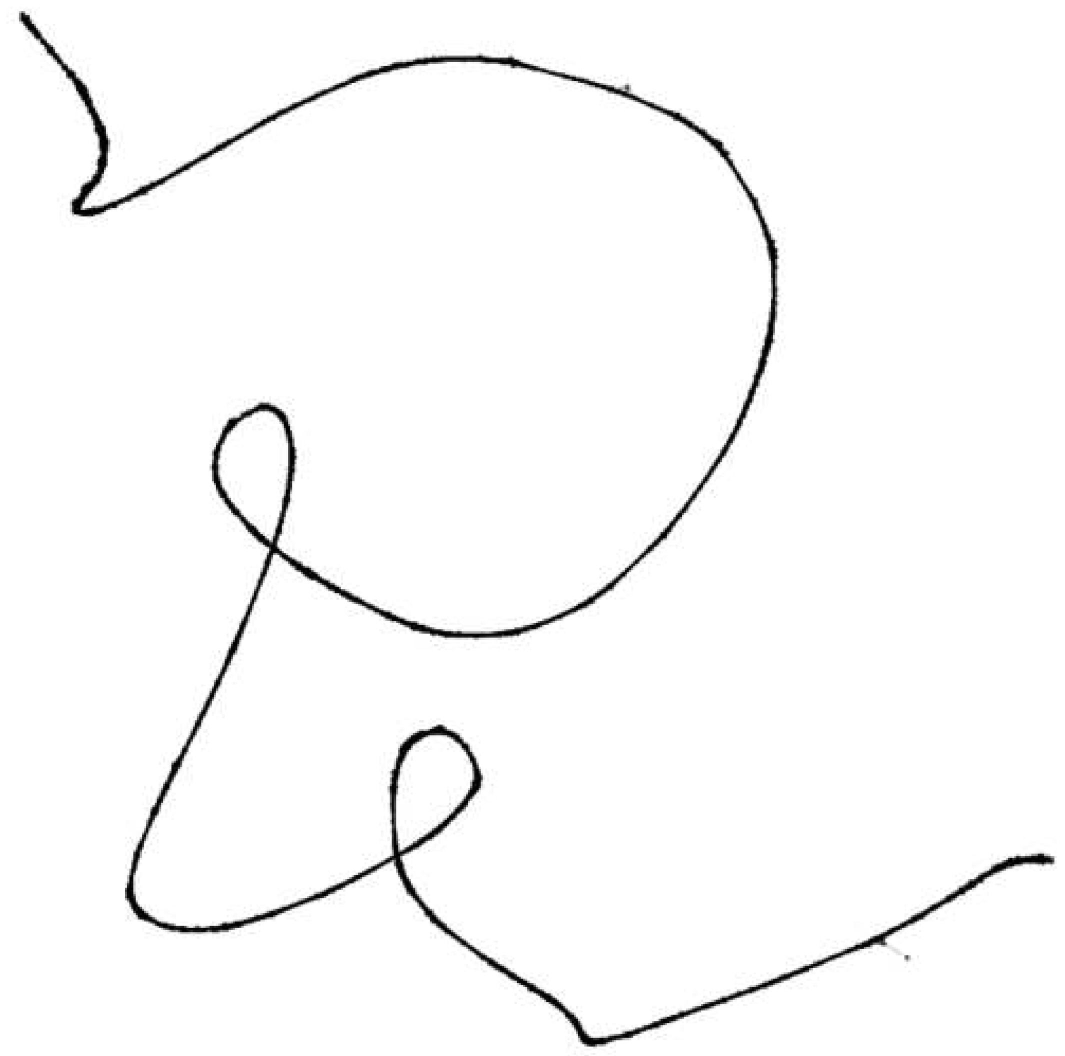

CL64
DNA_MULE_MuDR
Length of Reads (GP):3308 (0.16%)

Hbalanensis

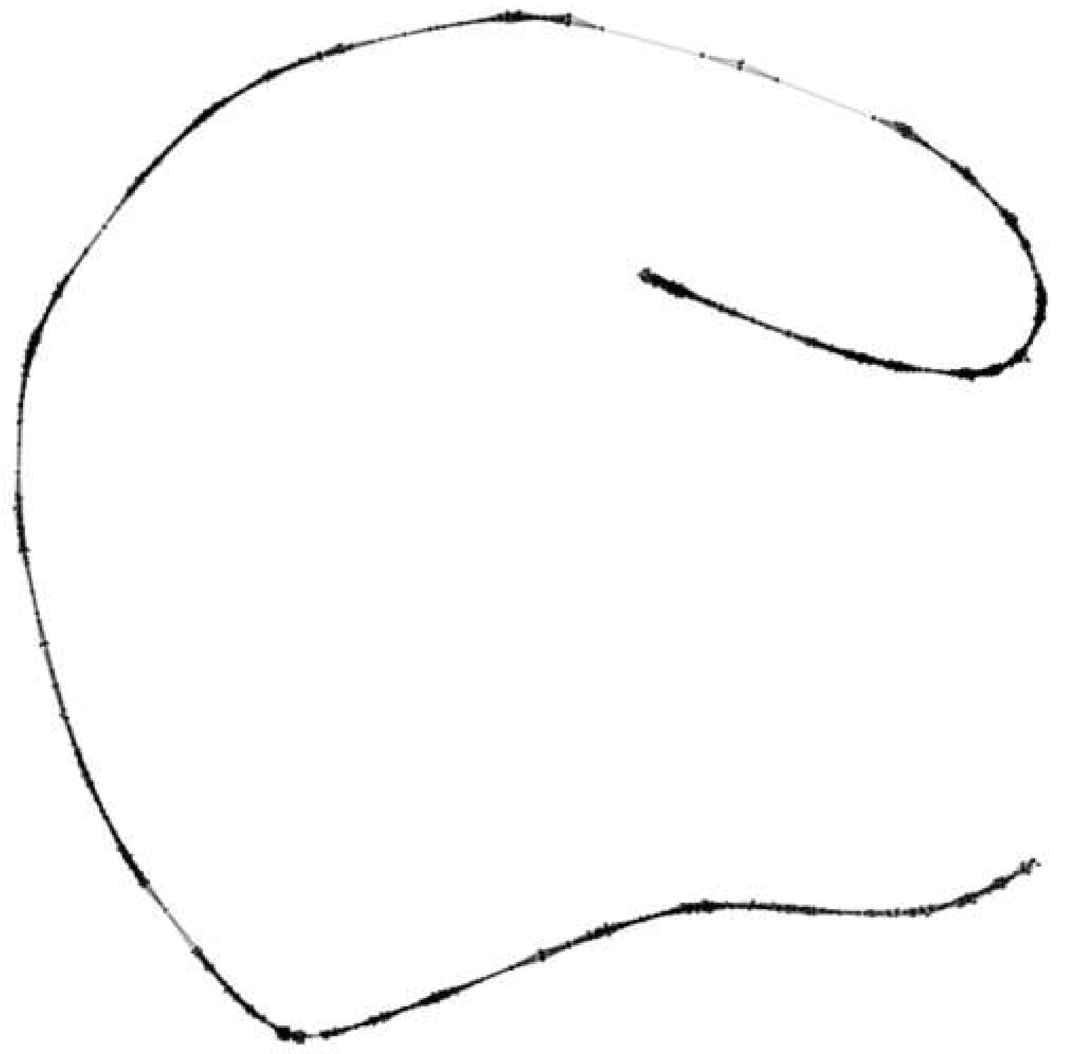

CL65
Low_complexity
Length of Reads (GP):807 (0.06%)

Tgrandiflorum

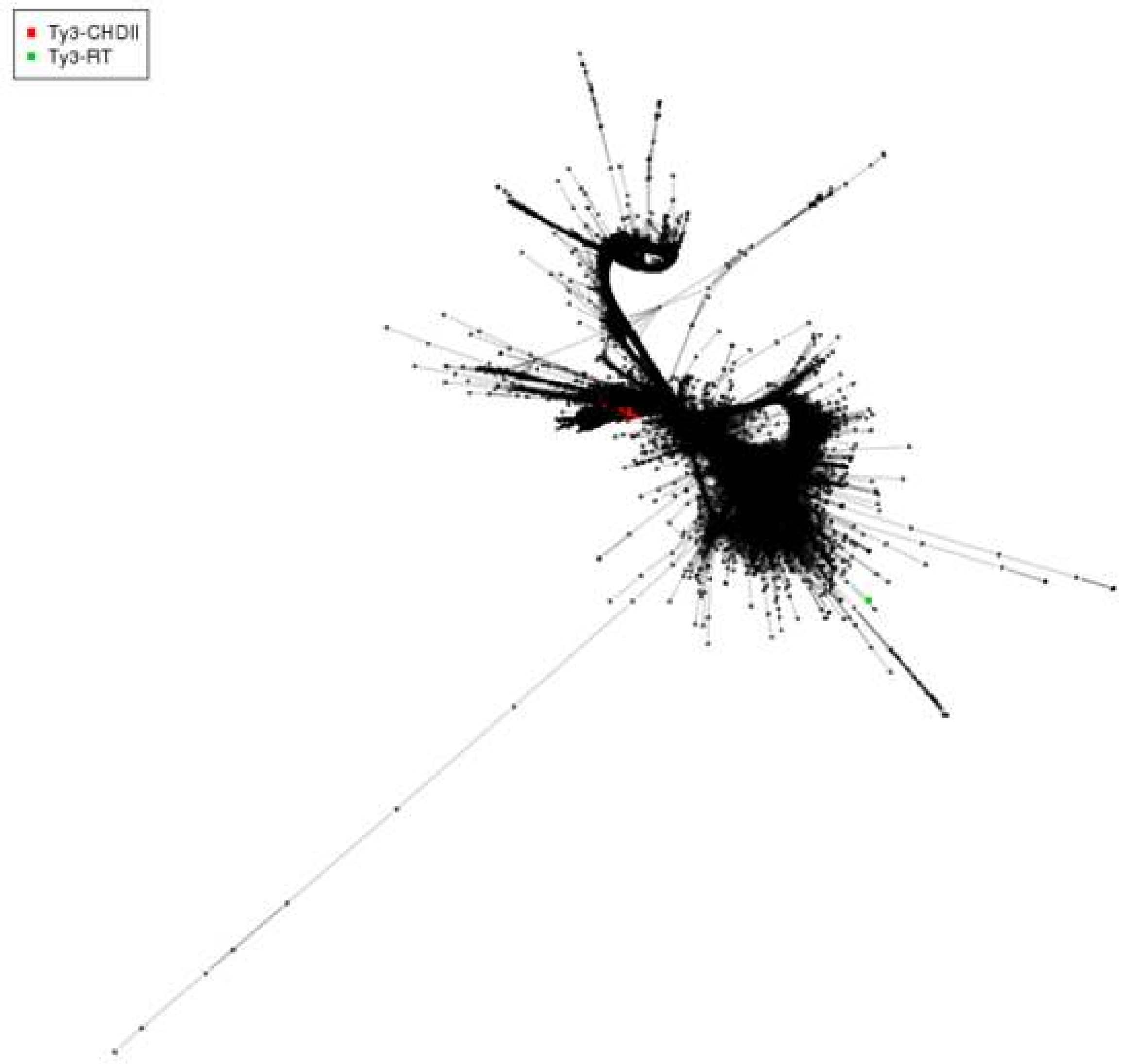

CL65
LTR_Gypsy
Length of Reads (GP):21331 (0.27%)

Tcacao

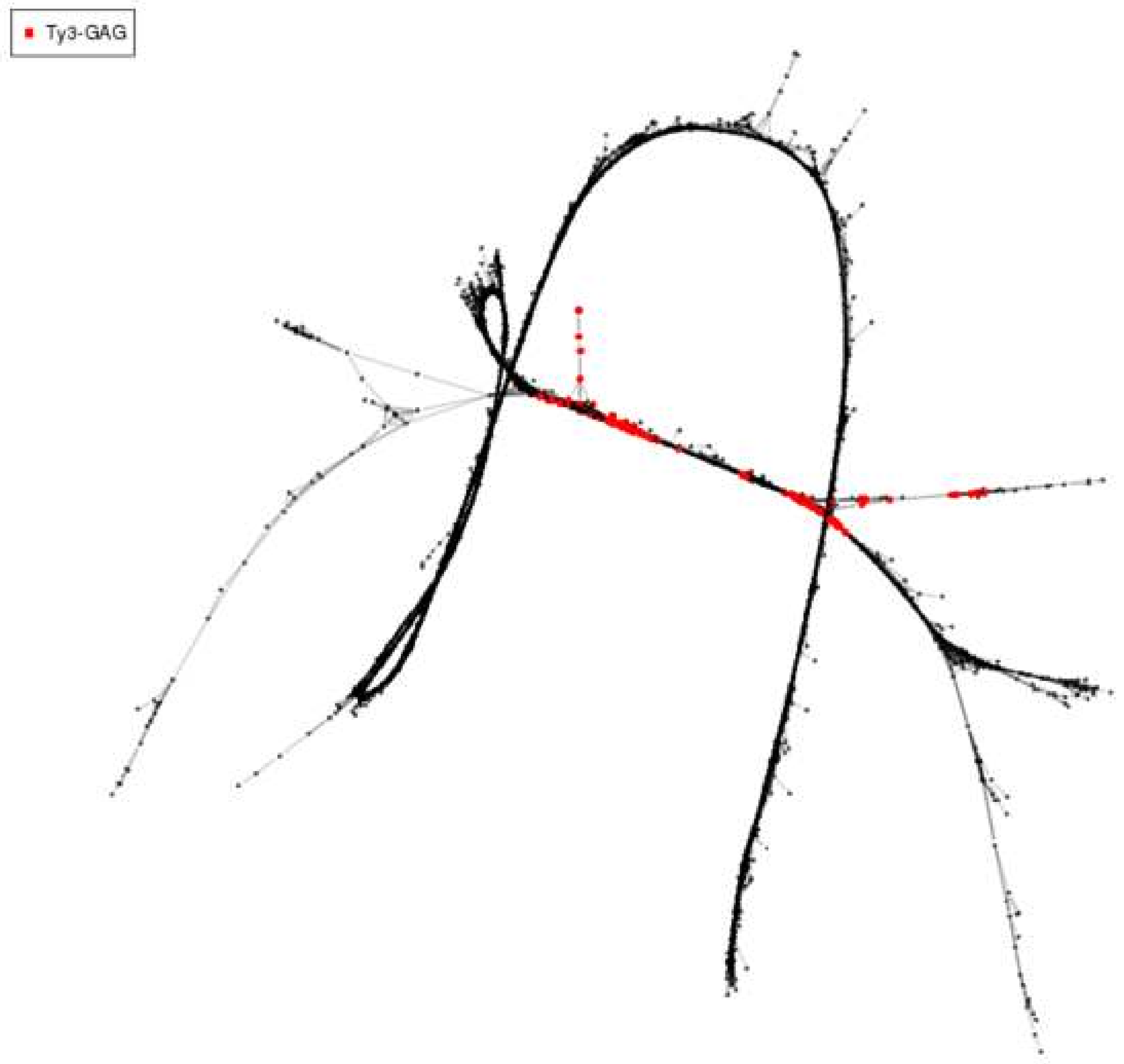

CL65
LTR_Gypsy
Length of Reads (GP):2913 (0.14%)

Hbalanensis

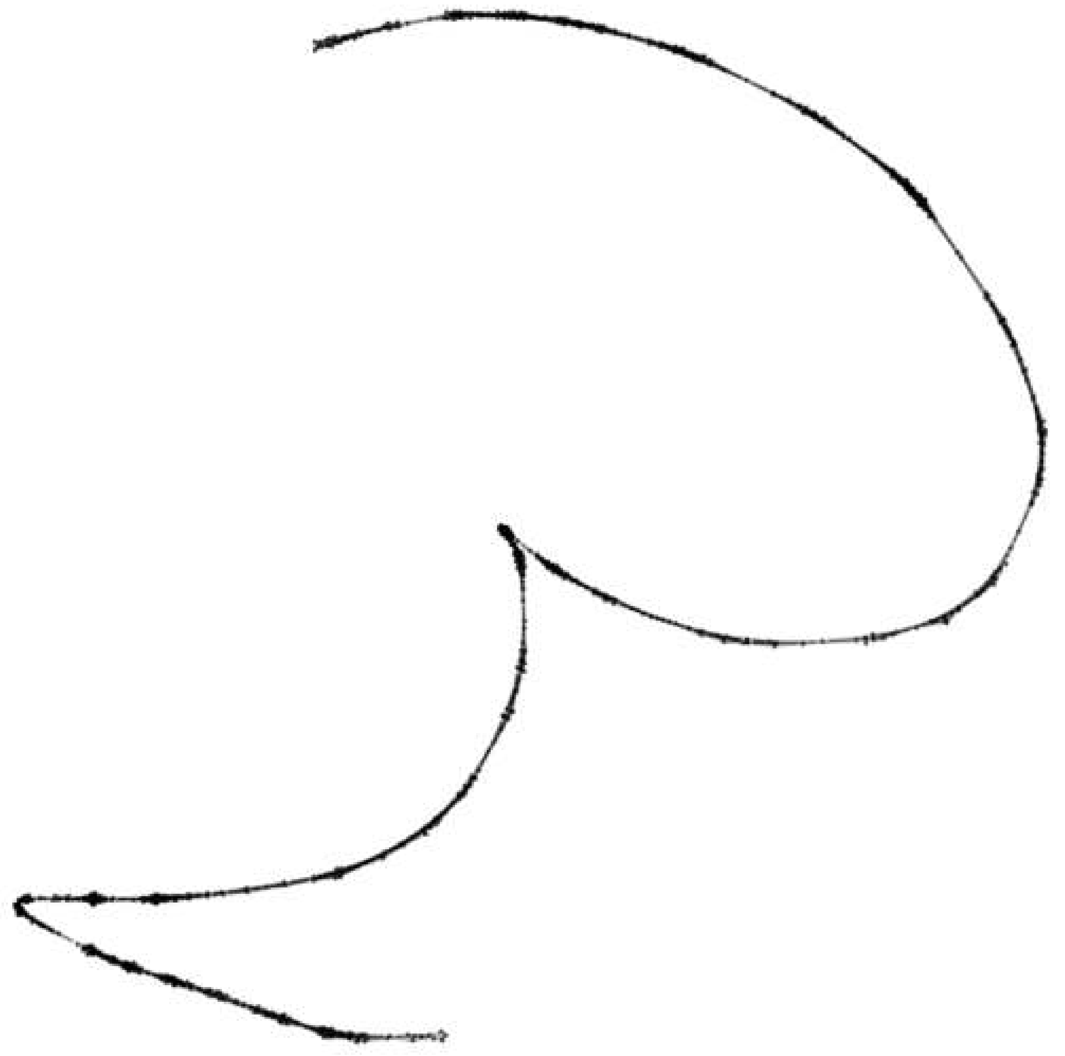

CL66
Low_complexity
Length of Reads (GP):748 (0.06%)

Tgrandiflorum

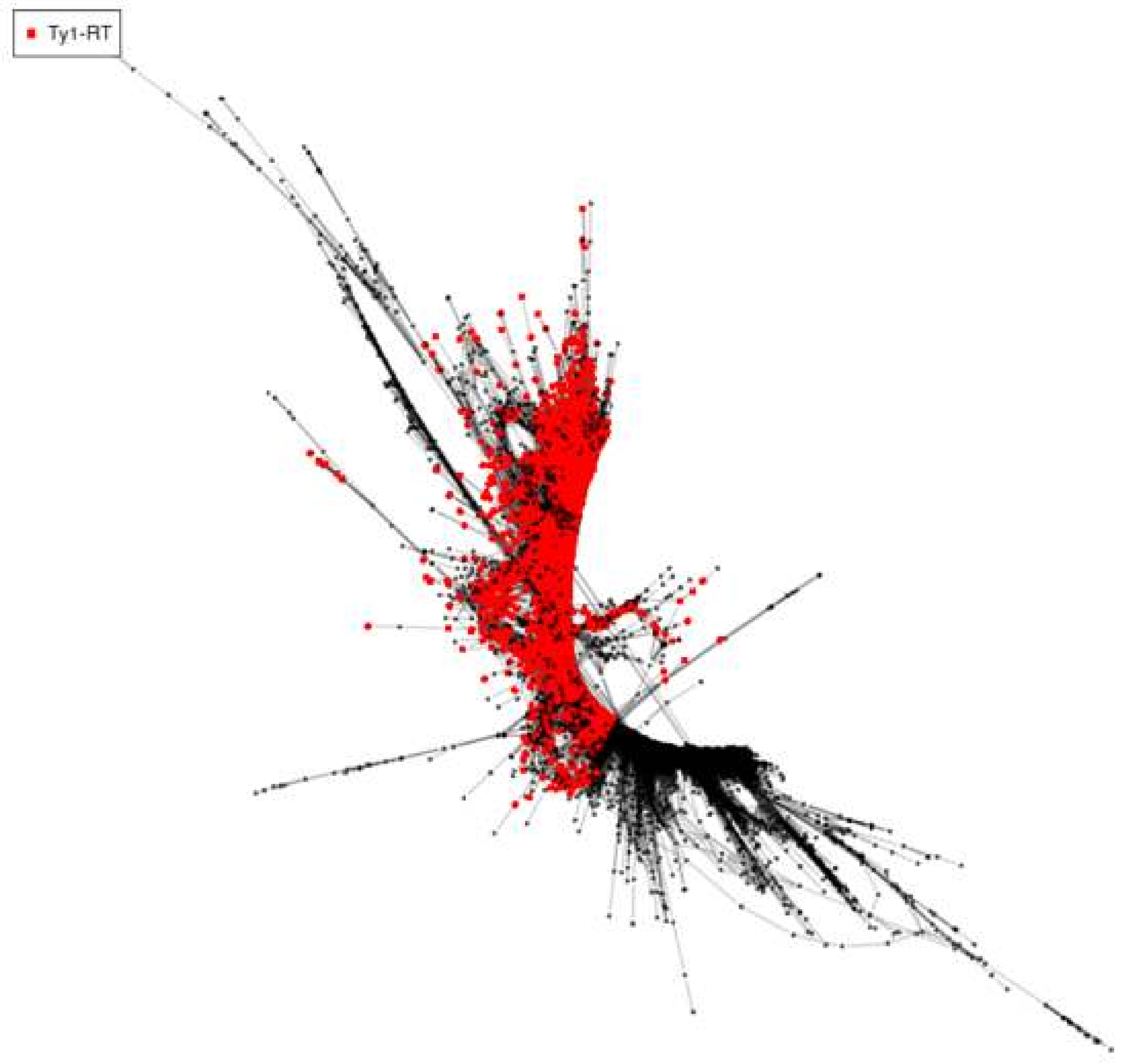

CL66
LTR_Copia
Length of Reads (GP):20781 (0.26%)

Tcacao

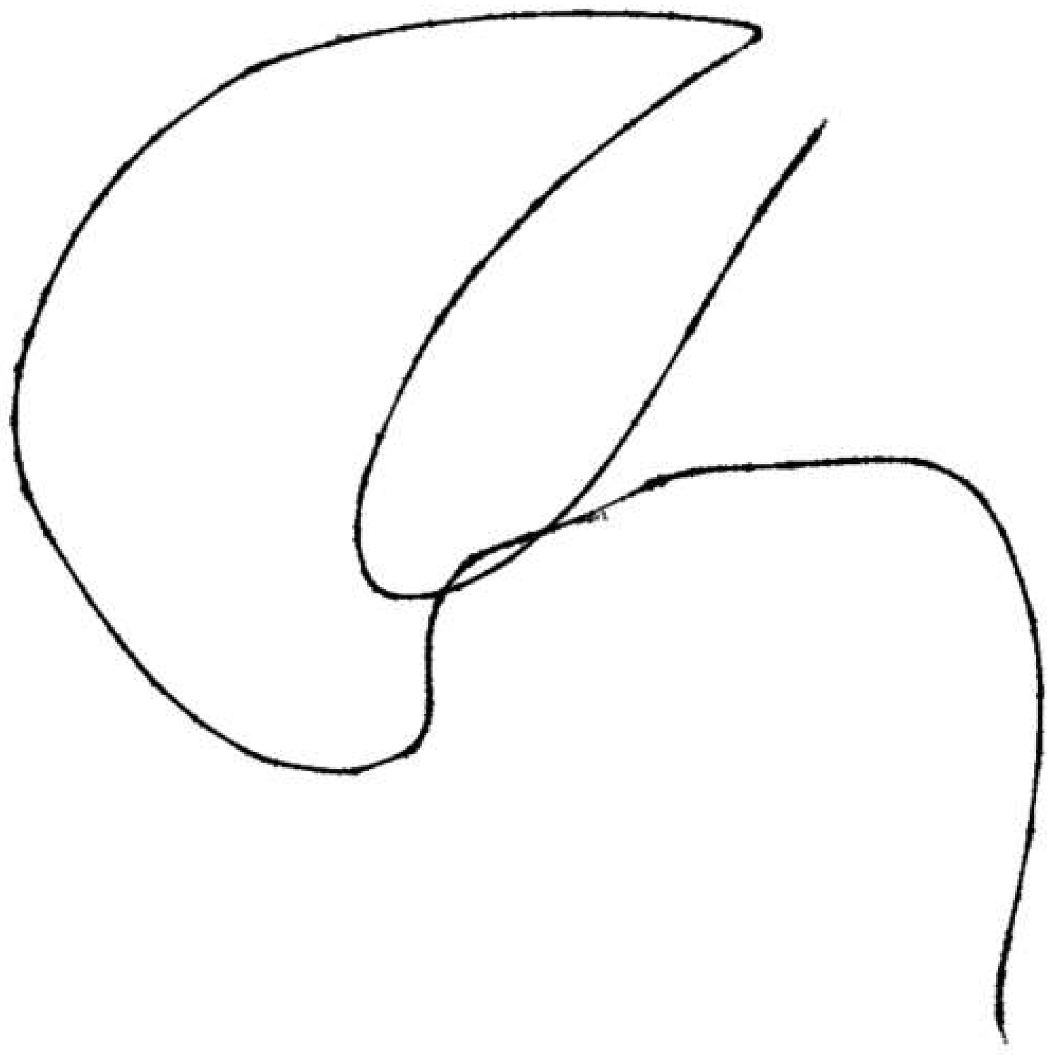

CL66
LTR_Copia
Length of Reads (GP):2877 (0.14%)

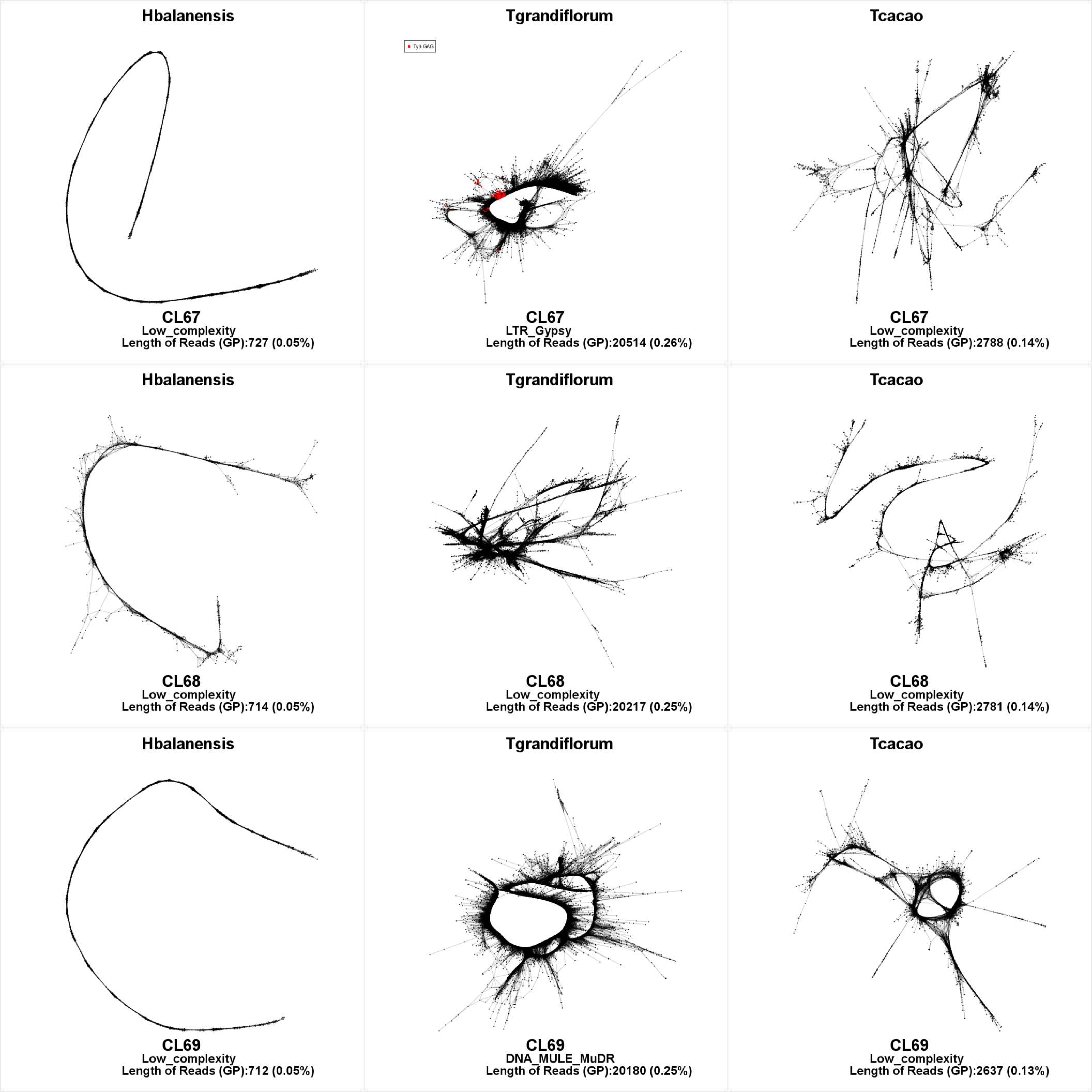
Hbalanensis
CL67
Low_complexity
Length of Reads (GP):727 (0.05%)
Tgrandiflorum
Ty3-GAG
CL67
LTR_Gypsy
Length of Reads (GP):20514 (0.26%)
Tcacao
CL67
Low_complexity
Length of Reads (GP):2788 (0.14%)
Hbalanensis
CL68
Low_complexity
Length of Reads (GP):714 (0.05%)
Tgrandiflorum
CL68
Low_complexity
Length of Reads (GP):20217 (0.25%)
Tcacao
CL68
Low_complexity
Length of Reads (GP):2781 (0.14%)
Hbalanensis
CL69
Low_complexity
Length of Reads (GP):712 (0.05%)
Tgrandiflorum
CL69
DNA_MULE_MuDR
Length of Reads (GP):20180 (0.25%)
Tcacao
CL69
Low_complexity
Length of Reads (GP):2637 (0.13%)

Hbalanensis

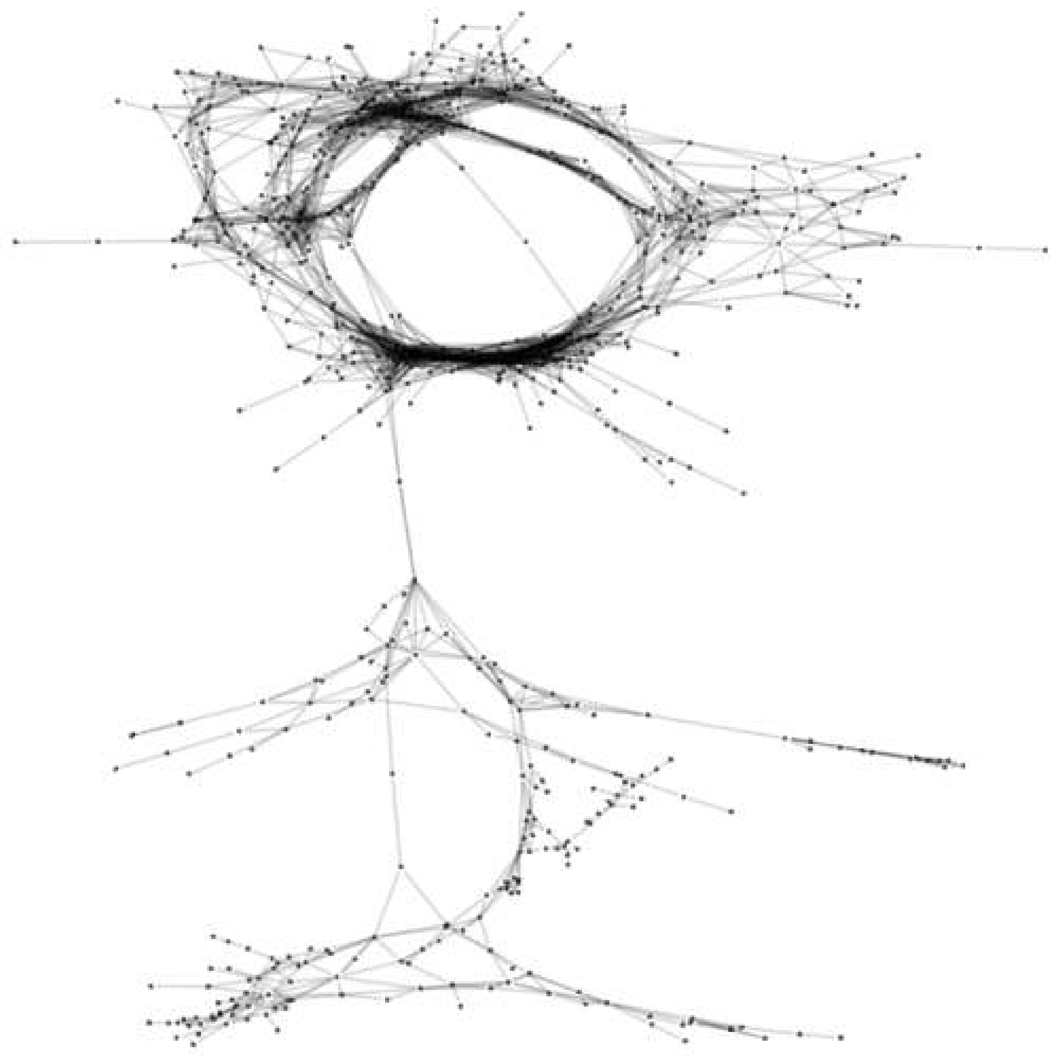

CL70
Low_complexity
Length of Reads (GP):711 (0.05%)

Tgrandiflorum

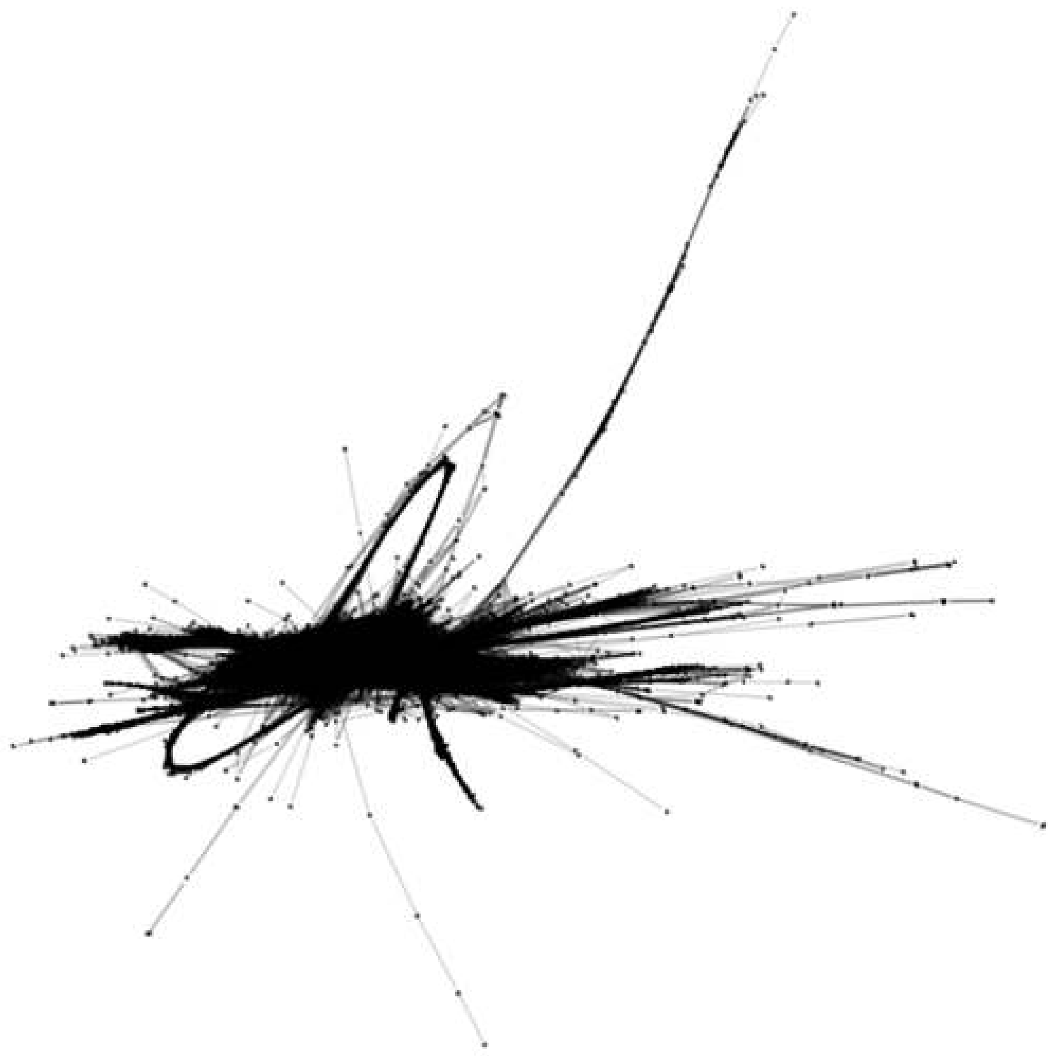

CL70
LTR_Gypsy
Length of Reads (GP):19832 (0.25%)

Tcacao

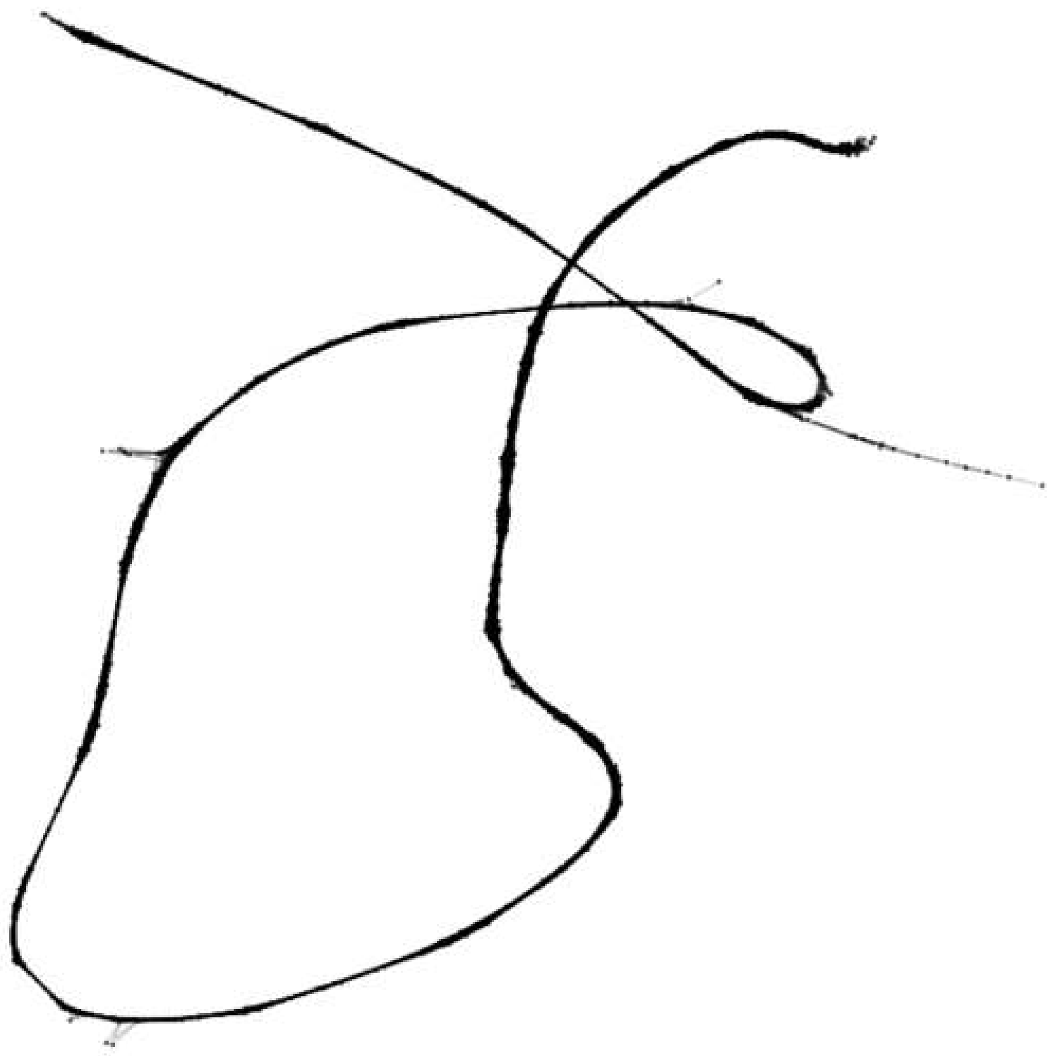

CL70
rRNA
Length of Reads (GP):2617 (0.13%)

Hbalanensis

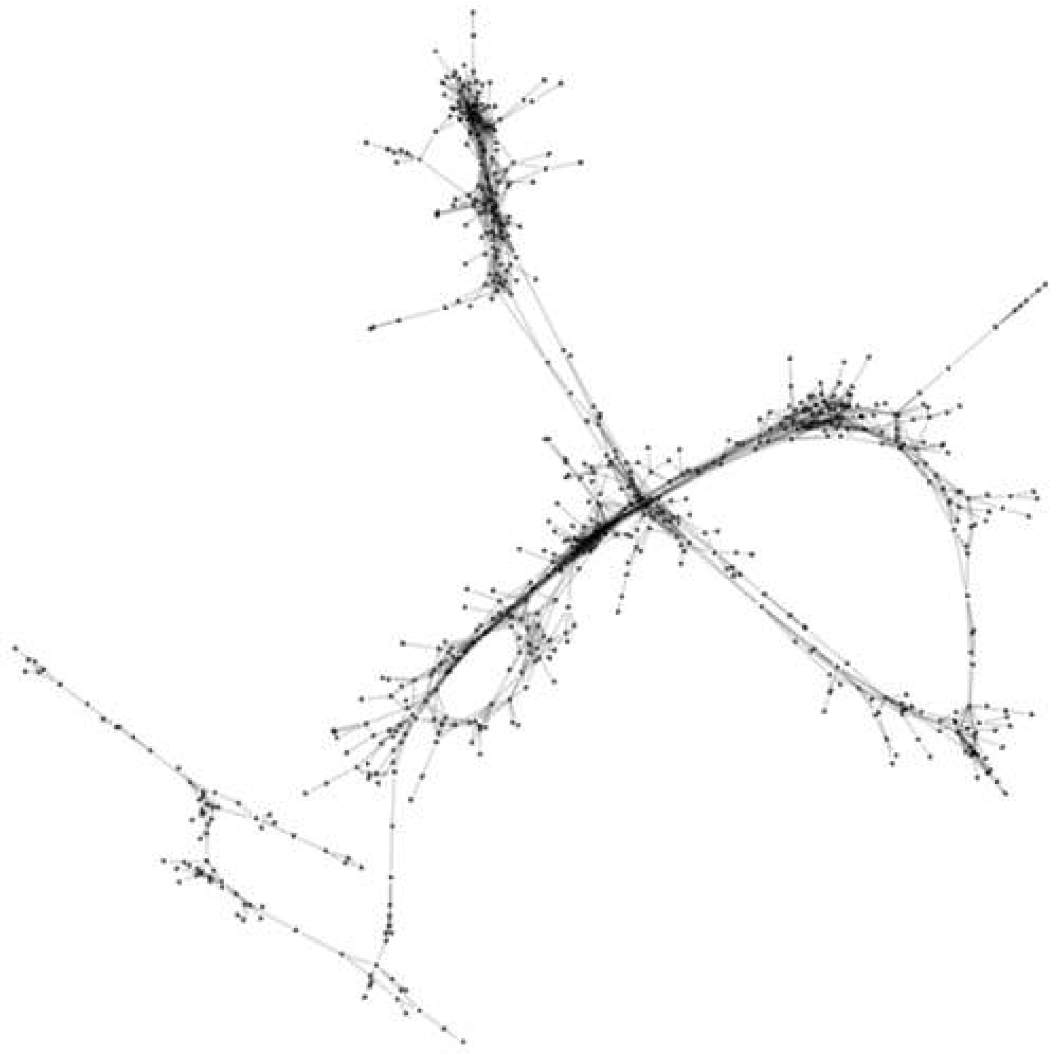

CL71
Low_complexity
Length of Reads (GP):704 (0.05%)

Tgrandiflorum

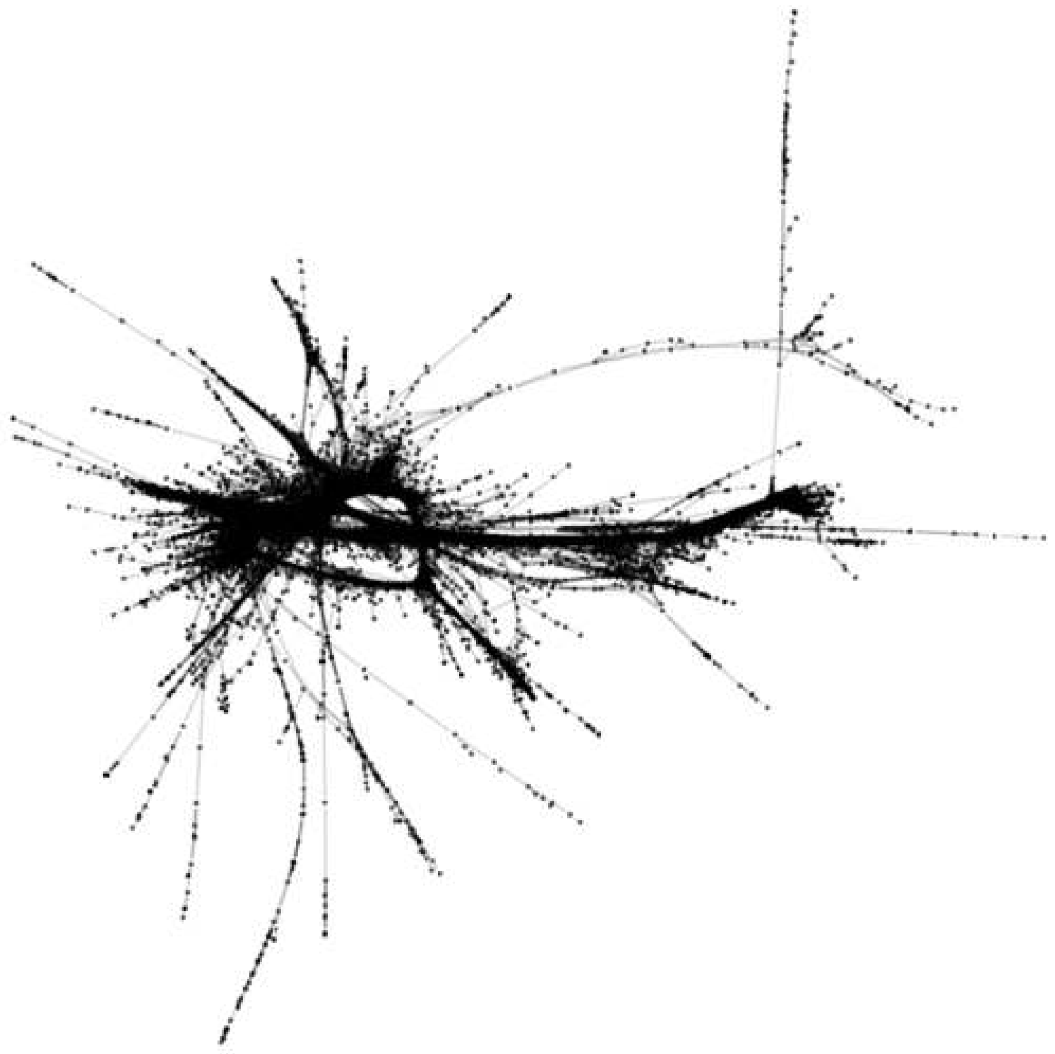

CL71
Low_complexity
Length of Reads (GP):19095 (0.24%)

Tcacao

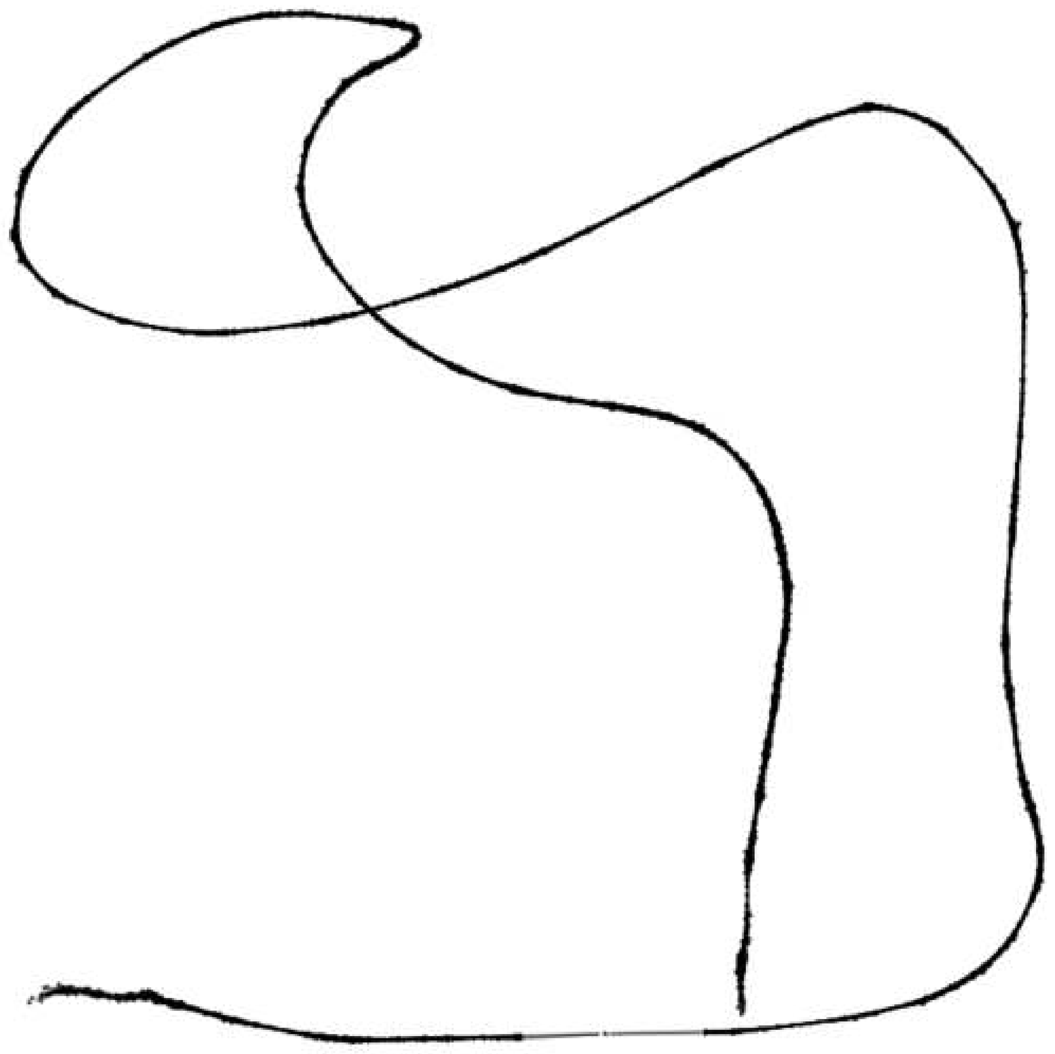

CL71
Low_complexity
Length of Reads (GP):2409 (0.12%)

Hbalanensis

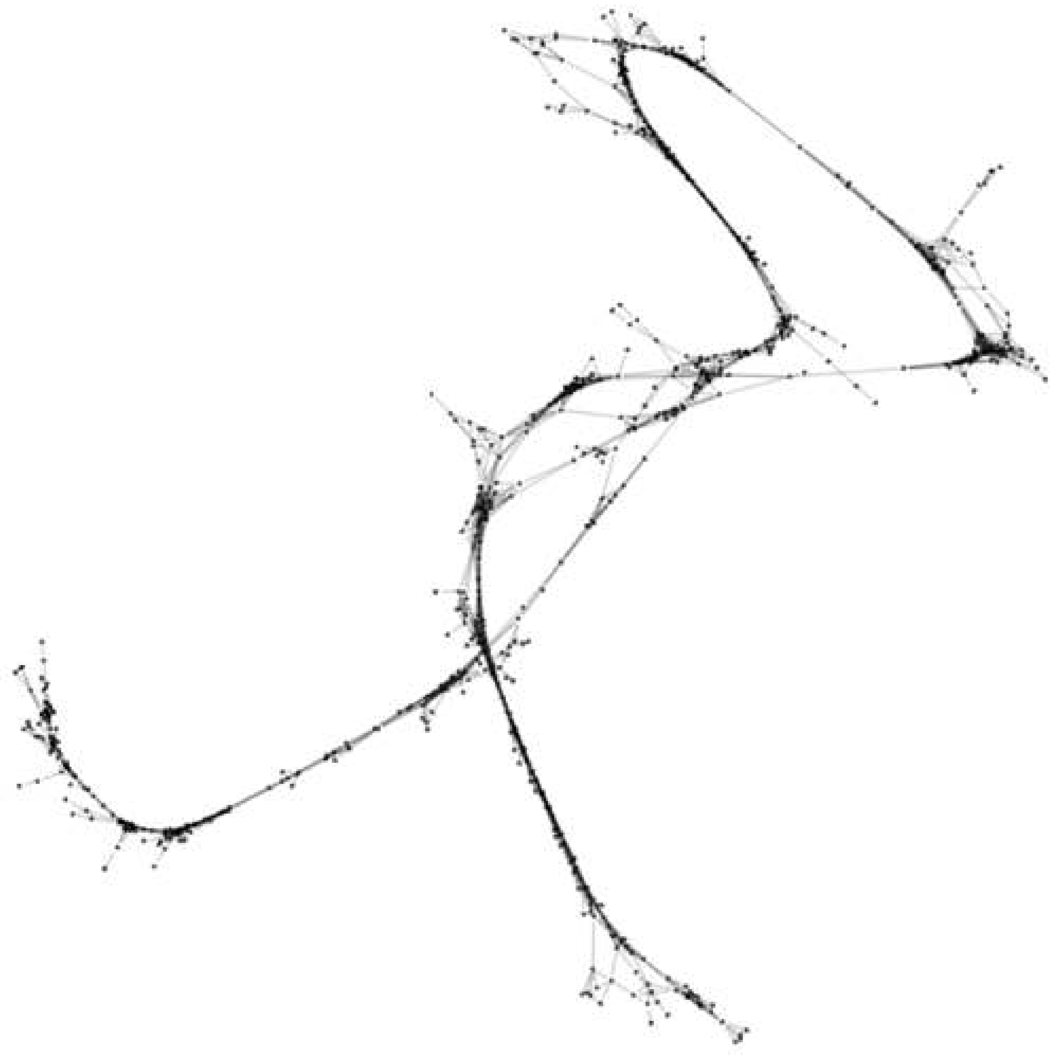

CL72
Low_complexity
Length of Reads (GP):692 (0.05%)

Tgrandiflorum

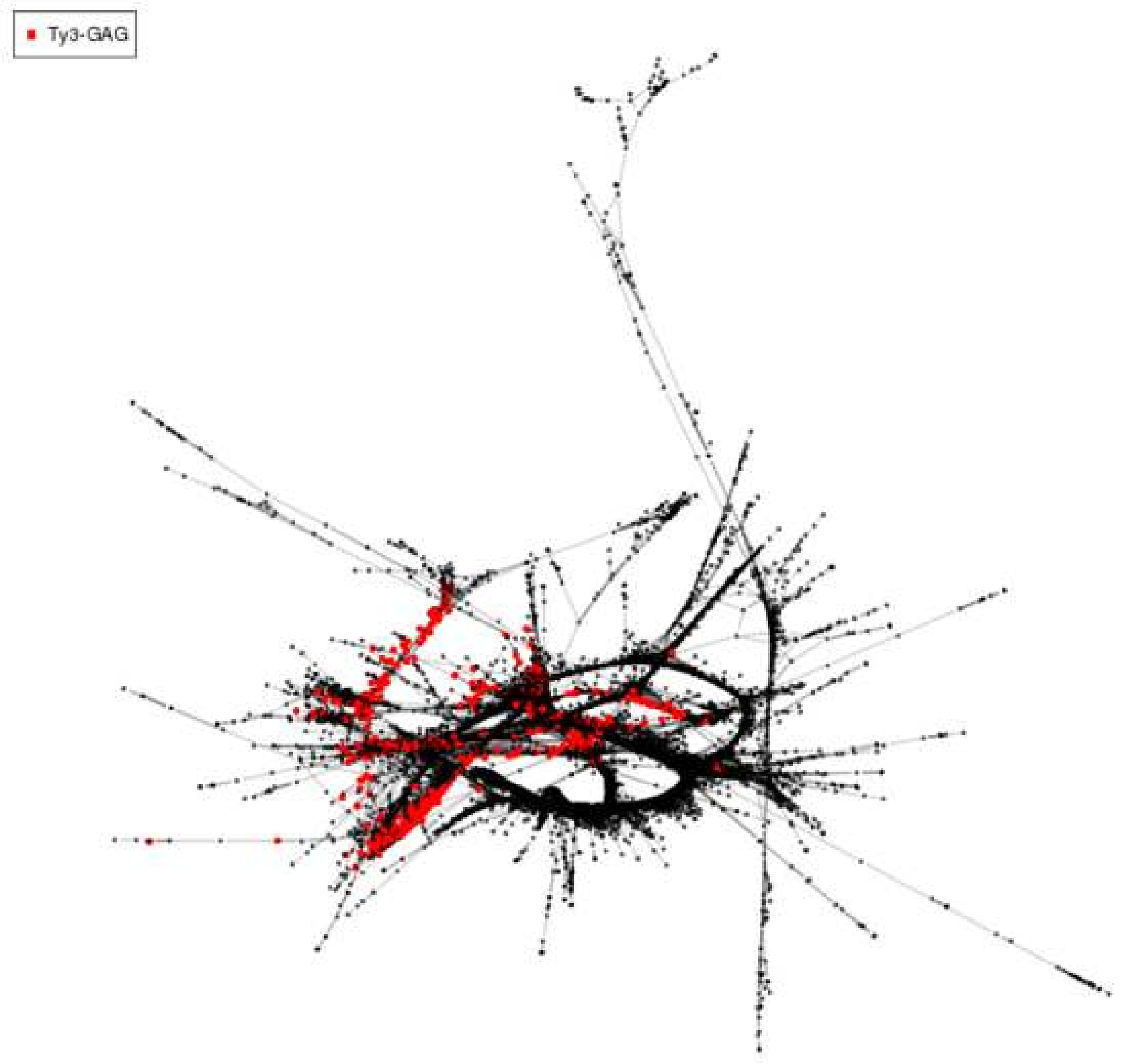

CL72
LTR_Copia
Length of Reads (GP):19040 (0.24%)

Tcacao

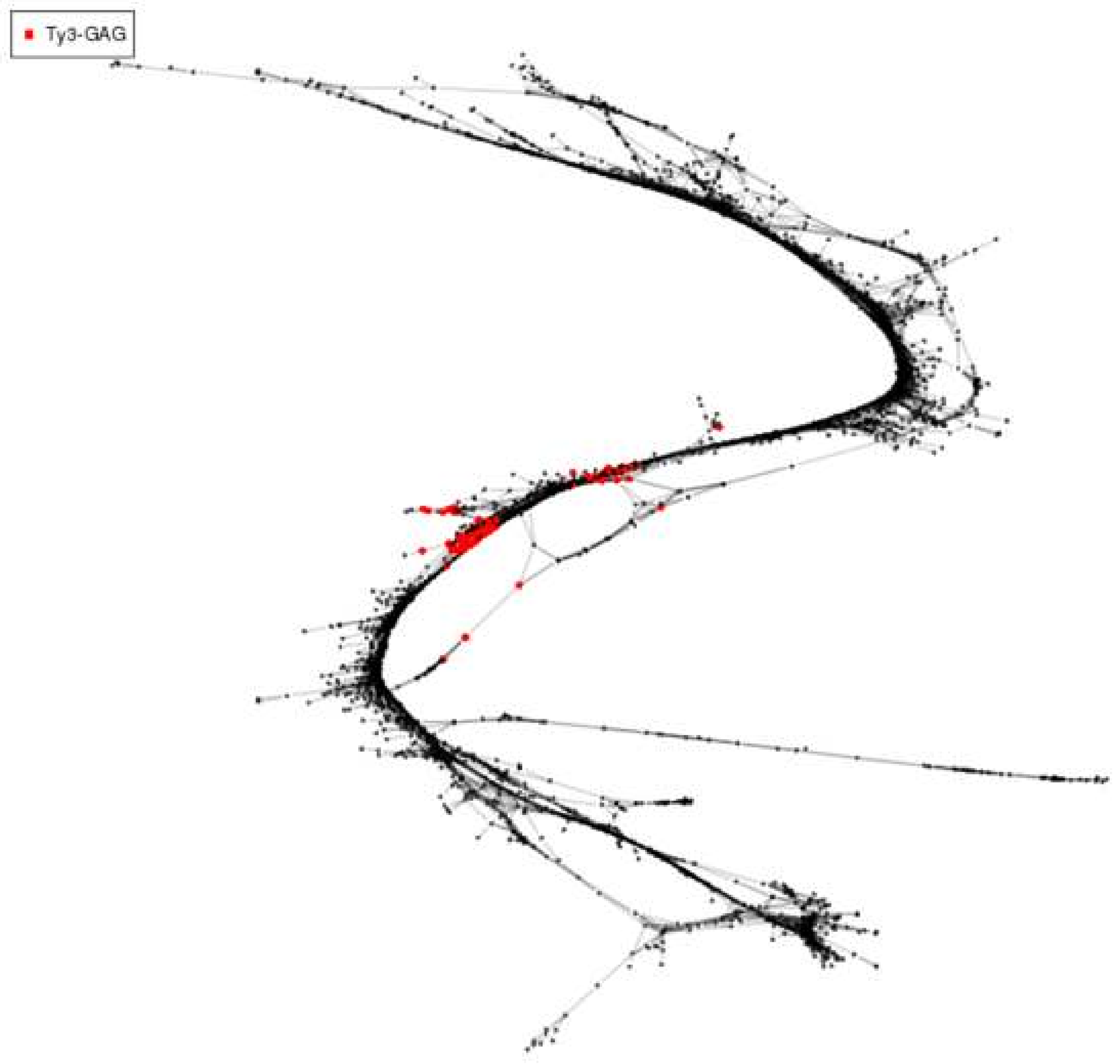

CL72
LTR_Gypsy
Length of Reads (GP):2398 (0.12%)

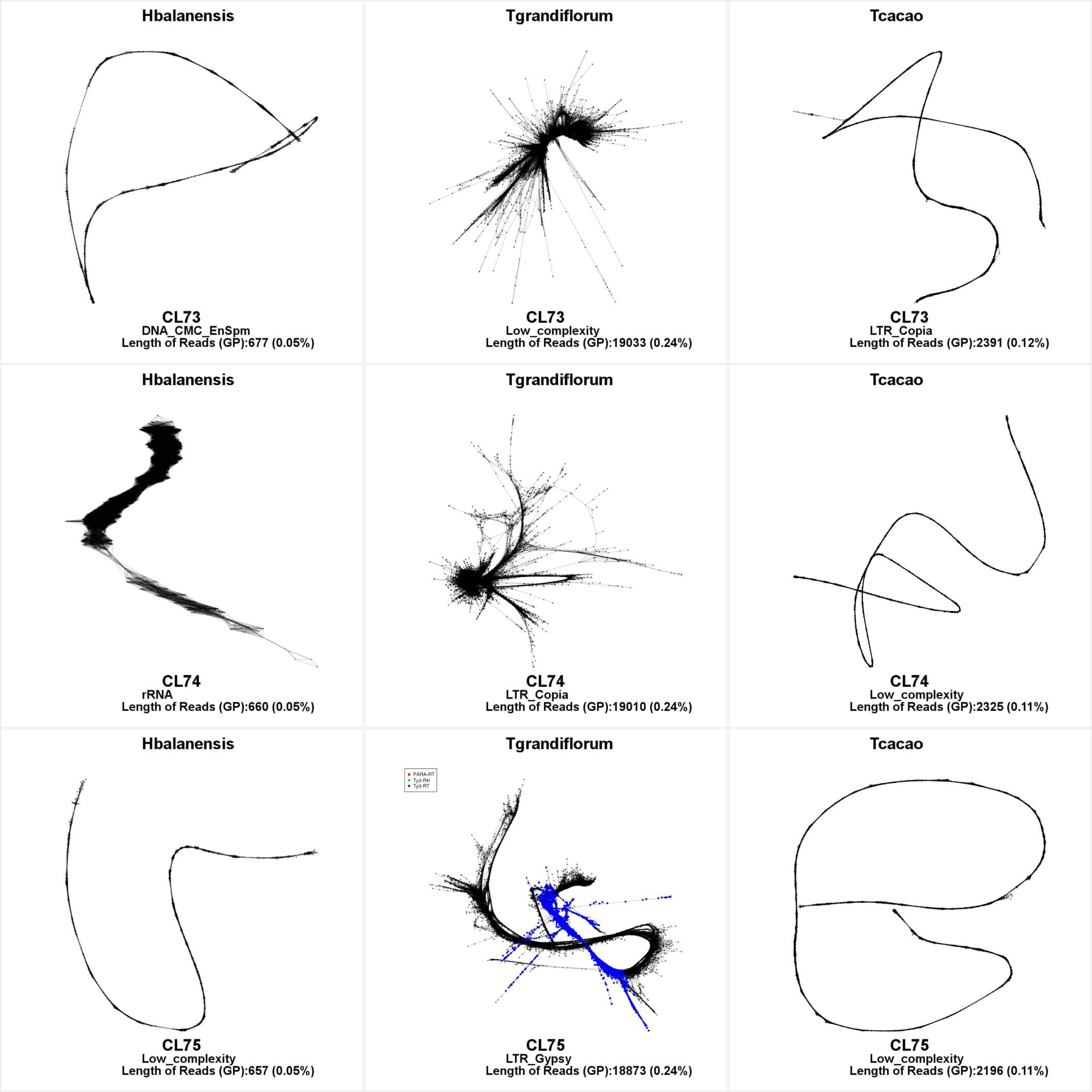
Hbalanensis
CL73
DNA_CMC_EnSpm
Length of Reads (GP):677 (0.05%)
Tgrandiflorum
CL73
Low_complexity
Length of Reads (GP):19033 (0.24%)
Tcacao
CL73
LTR_Copia
Length of Reads (GP):2391 (0.12%)
Hbalanensis
CL74
rRNA
Length of Reads (GP):660 (0.05%)
Tgrandiflorum
CL74
LTR_Copia
Length of Reads (GP):19010 (0.24%)
Tcacao
CL74
Low_complexity
Length of Reads (GP):2325 (0.11%)
Hbalanensis
CL75
Low_complexity
Length of Reads (GP):657 (0.05%)
Tgrandiflorum
PARA-RT
Ty3-RH
Ty3-RT
CL75
LTR_Gypsy
Length of Reads (GP):18873 (0.24%)
Tcacao
CL75
Low_complexity
Length of Reads (GP):2196 (0.11%)

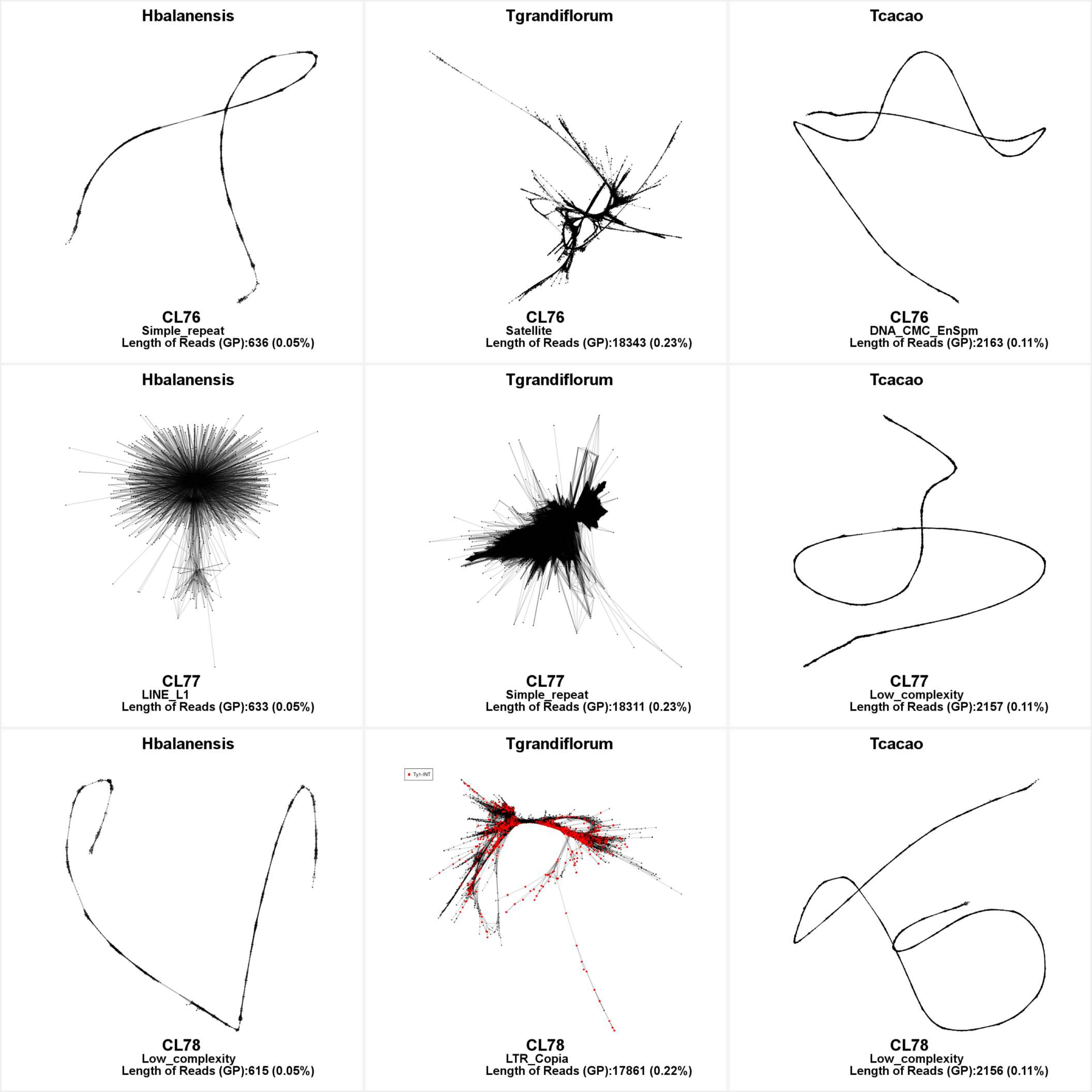
Hbalanensis
CL76
Simple_repeat
Length of Reads (GP):636 (0.05%)
Tgrandiflorum
CL76
Satellite
Length of Reads (GP):18343 (0.23%)
Tcacao
CL76
DNA_CMC_EnSpm
Length of Reads (GP):2163 (0.11%)
Hbalanensis
CL77
LINE_L1
Length of Reads (GP):633 (0.05%)
Tgrandiflorum
CL77
Simple_repeat
Length of Reads (GP):18311 (0.23%)
Tcacao
CL77
Low_complexity
Length of Reads (GP):2157 (0.11%)
Hbalanensis
CL78
Low_complexity
Length of Reads (GP):615 (0.05%)
Tgrandiflorum
Ty1-INT
CL78
LTR_Copia
Length of Reads (GP):17861 (0.22%)
Tcacao
CL78
Low_complexity
Length of Reads (GP):2156 (0.11%)

Hbalanensis

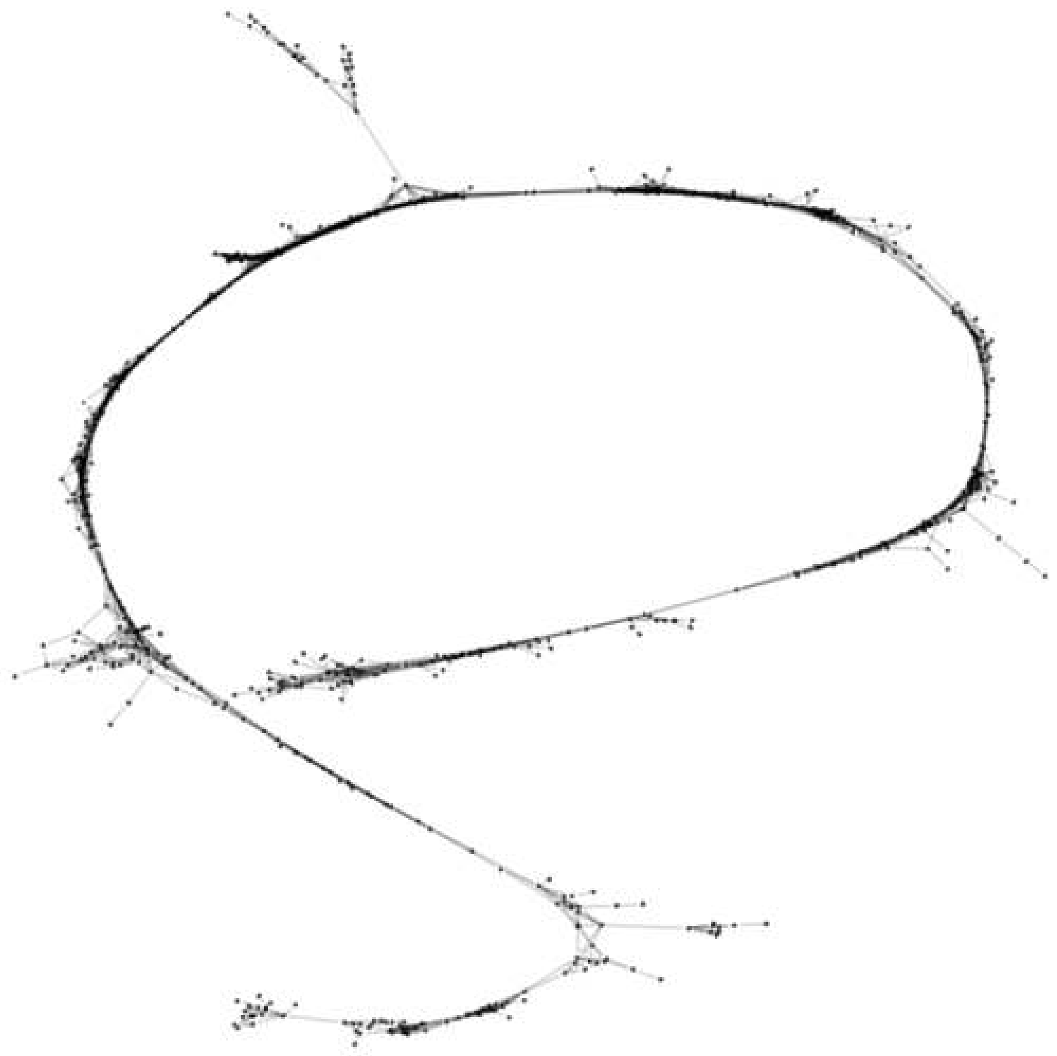

CL79
LTR_Copia
Length of Reads (GP):612 (0.05%)

Tgrandiflorum

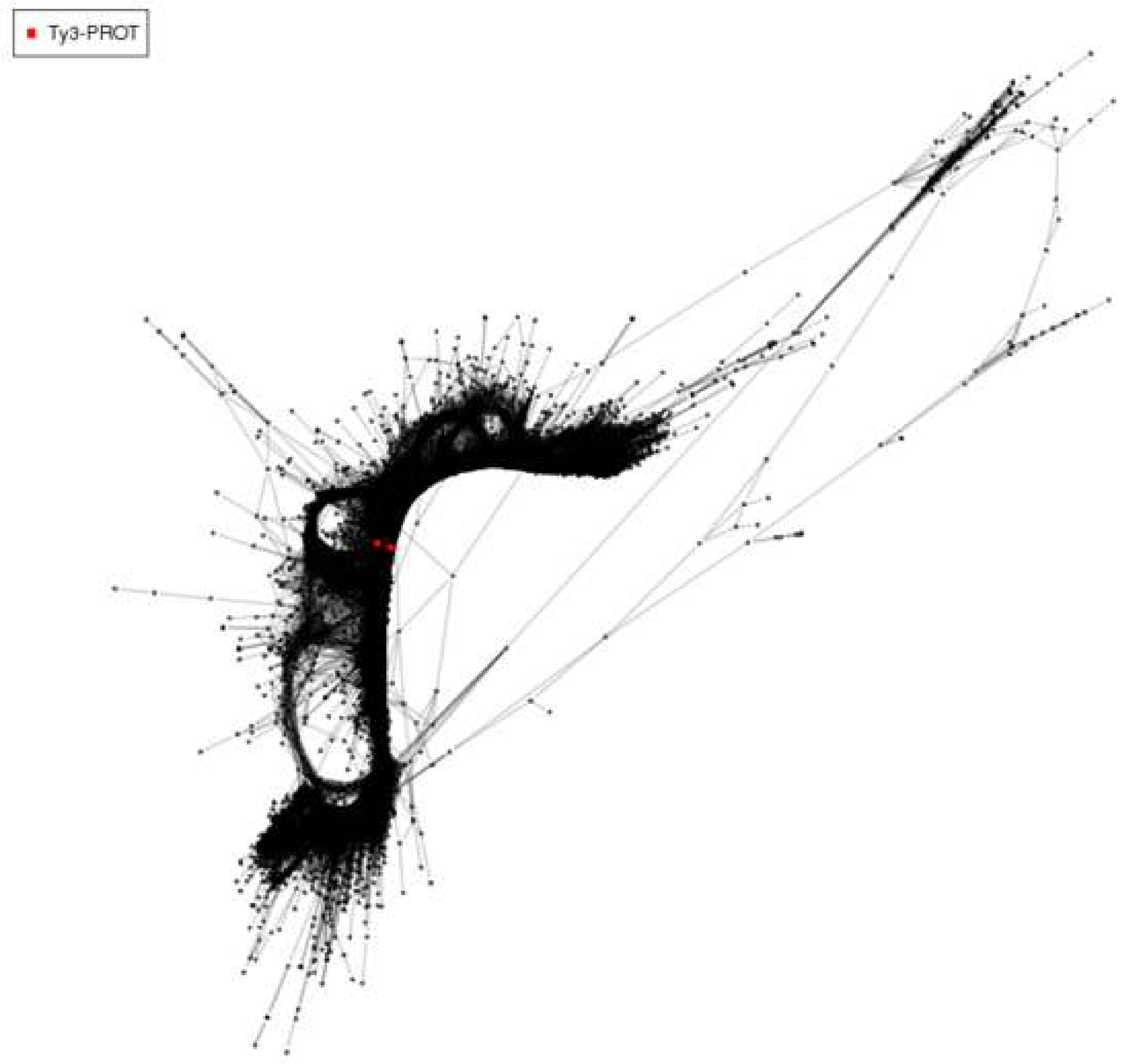

CL79
LTR_Gypsy
Length of Reads (GP):17641 (0.22%)

Tcacao

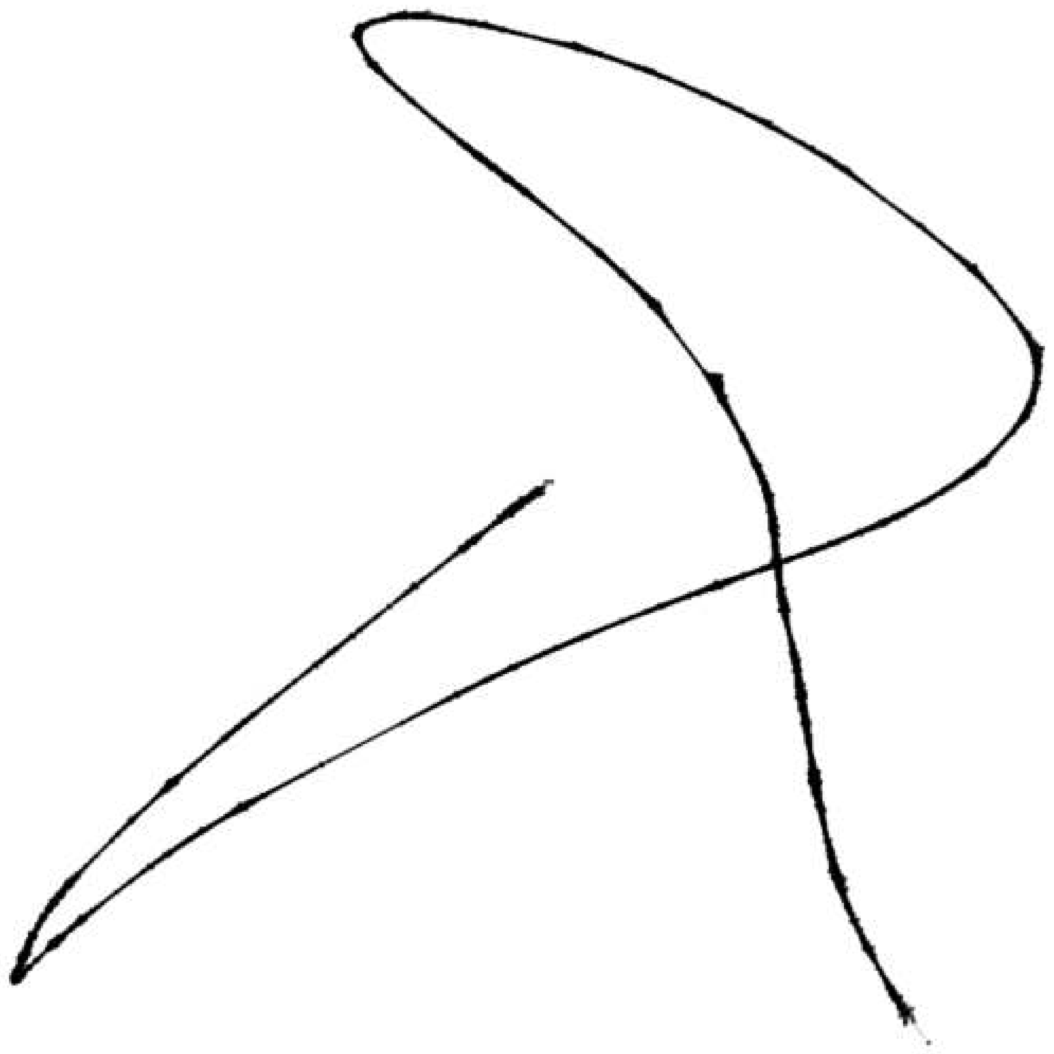

CL79
Low_complexity
Length of Reads (GP):2148 (0.11%)

Hbalanensis

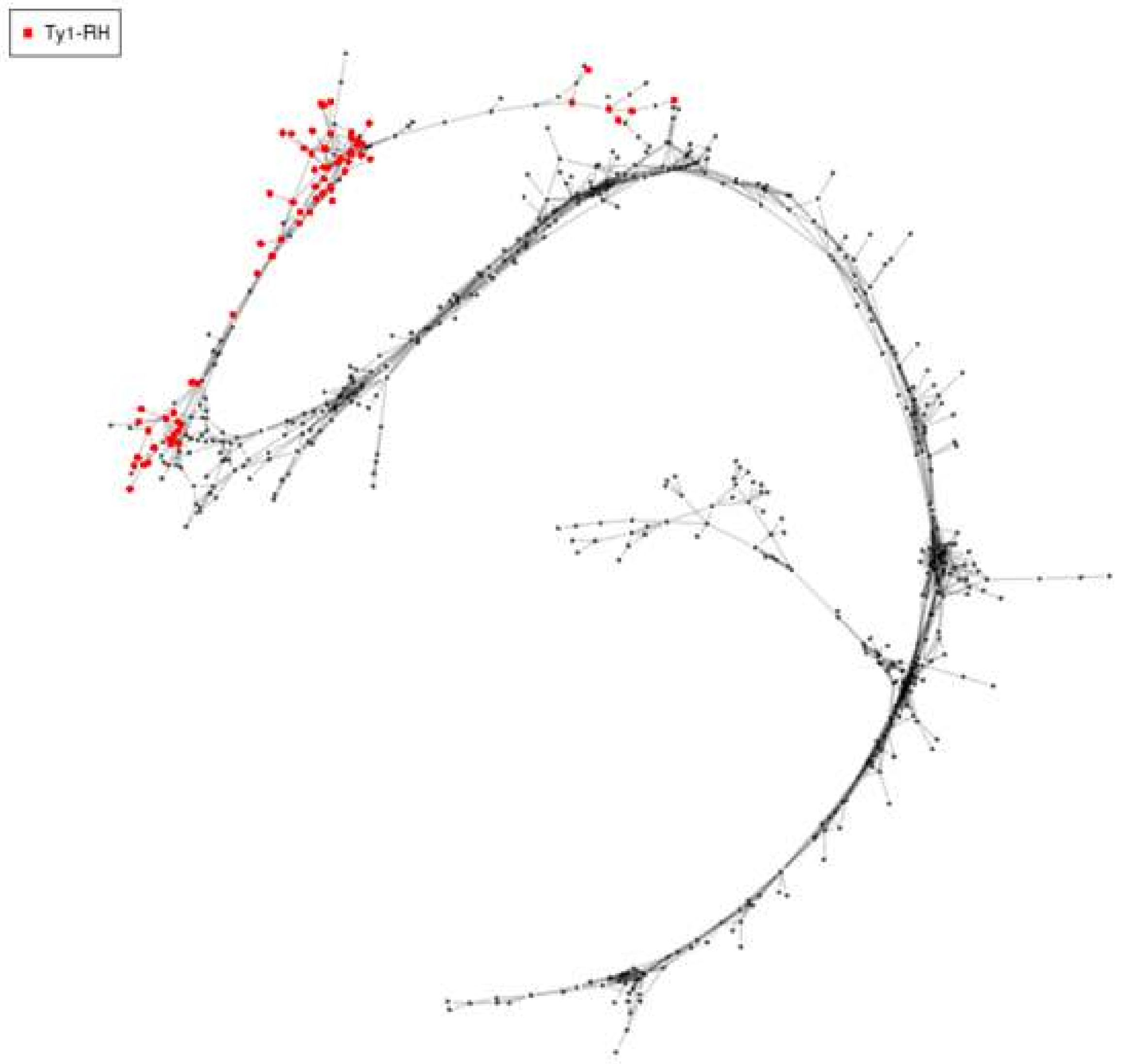

CL80
LTR_Copia
Length of Reads (GP):601 (0.05%)

Tgrandiflorum

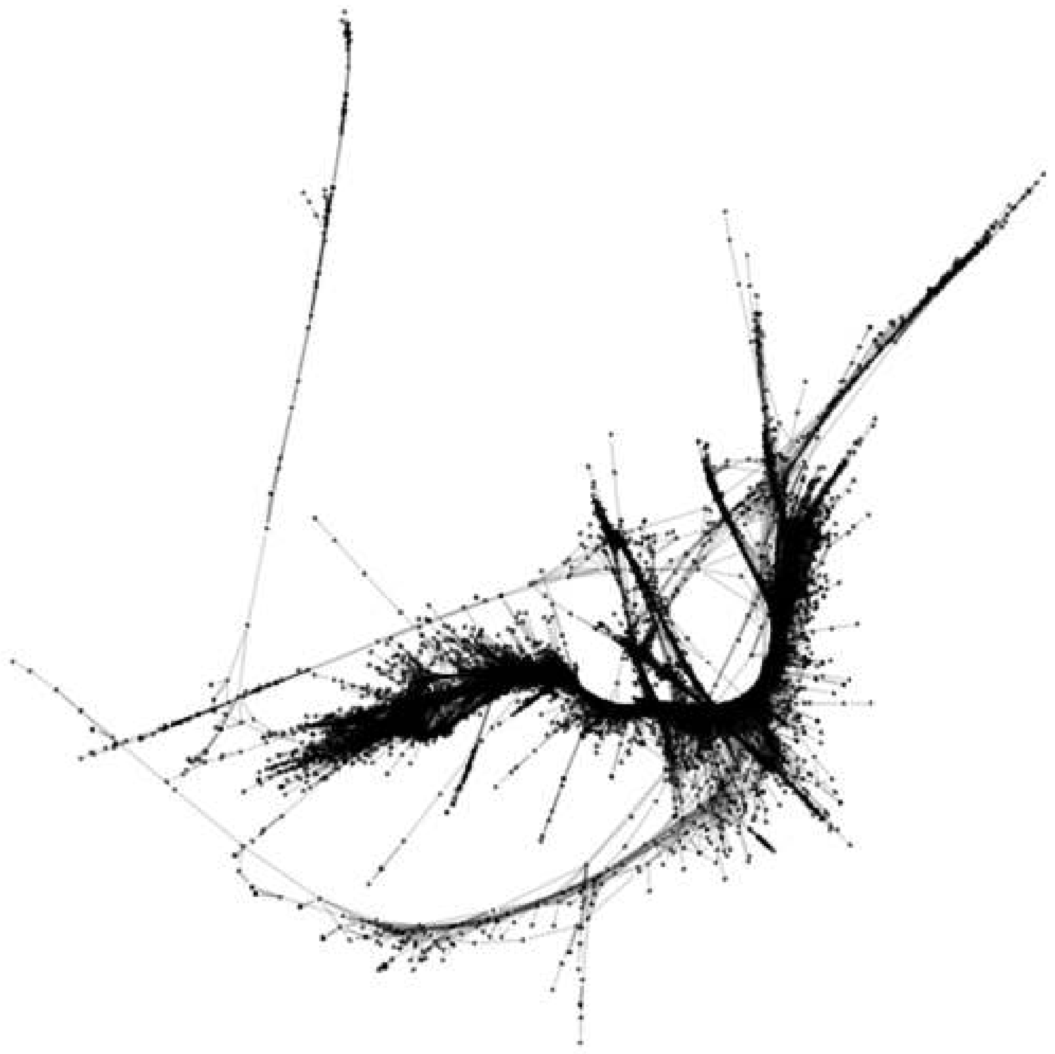

CL80
Low_complexity
Length of Reads (GP):17292 (0.22%)

Tcacao

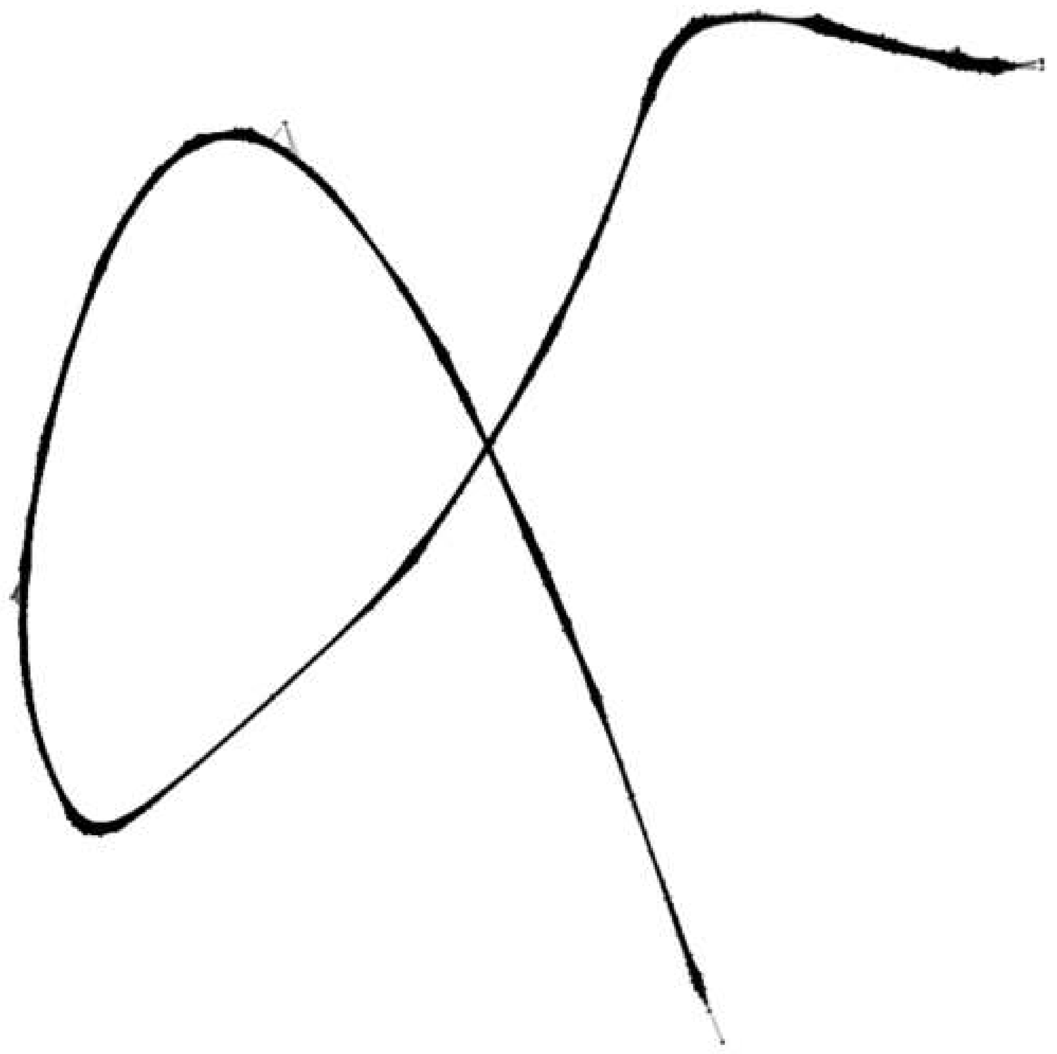

CL80
rRNA
Length of Reads (GP):2145 (0.11%)

Hbalanensis

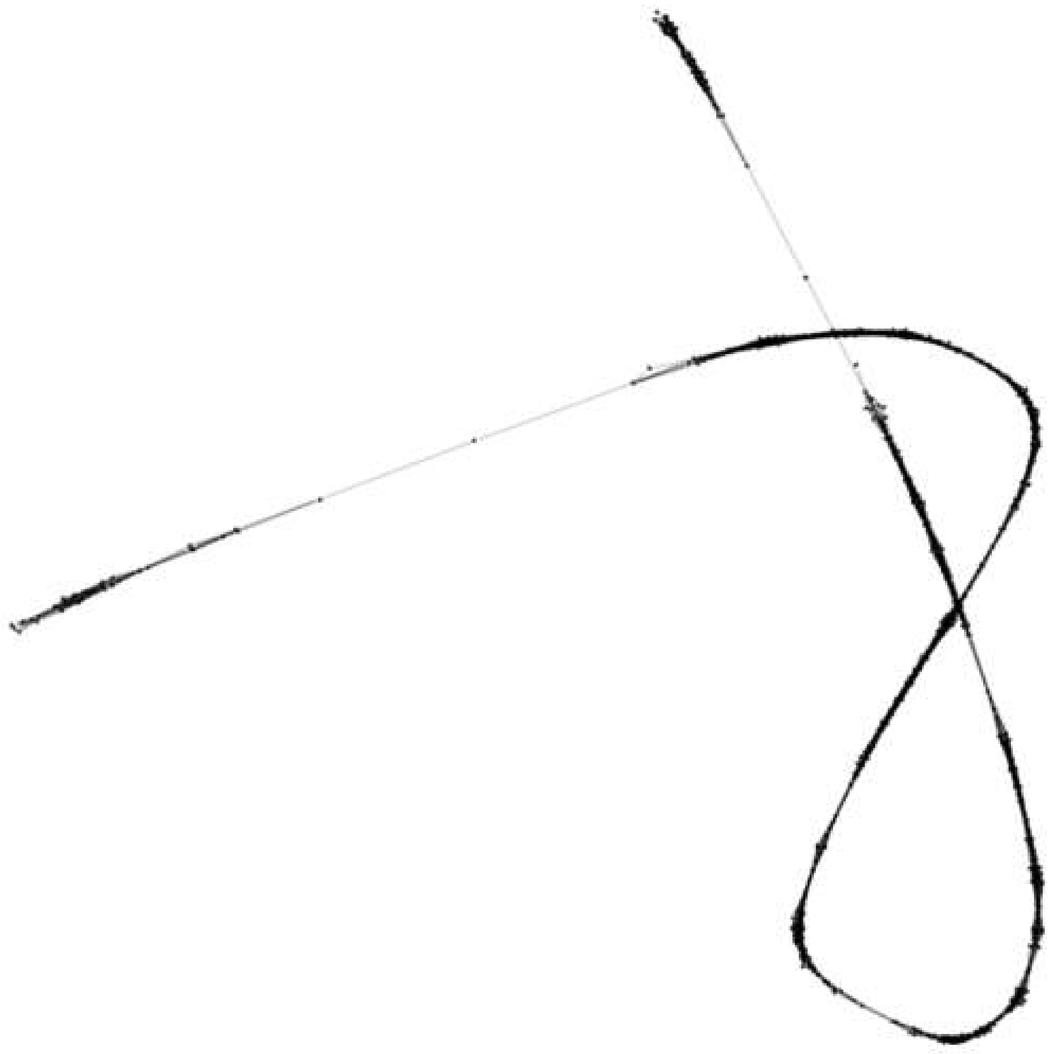

CL81
Low_complexity
Length of Reads (GP):579 (0.04%)

Tgrandiflorum

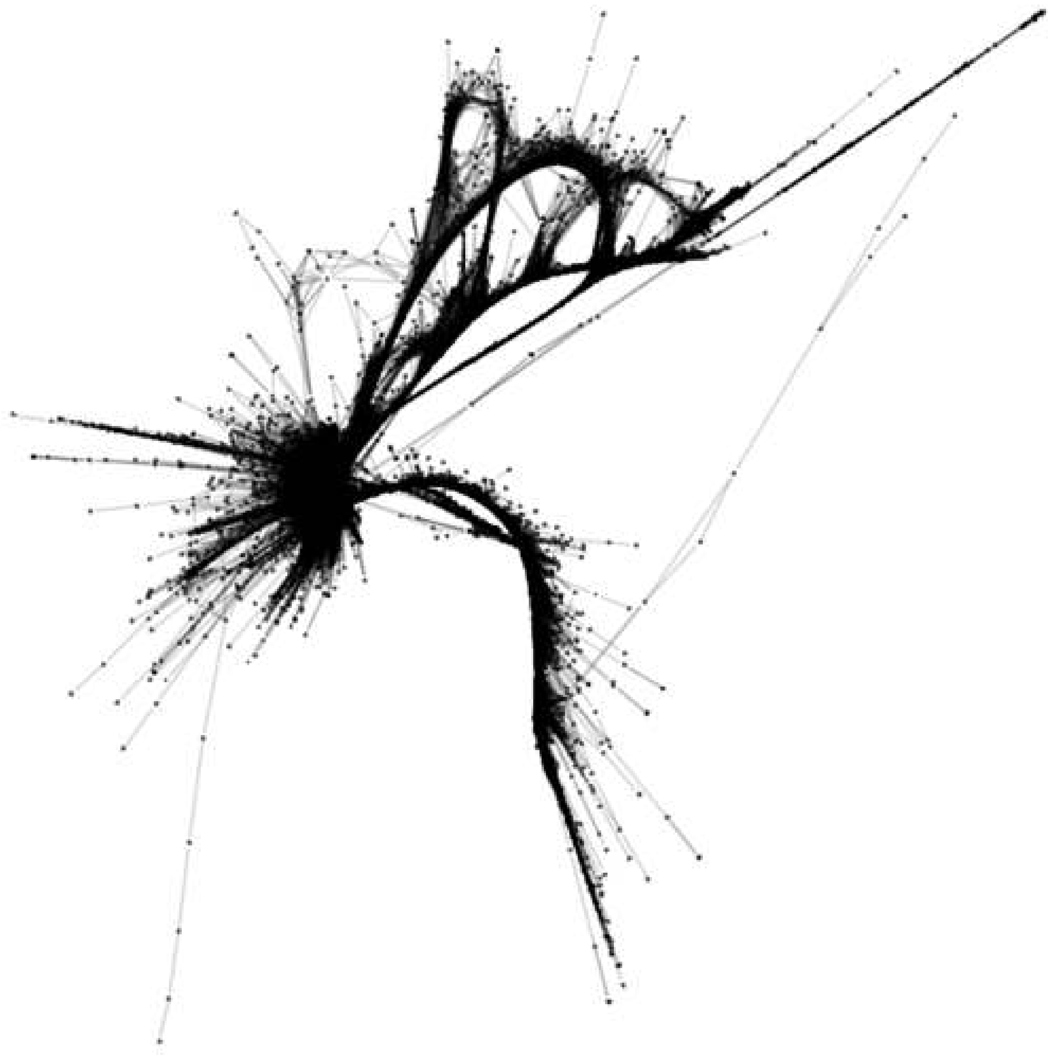

CL81
Low_complexity
Length of Reads (GP):17068 (0.21%)

Tcacao

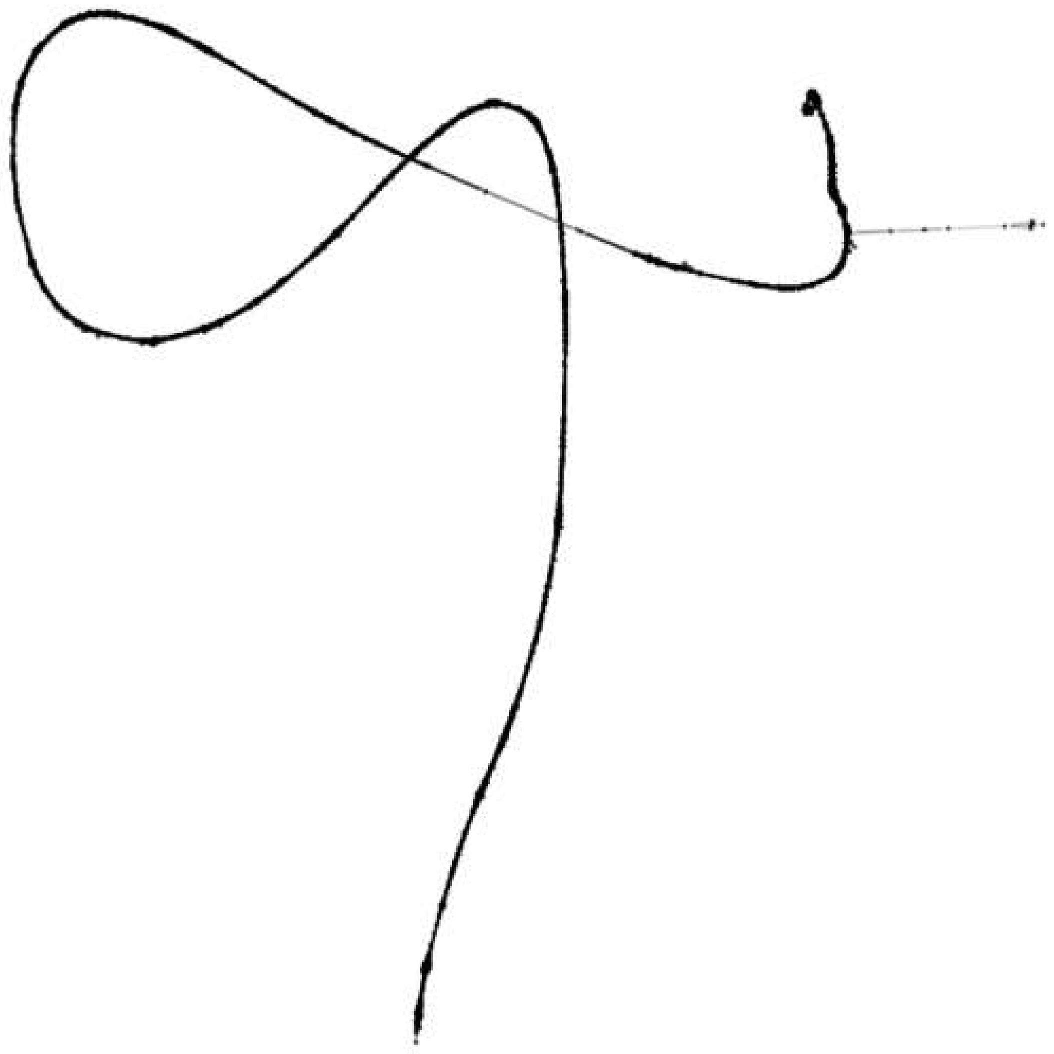

CL81
Low_complexity
Length of Reads (GP):2087 (0.1%)

Hbalanensis

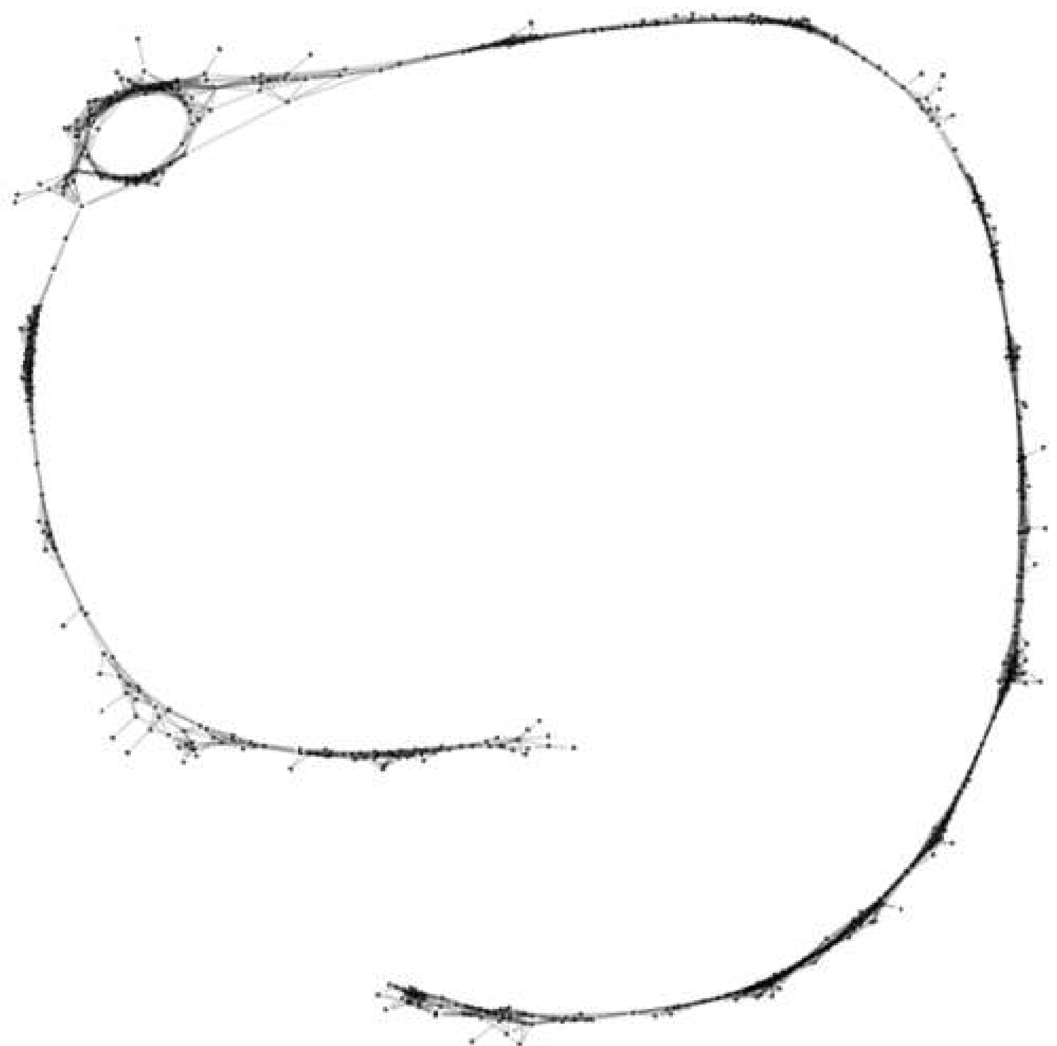

CL82
Low_complexity
Length of Reads (GP):566 (0.04%)

Tgrandiflorum

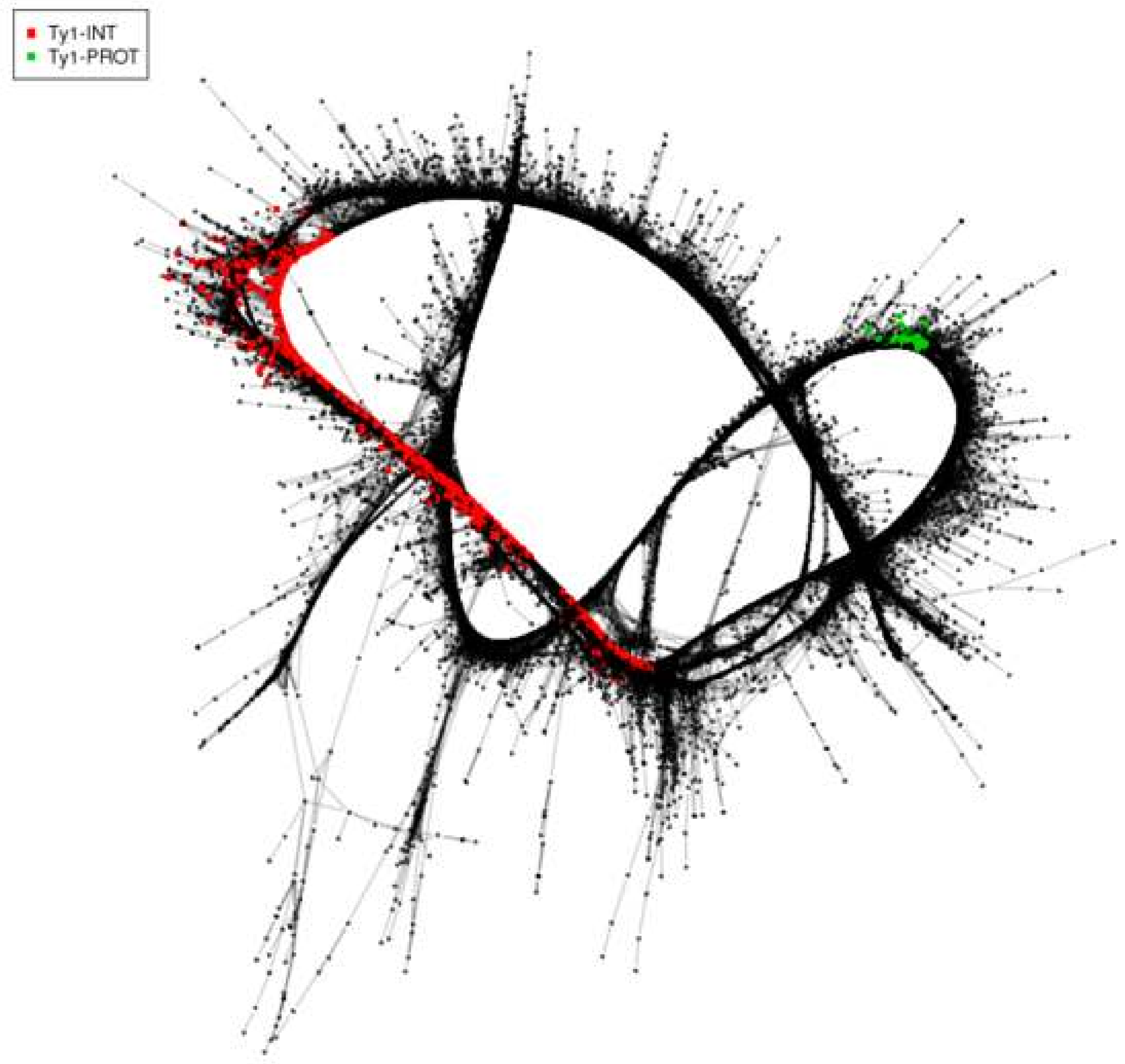

CL82
LTR_Copia
Length of Reads (GP):17037 (0.21%)

Tcacao

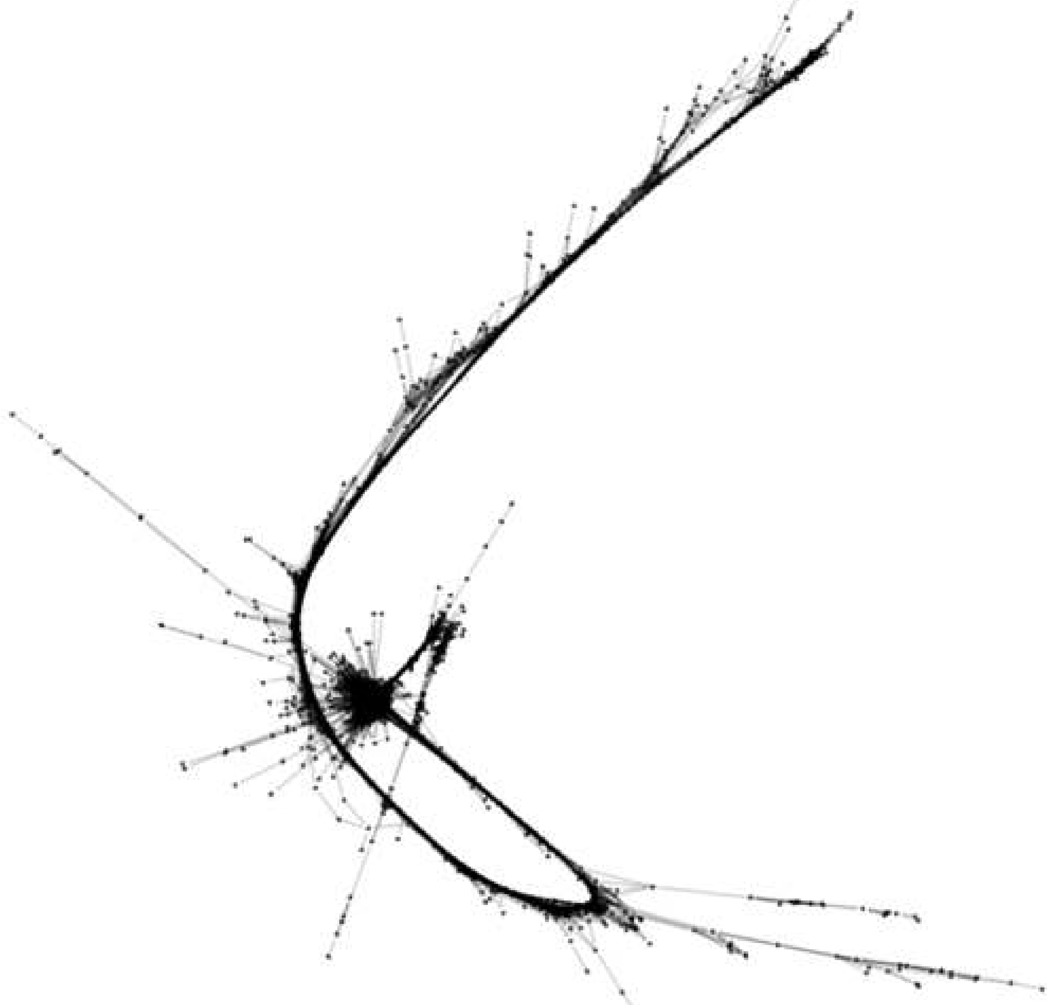

CL82
Low_complexity
Length of Reads (GP):2066 (0.1%)

Hbalanensis

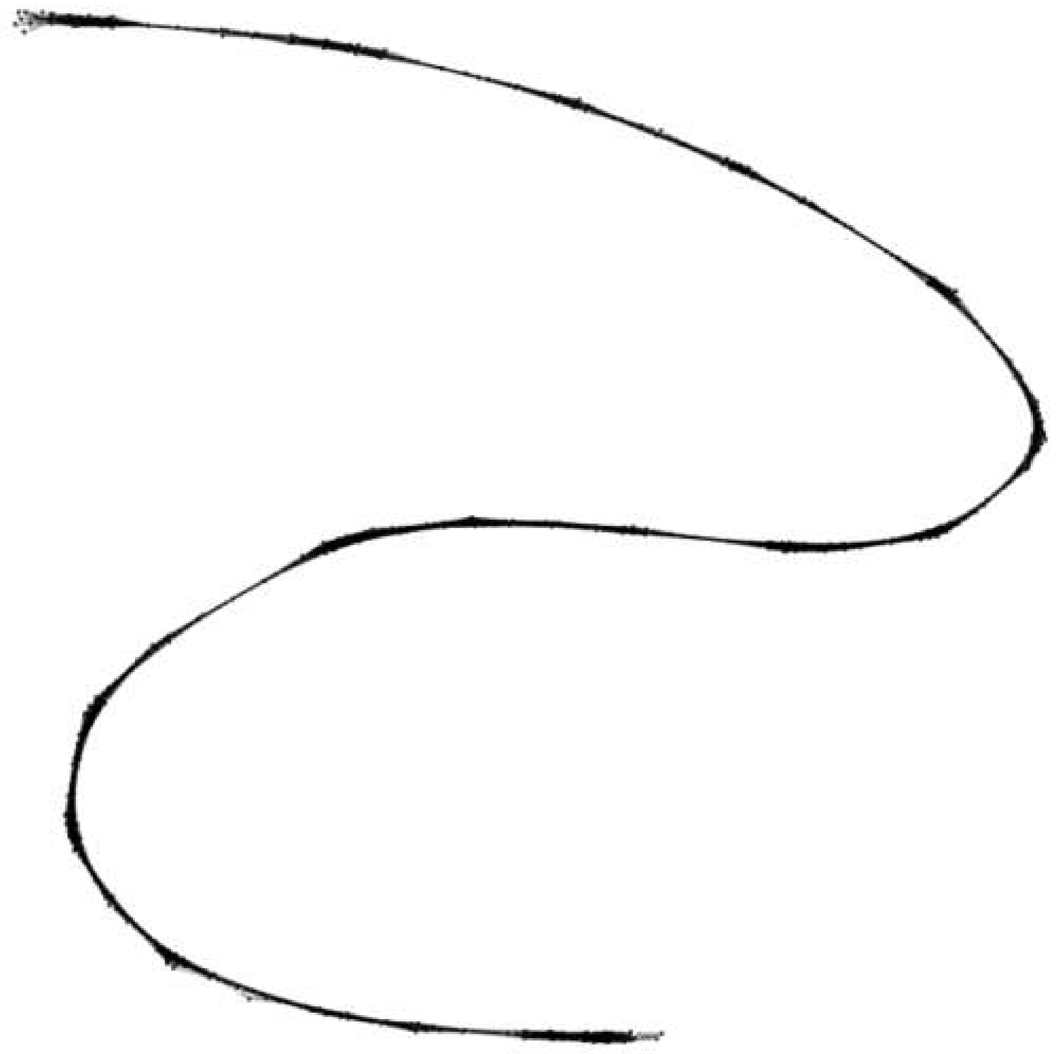

CL83
Low_complexity
Length of Reads (GP):564 (0.04%)

Tgrandiflorum

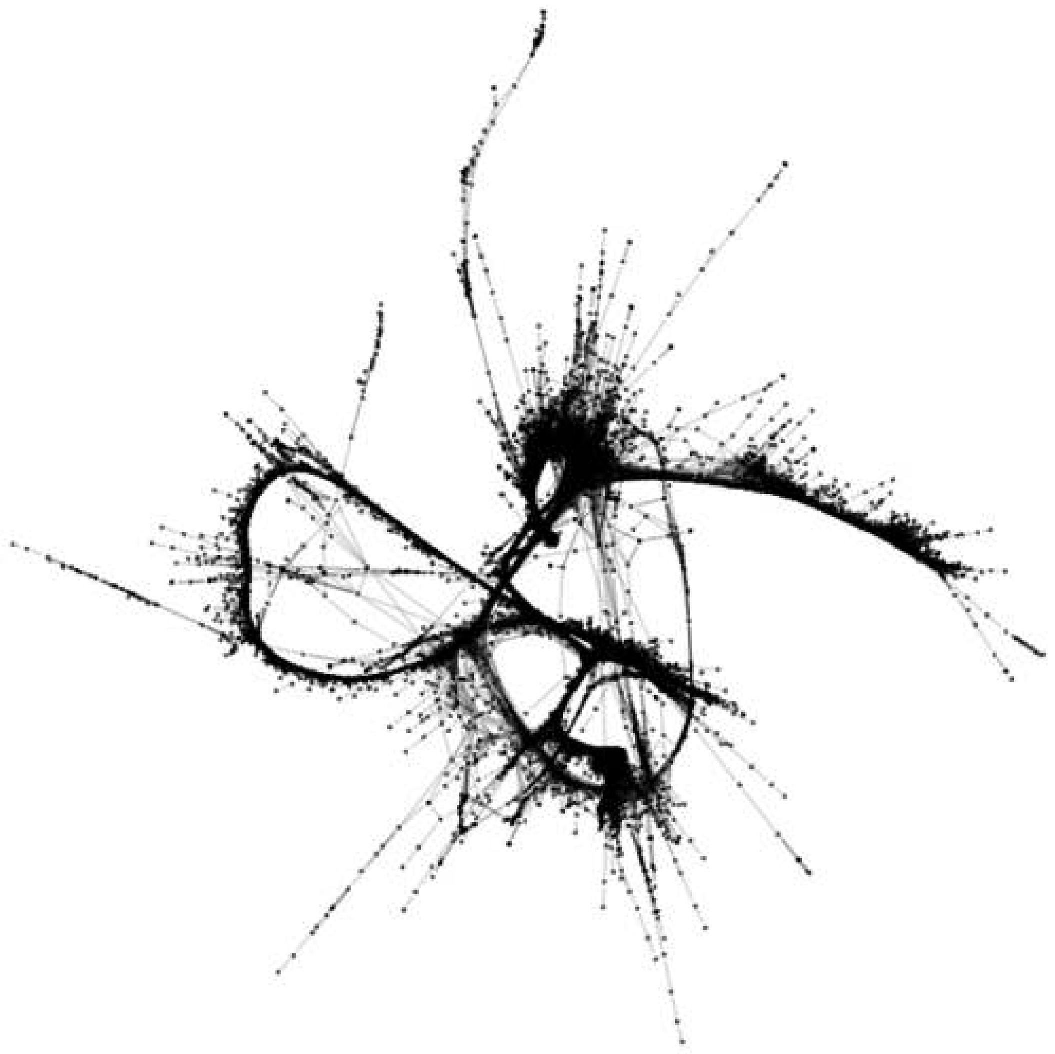

CL83
Low_complexity
Length of Reads (GP):16914 (0.21%)

Tcacao

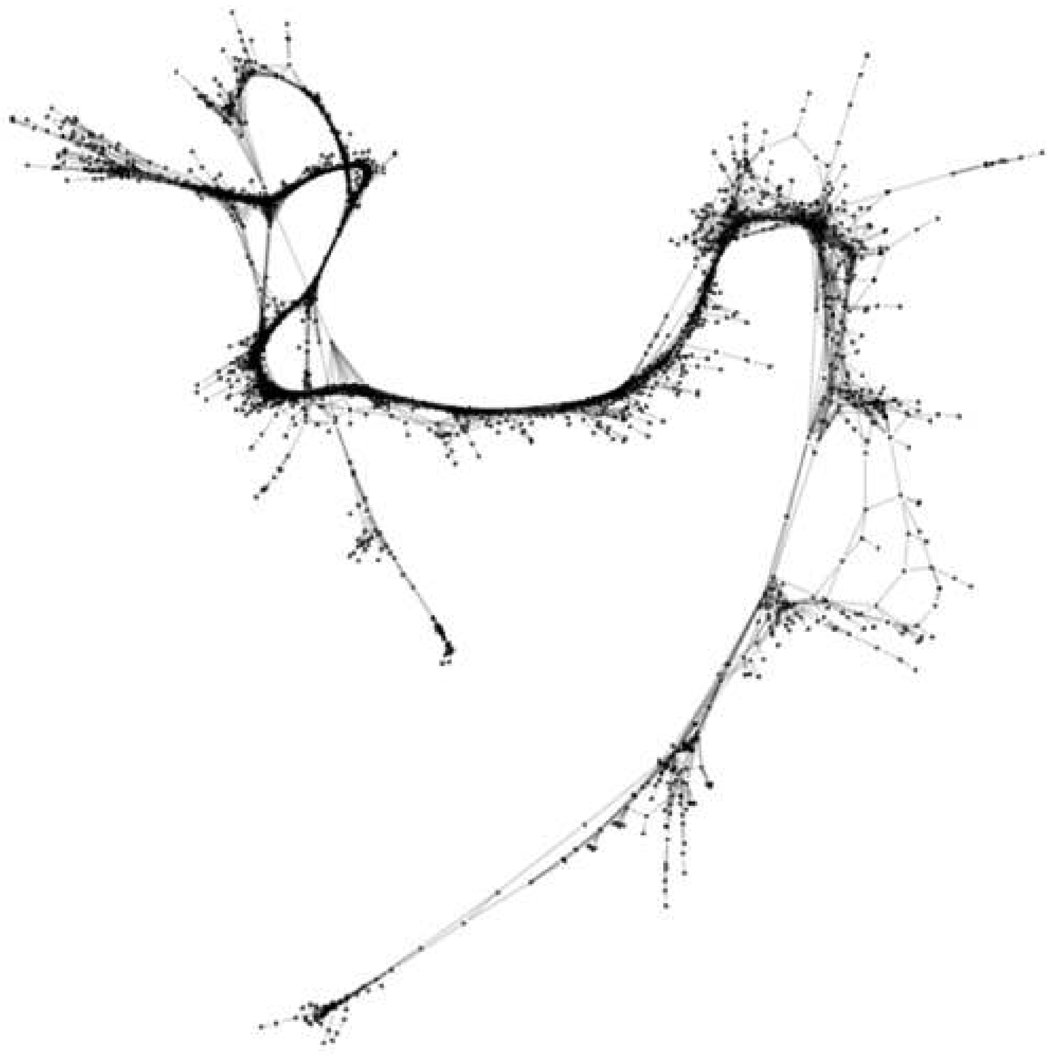

CL83
Low_complexity
Length of Reads (GP):1976 (0.1%)

Hbalanensis

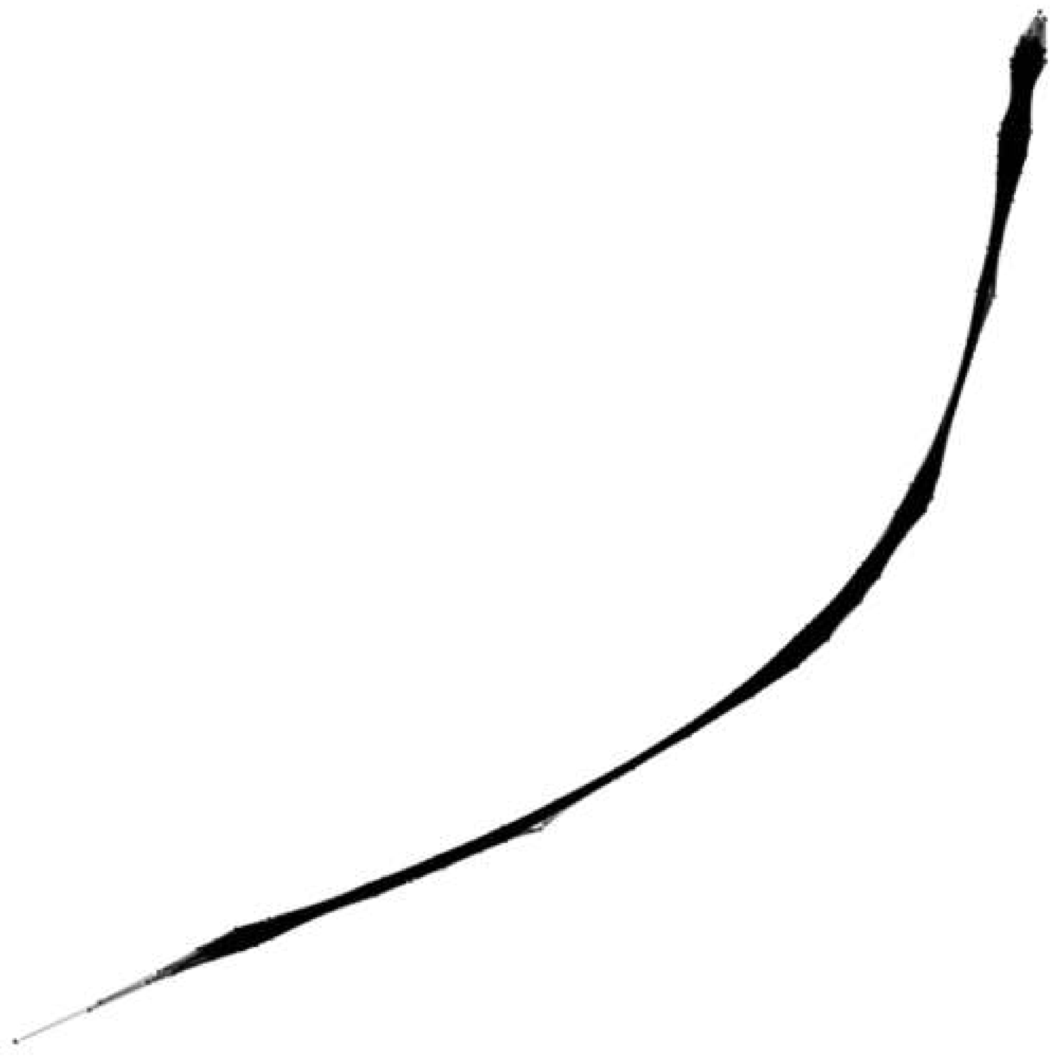

CL84
rRNA
Length of Reads (GP):533 (0.04%)

Tgrandiflorum

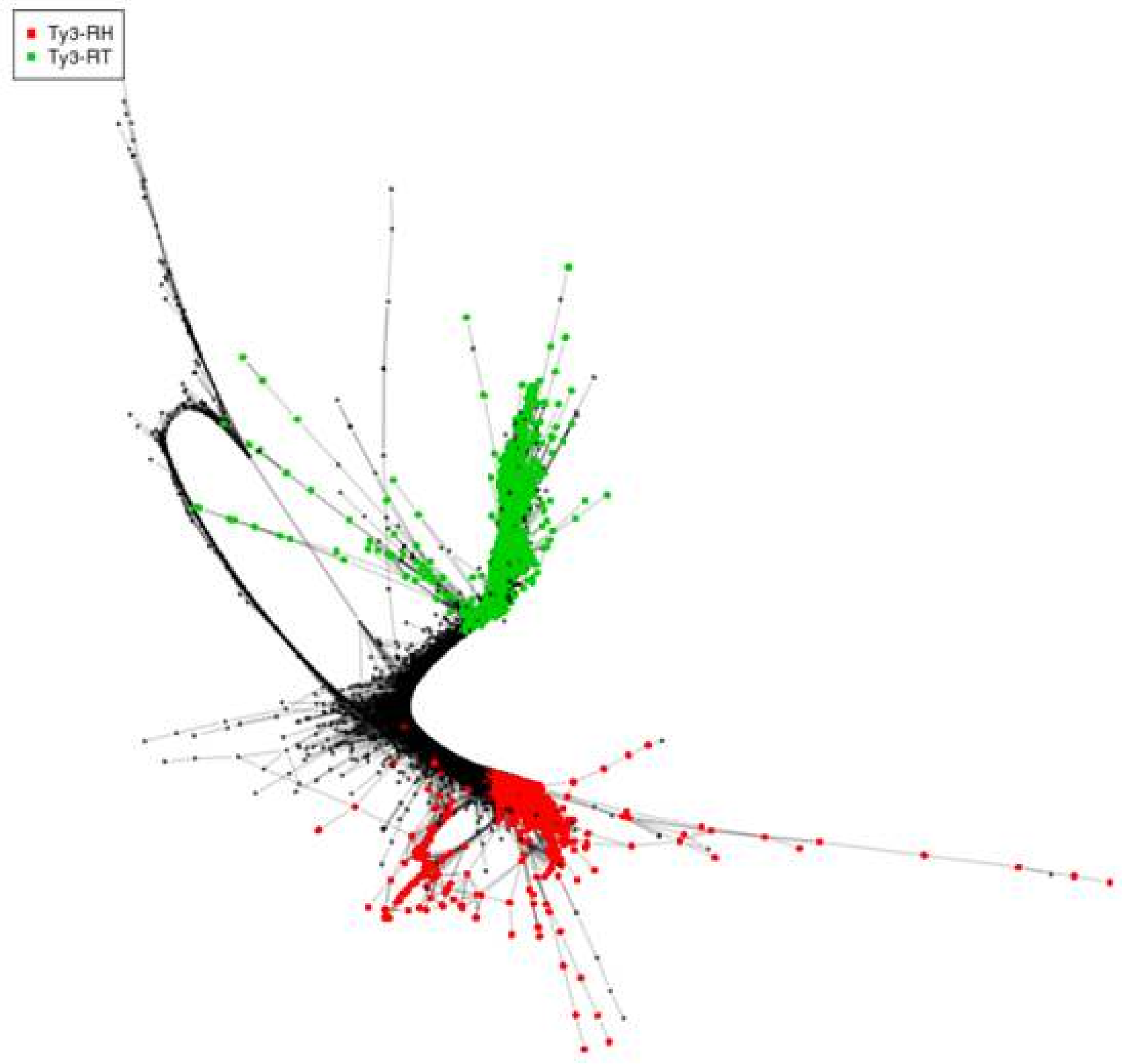

CL84
LTR_Gypsy
Length of Reads (GP):16890 (0.21%)

Tcacao

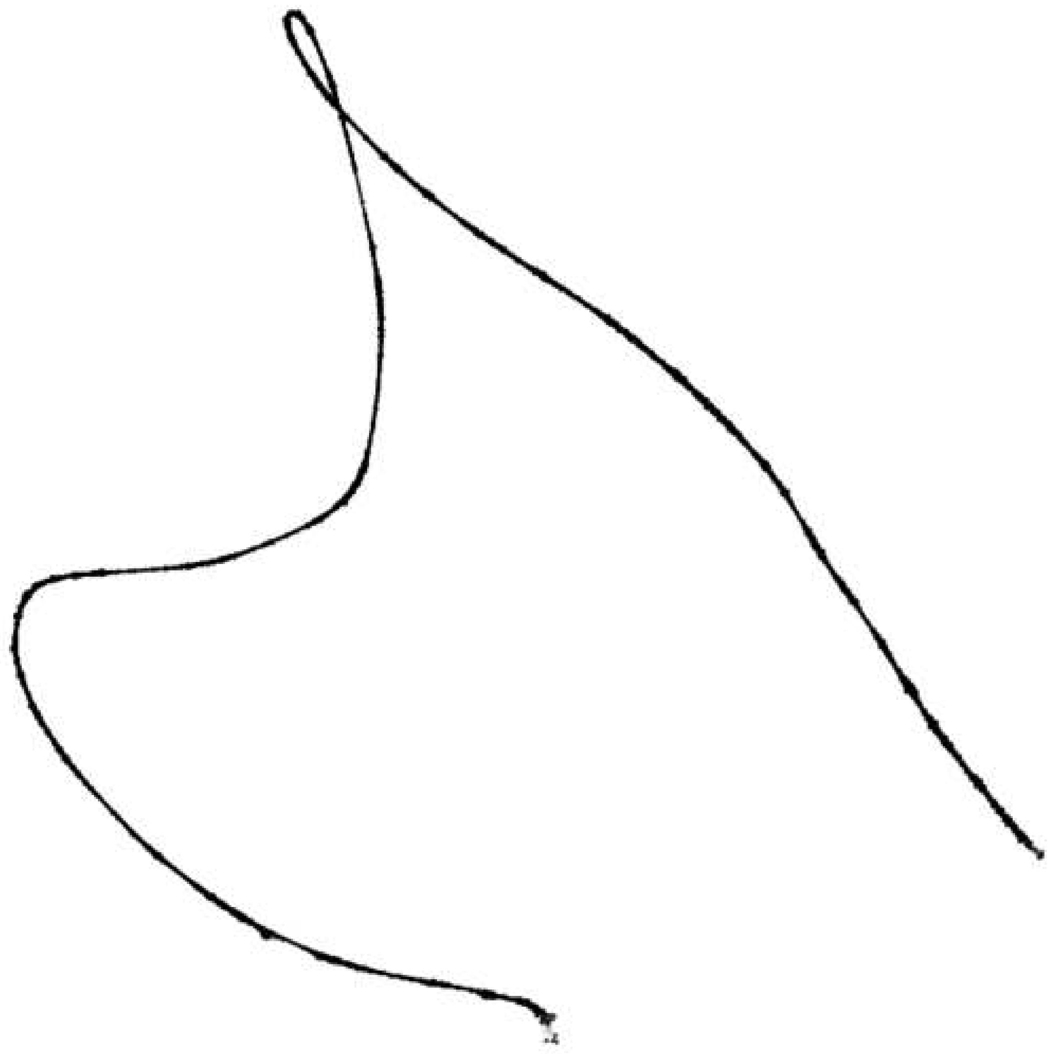

CL84
Low_complexity
Length of Reads (GP):1958 (0.1%)

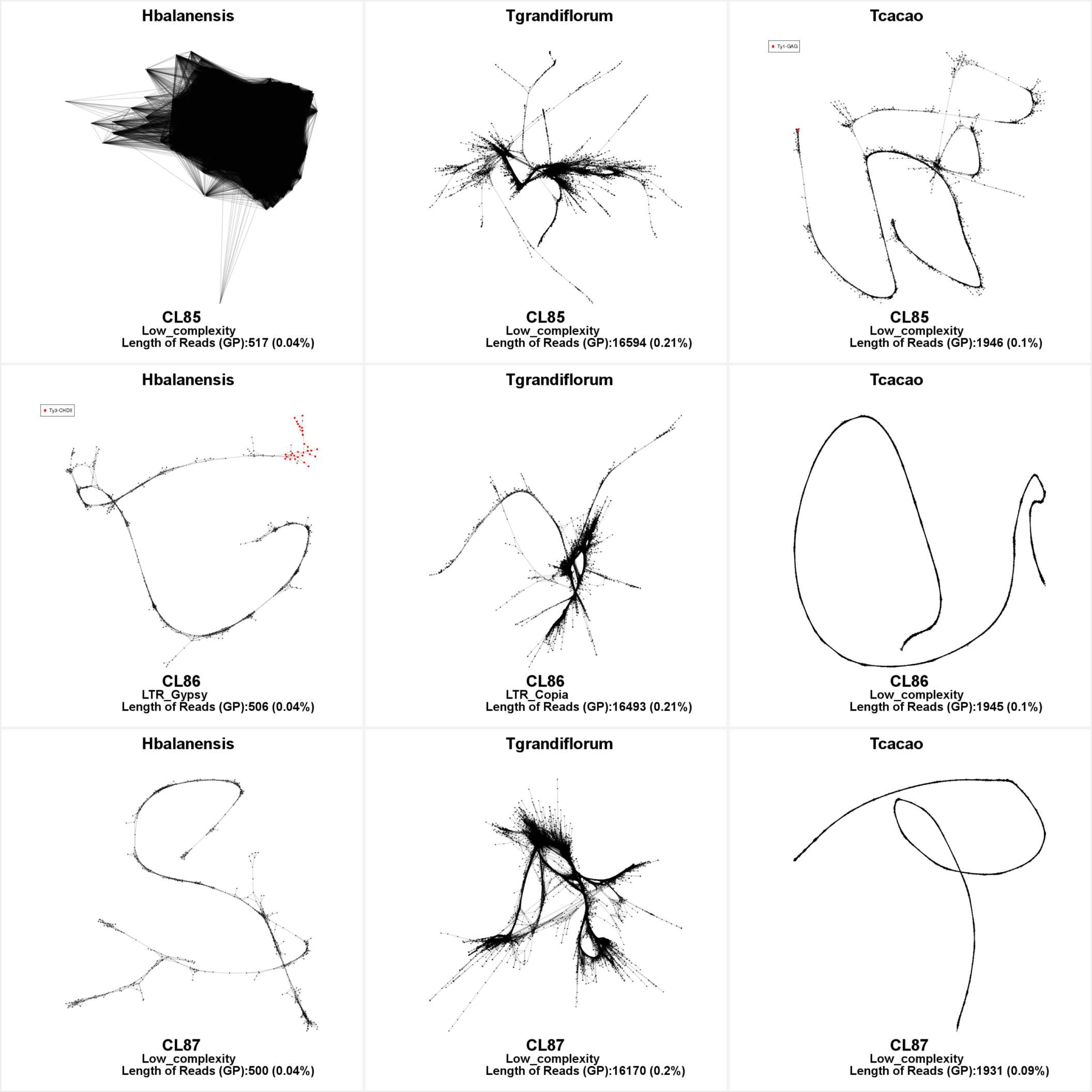
Hbalanensis
CL85
Low_complexity
Length of Reads (GP):517 (0.04%)
Tgrandiflorum
CL85
Low_complexity
Length of Reads (GP):16594 (0.21%)
Tcacao
Ty1-GAG
CL85
Low_complexity
Length of Reads (GP):1946 (0.1%)
Hbalanensis
Ty3-CHDII
CL86
LTR_Gypsy
Length of Reads (GP):506 (0.04%)
Tgrandiflorum
CL86
LTR_Copia
Length of Reads (GP):16493 (0.21%)
Tcacao
CL86
Low_complexity
Length of Reads (GP):1945 (0.1%)
Hbalanensis
CL87
Low_complexity
Length of Reads (GP):500 (0.04%)
Tgrandiflorum
CL87
Low_complexity
Length of Reads (GP):16170 (0.2%)
Tcacao
CL87
Low_complexity
Length of Reads (GP):1931 (0.09%)

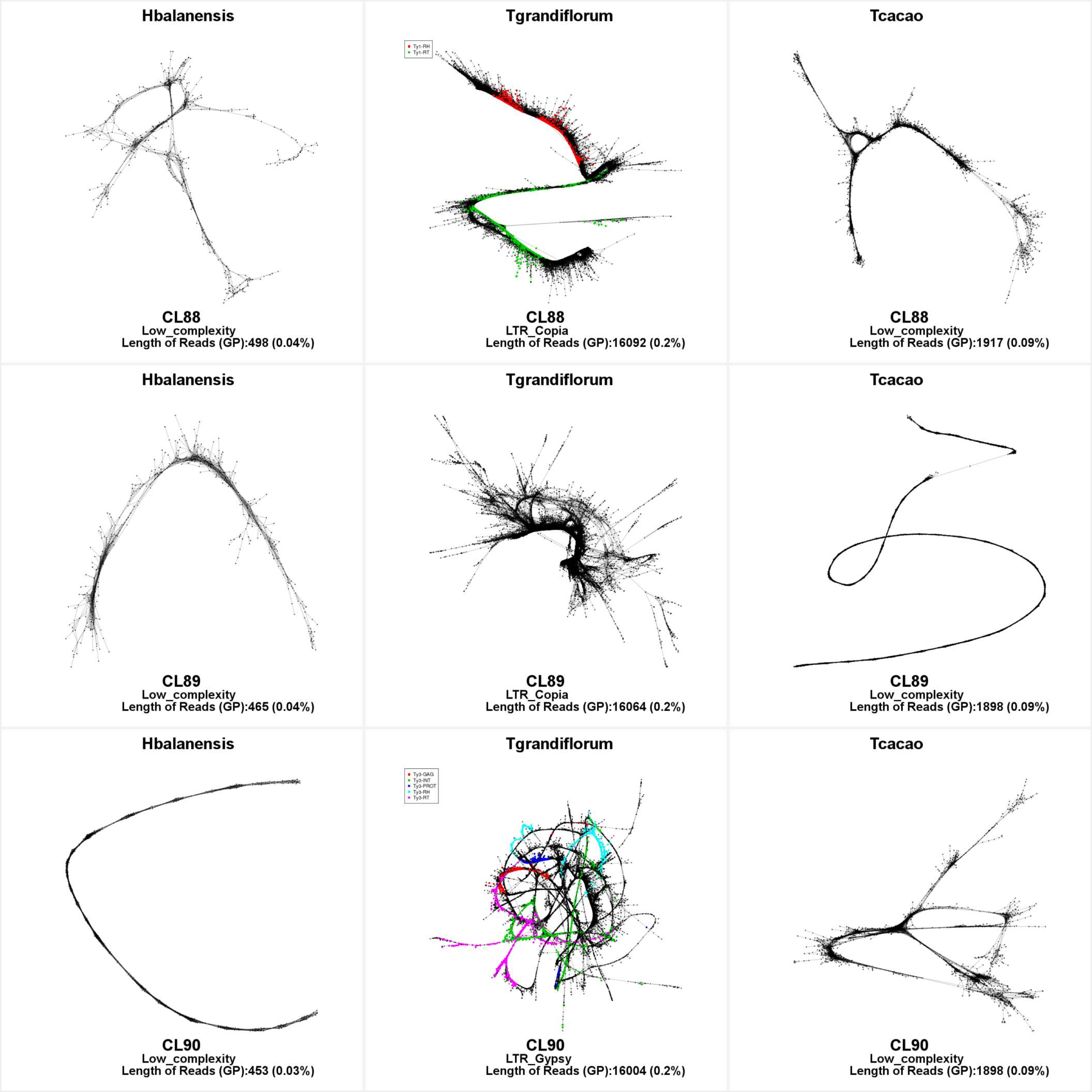

Hbalanensis
CL88
Low_complexity
Length of Reads (GP):498 (0.04%)
Tgrandiflorum
Ty1-RH
Ty1-RT
CL88
LTR_Copia
Length of Reads (GP):16092 (0.2%)
Tcacao
CL88
Low_complexity
Length of Reads (GP):1917 (0.09%)
Hbalanensis
CL89
Low_complexity
Length of Reads (GP):465 (0.04%)
Tgrandiflorum
CL89
LTR_Copia
Length of Reads (GP):16064 (0.2%)
Tcacao
CL89
Low_complexity
Length of Reads (GP):1898 (0.09%)
Hbalanensis
CL90
Low_complexity
Length of Reads (GP):453 (0.03%)
Tgrandiflorum
Ty3-GAG
Ty3-INT
Ty3-PROT
Ty3-RH
Ty3-RT
CL90
LTR_Gypsy
Length of Reads (GP):16004 (0.2%)
Tcacao
CL90
Low_complexity
Length of Reads (GP):1898 (0.09%)

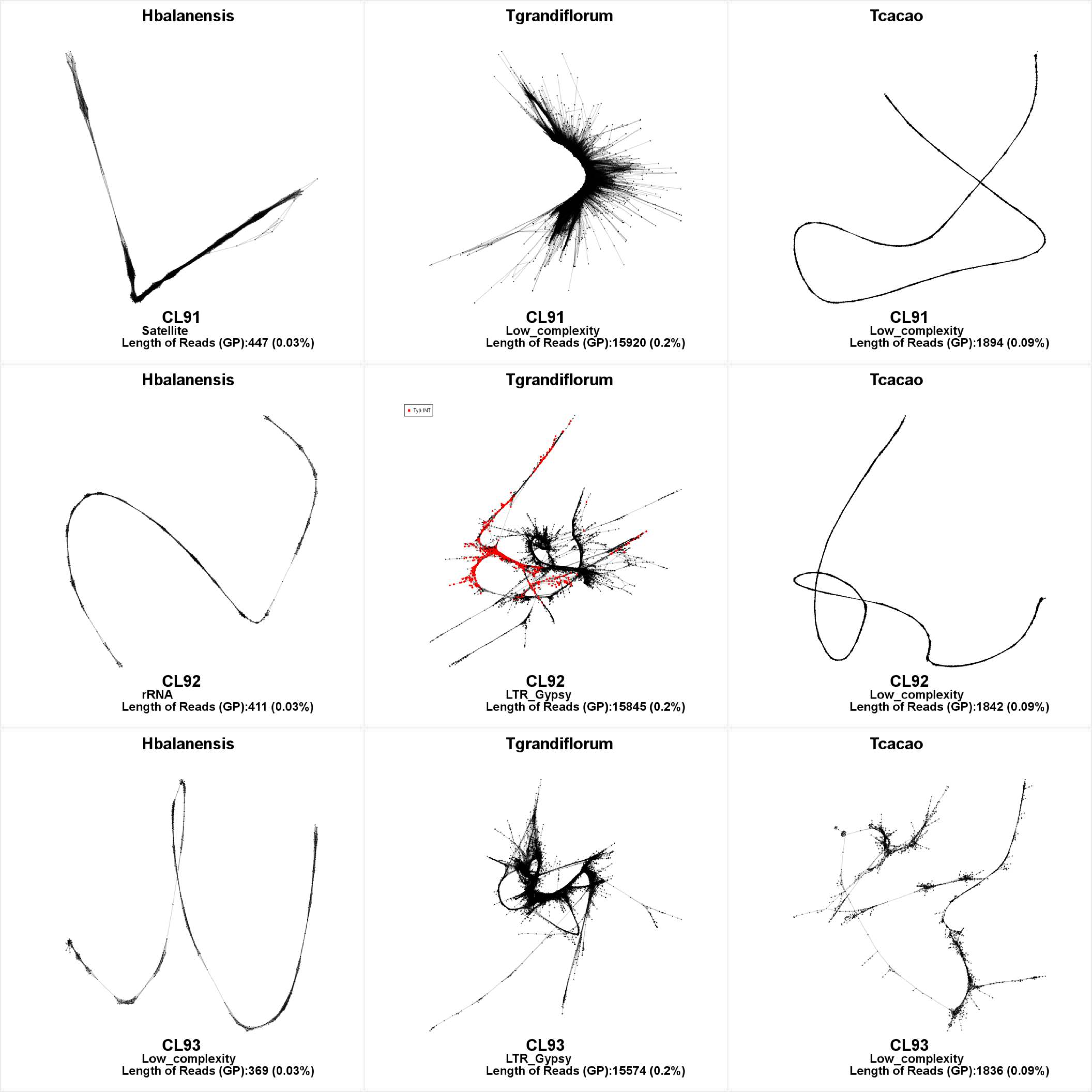
Hbalanensis
CL91
Satellite
Length of Reads (GP):447 (0.03%)
Tgrandiflorum
CL91
Low_complexity
Length of Reads (GP):15920 (0.2%)
Tcacao
CL91
Low_complexity
Length of Reads (GP):1894 (0.09%)
Hbalanensis
CL92
rRNA
Length of Reads (GP):411 (0.03%)
Tgrandiflorum
Ty3-INT
CL92
LTR_Gypsy
Length of Reads (GP):15845 (0.2%)
Tcacao
CL92
Low_complexity
Length of Reads (GP):1842 (0.09%)
Hbalanensis
CL93
Low_complexity
Length of Reads (GP):369 (0.03%)
Tgrandiflorum
CL93
LTR_Gypsy
Length of Reads (GP):15574 (0.2%)
Tcacao
CL93
Low_complexity
Length of Reads (GP):1836 (0.09%)

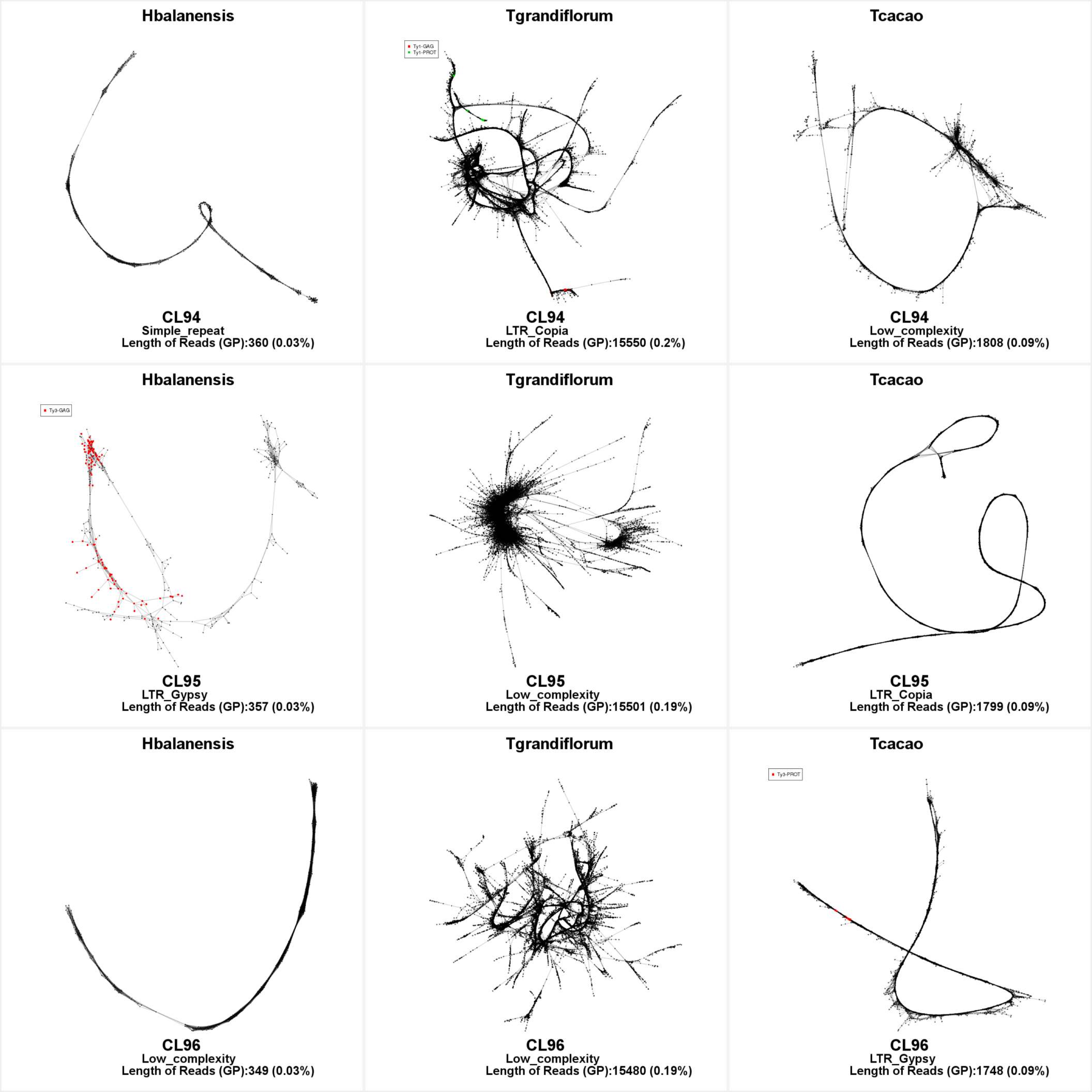
Hbalanensis
CL94
Simple_repeat
Length of Reads (GP):360 (0.03%)
Tgrandiflorum
Ty1-GAG
Ty1-PROT
CL94
LTR_Copia
Length of Reads (GP):15550 (0.2%)
Tcacao
CL94
Low_complexity
Length of Reads (GP):1808 (0.09%)
Hbalanensis
Ty3-GAG
CL95
LTR_Gypsy
Length of Reads (GP):357 (0.03%)
Tgrandiflorum
CL95
Low_complexity
Length of Reads (GP):15501 (0.19%)
Tcacao
CL95
LTR_Copia
Length of Reads (GP):1799 (0.09%)
Hbalanensis
CL96
Low_complexity
Length of Reads (GP):349 (0.03%)
Tgrandiflorum
CL96
Low_complexity
Length of Reads (GP):15480 (0.19%)
Tcacao
Ty3-PROT
CL96
LTR_Gypsy
Length of Reads (GP):1748 (0.09%)

Hbalanensis

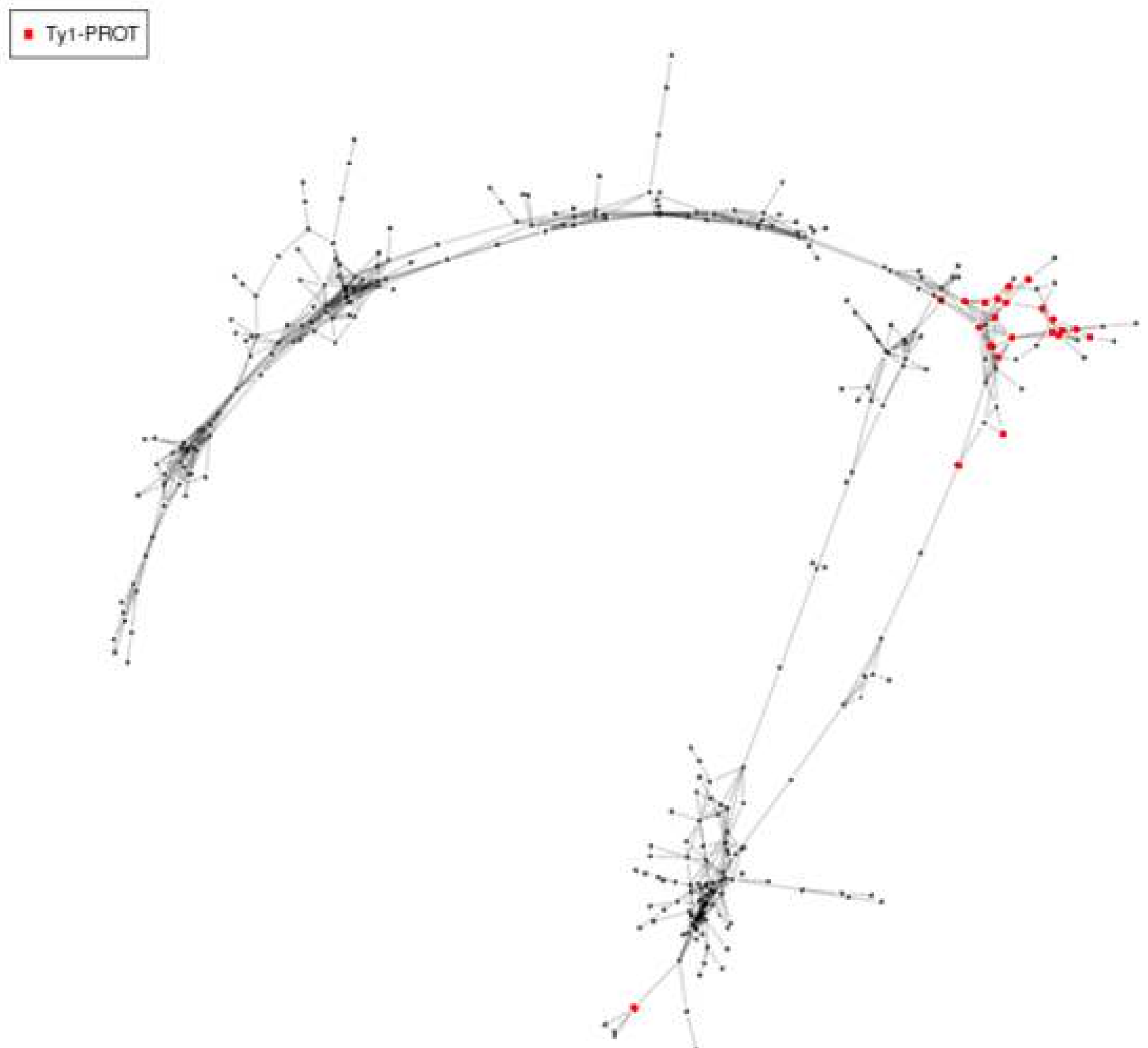

CL97
LTR_Copia
Length of Reads (GP):338 (0.03%)

Tgrandiflorum

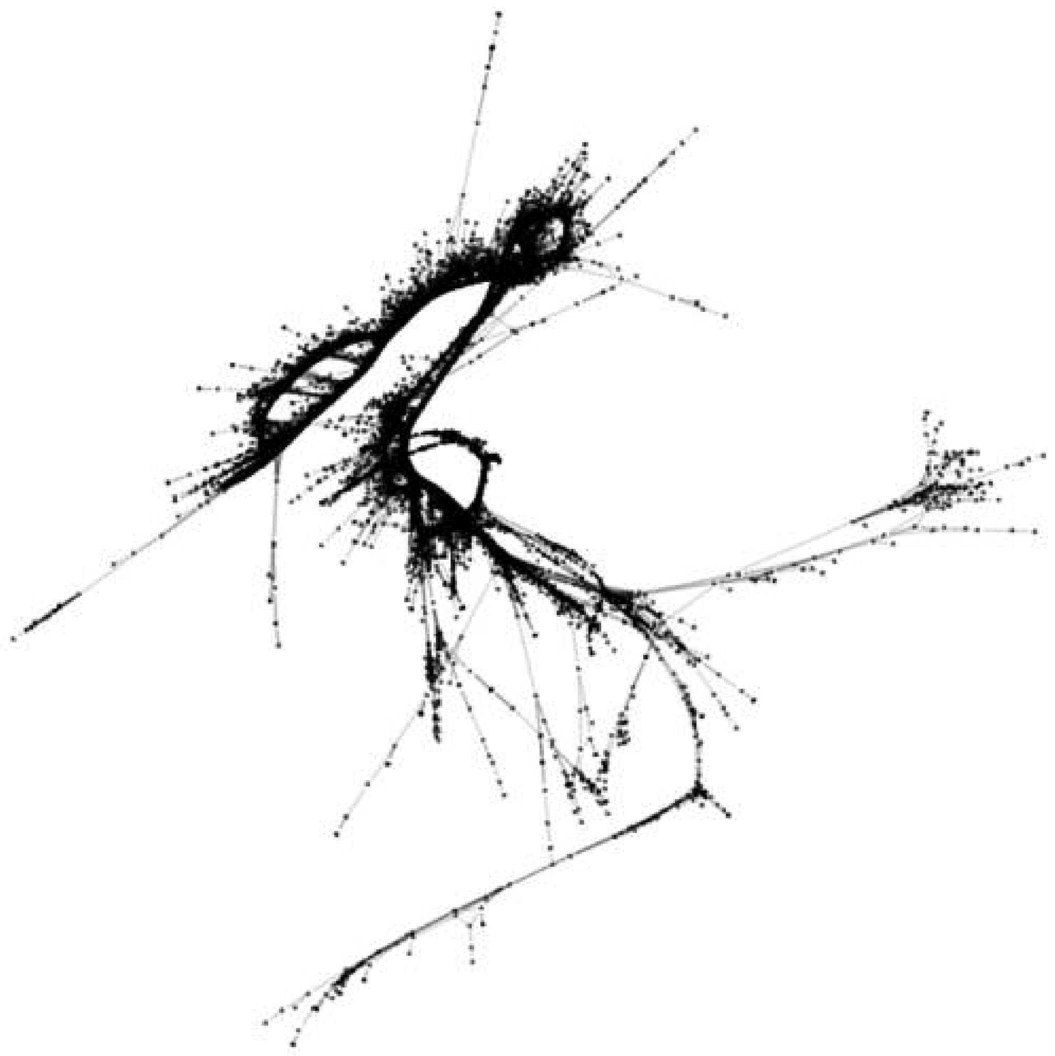

CL97
Low_complexity
Length of Reads (GP):15424 (0.19%)

Tcacao

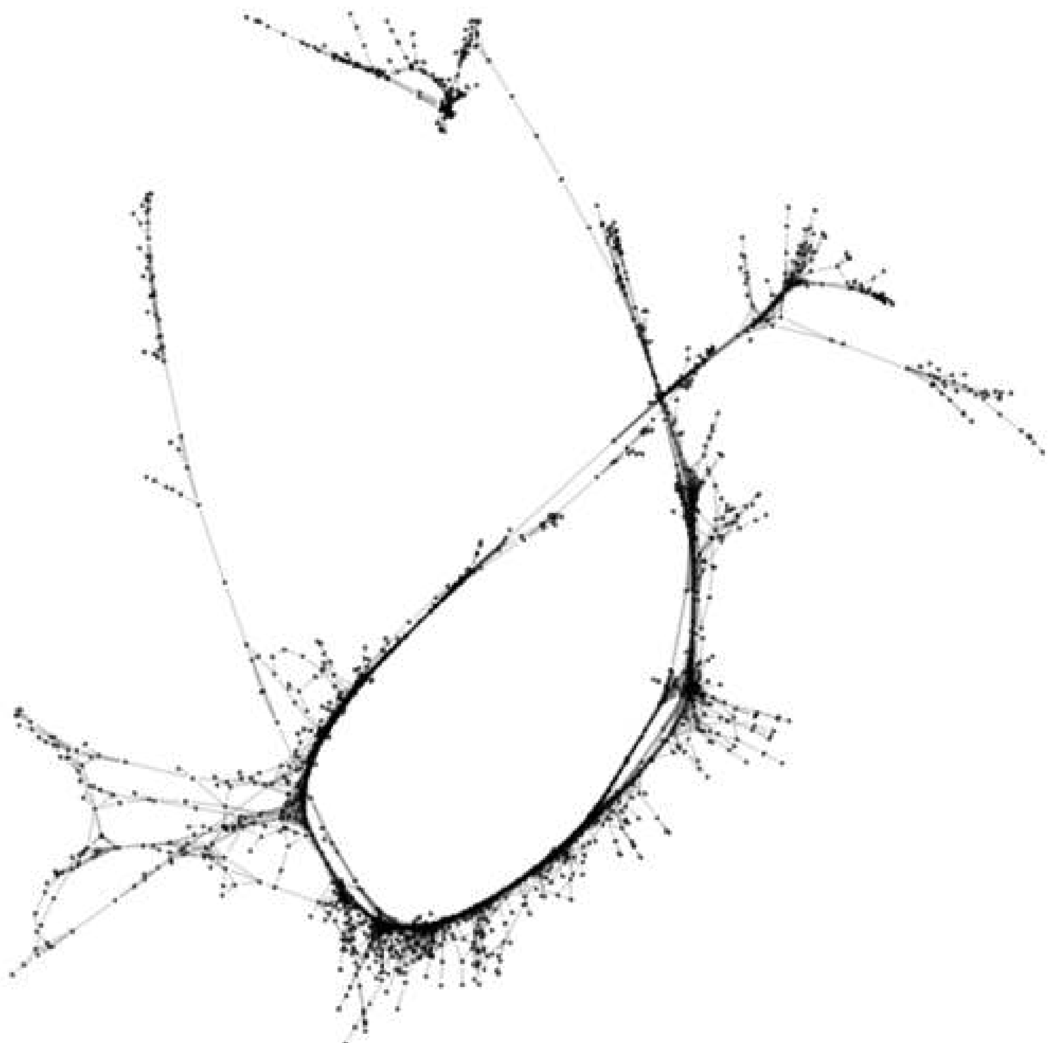

CL97
Low_complexity
Length of Reads (GP):1745 (0.09%)

Hbalanensis

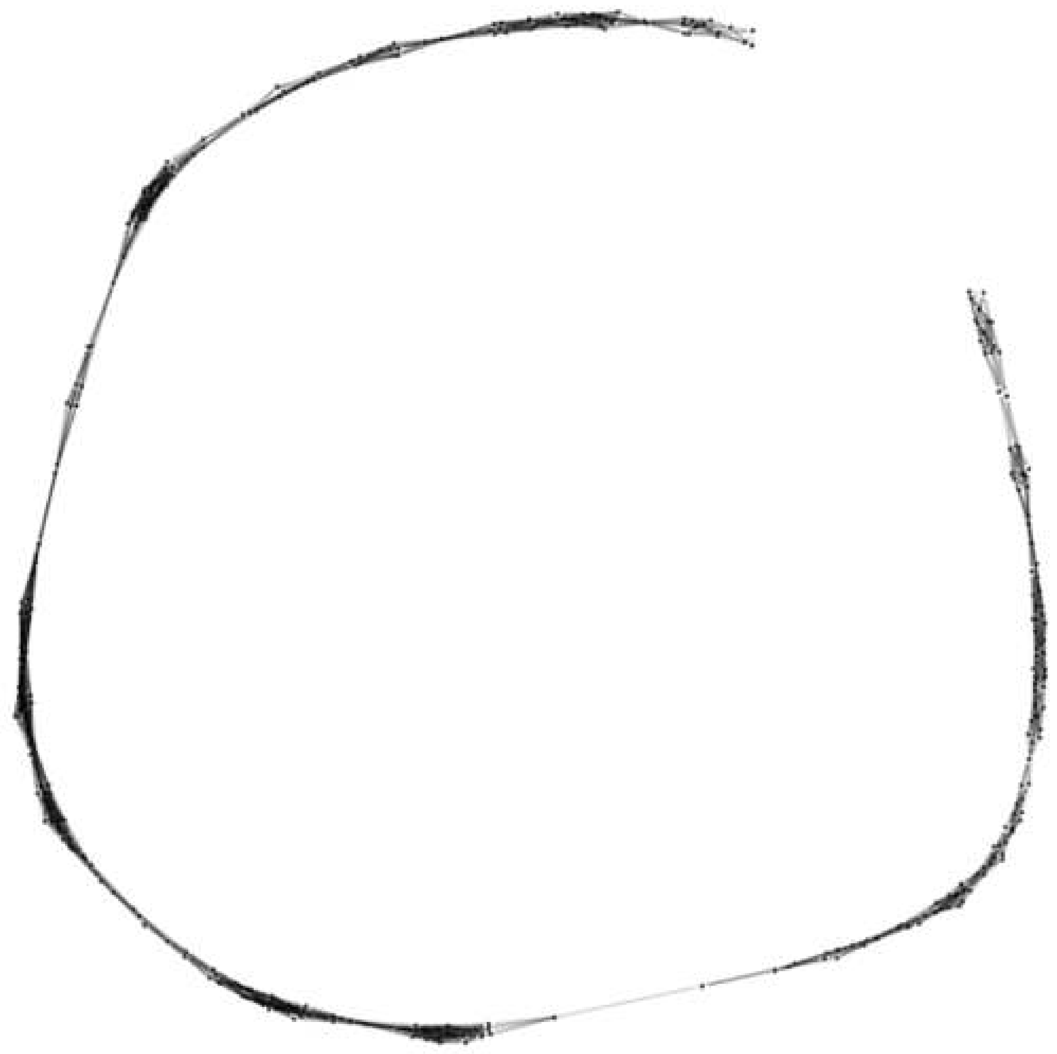

CL98
Low_complexity
Length of Reads (GP):336 (0.03%)

Tgrandiflorum

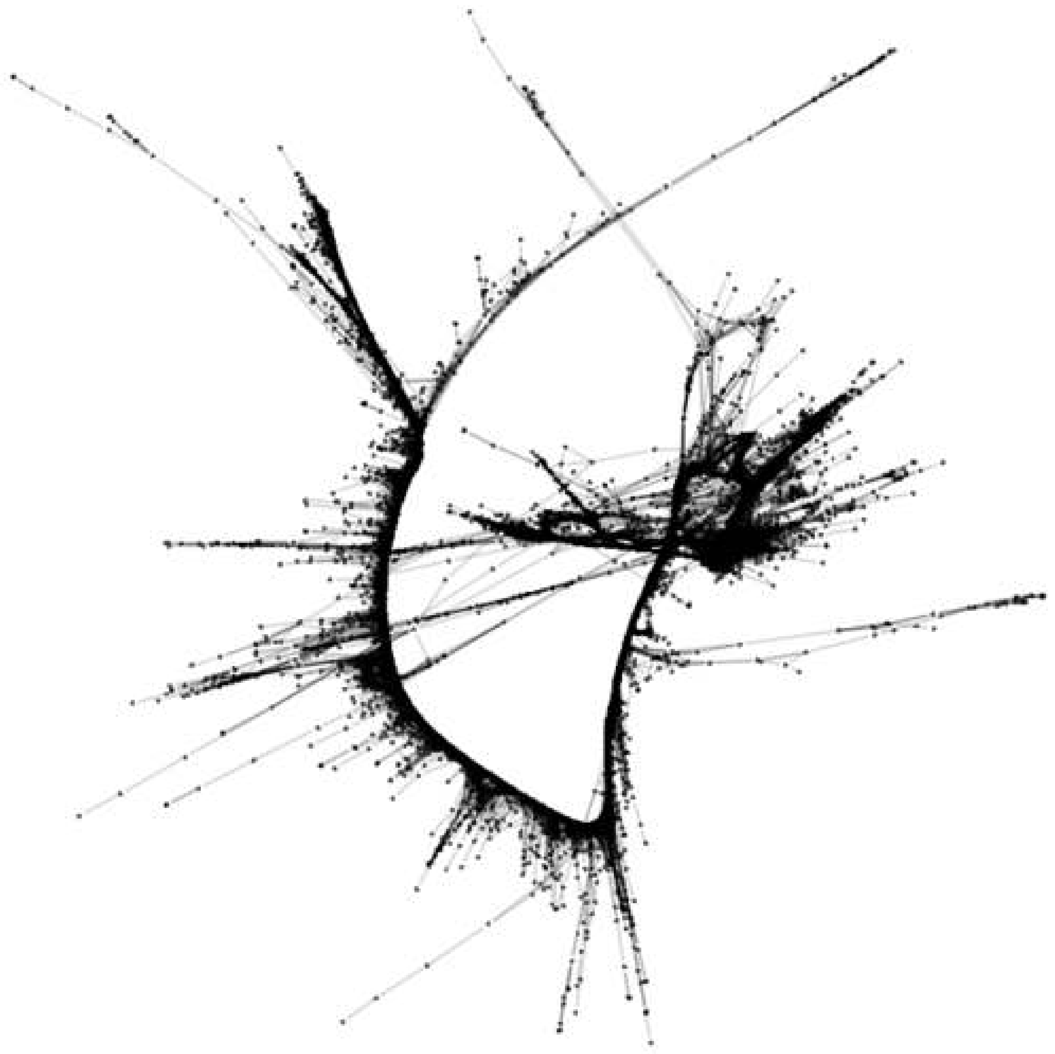

CL98
Low_complexity
Length of Reads (GP):15406 (0.19%)

Tcacao

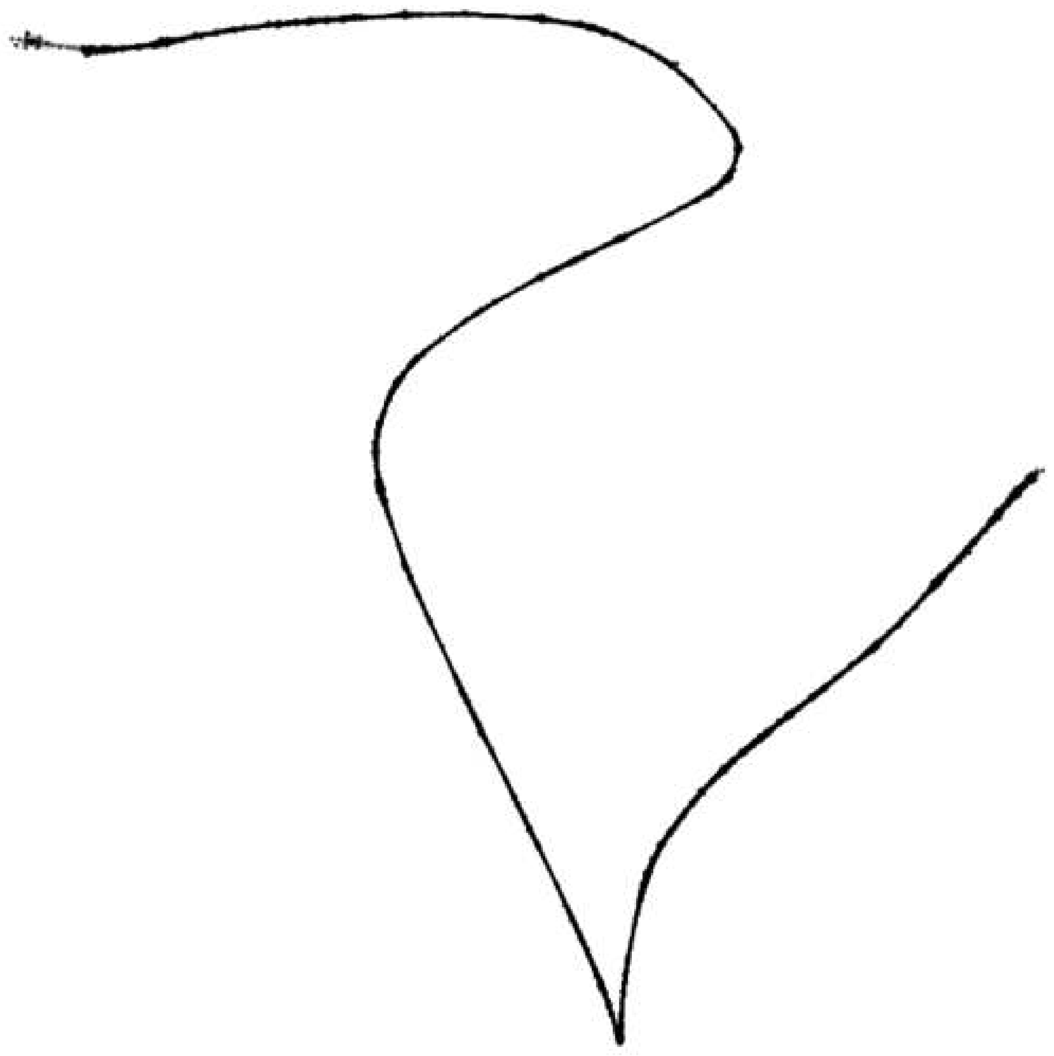

CL98
Low_complexity
Length of Reads (GP):1718 (0.08%)

Hbalanensis

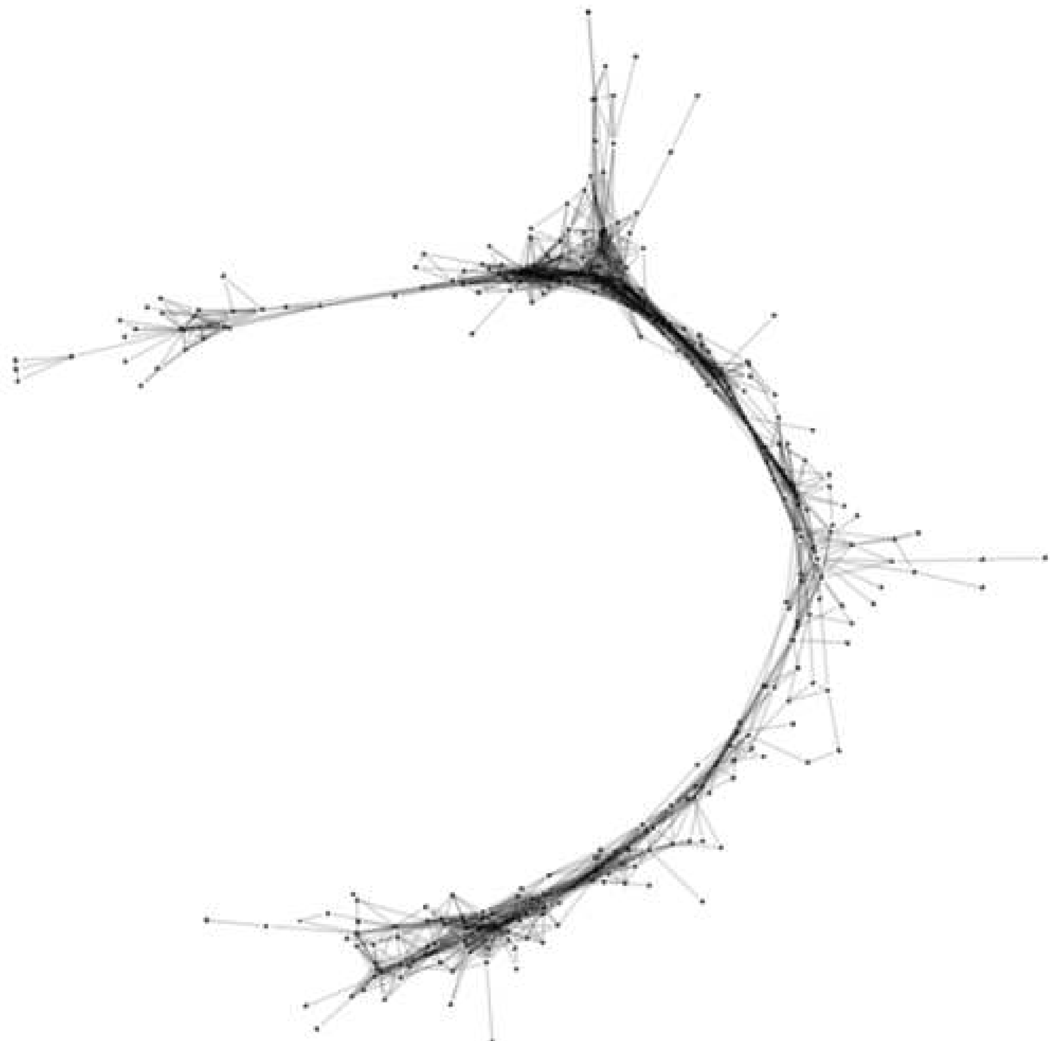

CL99
Low_complexity
Length of Reads (GP):334 (0.03%)

Tgrandiflorum

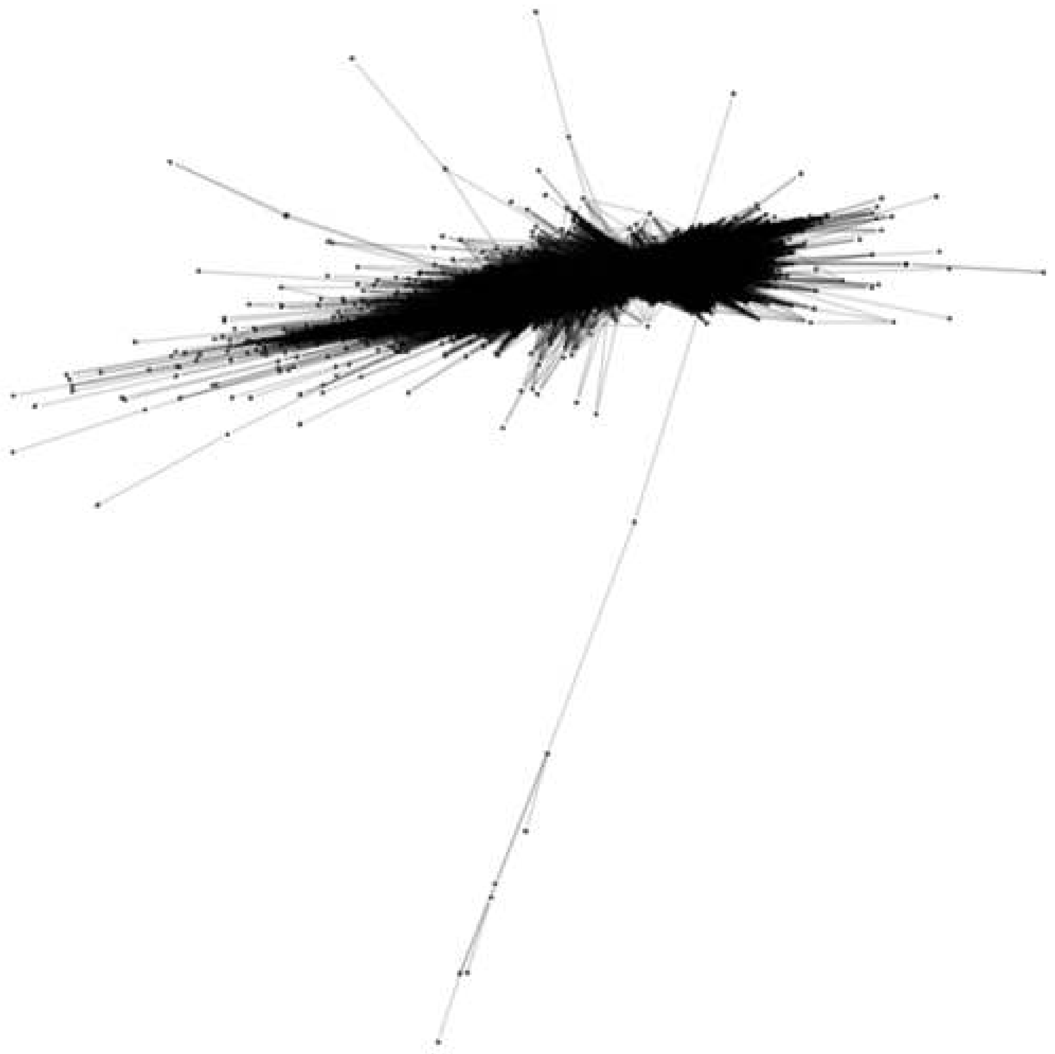

CL99
LTR_Copia
Length of Reads (GP):15217 (0.19%)

Tcacao

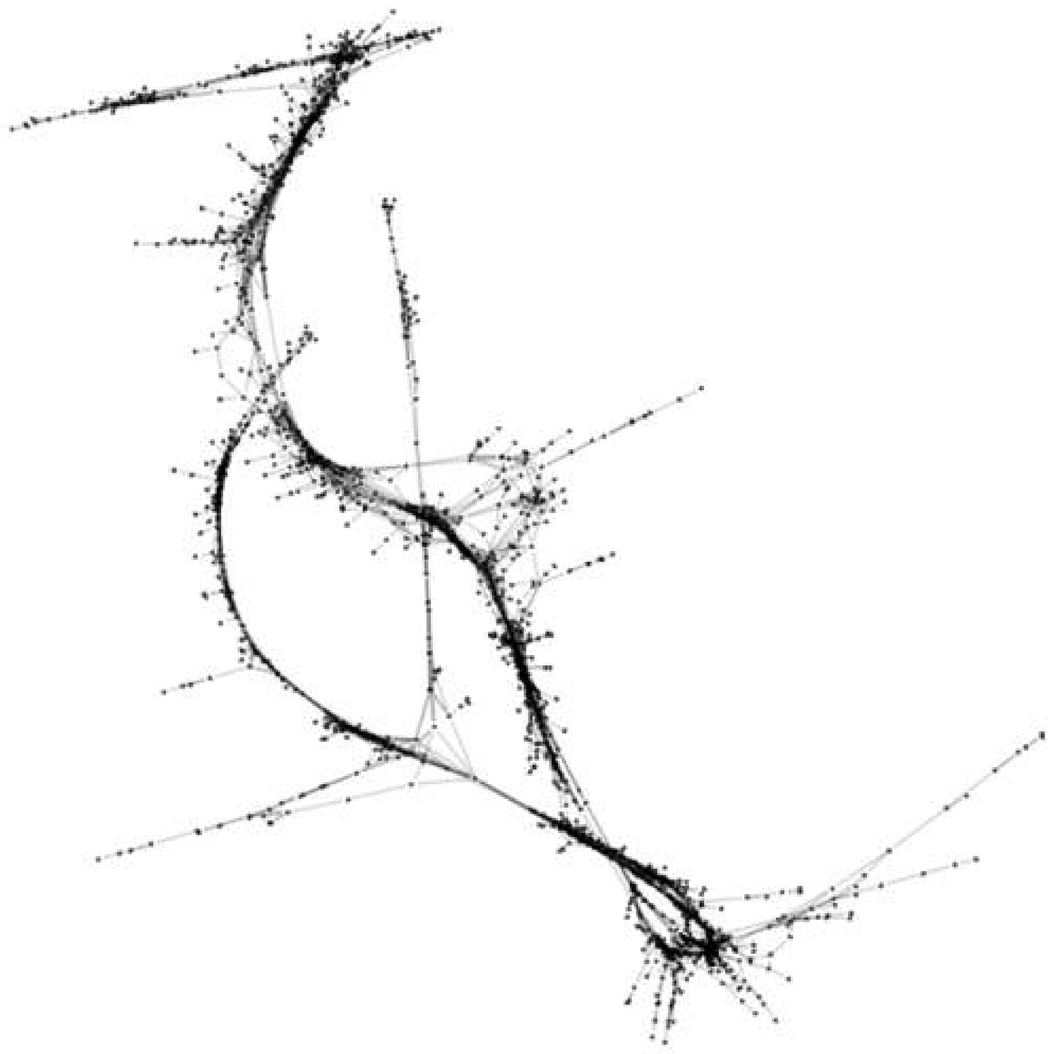

CL99
LTR_Copia
Length of Reads (GP):1659 (0.08%)

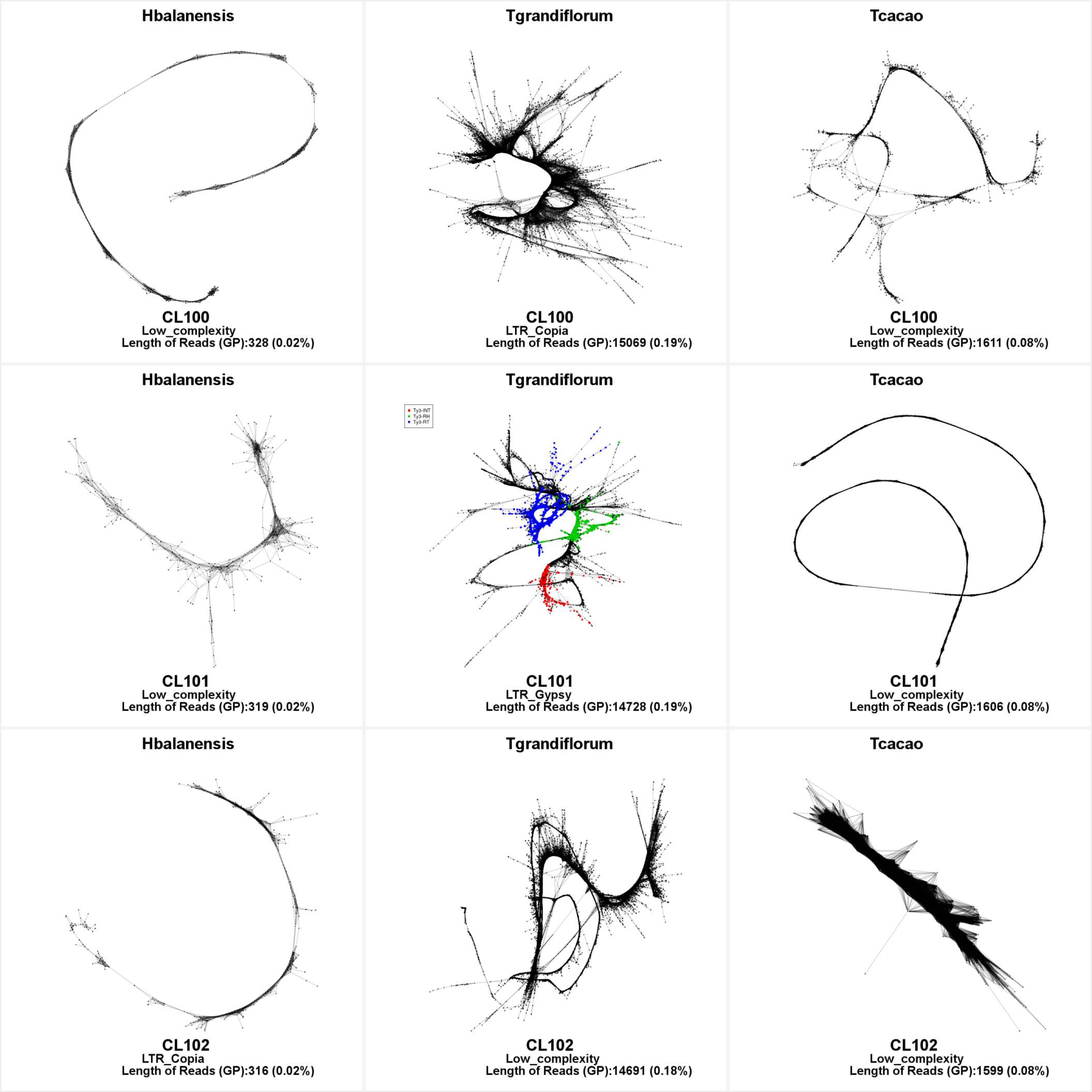
Hbalanensis
CL100
Low_complexity
Length of Reads (GP):328 (0.02%)
Tgrandiflorum
CL100
LTR_Copia
Length of Reads (GP):15069 (0.19%)
Tcacao
CL100
Low_complexity
Length of Reads (GP):1611 (0.08%)
Hbalanensis
CL101
Low_complexity
Length of Reads (GP):319 (0.02%)
Tgrandiflorum
Ty3-INT
Ty3-RH
Ty3-RT
CL101
LTR_Gypsy
Length of Reads (GP):14728 (0.19%)
Tcacao
CL101
Low_complexity
Length of Reads (GP):1606 (0.08%)
Hbalanensis
CL102
LTR_Copia
Length of Reads (GP):316 (0.02%)
Tgrandiflorum
CL102
Low_complexity
Length of Reads (GP):14691 (0.18%)
Tcacao
CL102
Low_complexity
Length of Reads (GP):1599 (0.08%)

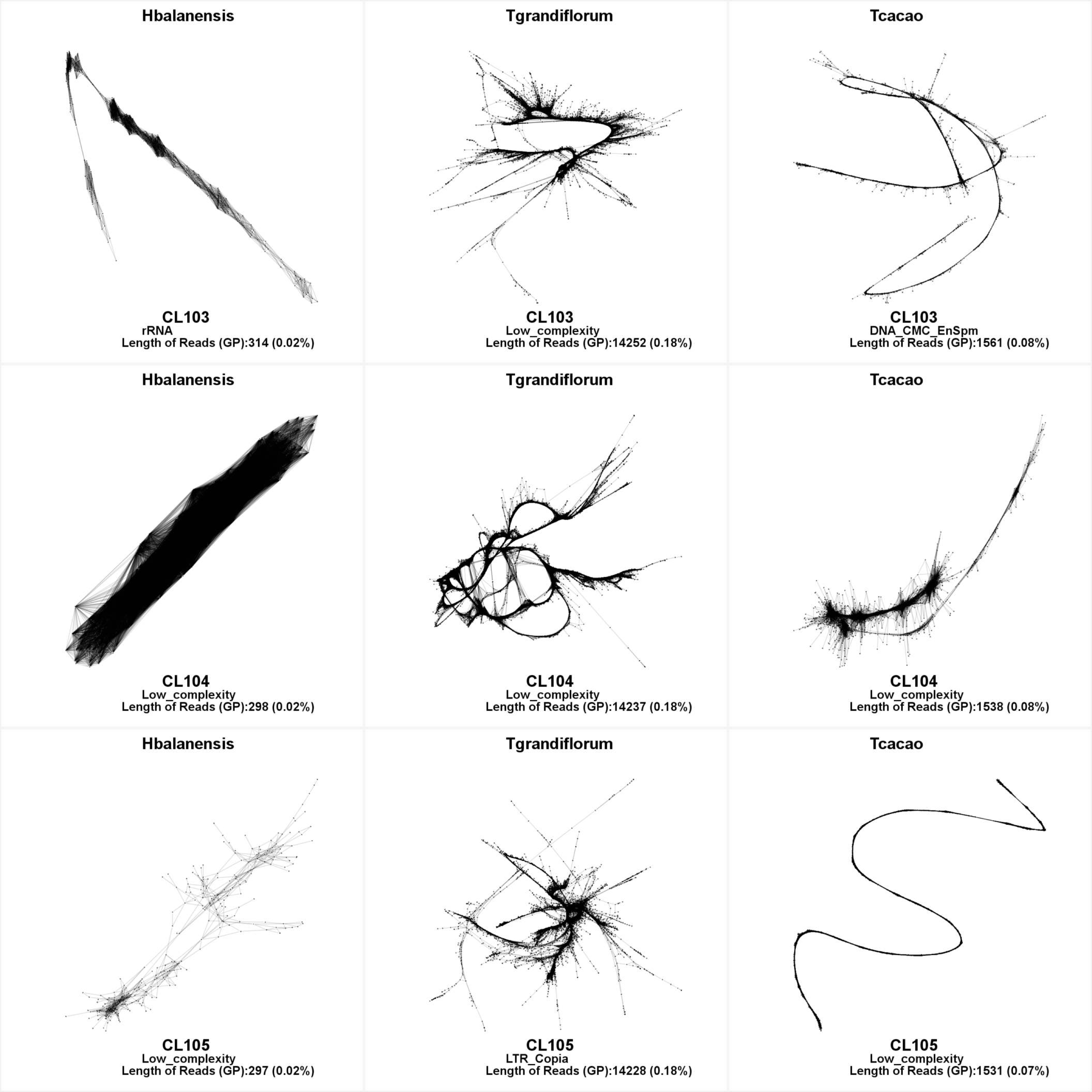
Hbalanensis
CL103
rRNA
Length of Reads (GP):314 (0.02%)
Tgrandiflorum
CL103
Low_complexity
Length of Reads (GP):14252 (0.18%)
Tcacao
CL103
DNA_CMC_EnSpm
Length of Reads (GP):1561 (0.08%)
Hbalanensis
CL104
Low_complexity
Length of Reads (GP):298 (0.02%)
Tgrandiflorum
CL104
Low_complexity
Length of Reads (GP):14237 (0.18%)
Tcacao
CL104
Low_complexity
Length of Reads (GP):1538 (0.08%)
Hbalanensis
CL105
Low_complexity
Length of Reads (GP):297 (0.02%)
Tgrandiflorum
CL105
LTR_Copia
Length of Reads (GP):14228 (0.18%)
Tcacao
CL105
Low_complexity
Length of Reads (GP):1531 (0.07%)

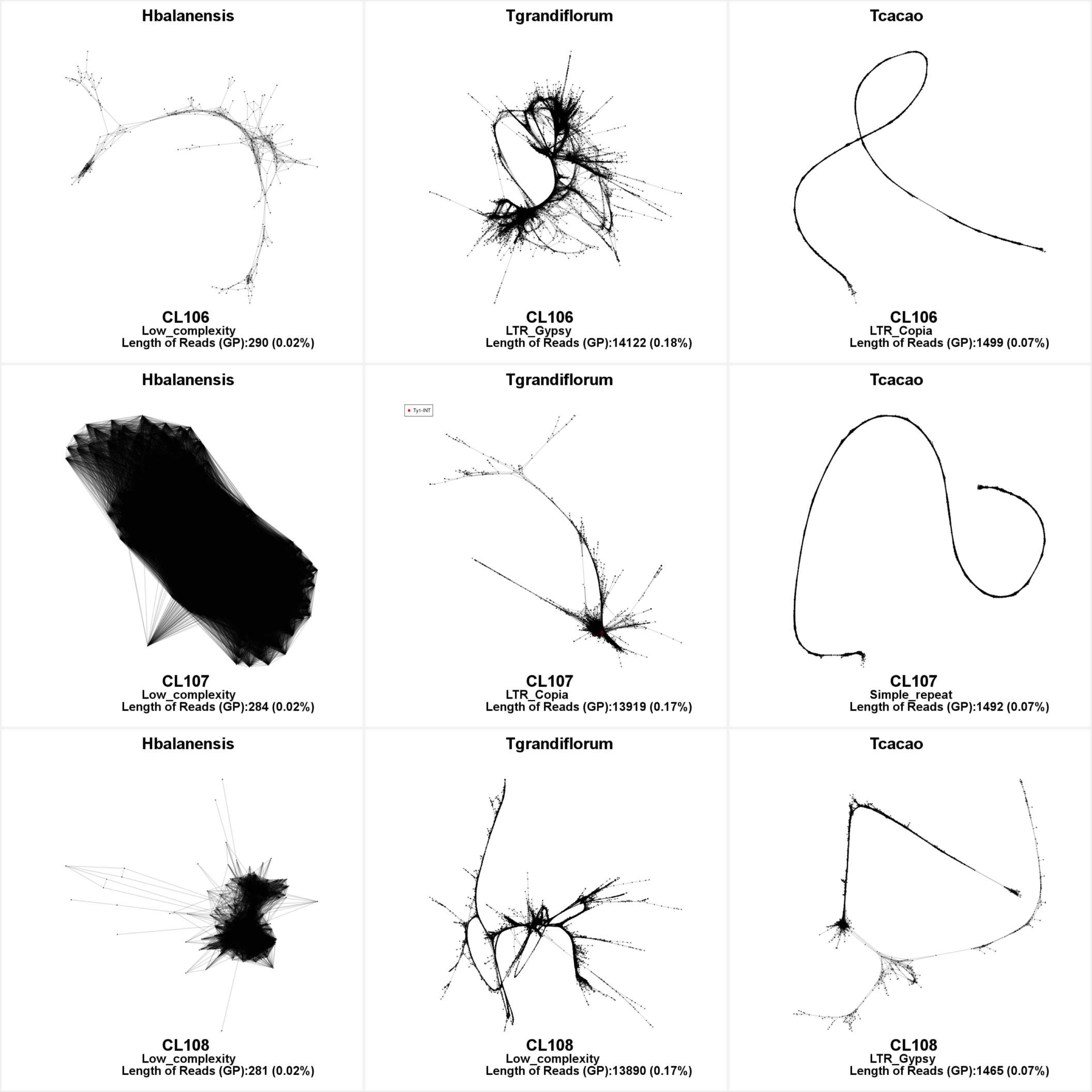
Hbalanensis
CL106
Low_complexity
Length of Reads (GP):290 (0.02%)
Tgrandiflorum
CL106
LTR_Gypsy
Length of Reads (GP):14122 (0.18%)
Tcacao
CL106
LTR_Copia
Length of Reads (GP):1499 (0.07%)
Hbalanensis
CL107
Low_complexity
Length of Reads (GP):284 (0.02%)
Tgrandiflorum
Ty1-INT
CL107
LTR_Copia
Length of Reads (GP):13919 (0.17%)
Tcacao
CL107
Simple_repeat
Length of Reads (GP):1492 (0.07%)
Hbalanensis
CL108
Low_complexity
Length of Reads (GP):281 (0.02%)
Tgrandiflorum
CL108
Low_complexity
Length of Reads (GP):13890 (0.17%)
Tcacao
CL108
LTR_Gypsy
Length of Reads (GP):1465 (0.07%)

Hbalanensis

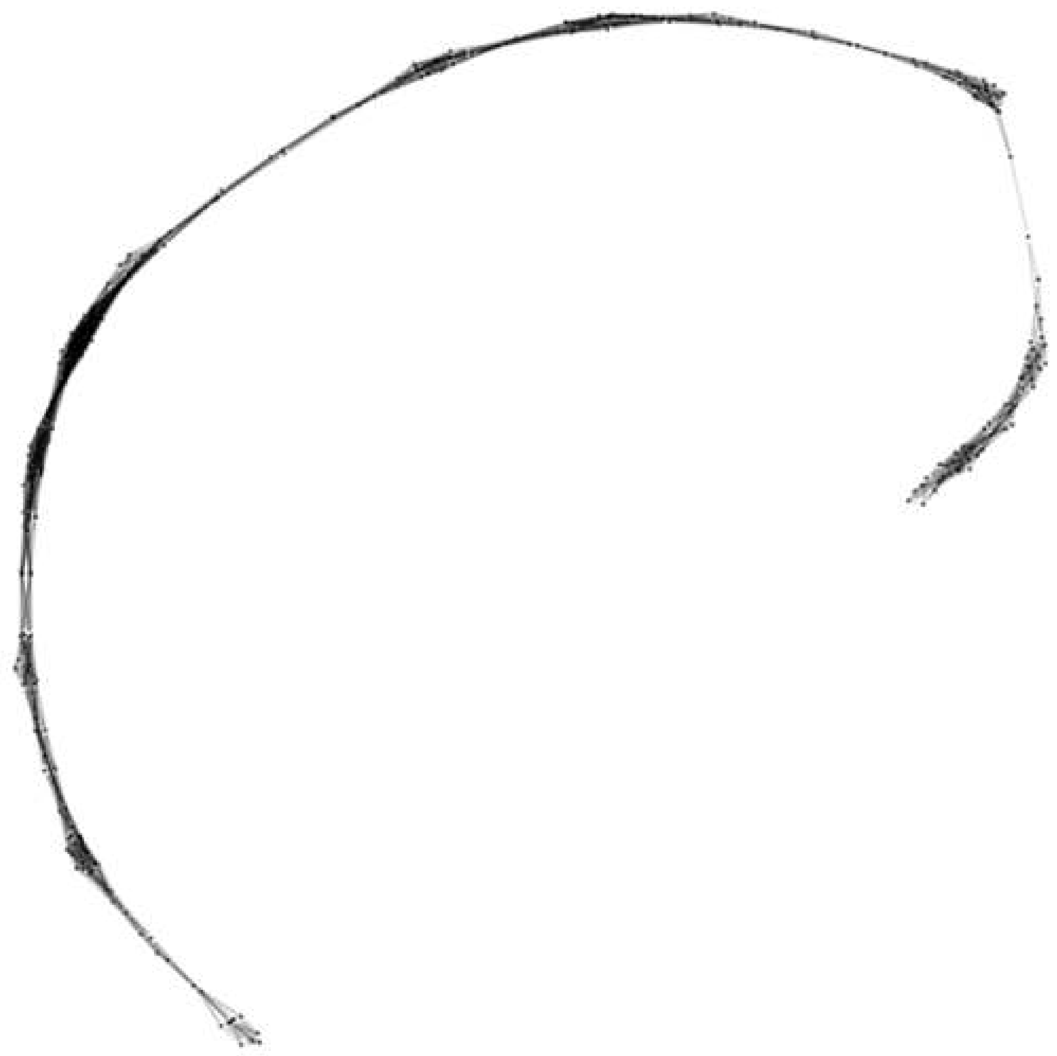

CL109
Low_complexity
Length of Reads (GP):280 (0.02%)

Tgrandiflorum

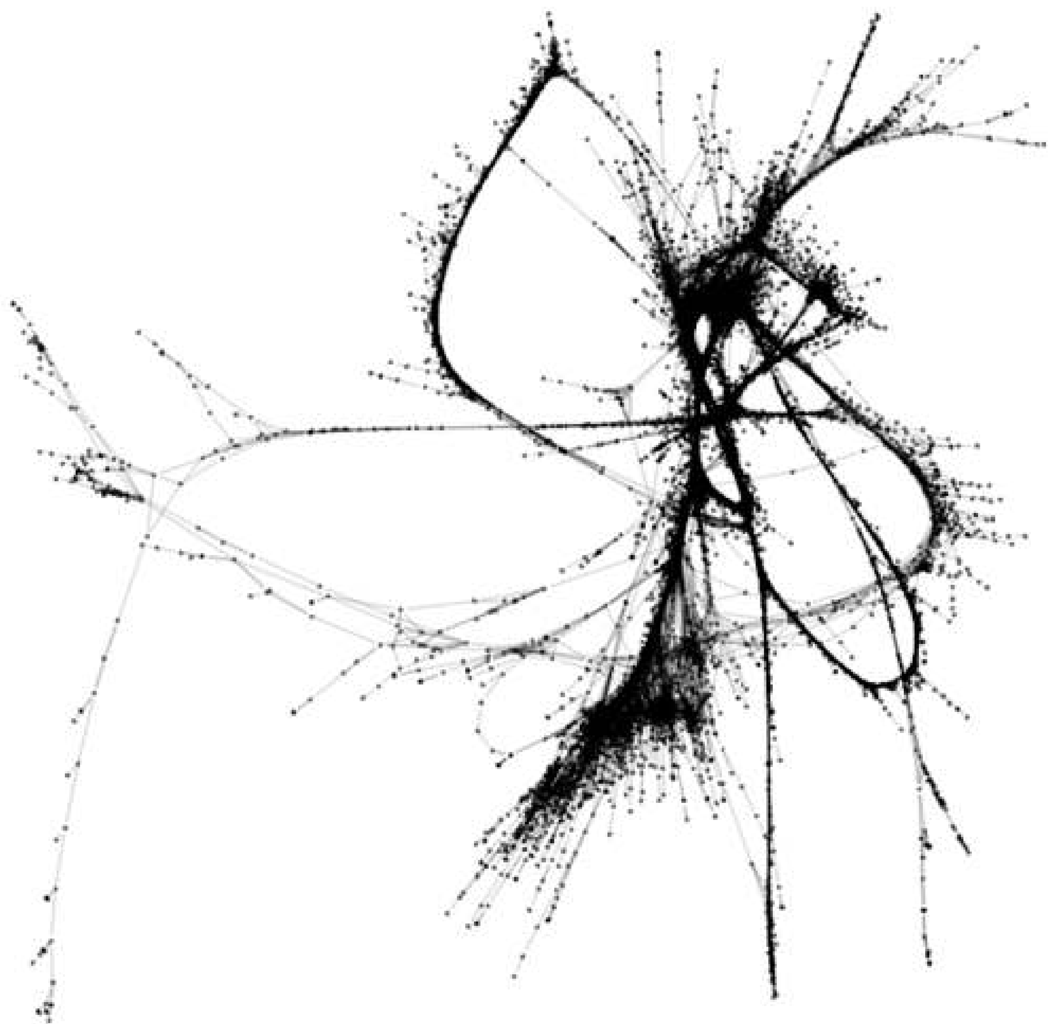

CL109
Low_complexity
Length of Reads (GP):13833 (0.17%)

Tcacao

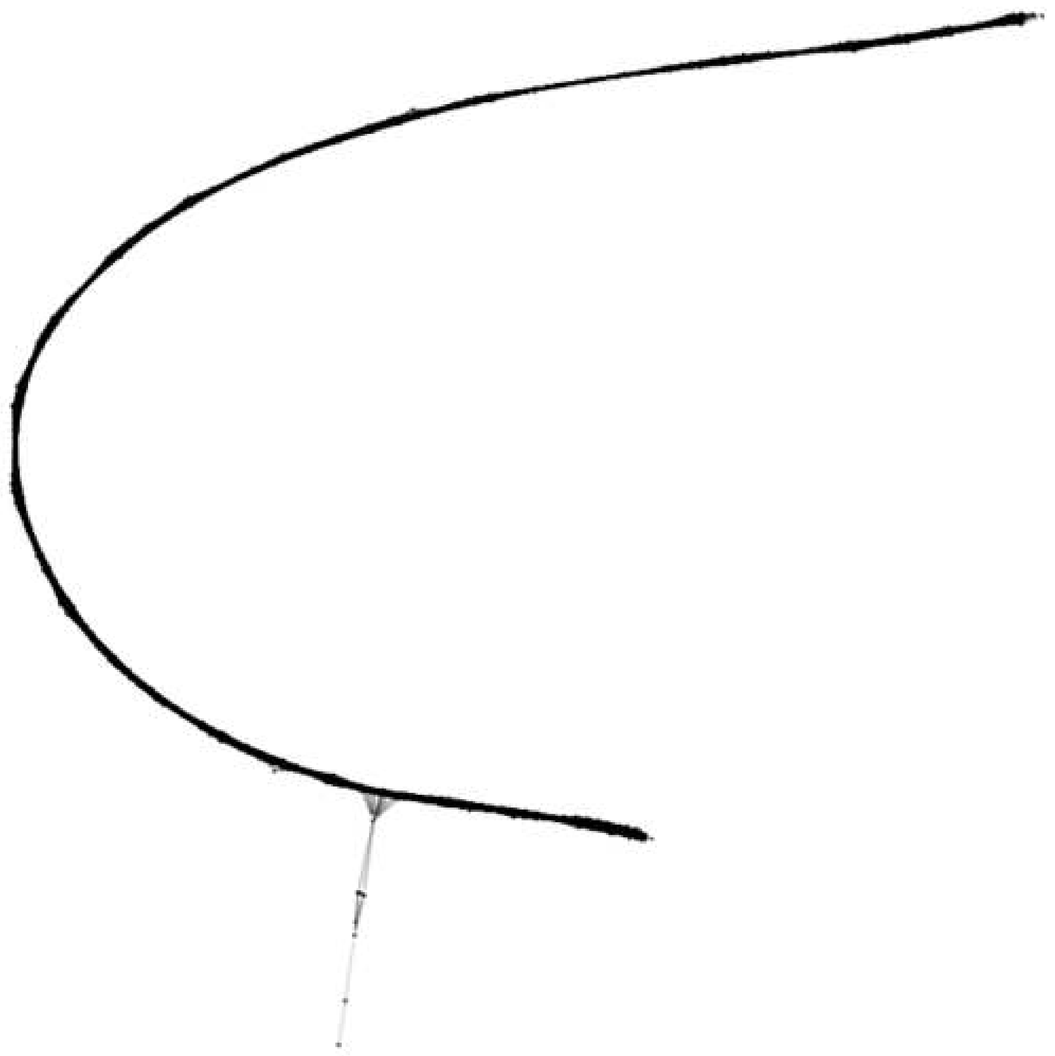

CL109
LTR_Gypsy
Length of Reads (GP):1451 (0.07%)

Hbalanensis

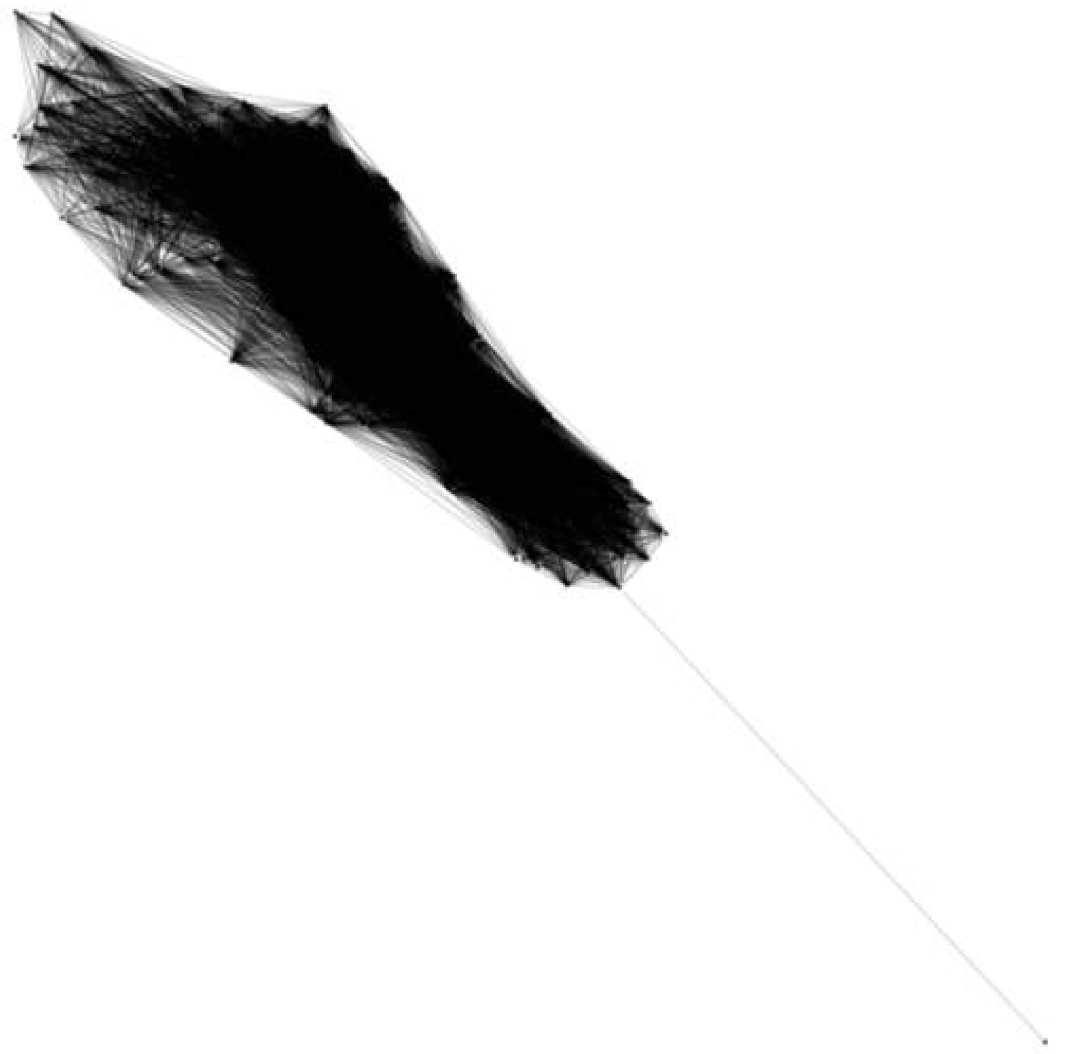

CL110
Low_complexity
Length of Reads (GP):279 (0.02%)

Tgrandiflorum

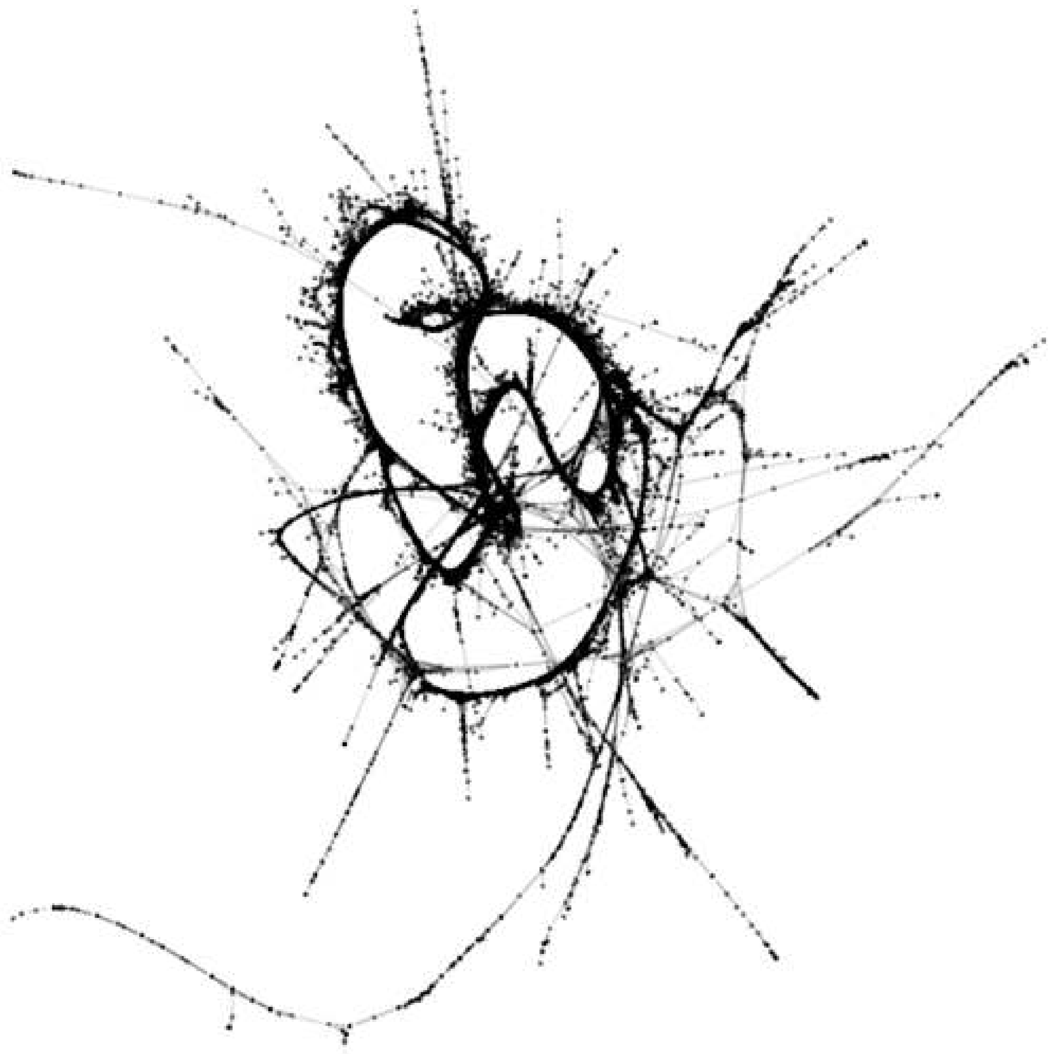

CL110
DNA_CMC_EnSpm
Length of Reads (GP):13783 (0.17%)

Tcacao

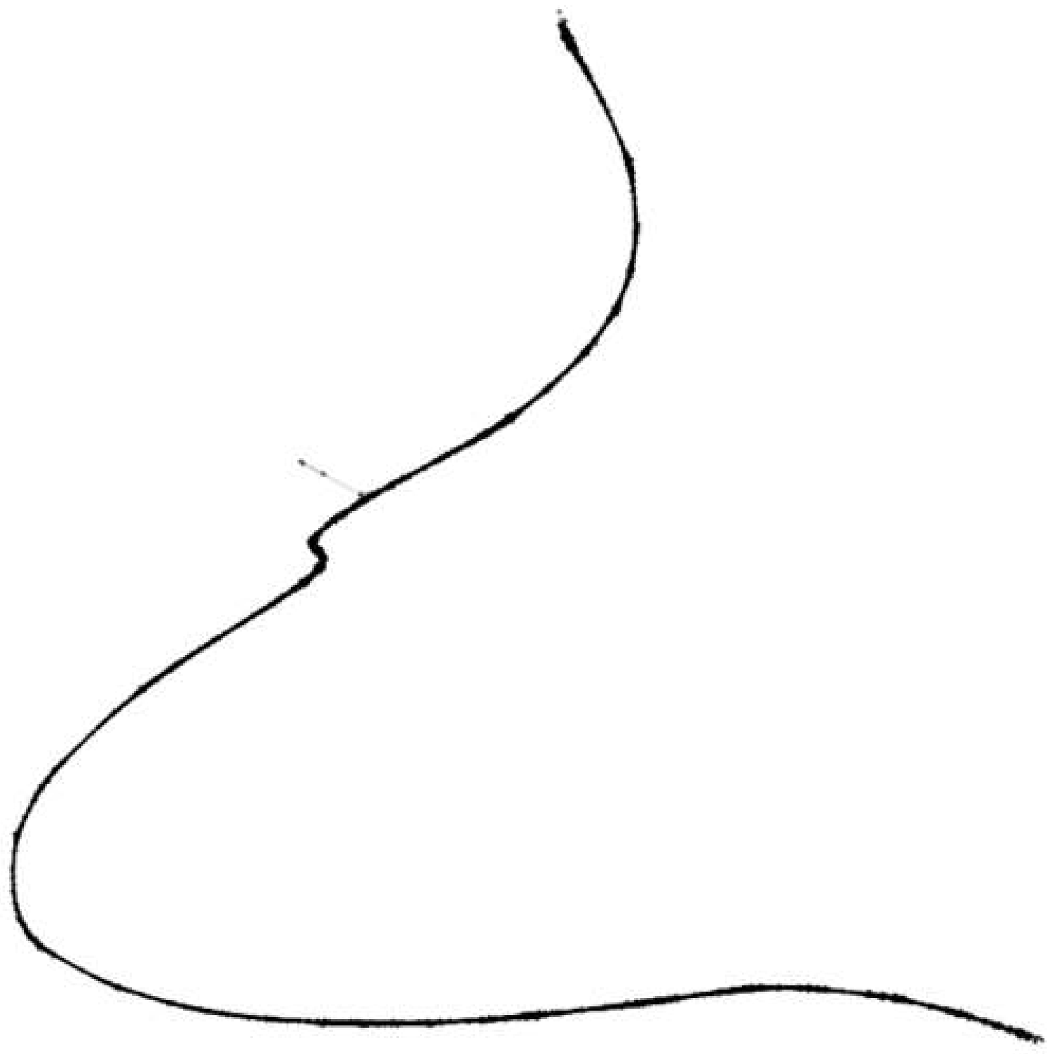

CL110
LTR_Gypsy
Length of Reads (GP):1438 (0.07%)

Hbalanensis

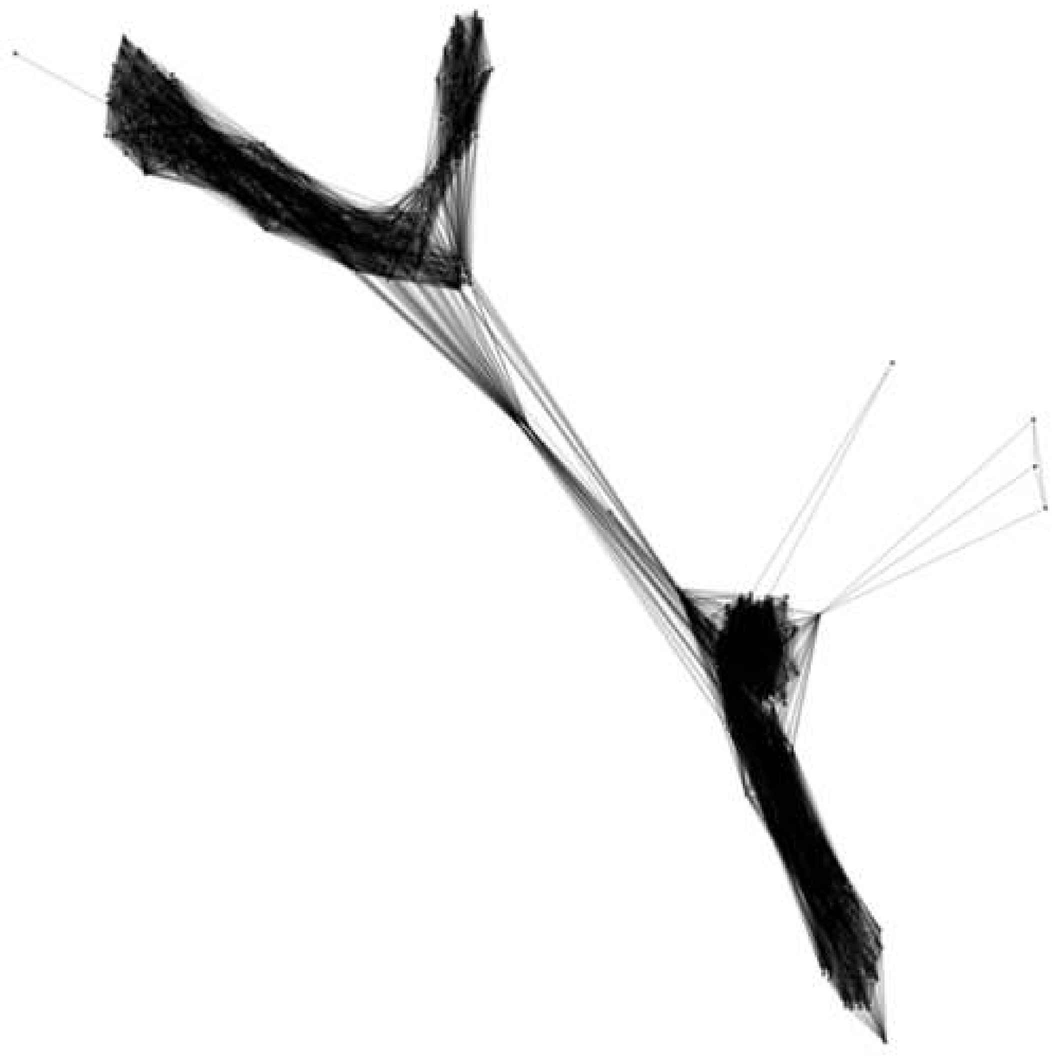

CL111
Low_complexity
Length of Reads (GP):277 (0.02%)

Tgrandiflorum

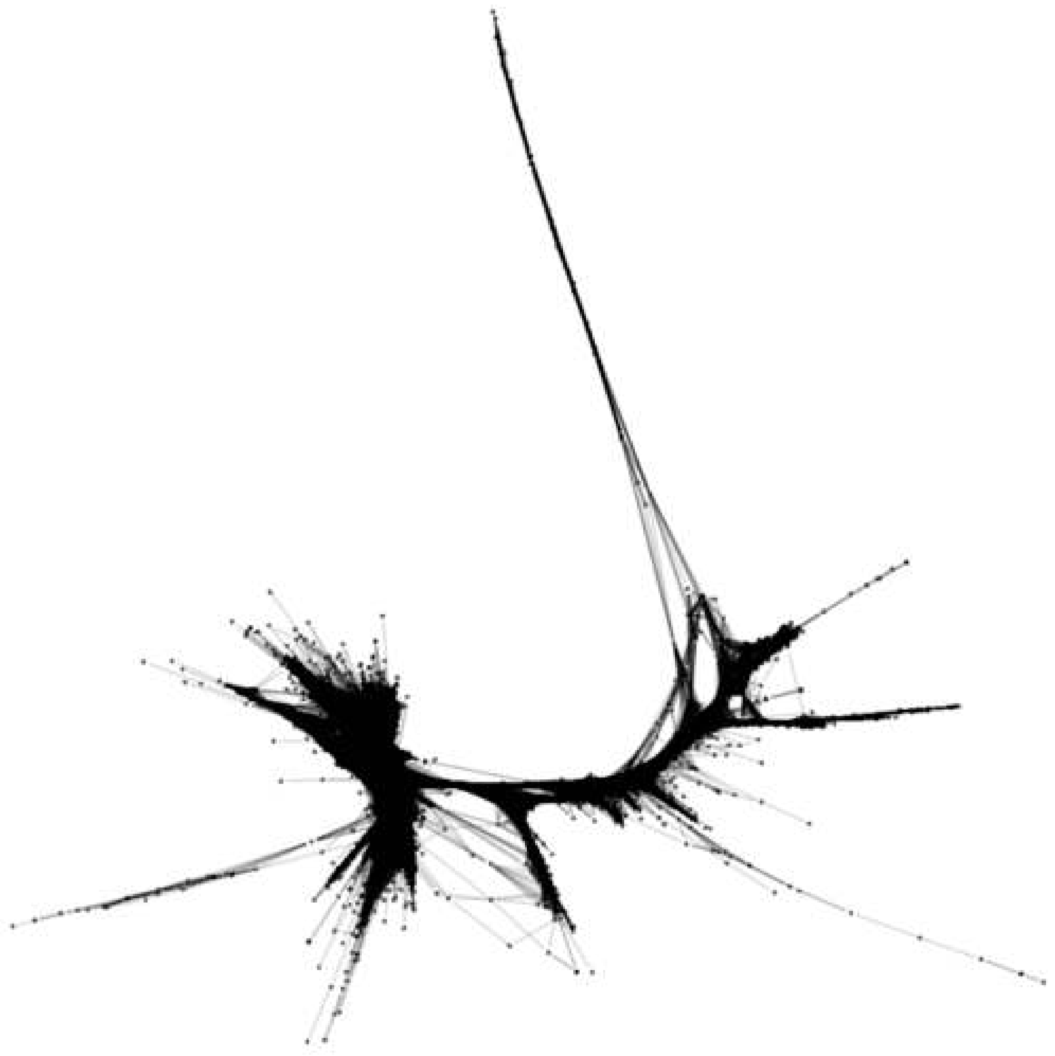

CL111
Low_complexity
Length of Reads (GP):13718 (0.17%)

Tcacao

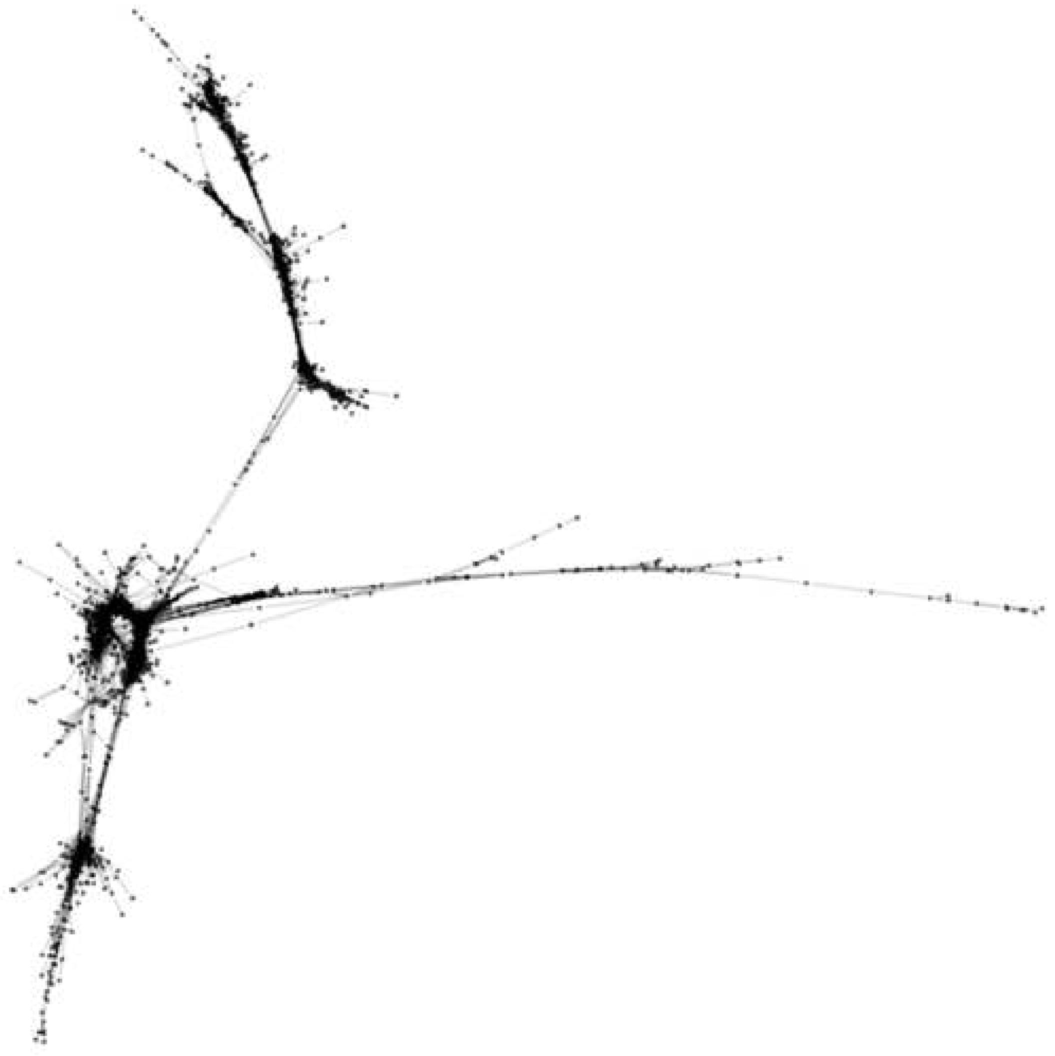

CL111
Low_complexity
Length of Reads (GP):1327 (0.06%)

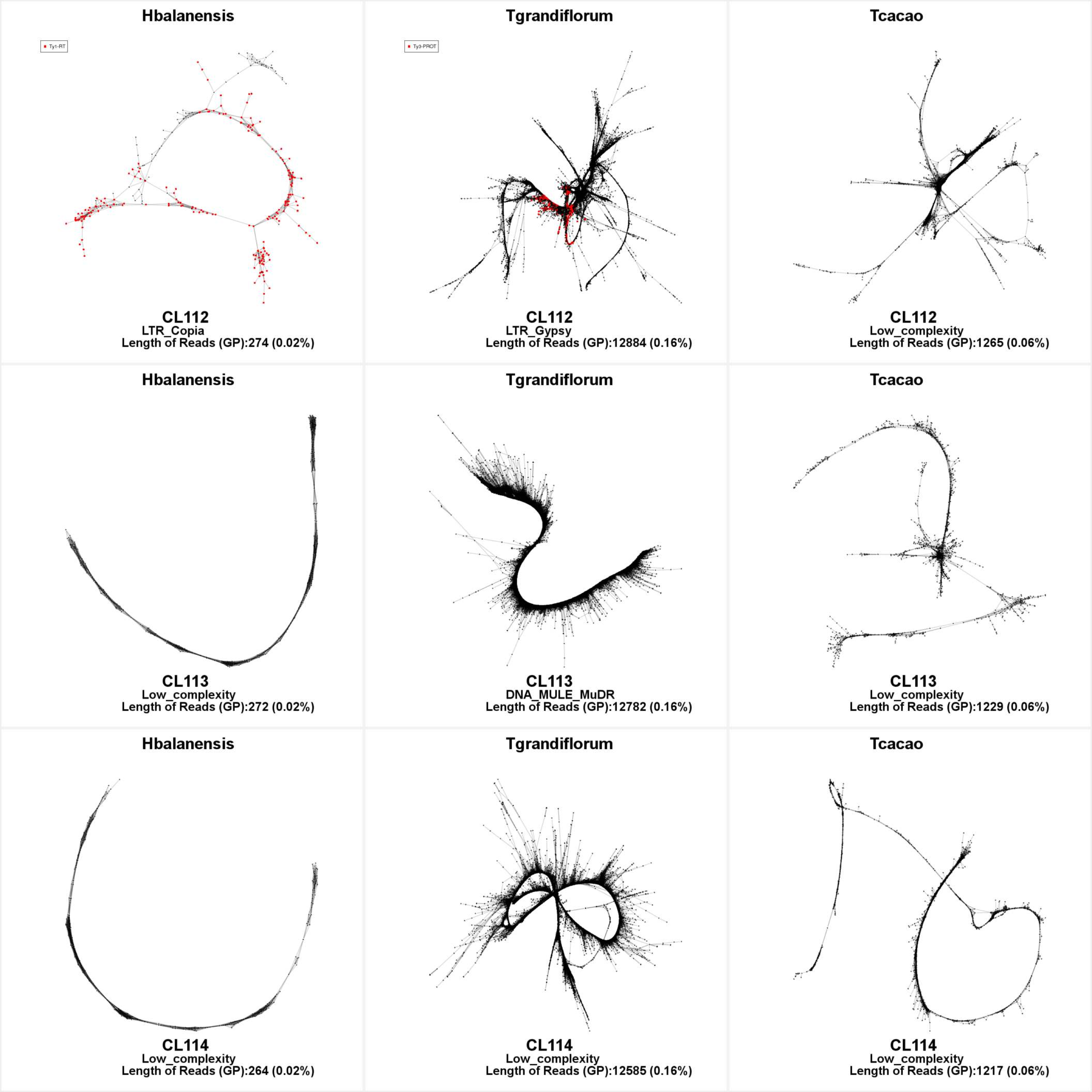
Hbalanensis
Ty1-RT
CL112
LTR_Copia
Length of Reads (GP):274 (0.02%)
Tgrandiflorum
Ty3-PROT
CL112
LTR_Gypsy
Length of Reads (GP):12884 (0.16%)
Tcacao
CL112
Low_complexity
Length of Reads (GP):1265 (0.06%)
Hbalanensis
CL113
Low_complexity
Length of Reads (GP):272 (0.02%)
Tgrandiflorum
CL113
DNA_MULE_MuDR
Length of Reads (GP):12782 (0.16%)
Tcacao
CL113
Low_complexity
Length of Reads (GP):1229 (0.06%)
Hbalanensis
CL114
Low_complexity
Length of Reads (GP):264 (0.02%)
Tgrandiflorum
CL114
Low_complexity
Length of Reads (GP):12585 (0.16%)
Tcacao
CL114
Low_complexity
Length of Reads (GP):1217 (0.06%)

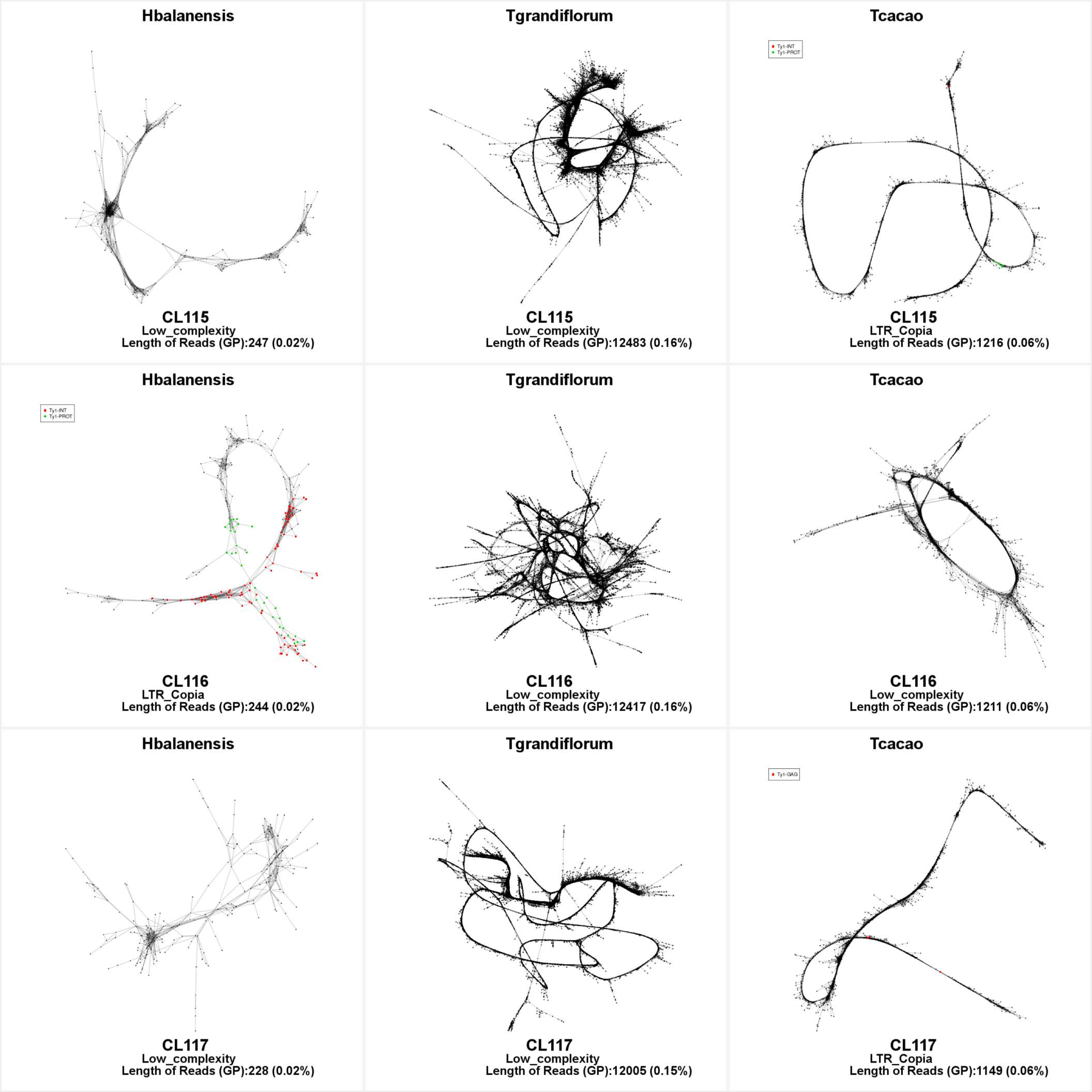

Hbalanensis
CL115
Low_complexity
Length of Reads (GP):247 (0.02%)
Tgrandiflorum
CL115
Low_complexity
Length of Reads (GP):12483 (0.16%)
Tcacao
Ty1-INT
Ty1-PROT
CL115
LTR_Copia
Length of Reads (GP):1216 (0.06%)
Hbalanensis
Ty1-INT
Ty1-PROT
CL116
LTR_Copia
Length of Reads (GP):244 (0.02%)
Tgrandiflorum
CL116
Low_complexity
Length of Reads (GP):12417 (0.16%)
Tcacao
CL116
Low_complexity
Length of Reads (GP):1211 (0.06%)
Hbalanensis
CL117
Low_complexity
Length of Reads (GP):228 (0.02%)
Tgrandiflorum
CL117
Low_complexity
Length of Reads (GP):12005 (0.15%)
Tcacao
Ty1-GAG
CL117
LTR_Copia
Length of Reads (GP):1149 (0.06%)

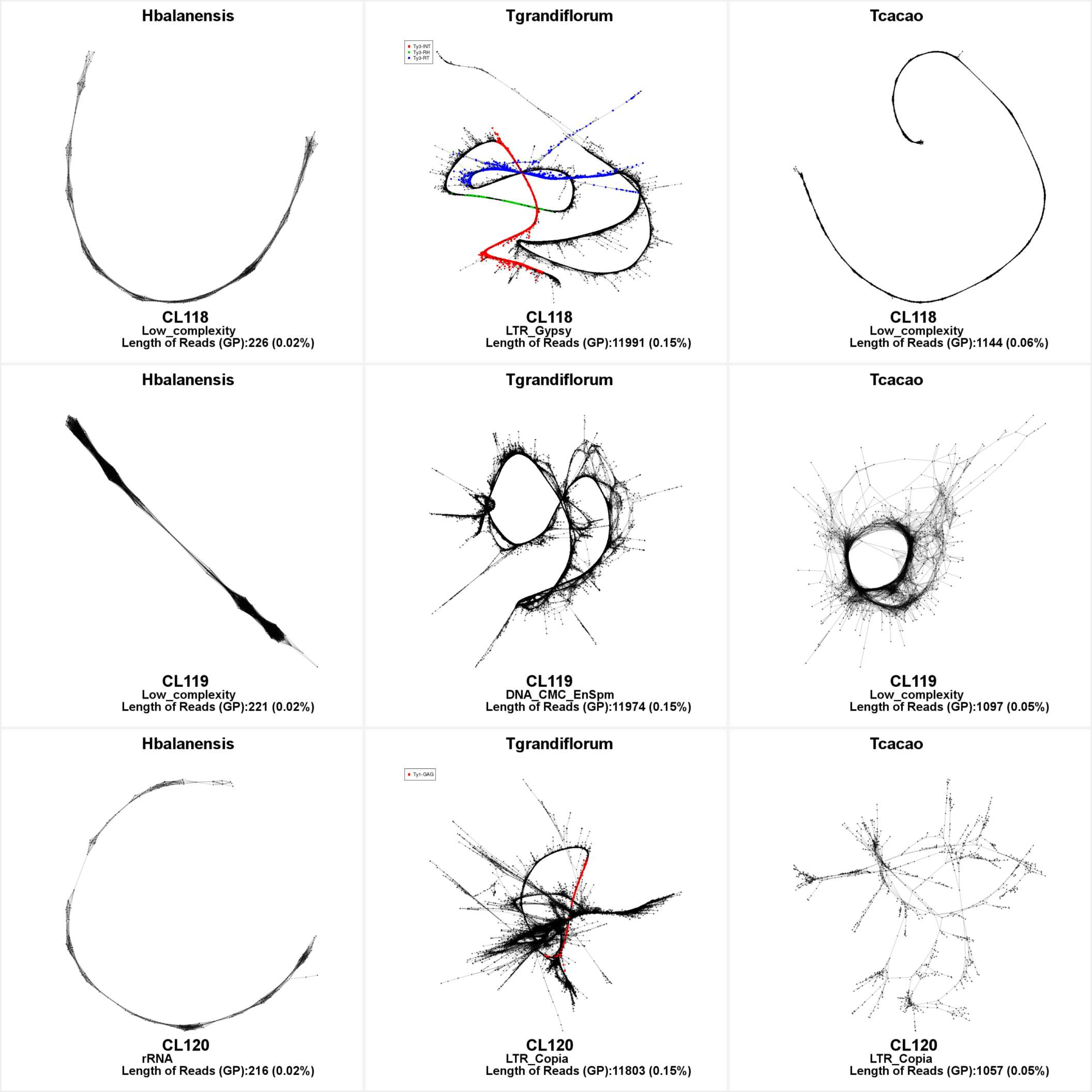
Hbalanensis
CL118
Low_complexity
Length of Reads (GP):226 (0.02%)
Tgrandiflorum
Ty3-INT
Ty3-RH
Ty3-RT
CL118
LTR_Gypsy
Length of Reads (GP):11991 (0.15%)
Tcacao
CL118
Low_complexity
Length of Reads (GP):1144 (0.06%)
Hbalanensis
CL119
Low_complexity
Length of Reads (GP):221 (0.02%)
Tgrandiflorum
CL119
DNA_CMC_EnSpm
Length of Reads (GP):11974 (0.15%)
Tcacao
CL119
Low_complexity
Length of Reads (GP):1097 (0.05%)
Hbalanensis
CL120
rRNA
Length of Reads (GP):216 (0.02%)
Tgrandiflorum
Ty1-GAG
CL120
LTR_Copia
Length of Reads (GP):11803 (0.15%)
Tcacao
CL120
LTR_Copia
Length of Reads (GP):1057 (0.05%)

Hbalanensis

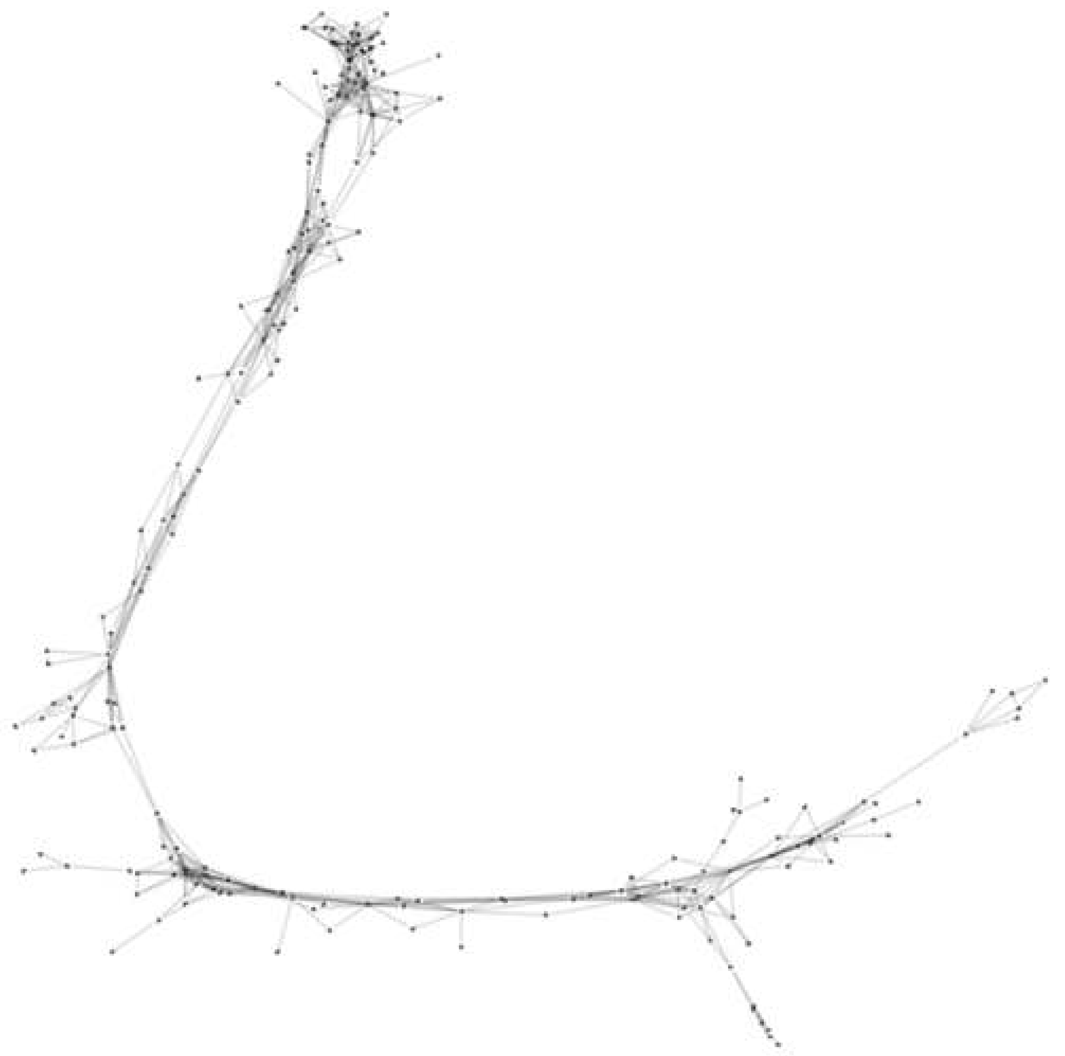

CL121
Low_complexity
Length of Reads (GP):206 (0.02%)

Tgrandiflorum

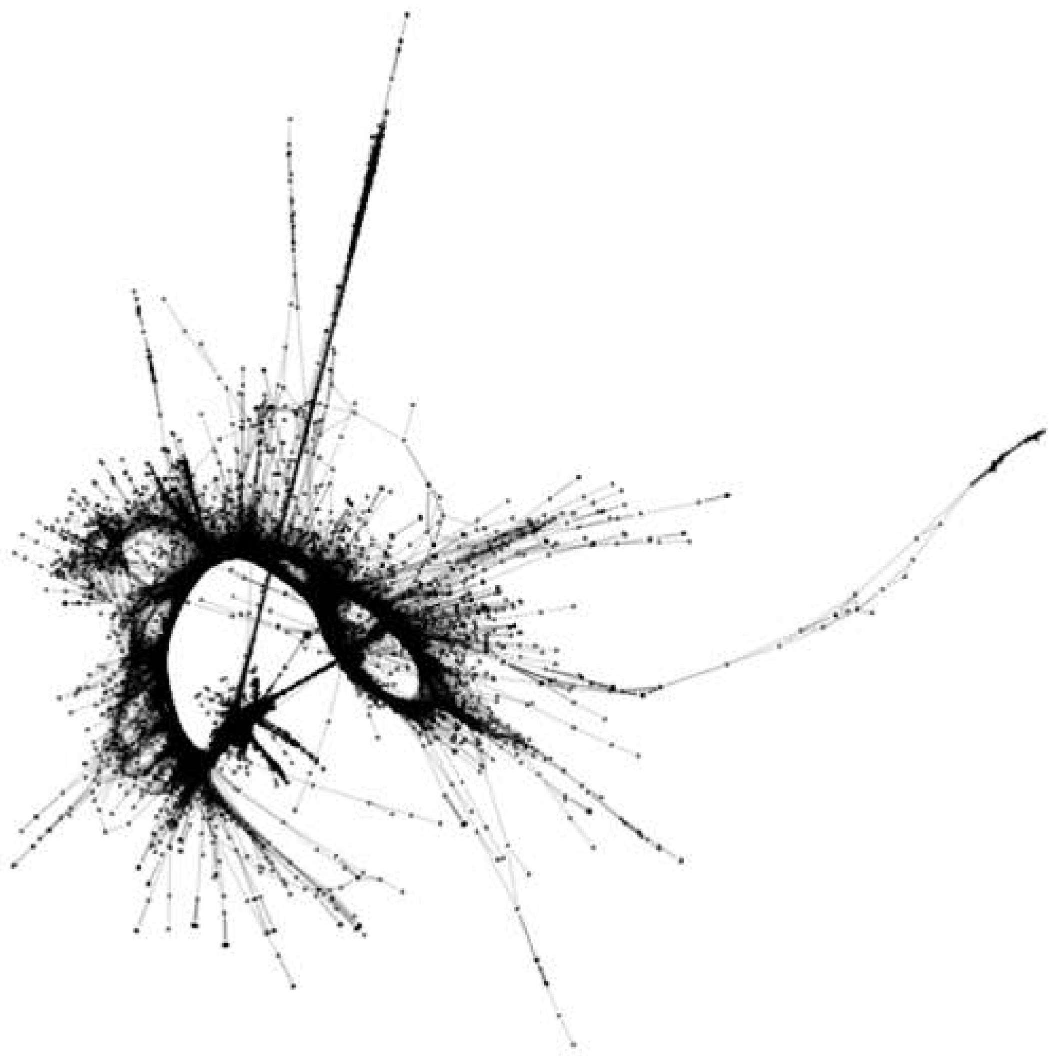

CL121
Low_complexity
Length of Reads (GP):11794 (0.15%)

Tcacao

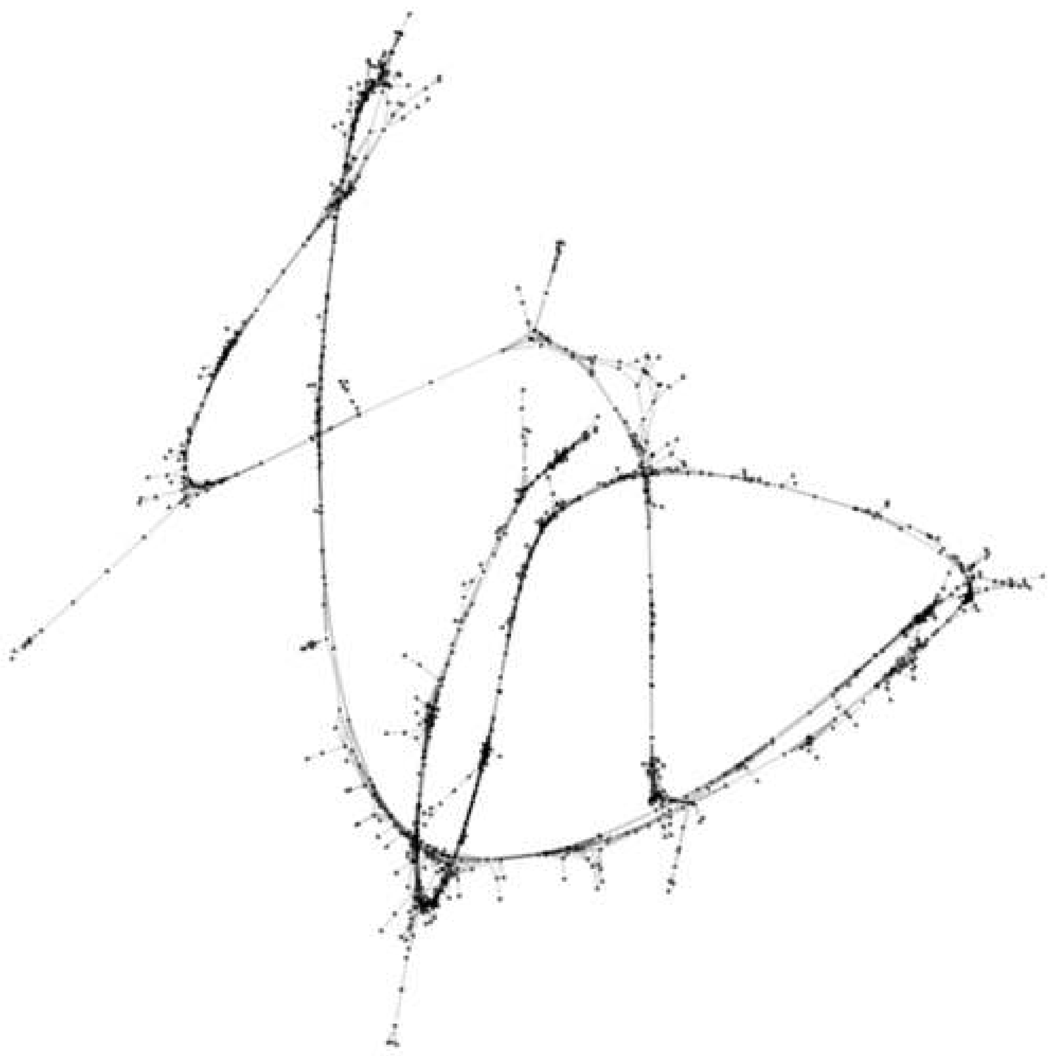

CL121
DNA_CMC_EnSpm
Length of Reads (GP):1032 (0.05%)

Hbalanensis

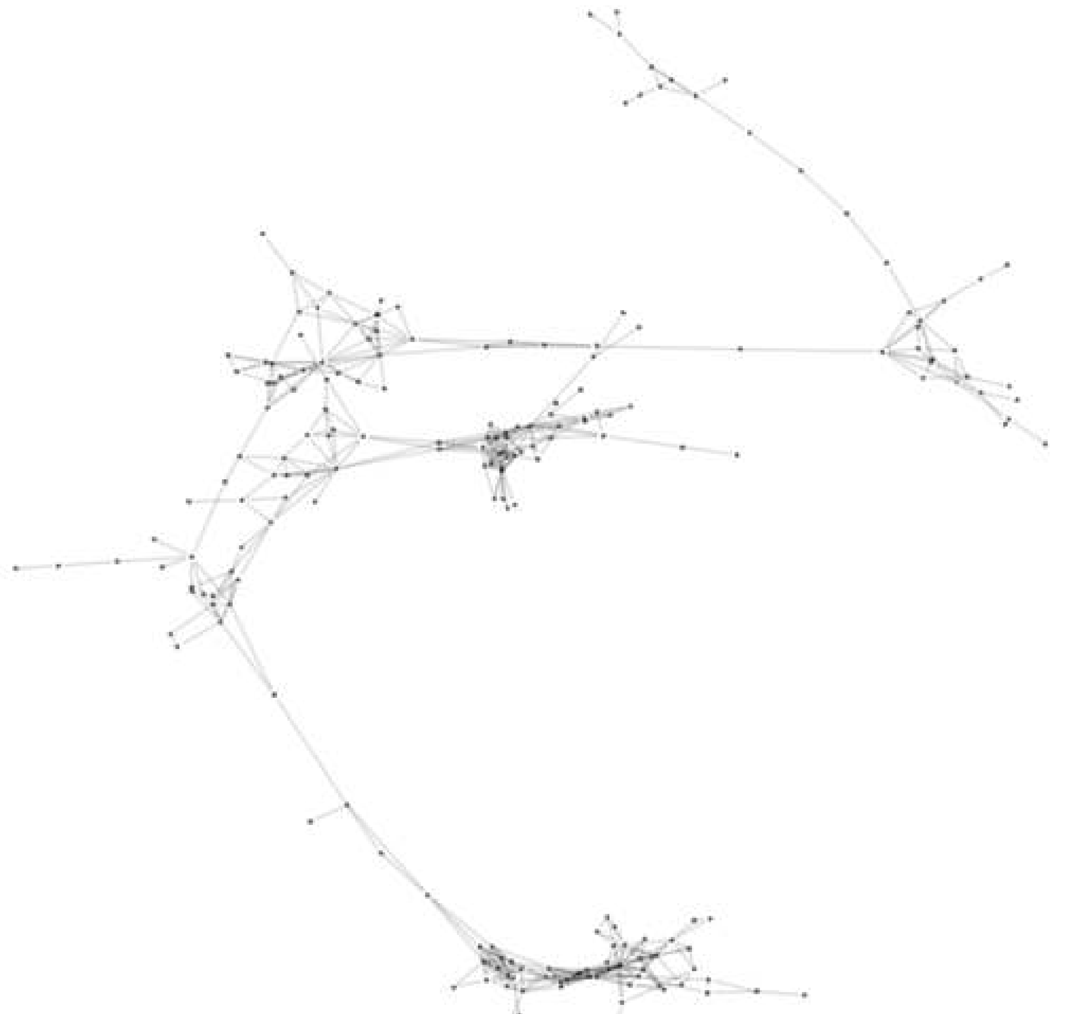

CL122
LTR_Copia
Length of Reads (GP):206 (0.02%)

Tgrandiflorum

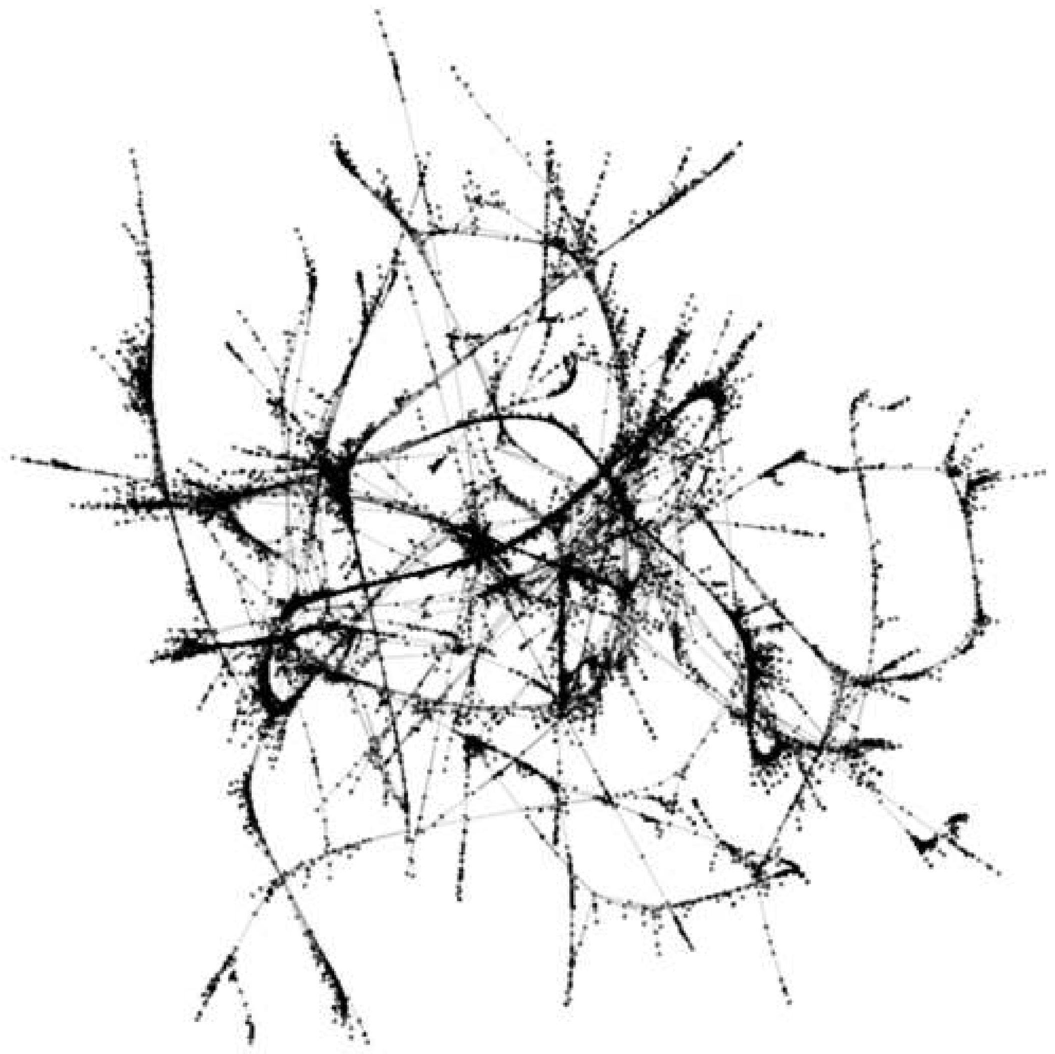

CL122
Low_complexity
Length of Reads (GP):11687 (0.15%)

Tcacao

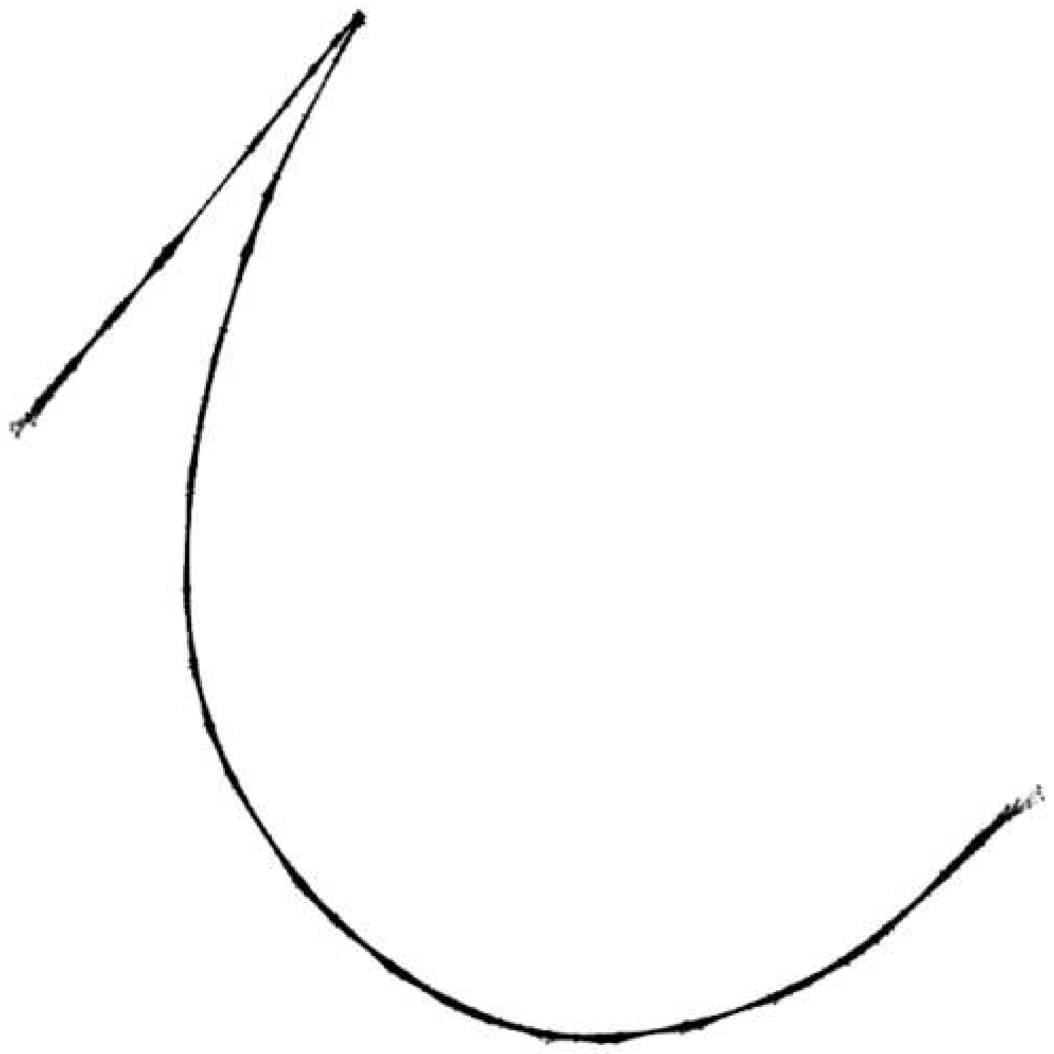

CL122
LTR_Gypsy
Length of Reads (GP):996 (0.05%)

Hbalanensis

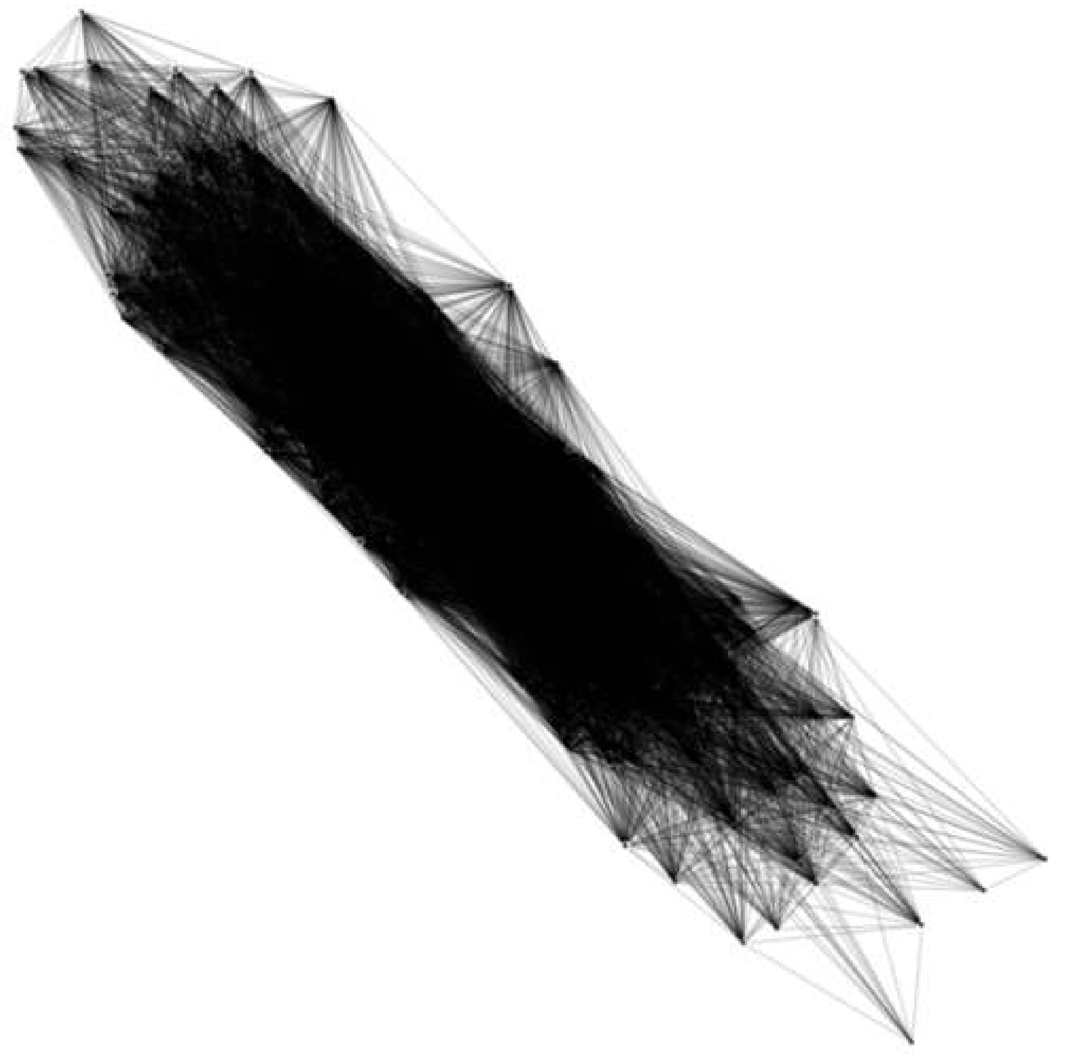

CL123
Low_complexity
Length of Reads (GP):203 (0.02%)

Tgrandiflorum

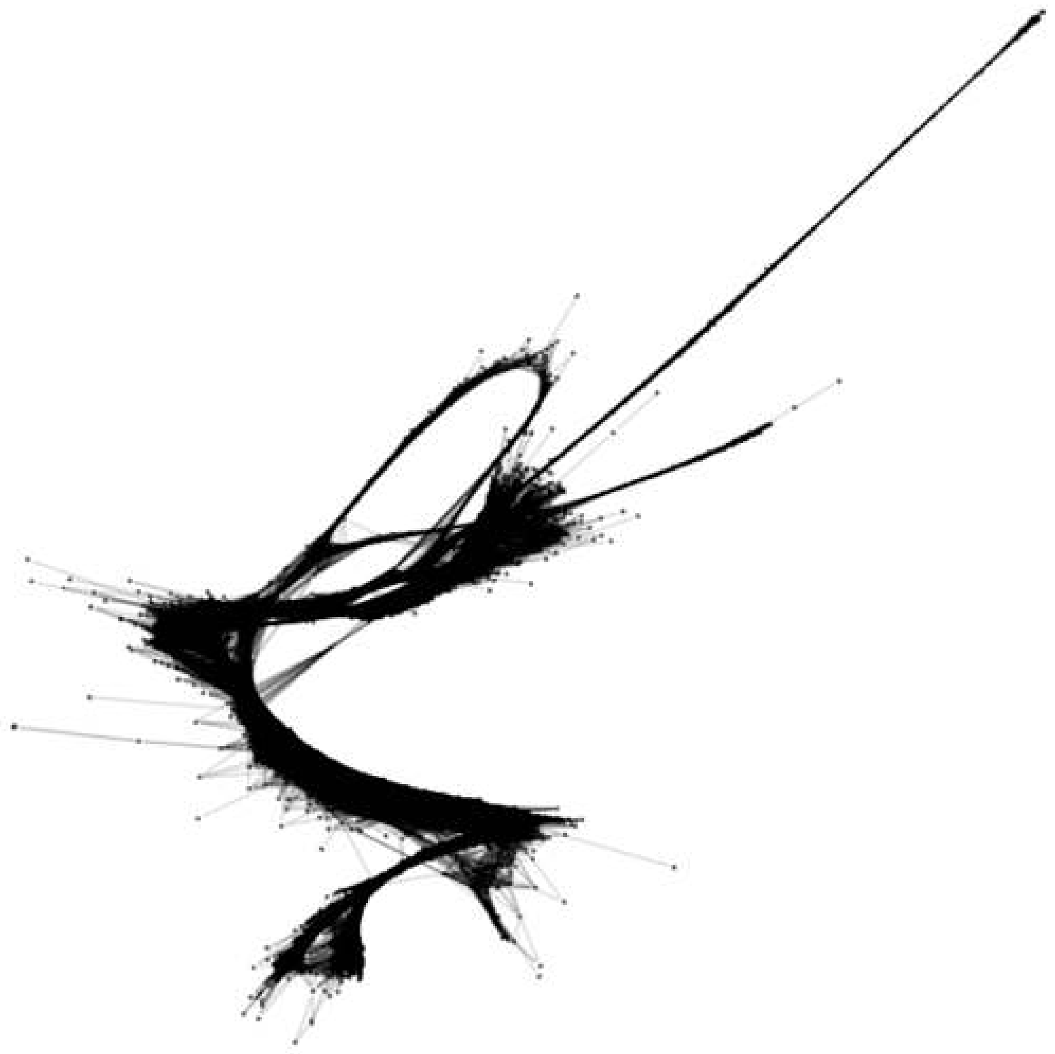

CL123
Low_complexity
Length of Reads (GP):11652 (0.15%)

Tcacao

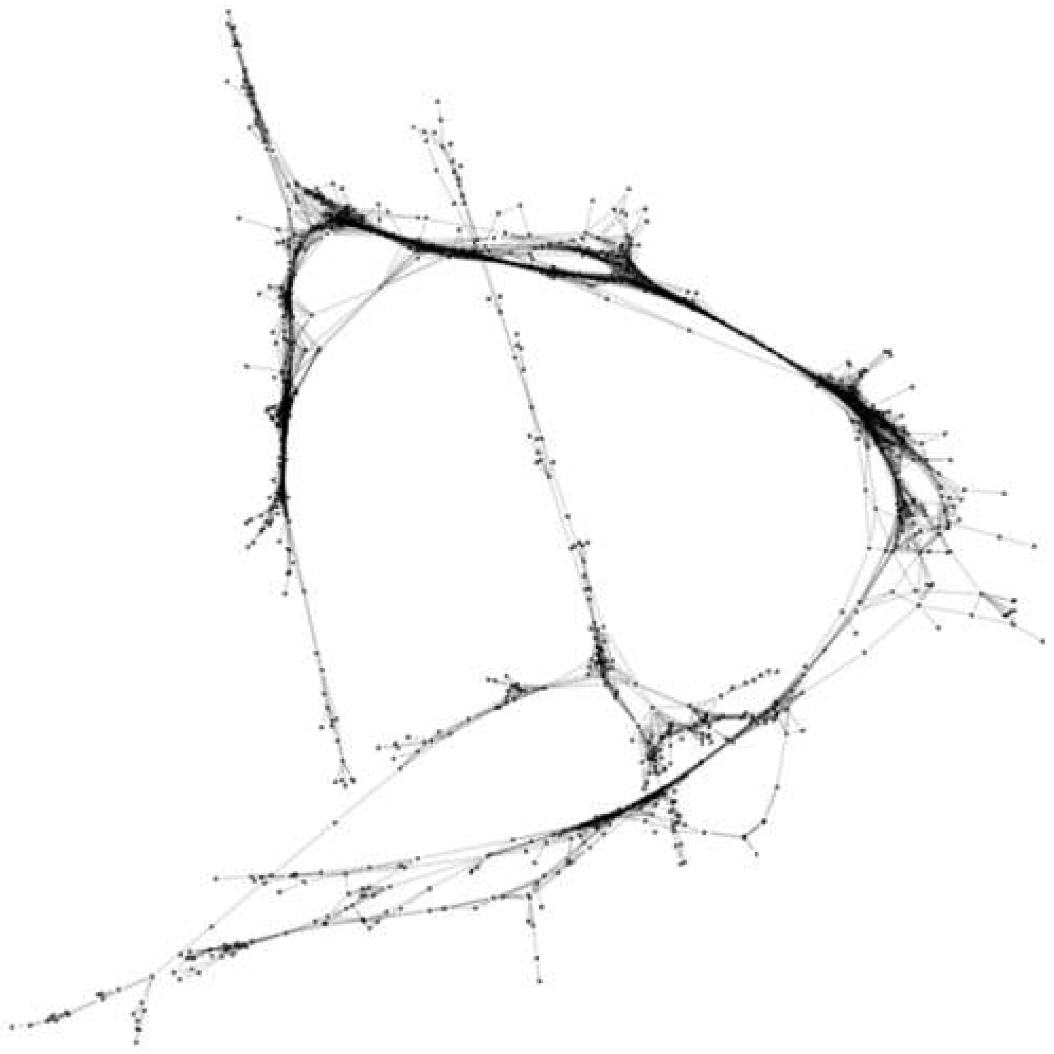

CL123
Low_complexity
Length of Reads (GP):992 (0.05%)

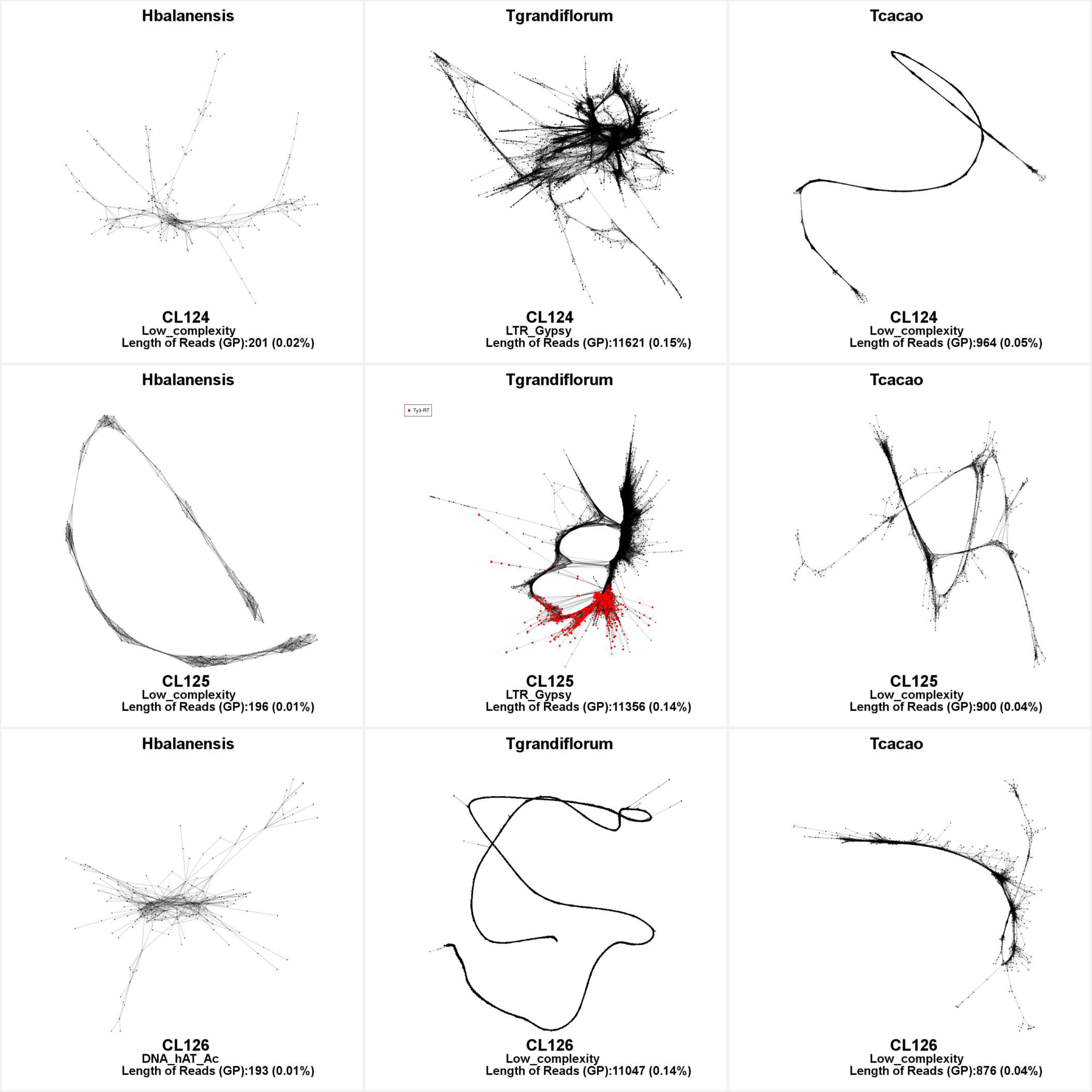

Hbalanensis
CL124
Low_complexity
Length of Reads (GP):201 (0.02%)
Tgrandiflorum
CL124
LTR_Gypsy
Length of Reads (GP):11621 (0.15%)
Tcacao
CL124
Low_complexity
Length of Reads (GP):964 (0.05%)
Hbalanensis
CL125
Low_complexity
Length of Reads (GP):196 (0.01%)
Tgrandiflorum
Ty3-RT
CL125
LTR_Gypsy
Length of Reads (GP):11356 (0.14%)
Tcacao
CL125
Low_complexity
Length of Reads (GP):900 (0.04%)
Hbalanensis
CL126
DNA_hAT_Ac
Length of Reads (GP):193 (0.01%)
Tgrandiflorum
CL126
Low_complexity
Length of Reads (GP):11047 (0.14%)
Tcacao
CL126
Low_complexity
Length of Reads (GP):876 (0.04%)

Hbalanensis

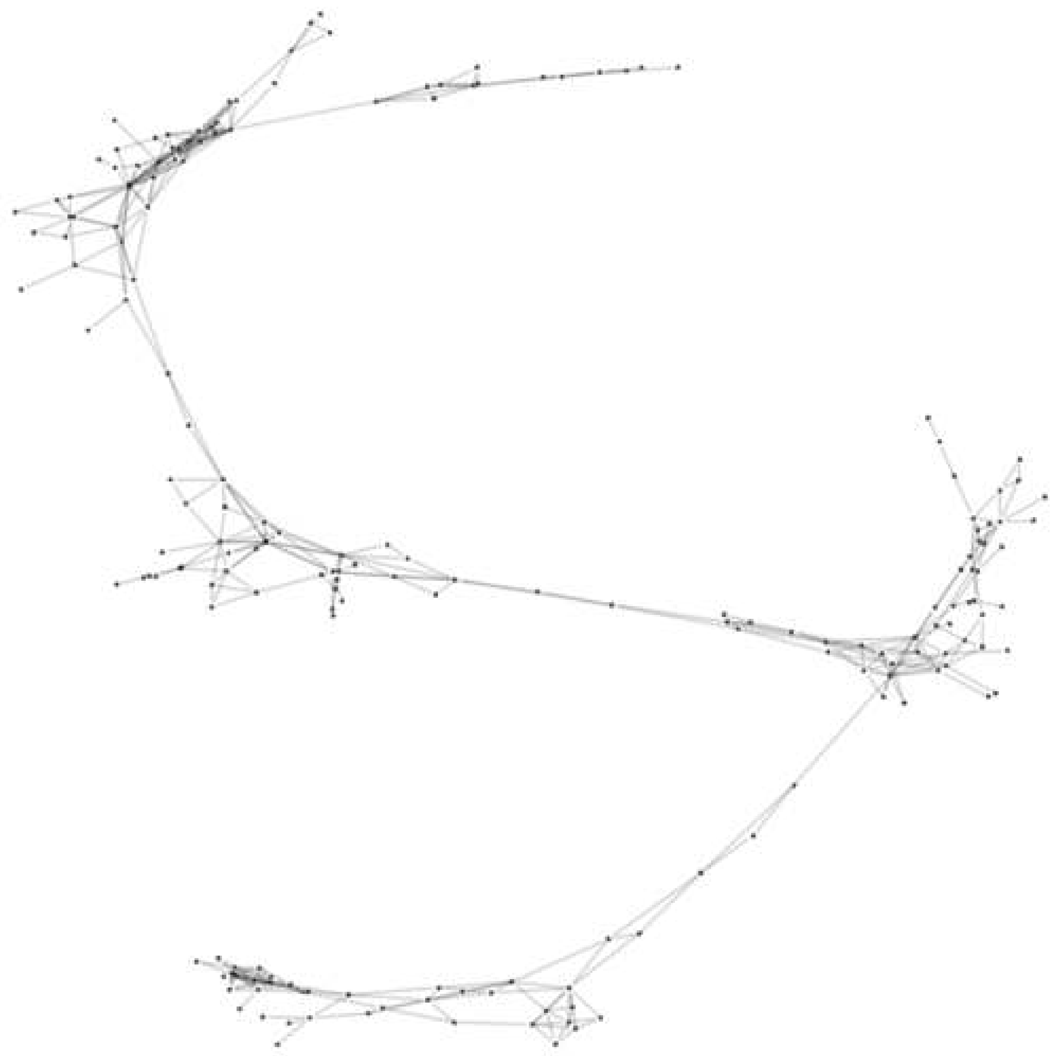

CL127
Low_complexity
Length of Reads (GP):191 (0.01%)

Tgrandiflorum

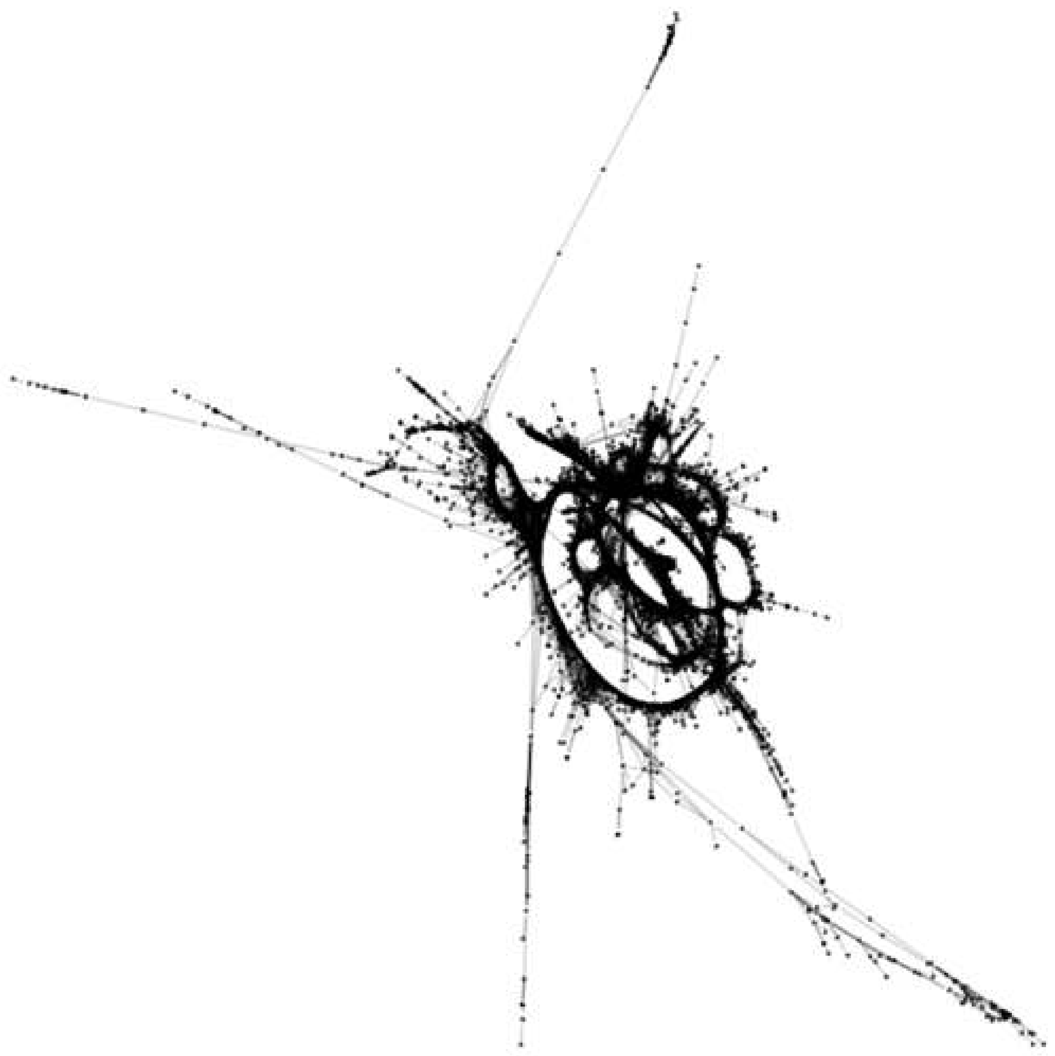

CL127
Low_complexity
Length of Reads (GP):11013 (0.14%)

Tcacao

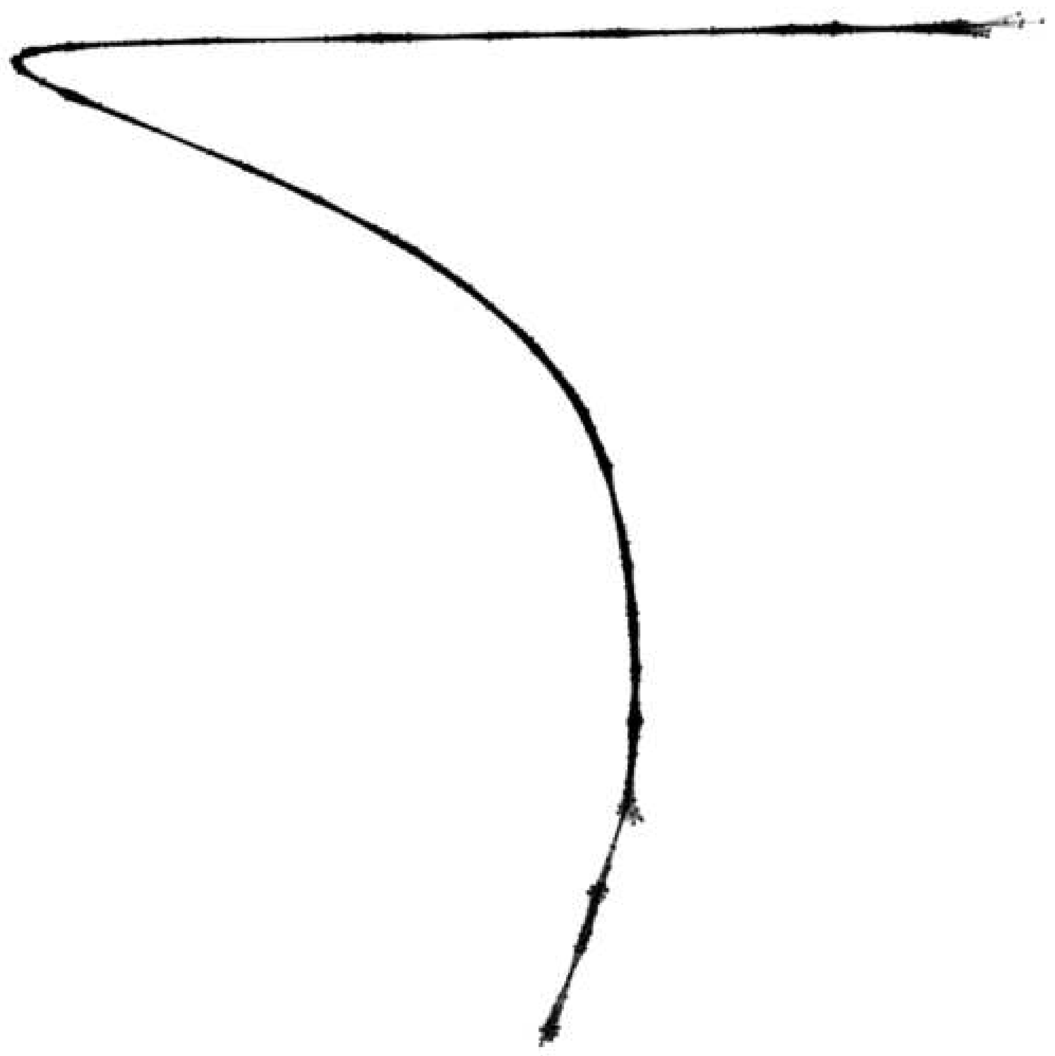

CL127
LTR_Gypsy
Length of Reads (GP):838 (0.04%)

Hbalanensis

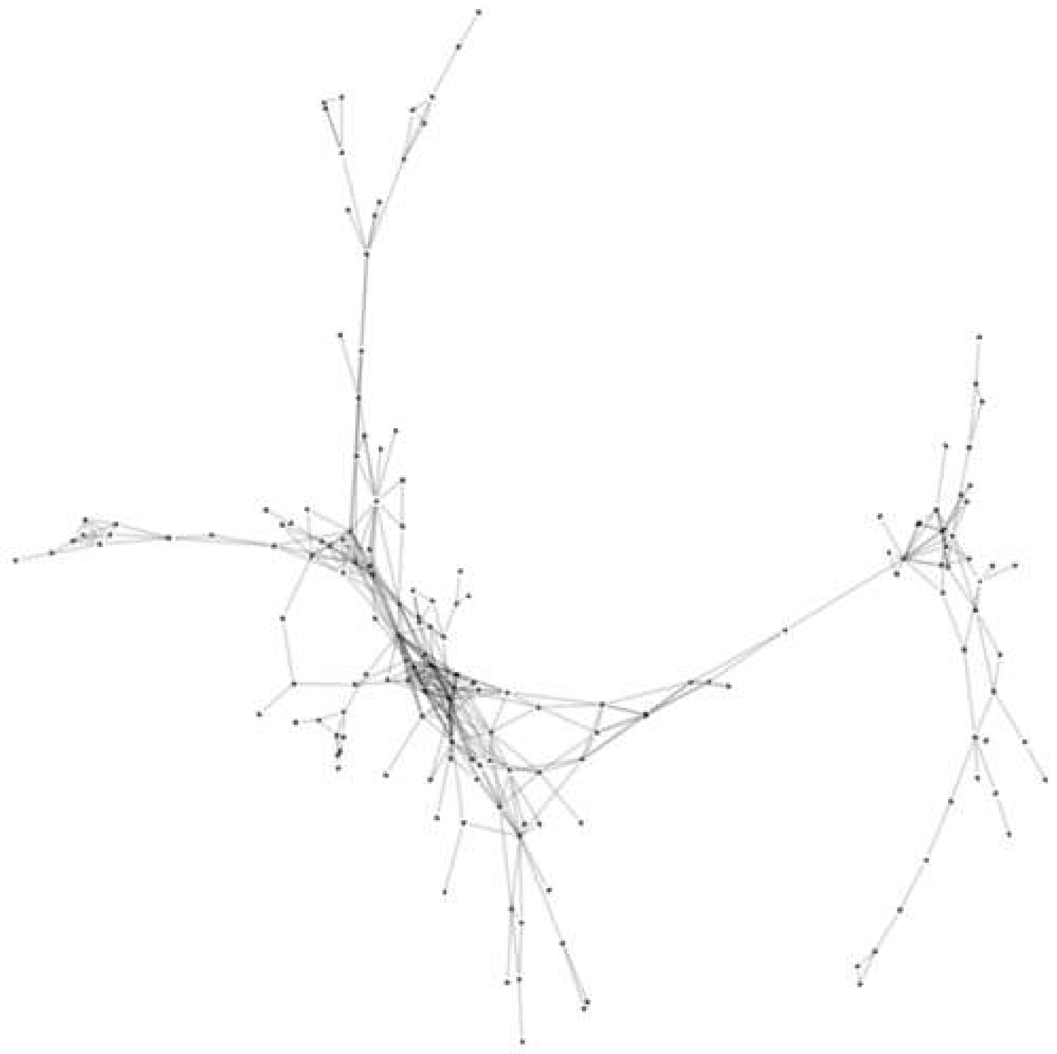

CL128
Low_complexity
Length of Reads (GP):174 (0.01%)

Tgrandiflorum

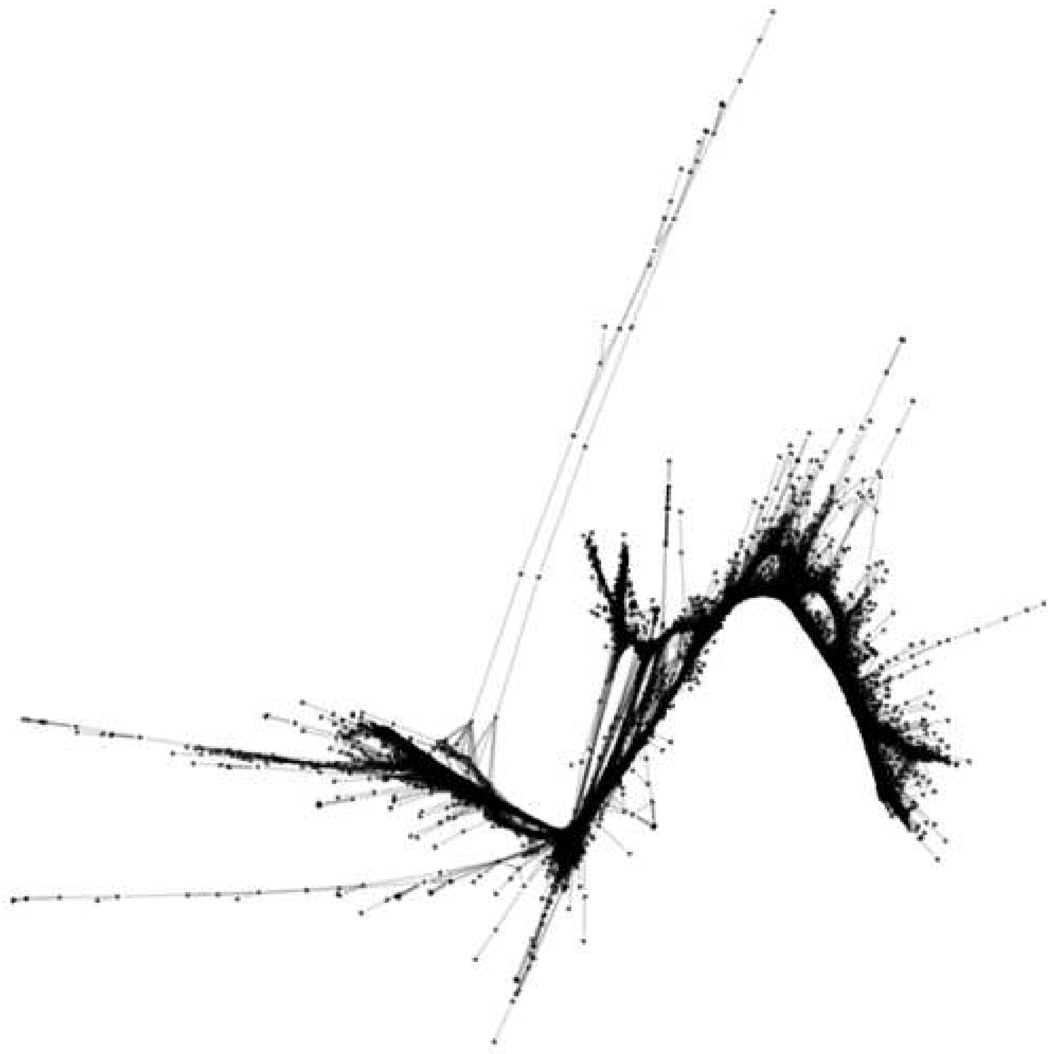

CL128
Simple_repeat
Length of Reads (GP):10842 (0.14%)

Tcacao

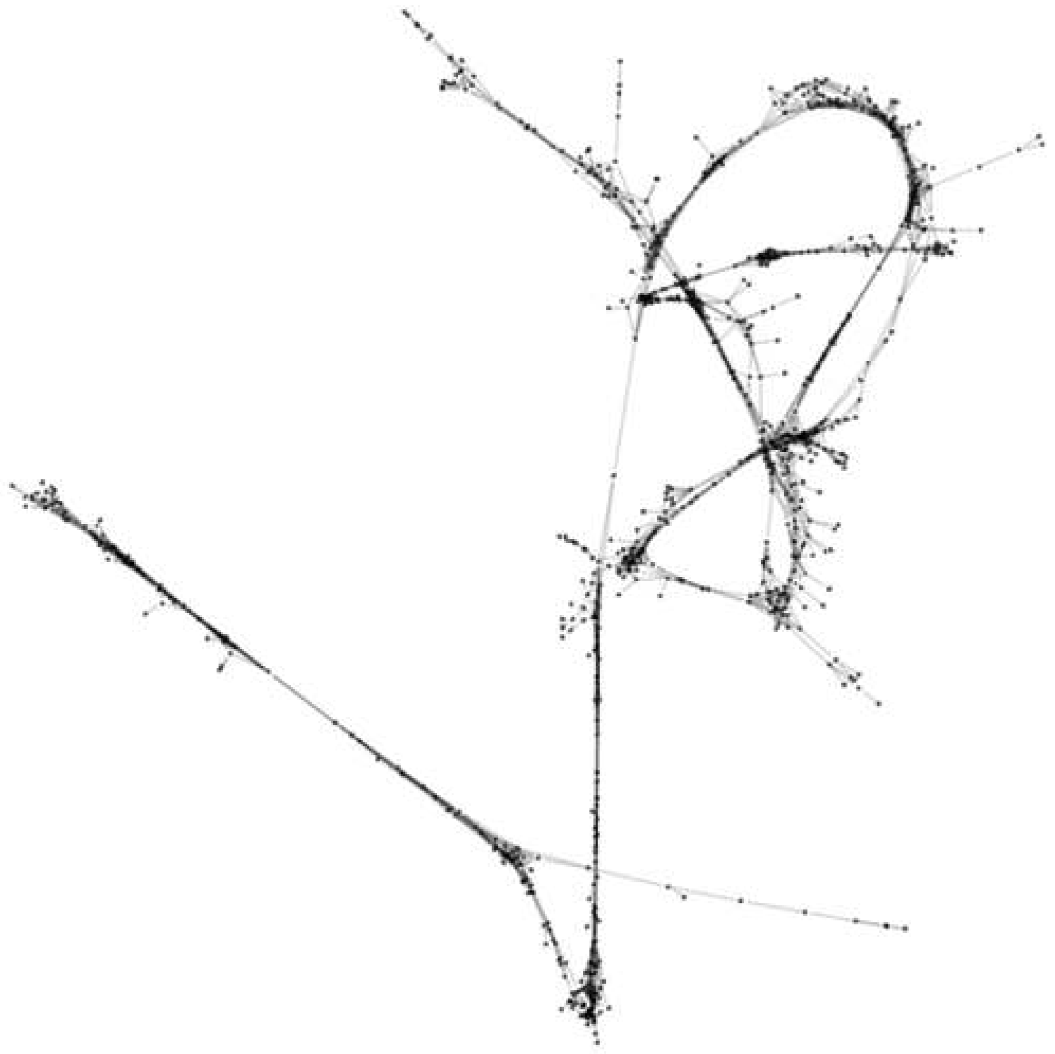

CL128
Low_complexity
Length of Reads (GP):824 (0.04%)

Hbalanensis

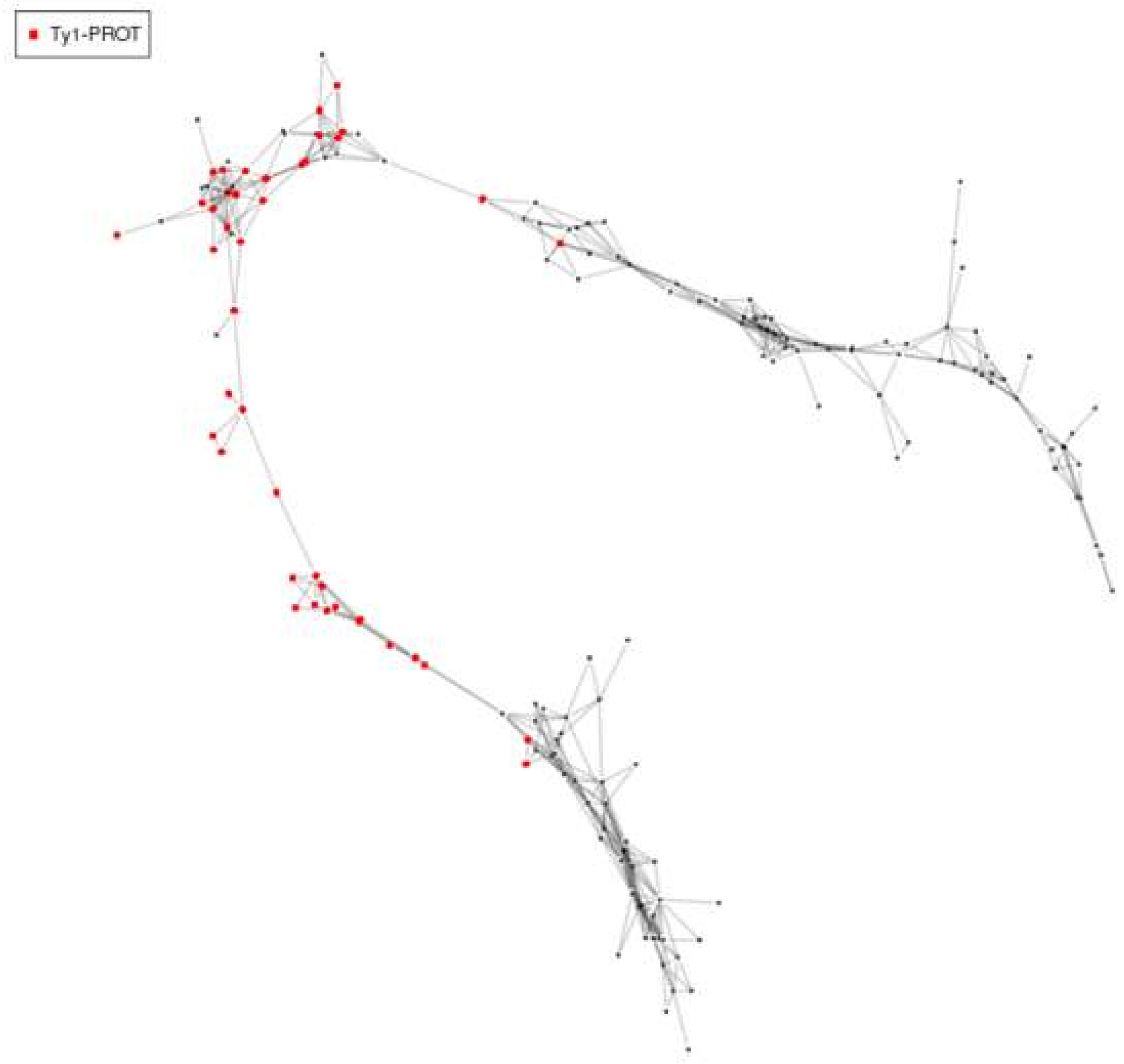

CL129
LTR_Copia
Length of Reads (GP):173 (0.01%)

Tgrandiflorum

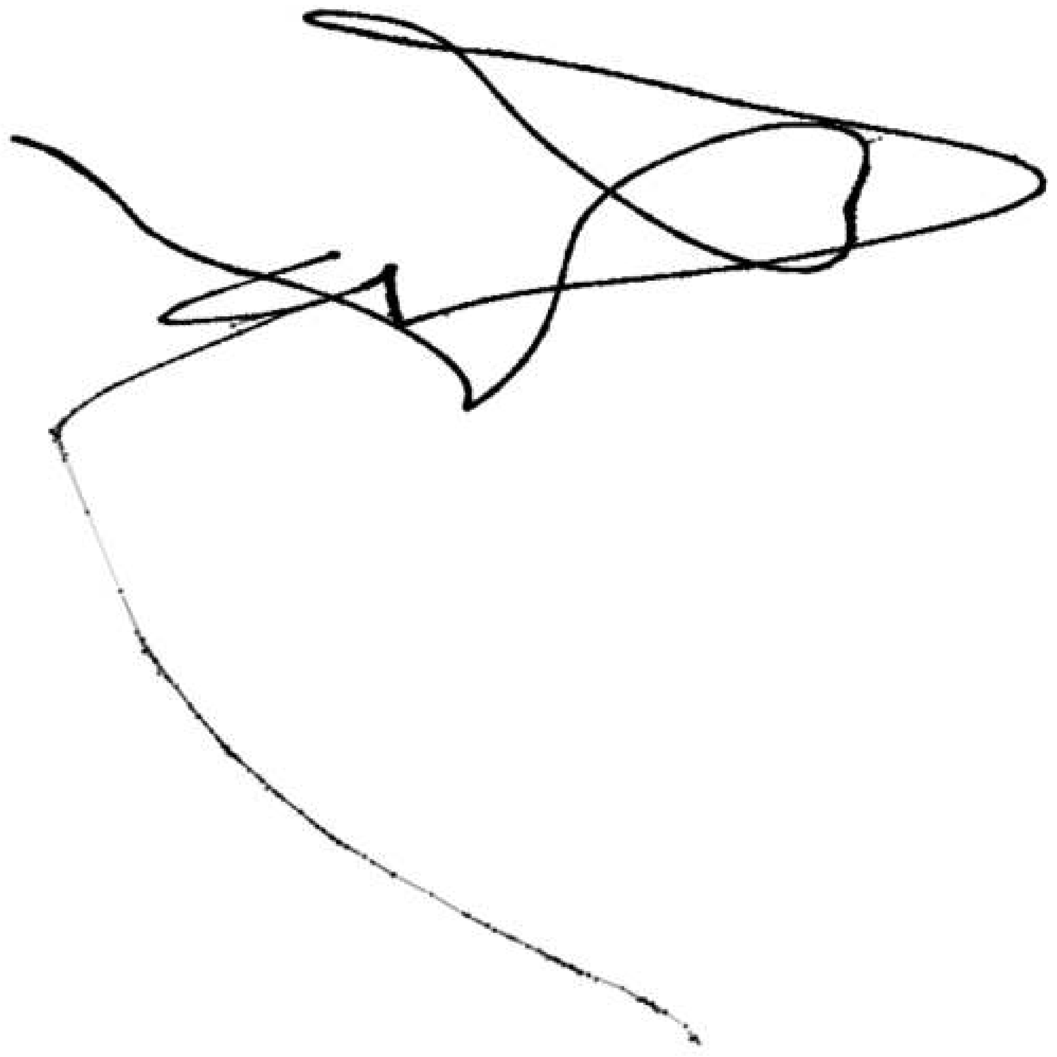

CL129
Low_complexity
Length of Reads (GP):10749 (0.14%)

Tcacao

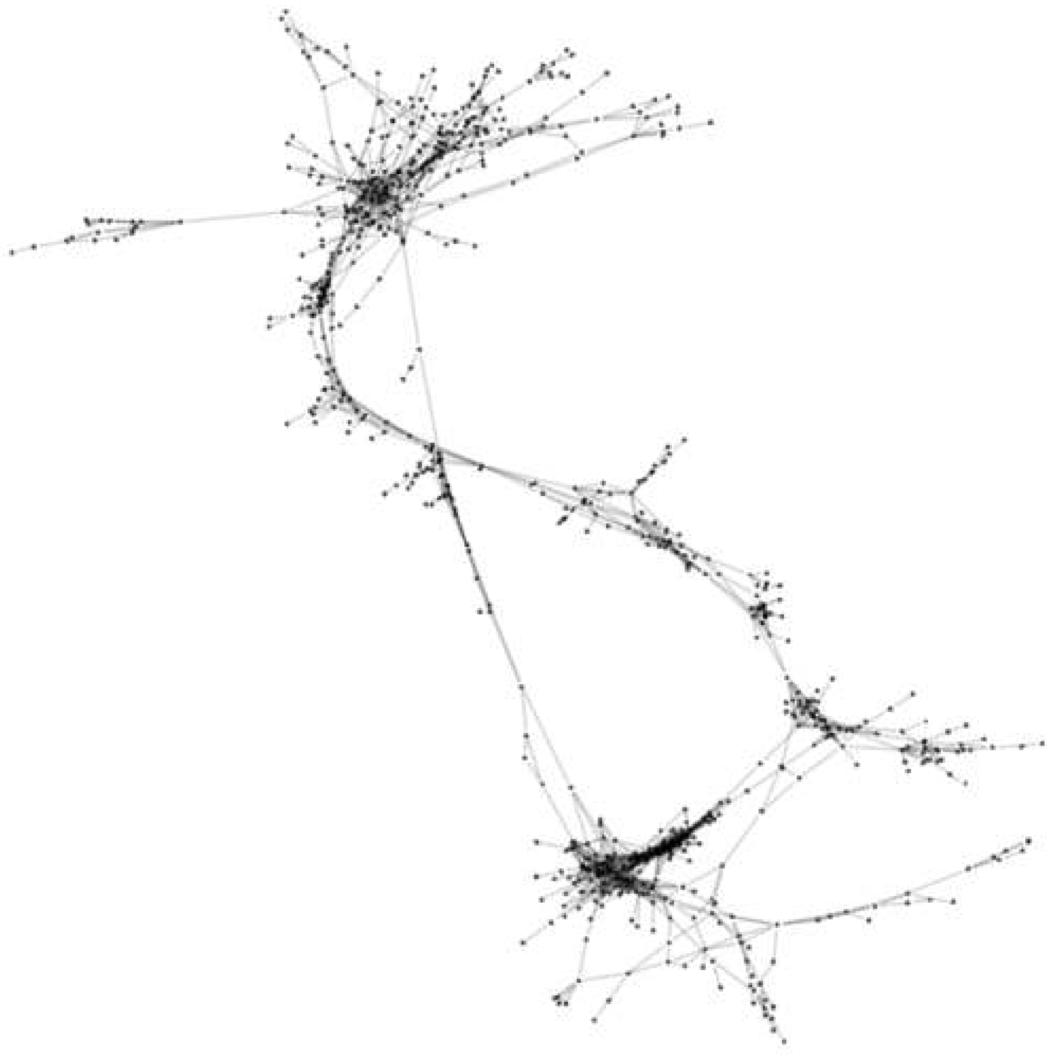

CL129
Low_complexity
Length of Reads (GP):772 (0.04%)

Hbalanensis

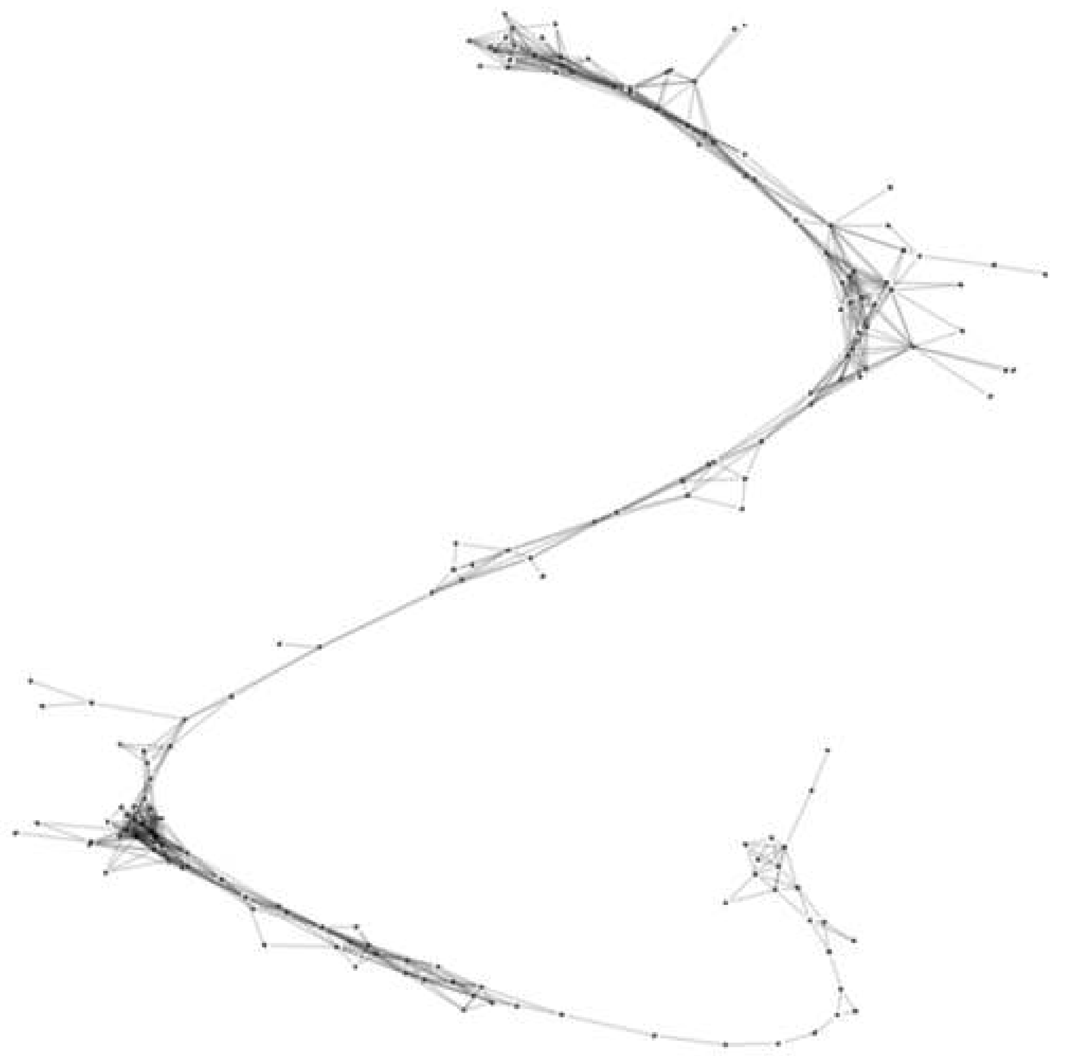

CL130
LTR_Copia
Length of Reads (GP):171 (0.01%)

Tgrandiflorum

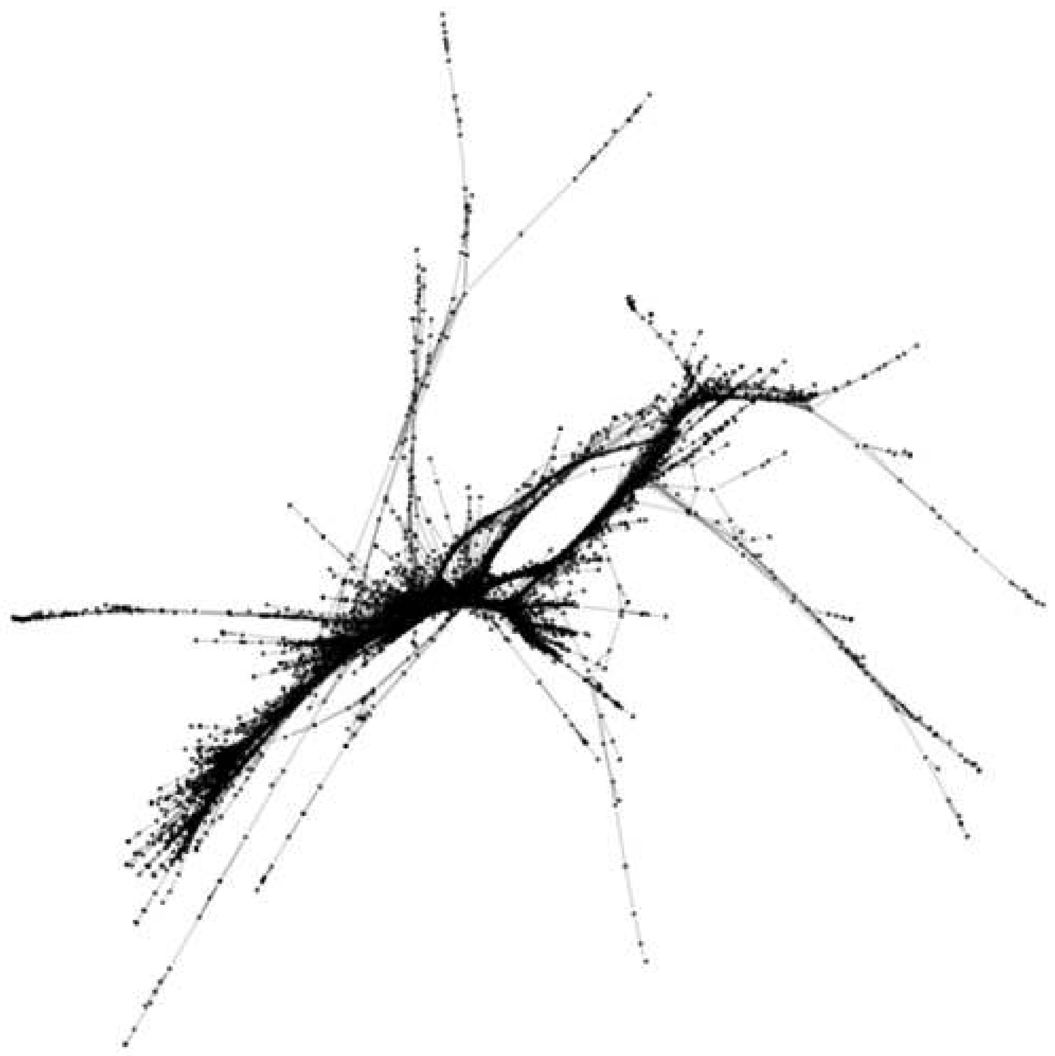

CL130
LTR_Copia
Length of Reads (GP):10731 (0.13%)

Tcacao

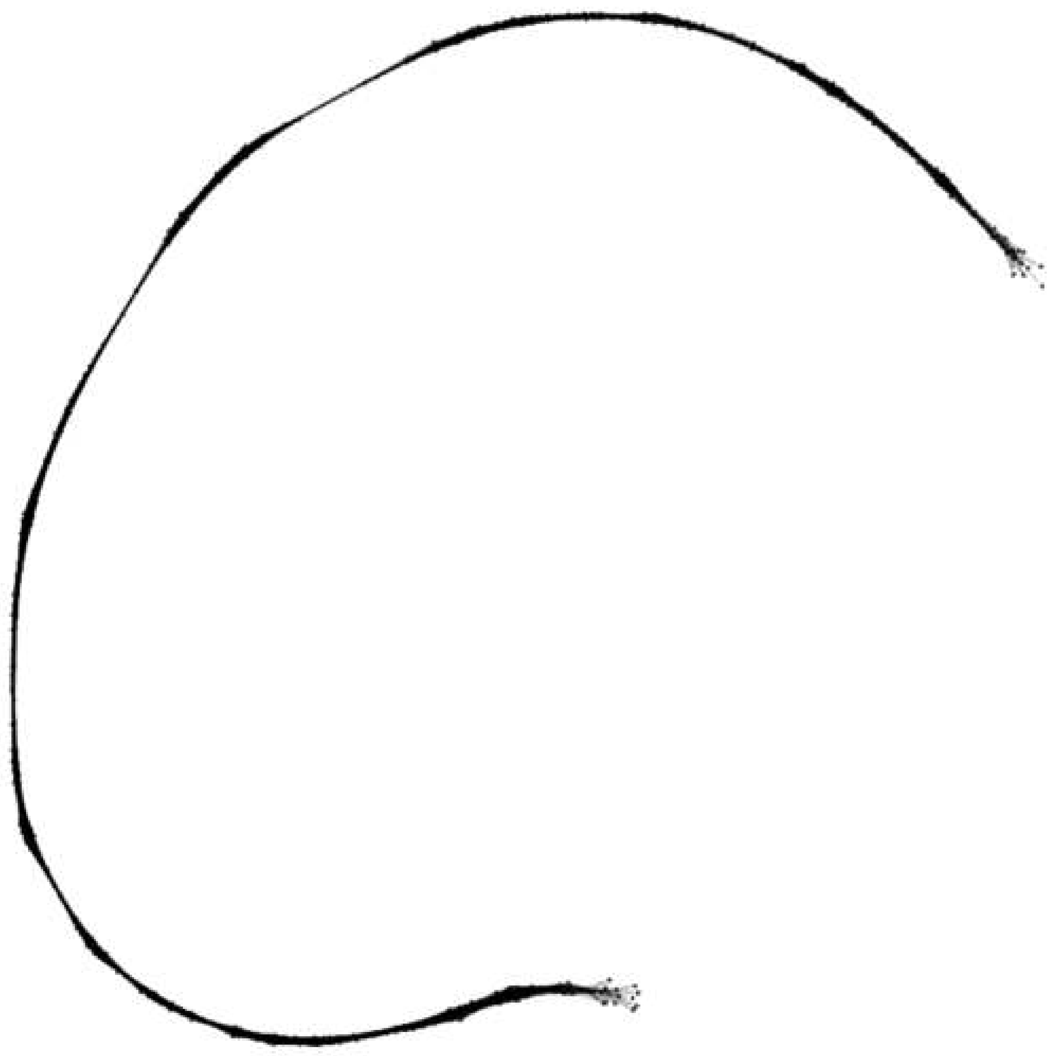

CL130
LTR_Gypsy
Length of Reads (GP):766 (0.04%)

Hbalanensis

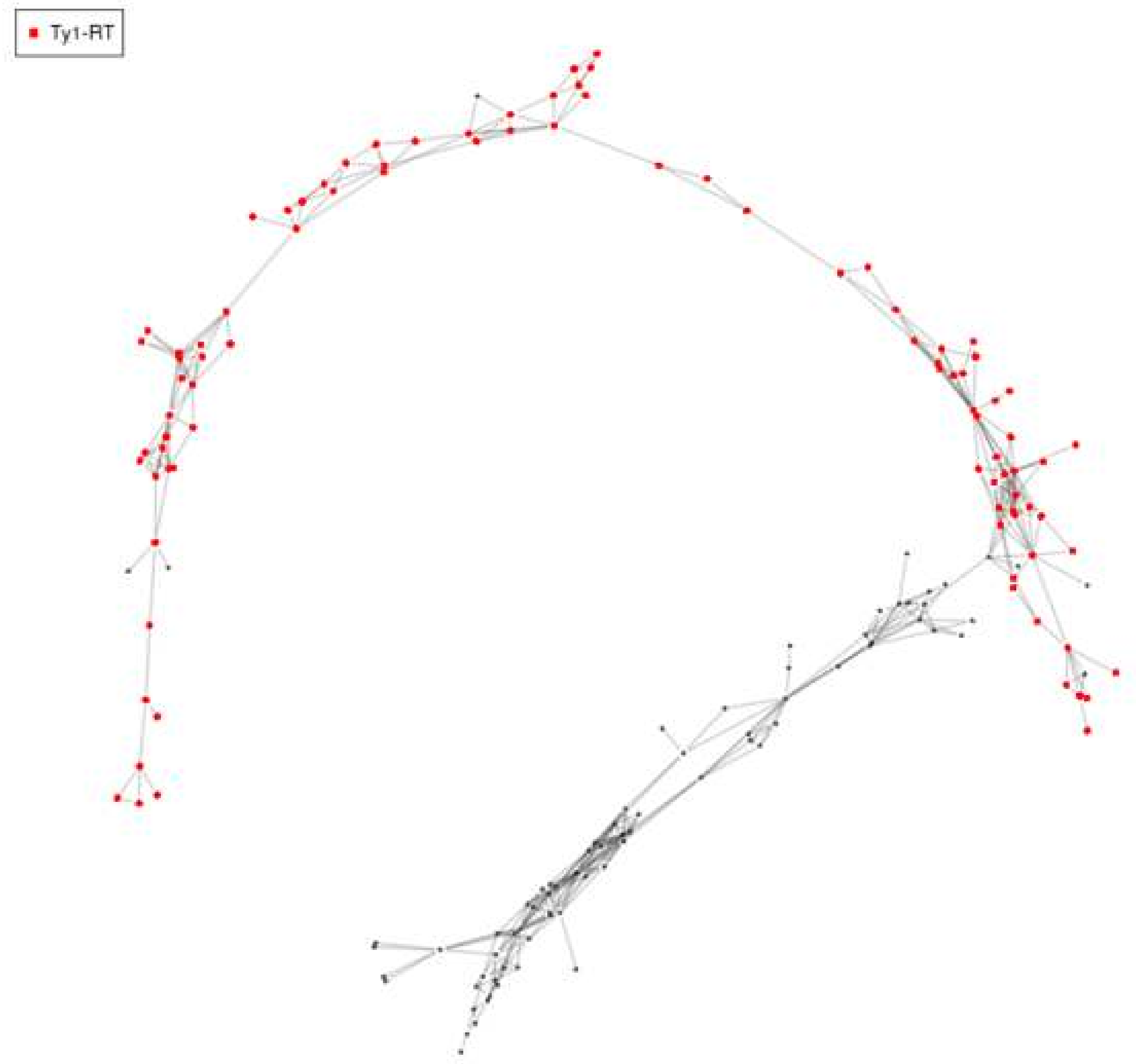

CL131
LTR_Copia
Length of Reads (GP):171 (0.01%)

Tgrandiflorum

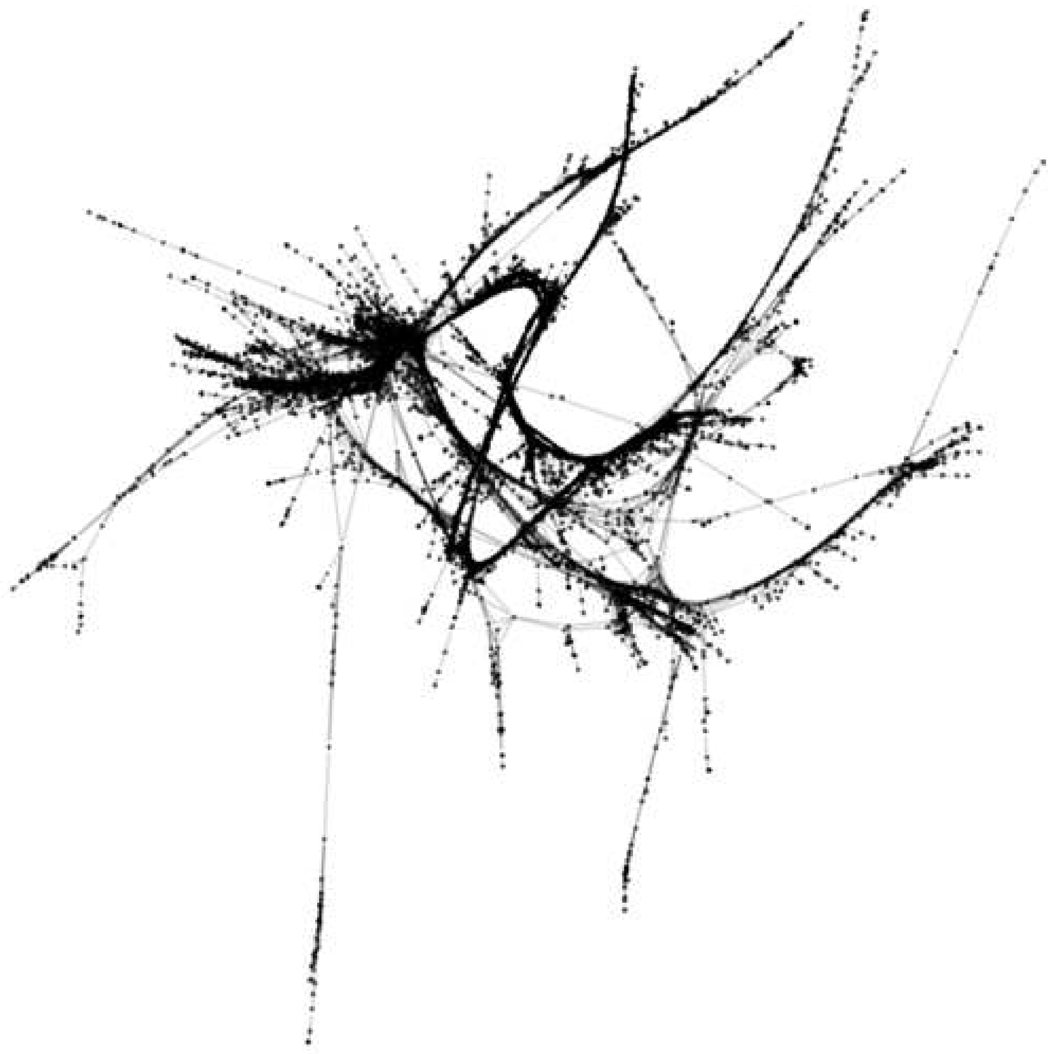

CL131
Low_complexity
Length of Reads (GP):10693 (0.13%)

Tcacao

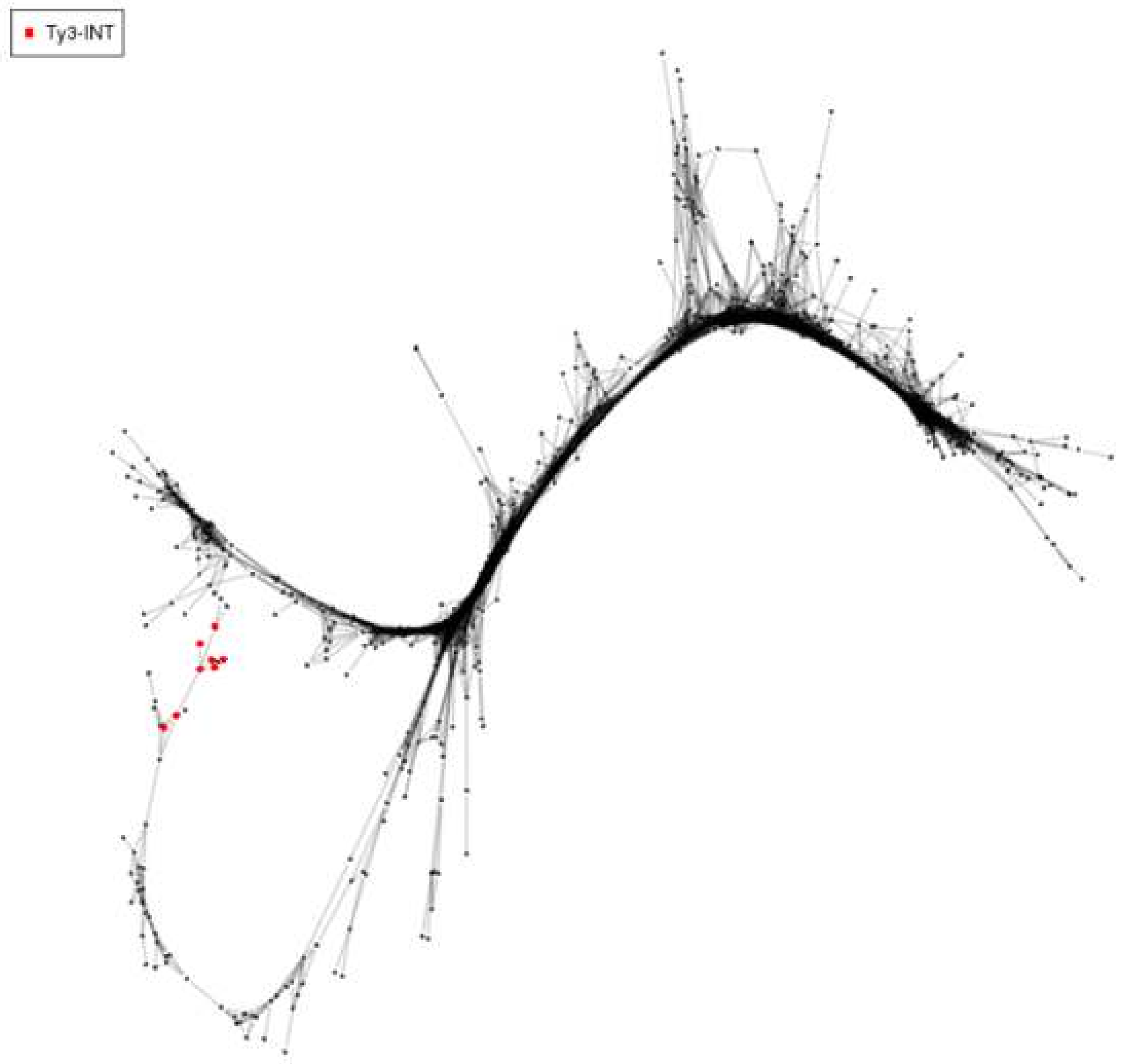

CL131
Low_complexity
Length of Reads (GP):746 (0.04%)

Hbalanensis

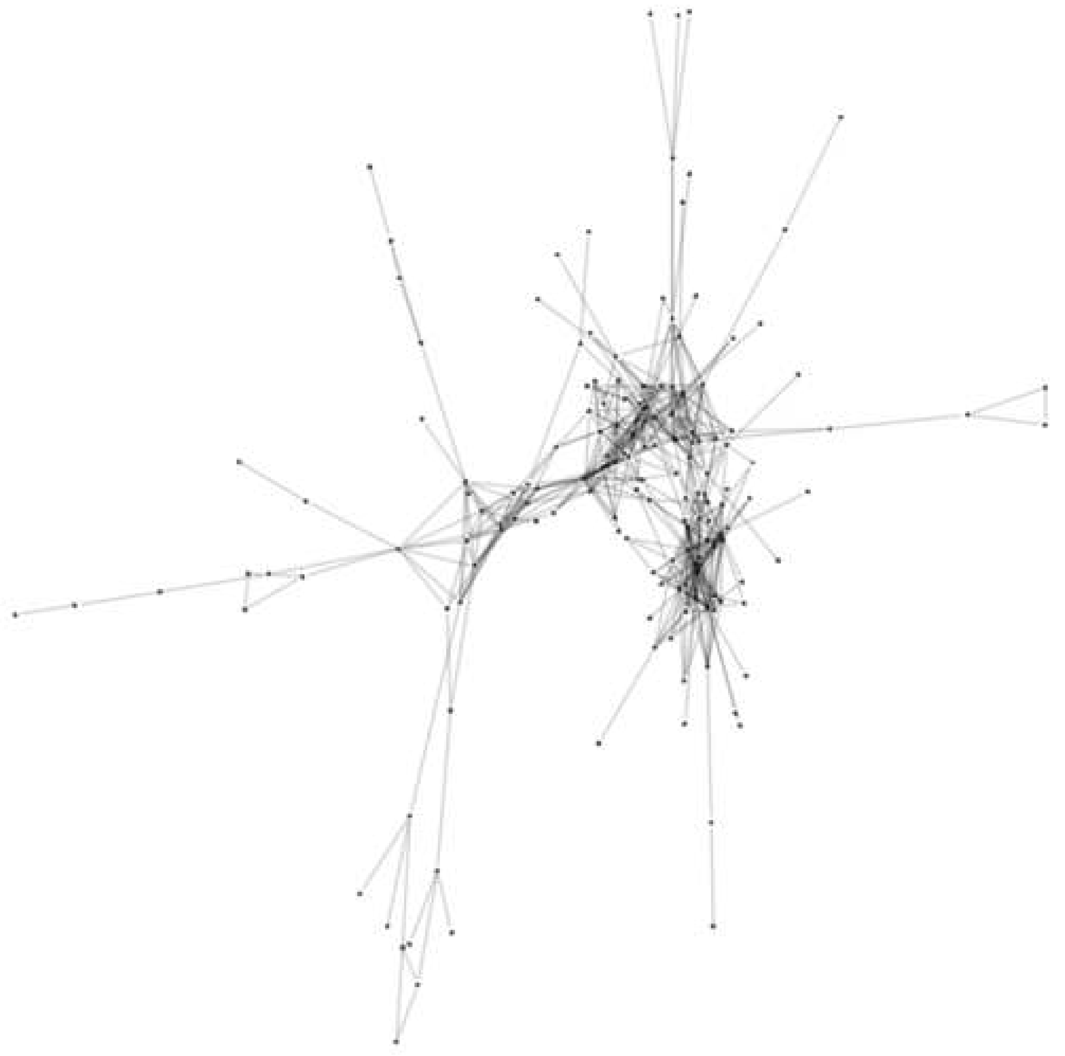

CL132
Low_complexity
Length of Reads (GP):170 (0.01%)

Tgrandiflorum

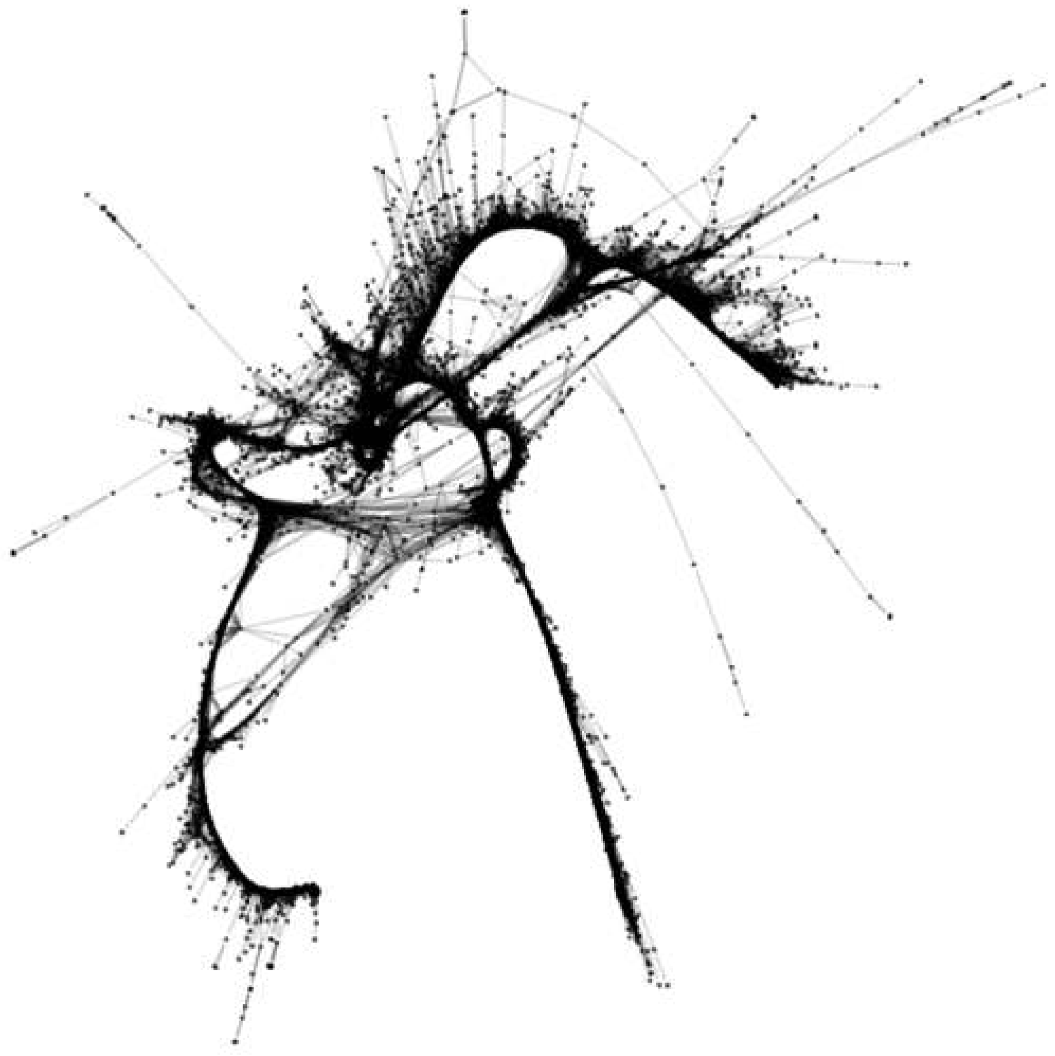

CL132
Low_complexity
Length of Reads (GP):10562 (0.13%)

Tcacao

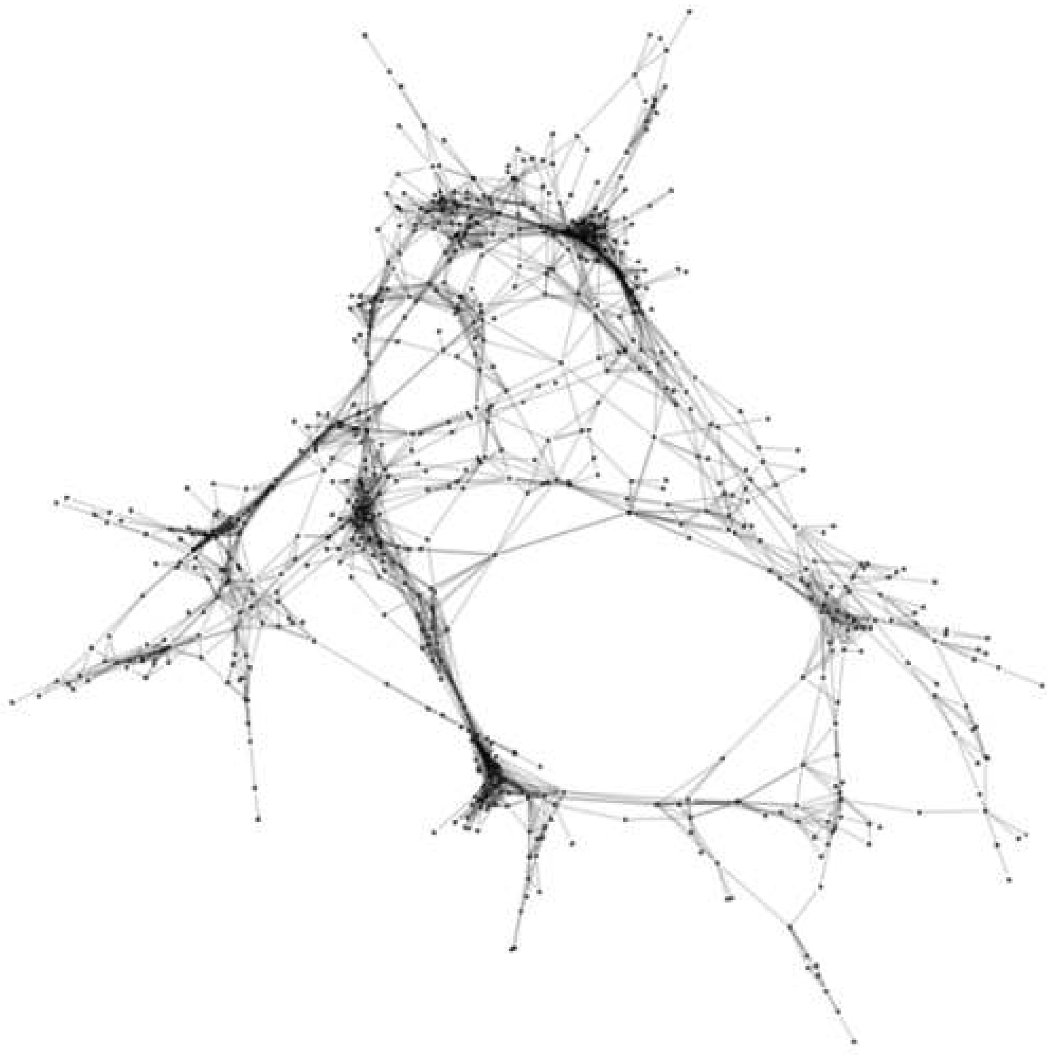

CL132
Low_complexity
Length of Reads (GP):735 (0.04%)

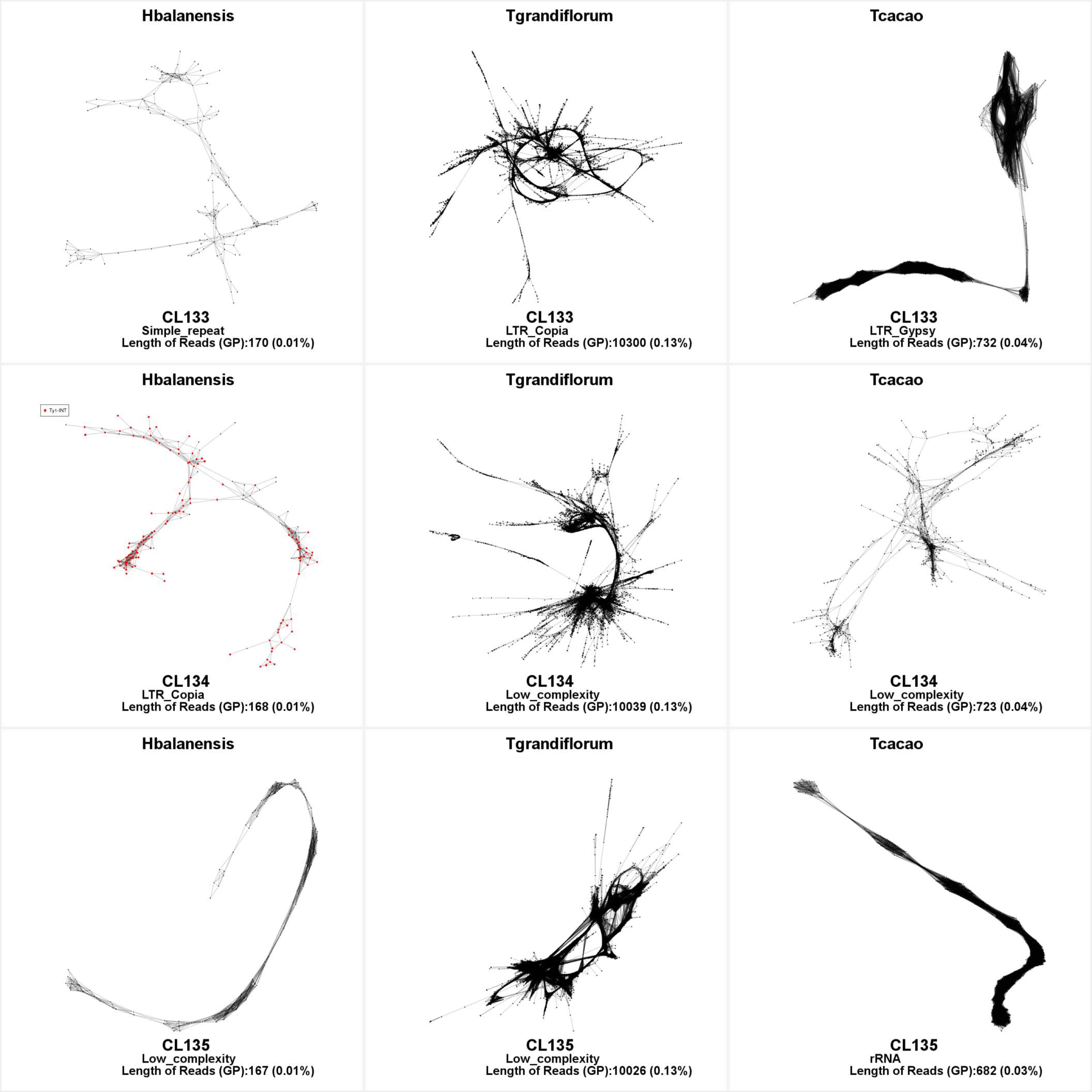
Hbalanensis
CL133
Simple_repeat
Length of Reads (GP):170 (0.01%)
Tgrandiflorum
CL133
LTR_Copia
Length of Reads (GP):10300 (0.13%)
Tcacao
CL133
LTR_Gypsy
Length of Reads (GP):732 (0.04%)
Hbalanensis
Ty1-INT
CL134
LTR_Copia
Length of Reads (GP):168 (0.01%)
Tgrandiflorum
CL134
Low_complexity
Length of Reads (GP):10039 (0.13%)
Tcacao
CL134
Low_complexity
Length of Reads (GP):723 (0.04%)
Hbalanensis
CL135
Low_complexity
Length of Reads (GP):167 (0.01%)
Tgrandiflorum
CL135
Low_complexity
Length of Reads (GP):10026 (0.13%)
Tcacao
CL135
rRNA
Length of Reads (GP):682 (0.03%)

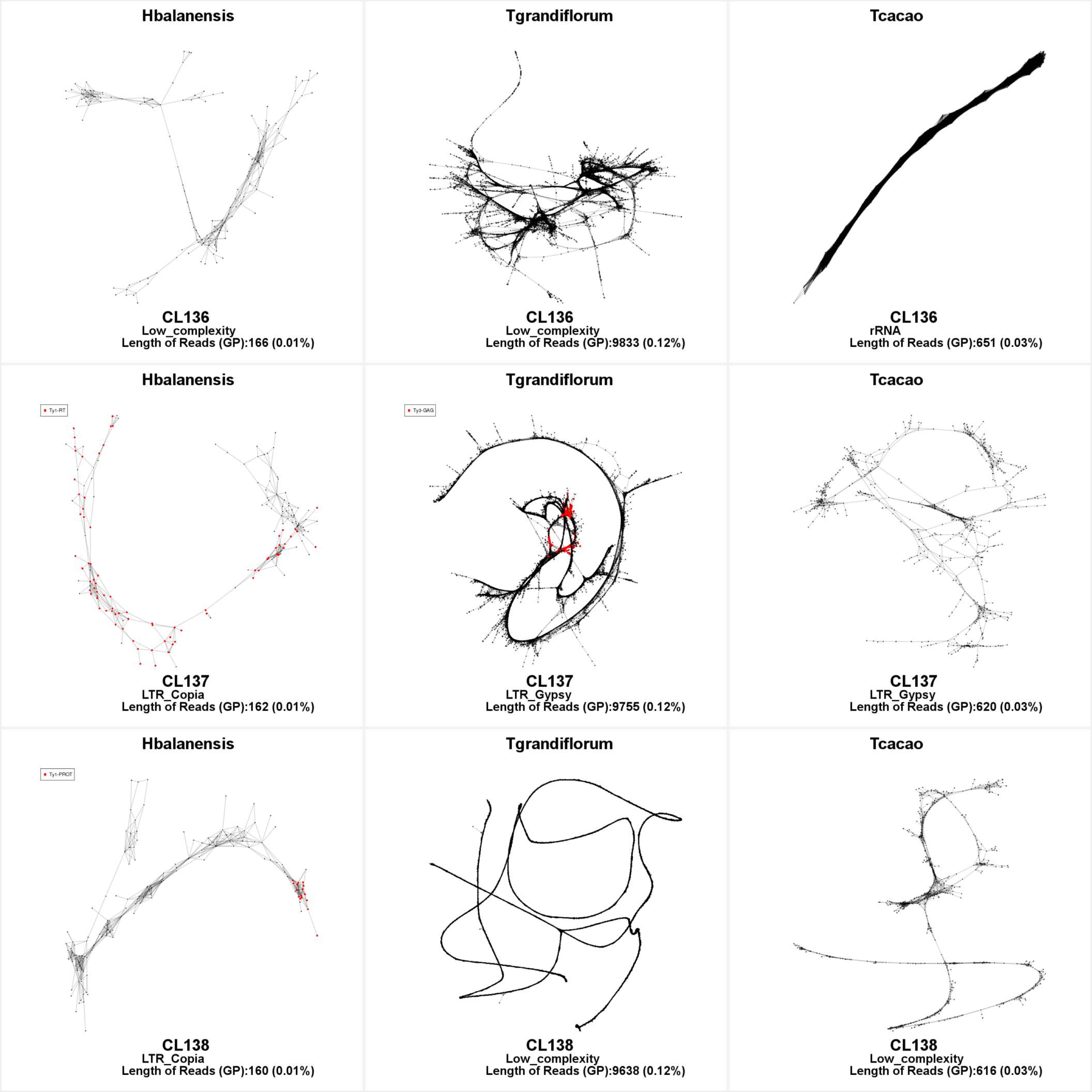
Hbalanensis
CL136
Low_complexity
Length of Reads (GP):166 (0.01%)
Tgrandiflorum
CL136
Low_complexity
Length of Reads (GP):9833 (0.12%)
Tcacao
CL136
rRNA
Length of Reads (GP):651 (0.03%)
Hbalanensis
Ty1-RT
CL137
LTR_Copia
Length of Reads (GP):162 (0.01%)
Tgrandiflorum
Ty3-GAG
CL137
LTR_Gypsy
Length of Reads (GP):9755 (0.12%)
Tcacao
CL137
LTR_Gypsy
Length of Reads (GP):620 (0.03%)
Hbalanensis
Ty1-PROT
CL138
LTR_Copia
Length of Reads (GP):160 (0.01%)
Tgrandiflorum
CL138
Low_complexity
Length of Reads (GP):9638 (0.12%)
Tcacao
CL138
Low_complexity
Length of Reads (GP):616 (0.03%)

Hbalanensis

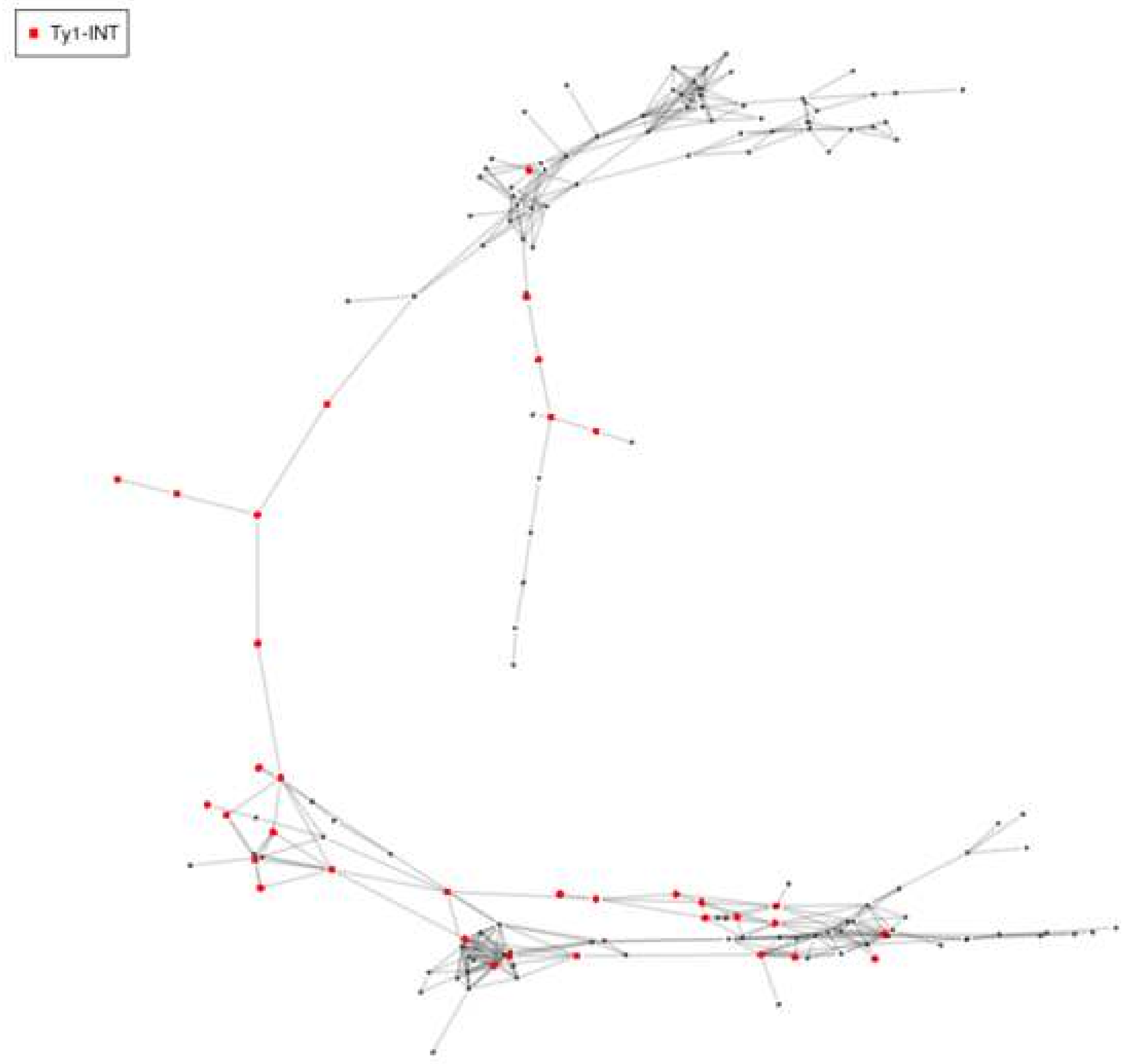

CL139
LTR_Copia
Length of Reads (GP):160 (0.01%)

Tgrandiflorum

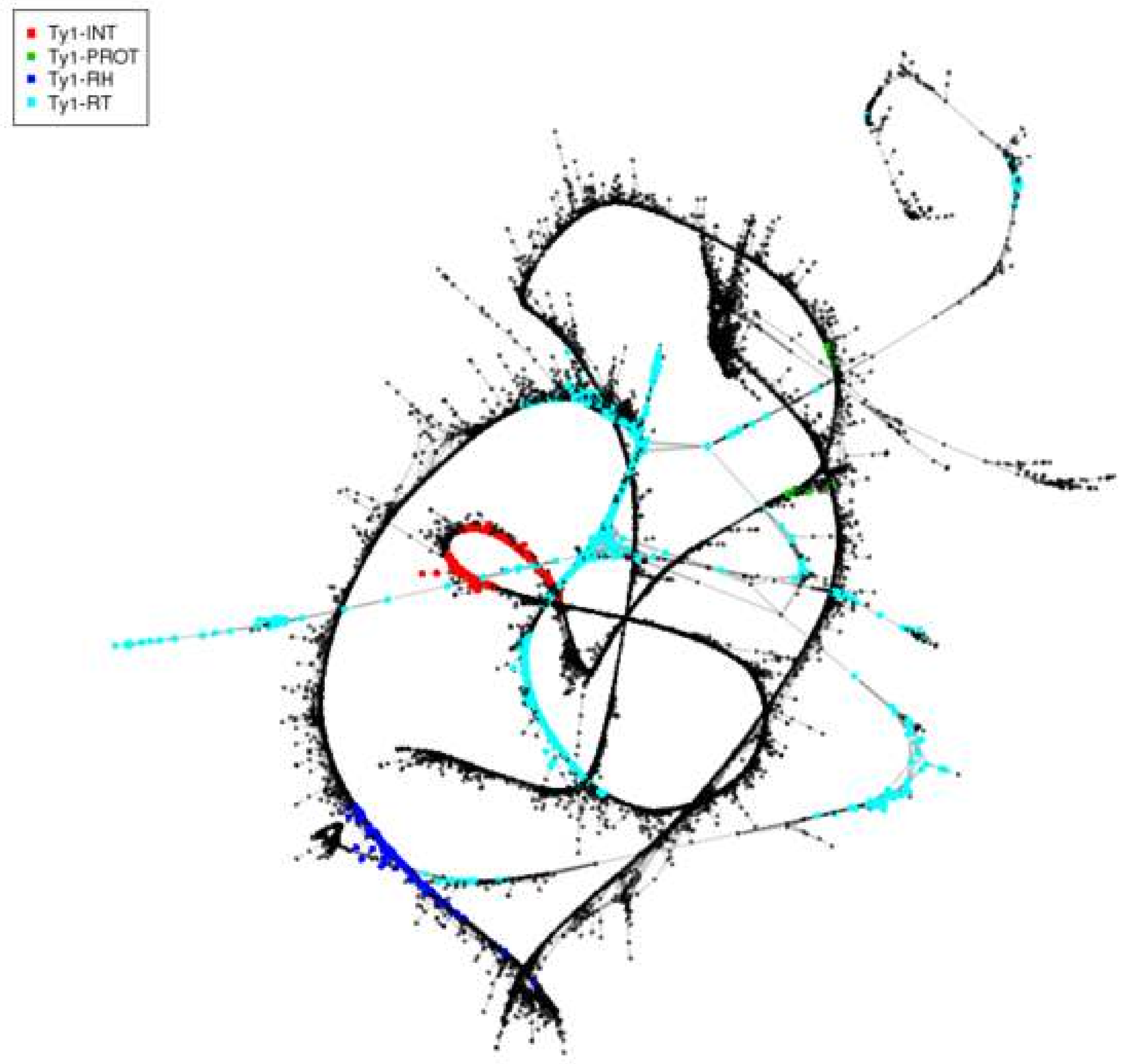

CL139
LTR_Copia
Length of Reads (GP):9273 (0.12%)

Tcacao

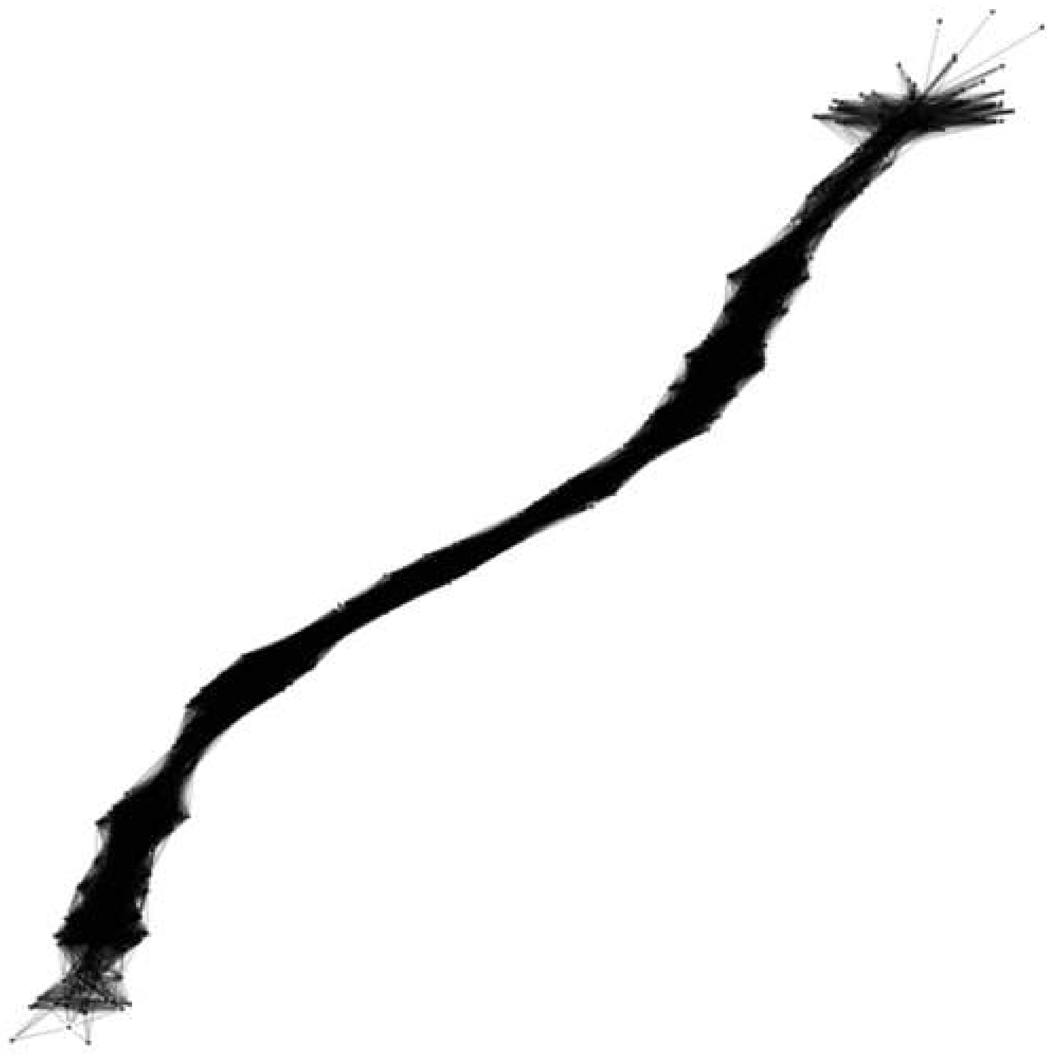

CL139
Low_complexity
Length of Reads (GP):613 (0.03%)

Hbalanensis

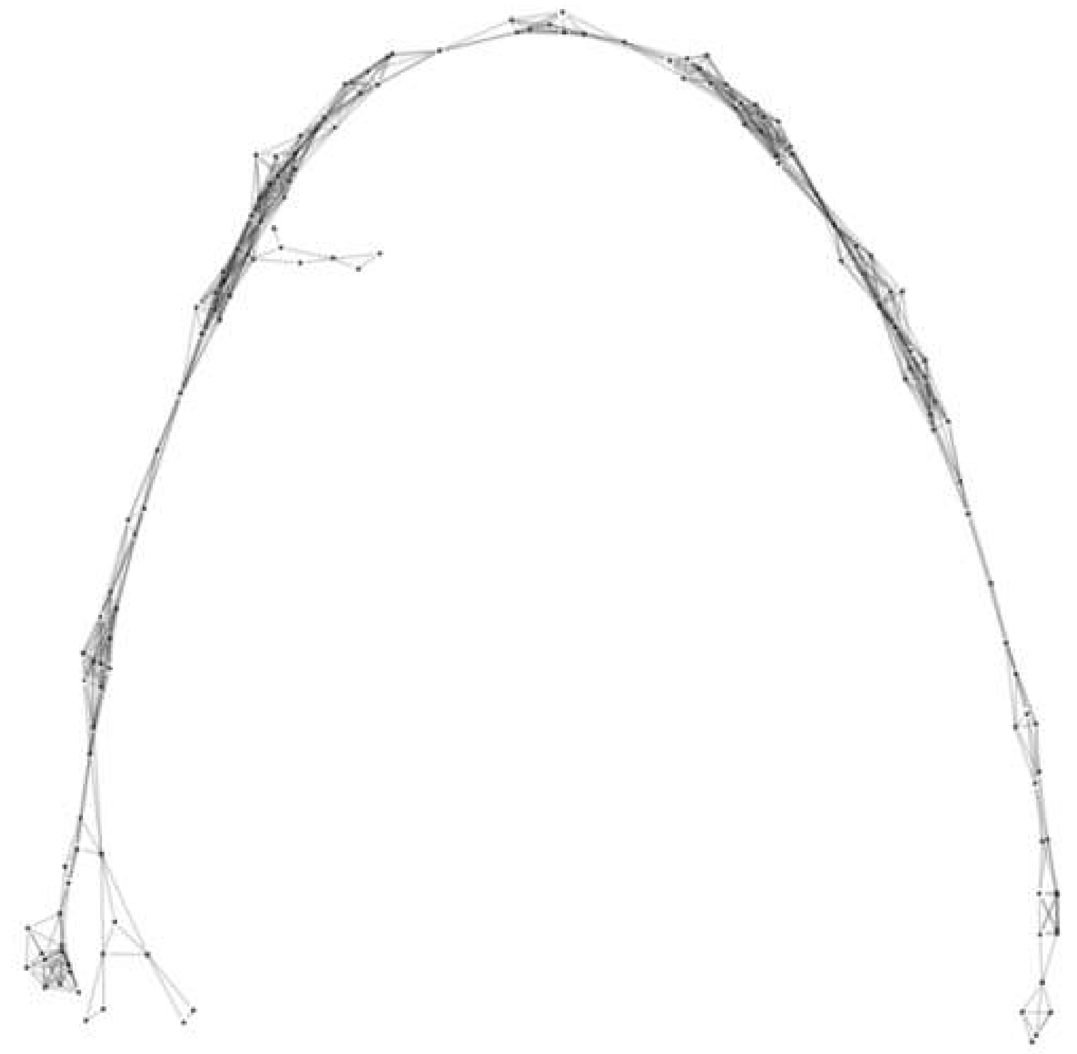

CL140
DNA_hAT_Ac
Length of Reads (GP):151 (0.01%)

Tgrandiflorum

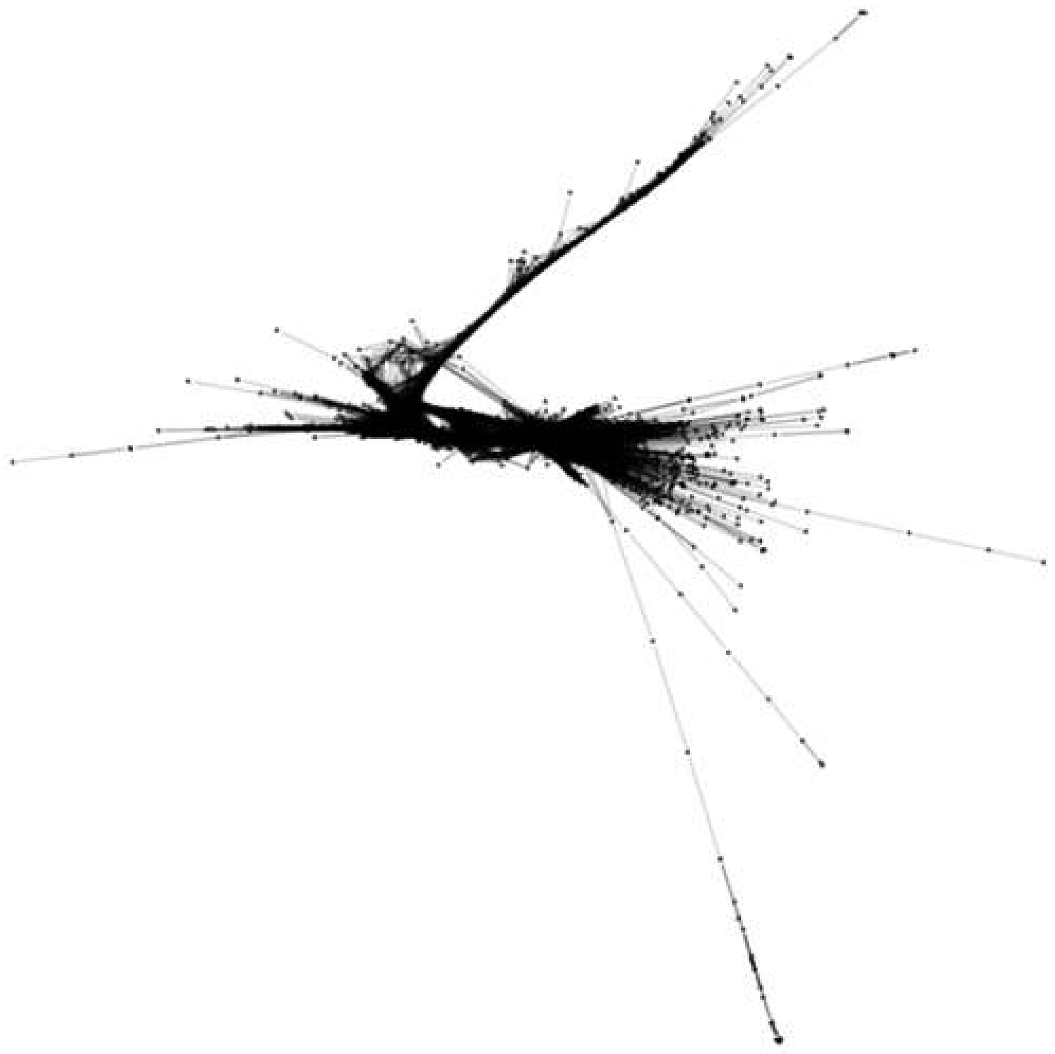

CL140
LTR_Copia
Length of Reads (GP):9241 (0.12%)

Tcacao

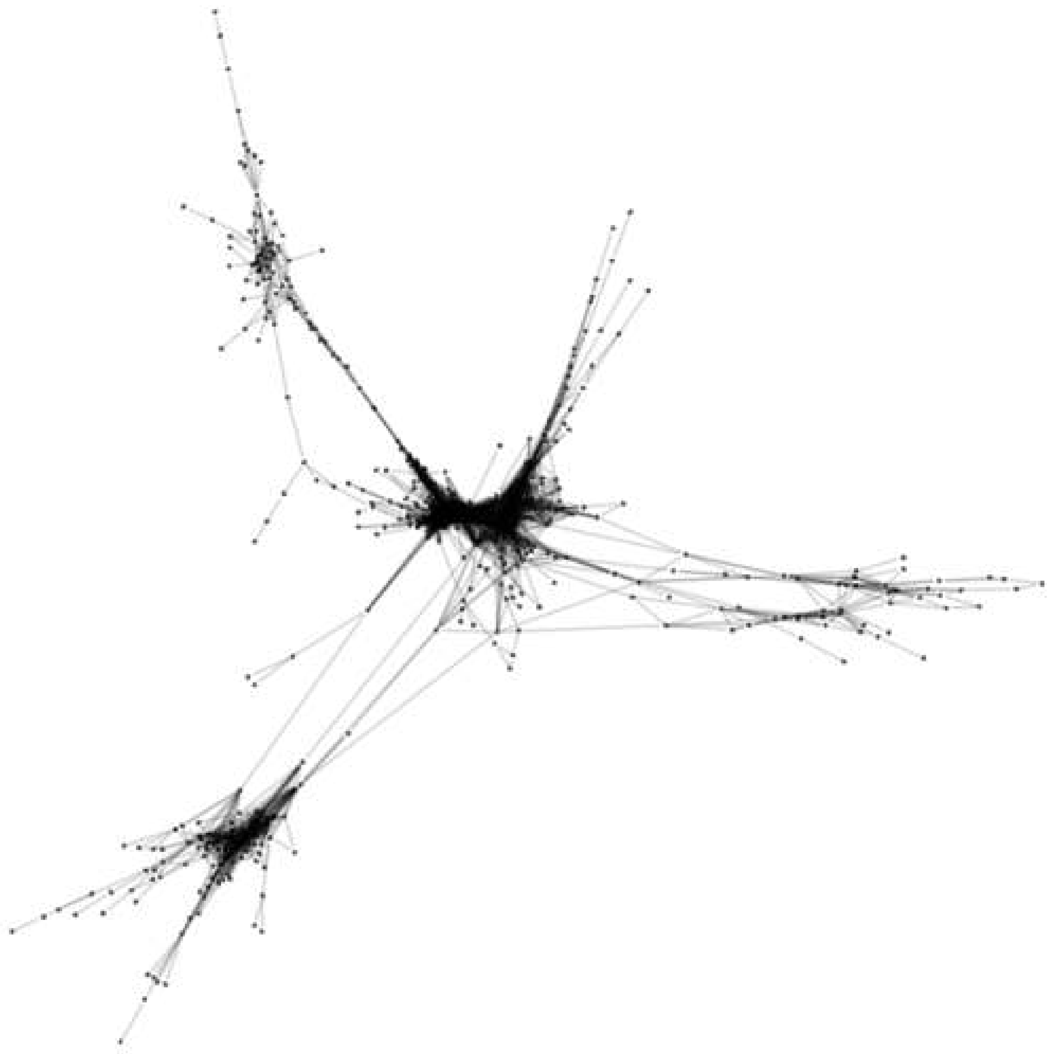

CL140
LTR_Gypsy
Length of Reads (GP):612 (0.03%)

Hbalanensis

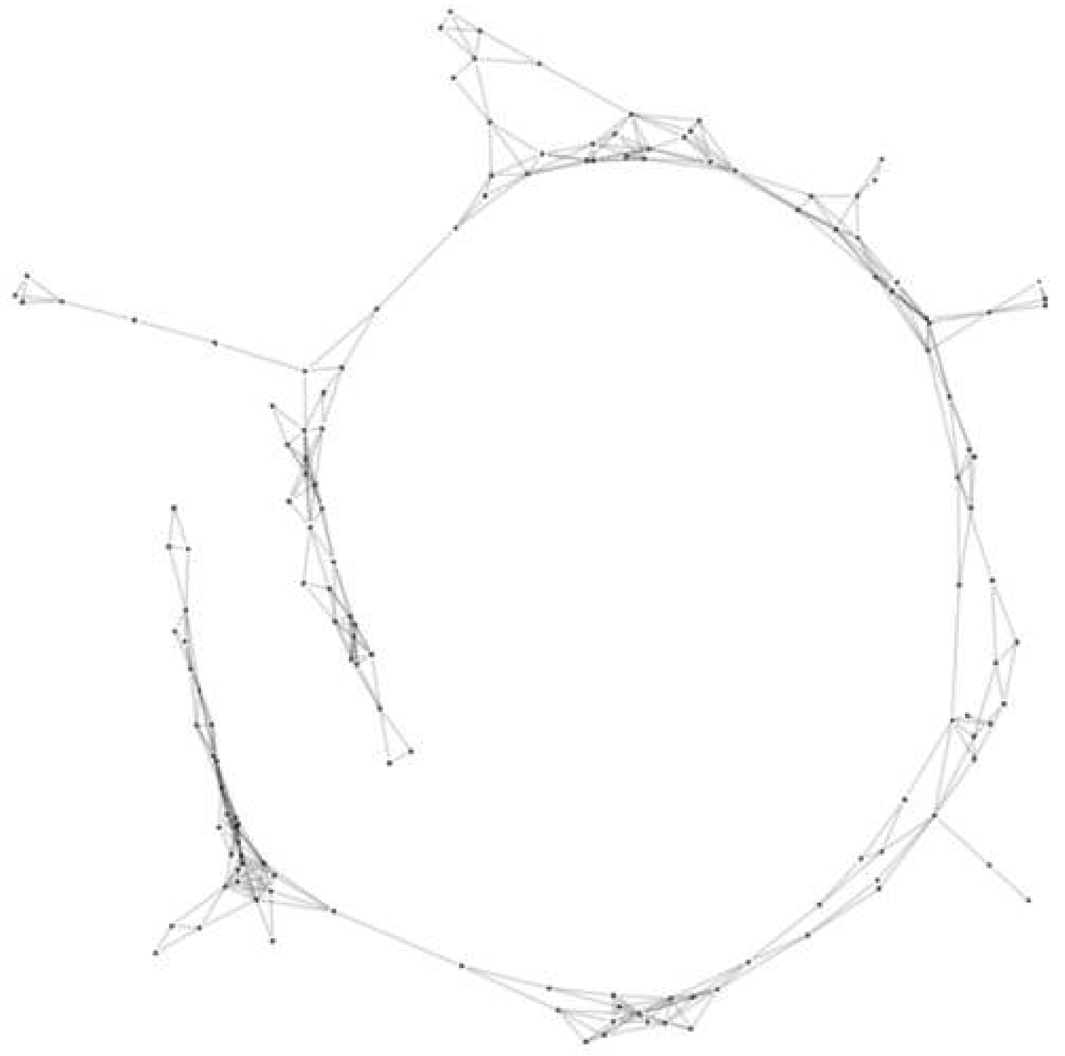

CL141
Low_complexity
Length of Reads (GP):150 (0.01%)

Tgrandiflorum

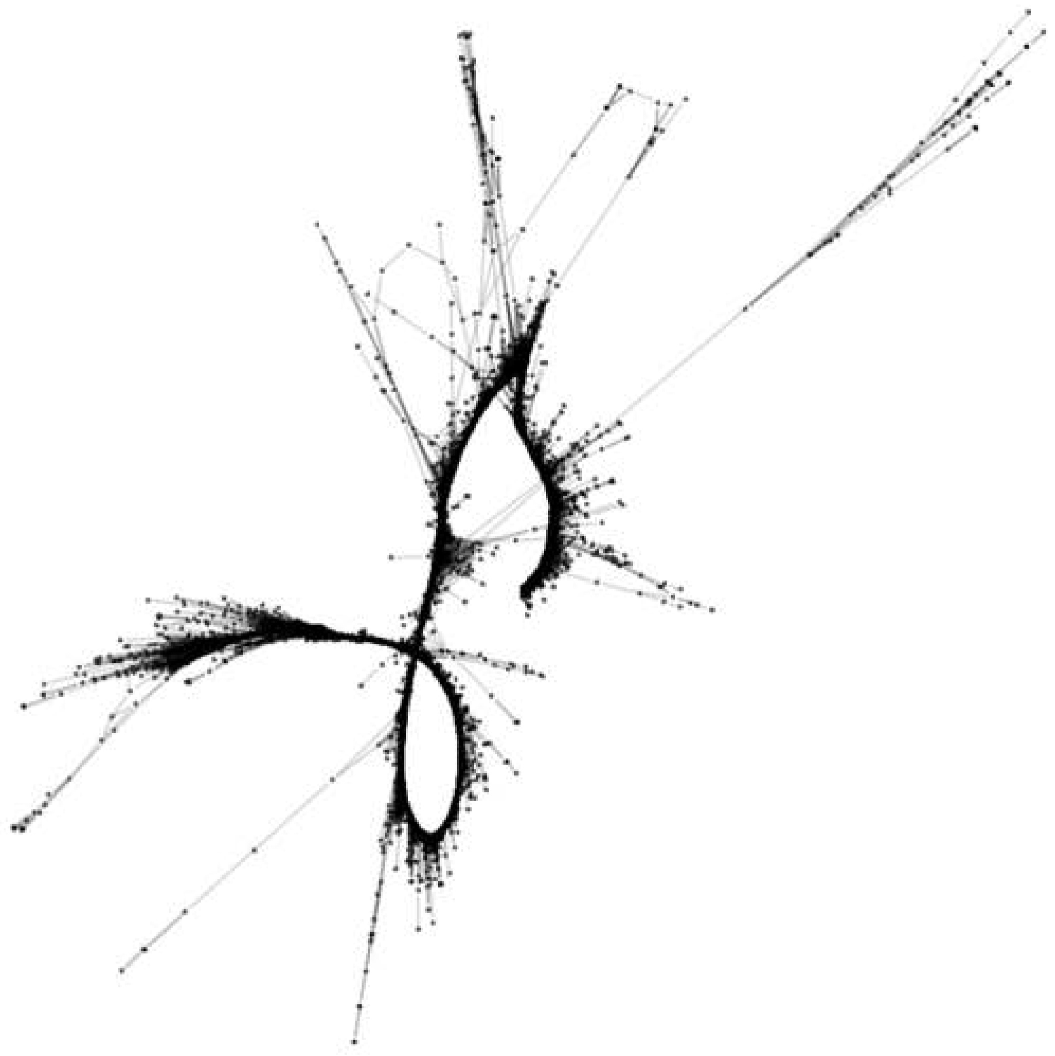

CL141
Low_complexity
Length of Reads (GP):9113 (0.11%)

Tcacao

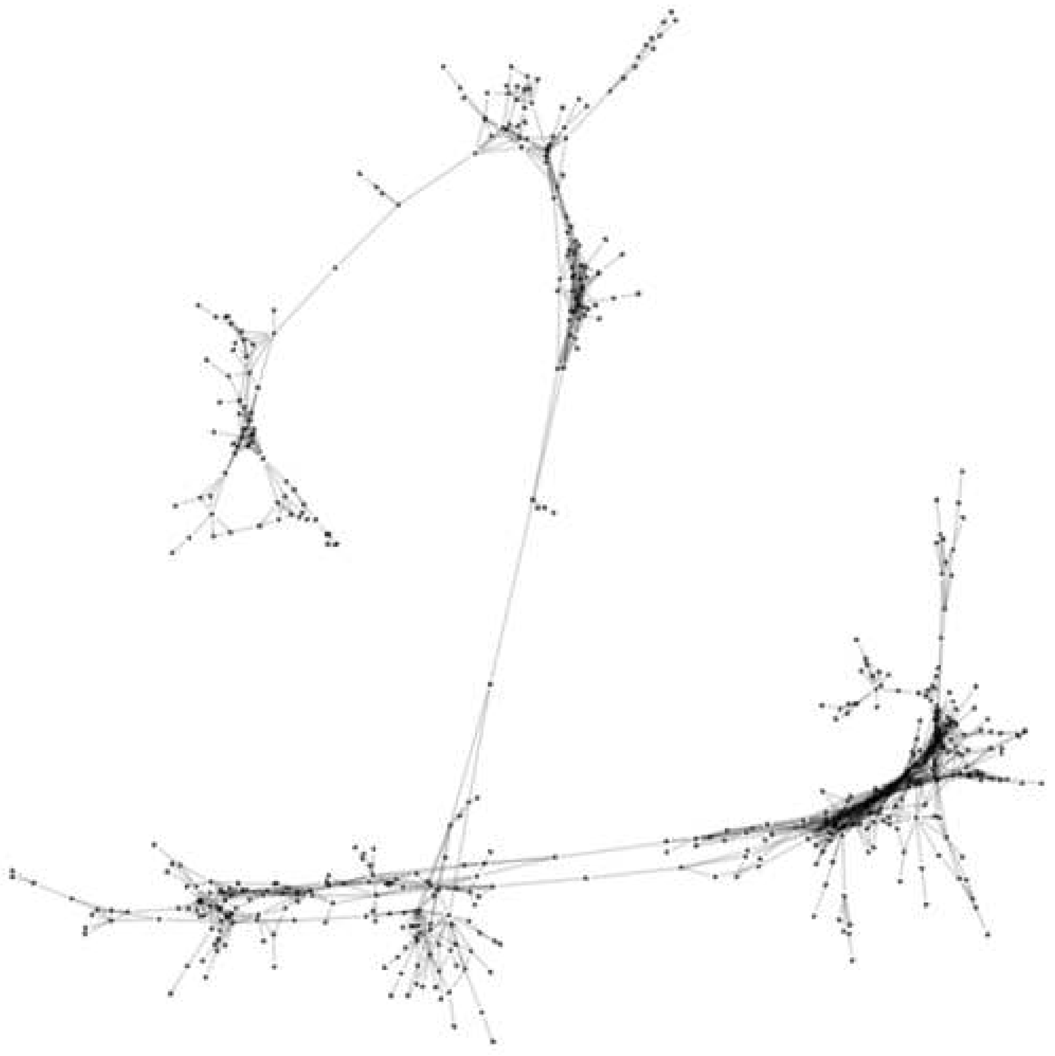

CL141
Low_complexity
Length of Reads (GP):582 (0.03%)

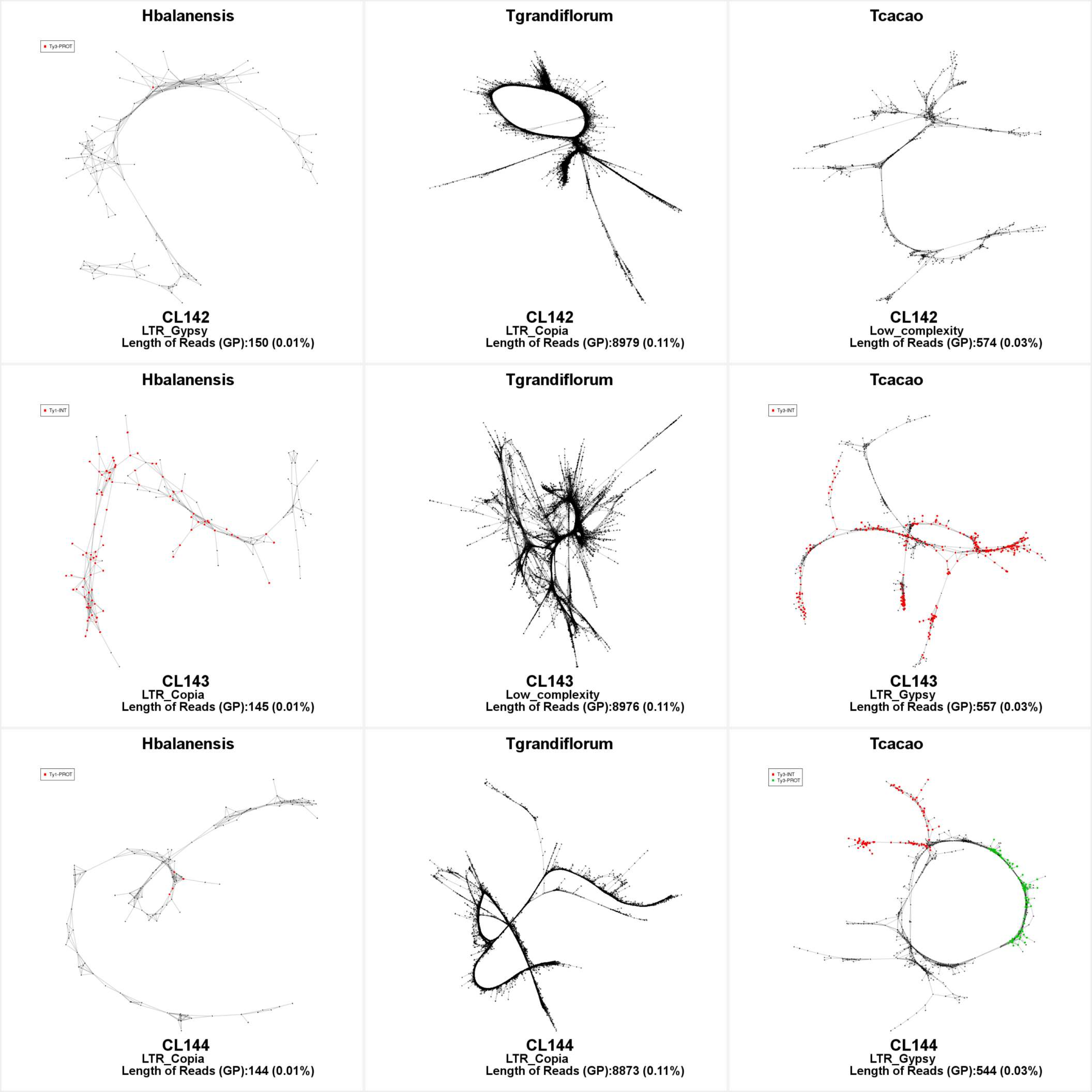

Hbalanensis
Ty3-PROT
CL142
LTR_Gypsy
Length of Reads (GP):150 (0.01%)
Tgrandiflorum
CL142
LTR_Copia
Length of Reads (GP):8979 (0.11%)
Tcacao
CL142
Low_complexity
Length of Reads (GP):574 (0.03%)
Hbalanensis
Ty1-INT
CL143
LTR_Copia
Length of Reads (GP):145 (0.01%)
Tgrandiflorum
CL143
Low_complexity
Length of Reads (GP):8976 (0.11%)
Tcacao
Ty3-INT
CL143
LTR_Gypsy
Length of Reads (GP):557 (0.03%)
Hbalanensis
Ty1-PROT
CL144
LTR_Copia
Length of Reads (GP):144 (0.01%)
Tgrandiflorum
CL144
LTR_Copia
Length of Reads (GP):8873 (0.11%)
Tcacao
Ty3-INT
Ty3-PROT
CL144
LTR_Gypsy
Length of Reads (GP):544 (0.03%)

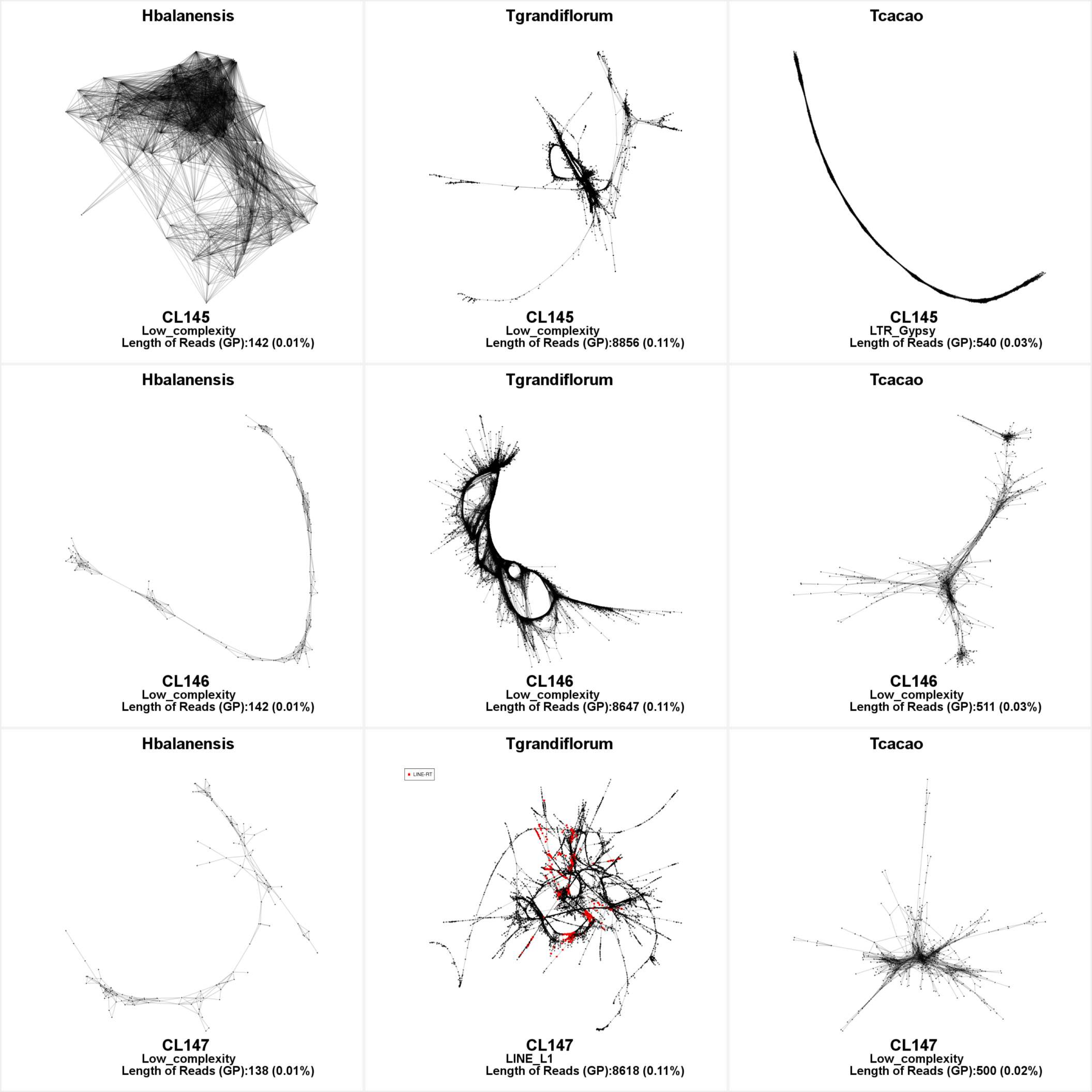
Hbalanensis
Tgrandiflorum
Tcacao
CL145
Low_complexity
Length of Reads (GP):142 (0.01%)
CL145
Low_complexity
Length of Reads (GP):8856 (0.11%)
CL145
LTR_Gypsy
Length of Reads (GP):540 (0.03%)
Hbalanensis
Tgrandiflorum
Tcacao
CL146
Low_complexity
Length of Reads (GP):142 (0.01%)
CL146
Low_complexity
Length of Reads (GP):8647 (0.11%)
CL146
Low_complexity
Length of Reads (GP):511 (0.03%)
Hbalanensis
Tgrandiflorum
Tcacao
LINE-RT
CL147
Low_complexity
Length of Reads (GP):138 (0.01%)
CL147
LINE_L1
Length of Reads (GP):8618 (0.11%)
CL147
Low_complexity
Length of Reads (GP):500 (0.02%)

Hbalanensis

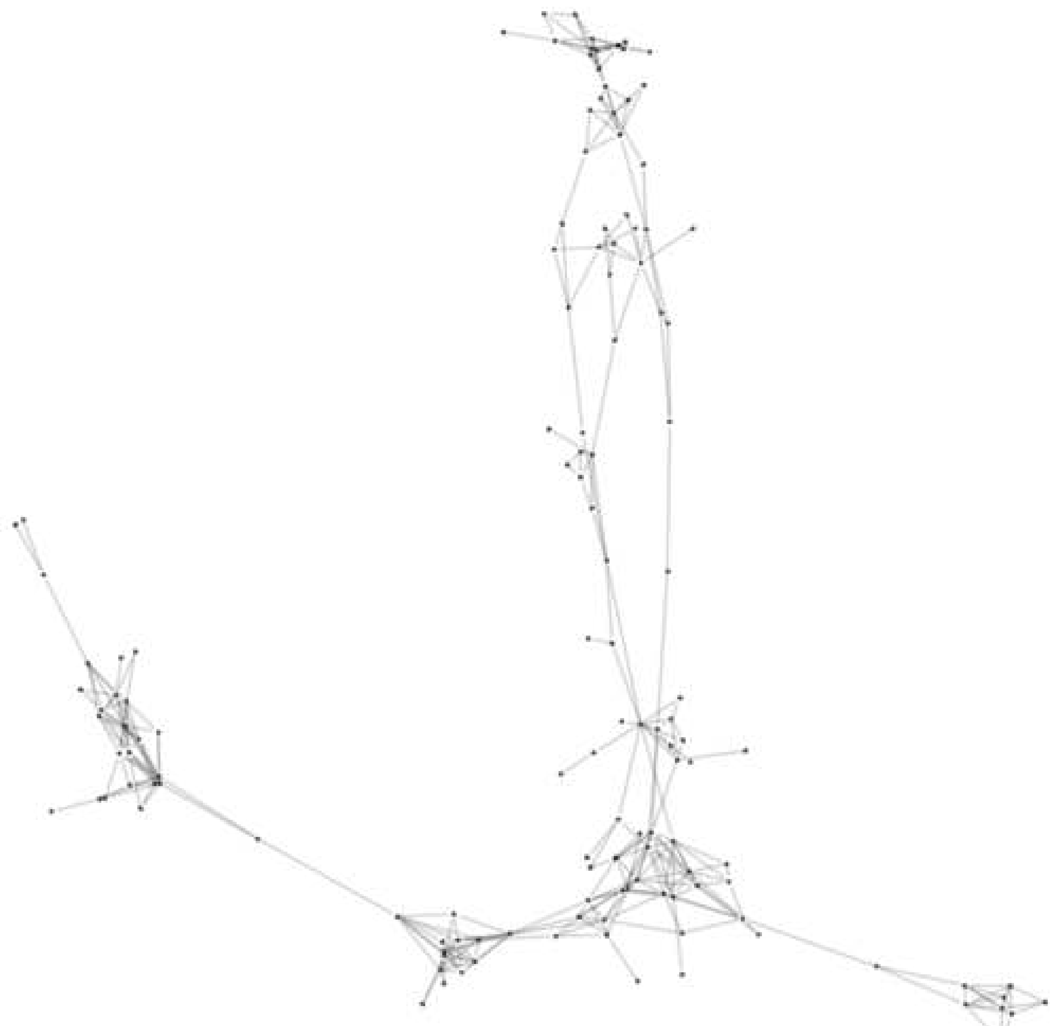

CL148
LTR_Copia
Length of Reads (GP):137 (0.01%)

Tgrandiflorum

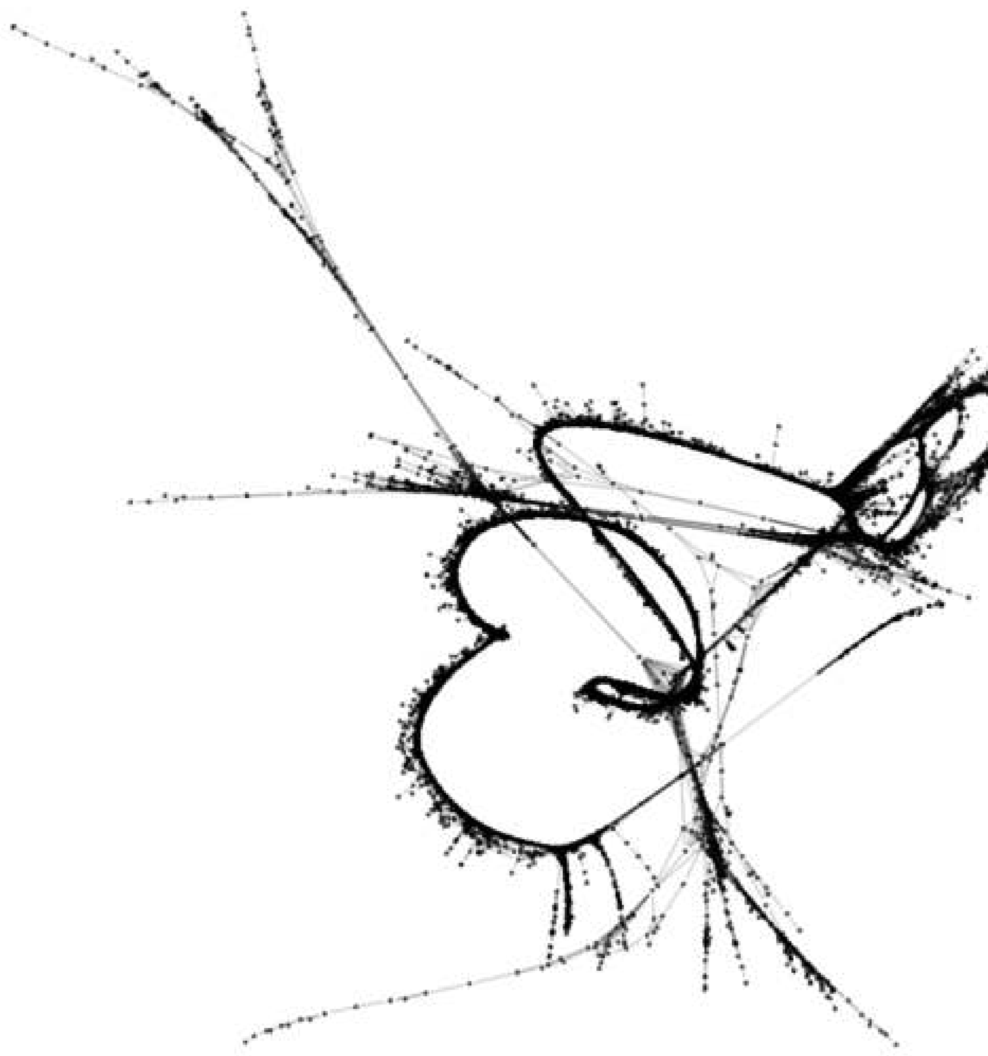

CL148
Low_complexity
Length of Reads (GP):8382 (0.11%)

Tcacao

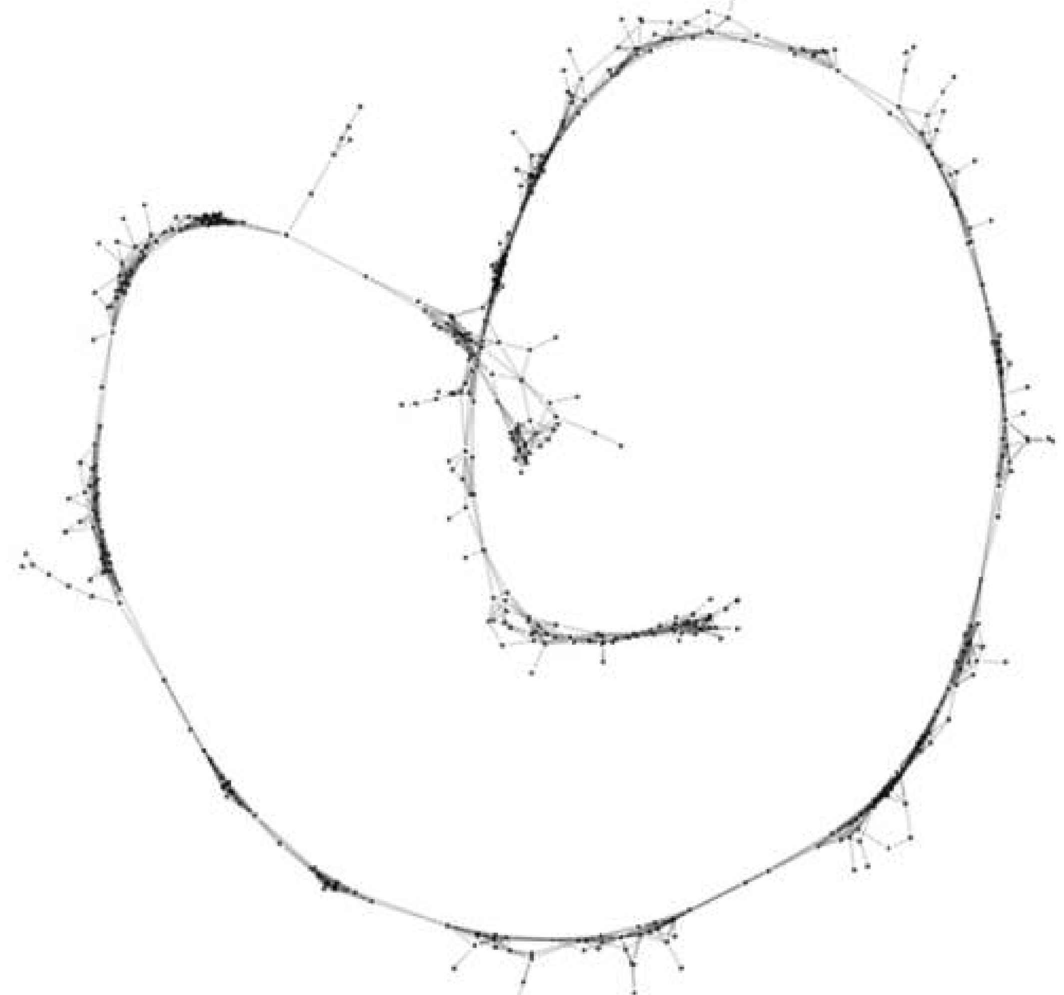

CL148
ARTEFACT
Length of Reads (GP):491 (0.02%)

Hbalanensis

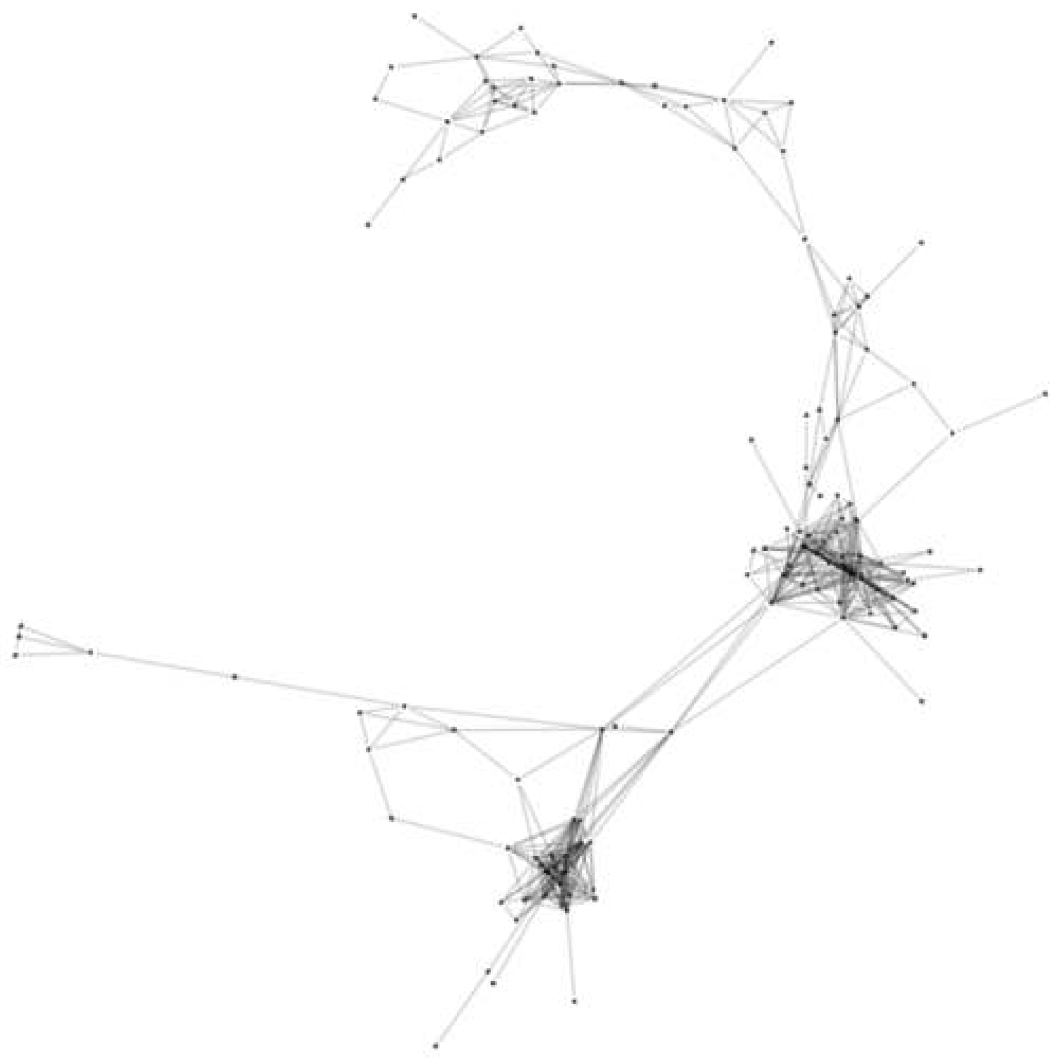

CL149
Low_complexity
Length of Reads (GP):134 (0.01%)

Tgrandiflorum

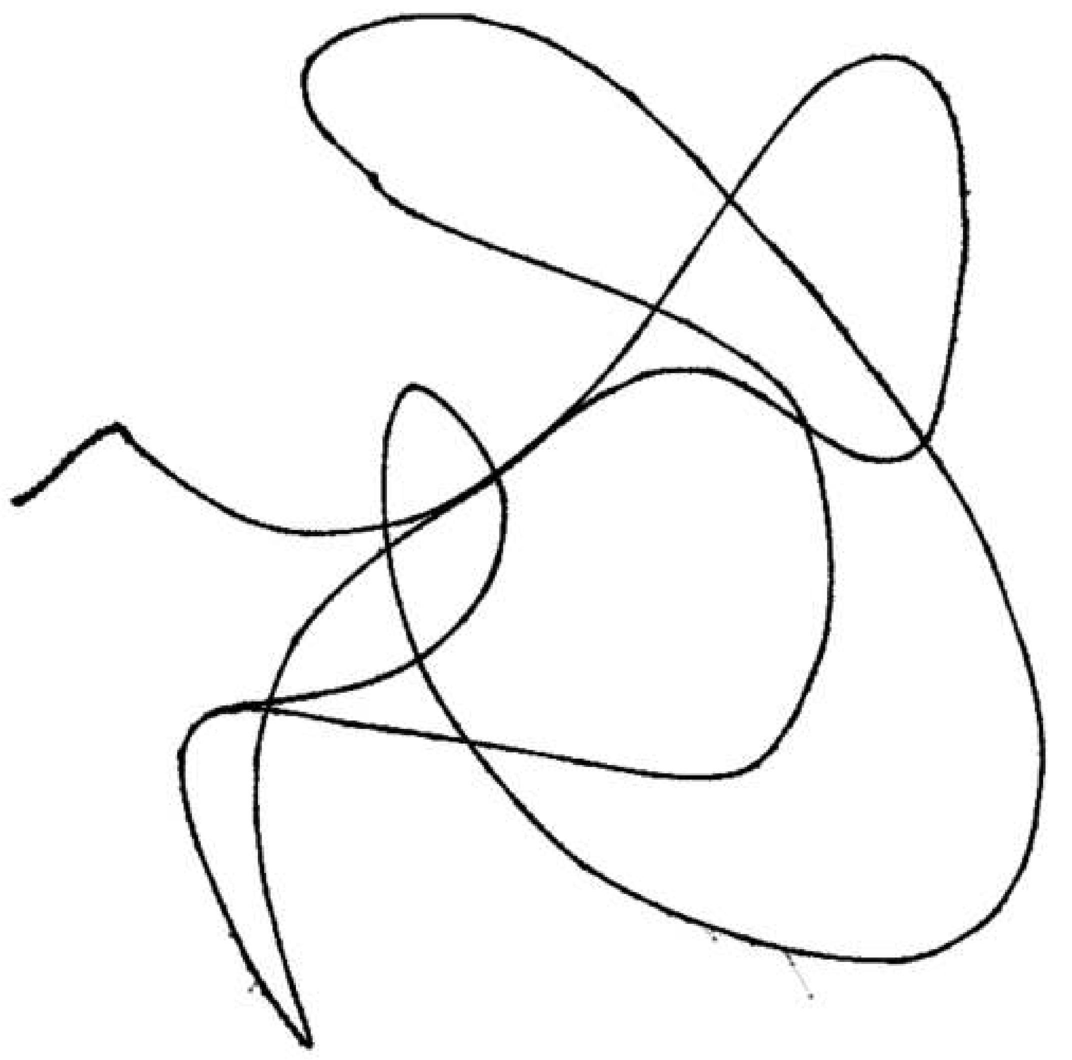

CL149
LTR_Copia
Length of Reads (GP):8309 (0.1%)

Tcacao

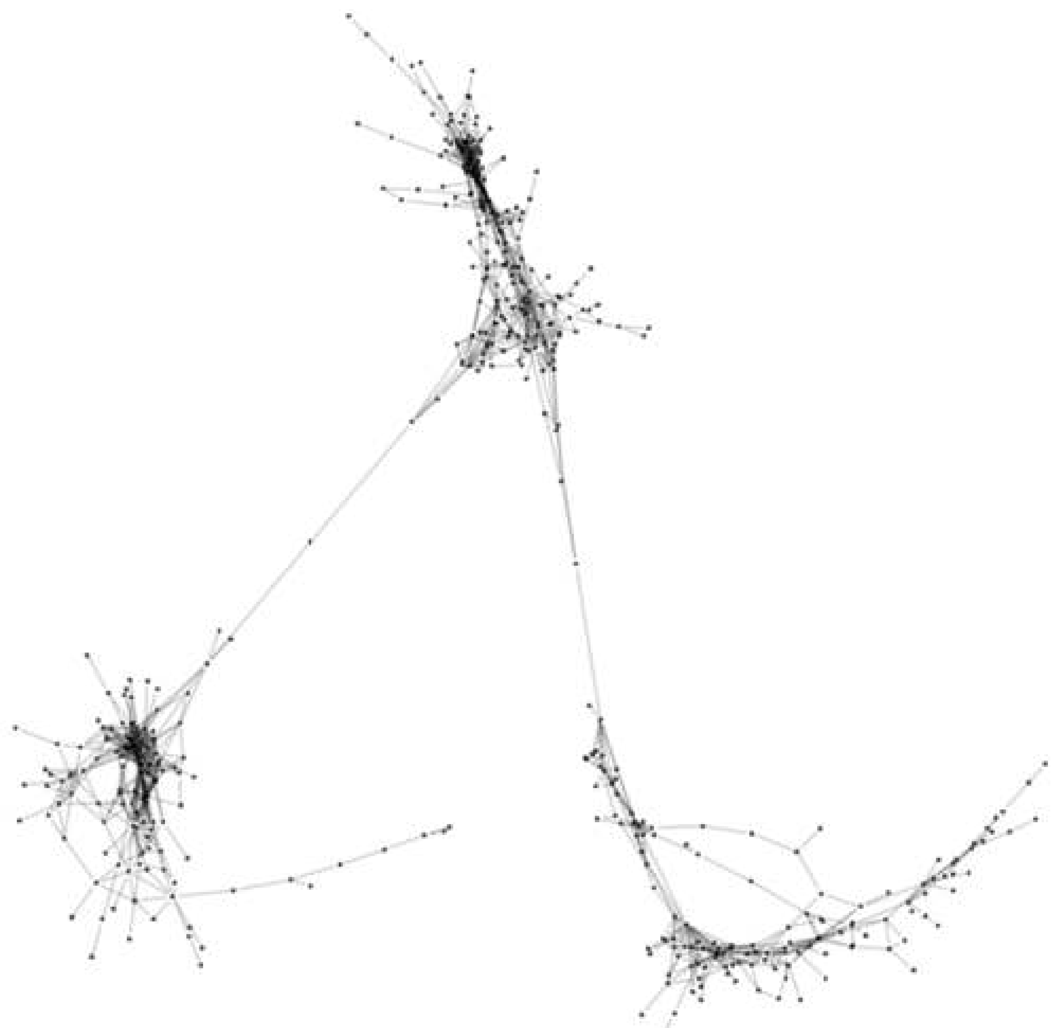

CL149
Low_complexity
Length of Reads (GP):474 (0.02%)

Tgrandiflorum

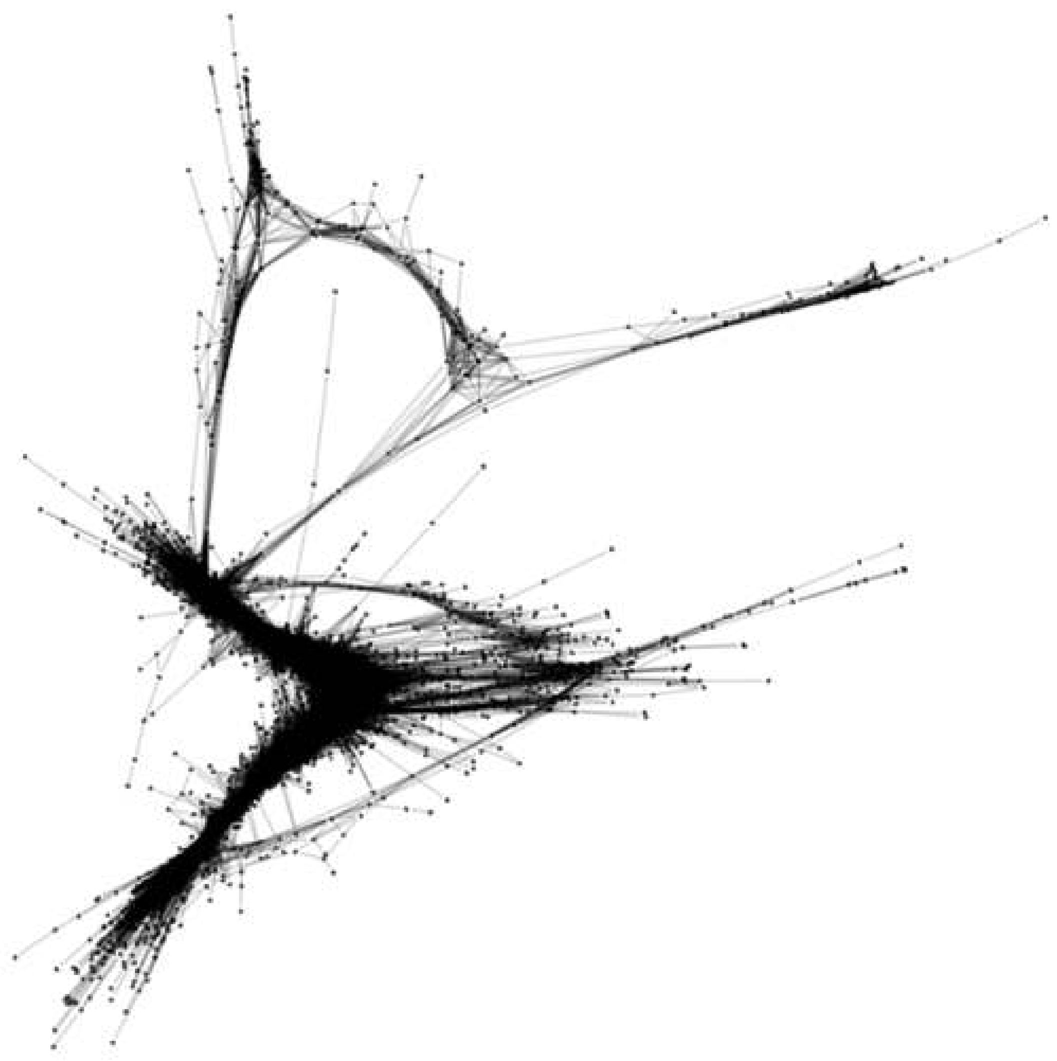

CL150
LTR_Gypsy
Length of Reads (GP):8113 (0.1%)

Tcacao

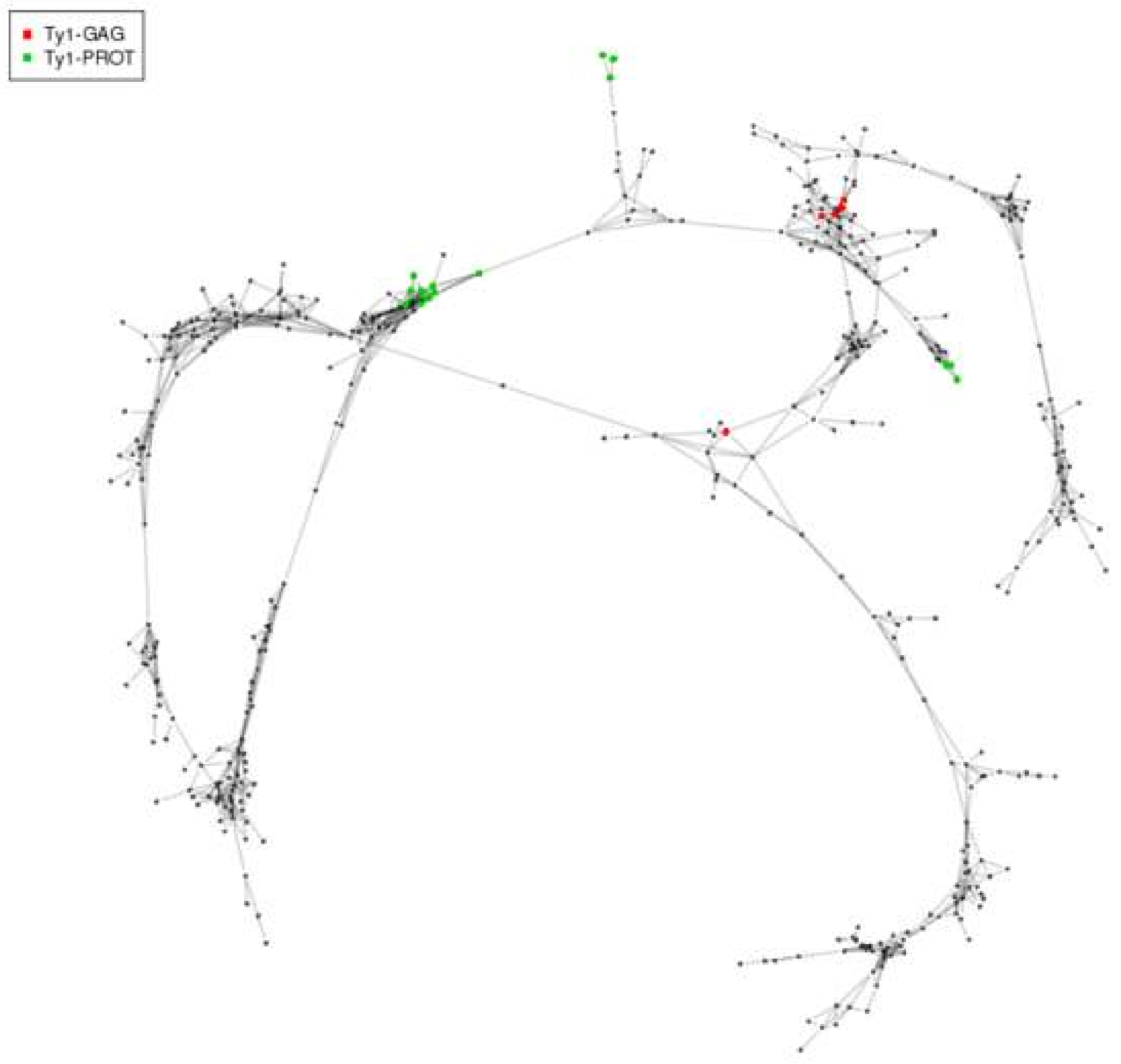

CL150
LTR_Copia
Length of Reads (GP):444 (0.02%)

Tgrandiflorum

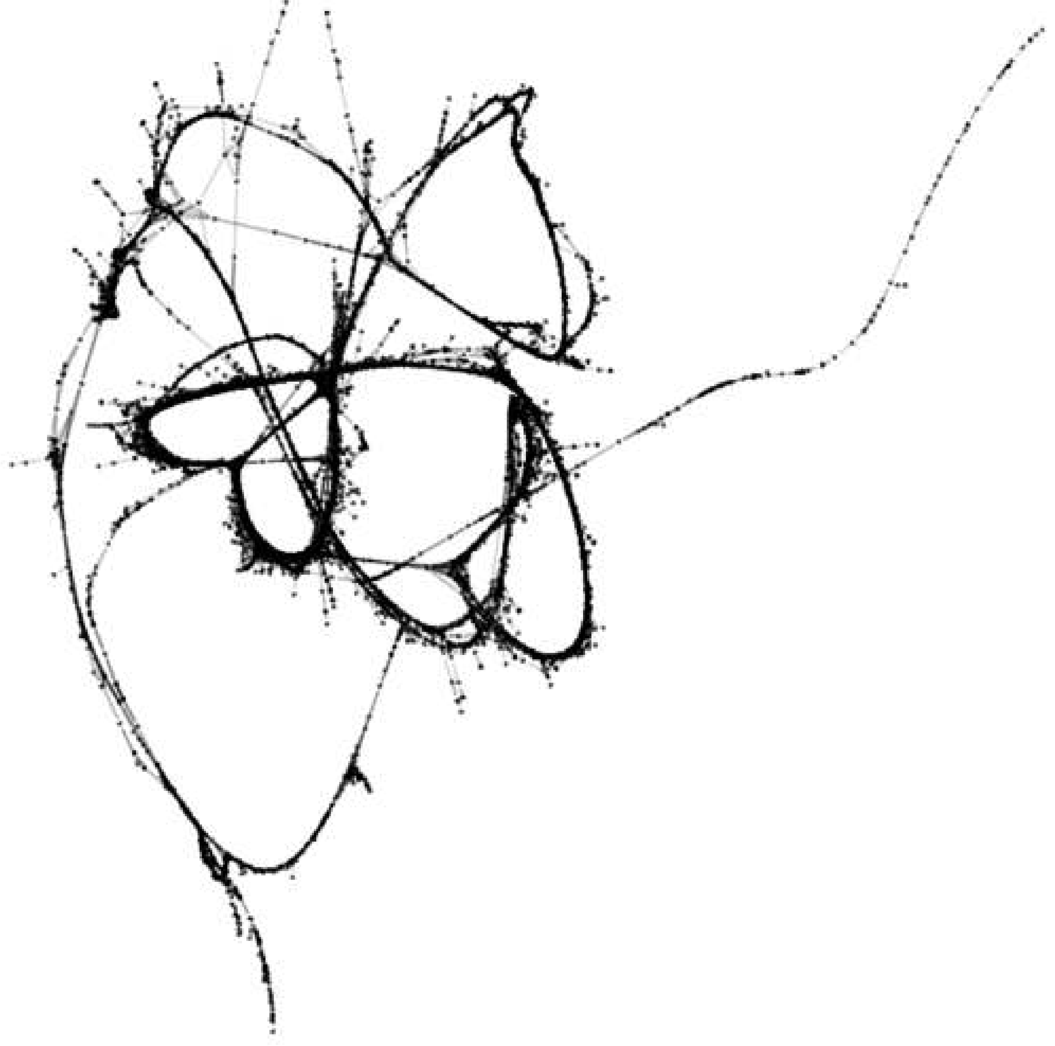

CL151
Low_complexity
Length of Reads (GP):8104 (0.1%)

Tcacao

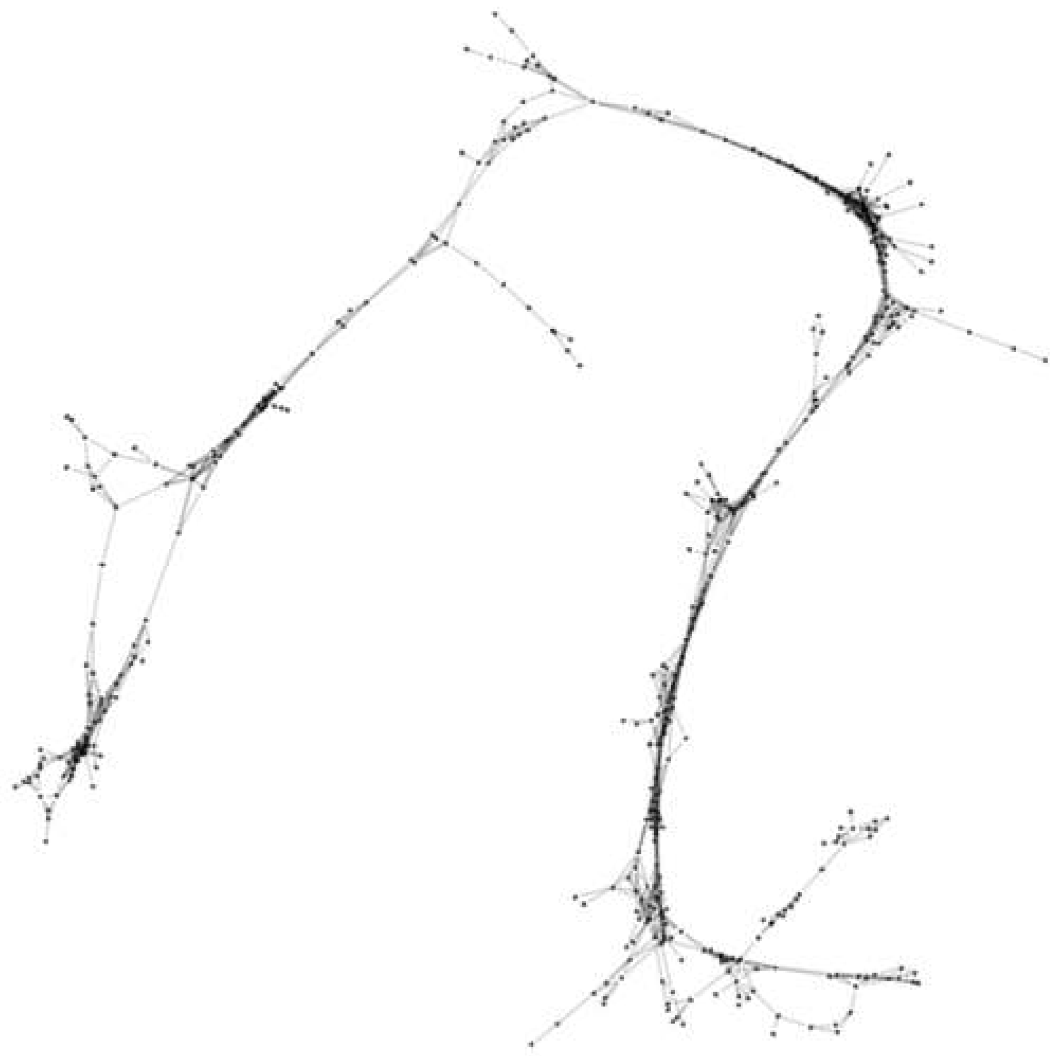

CL151
Low_complexity
Length of Reads (GP):441 (0.02%)

Tgrandiflorum

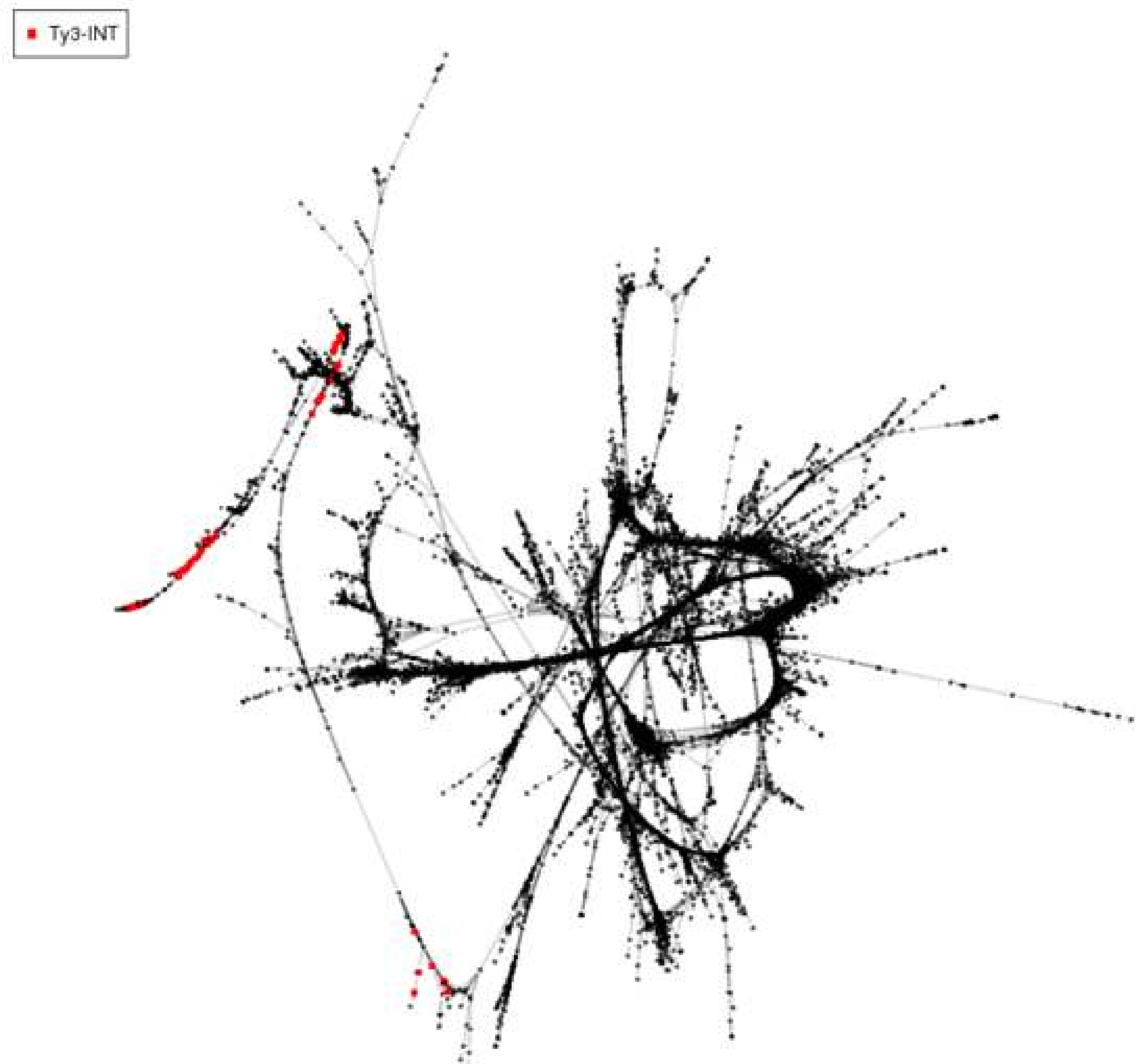

CL152
Low_complexity
Length of Reads (GP):8057 (0.1%)

Tcacao

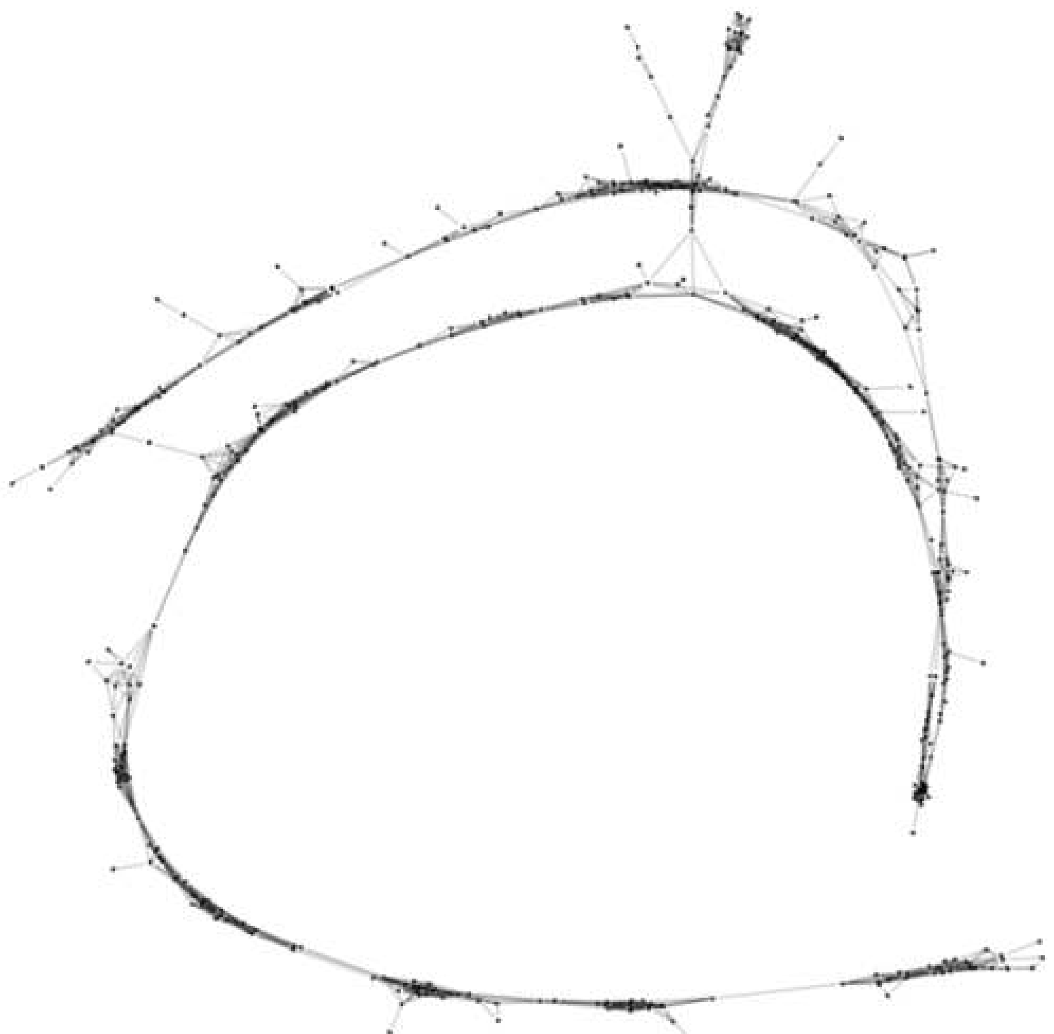

CL152
DNA_PIF_Harbinger
Length of Reads (GP):434 (0.02%)

Tgrandiflorum

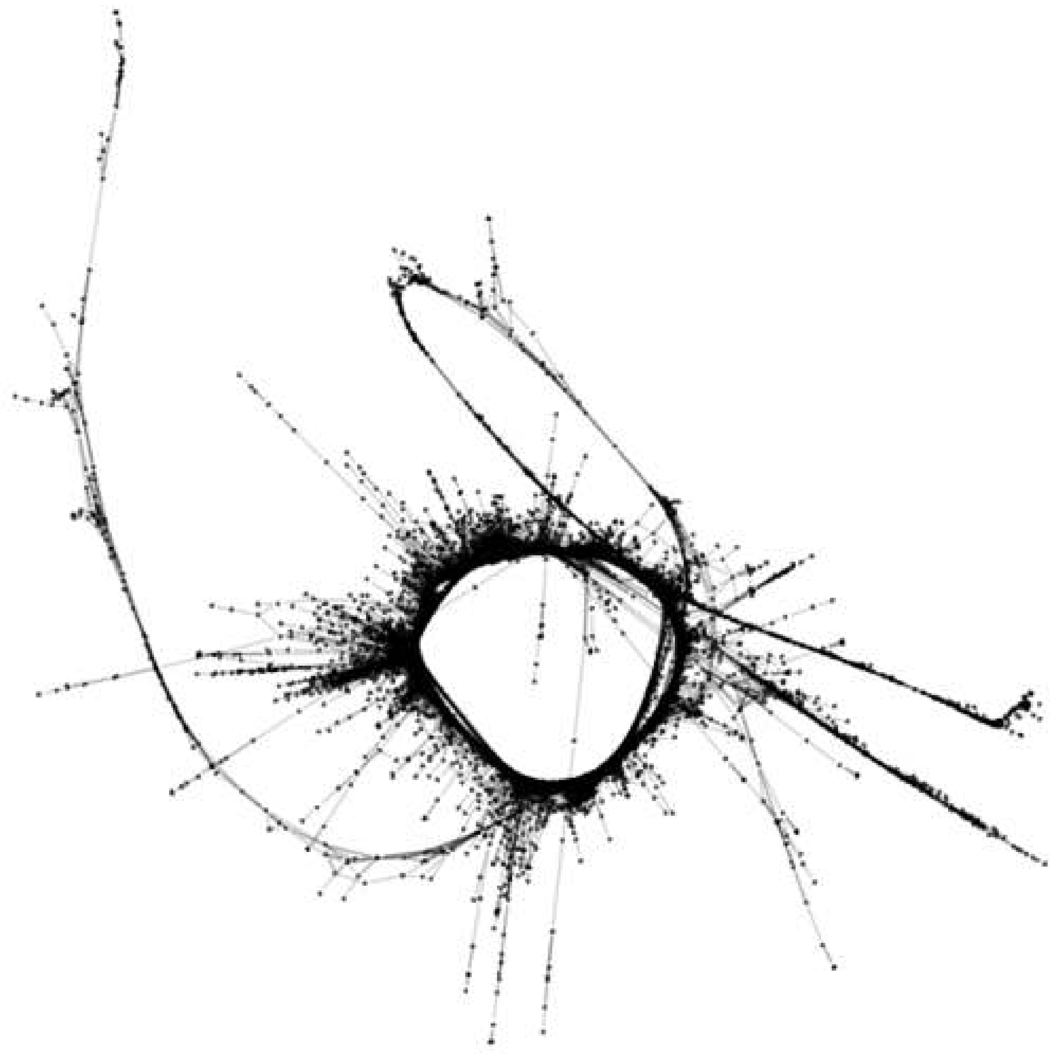

CL153
Low_complexity
Length of Reads (GP):7929 (0.1%)

Tcacao

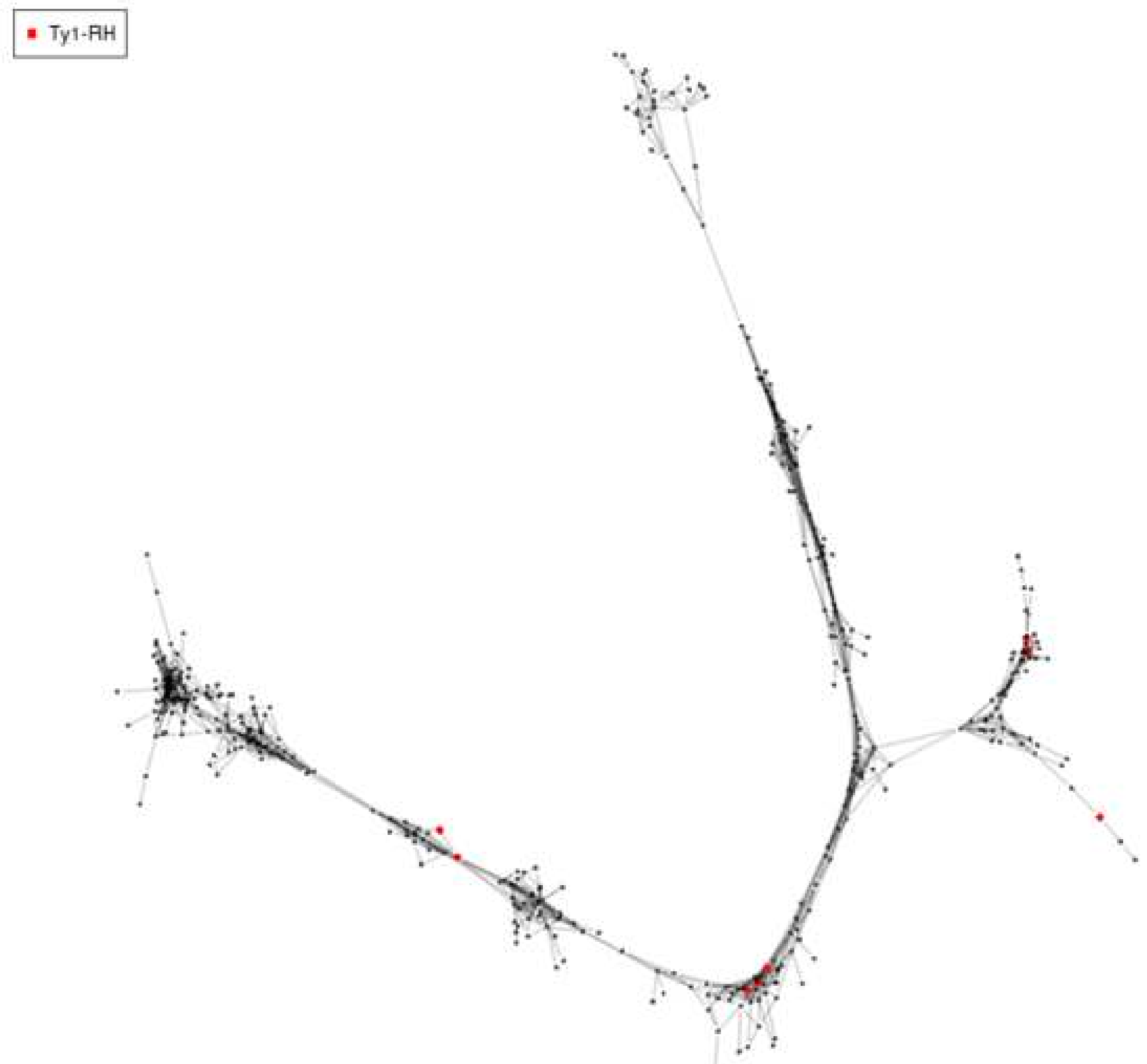

CL153
LTR_Copia
Length of Reads (GP):414 (0.02%)

Tgrandiflorum

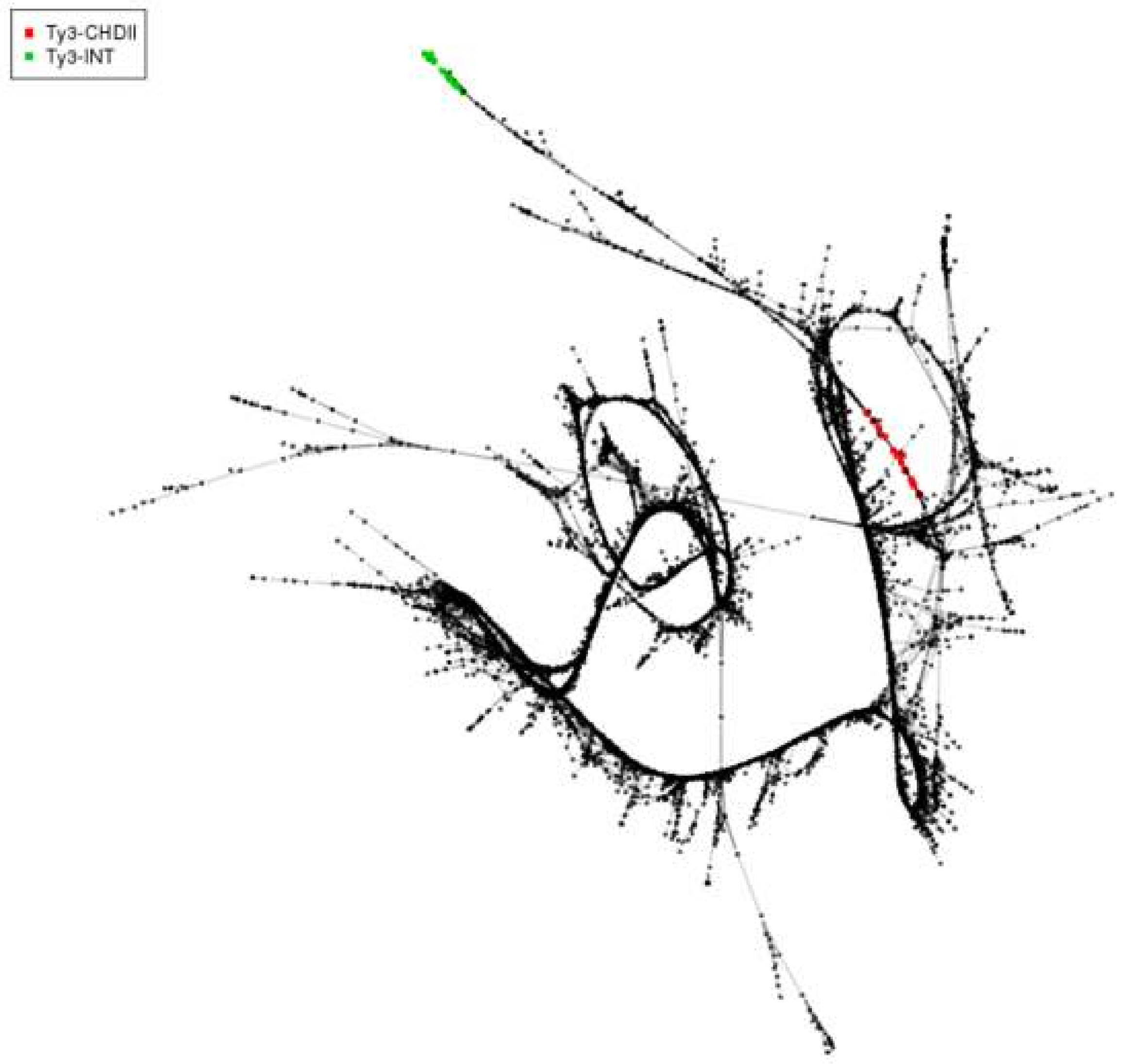

CL154
Low_complexity
Length of Reads (GP):7925 (0.1%)

Tcacao

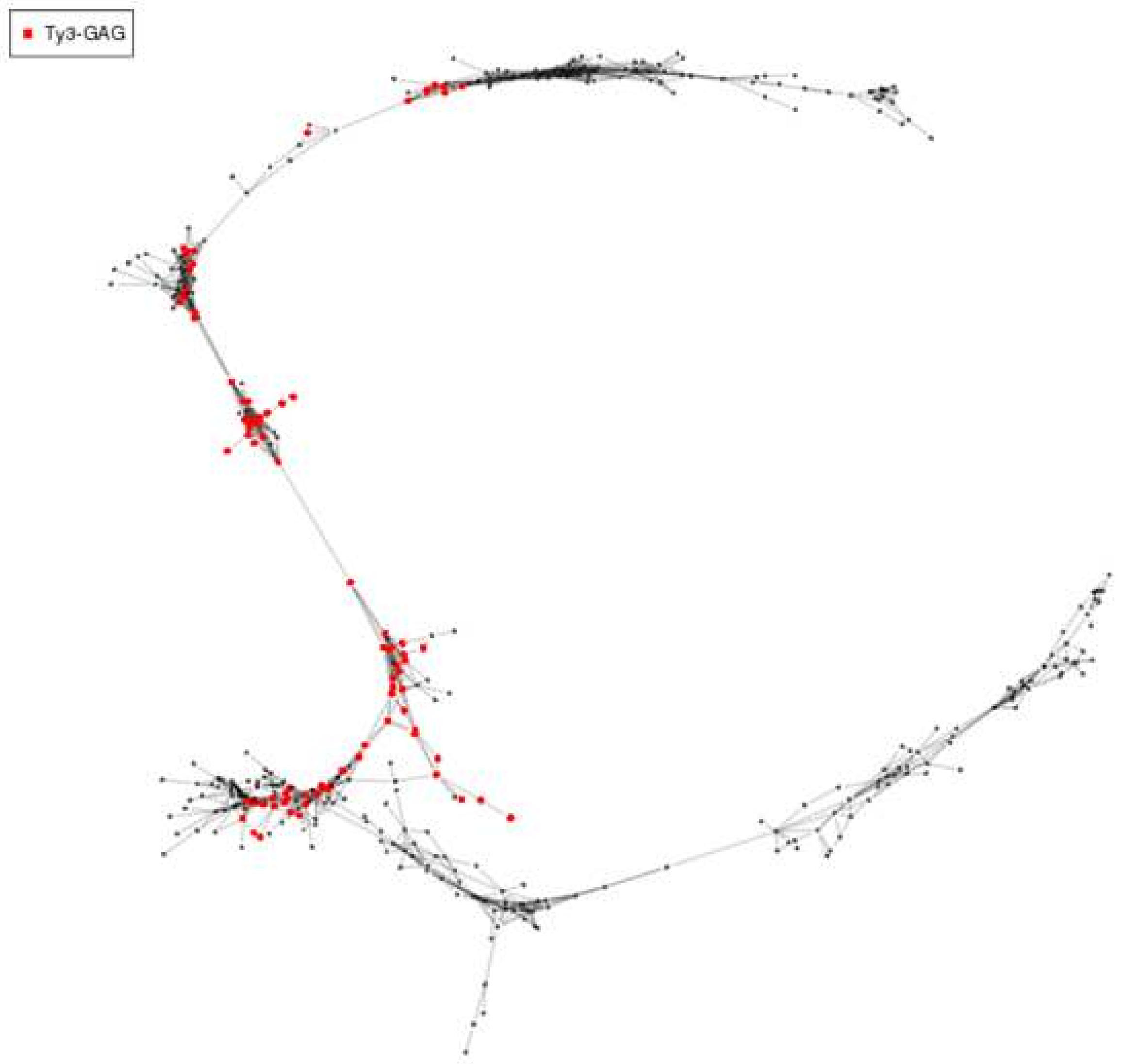

CL154
LTR_Gypsy
Length of Reads (GP):412 (0.02%)

Tgrandiflorum

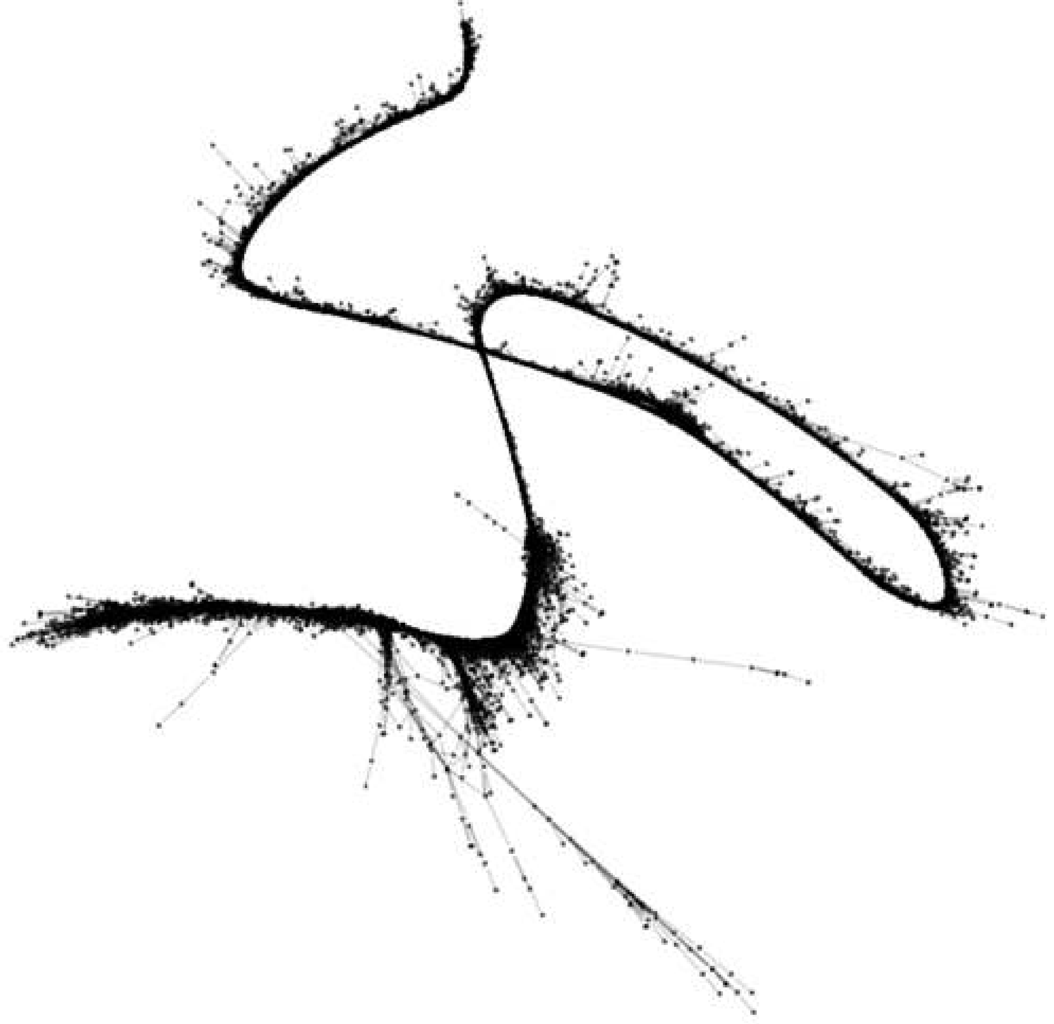

CL155
Low_complexity
Length of Reads (GP):7911 (0.1%)

Tcacao

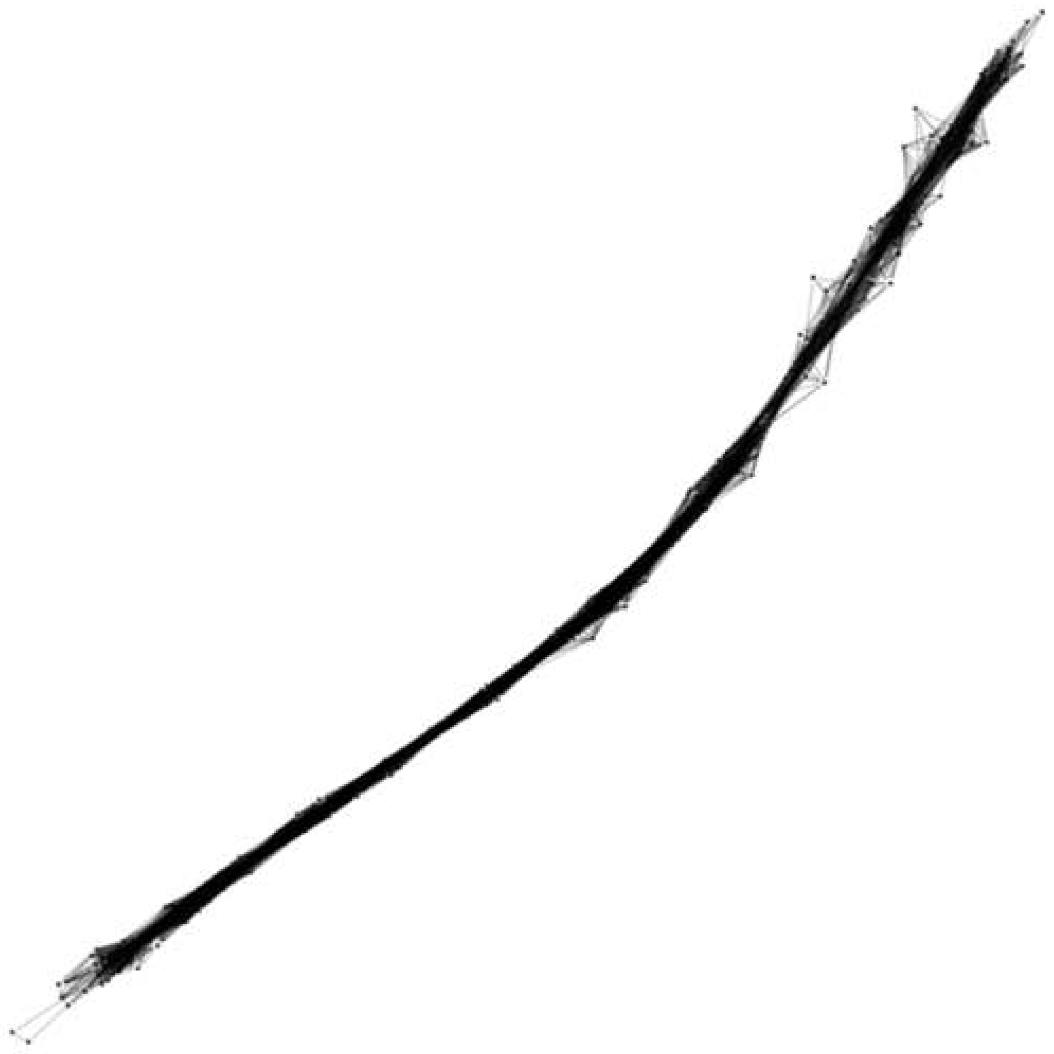

CL155
Simple_repeat
Length of Reads (GP):405 (0.02%)

Tgrandiflorum

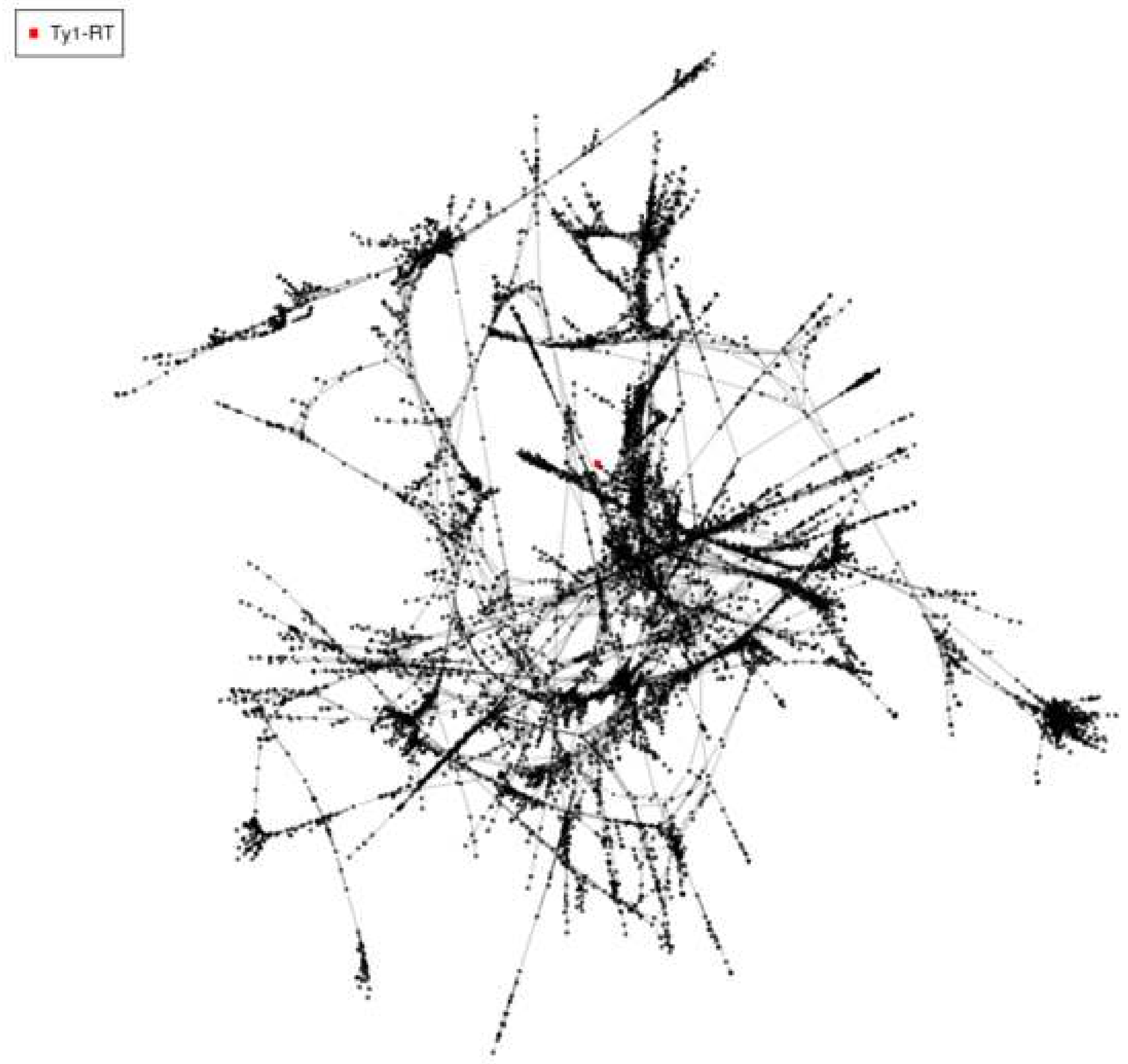

CL156
Low_complexity
Length of Reads (GP):7875 (0.1%)

Tcacao

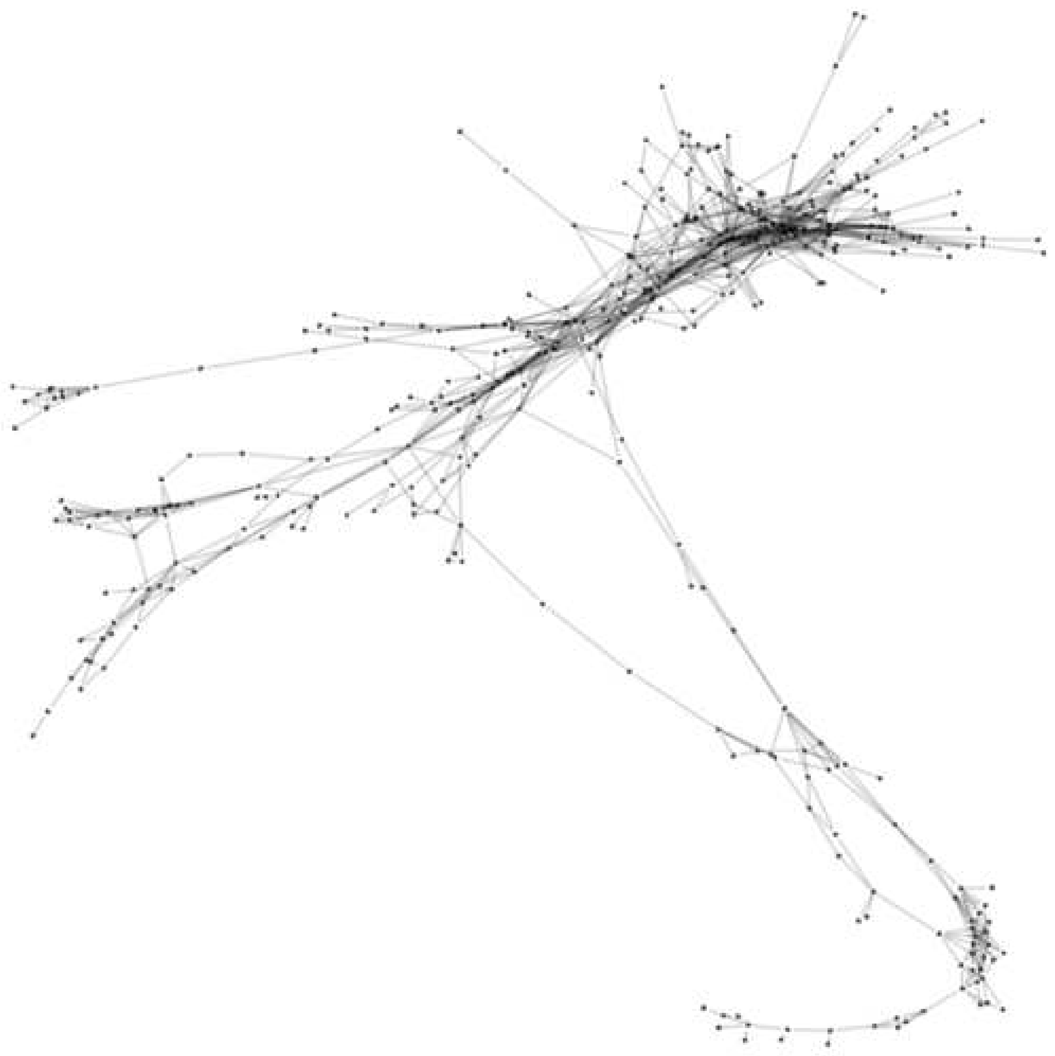

CL156
LTR_Gypsy
Length of Reads (GP):400 (0.02%)

Tgrandiflorum

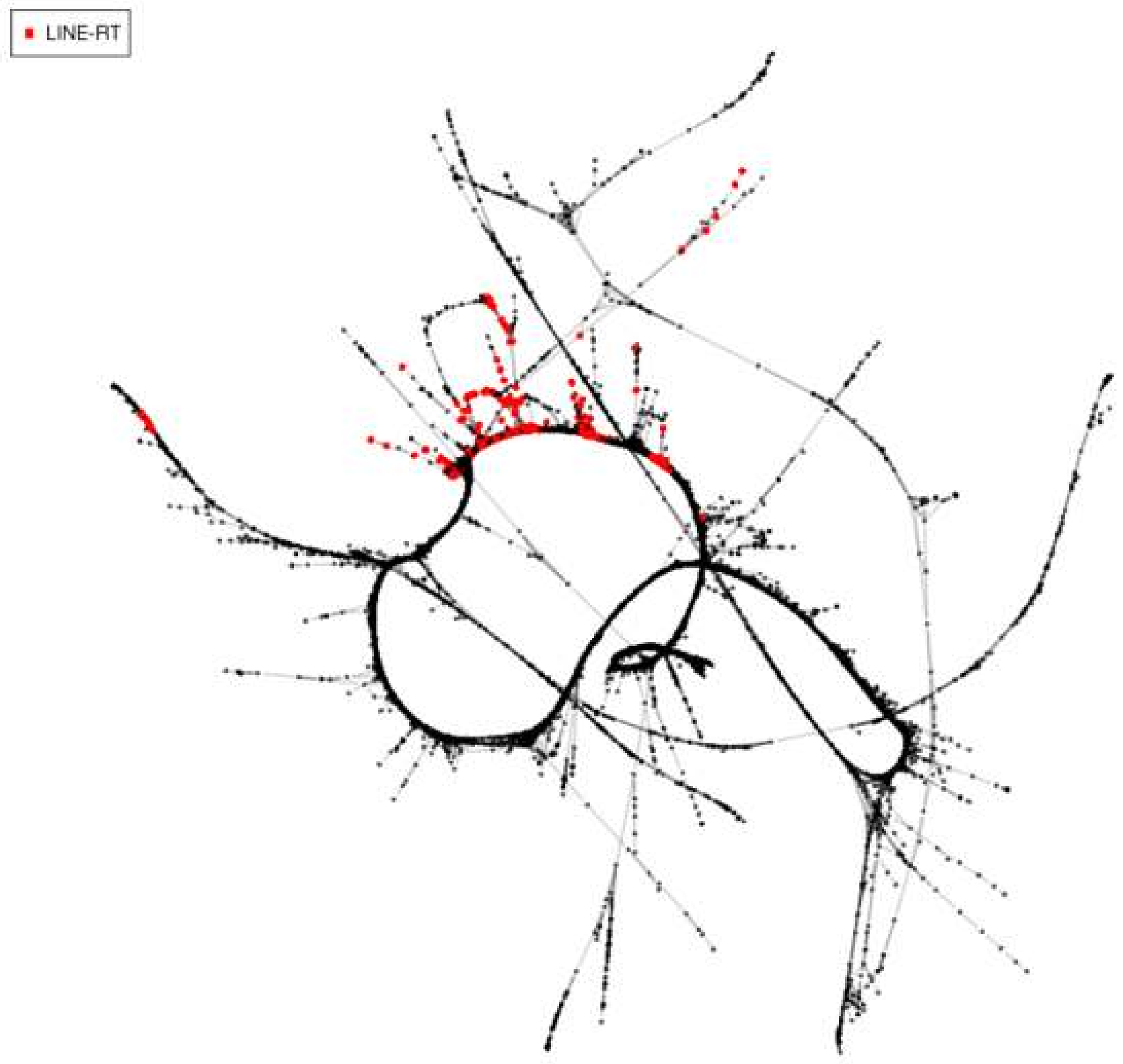

CL157
LINE_L1
Length of Reads (GP):7855 (0.1%)

Tcacao

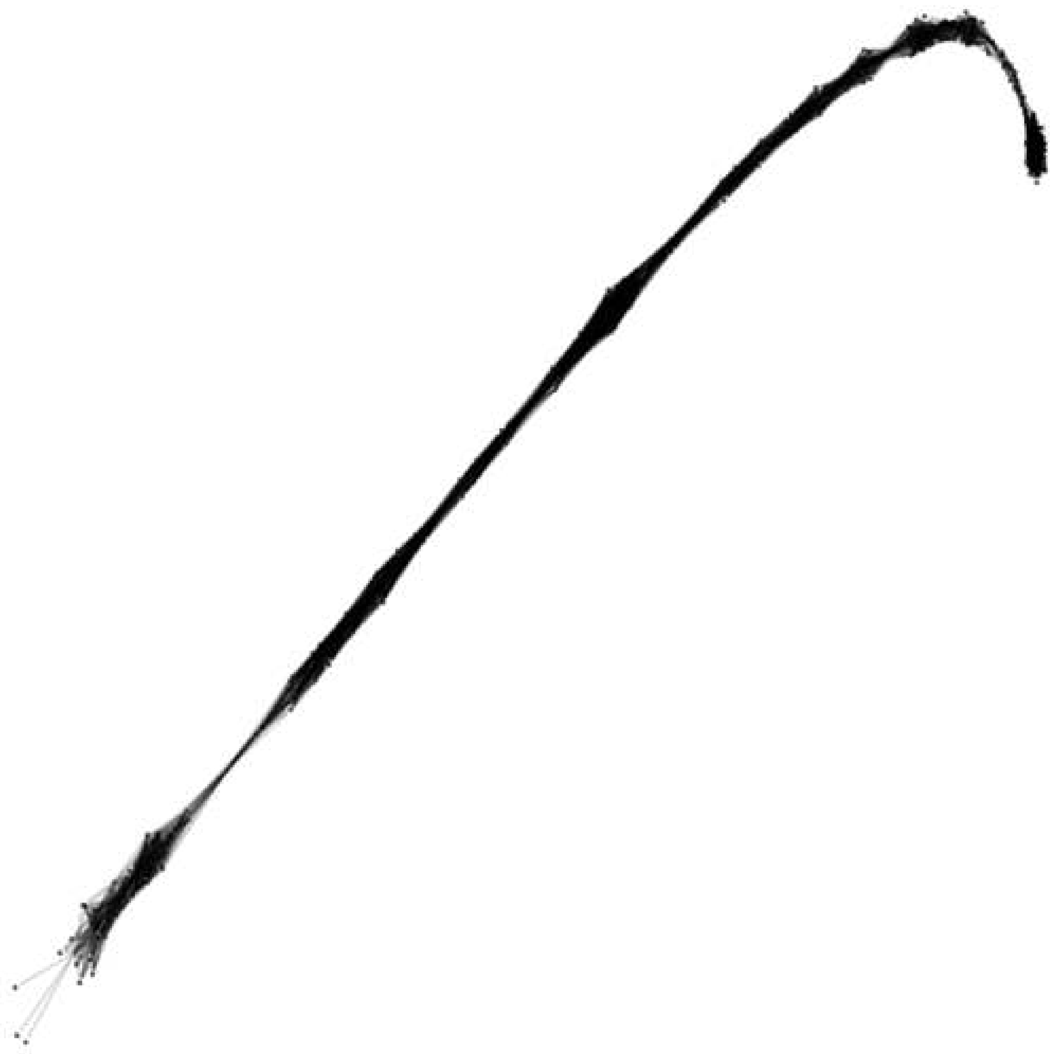

CL157
Low_complexity
Length of Reads (GP):397 (0.02%)

Tgrandiflorum

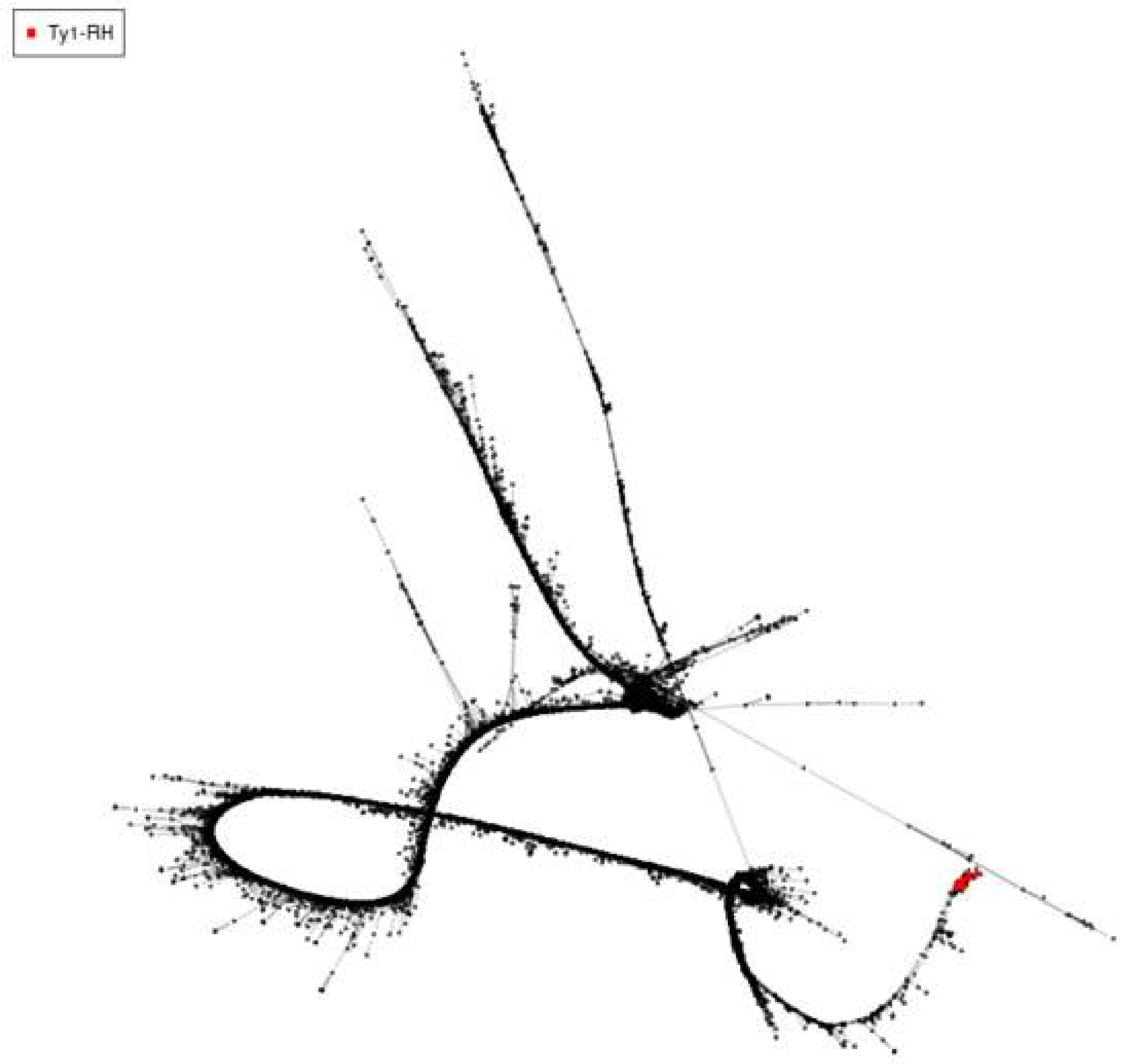

CL158
Low_complexity
Length of Reads (GP):7782 (0.1%)

Tcacao

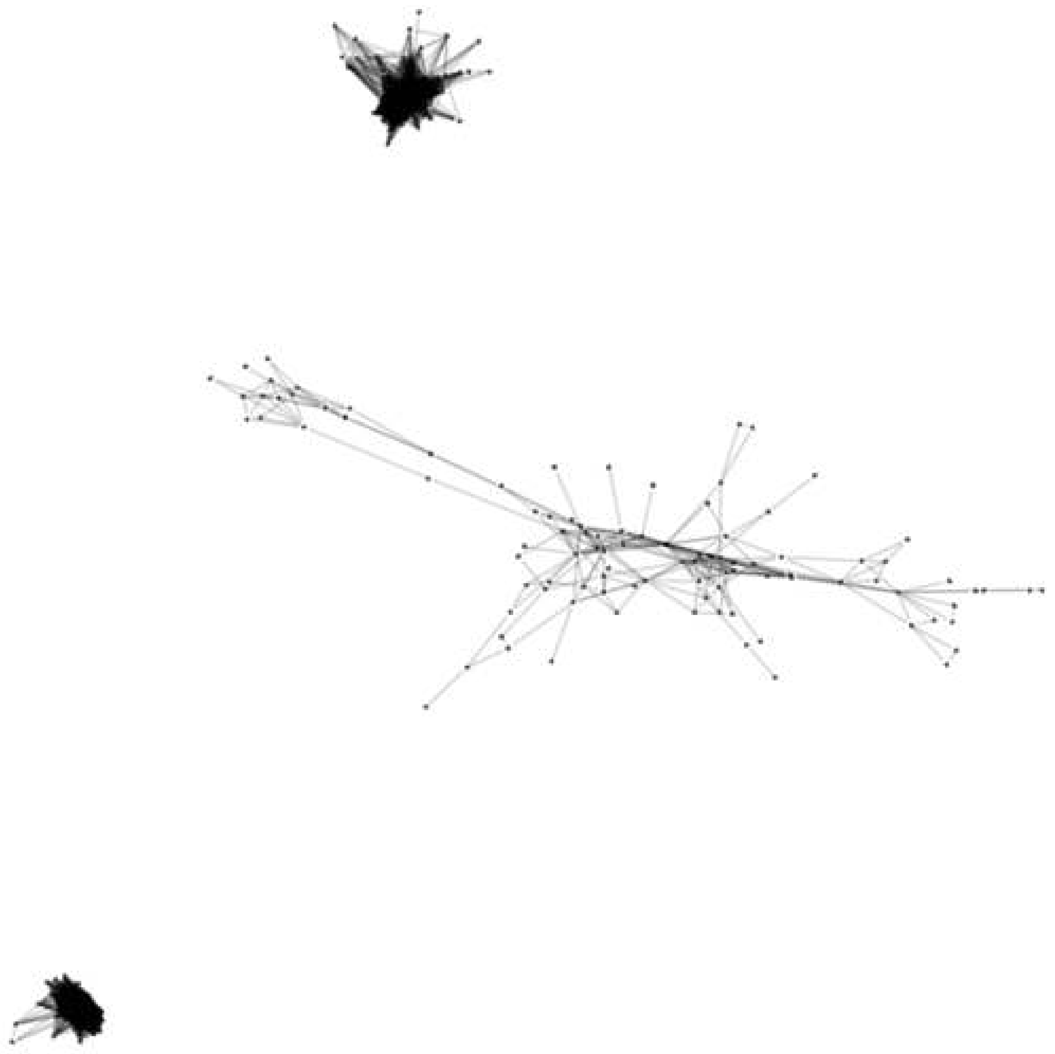

CL158
Low_complexity
Length of Reads (GP):379 (0.02%)

Tgrandiflorum

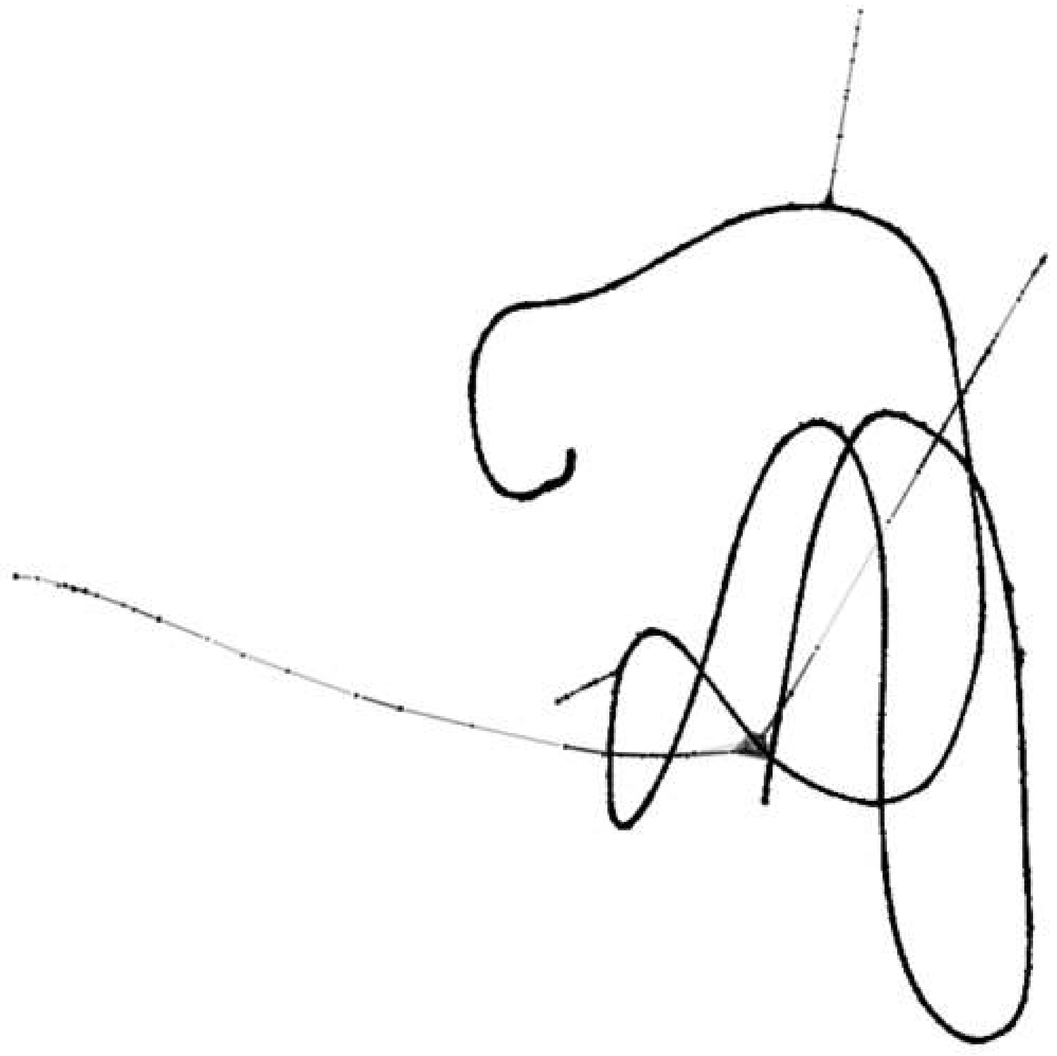

CL159
LTR_Copia
Length of Reads (GP):7702 (0.1%)

Tcacao

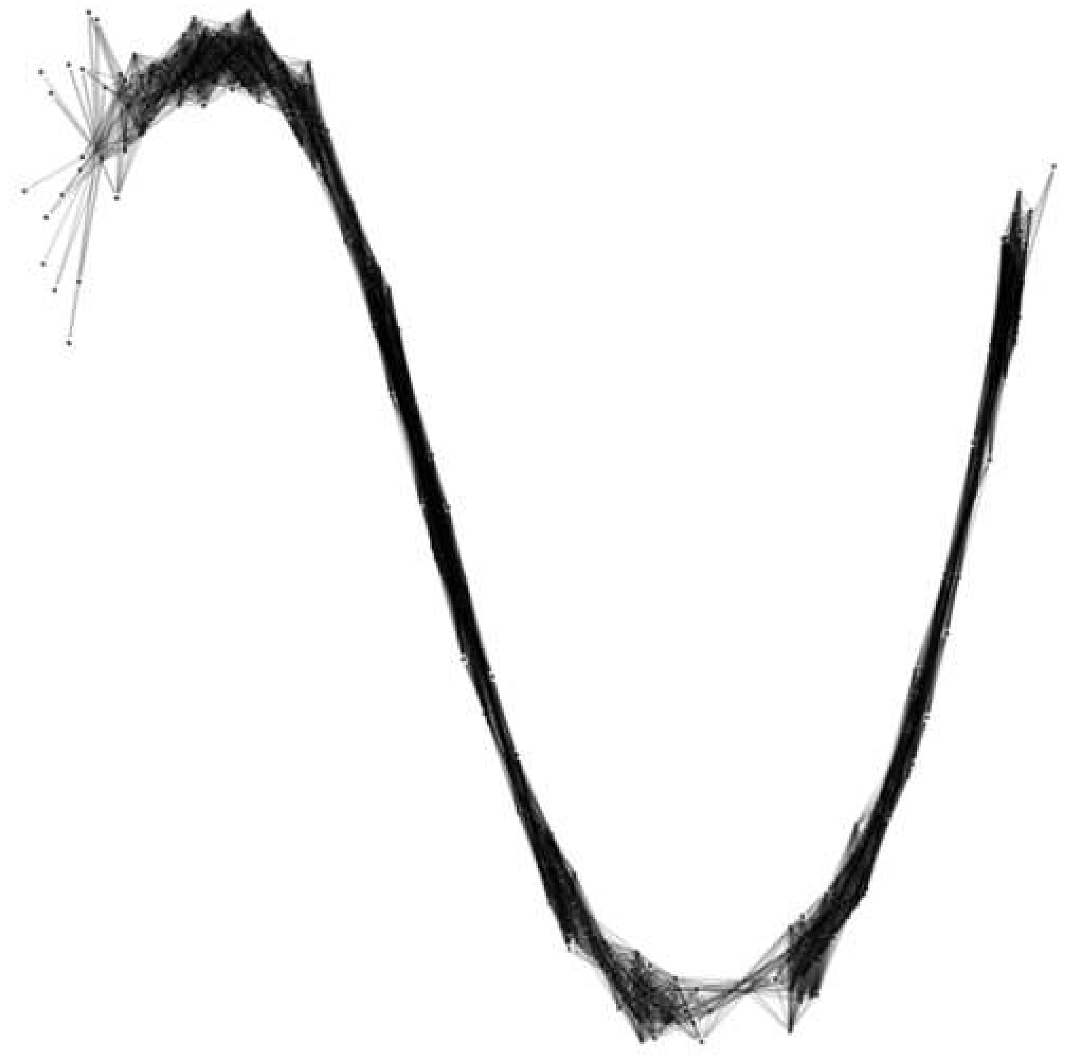

CL159
Low_complexity
Length of Reads (GP):374 (0.02%)

Tgrandiflorum

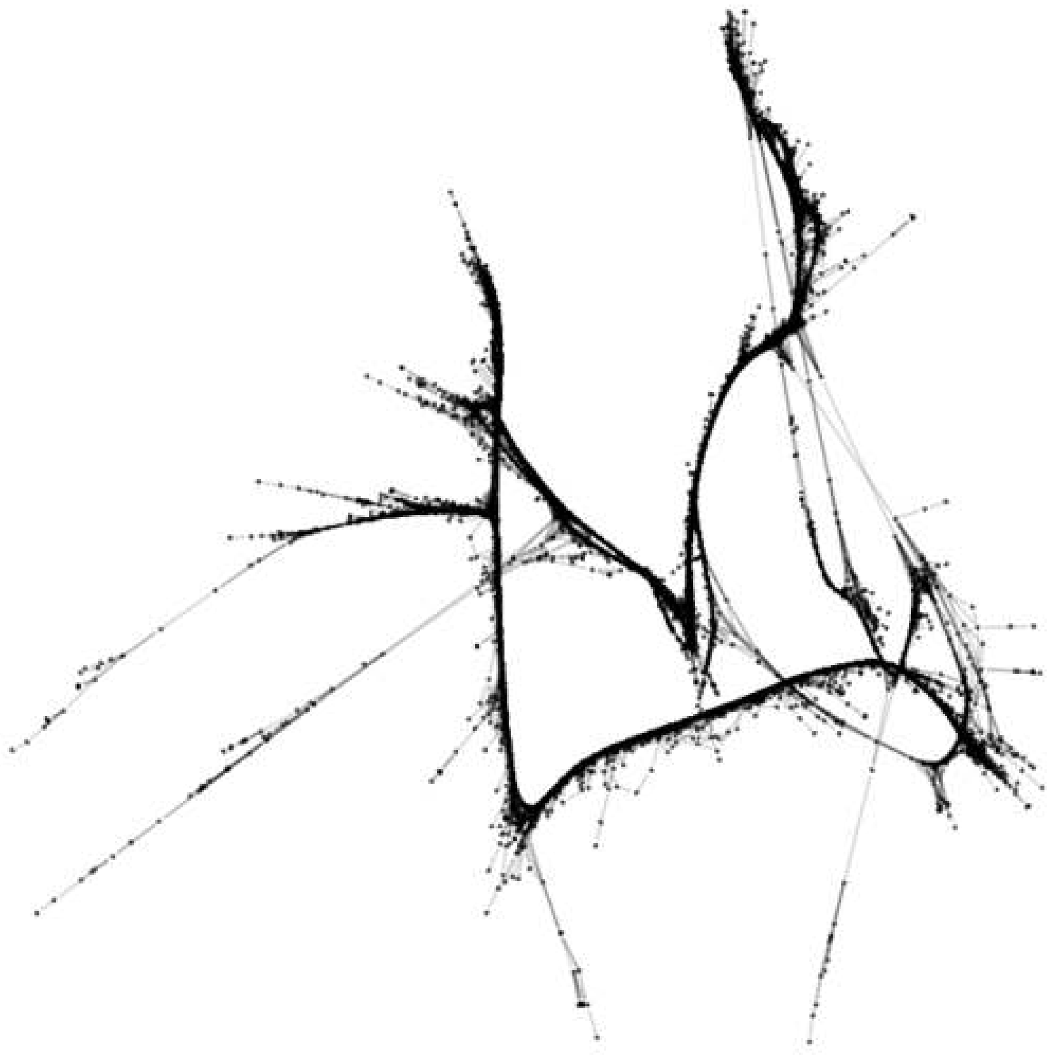

CL160
LTR_Gypsy
Length of Reads (GP):7647 (0.1%)

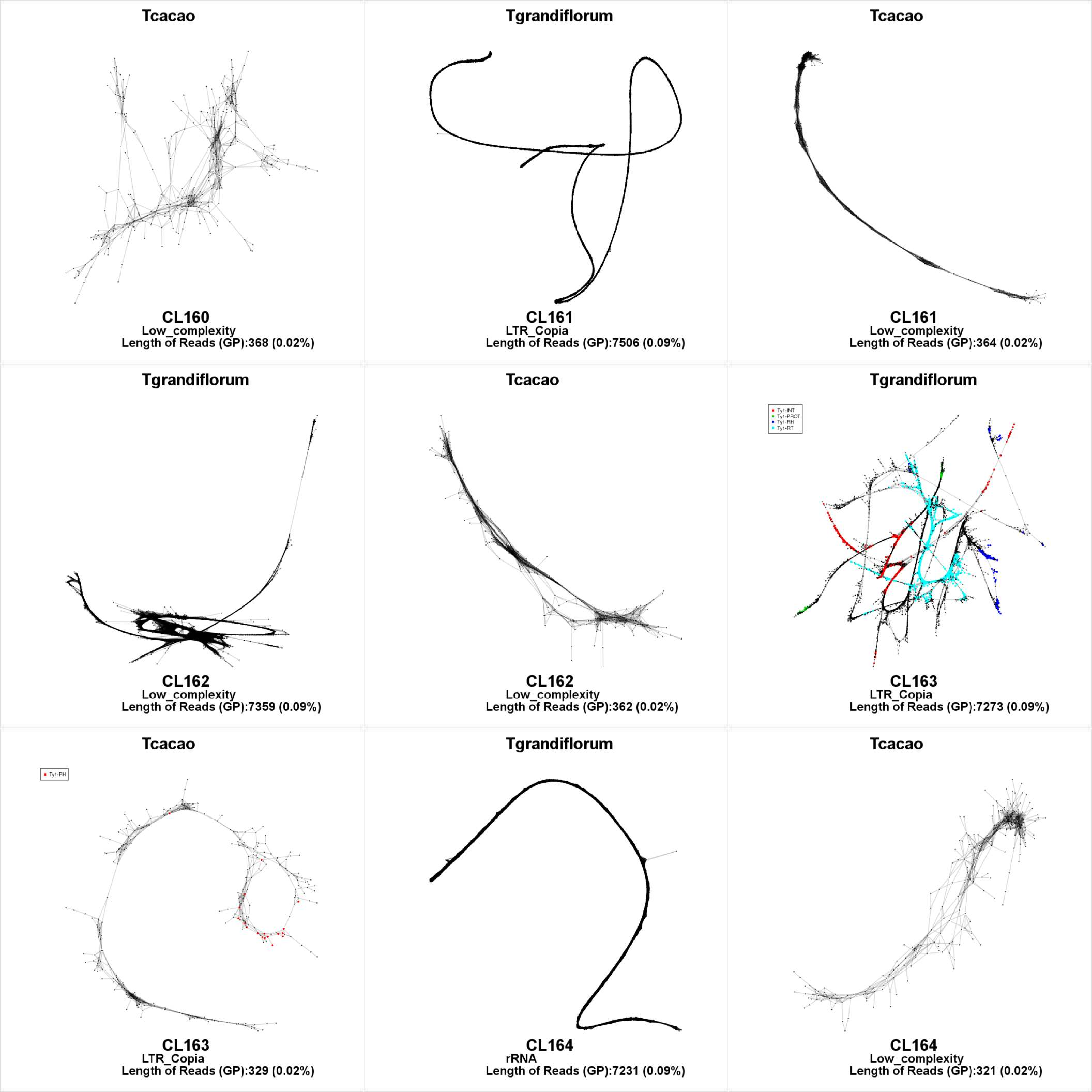
Tcacao
CL160
Low_complexity
Length of Reads (GP):368 (0.02%)
Tgrandiflorum
CL161
LTR_Copia
Length of Reads (GP):7506 (0.09%)
Tcacao
CL161
Low_complexity
Length of Reads (GP):364 (0.02%)
Tgrandiflorum
CL162
Low_complexity
Length of Reads (GP):7359 (0.09%)
Tcacao
CL162
Low_complexity
Length of Reads (GP):362 (0.02%)
Tgrandiflorum
Ty1-INT
Ty1-PROT
Ty1-RH
Ty1-RT
CL163
LTR_Copia
Length of Reads (GP):7273 (0.09%)
Tcacao
Ty1-RH
CL163
LTR_Copia
Length of Reads (GP):329 (0.02%)
Tgrandiflorum
CL164
rRNA
Length of Reads (GP):7231 (0.09%)
Tcacao
CL164
Low_complexity
Length of Reads (GP):321 (0.02%)

Tgrandiflorum

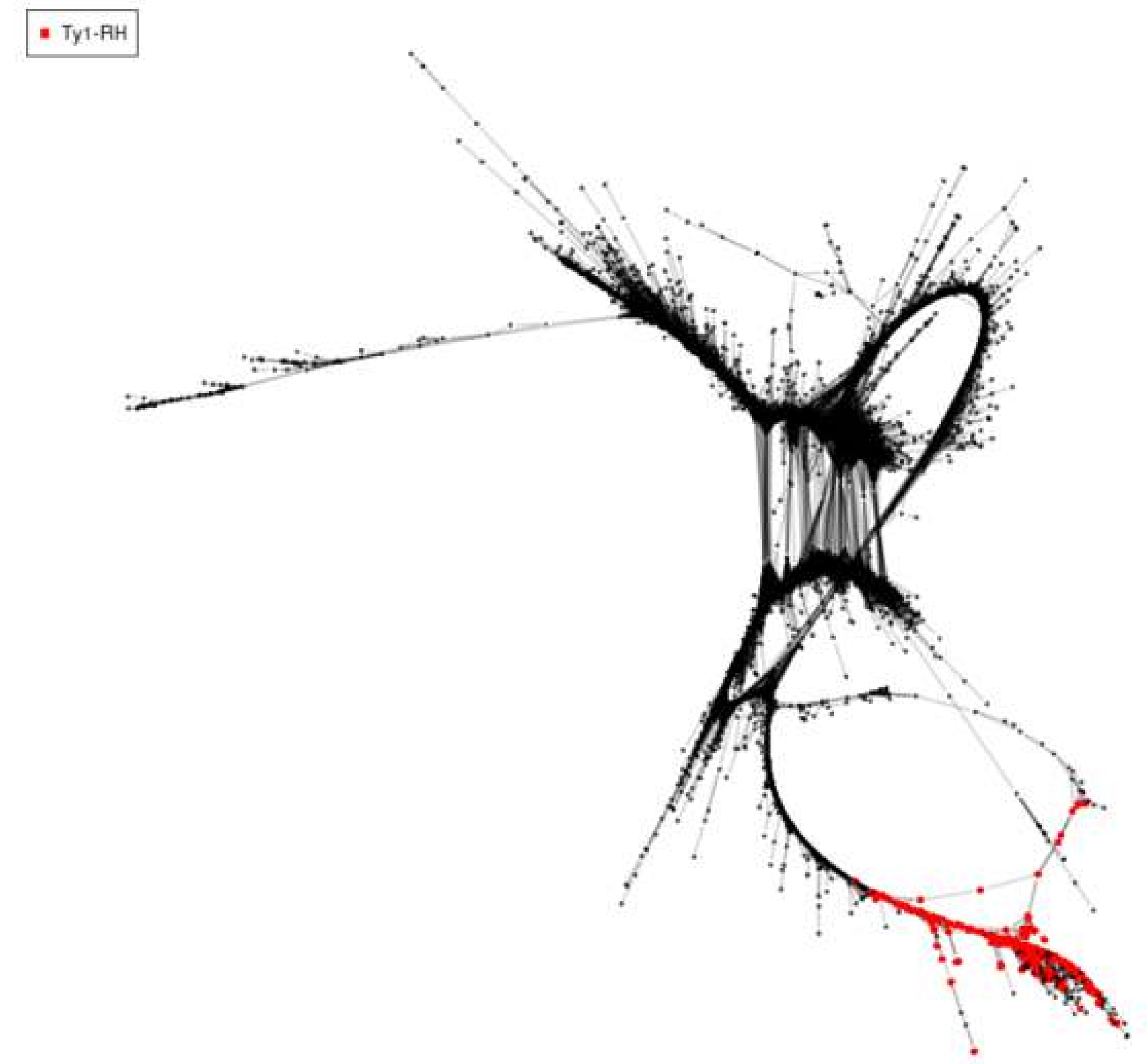

CL165
LTR_Copia
Length of Reads (GP):7163 (0.09%)

Tcacao

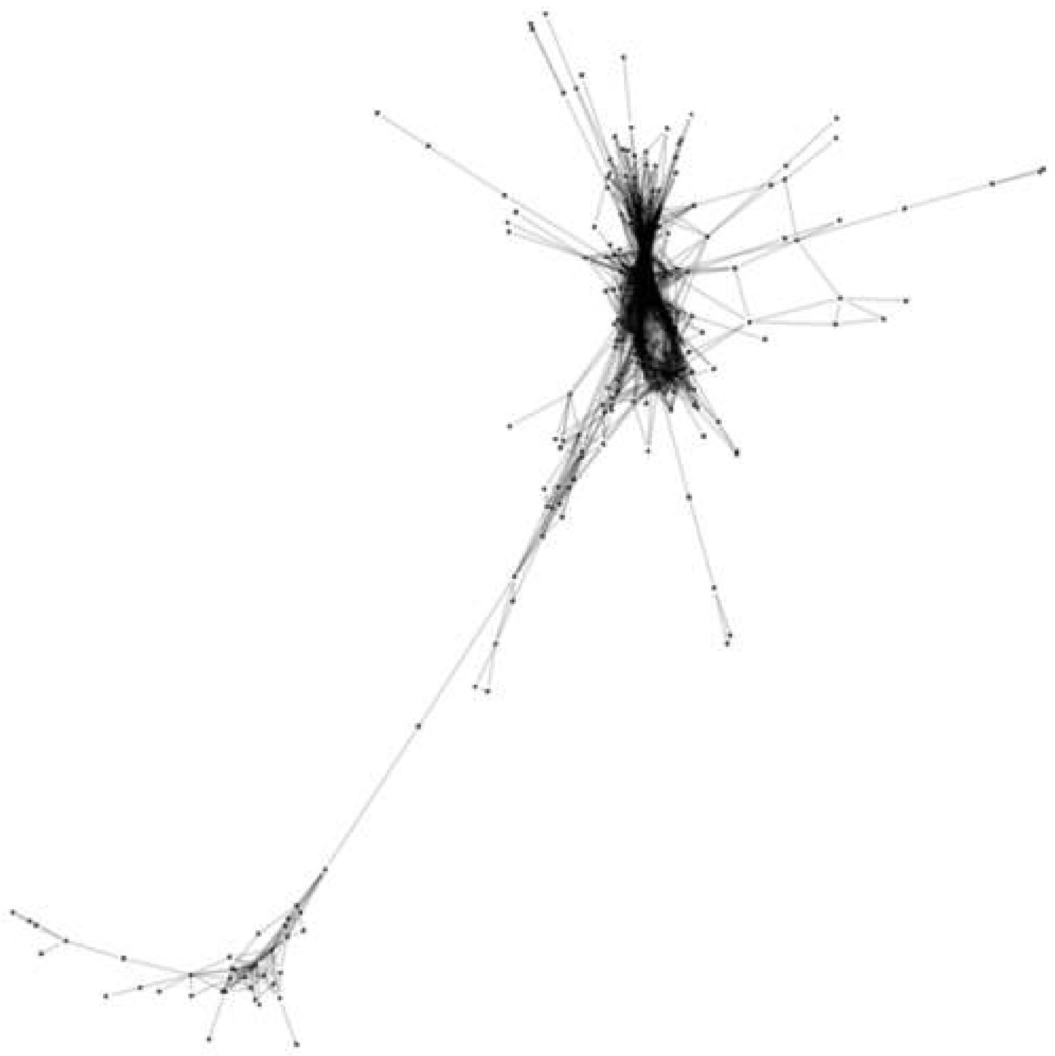

CL165
Low_complexity
Length of Reads (GP):320 (0.02%)

Tgrandiflorum

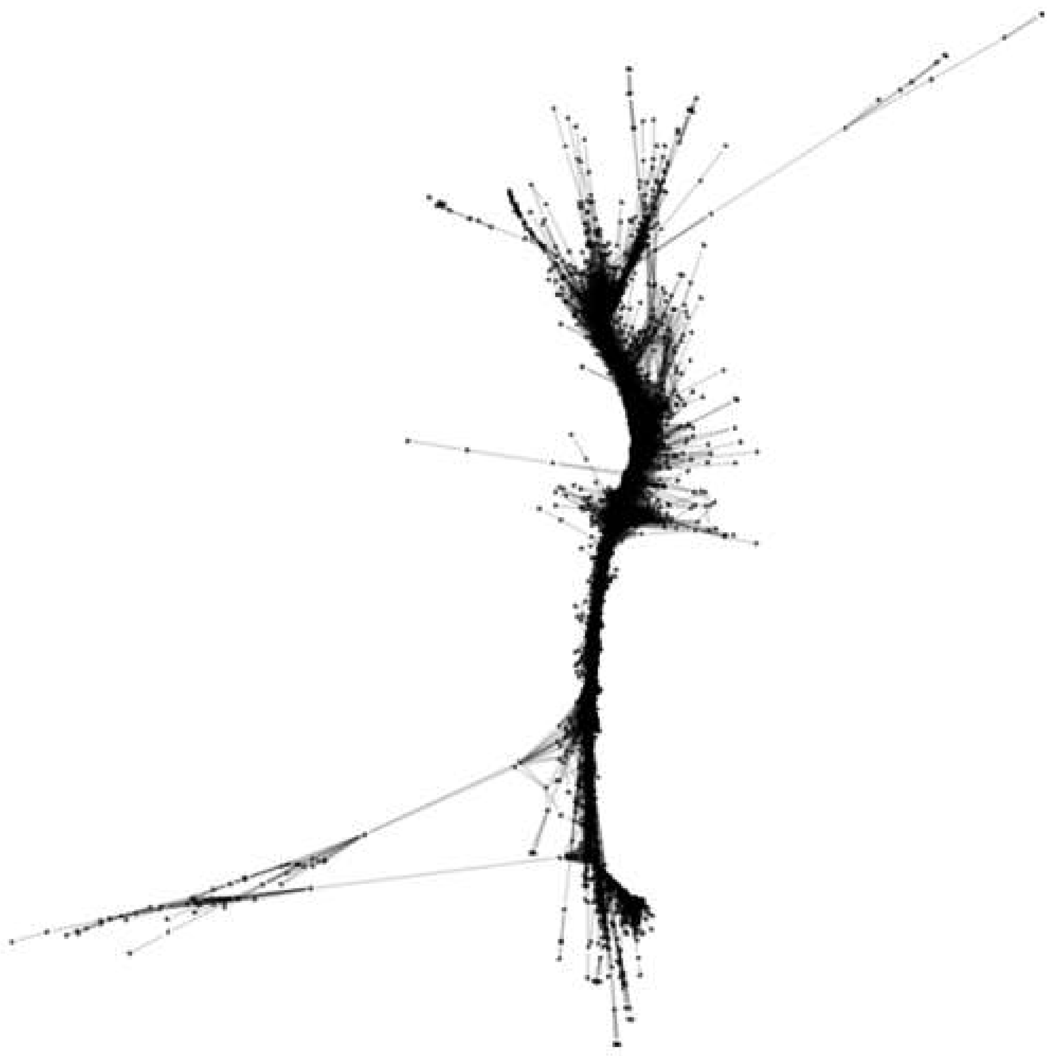

CL166
Low_complexity
Length of Reads (GP):7096 (0.09%)

Tcacao

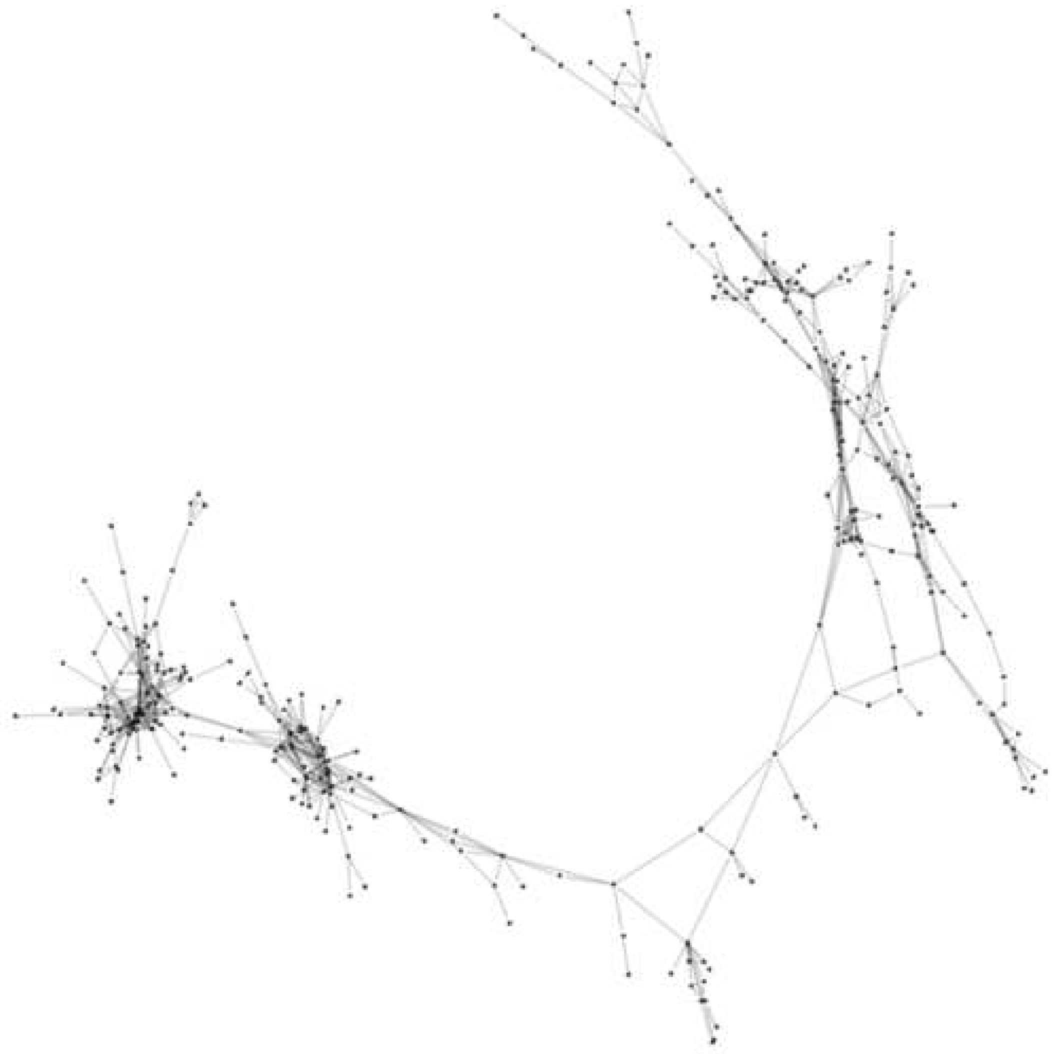

CL166
LTR_Gypsy
Length of Reads (GP):319 (0.02%)

Tgrandiflorum

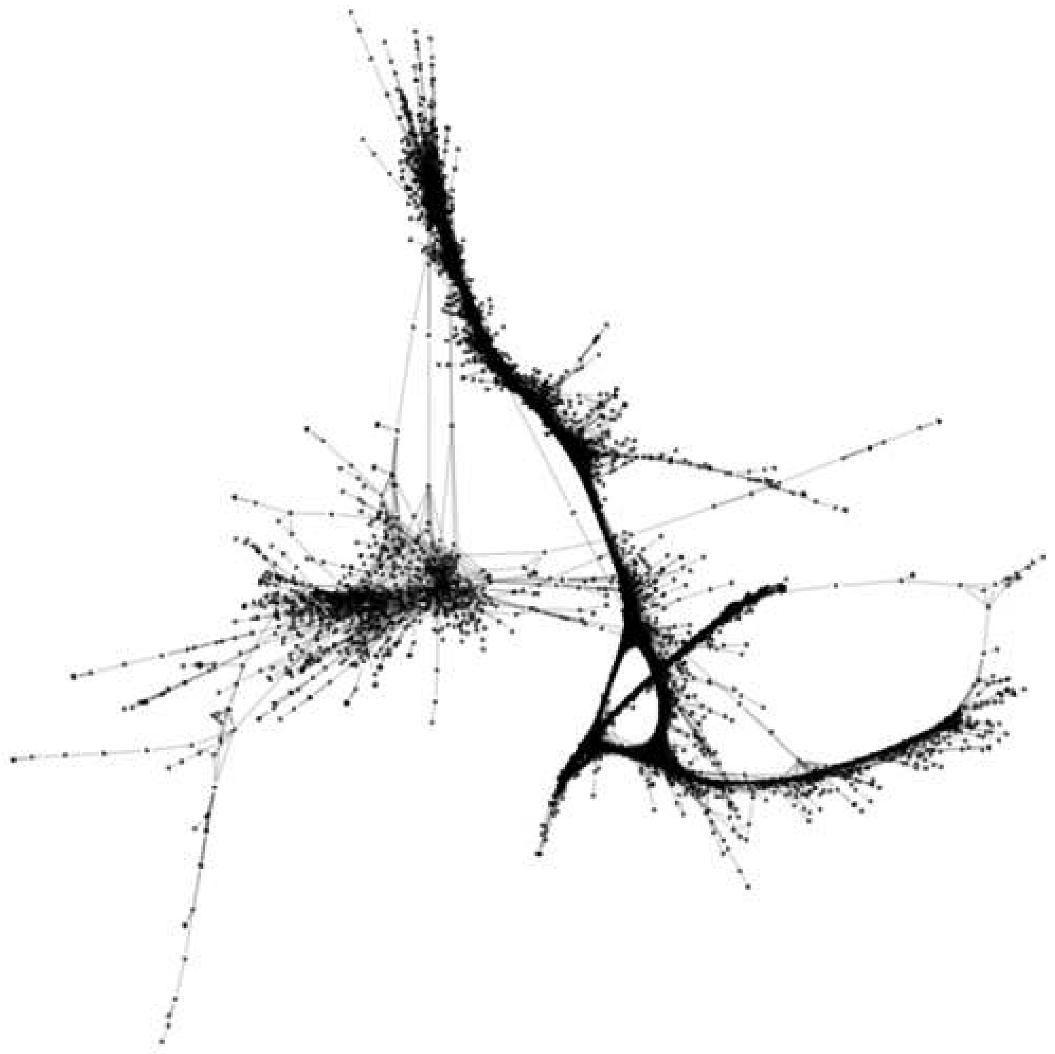

CL167
Low_complexity
Length of Reads (GP):7045 (0.09%)

Tcacao

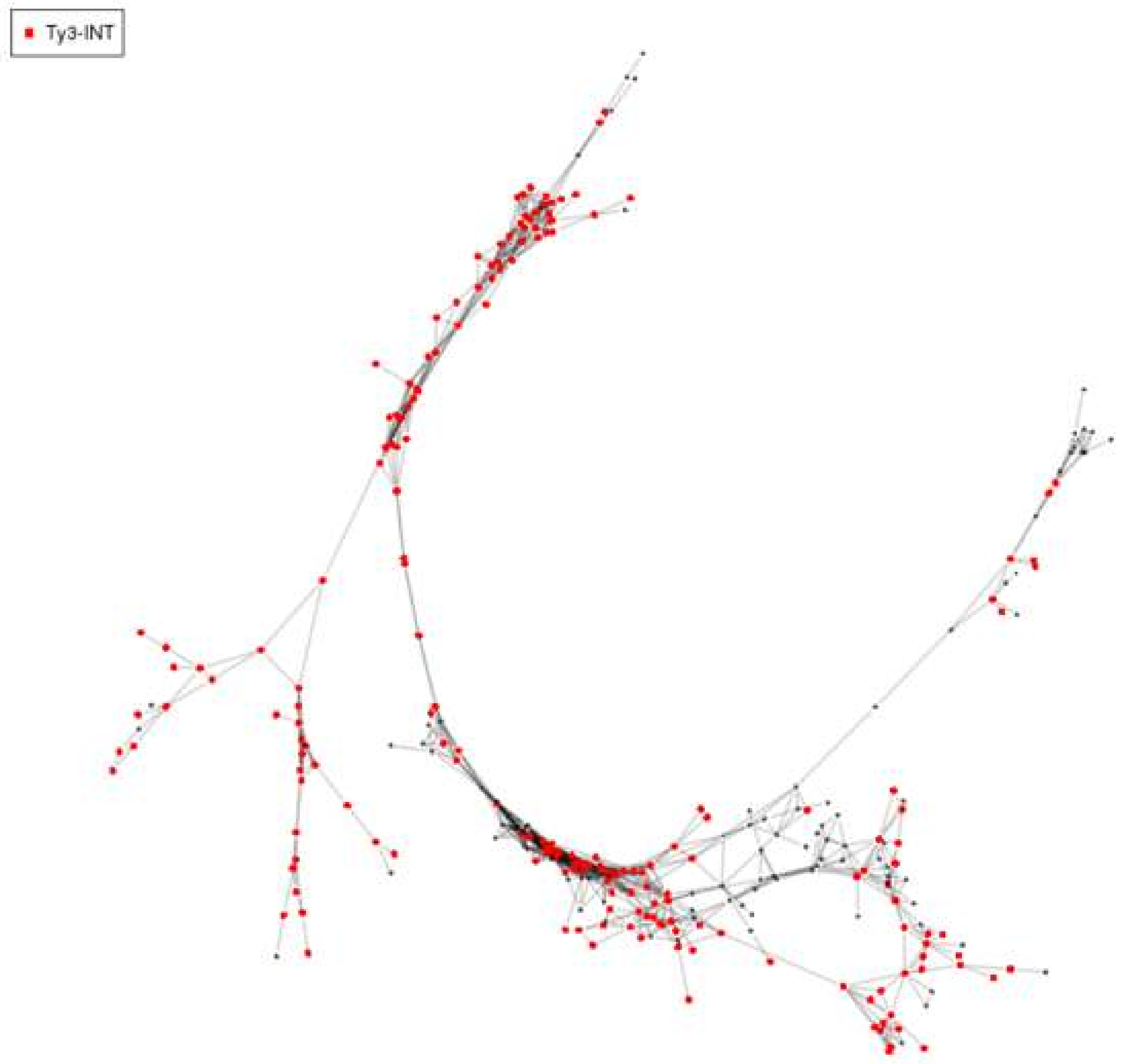

CL167
LTR_Gypsy
Length of Reads (GP):297 (0.01%)

Tgrandiflorum

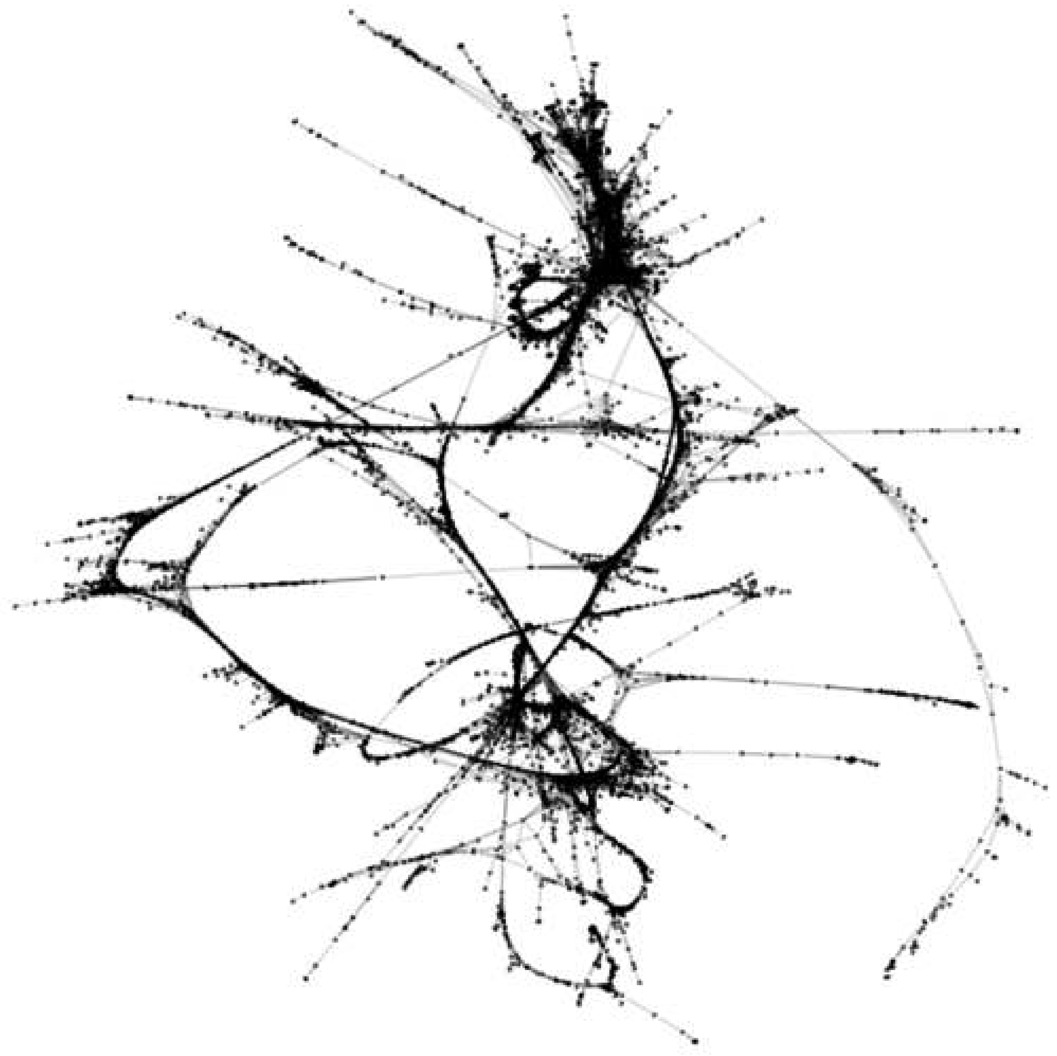

CL168
Low_complexity
Length of Reads (GP):6884 (0.09%)

Tcacao

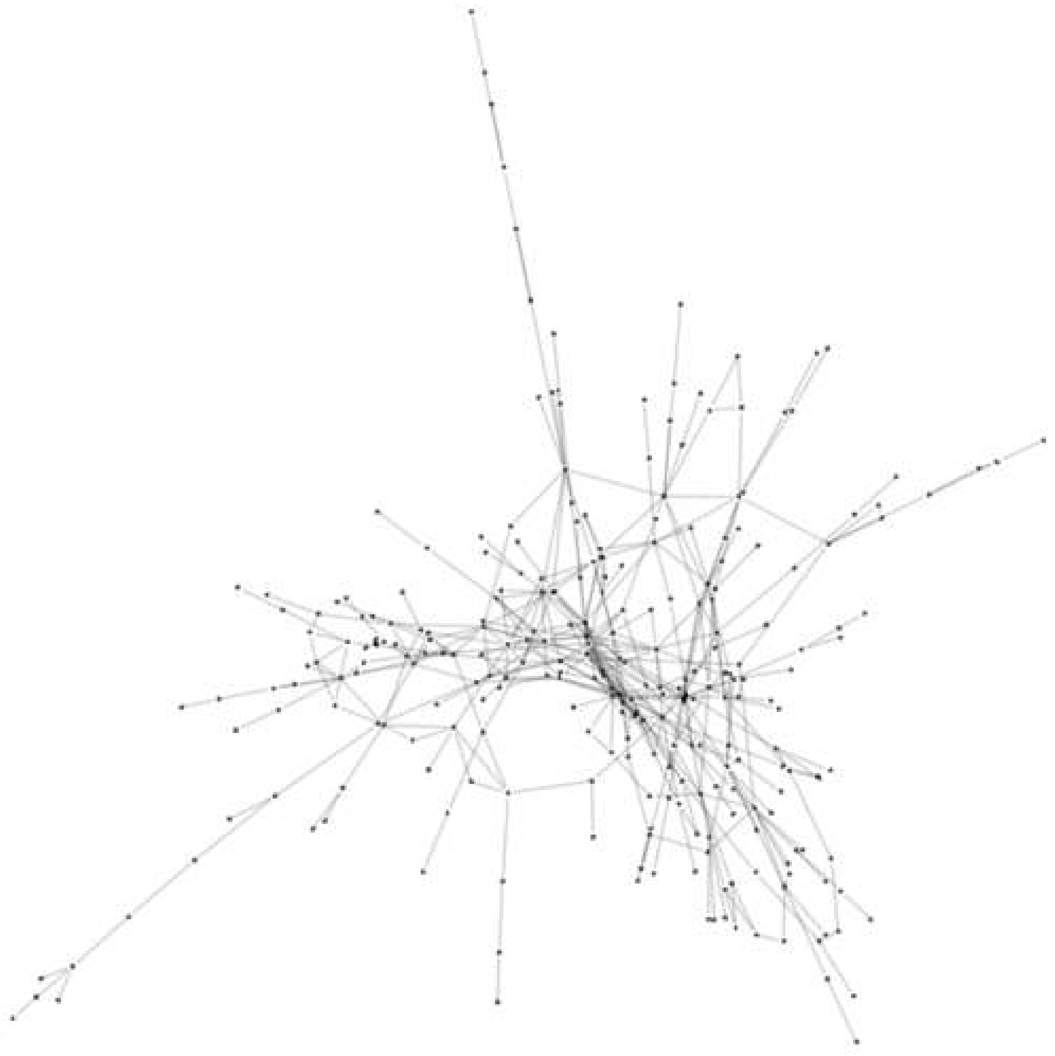

CL168
Low_complexity
Length of Reads (GP):280 (0.01%)

Tgrandiflorum

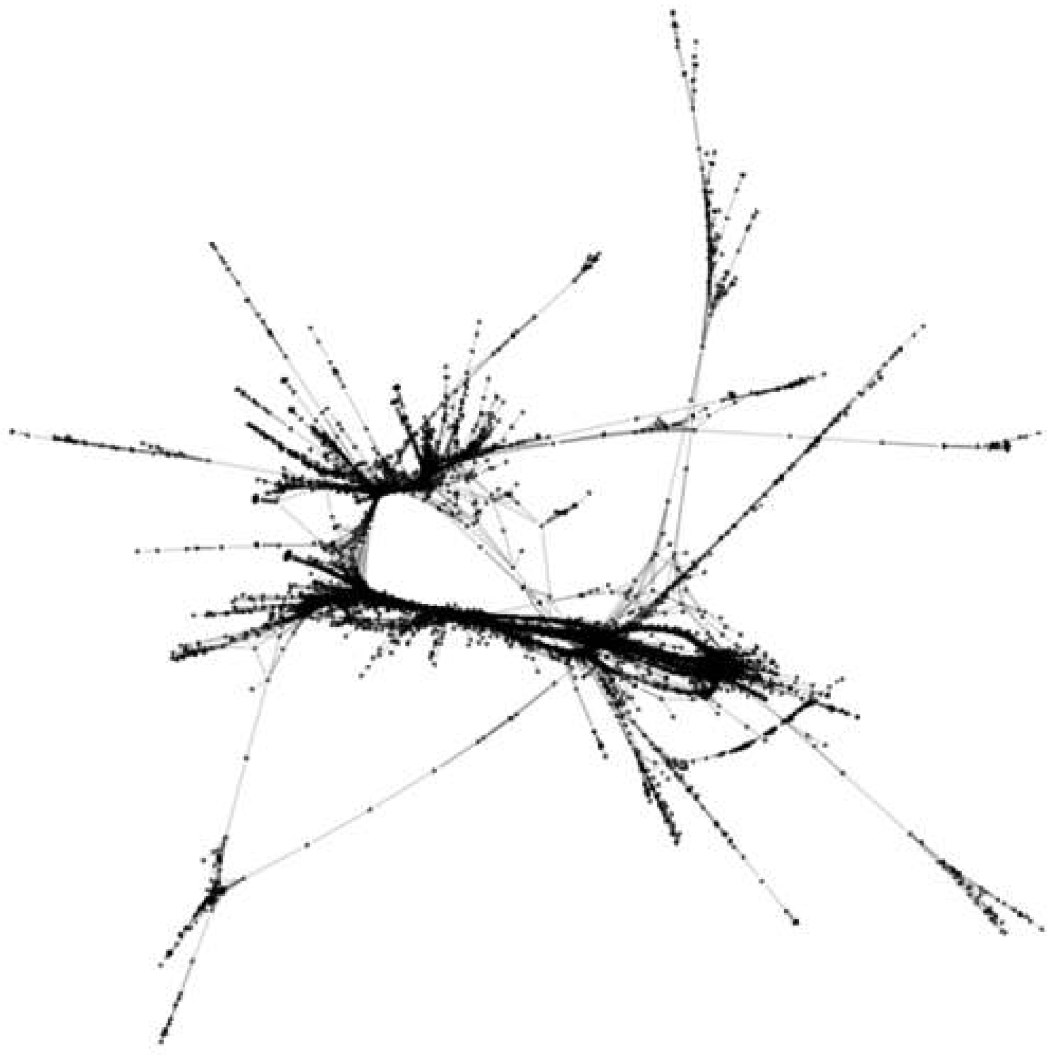

CL169
Low_complexity
Length of Reads (GP):6877 (0.09%)

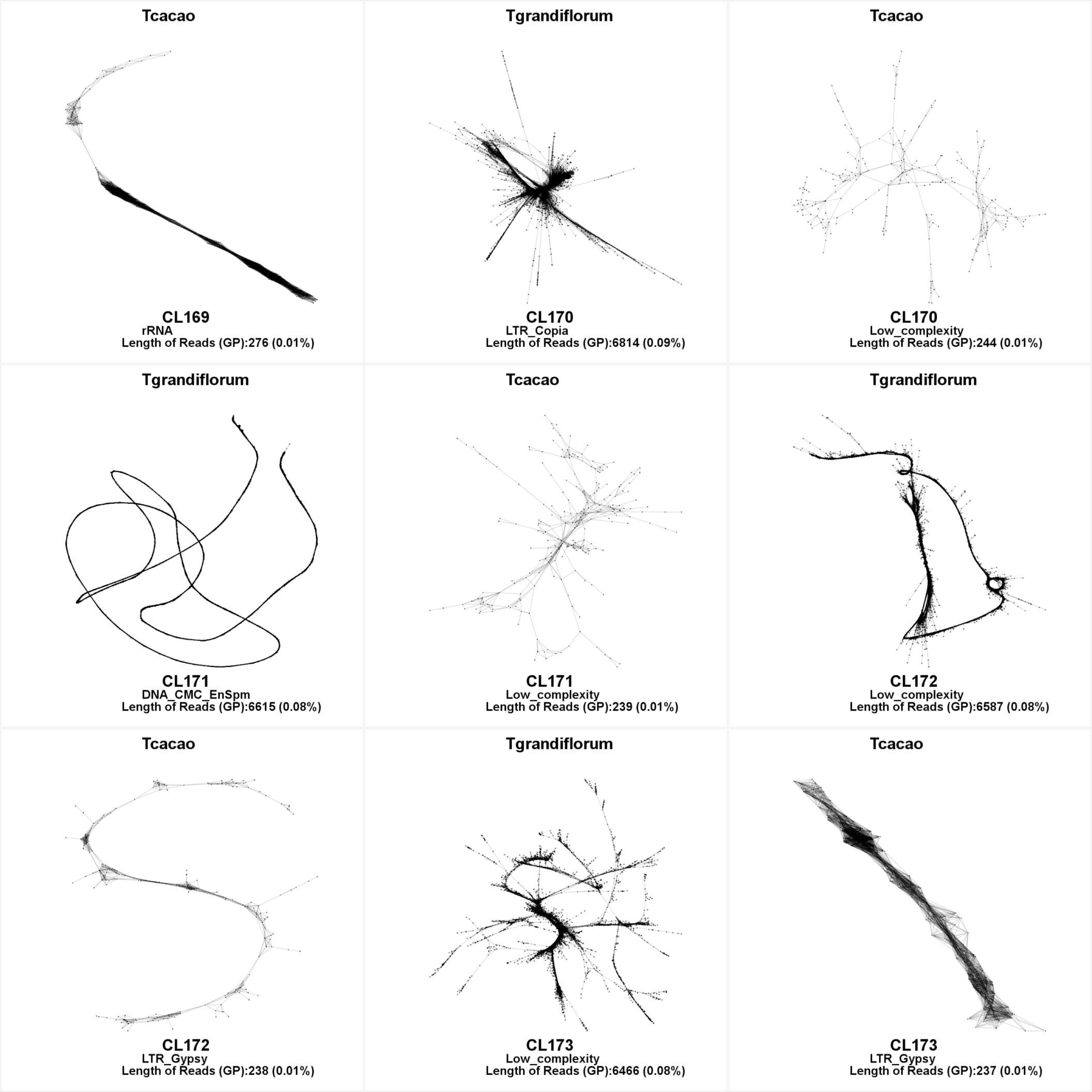
Tcacao
CL169
rRNA
Length of Reads (GP):276 (0.01%)
Tgrandiflorum
CL170
LTR_Copia
Length of Reads (GP):6814 (0.09%)
Tcacao
CL170
Low_complexity
Length of Reads (GP):244 (0.01%)
Tgrandiflorum
CL171
DNA_CMC_EnSpm
Length of Reads (GP):6615 (0.08%)
Tcacao
CL171
Low_complexity
Length of Reads (GP):239 (0.01%)
Tgrandiflorum
CL172
Low_complexity
Length of Reads (GP):6587 (0.08%)
Tcacao
CL172
LTR_Gypsy
Length of Reads (GP):238 (0.01%)
Tgrandiflorum
CL173
Low_complexity
Length of Reads (GP):6466 (0.08%)
Tcacao
CL173
LTR_Gypsy
Length of Reads (GP):237 (0.01%)

Tgrandiflorum

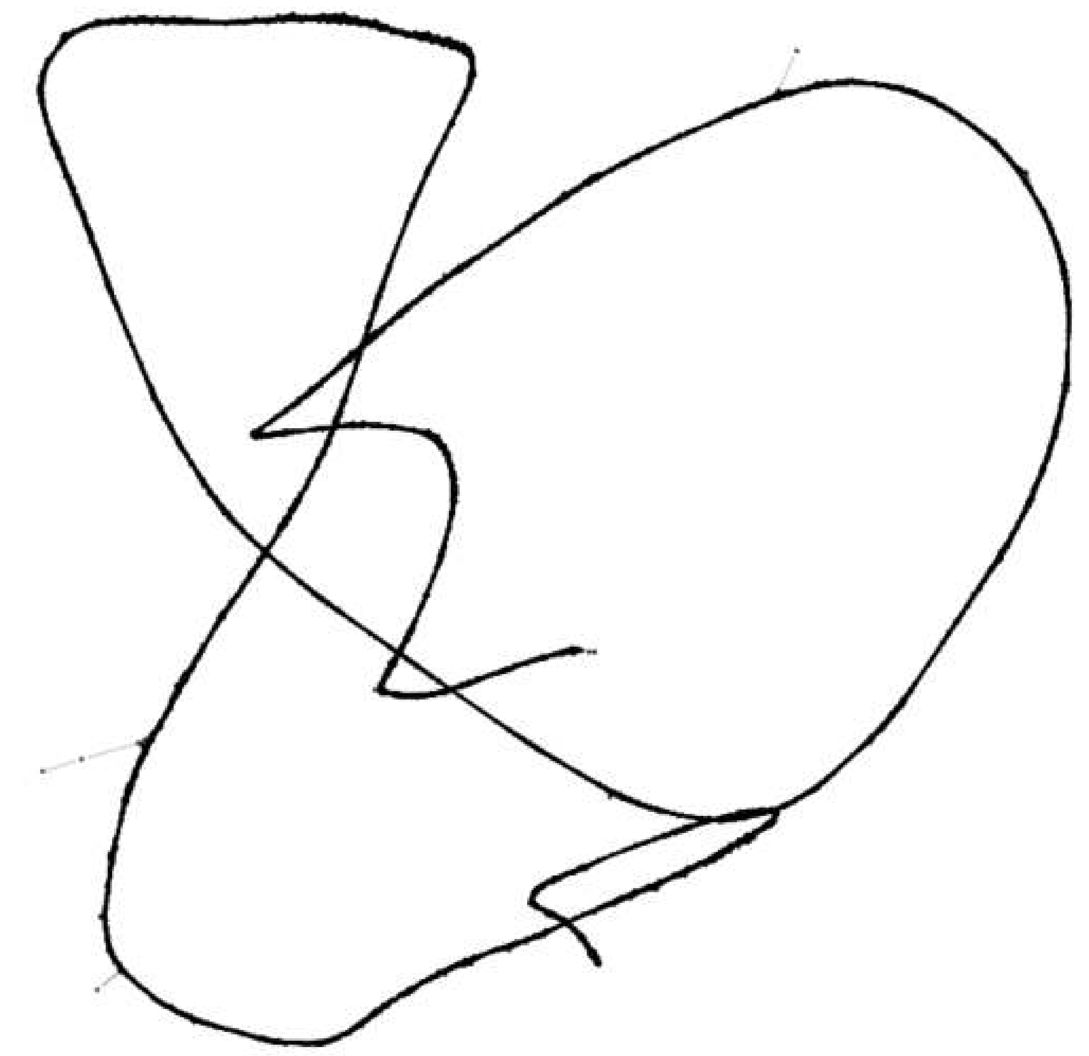

CL174
Low_complexity
Length of Reads (GP):6425 (0.08%)

Tcacao

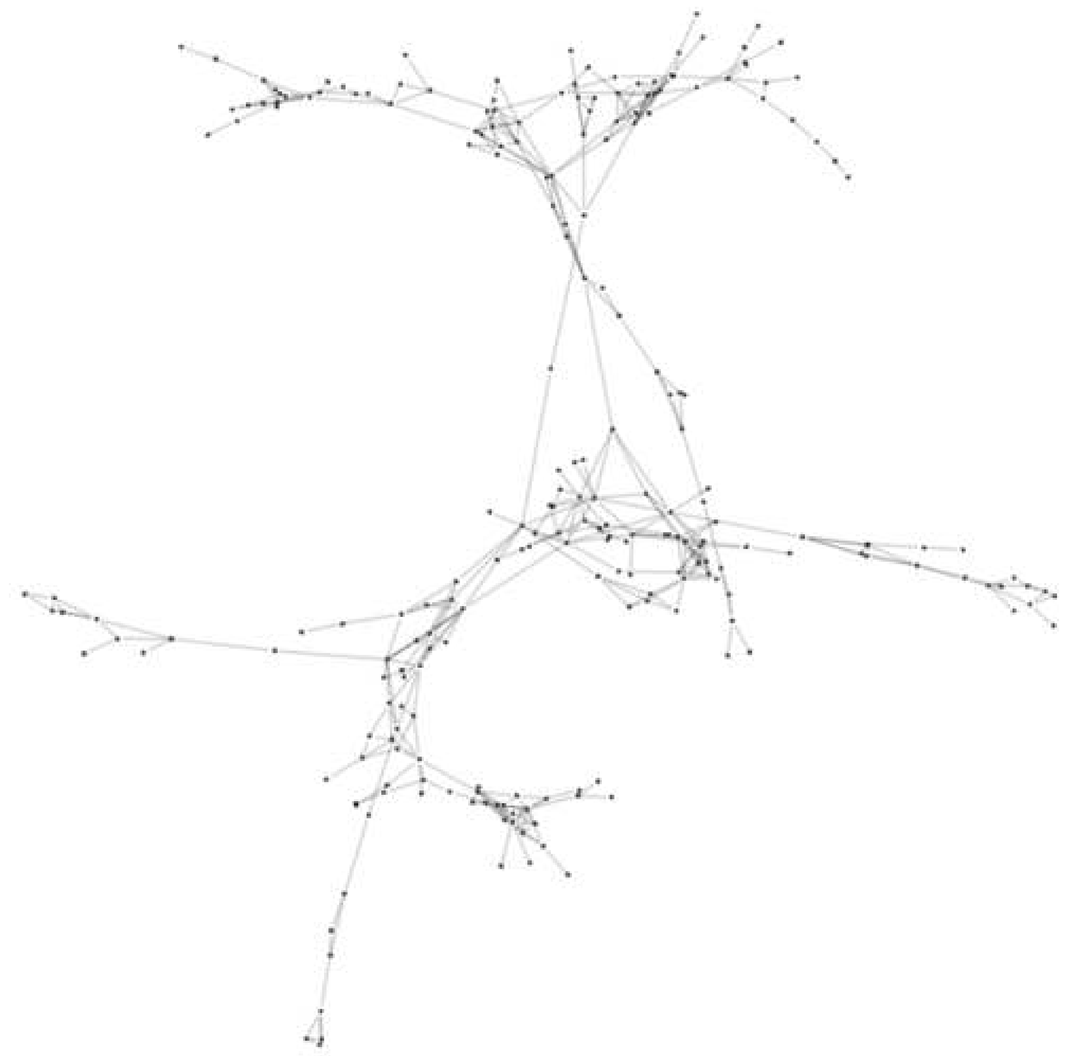

CL174
Low_complexity
Length of Reads (GP):235 (0.01%)

Tgrandiflorum

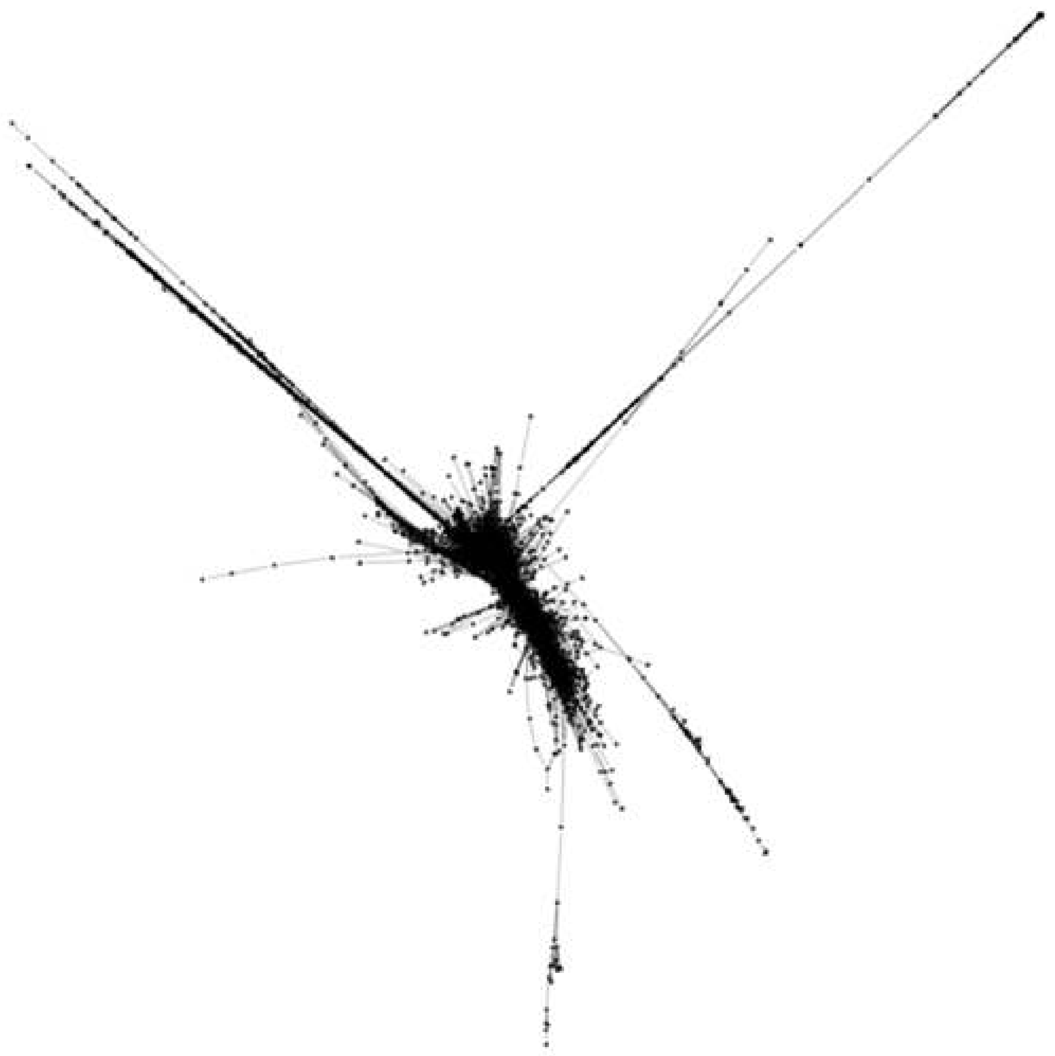

CL175
Low_complexity
Length of Reads (GP):6415 (0.08%)

Tcacao

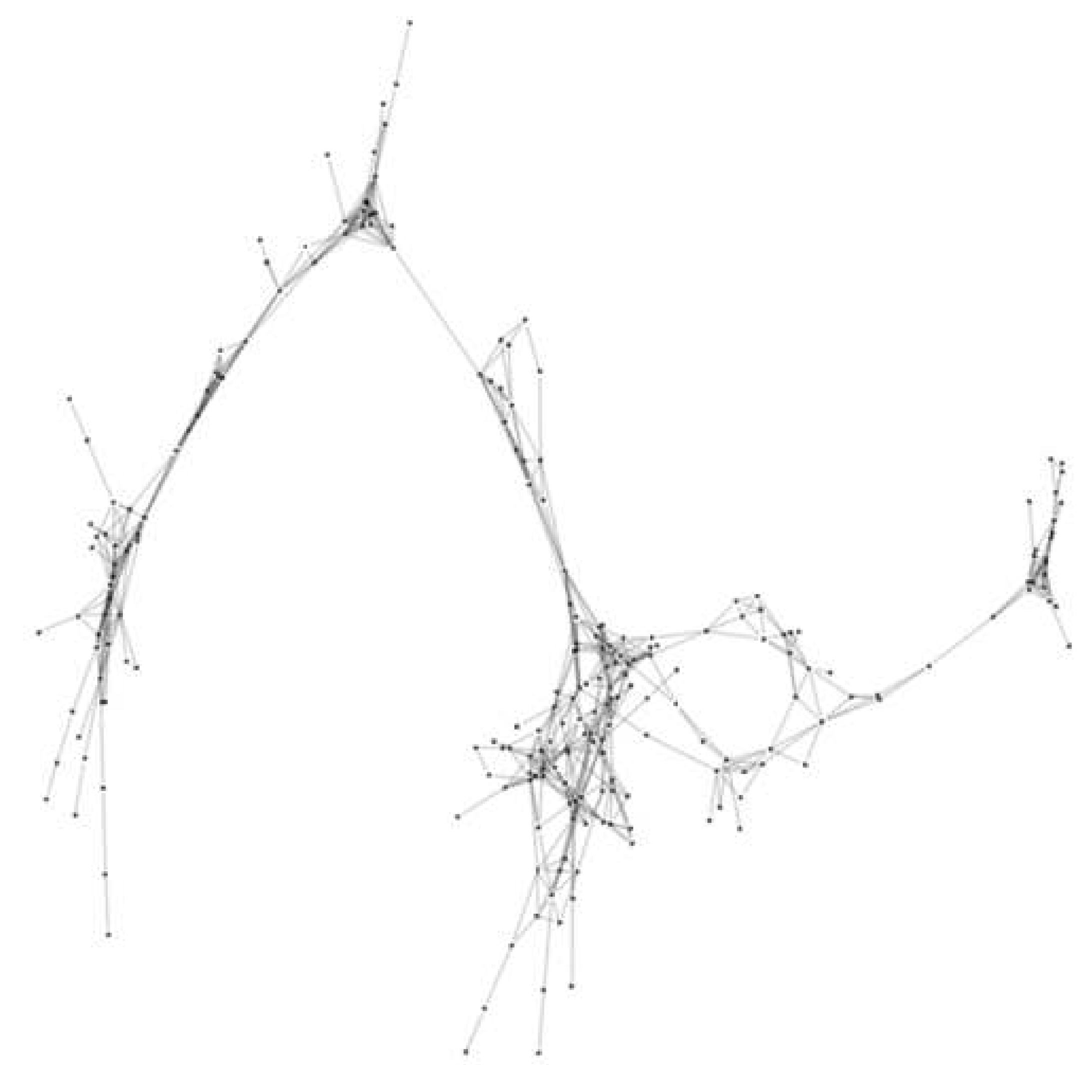

CL175
LTR_Gypsy
Length of Reads (GP):232 (0.01%)

Tgrandiflorum

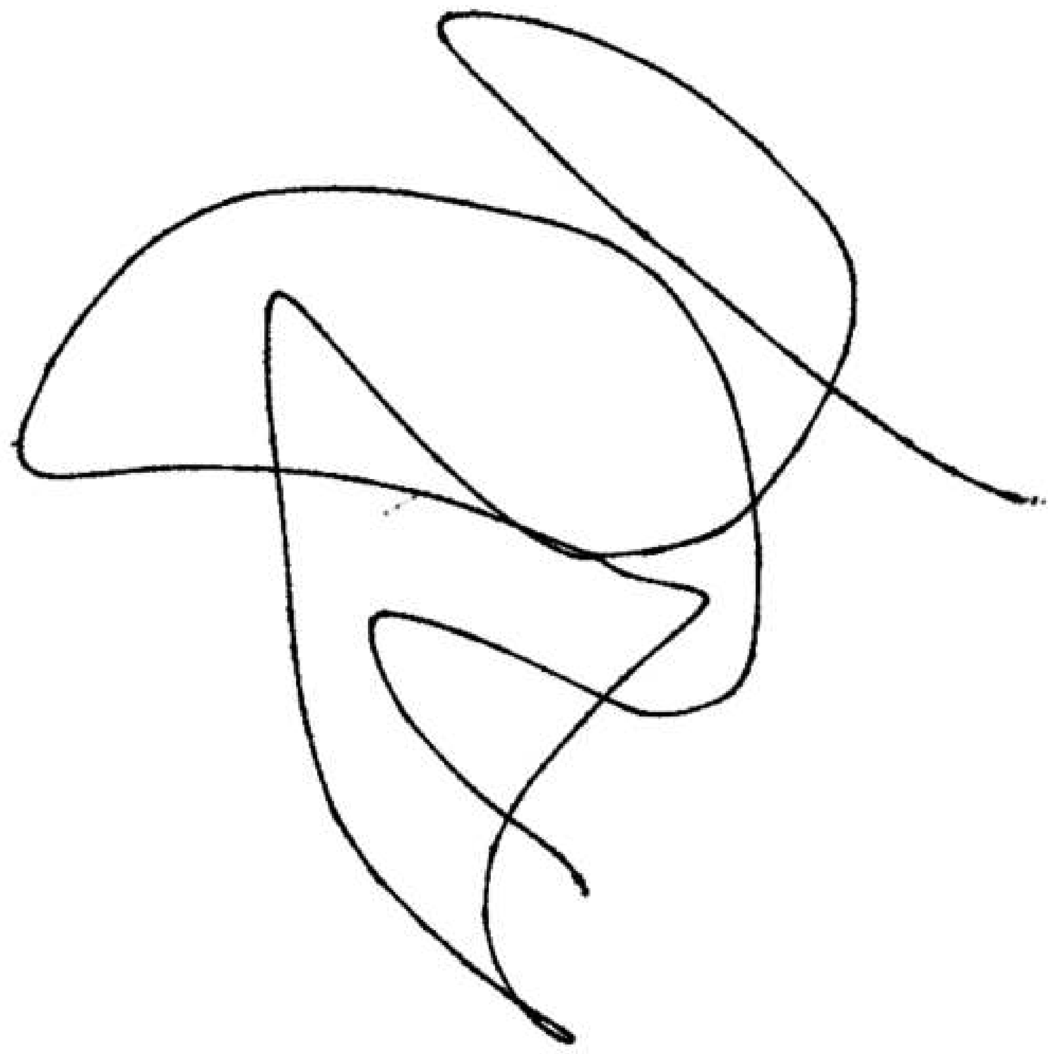

CL176
Low_complexity
Length of Reads (GP):6394 (0.08%)

Tcacao

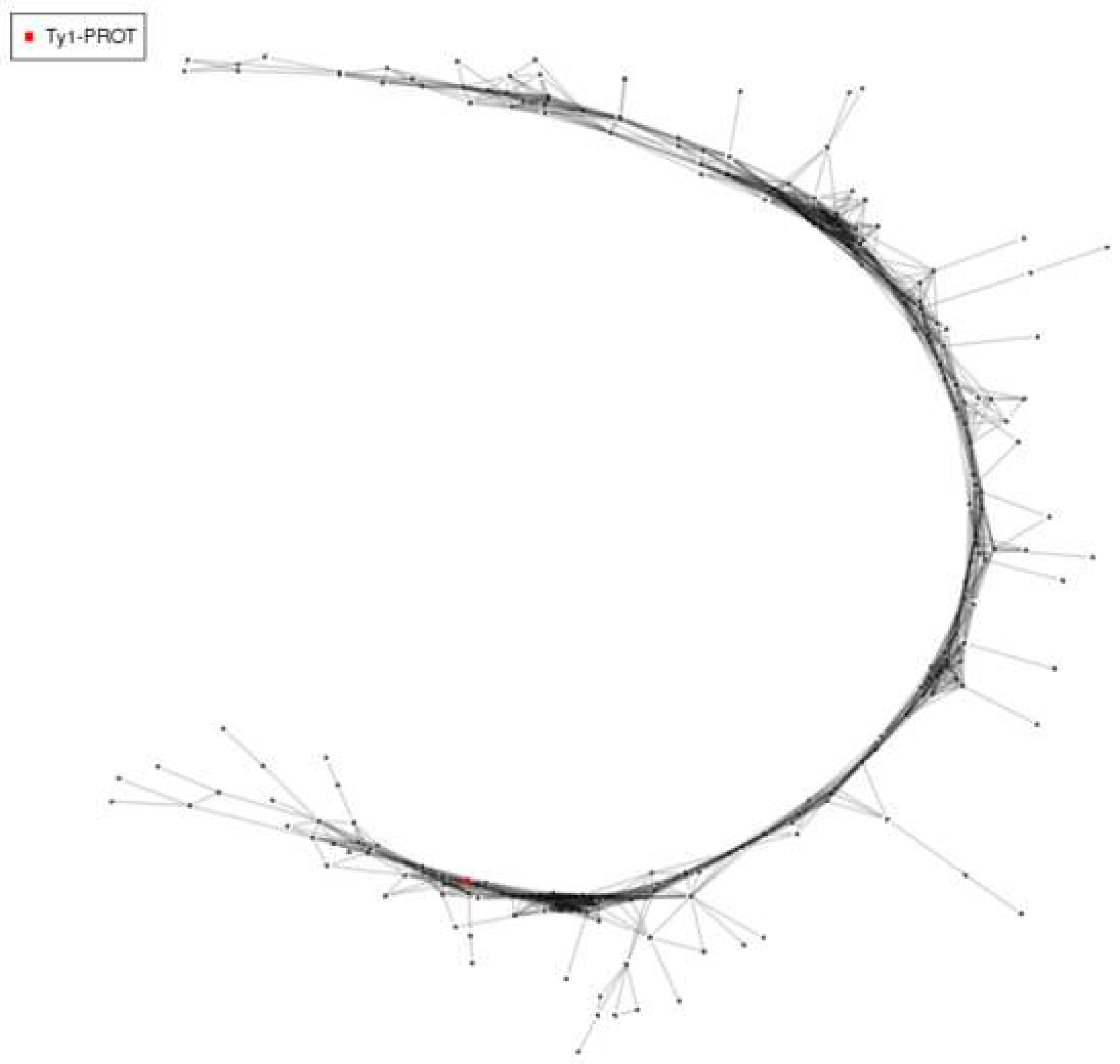

CL176
LTR_Copia
Length of Reads (GP):231 (0.01%)

Tgrandiflorum

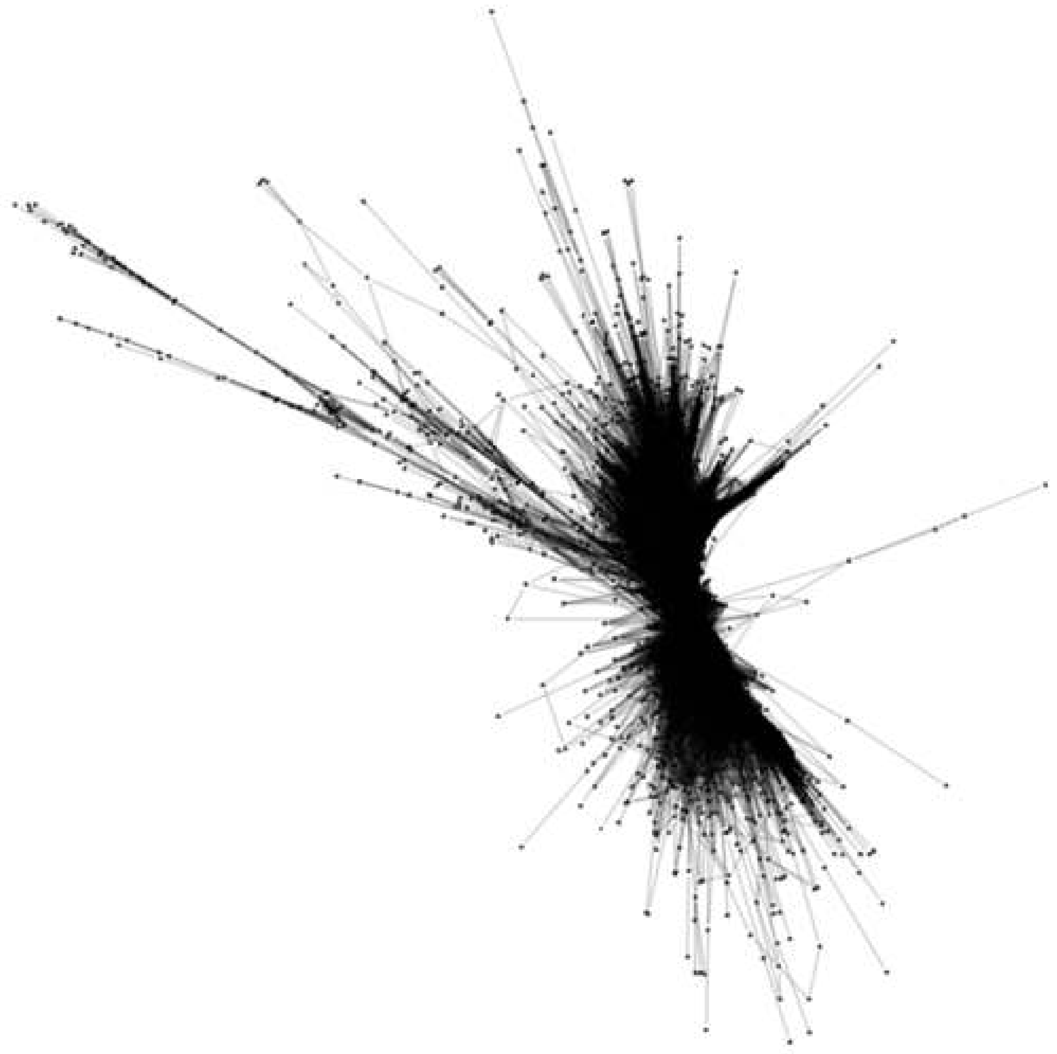

CL177
Low_complexity
Length of Reads (GP):6282 (0.08%)

Tcacao

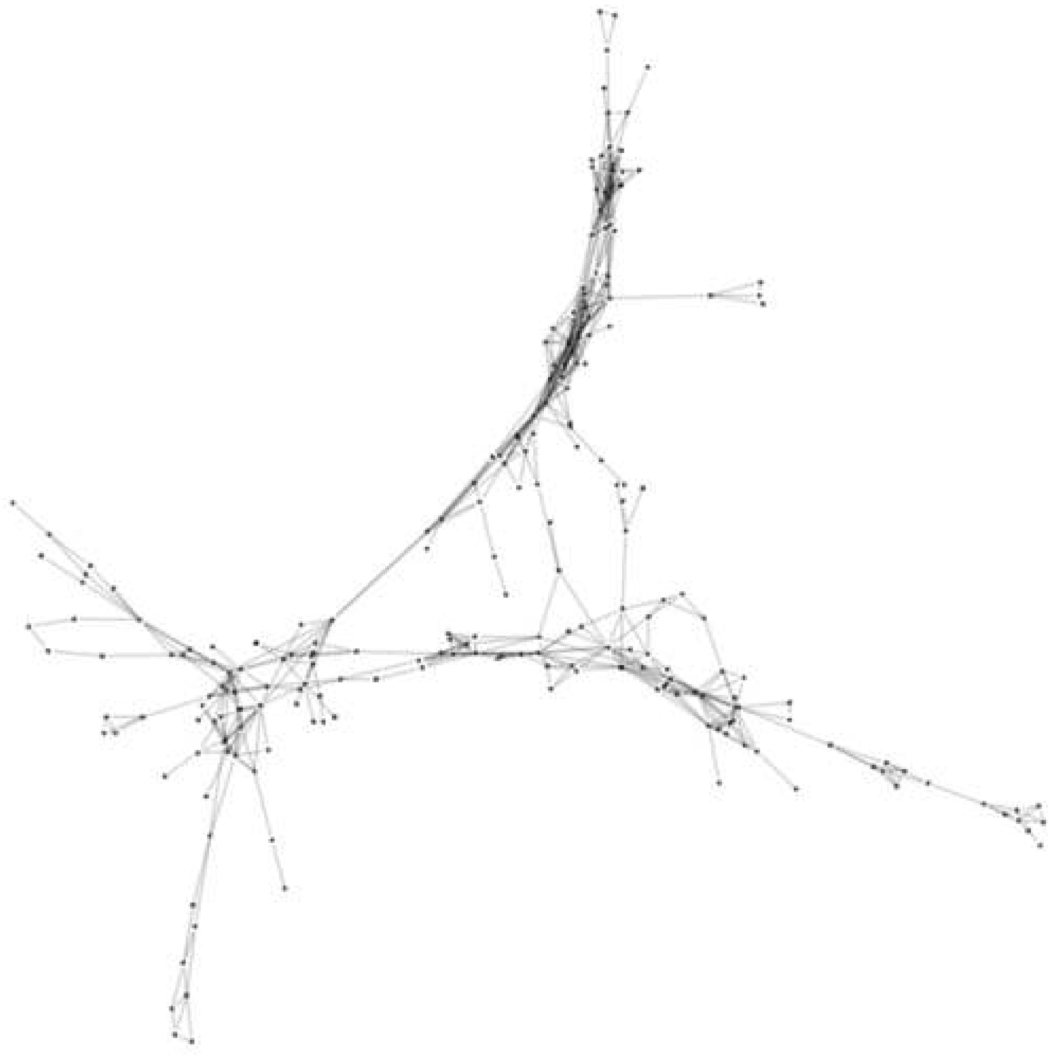

CL177
Simple_repeat
Length of Reads (GP):230 (0.01%)

Tgrandiflorum

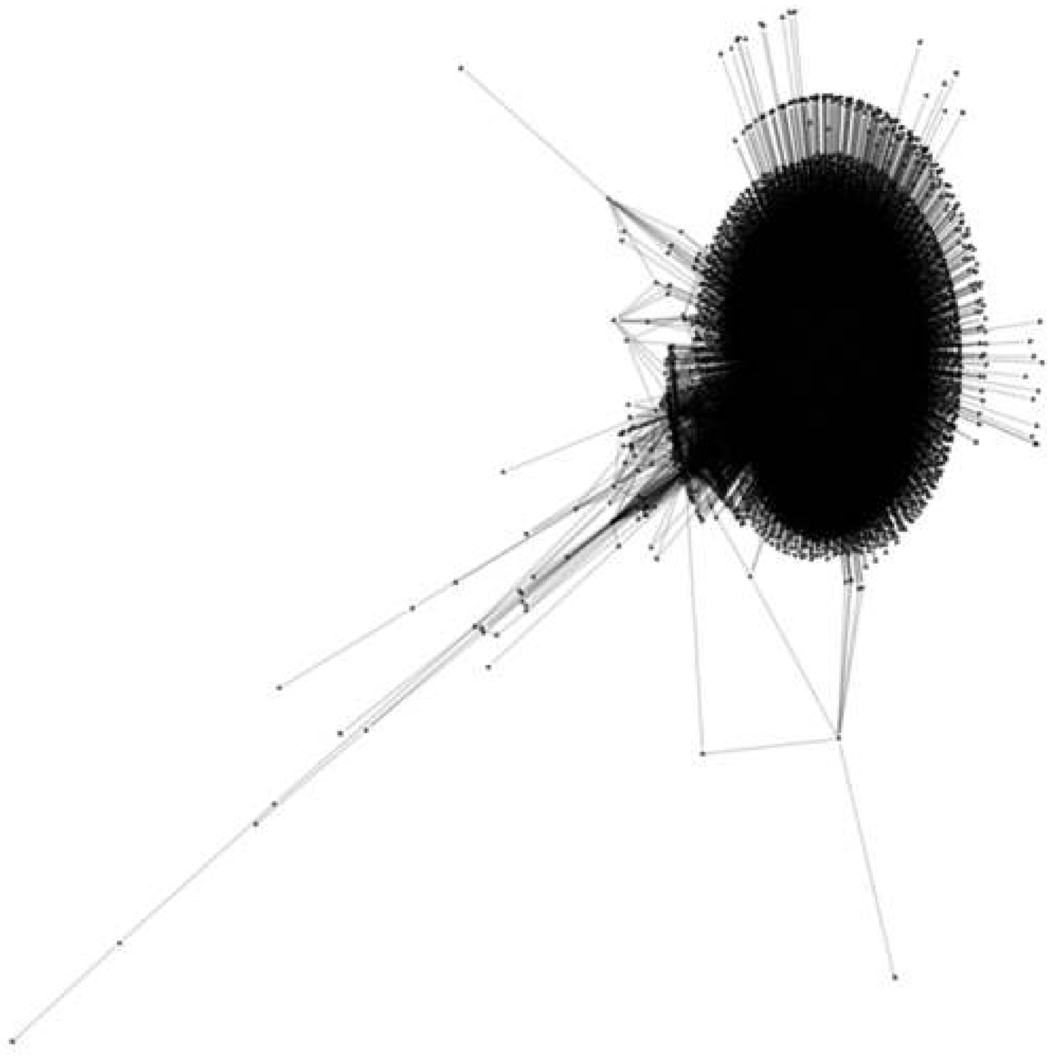

CL178
LINE_L1
Length of Reads (GP):6209 (0.08%)

Tcacao

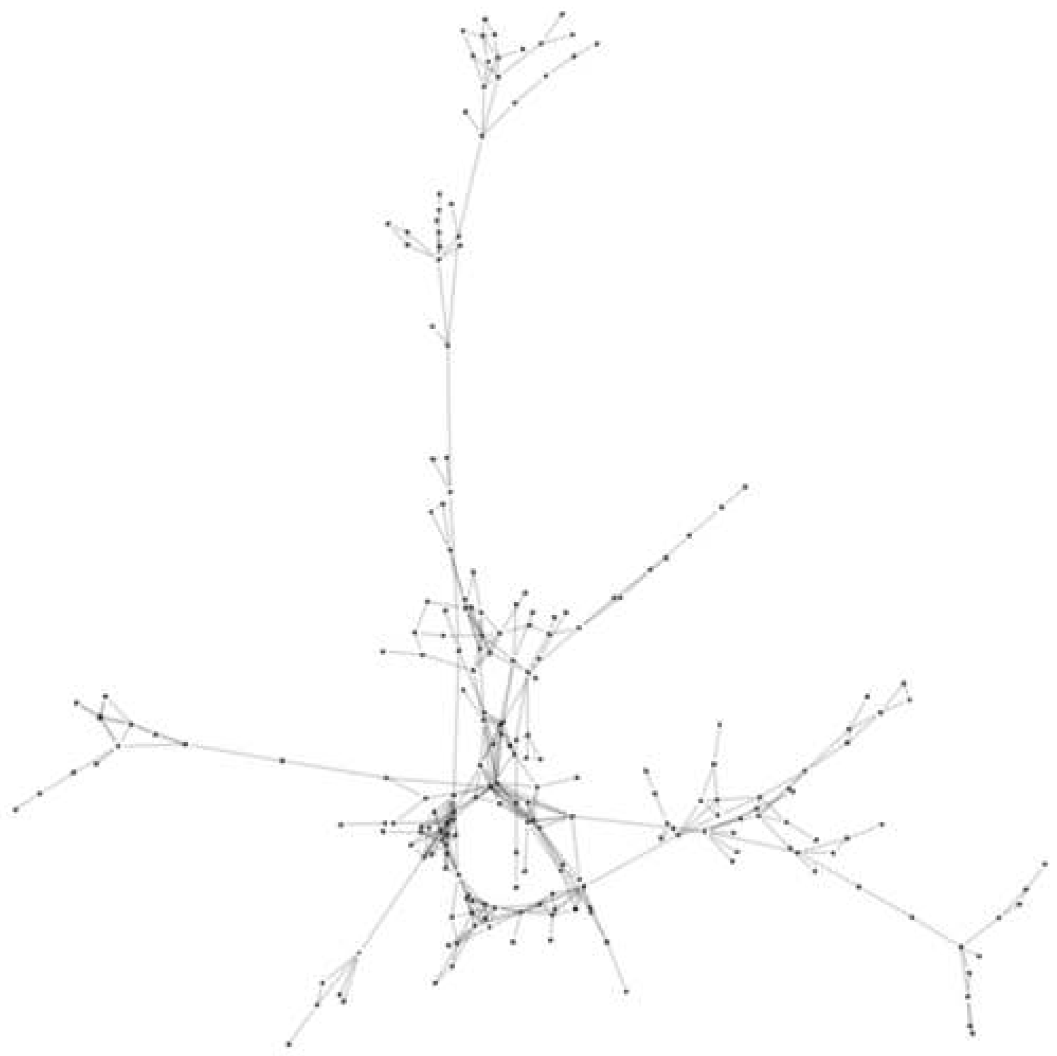

CL178
Low_complexity
Length of Reads (GP):227 (0.01%)

Tgrandiflorum

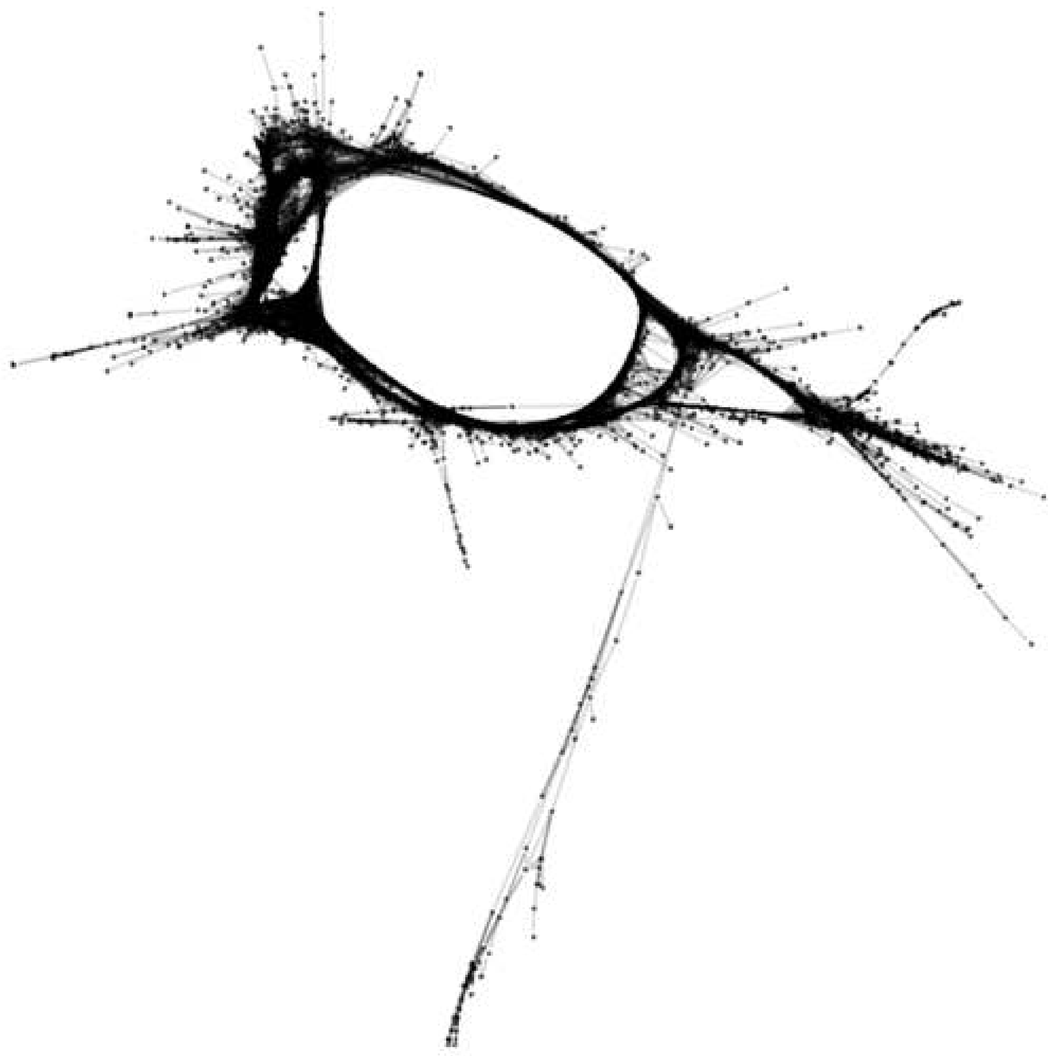

CL179
Low_complexity
Length of Reads (GP):6032 (0.08%)

Tcacao

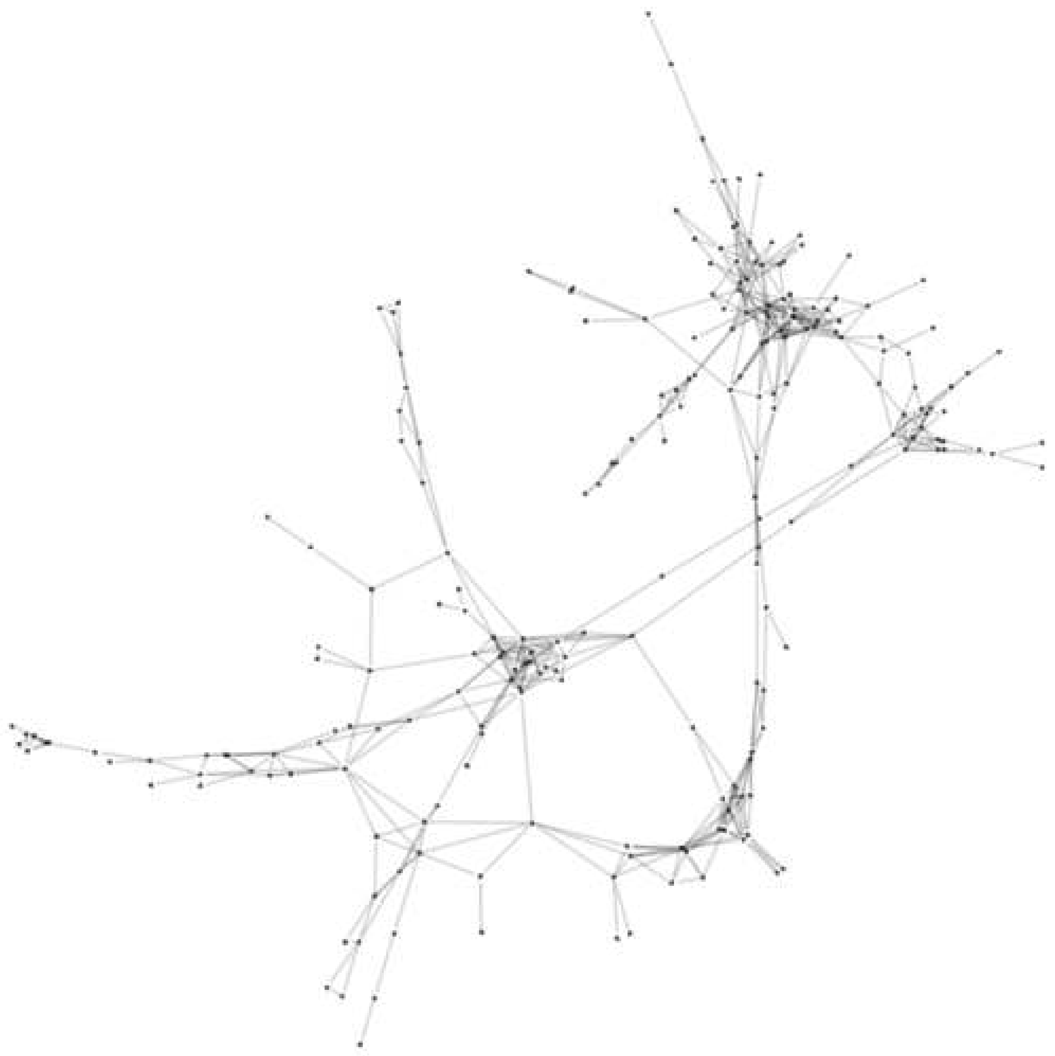

CL179
Low_complexity
Length of Reads (GP):225 (0.01%)

Tgrandiflorum

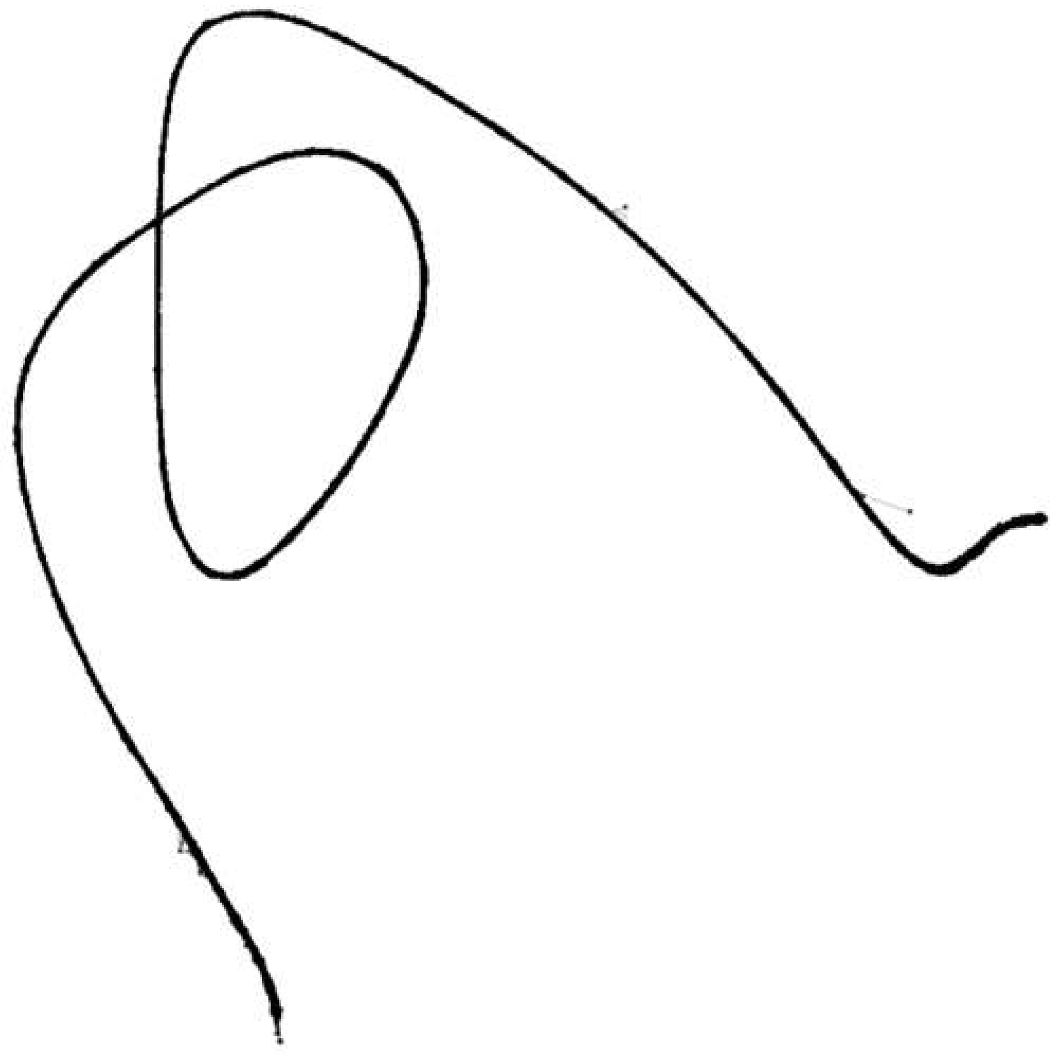

CL180
LTR_Copia
Length of Reads (GP):6004 (0.08%)

Tcacao

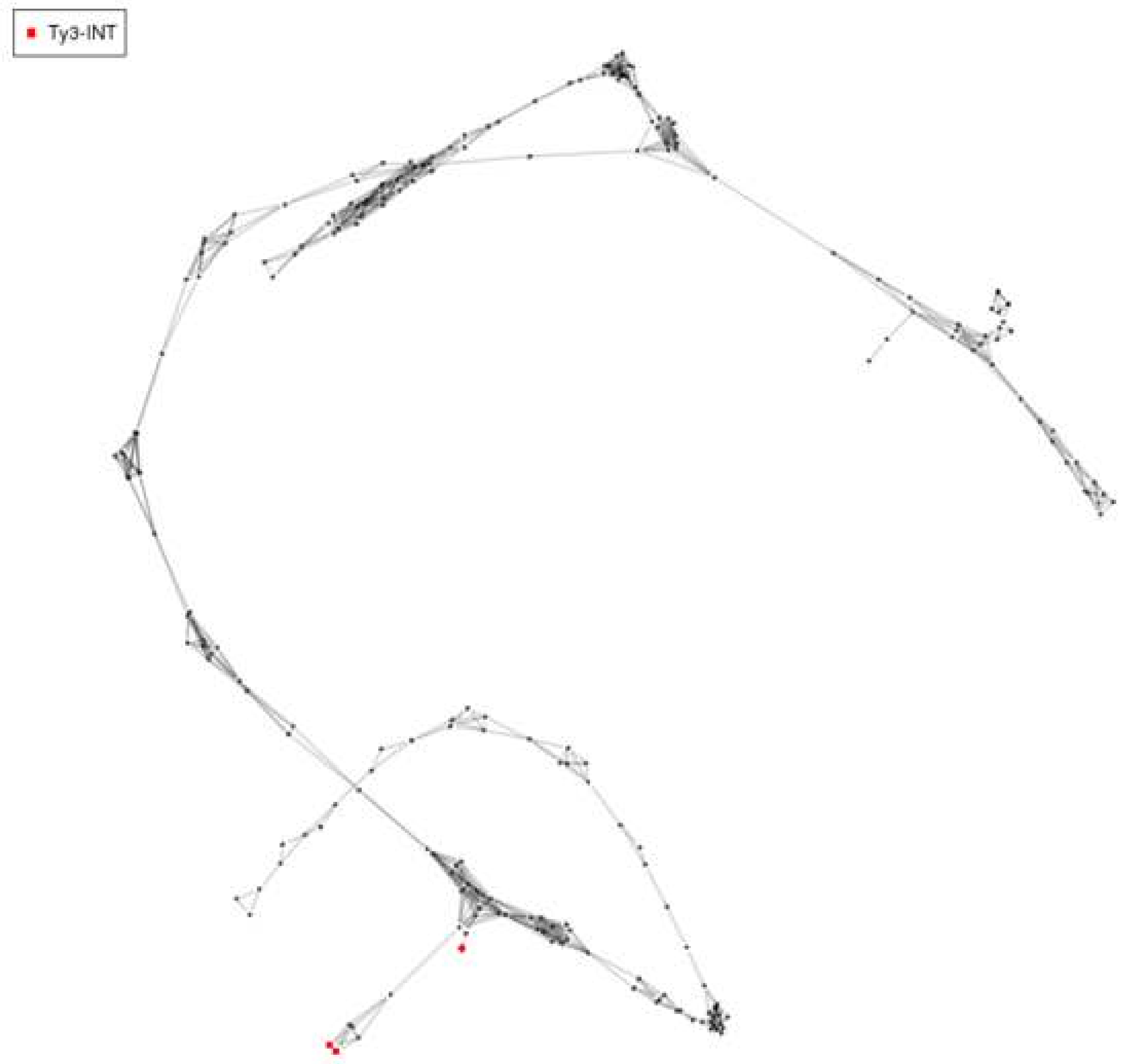

CL180
LTR_Gypsy
Length of Reads (GP):221 (0.01%)

Tgrandiflorum

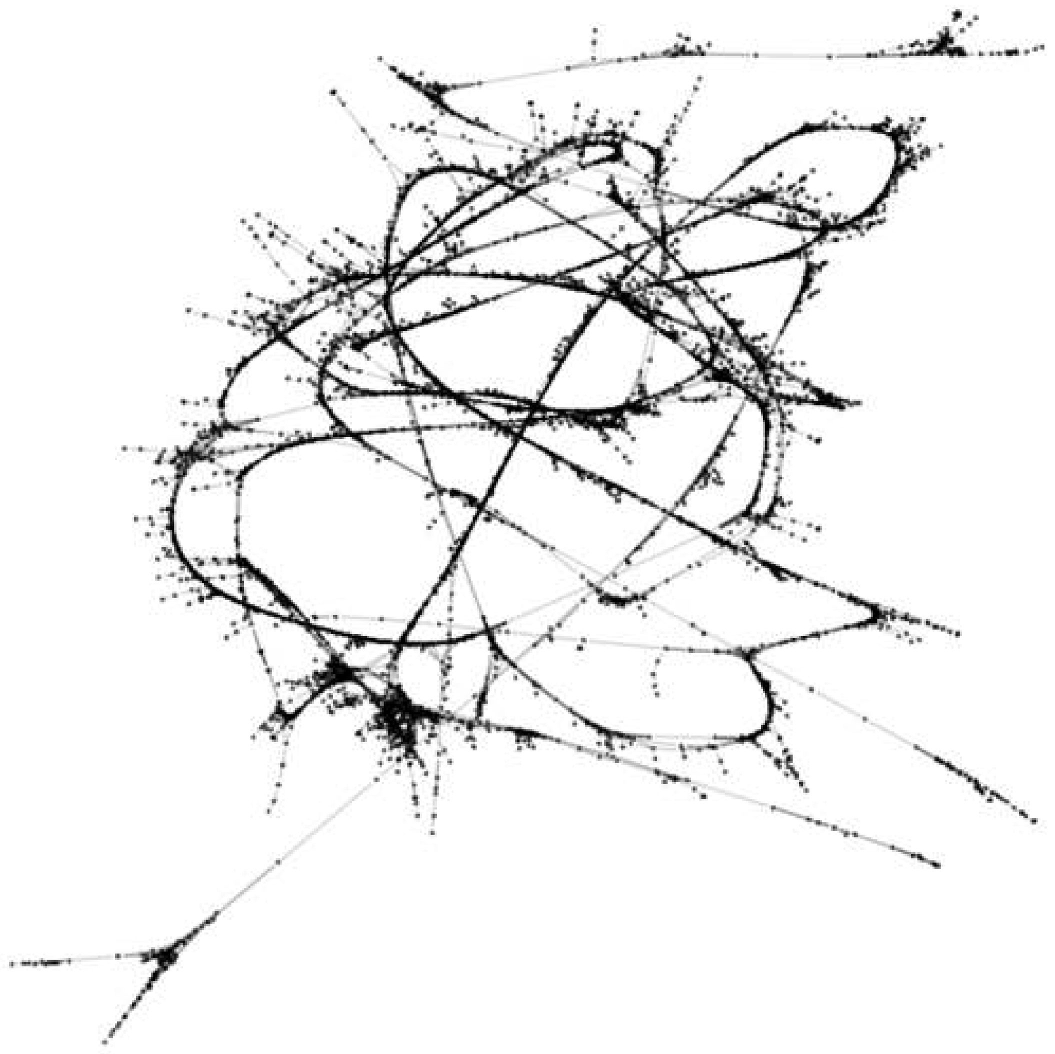

CL181
Low_complexity
Length of Reads (GP):5927 (0.07%)

Tcacao

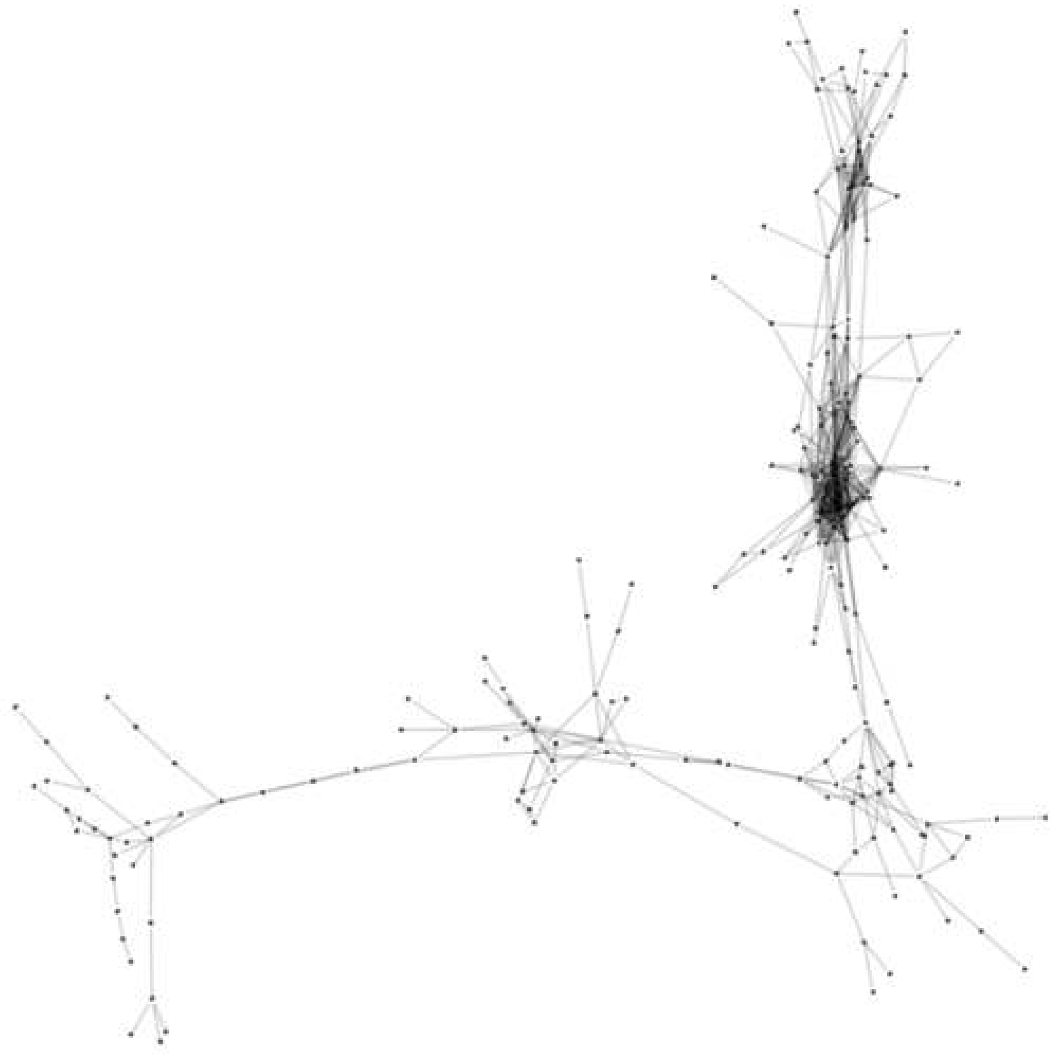

CL181
Low_complexity
Length of Reads (GP):221 (0.01%)

Tgrandiflorum

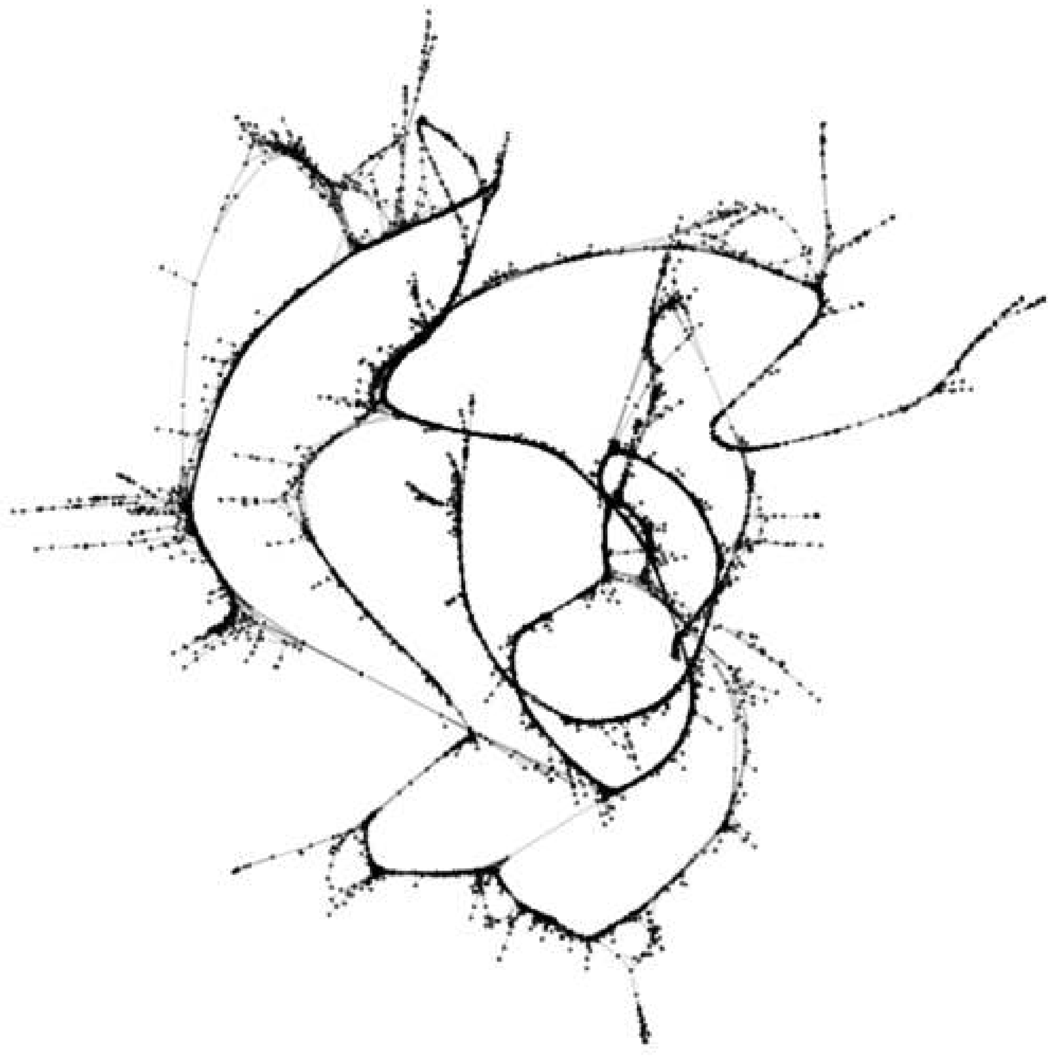

CL182
DNA_hAT_Ac
Length of Reads (GP):5858 (0.07%)

Tcacao

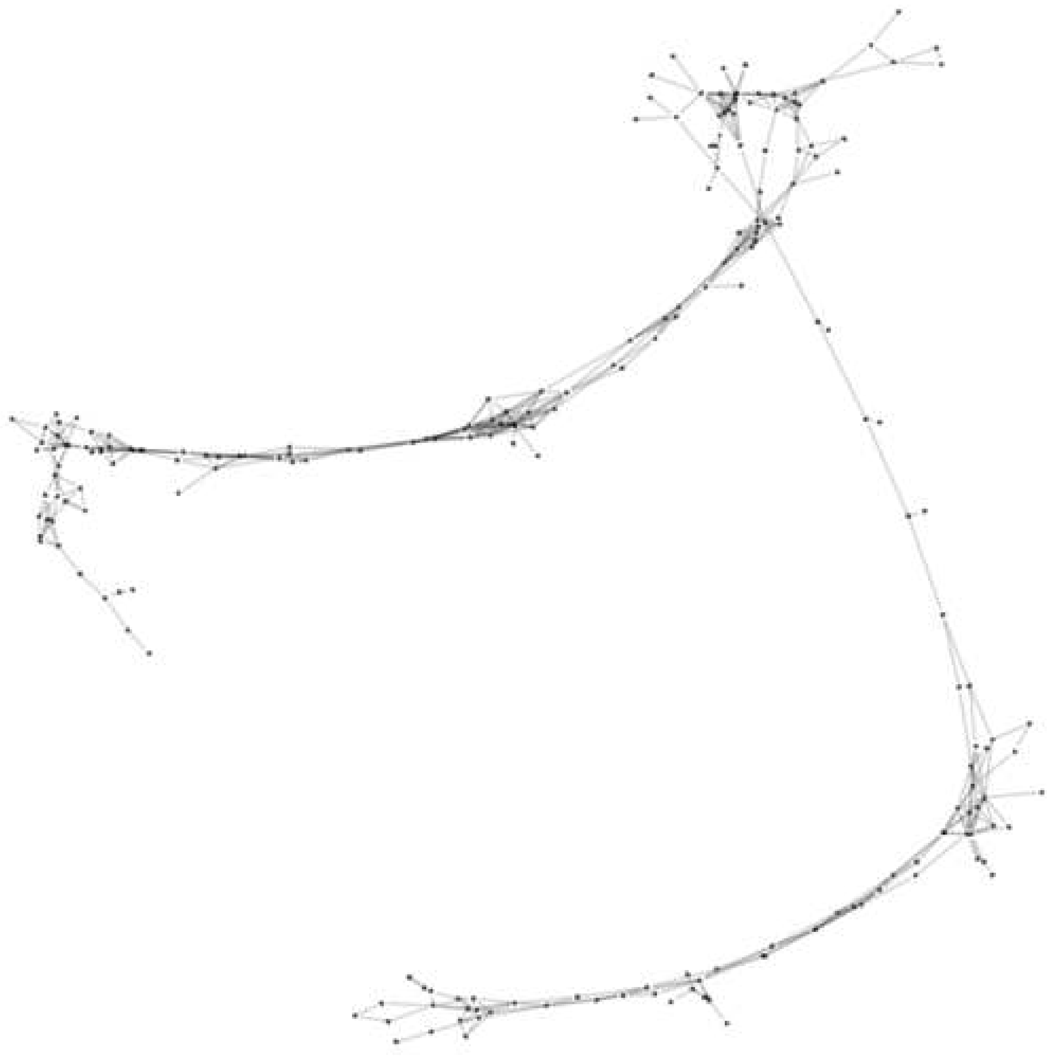

CL182
Low_complexity
Length of Reads (GP):217 (0.01%)

Tgrandiflorum

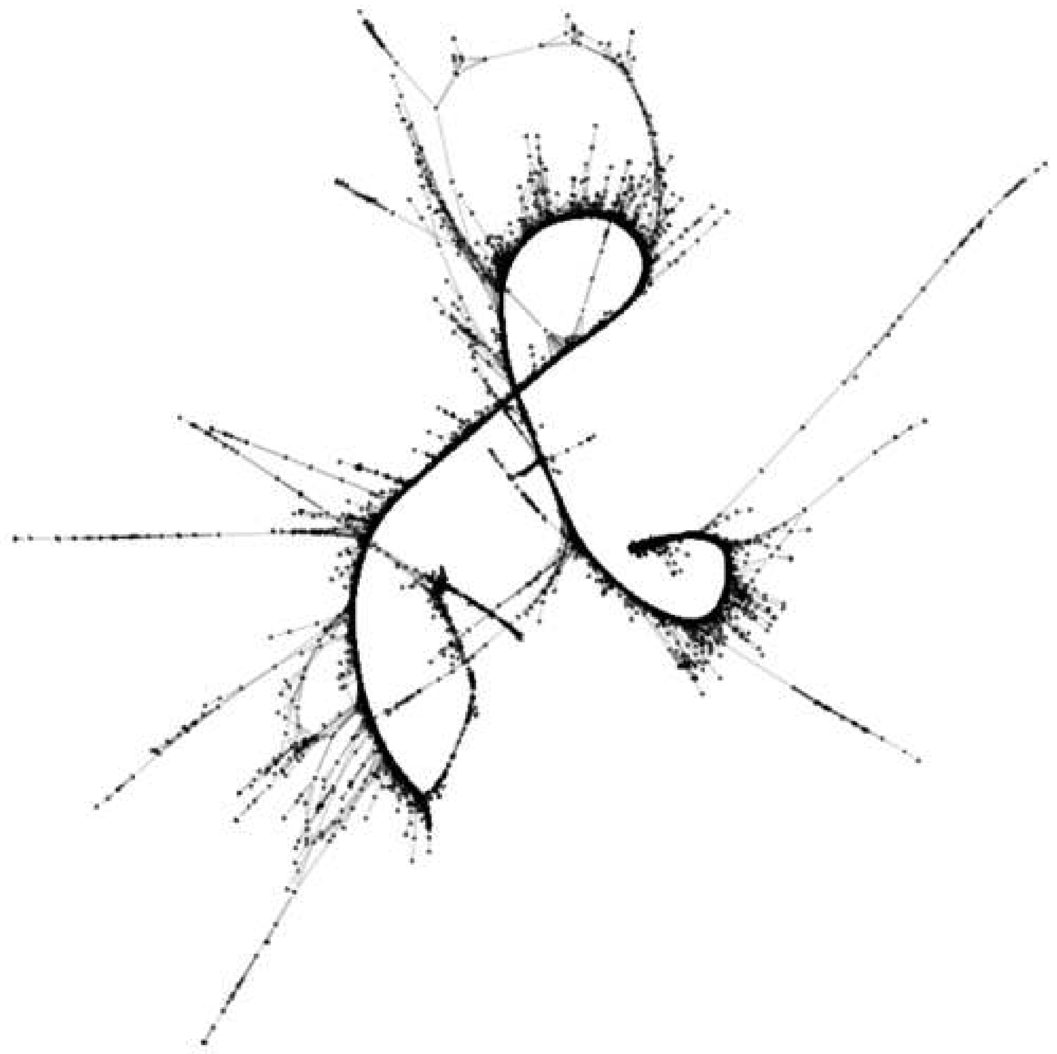

CL183
Low_complexity
Length of Reads (GP):5787 (0.07%)

Tcacao

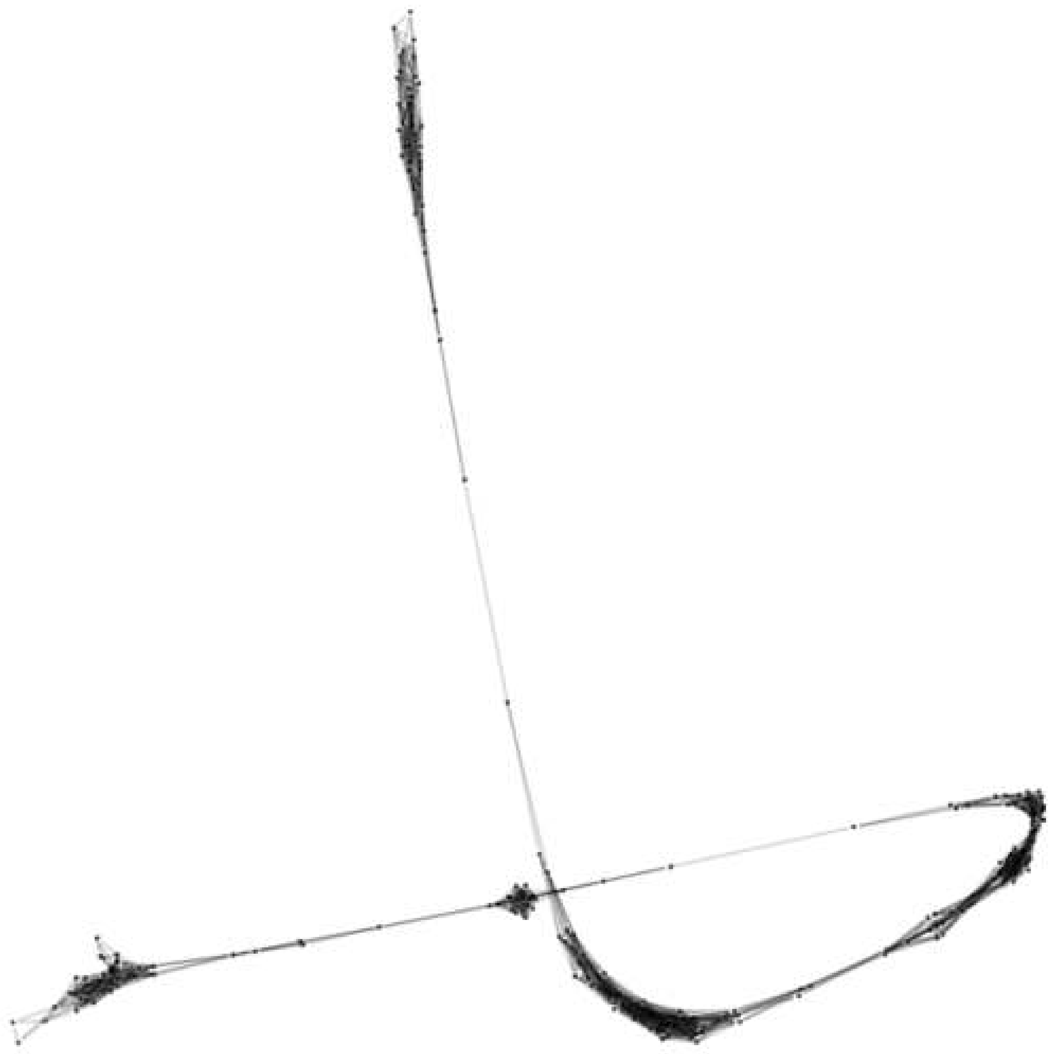

CL183
LTR_Gypsy
Length of Reads (GP):215 (0.01%)

Tgrandiflorum

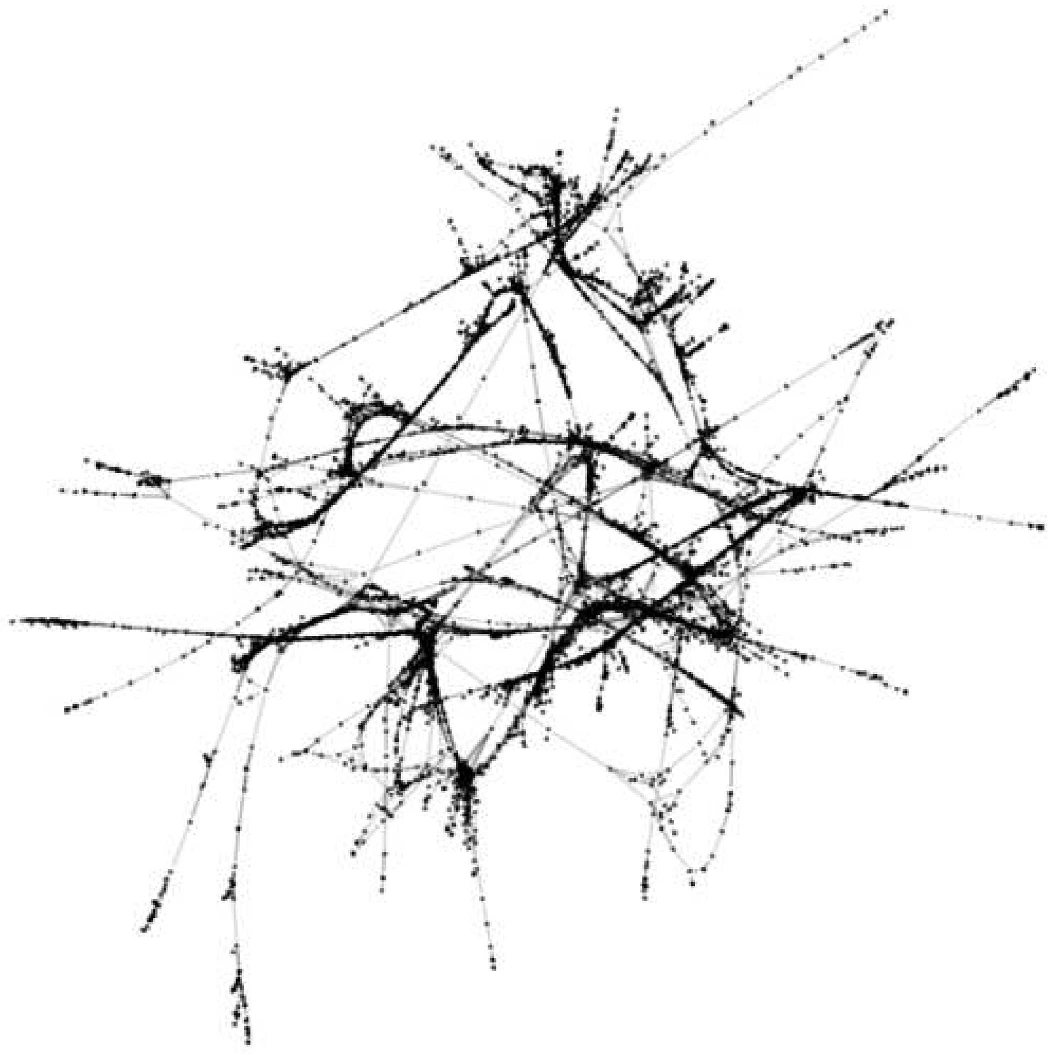

CL184
Low_complexity
Length of Reads (GP):5713 (0.07%)

Tcacao

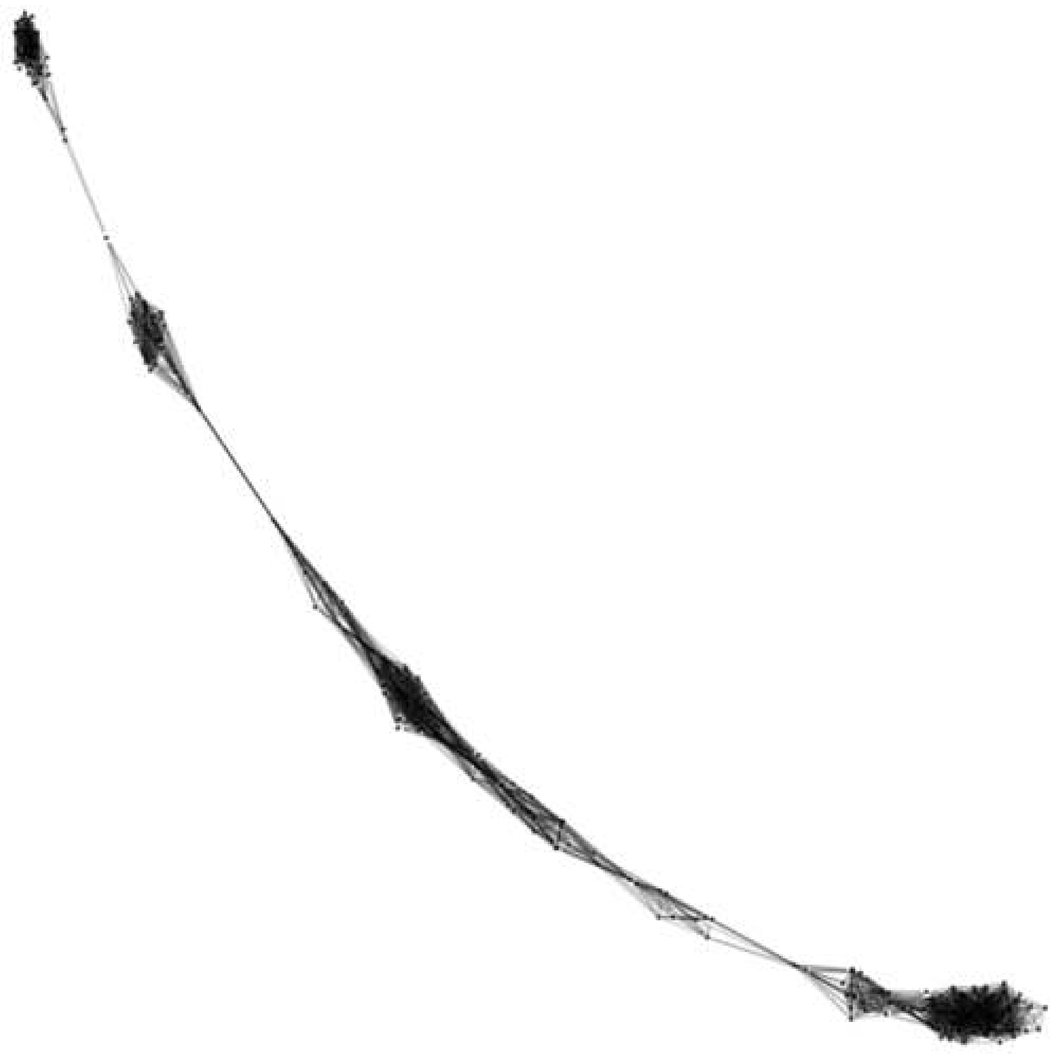

CL184
rRNA
Length of Reads (GP):214 (0.01%)

Tgrandiflorum

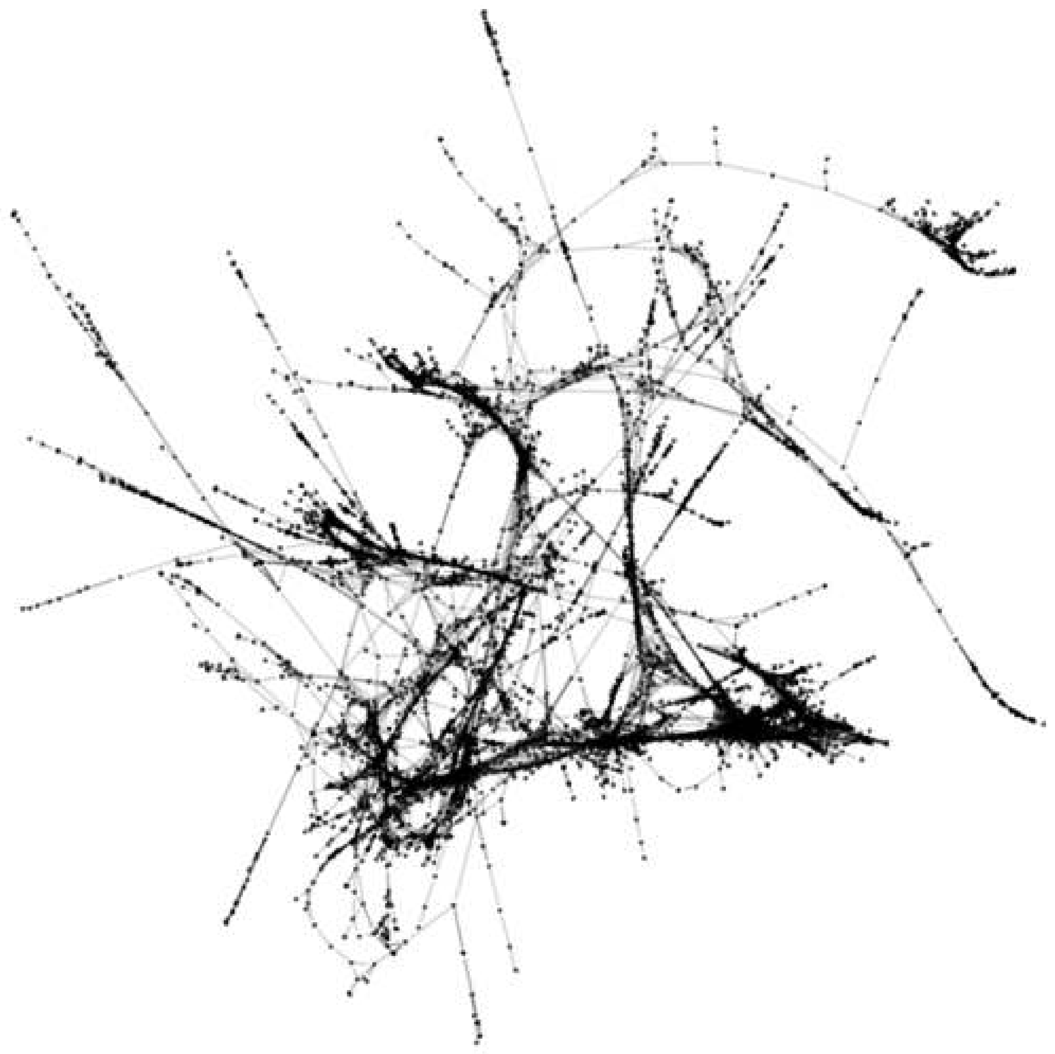

CL185
Low_complexity
Length of Reads (GP):5711 (0.07%)

Tgrandiflorum

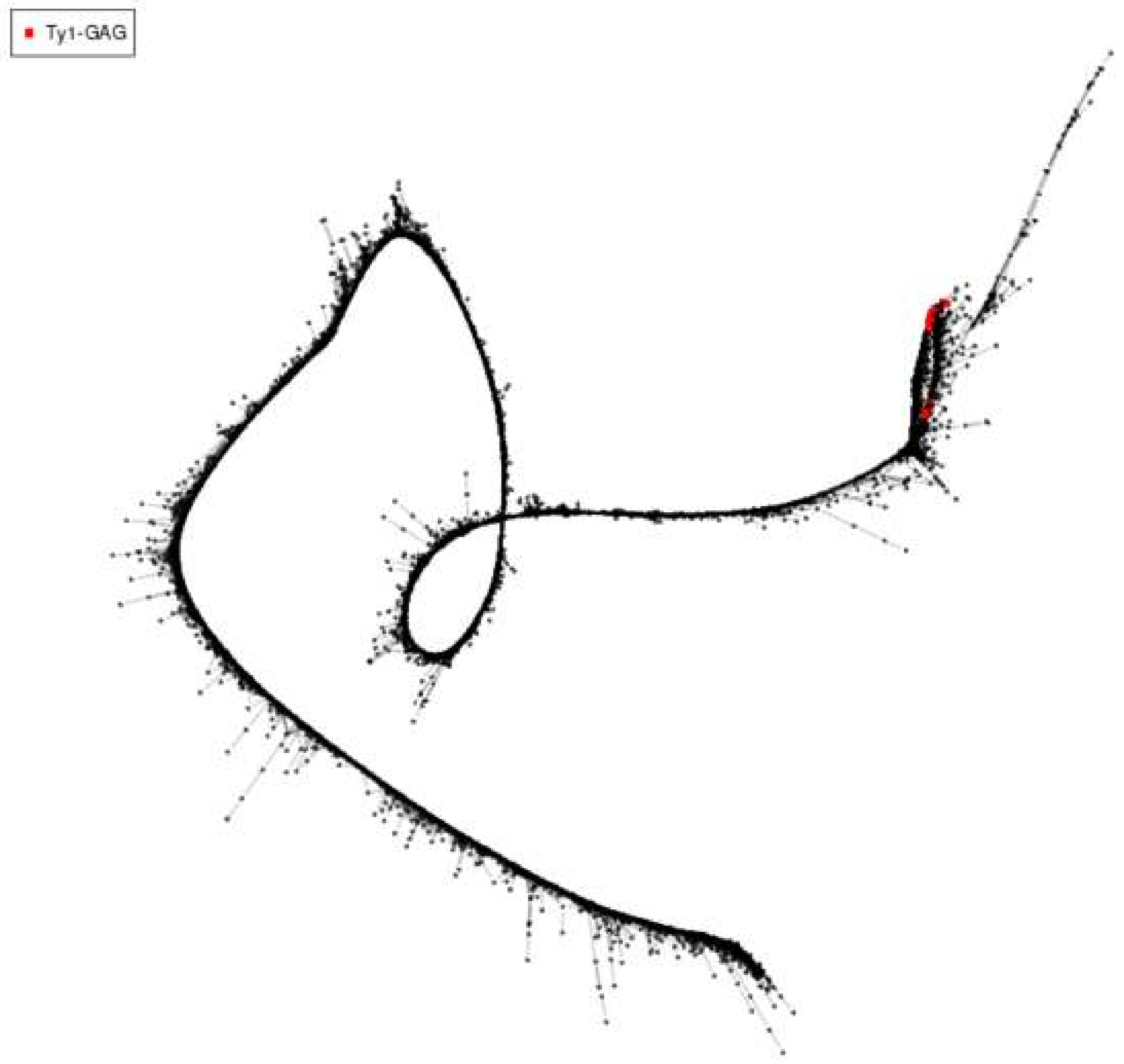

CL186
LTR_Copia
Length of Reads (GP):5684 (0.07%)

Tgrandiflorum

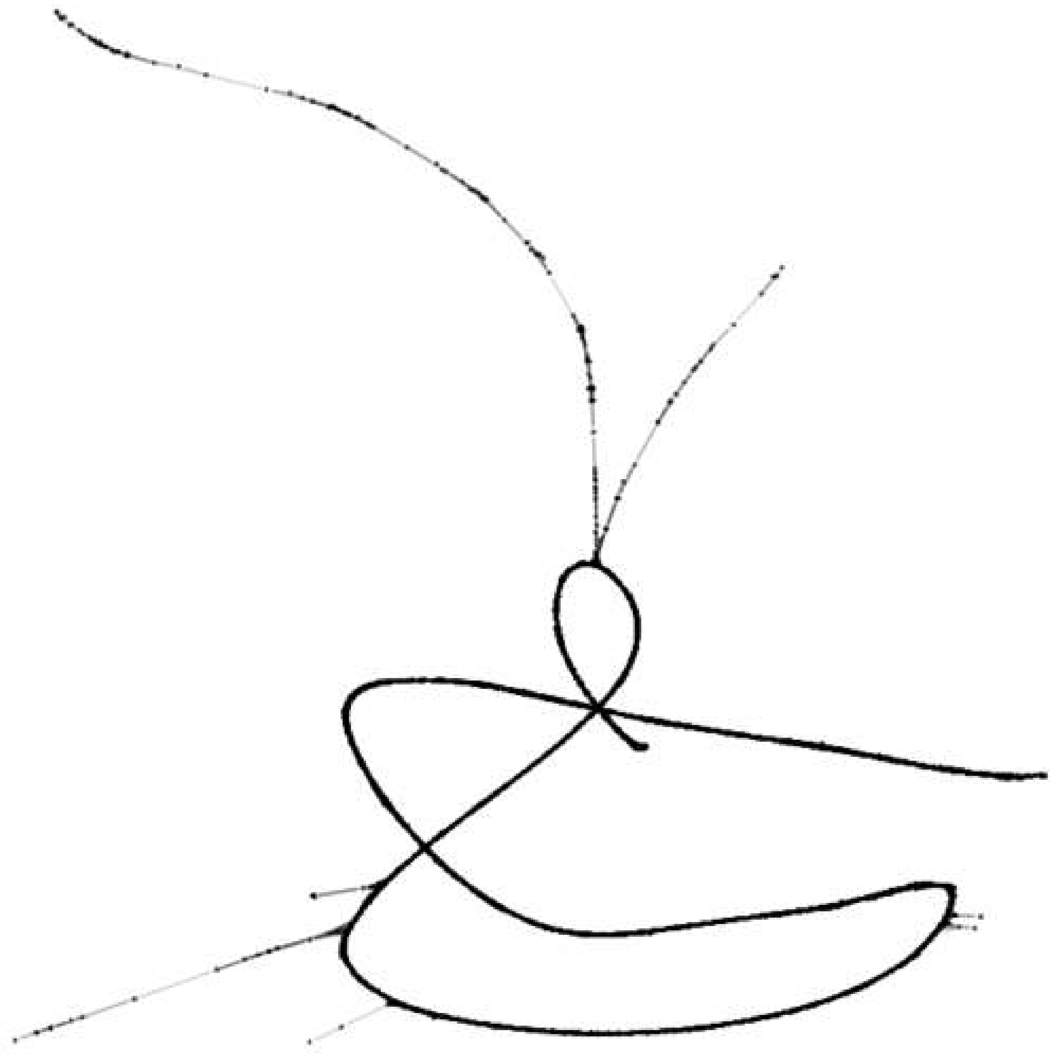

CL187
rRNA
Length of Reads (GP):5652 (0.07%)

Tgrandiflorum

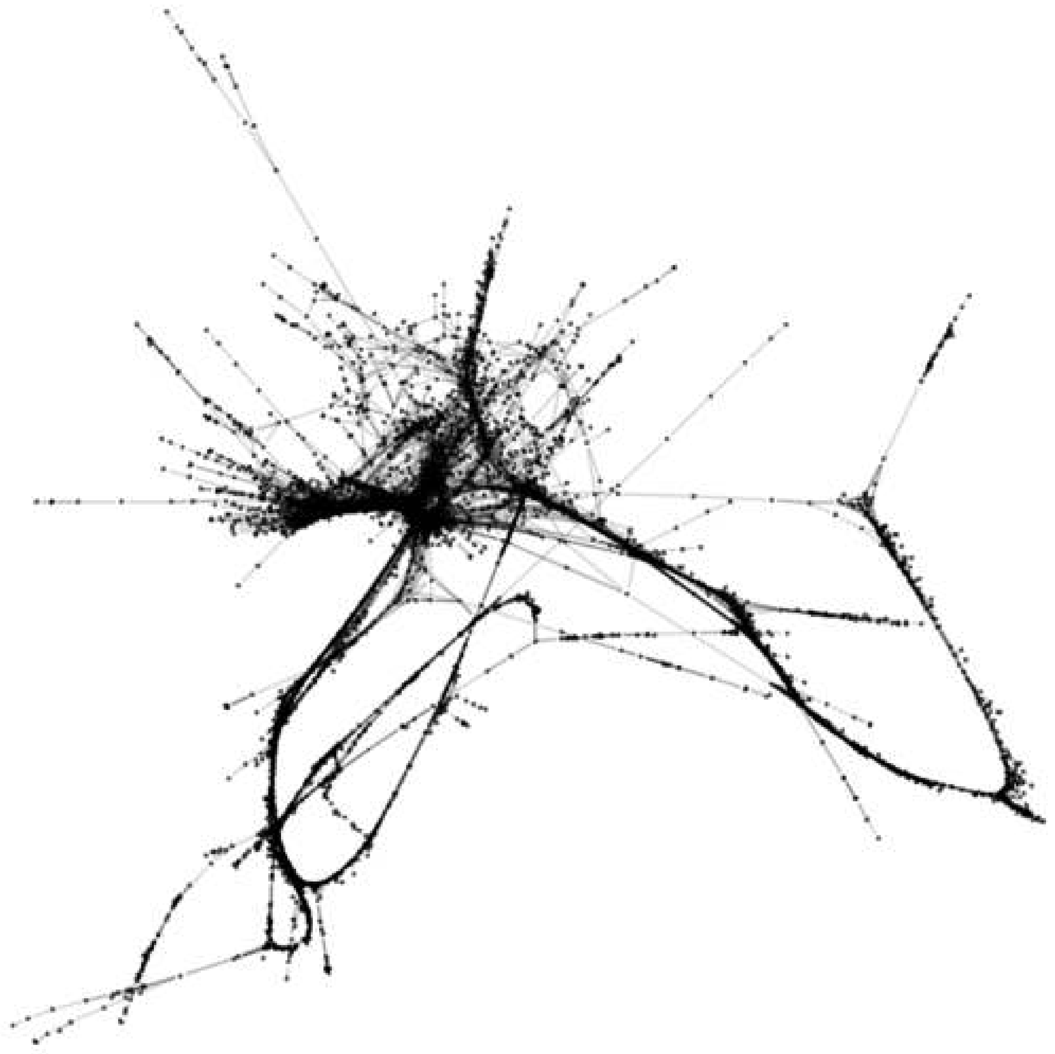

CL188
Low_complexity
Length of Reads (GP):5652 (0.07%)

Tgrandiflorum

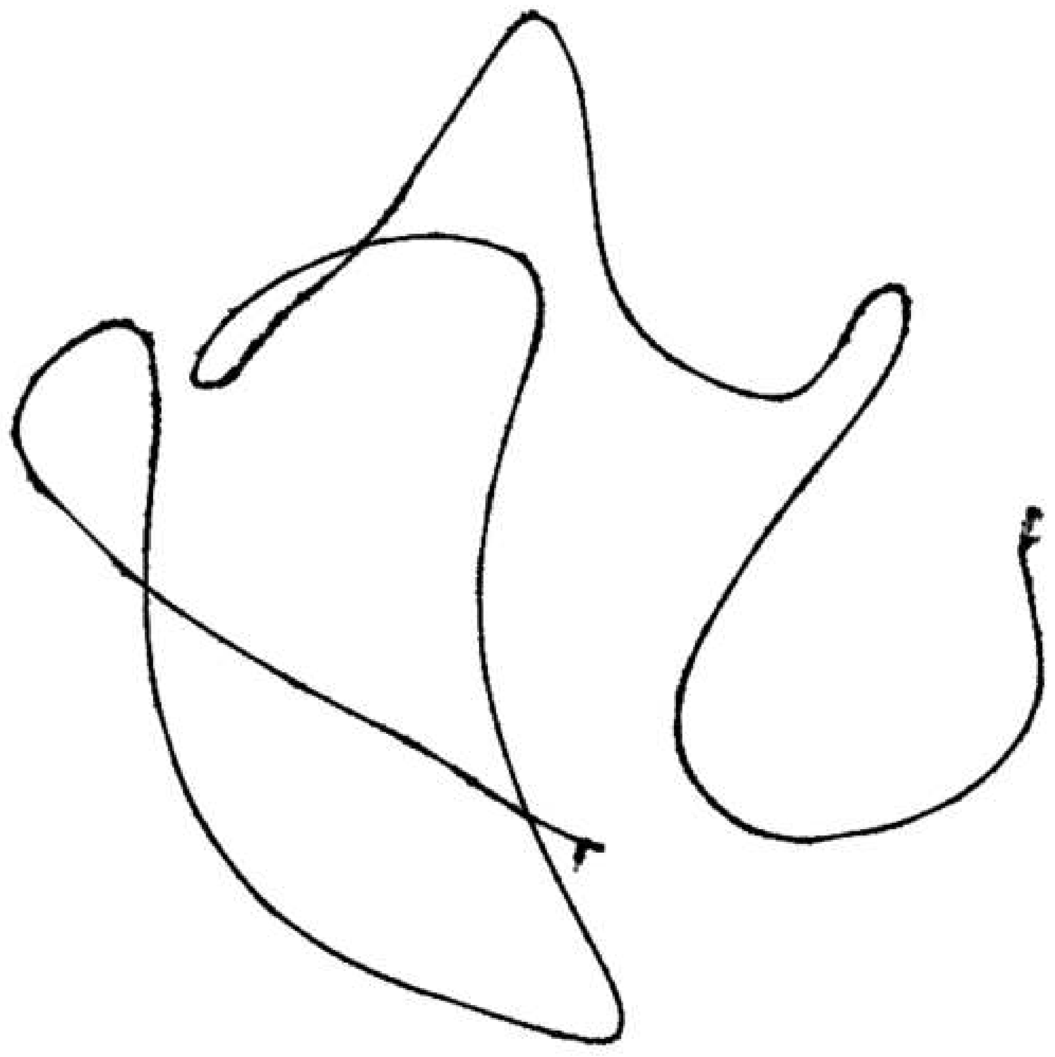

CL189
Low_complexity
Length of Reads (GP):5644 (0.07%)

Tgrandiflorum

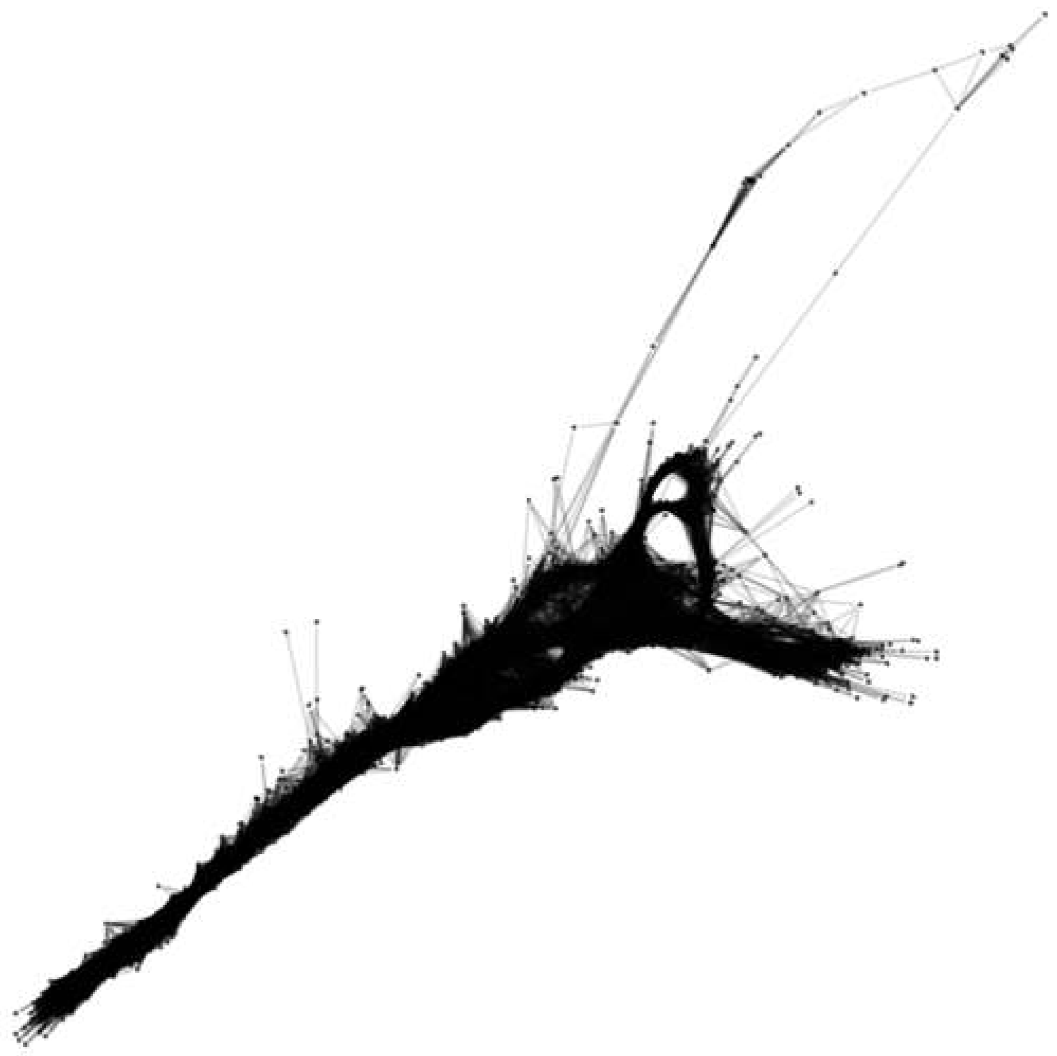

CL190
Low_complexity
Length of Reads (GP):5577 (0.07%)

Tgrandiflorum

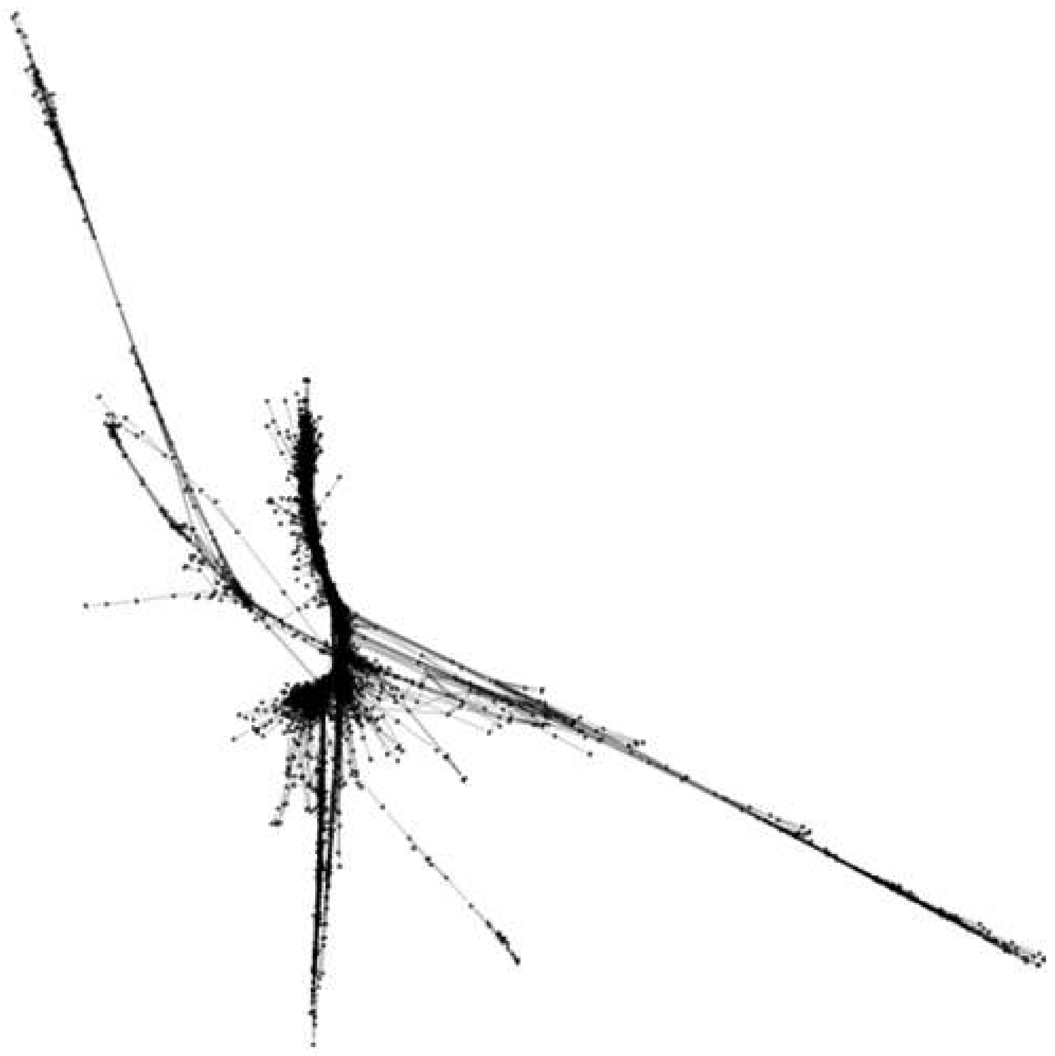

CL191
Low_complexity
Length of Reads (GP):5426 (0.07%)

Tgrandiflorum

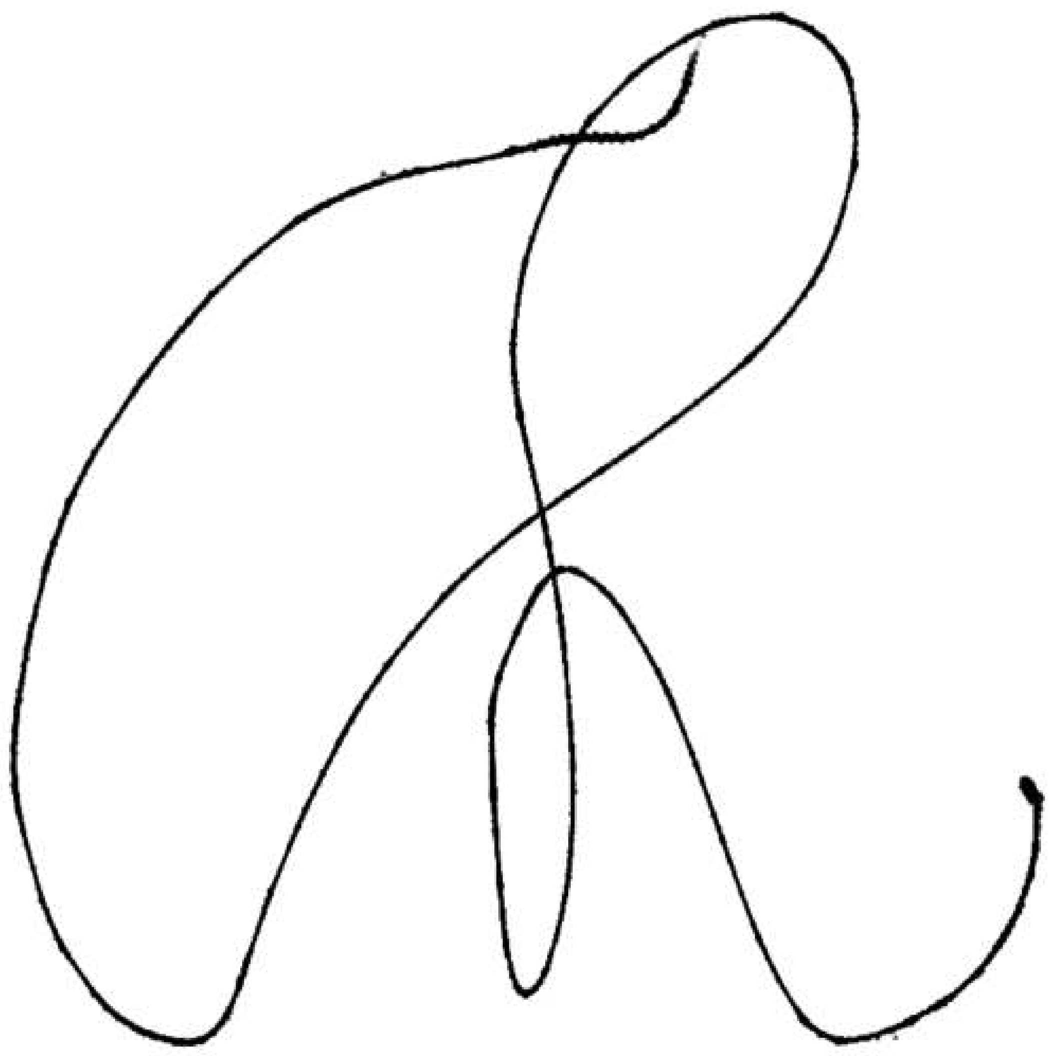

CL192
Low_complexity
Length of Reads (GP):5366 (0.07%)

Tgrandiflorum

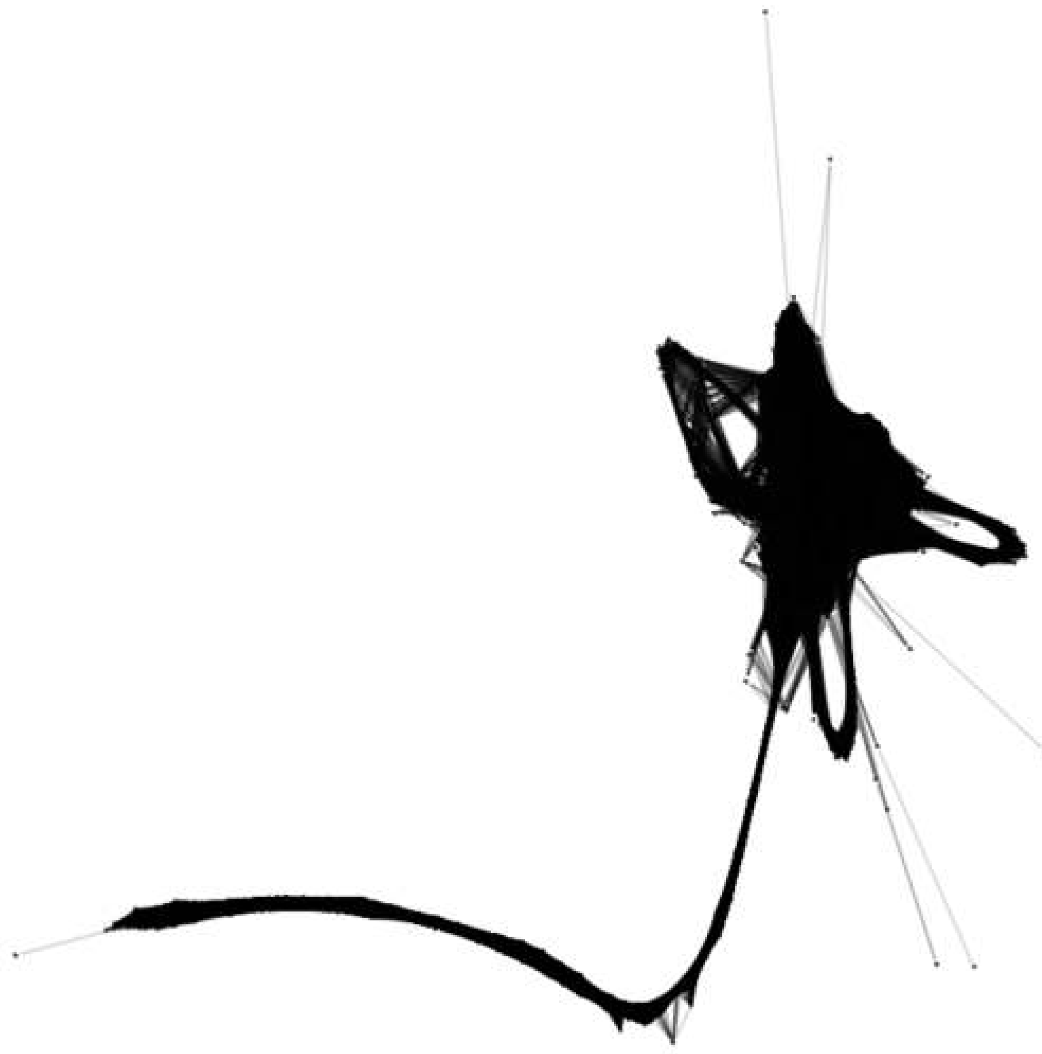

CL193
Simple_repeat
Length of Reads (GP):5363 (0.07%)

Tgrandiflorum

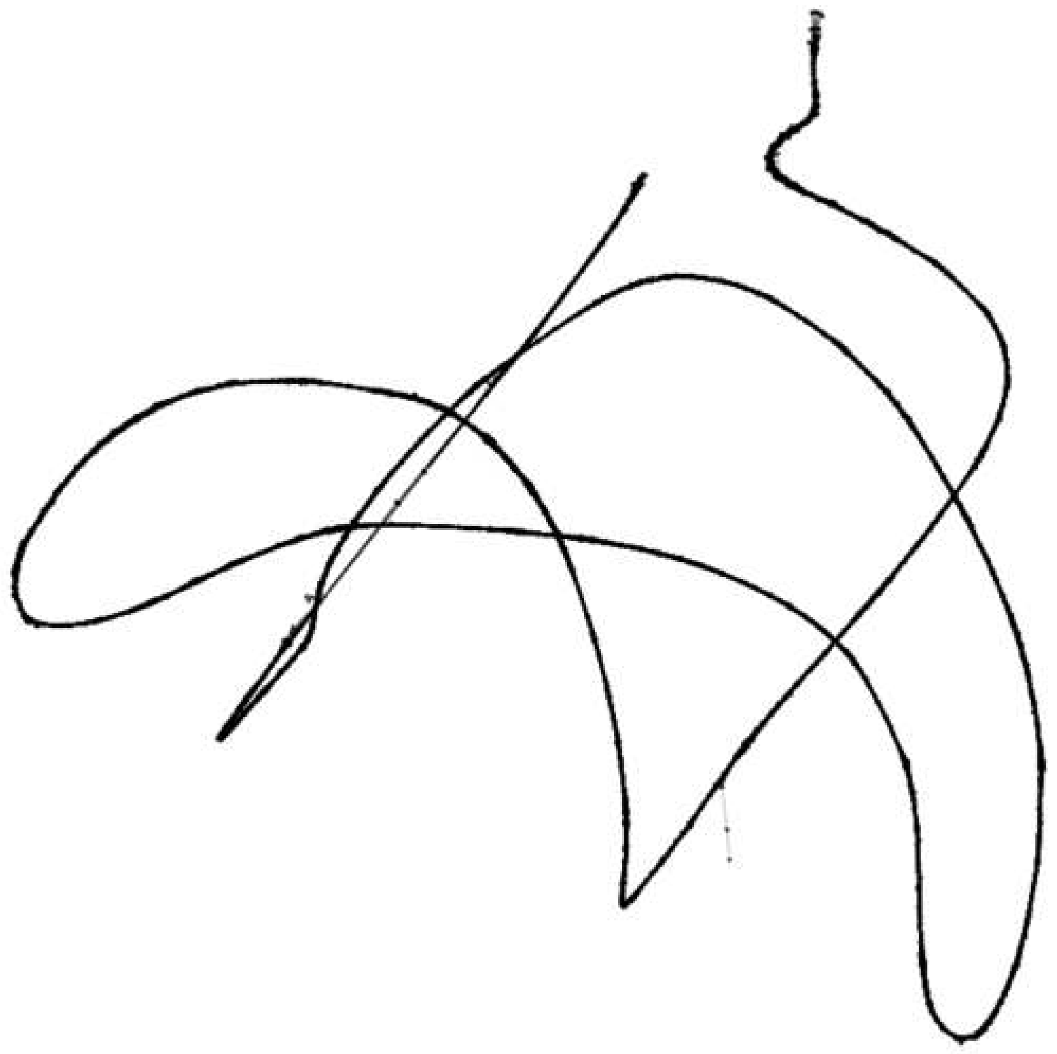

CL194
Low_complexity
Length of Reads (GP):5350 (0.07%)

Tgrandiflorum

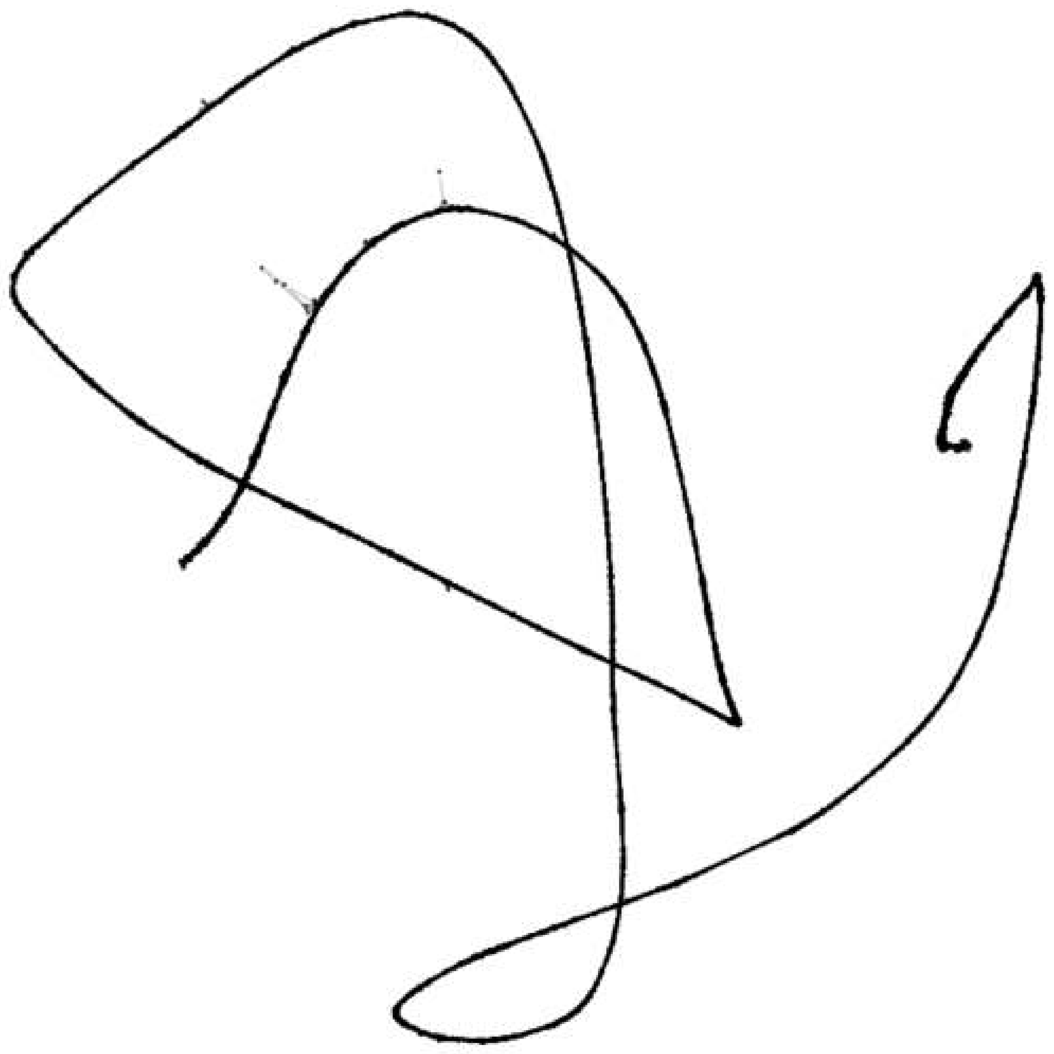

CL195
Low_complexity
Length of Reads (GP):5228 (0.07%)

Tgrandiflorum

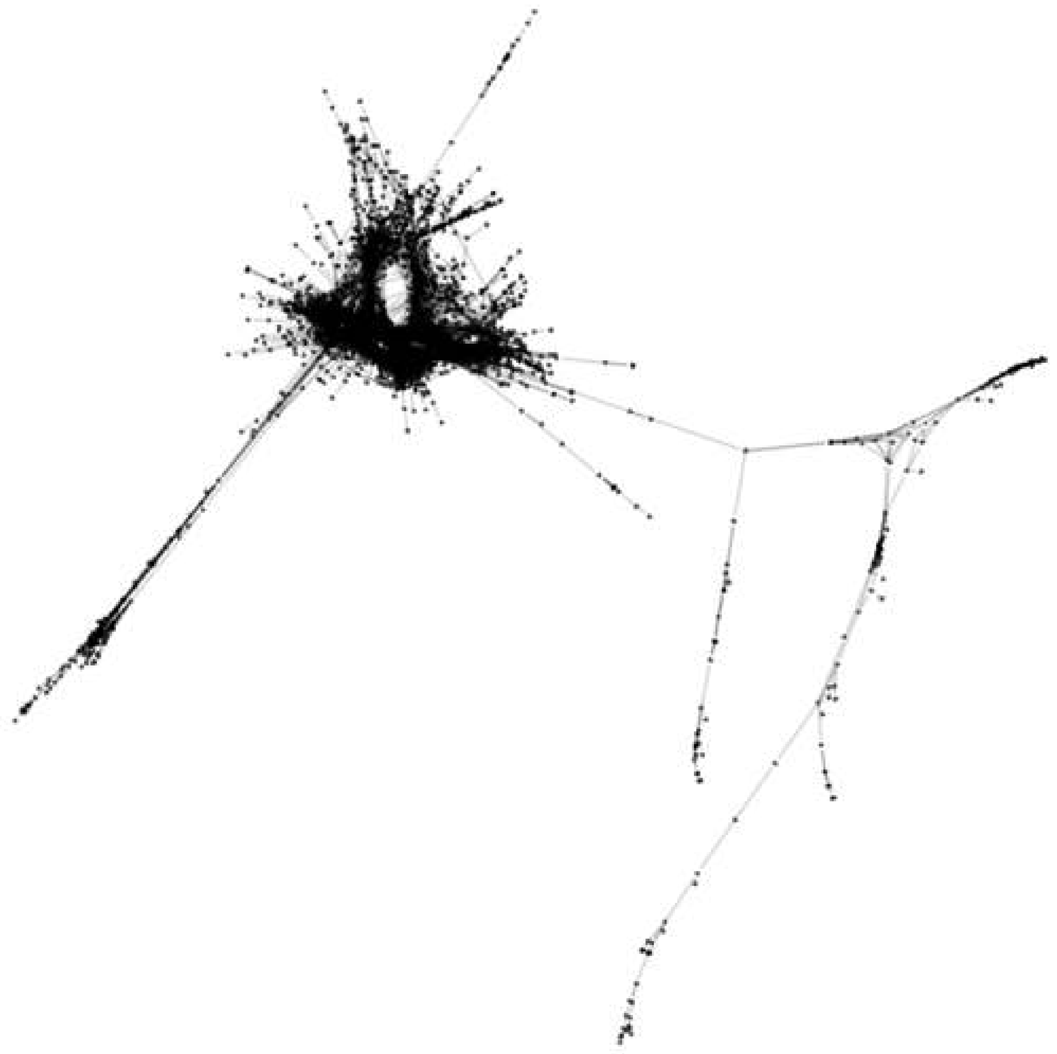

CL196
Low_complexity
Length of Reads (GP):5173 (0.07%)

Tgrandiflorum

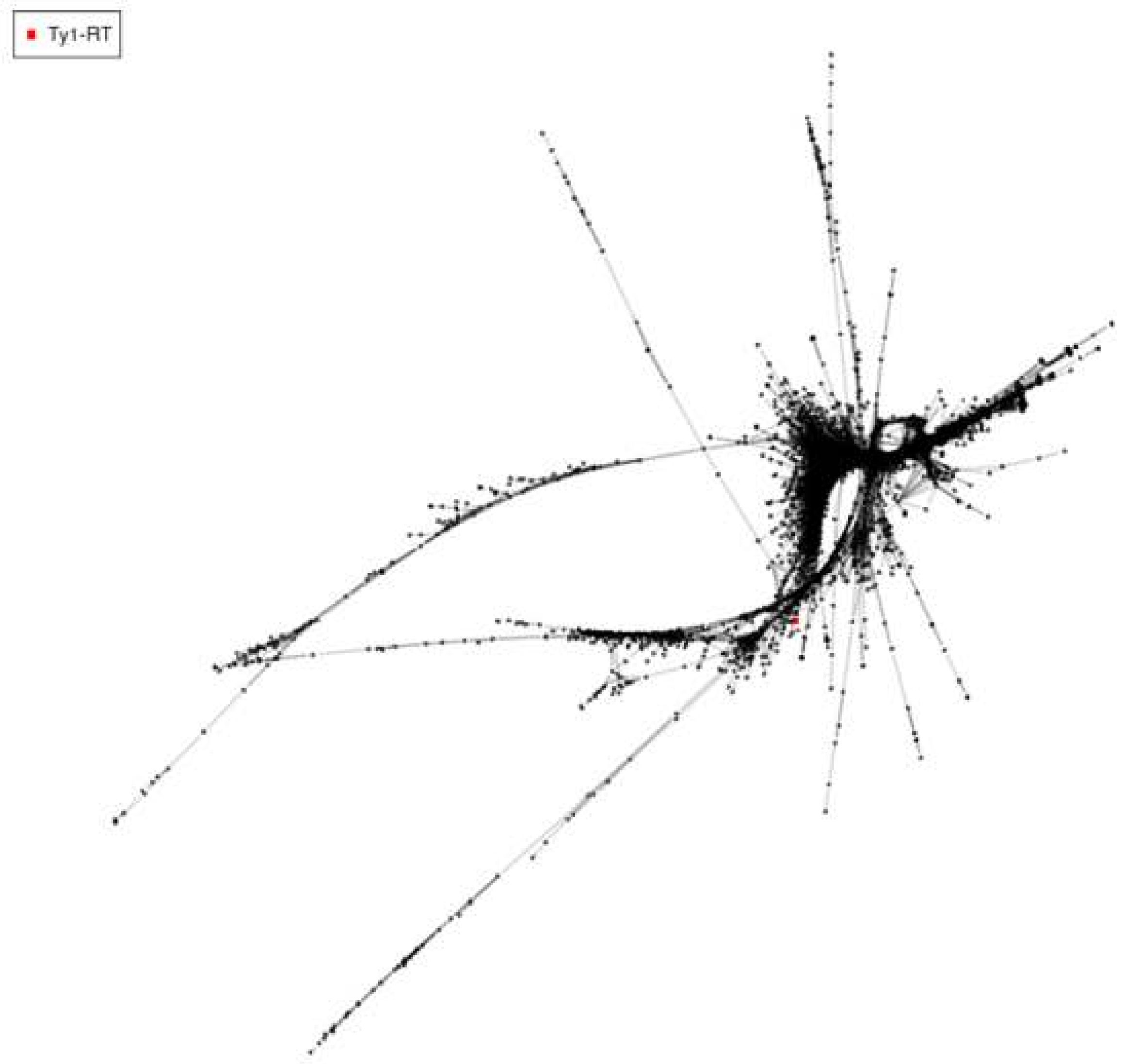

CL197
Low_complexity
Length of Reads (GP):5144 (0.06%)

Tgrandiflorum

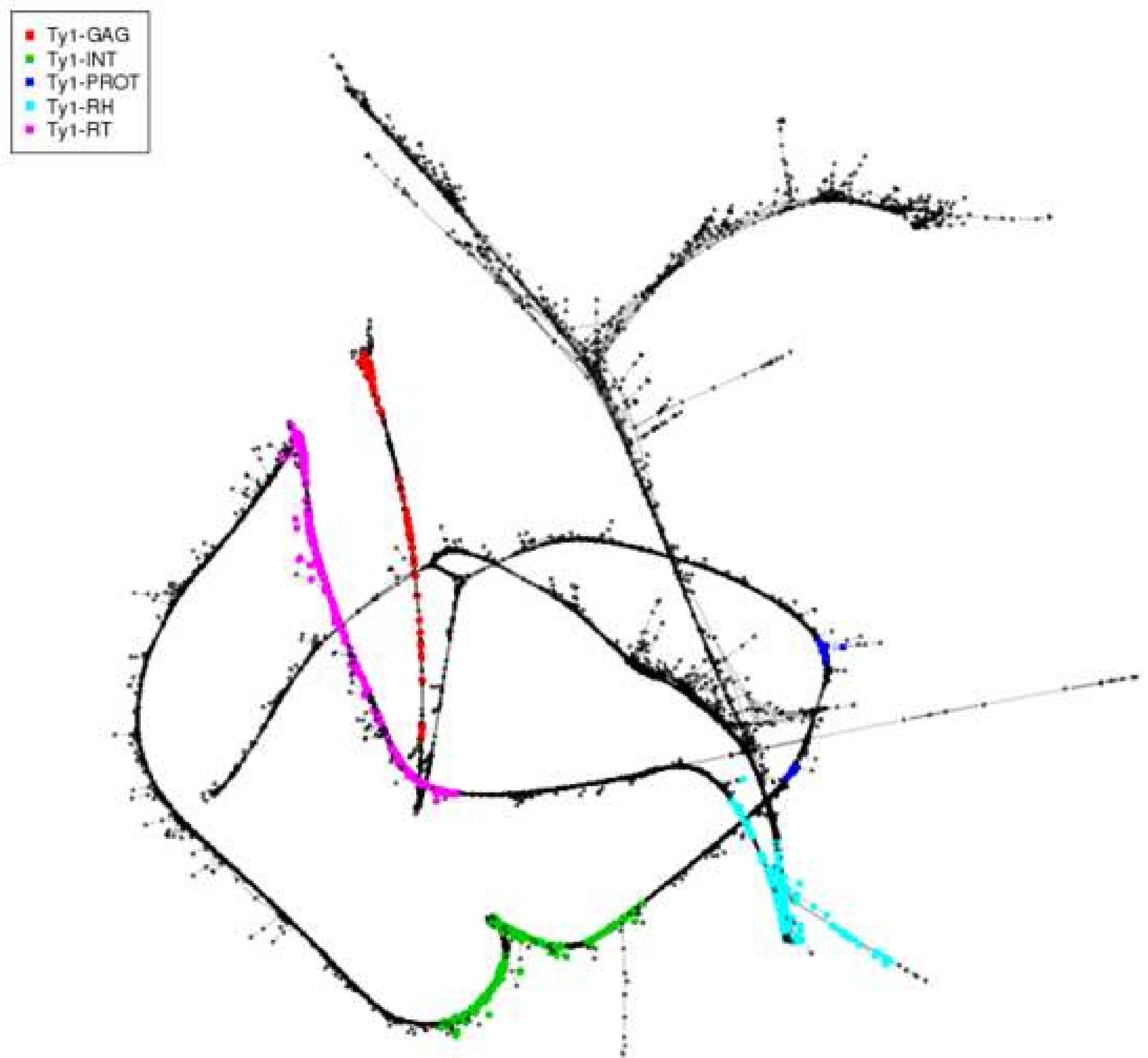

CL198
LTR_Copia
Length of Reads (GP):5122 (0.06%)

Tgrandiflorum

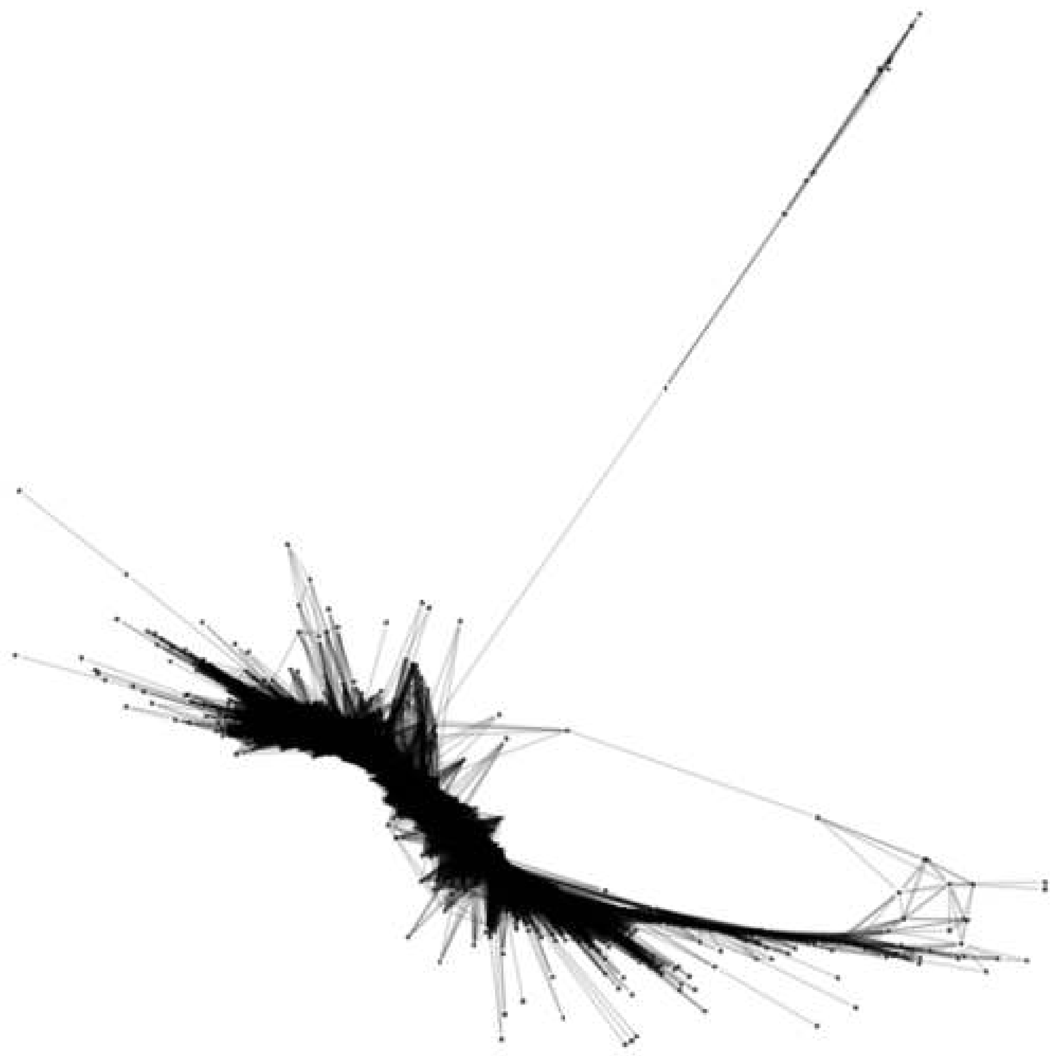

CL199
LTR_Gypsy
Length of Reads (GP):5117 (0.06%)

Tgrandiflorum

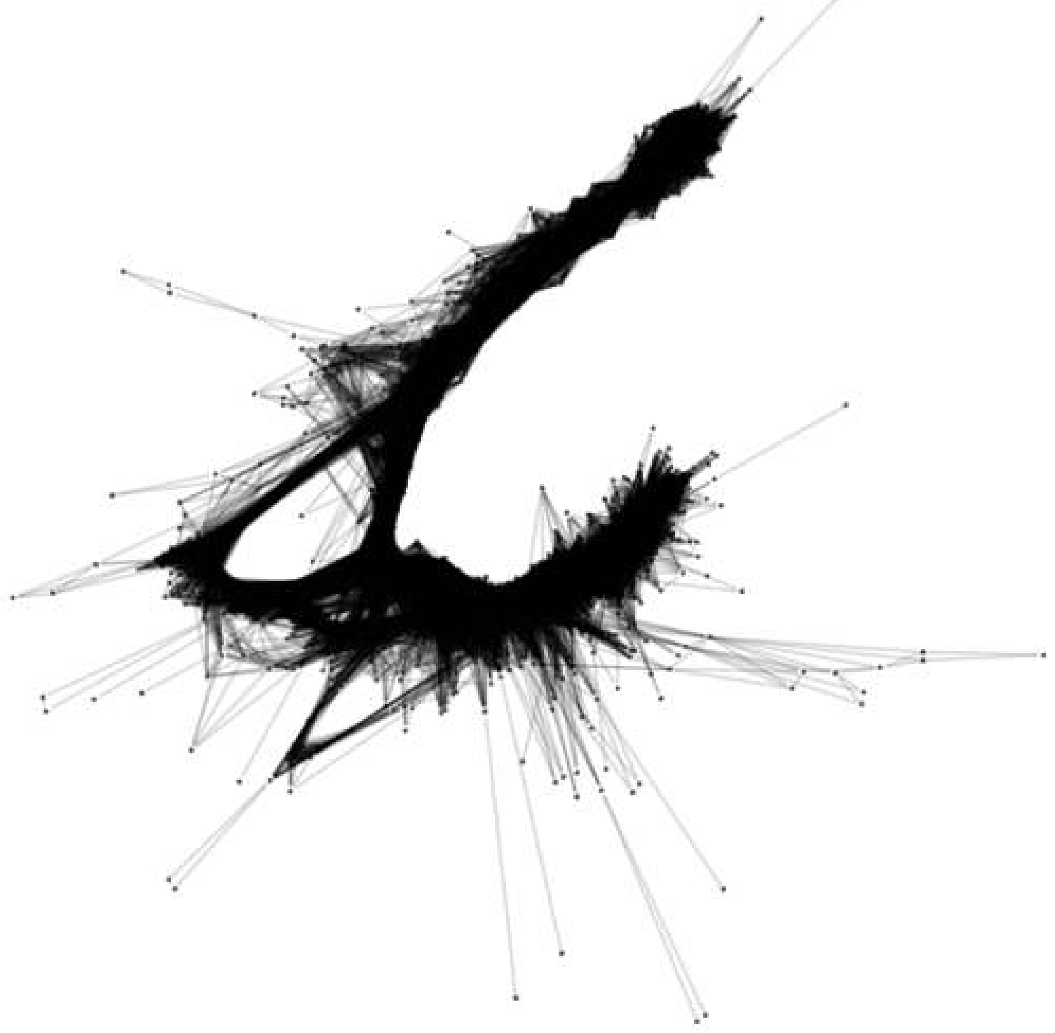

CL200
Low_complexity
Length of Reads (GP):4922 (0.06%)

Tgrandiflorum

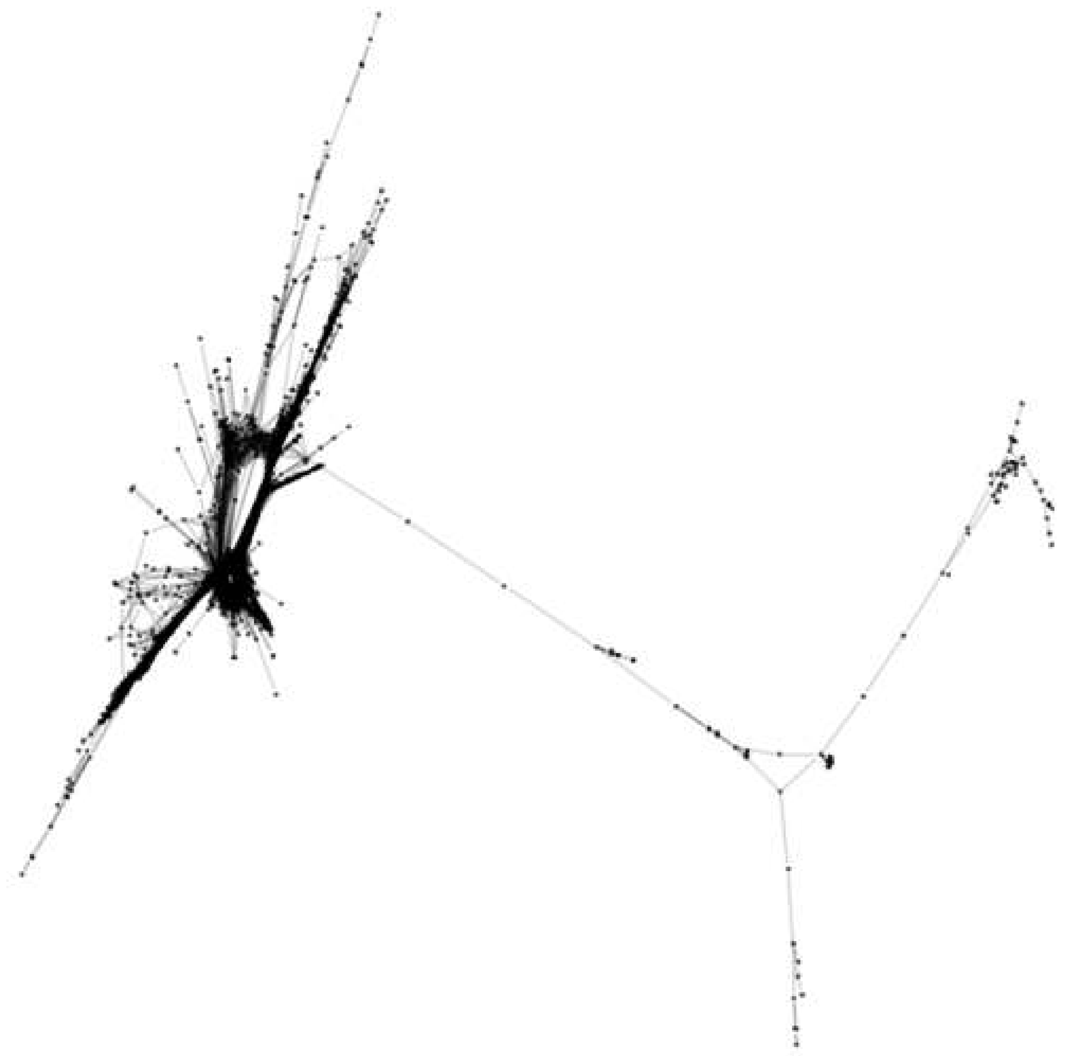

CL201
Low_complexity
Length of Reads (GP):4865 (0.06%)

Tgrandiflorum

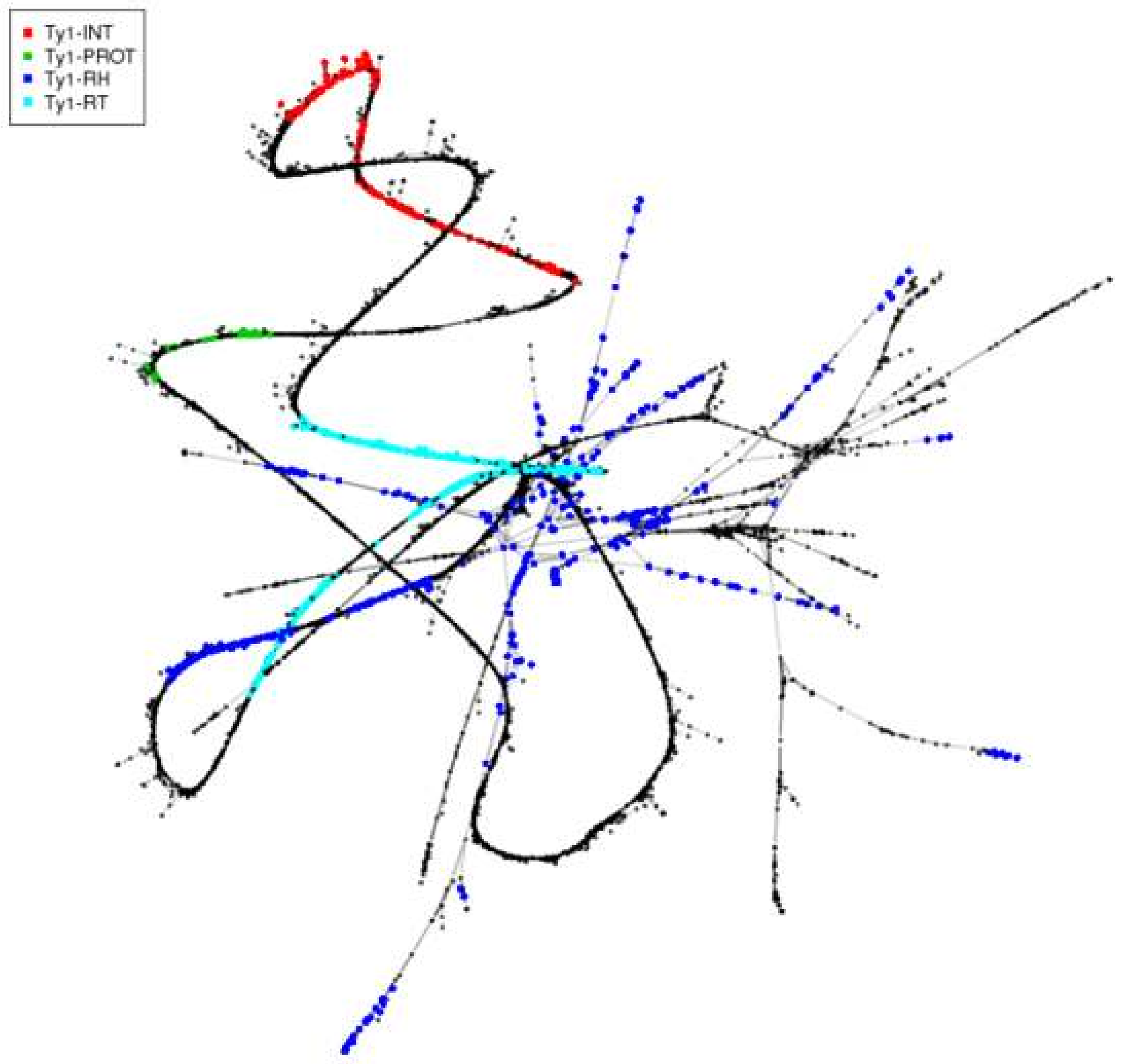

CL202
LTR_Copia
Length of Reads (GP):4822 (0.06%)

Tgrandiflorum

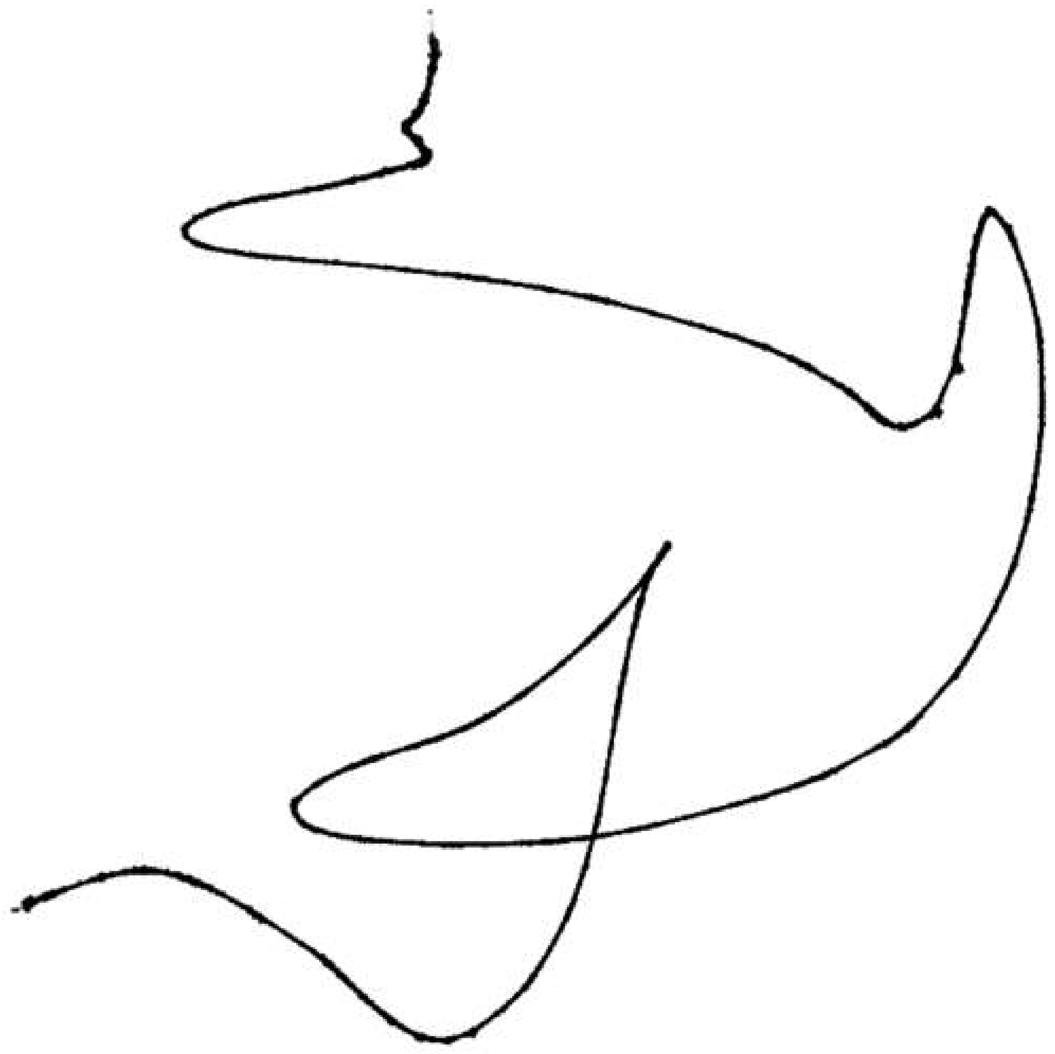

CL203
Low_complexity
Length of Reads (GP):4761 (0.06%)

Tgrandiflorum

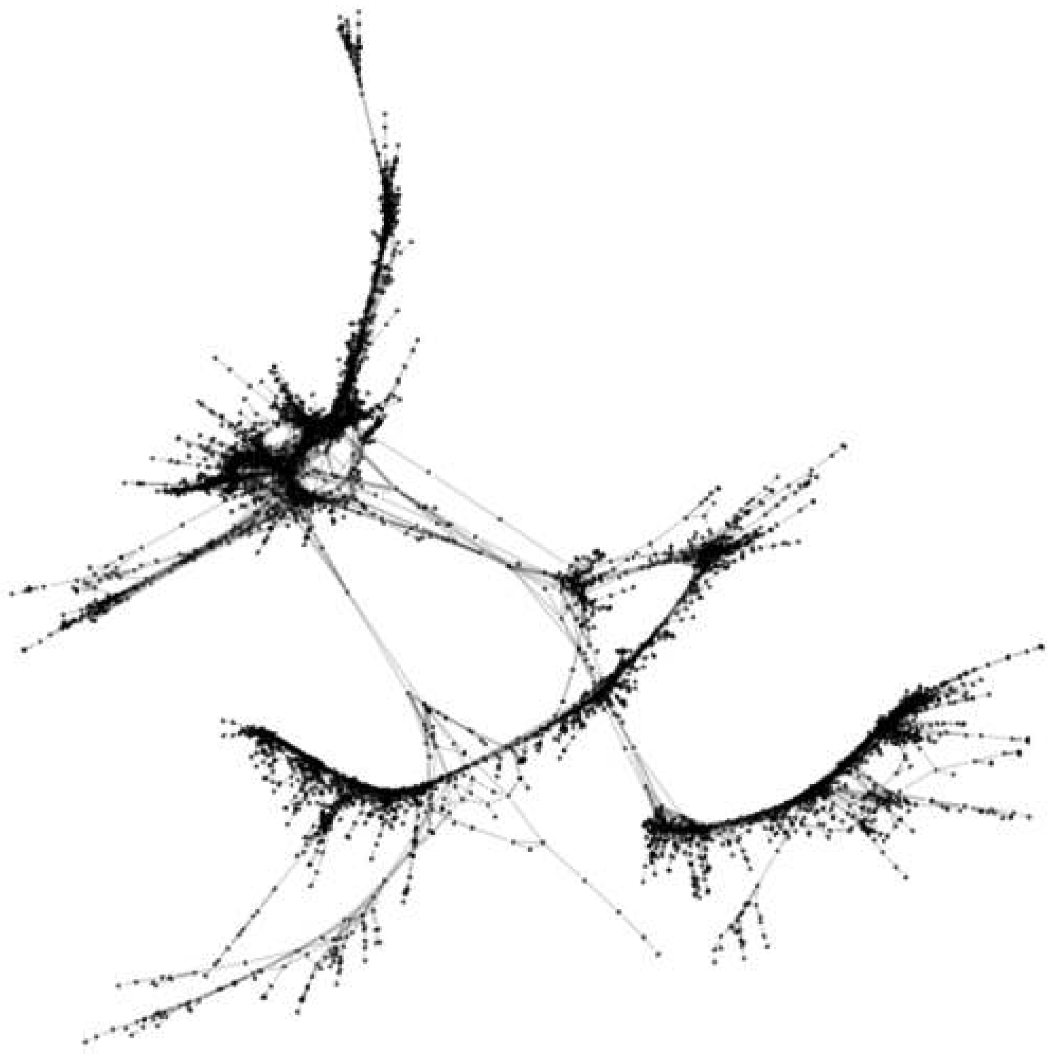

CL204
LTR_Copia
Length of Reads (GP):4572 (0.06%)

Tgrandiflorum

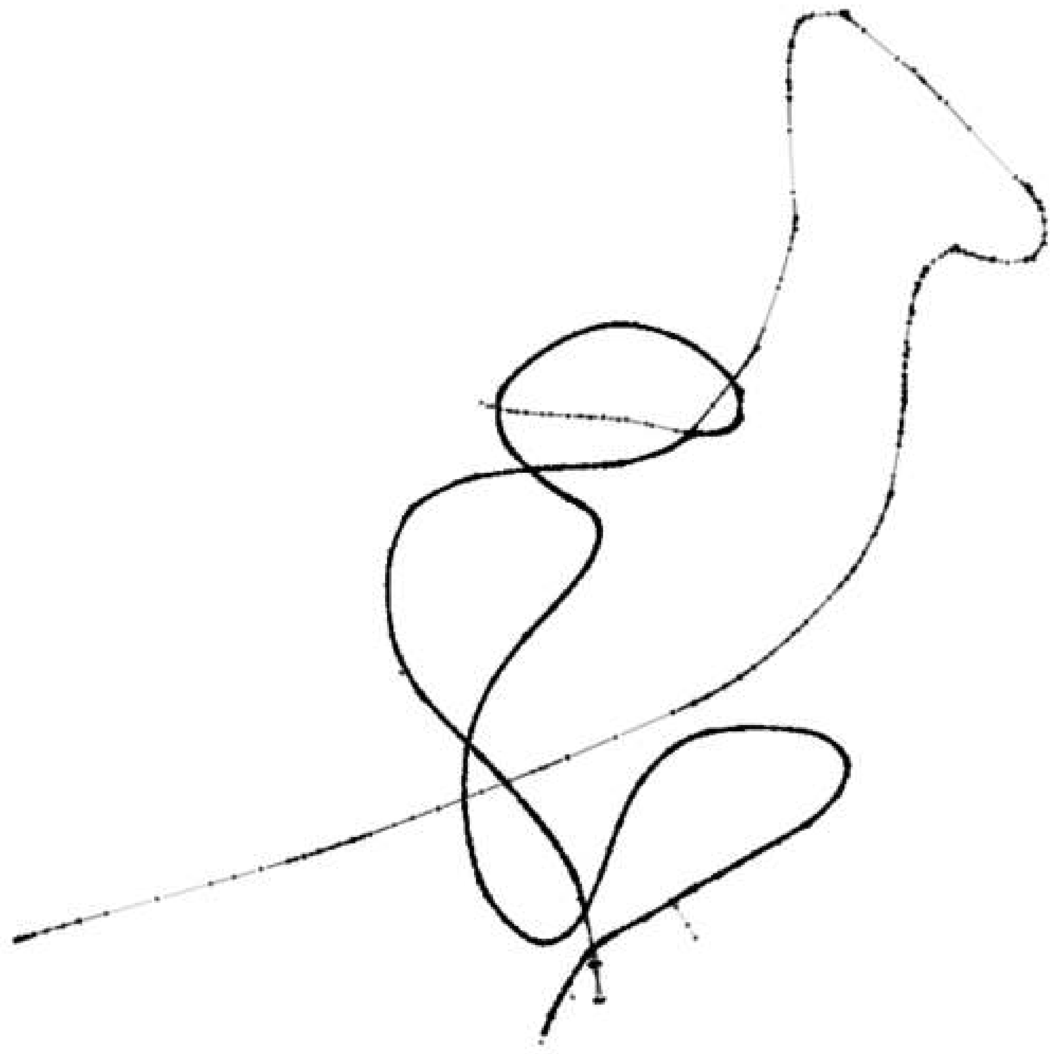

CL205
Low_complexity
Length of Reads (GP):4512 (0.06%)

Tgrandiflorum

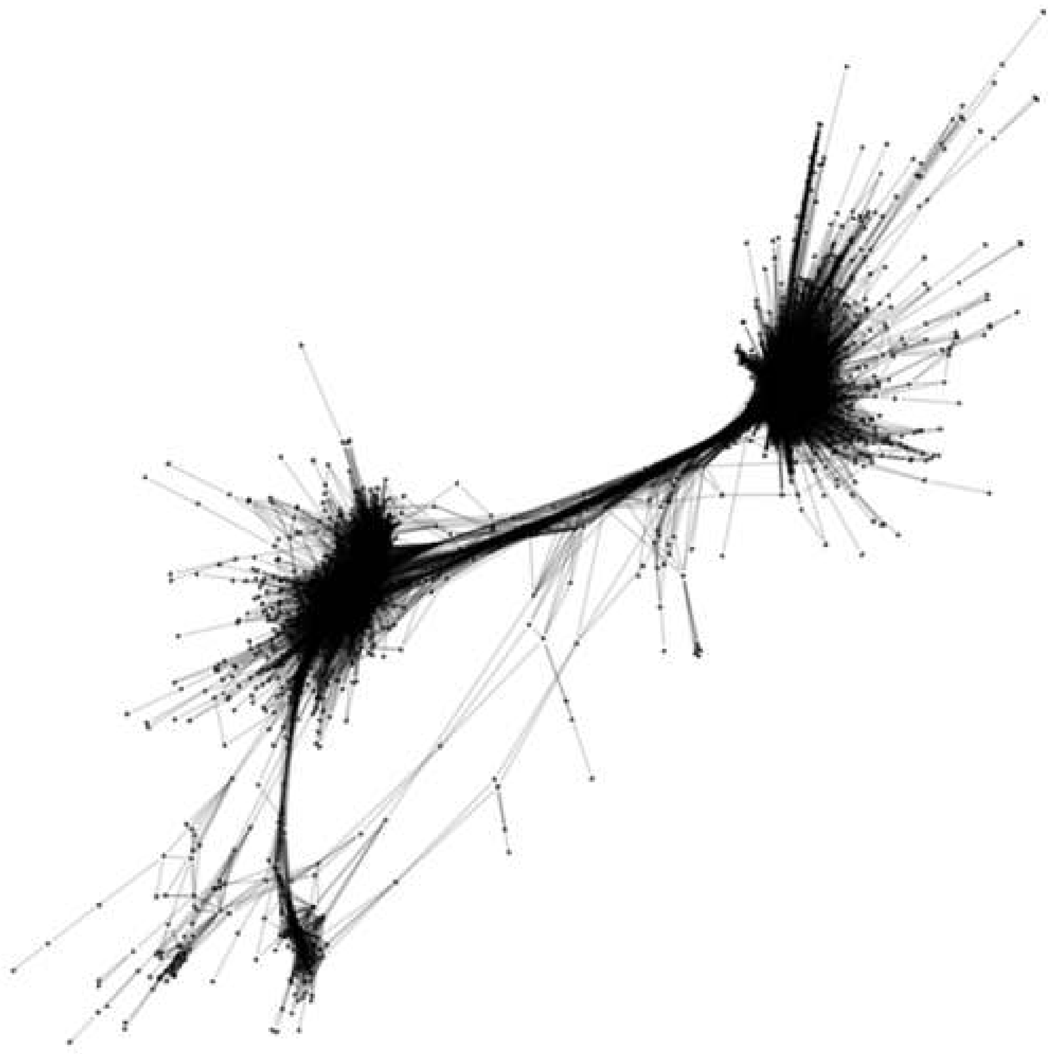

CL206
Low_complexity
Length of Reads (GP):4493 (0.06%)

Tgrandiflorum

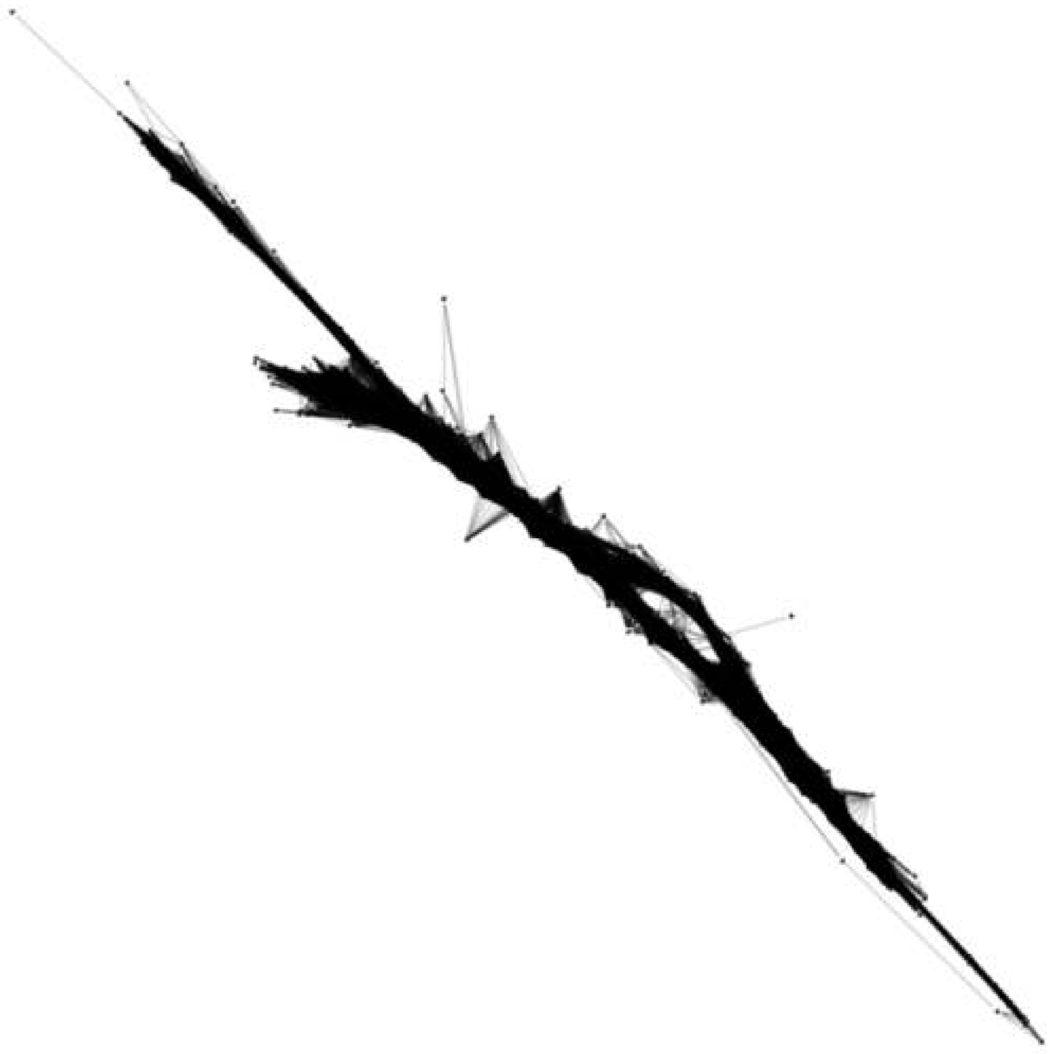

CL207
Low_complexity
Length of Reads (GP):4400 (0.06%)

Tgrandiflorum

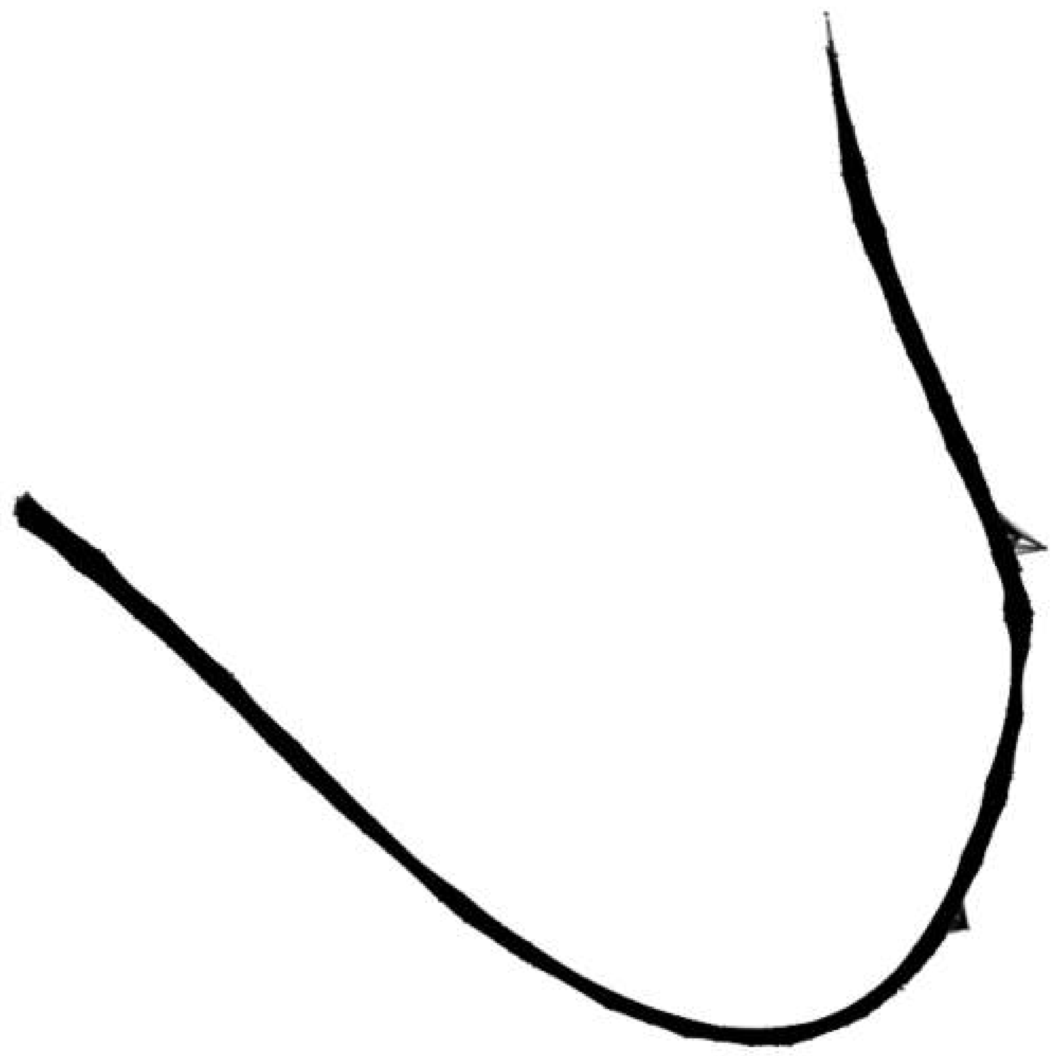

CL208
rRNA
Length of Reads (GP):4265 (0.05%)

Tgrandiflorum

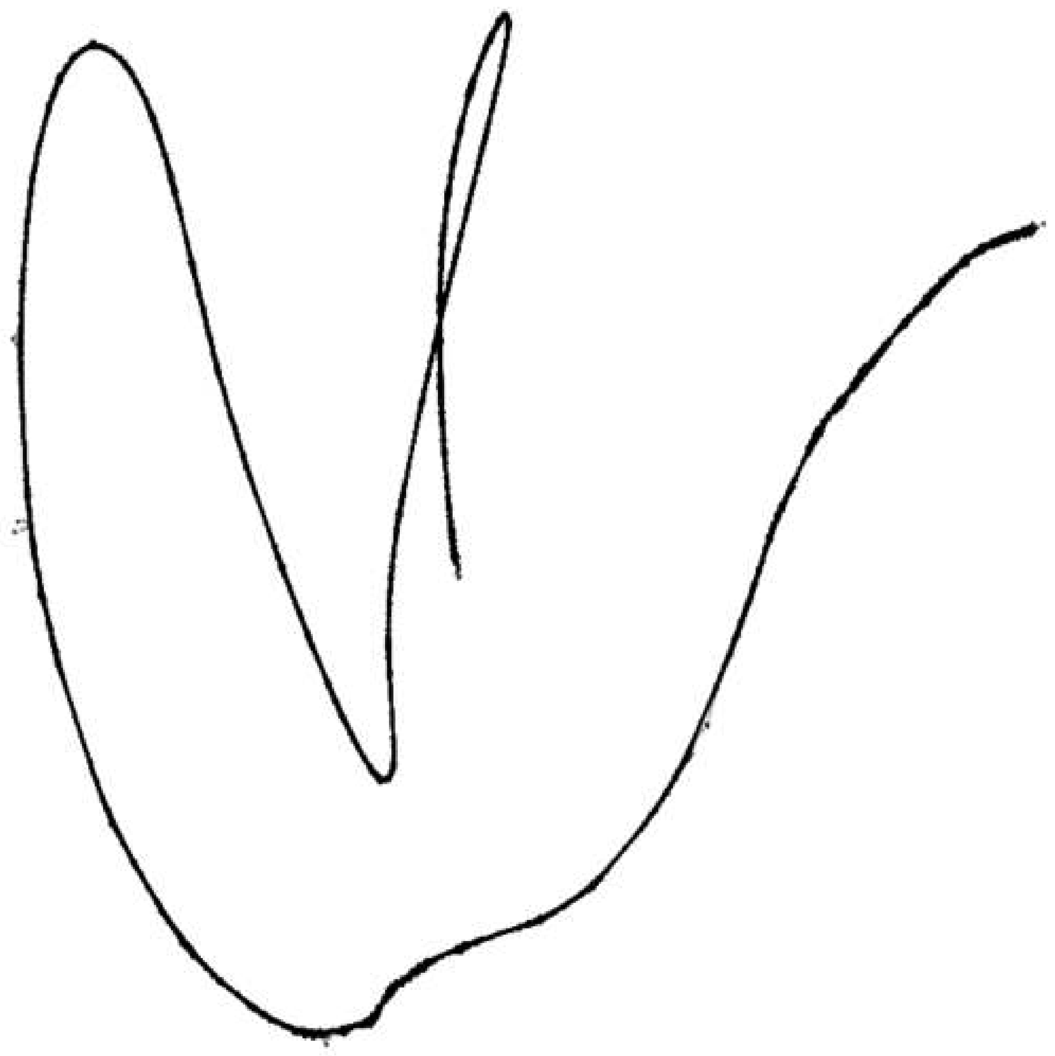

CL209
Low_complexity
Length of Reads (GP):4229 (0.05%)

Tgrandiflorum

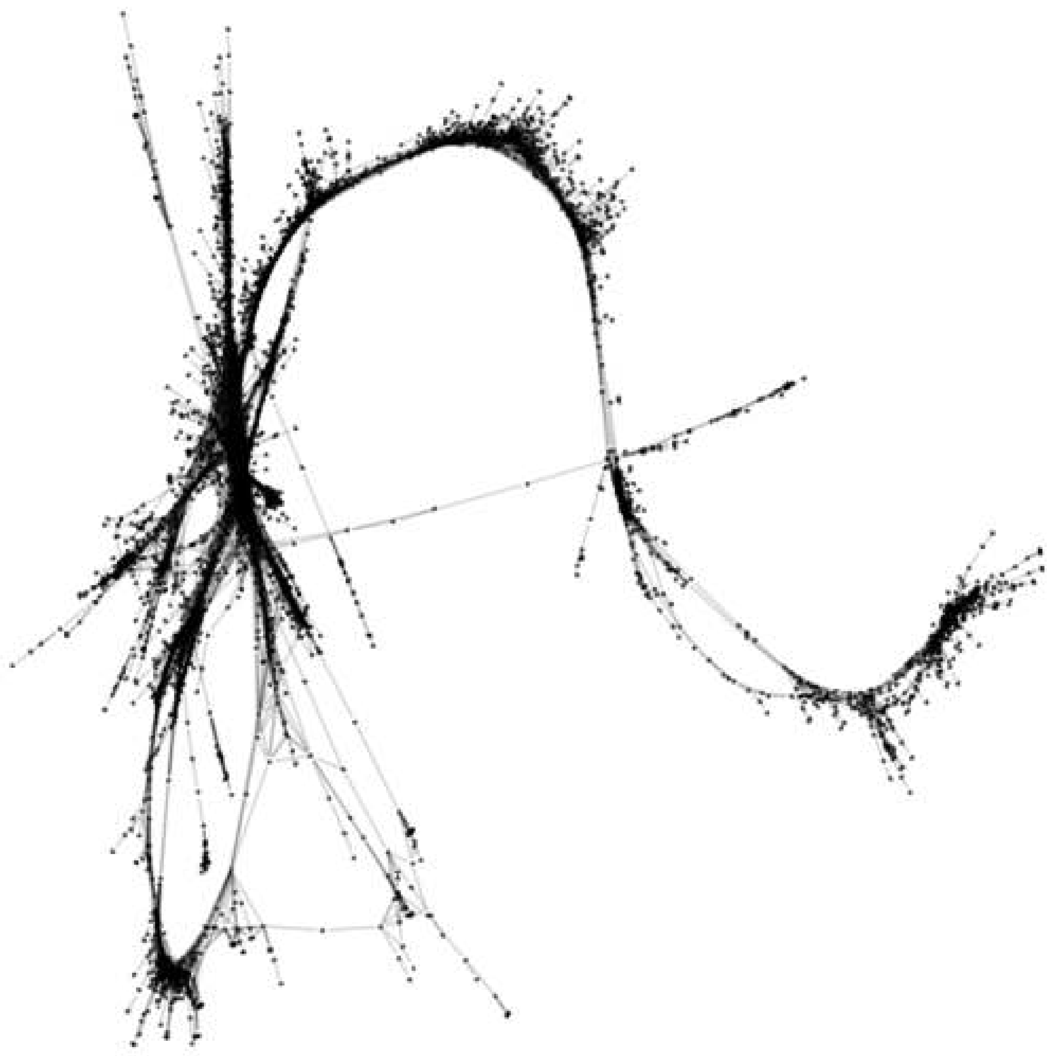

CL210
Low_complexity
Length of Reads (GP):4217 (0.05%)

Tgrandiflorum

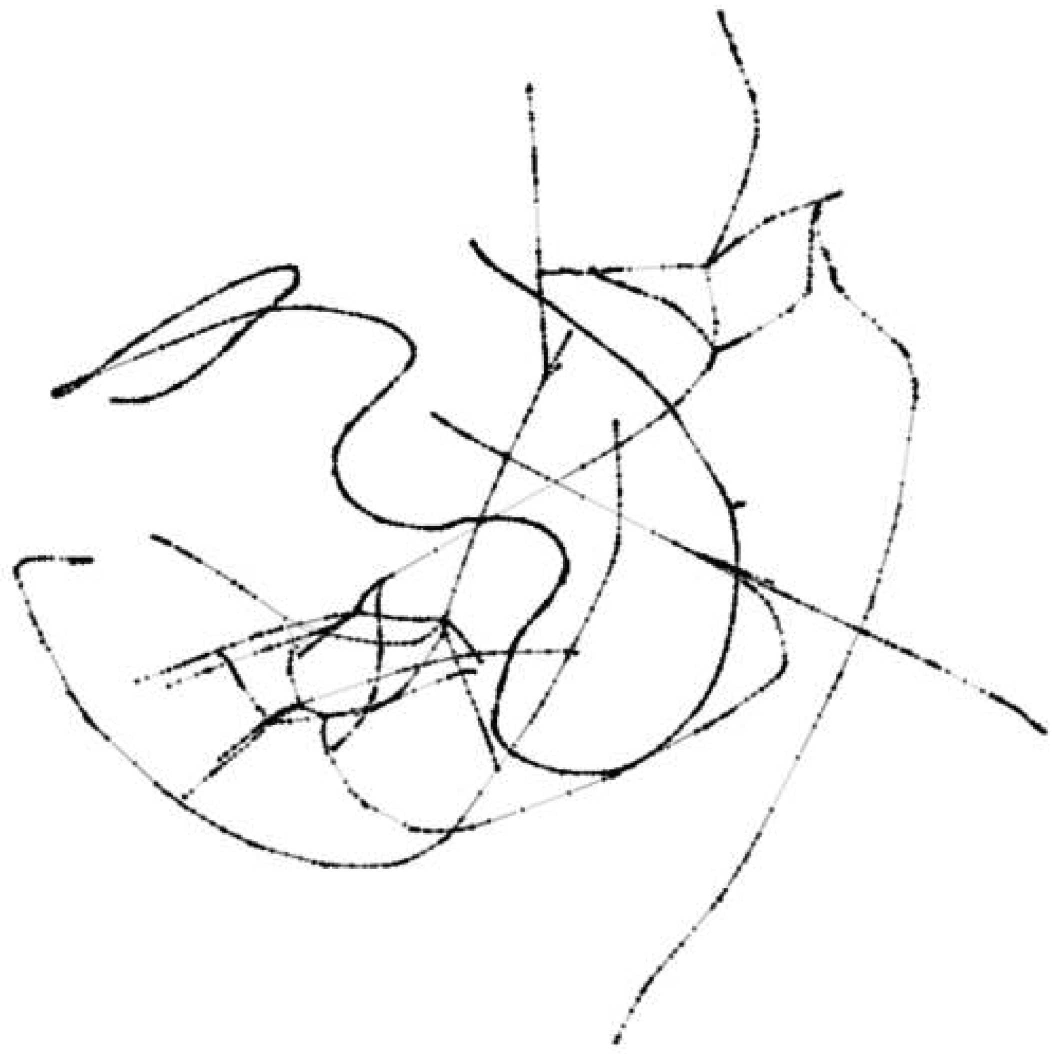

CL211
LTR_Copia
Length of Reads (GP):4204 (0.05%)

Tgrandiflorum

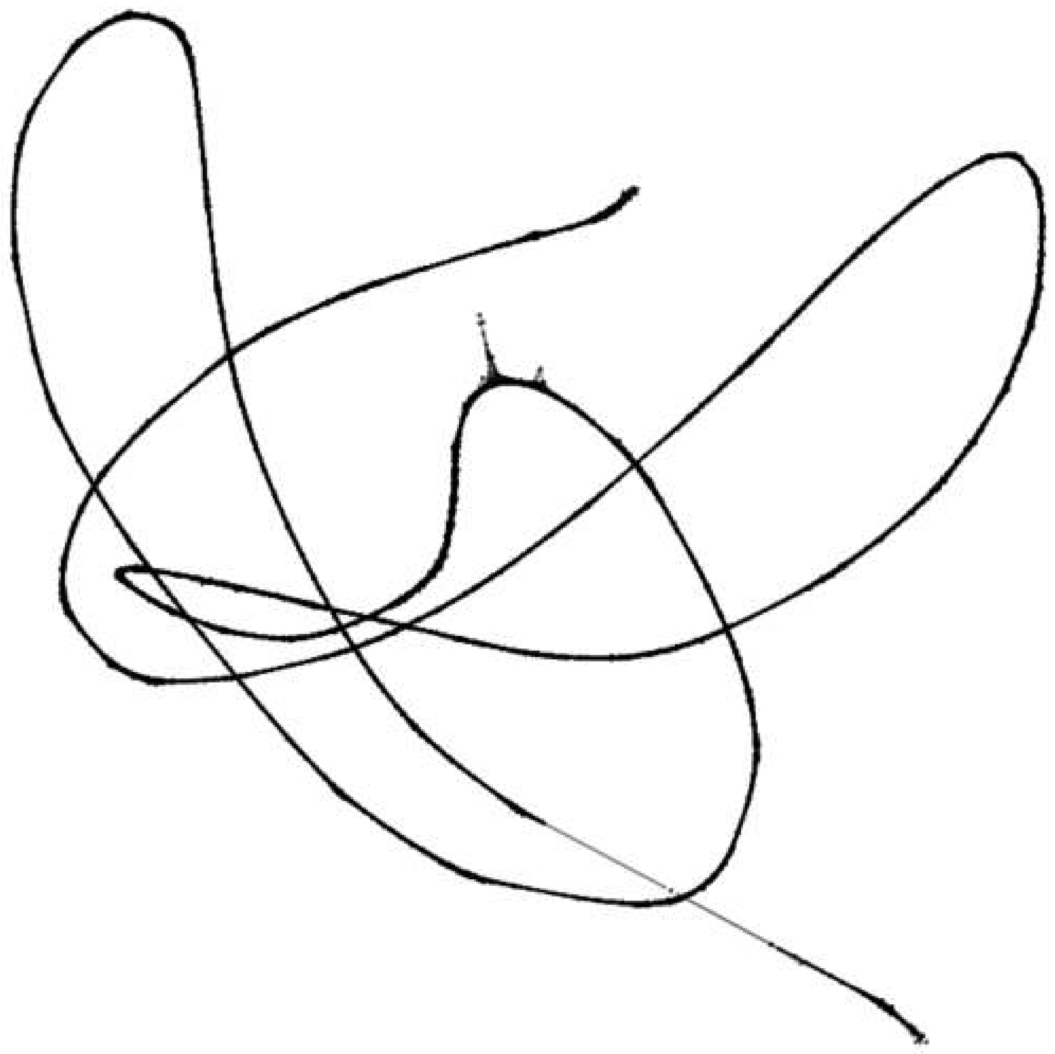

CL212
Low_complexity
Length of Reads (GP):4194 (0.05%)

Tgrandiflorum

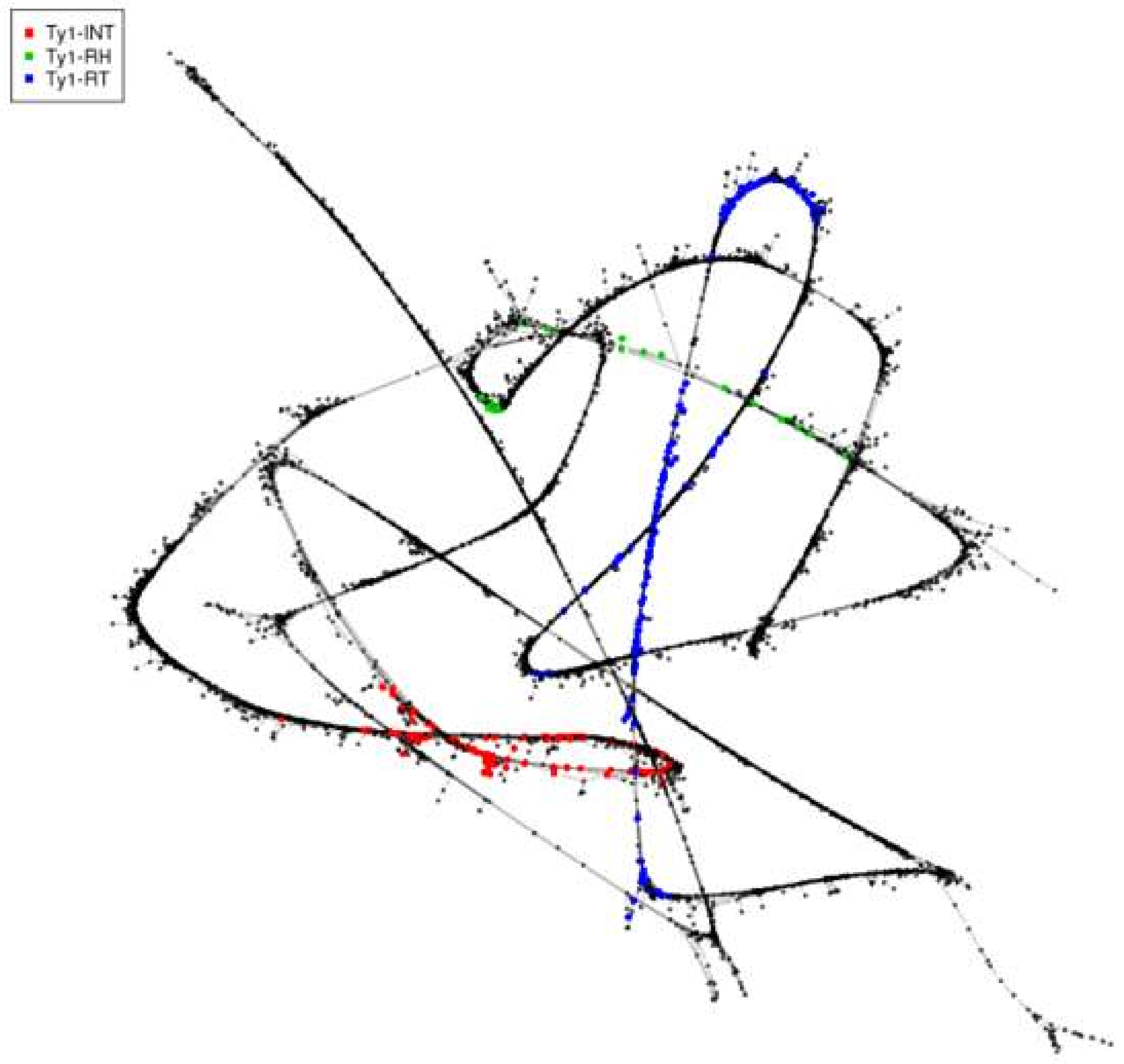

CL213
LTR_Copia
Length of Reads (GP):4038 (0.05%)

Tgrandiflorum

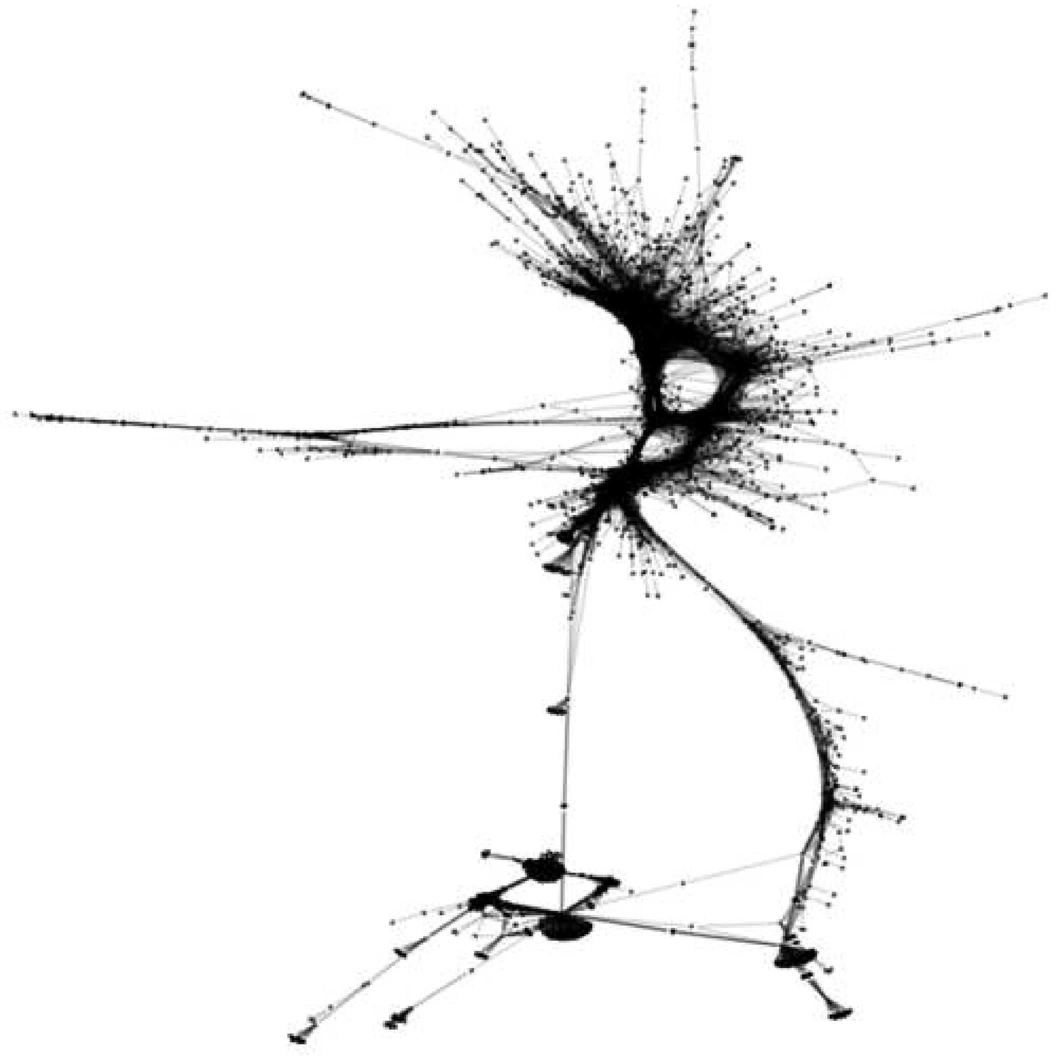

CL214
Low_complexity
Length of Reads (GP):3999 (0.05%)

Tgrandiflorum

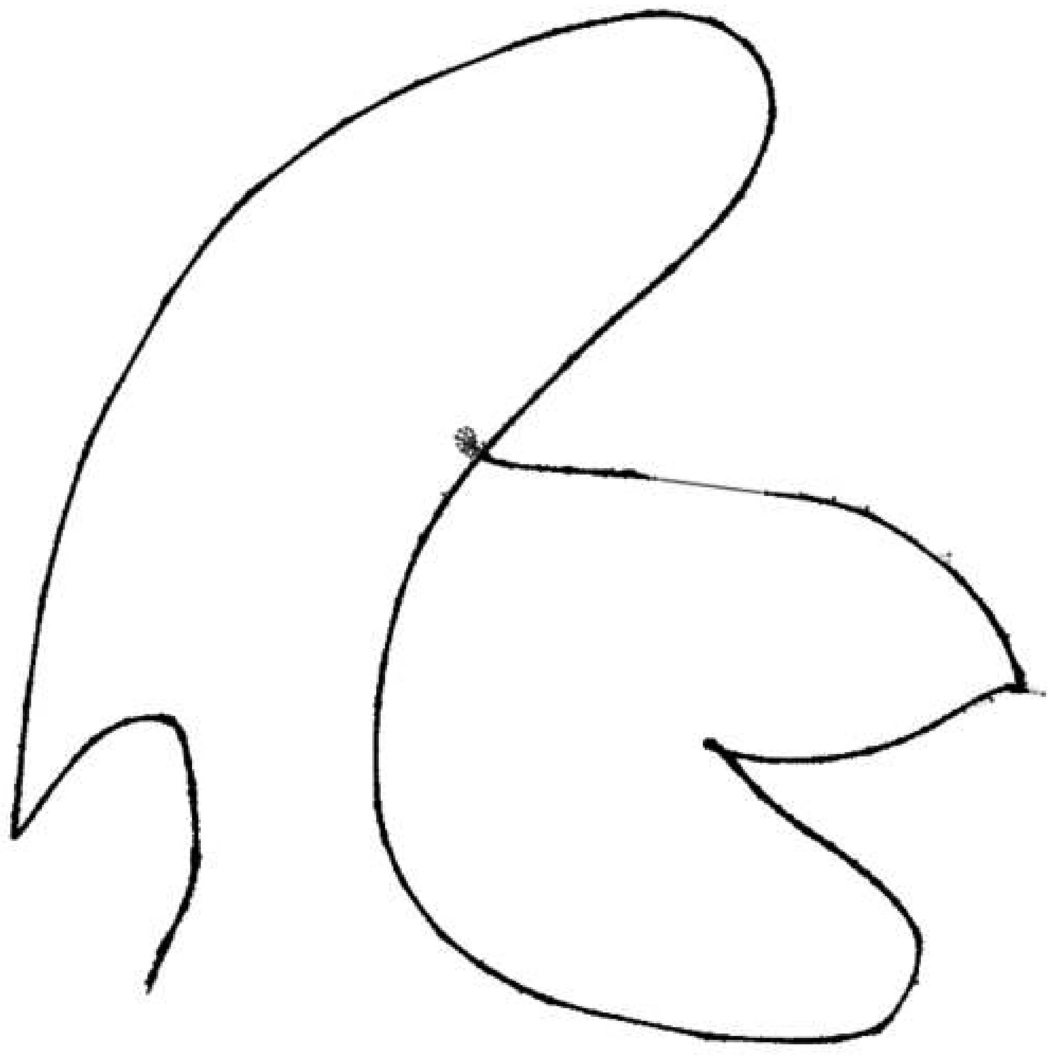

CL215
Low_complexity
Length of Reads (GP):3993 (0.05%)

Tgrandiflorum

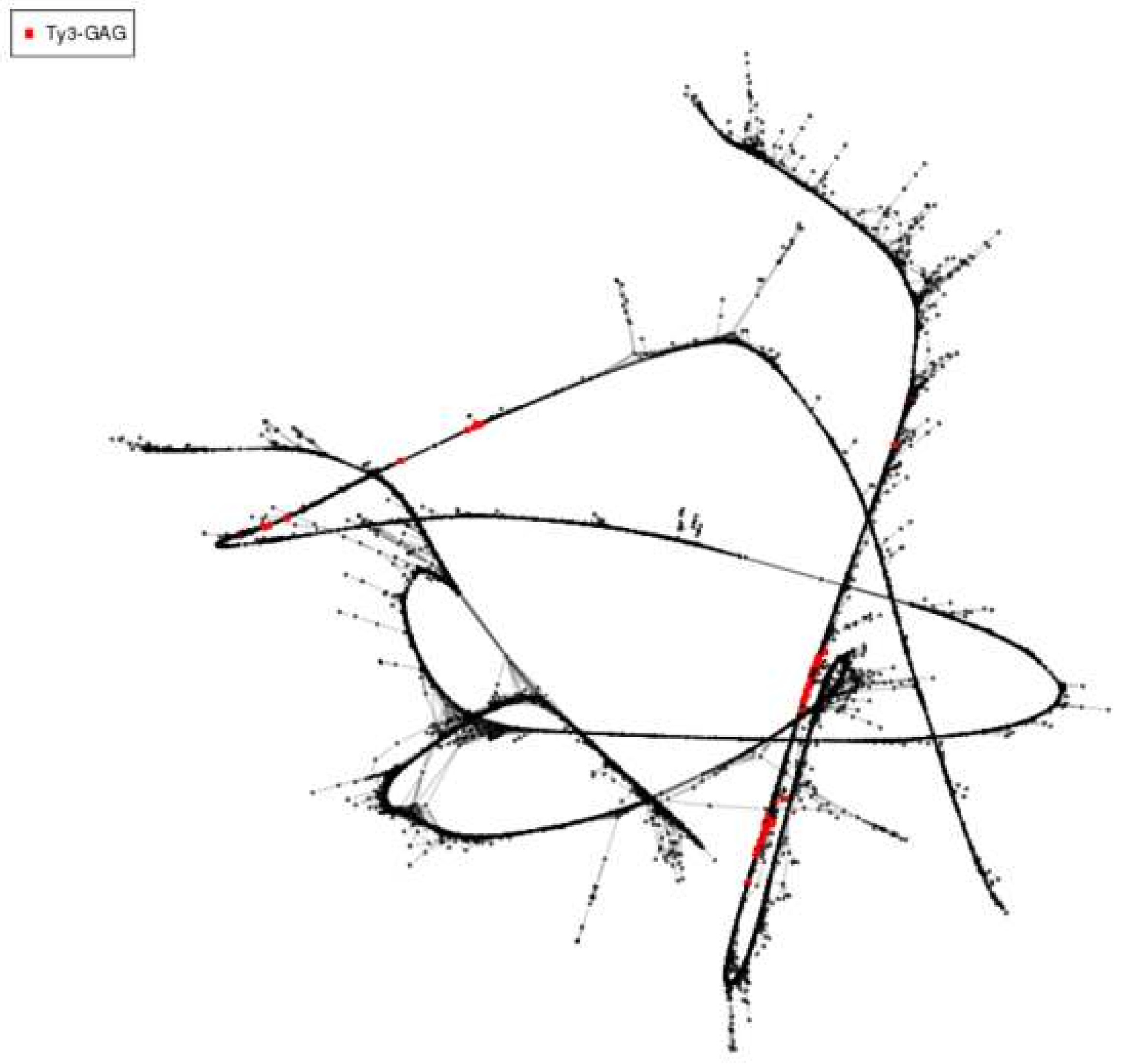

CL216
LTR_Gypsy
Length of Reads (GP):3973 (0.05%)

Tgrandiflorum

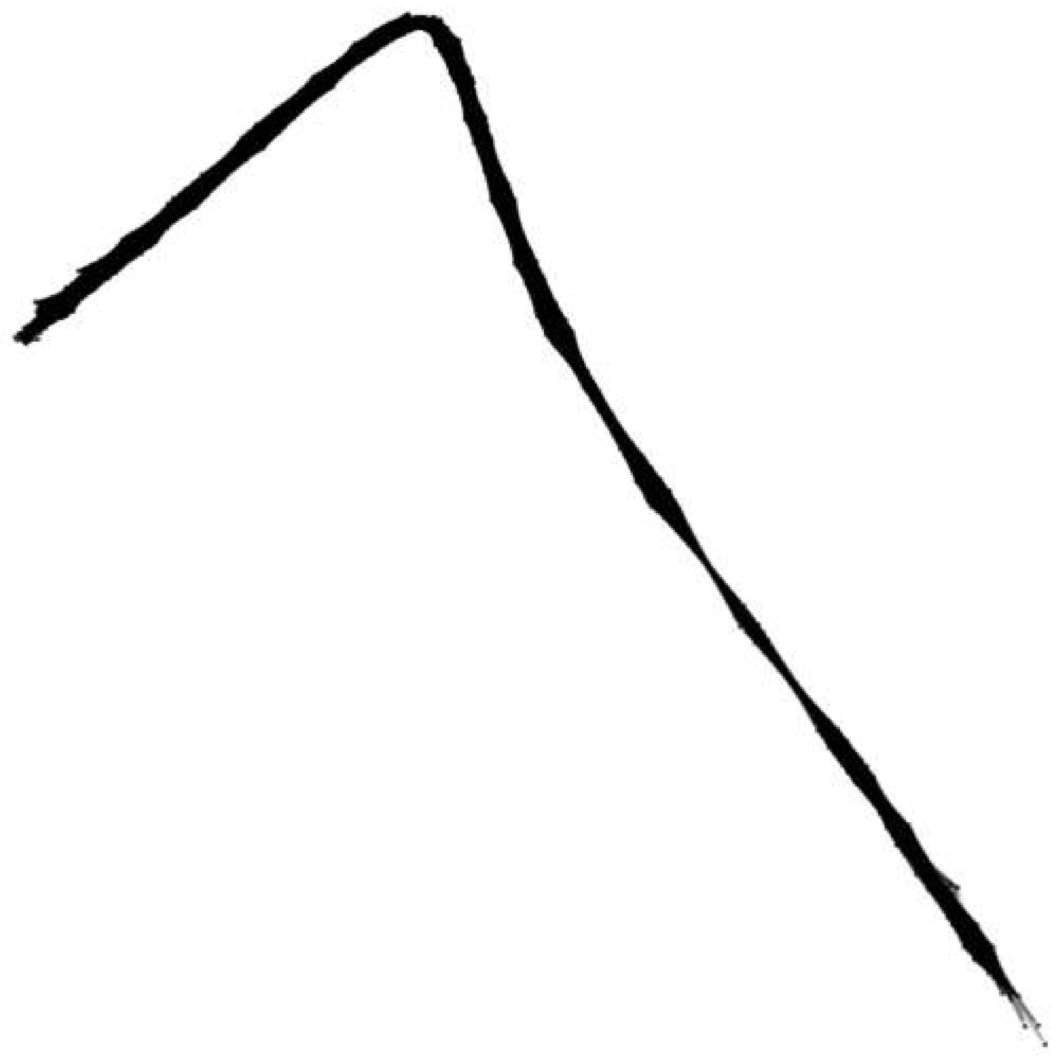

CL217
rRNA
Length of Reads (GP):3955 (0.05%)

Tgrandiflorum

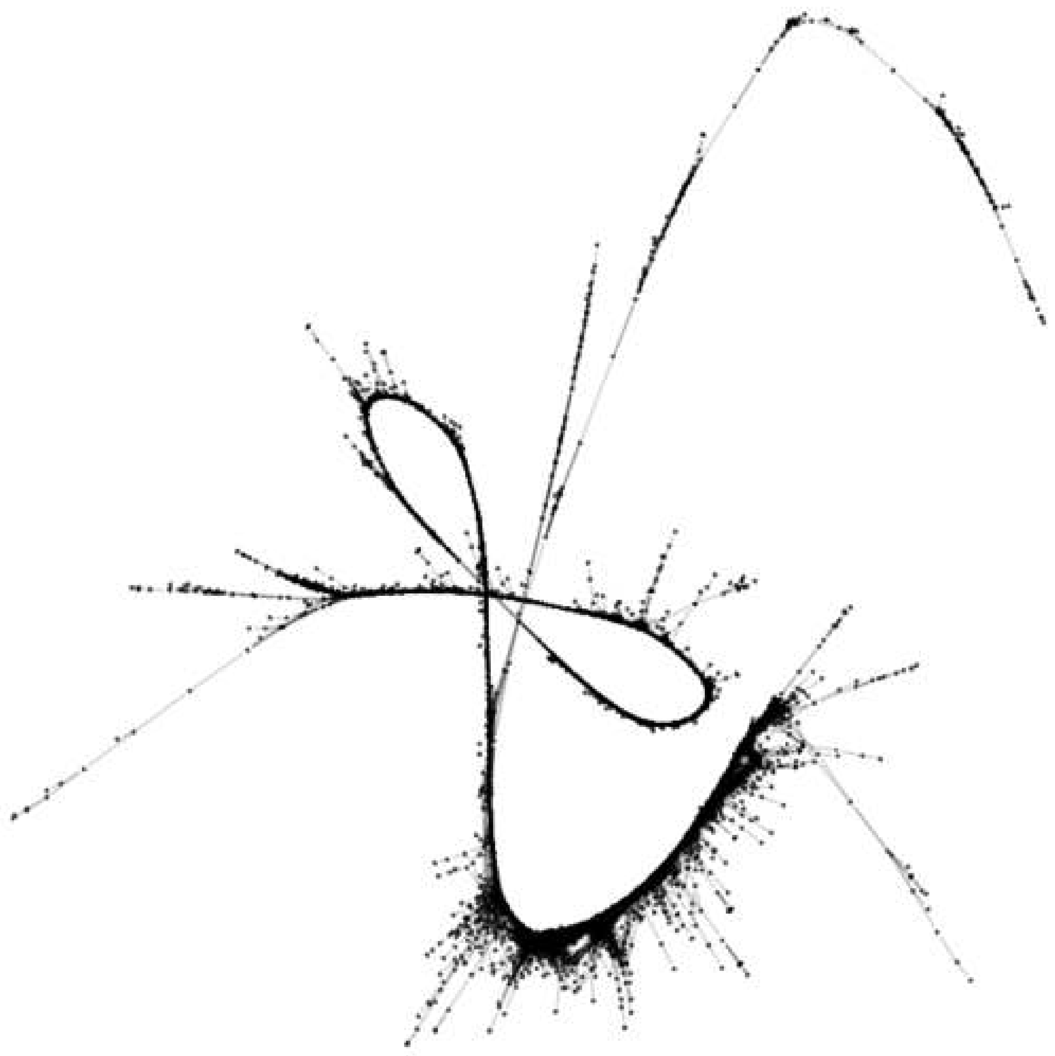

CL218
Low_complexity
Length of Reads (GP):3904 (0.05%)

Tgrandiflorum

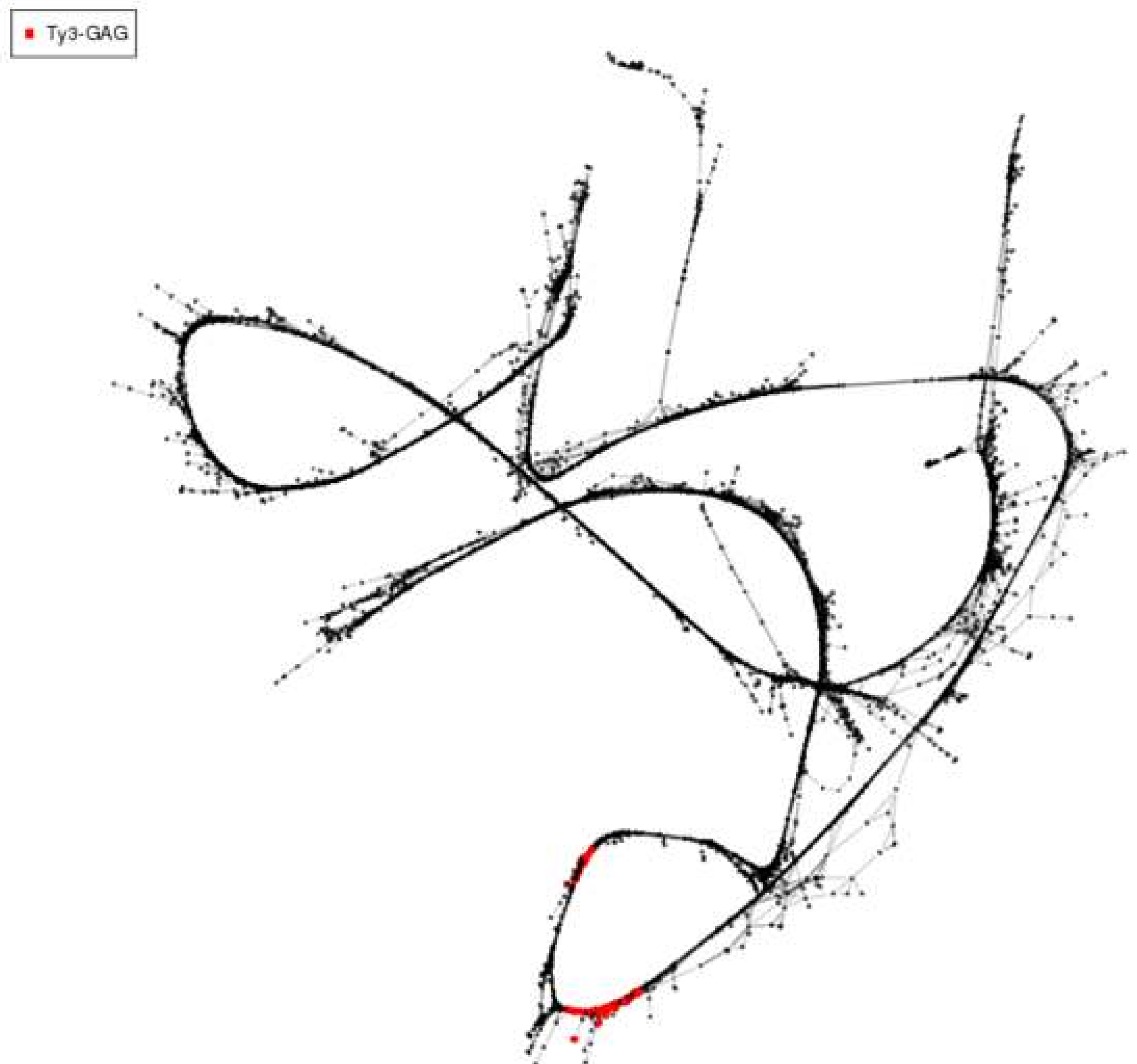

CL219
LTR_Gypsy
Length of Reads (GP):3882 (0.05%)

Tgrandiflorum

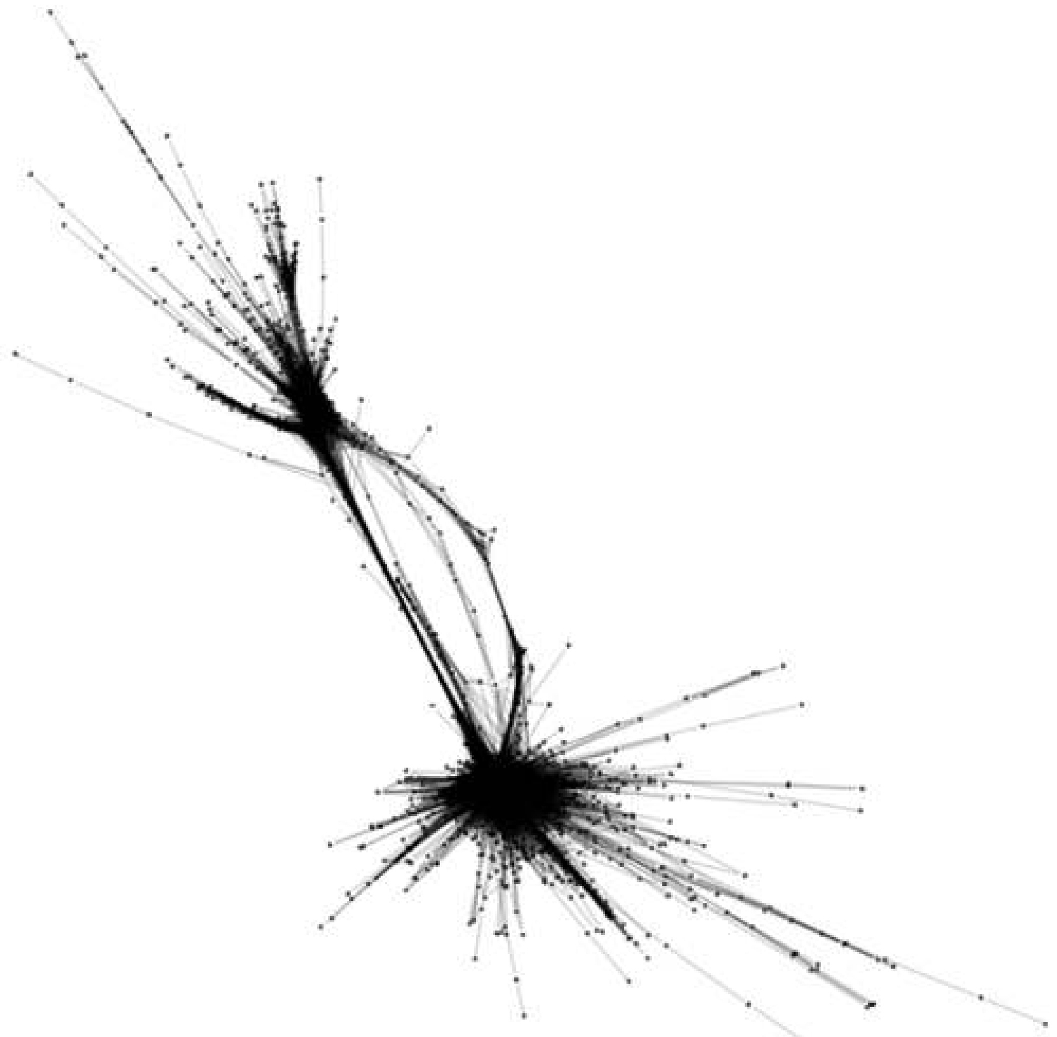

CL220
Low_complexity
Length of Reads (GP):3862 (0.05%)

Tgrandiflorum

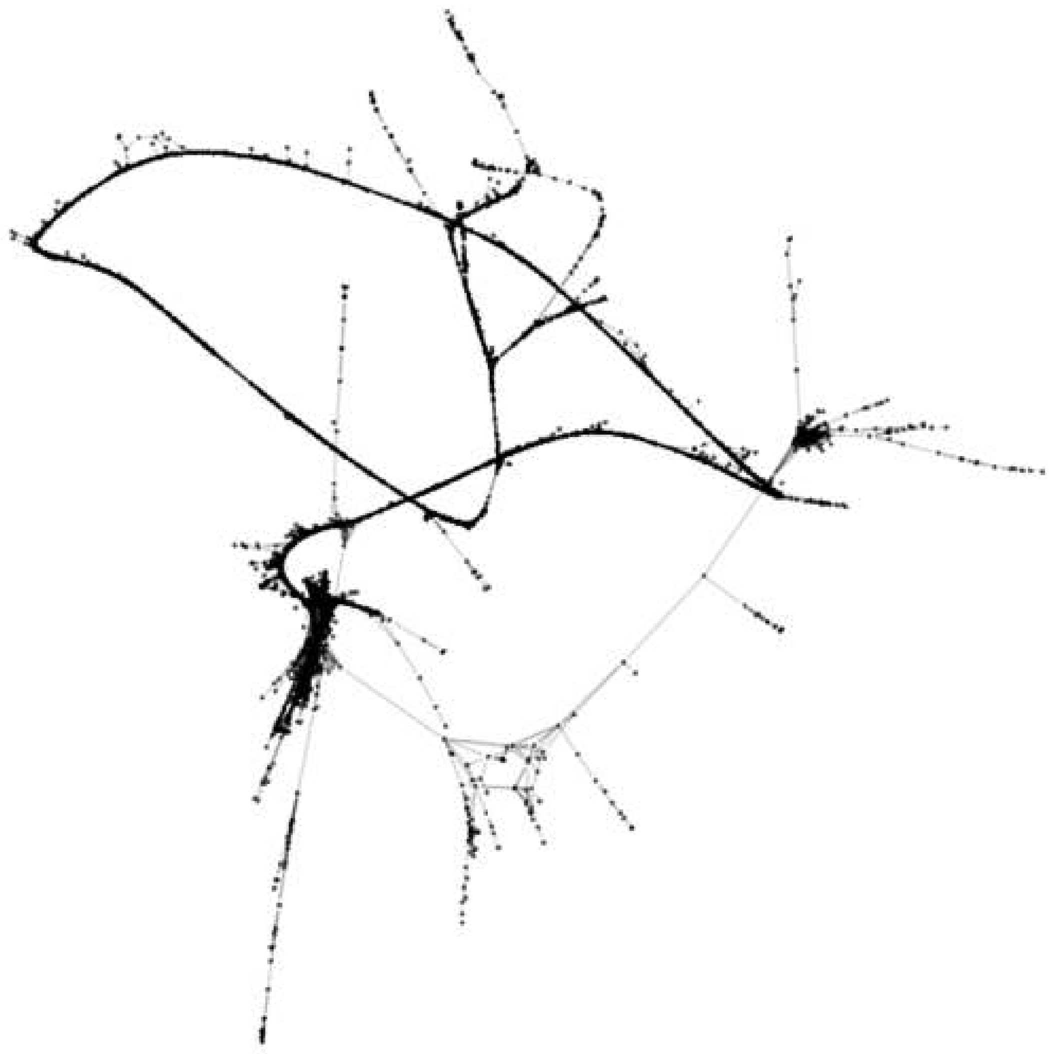

CL221
Low_complexity
Length of Reads (GP):3854 (0.05%)

Tgrandiflorum

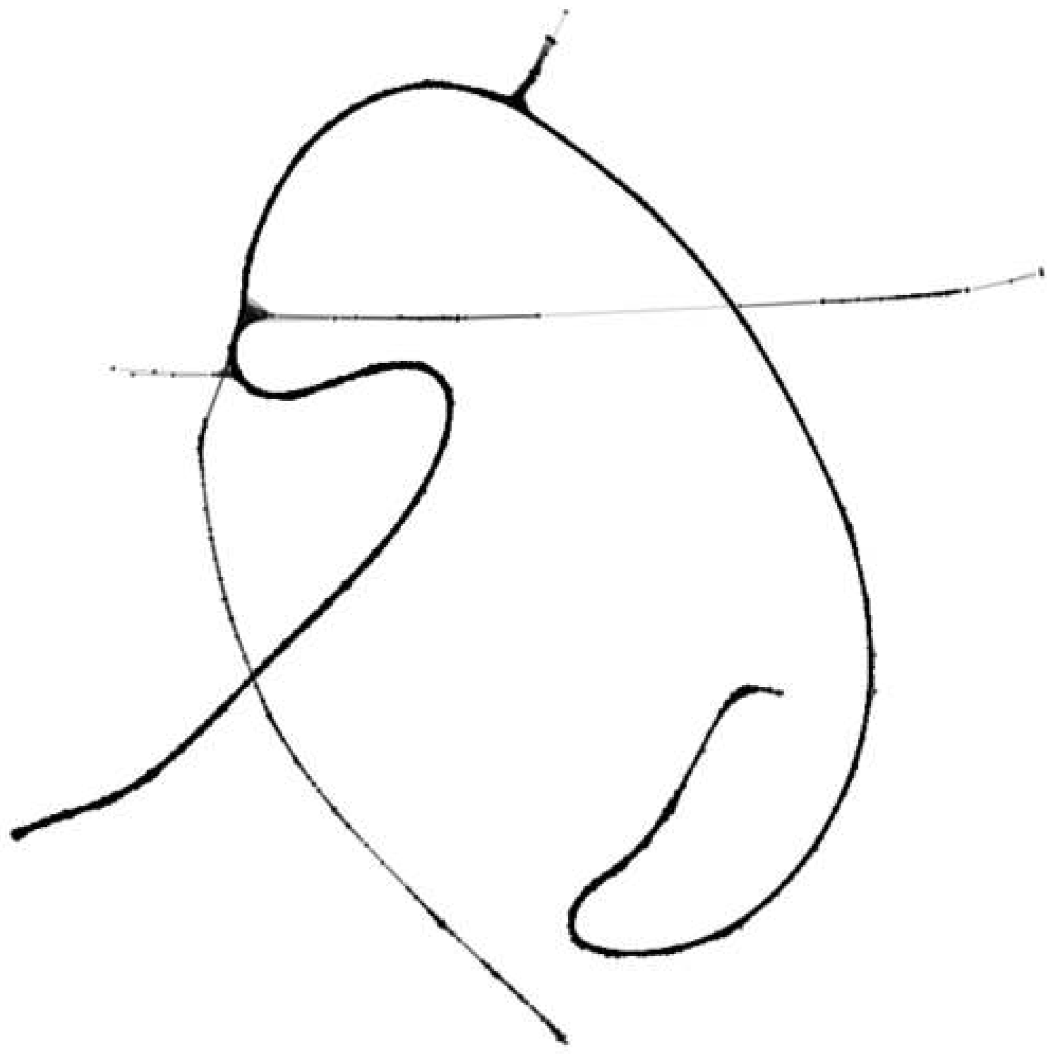

CL222
Low_complexity
Length of Reads (GP):3816 (0.05%)

Tgrandiflorum

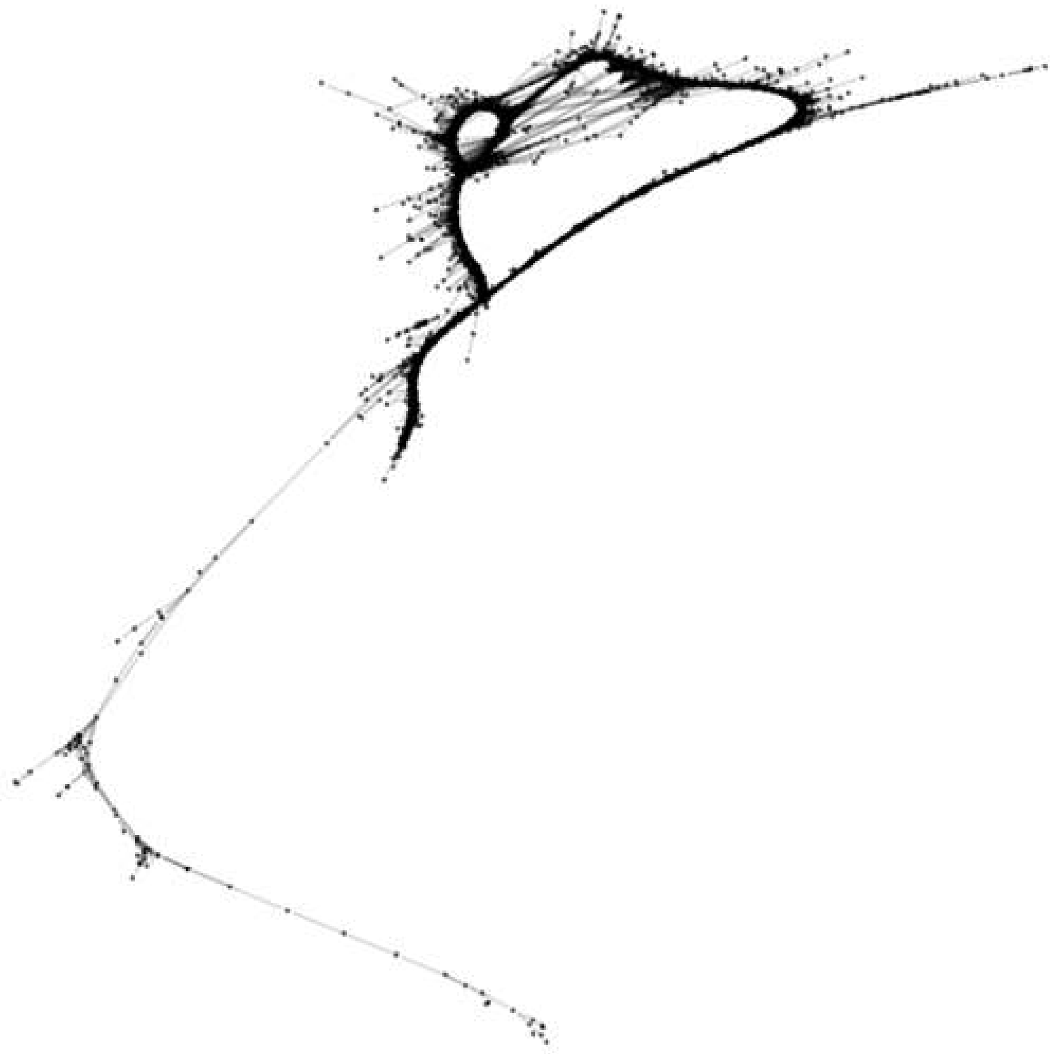

CL223
Low_complexity
Length of Reads (GP):3711 (0.05%)

Tgrandiflorum

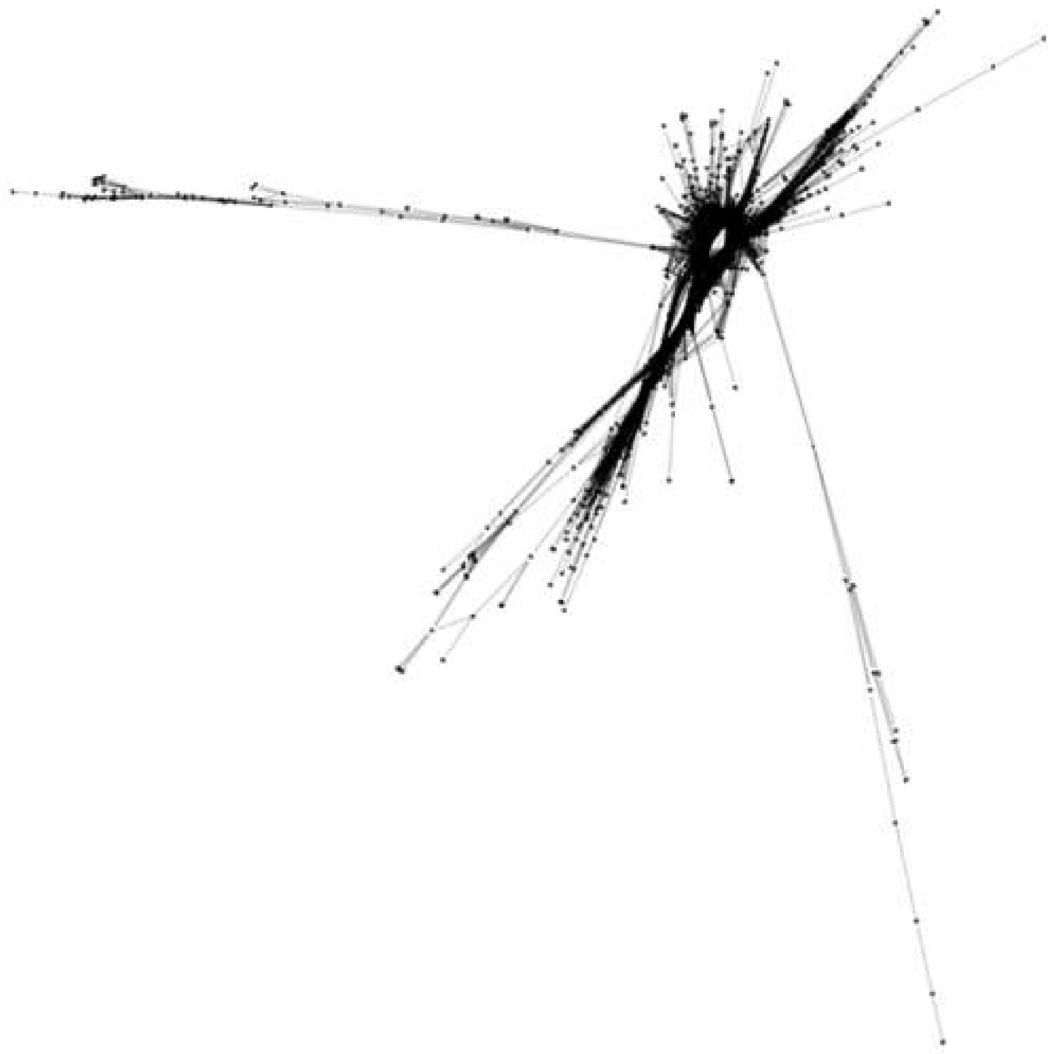

CL224
Low_complexity
Length of Reads (GP):3551 (0.04%)

Tgrandiflorum

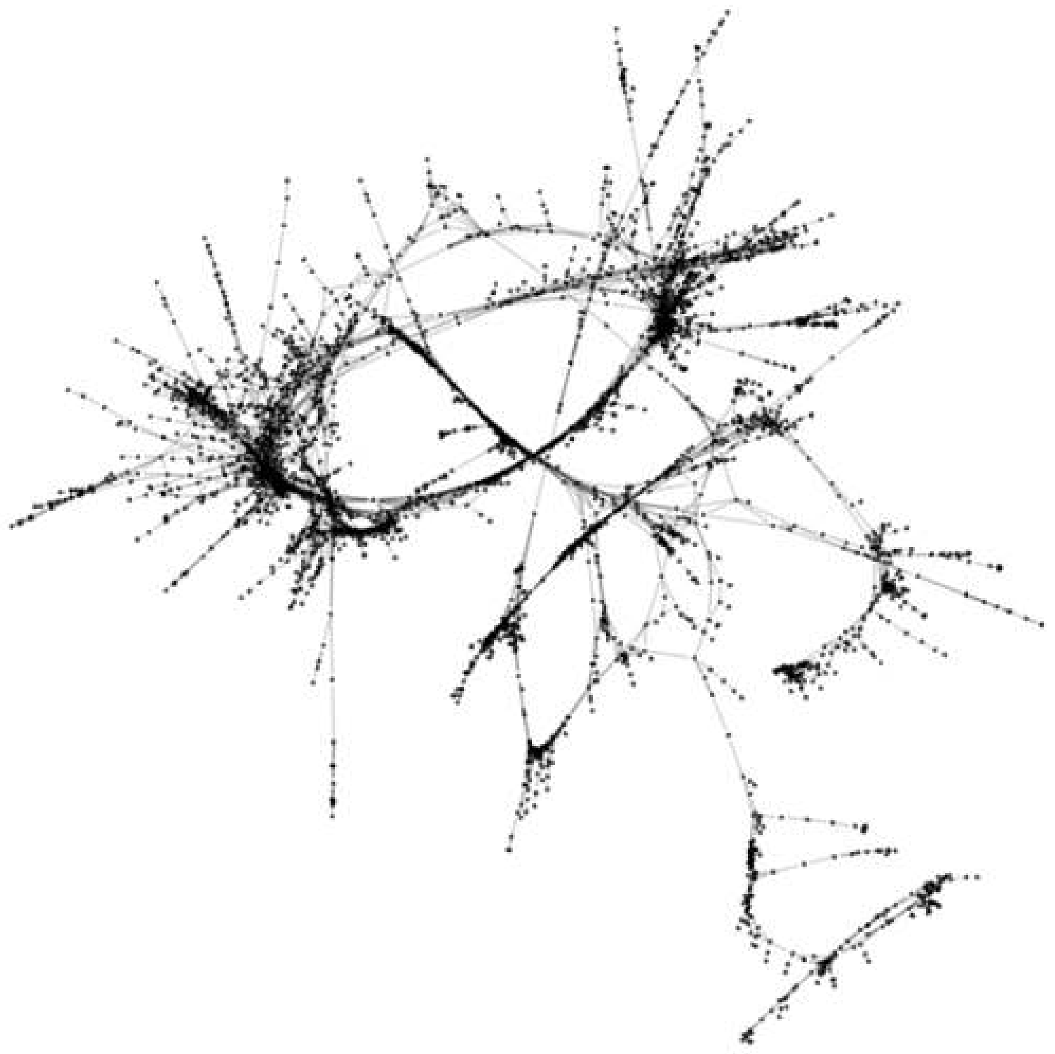

CL225
Simple_repeat
Length of Reads (GP):3447 (0.04%)

Tgrandiflorum

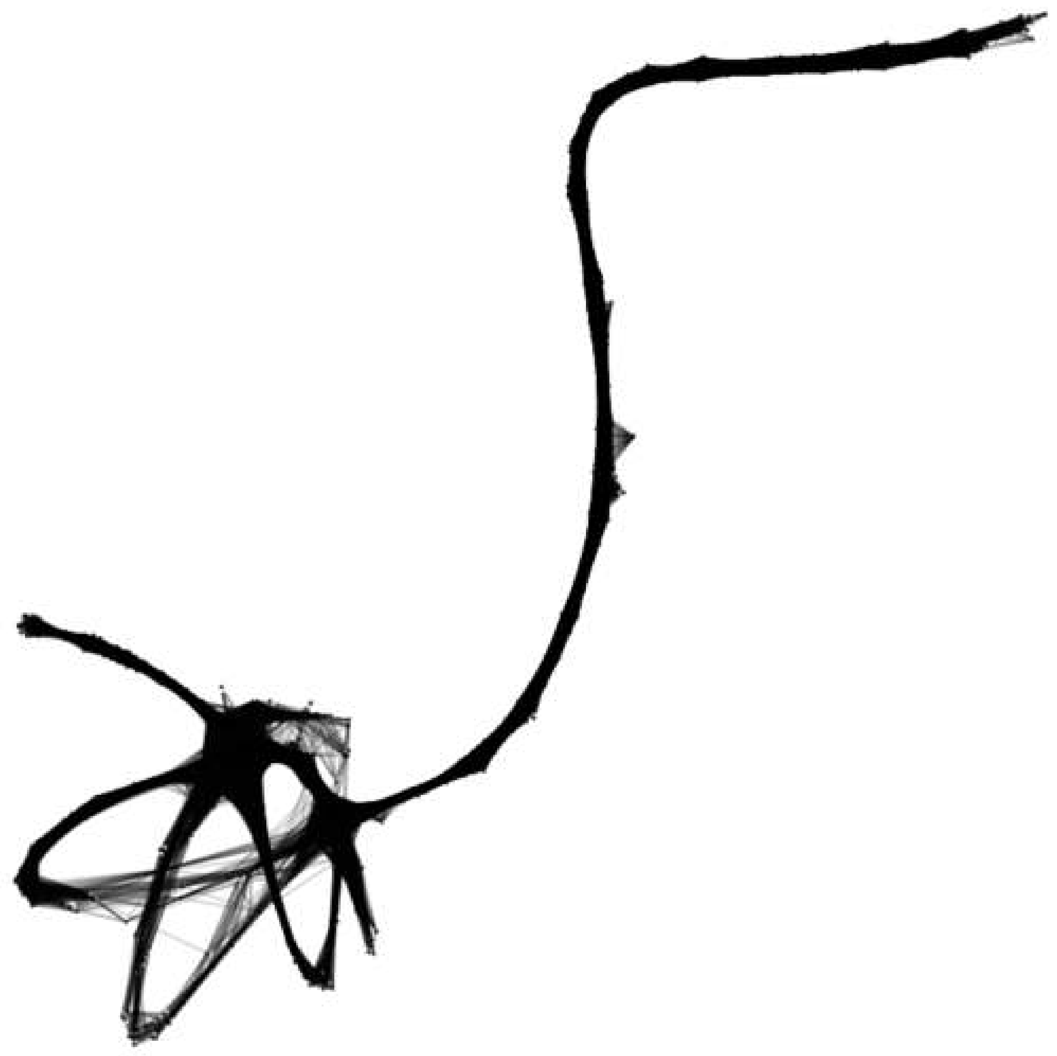

CL226
Low_complexity
Length of Reads (GP):3446 (0.04%)

Tgrandiflorum

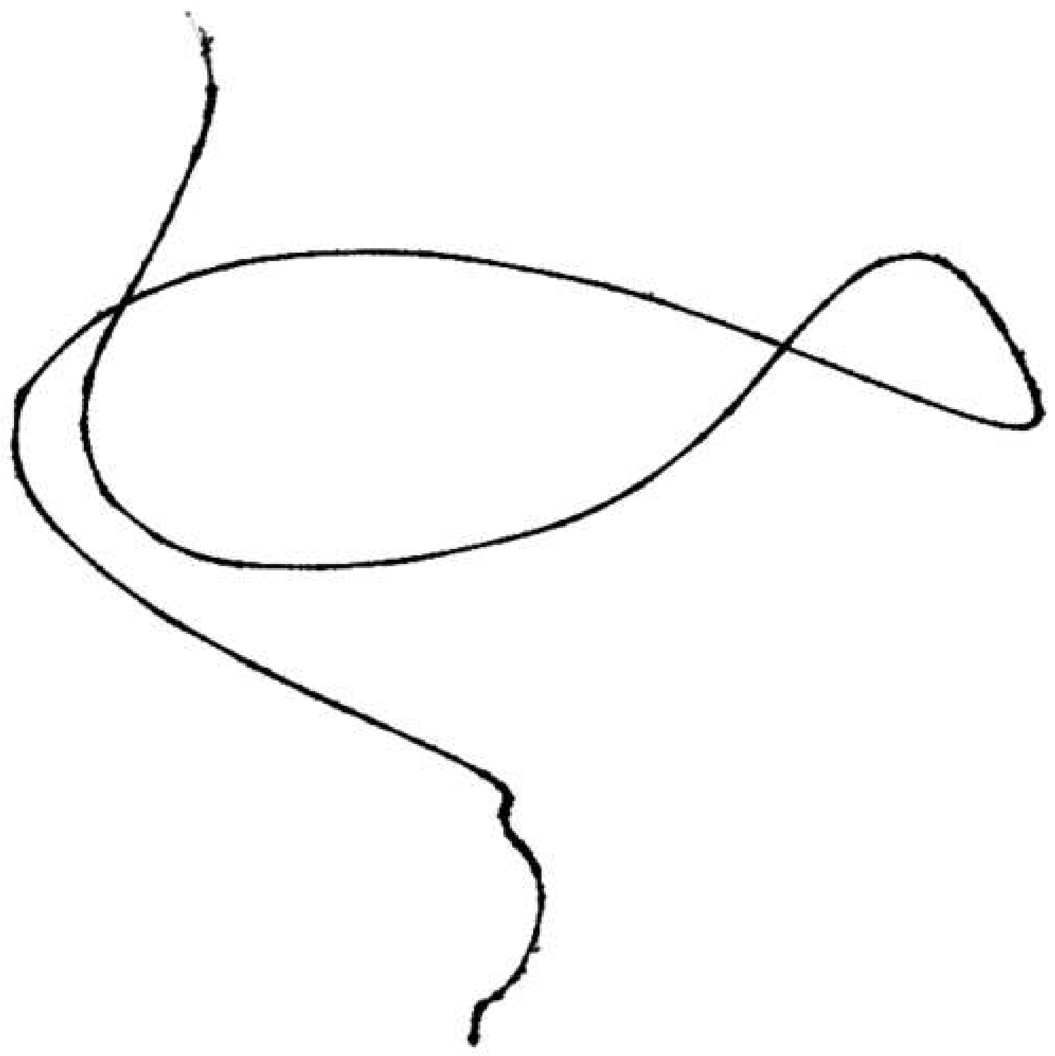

CL227
Low_complexity
Length of Reads (GP):3424 (0.04%)

Tgrandiflorum

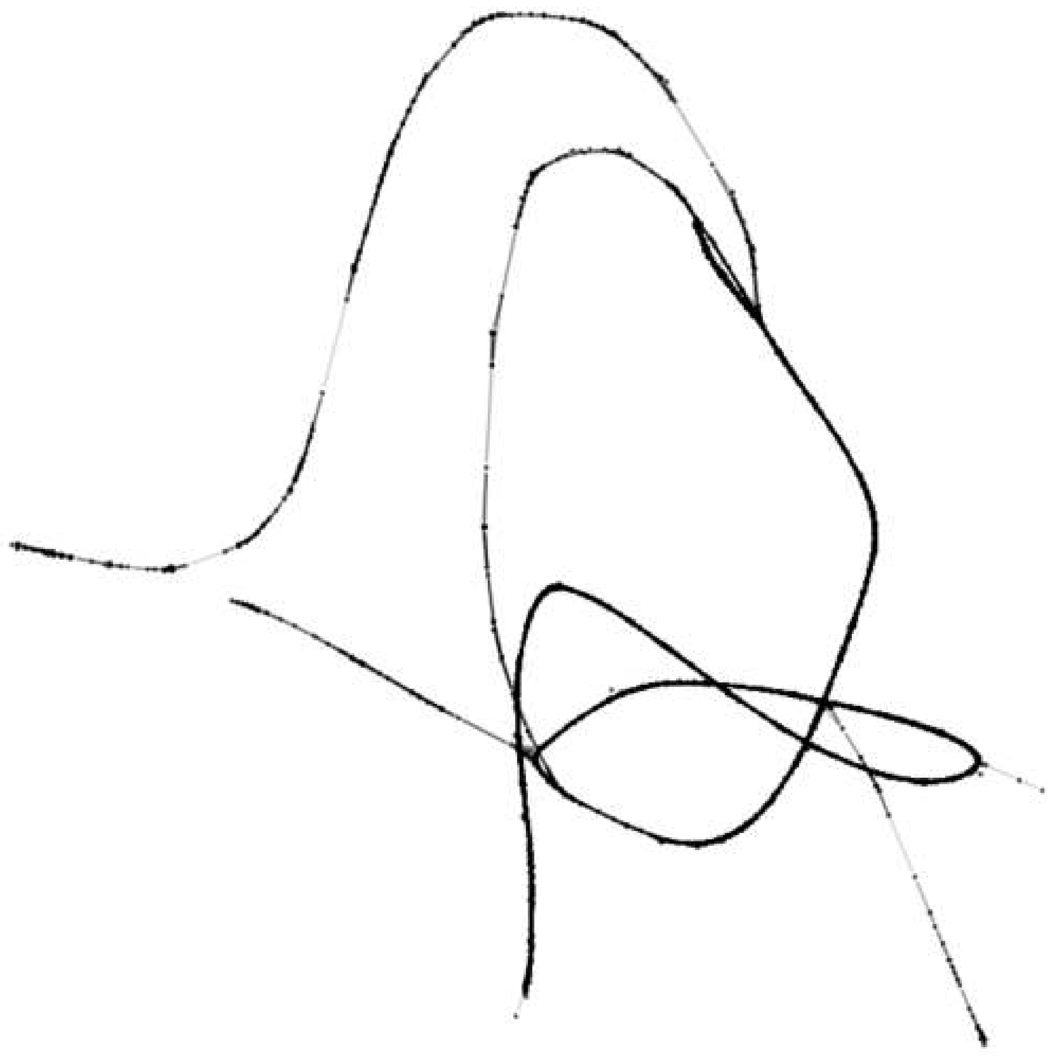

CL228
Low_complexity
Length of Reads (GP):3407 (0.04%)

Tgrandiflorum

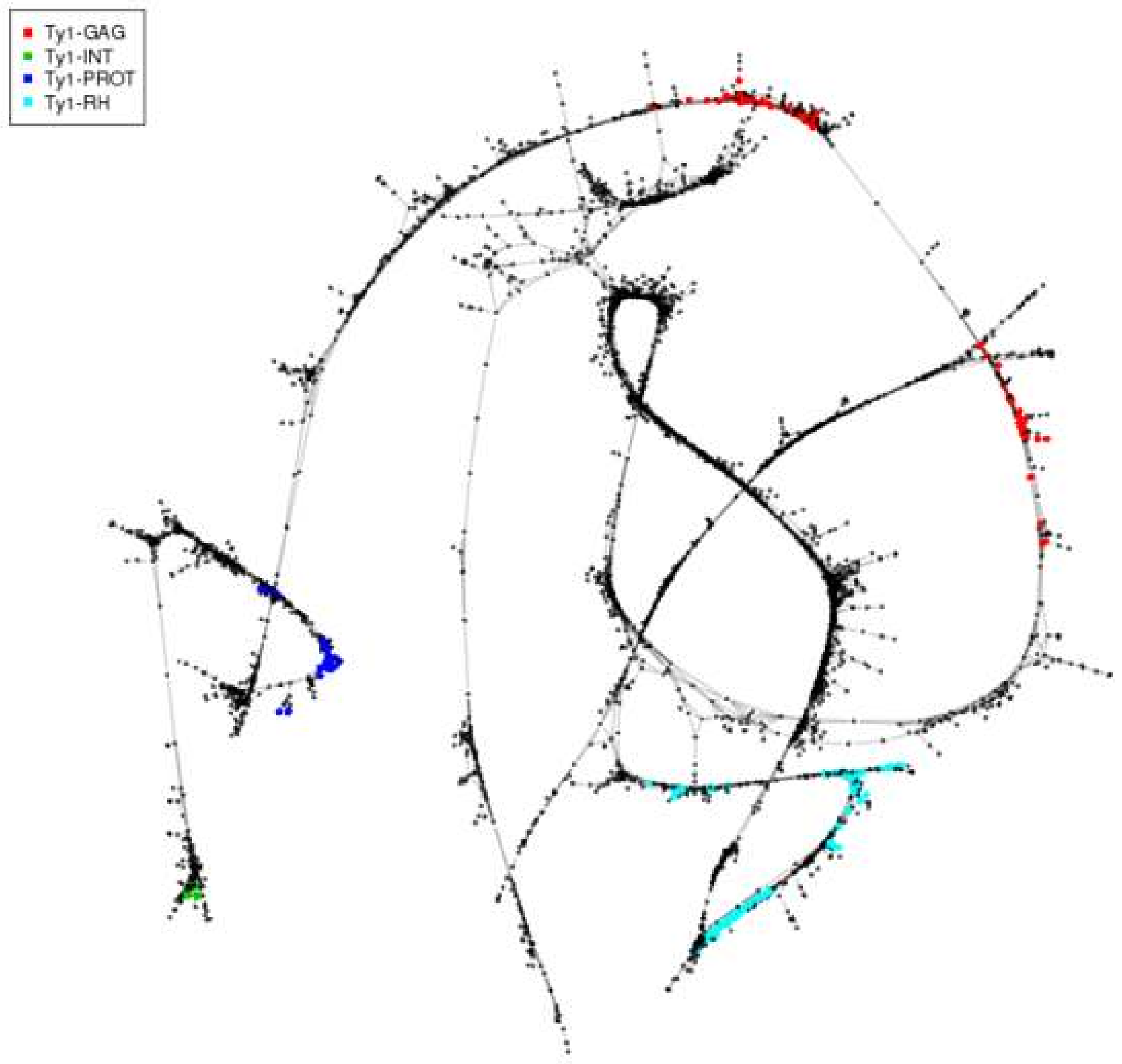

CL229
LTR_Copia
Length of Reads (GP):3367 (0.04%)

Tgrandiflorum

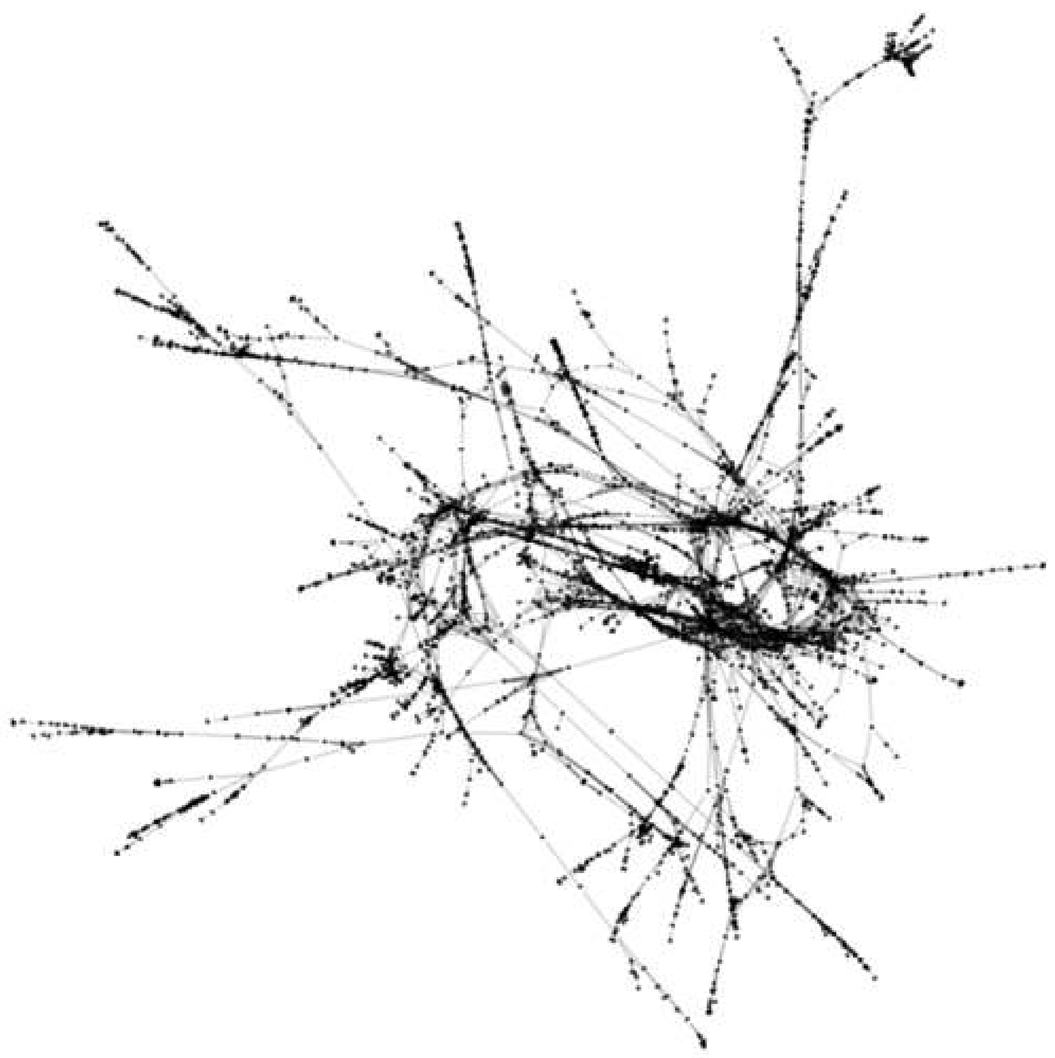

CL230
LTR_Copia
Length of Reads (GP):3331 (0.04%)

Tgrandiflorum

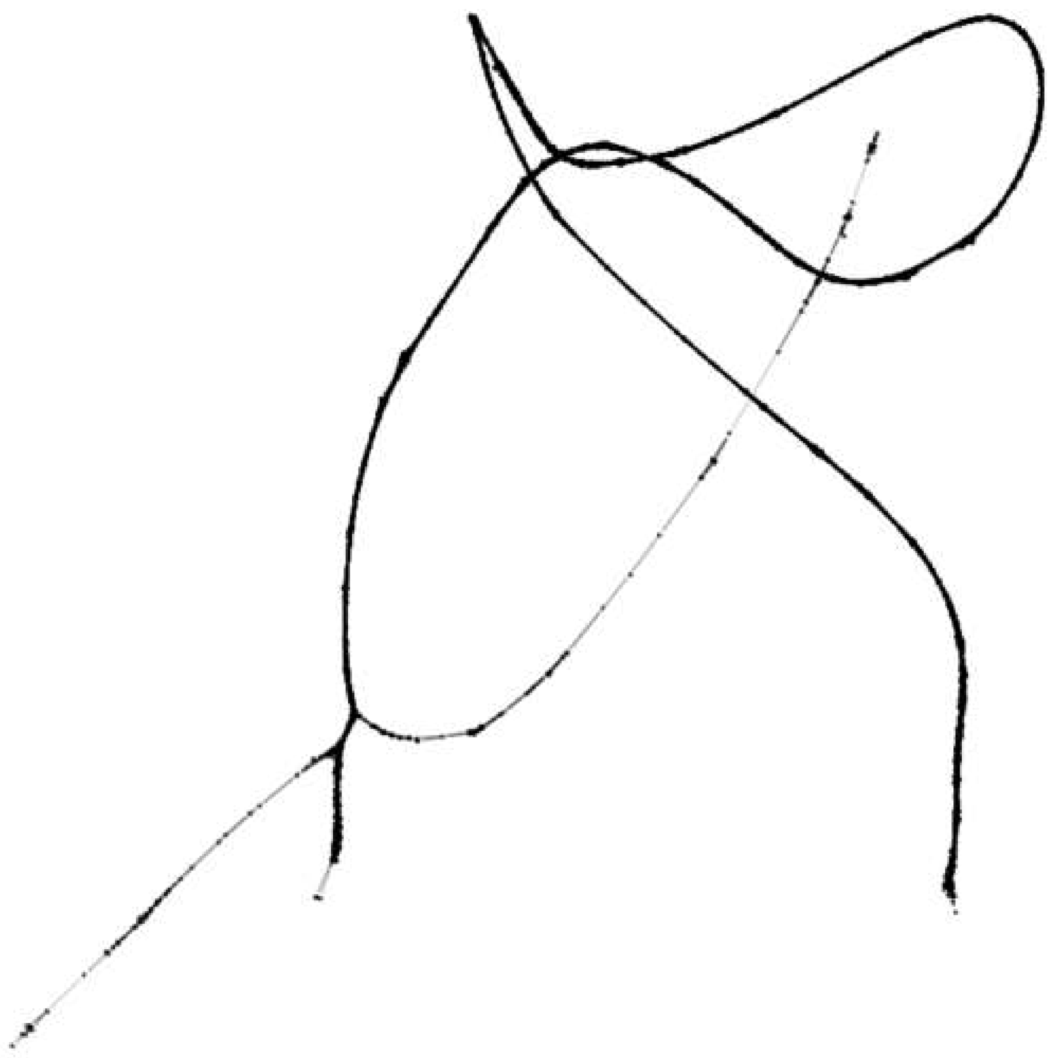

CL231
Low_complexity
Length of Reads (GP):3296 (0.04%)

Tgrandiflorum

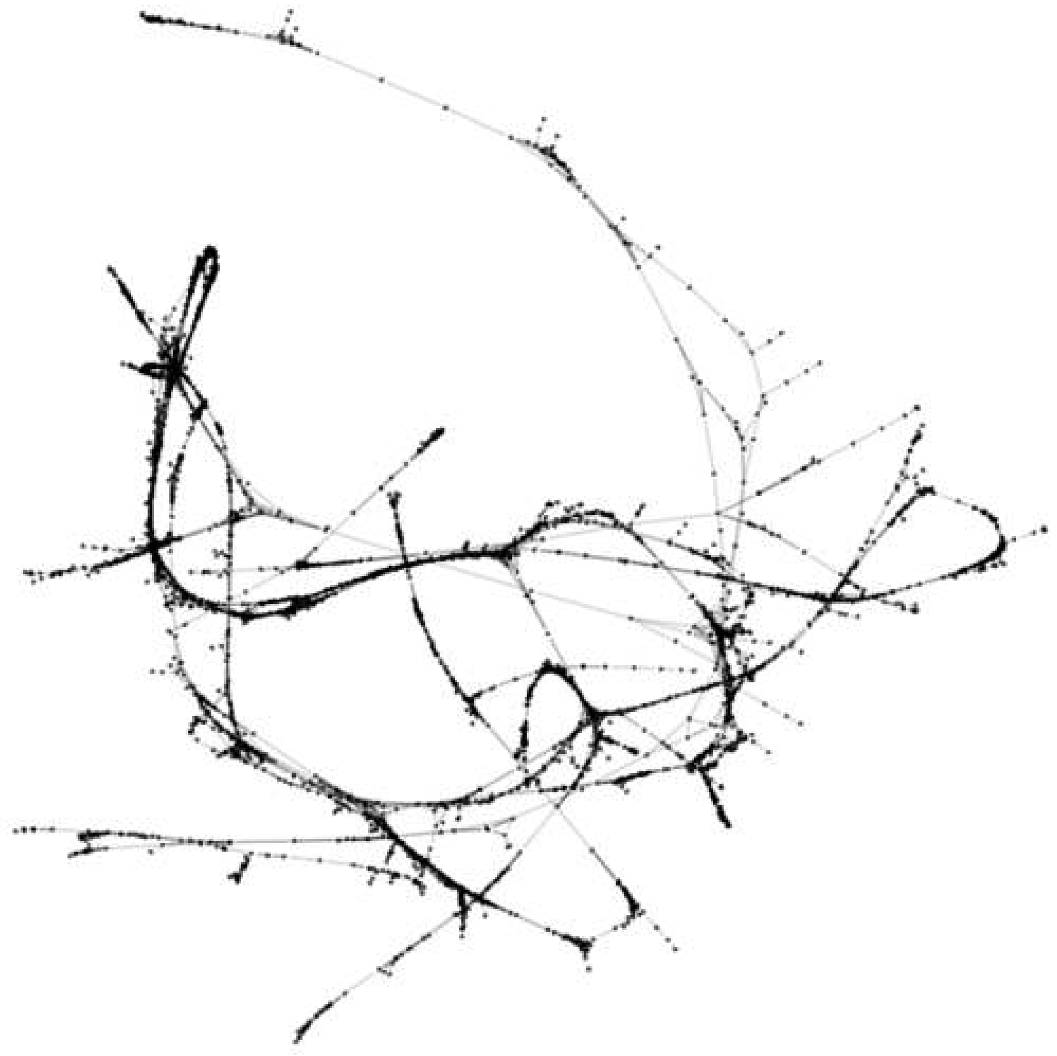

CL232
Low_complexity
Length of Reads (GP):3224 (0.04%)

Tgrandiflorum

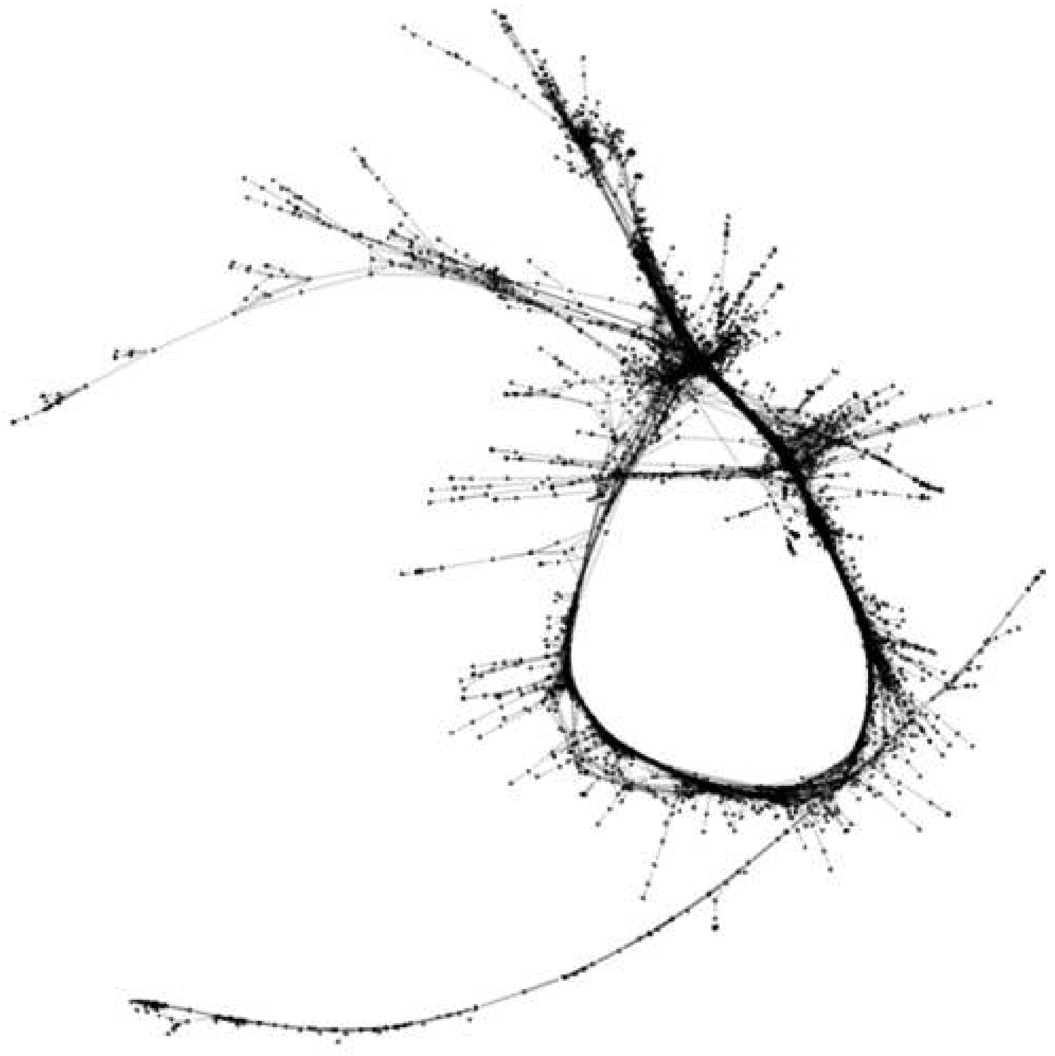

CL233
Low_complexity
Length of Reads (GP):3199 (0.04%)

Tgrandiflorum

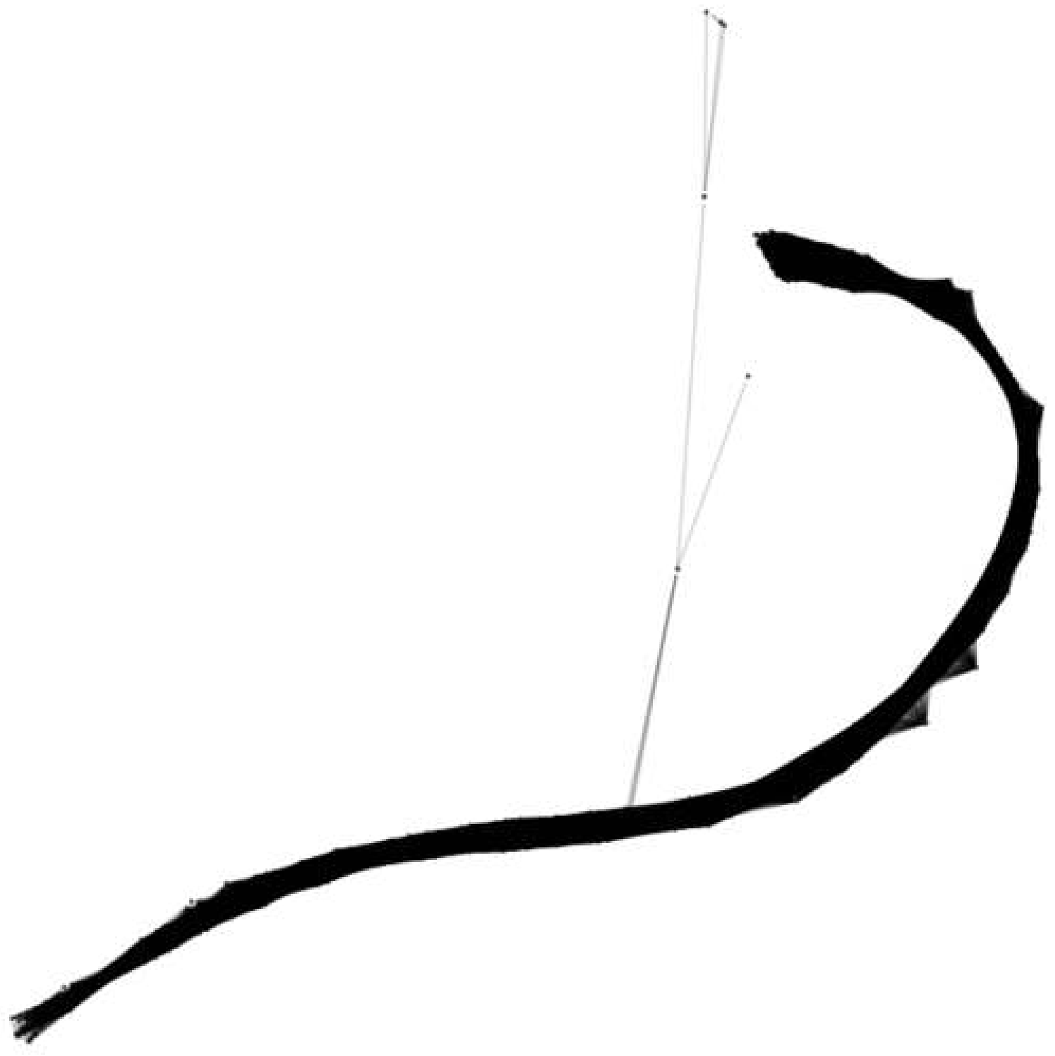

CL234
rRNA
Length of Reads (GP):3040 (0.04%)

Tgrandiflorum

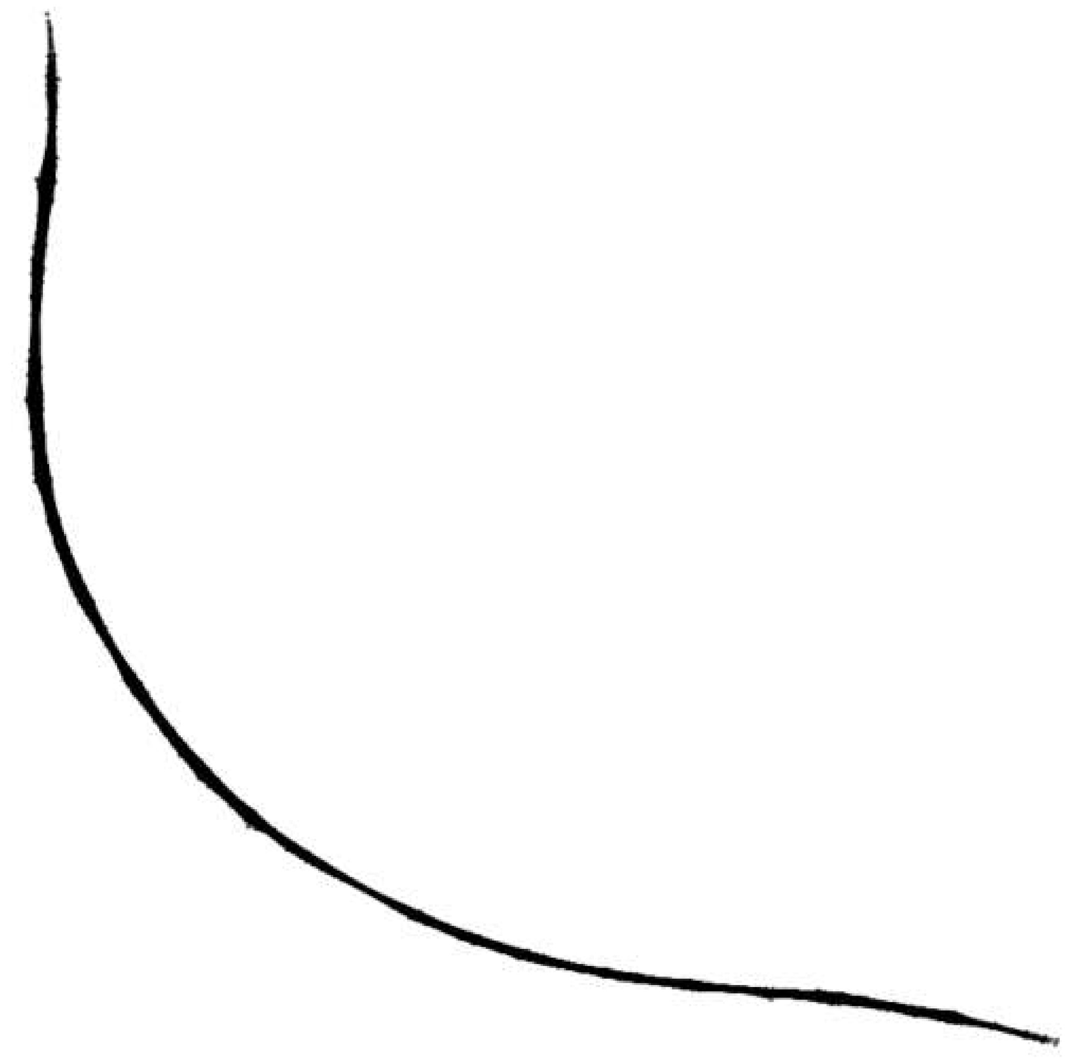

CL235
rRNA
Length of Reads (GP):2978 (0.04%)

Tgrandiflorum

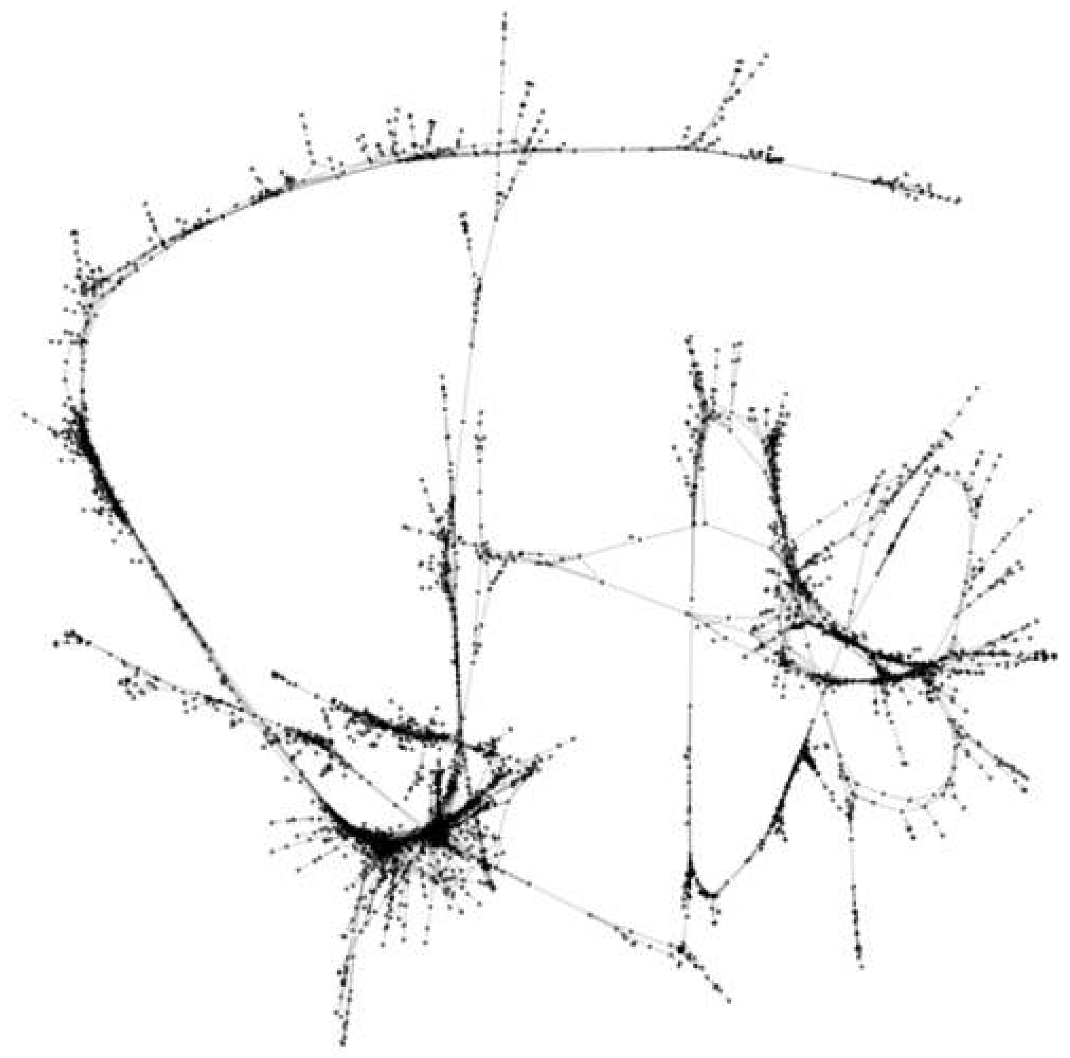

CL236
Low_complexity
Length of Reads (GP):2969 (0.04%)

Tgrandiflorum

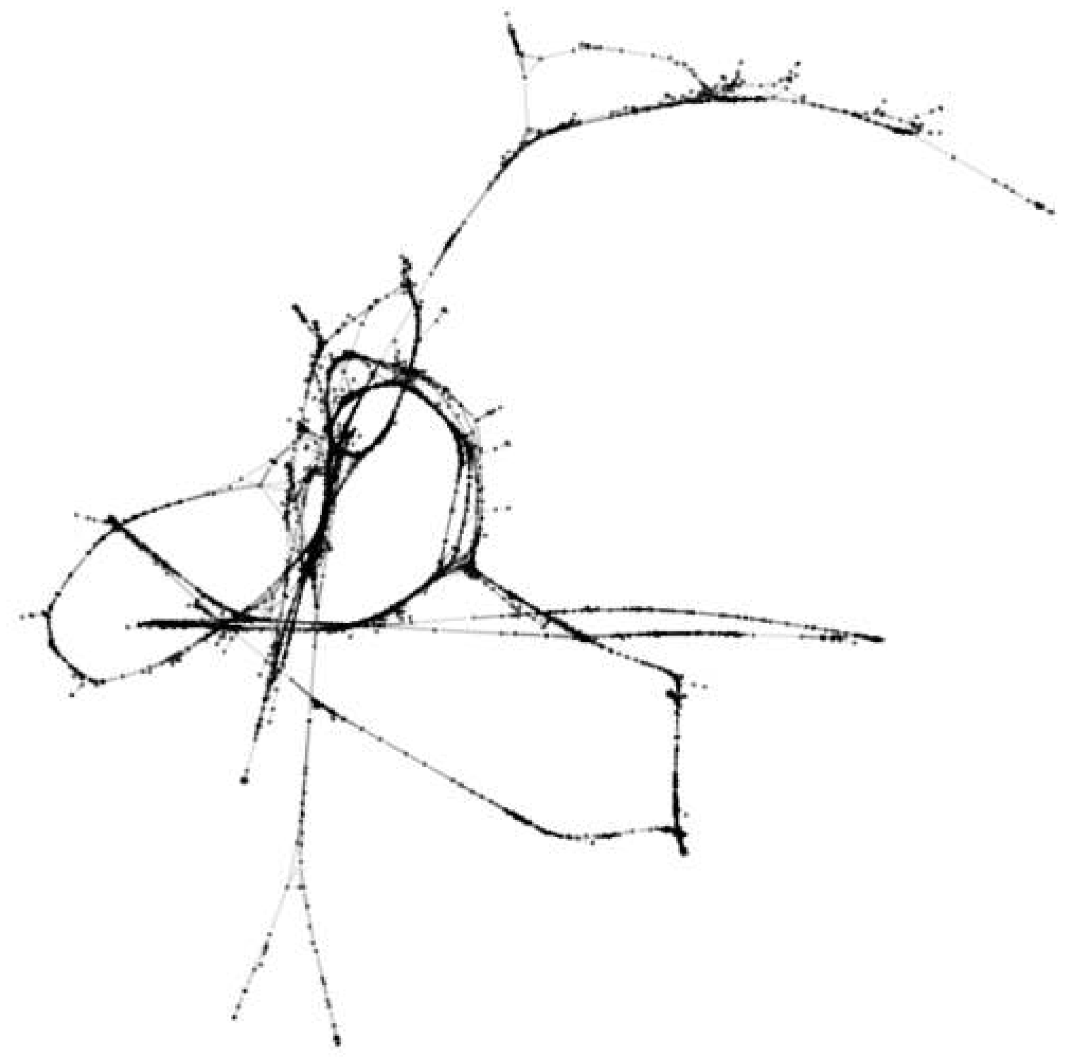

CL237
Low_complexity
Length of Reads (GP):2953 (0.04%)

Tgrandiflorum

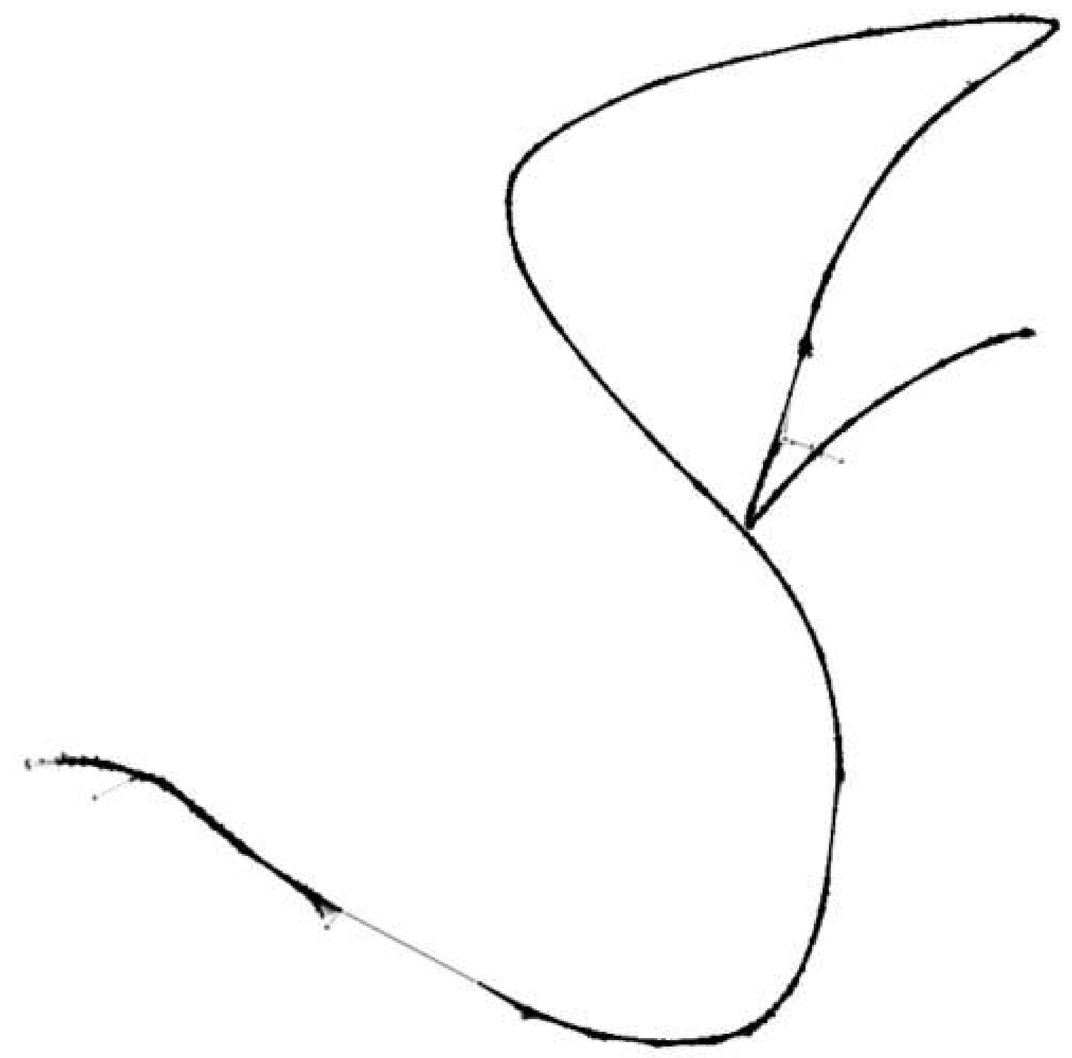

CL238
LTR_Copia
Length of Reads (GP):2932 (0.04%)

Tgrandiflorum

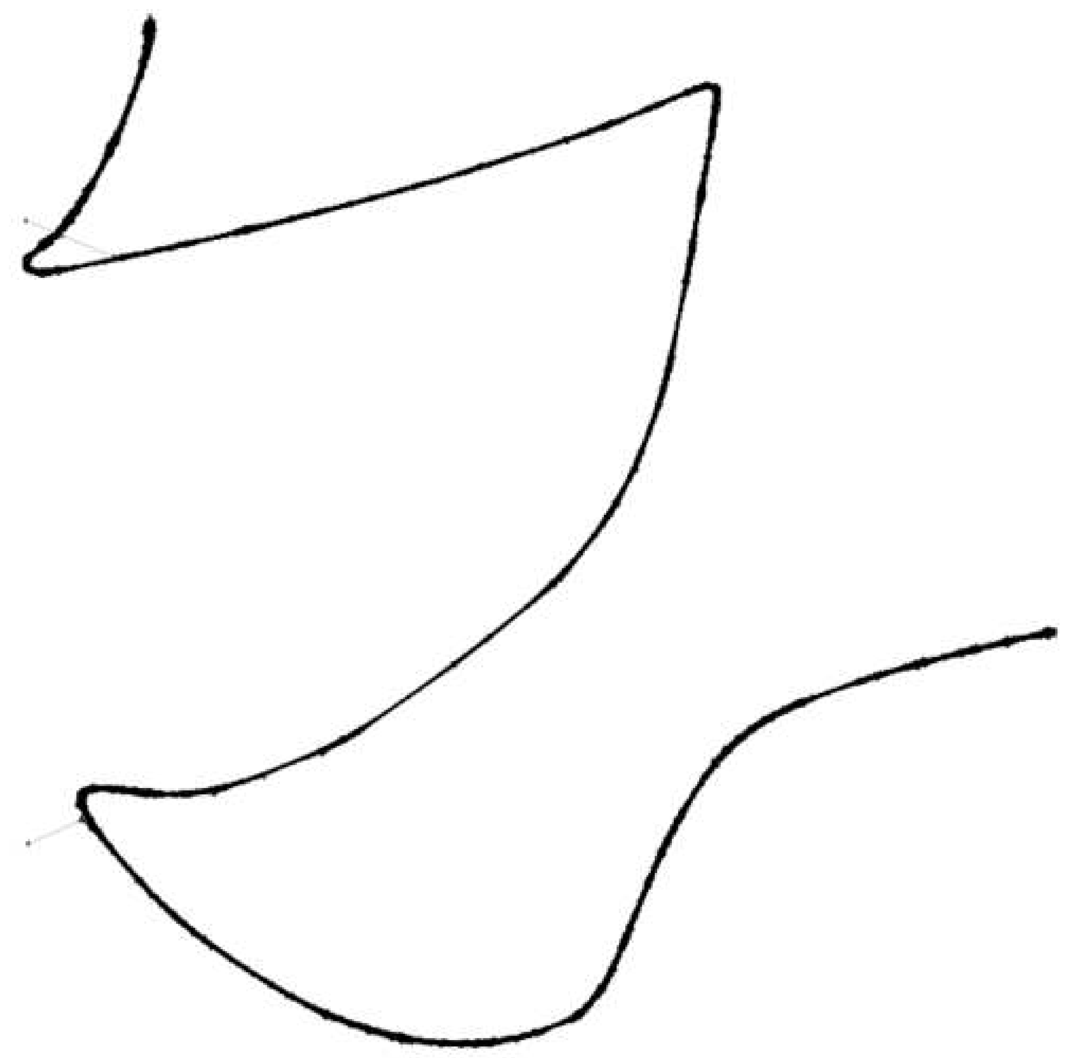

CL239
Low_complexity
Length of Reads (GP):2928 (0.04%)

Tgrandiflorum

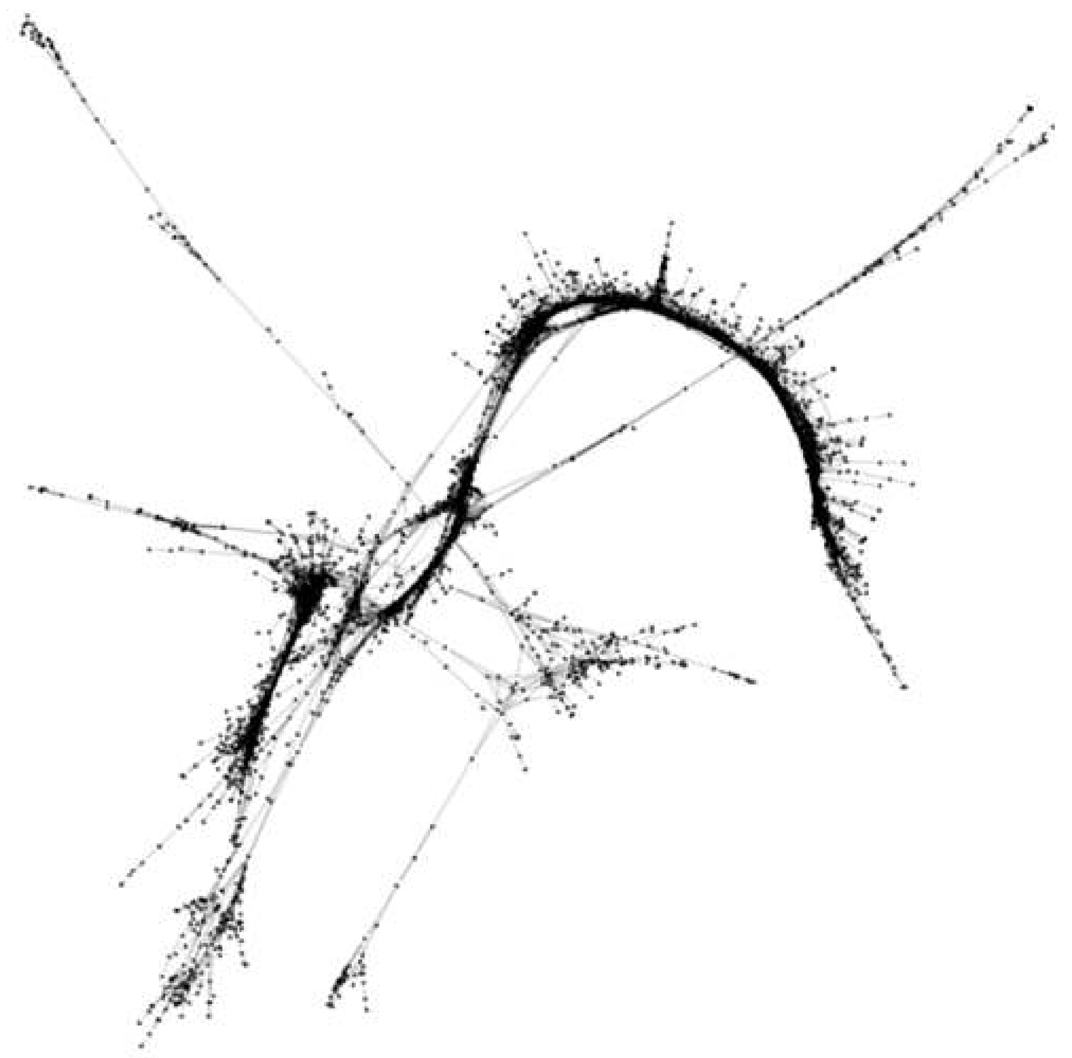

CL240
Low_complexity
Length of Reads (GP):2900 (0.04%)

Tgrandiflorum

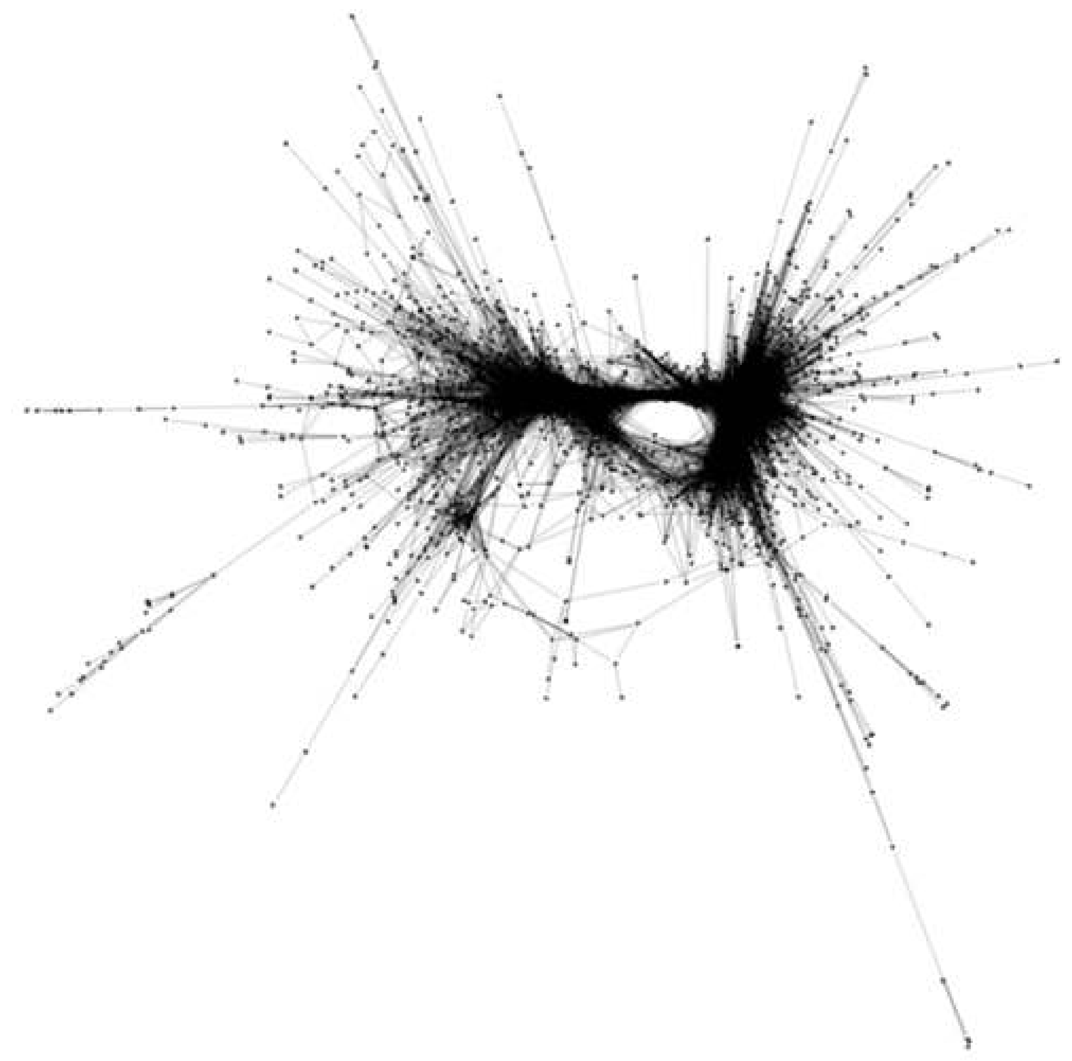

CL241
LTR_Gypsy
Length of Reads (GP):2885 (0.04%)

Tgrandiflorum

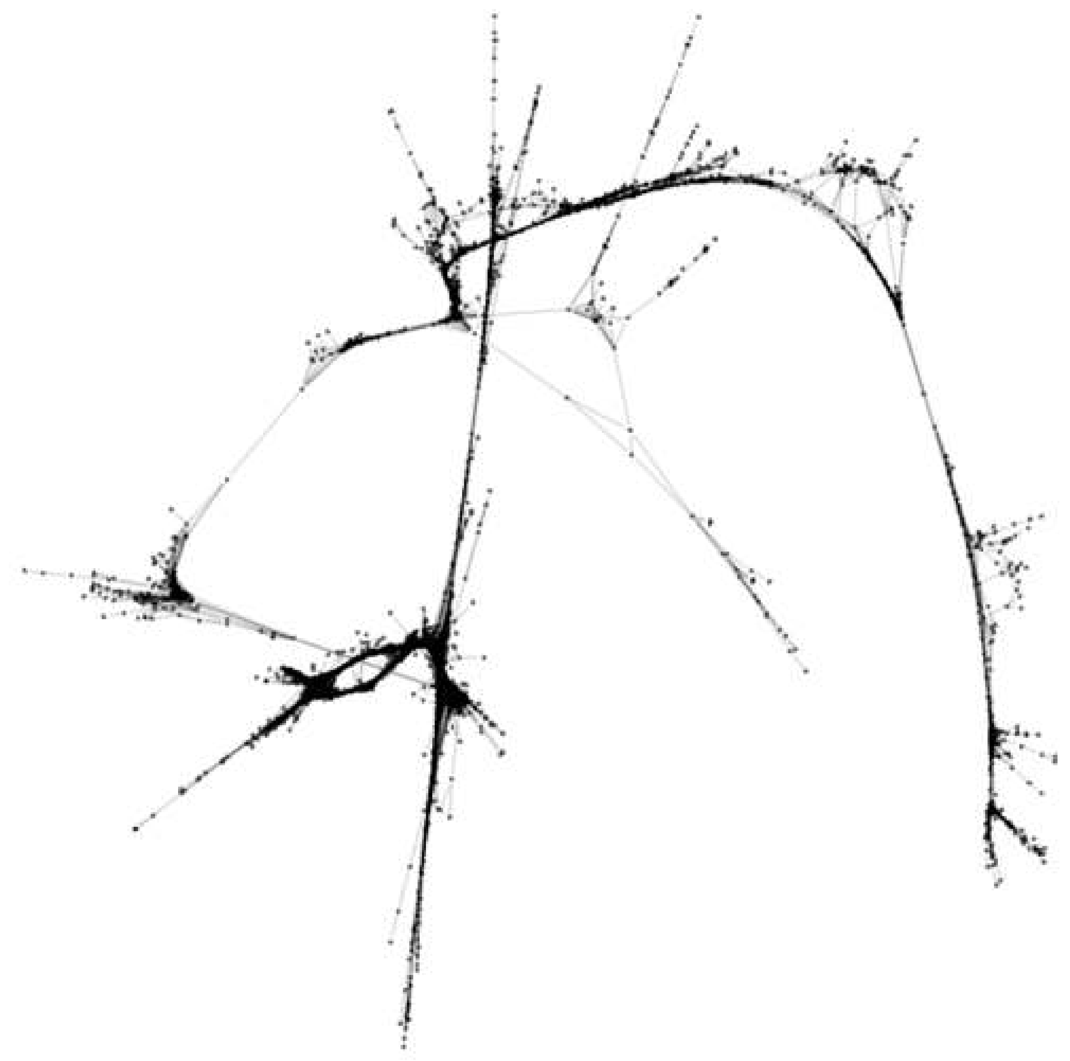

CL242
Low_complexity
Length of Reads (GP):2779 (0.03%)

Tgrandiflorum

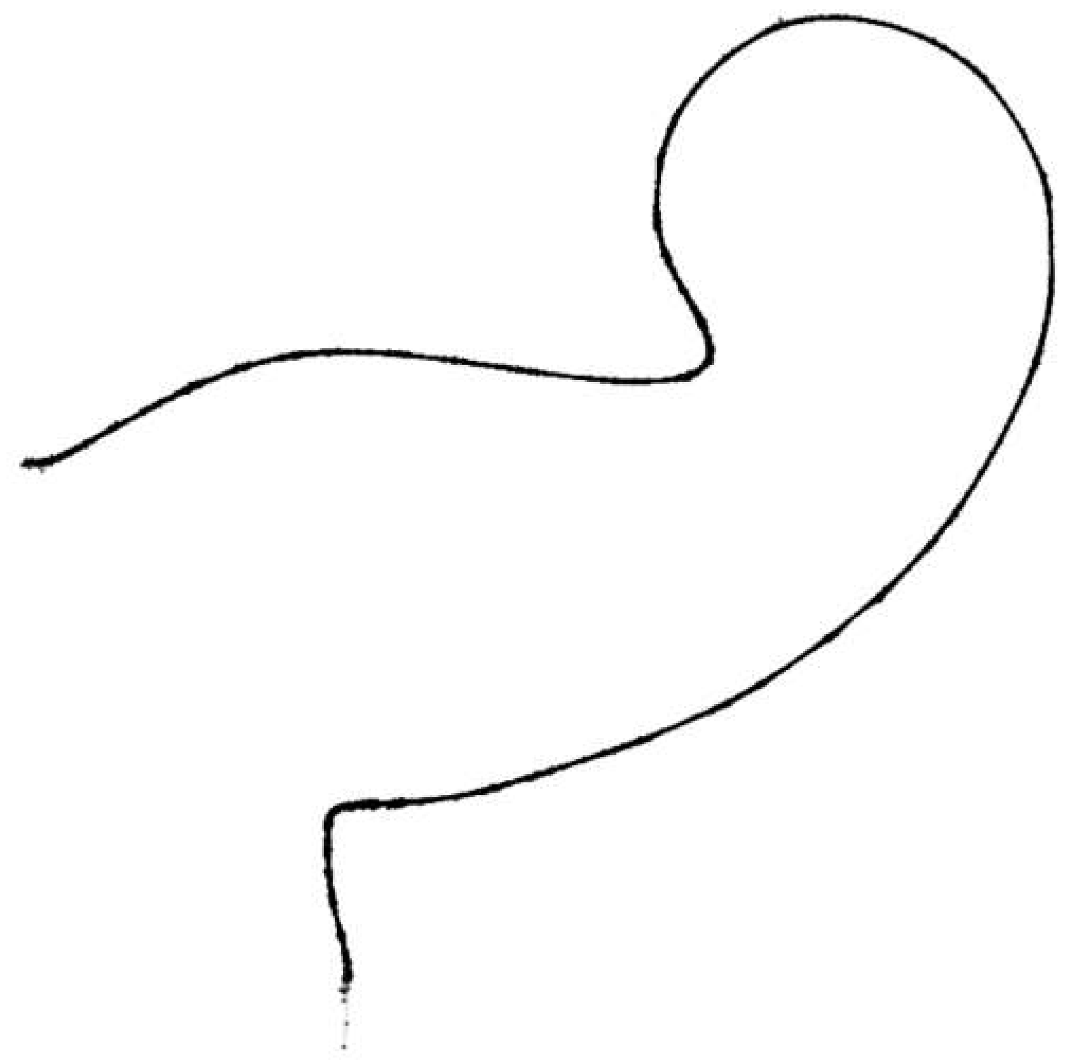

CL243
Simple_repeat
Length of Reads (GP):2669 (0.03%)

Tgrandiflorum

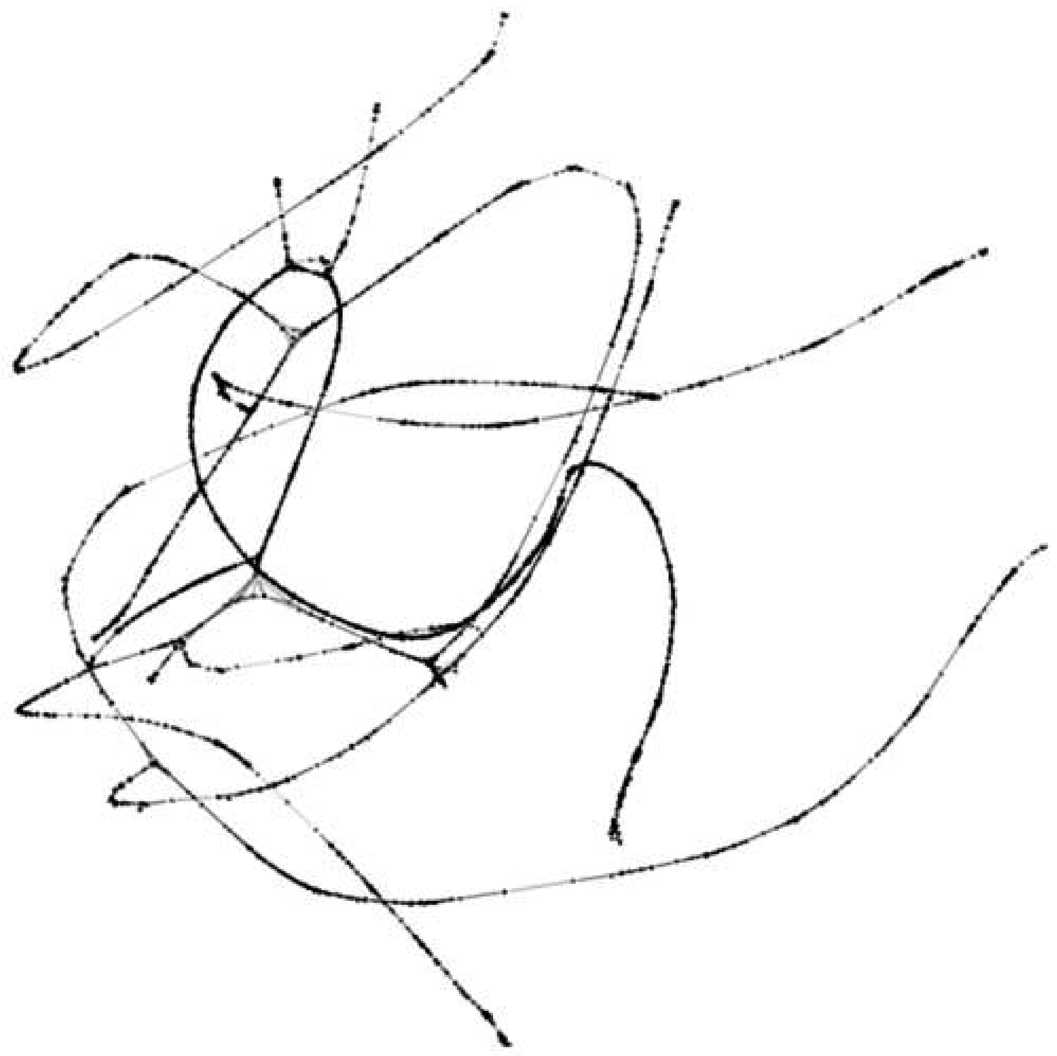

CL244
Low_complexity
Length of Reads (GP):2647 (0.03%)

Tgrandiflorum

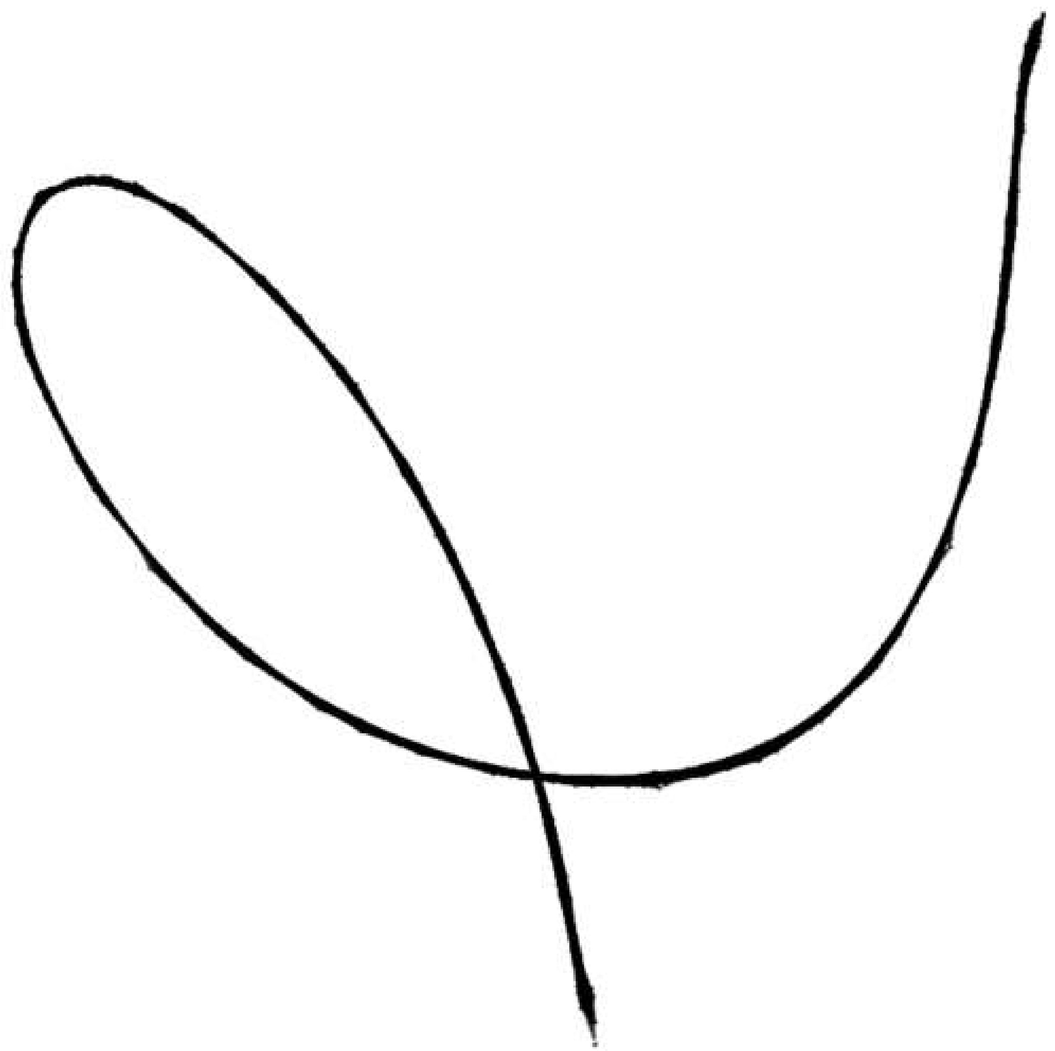

CL245
LTR_Copia
Length of Reads (GP):2541 (0.03%)

Tgrandiflorum

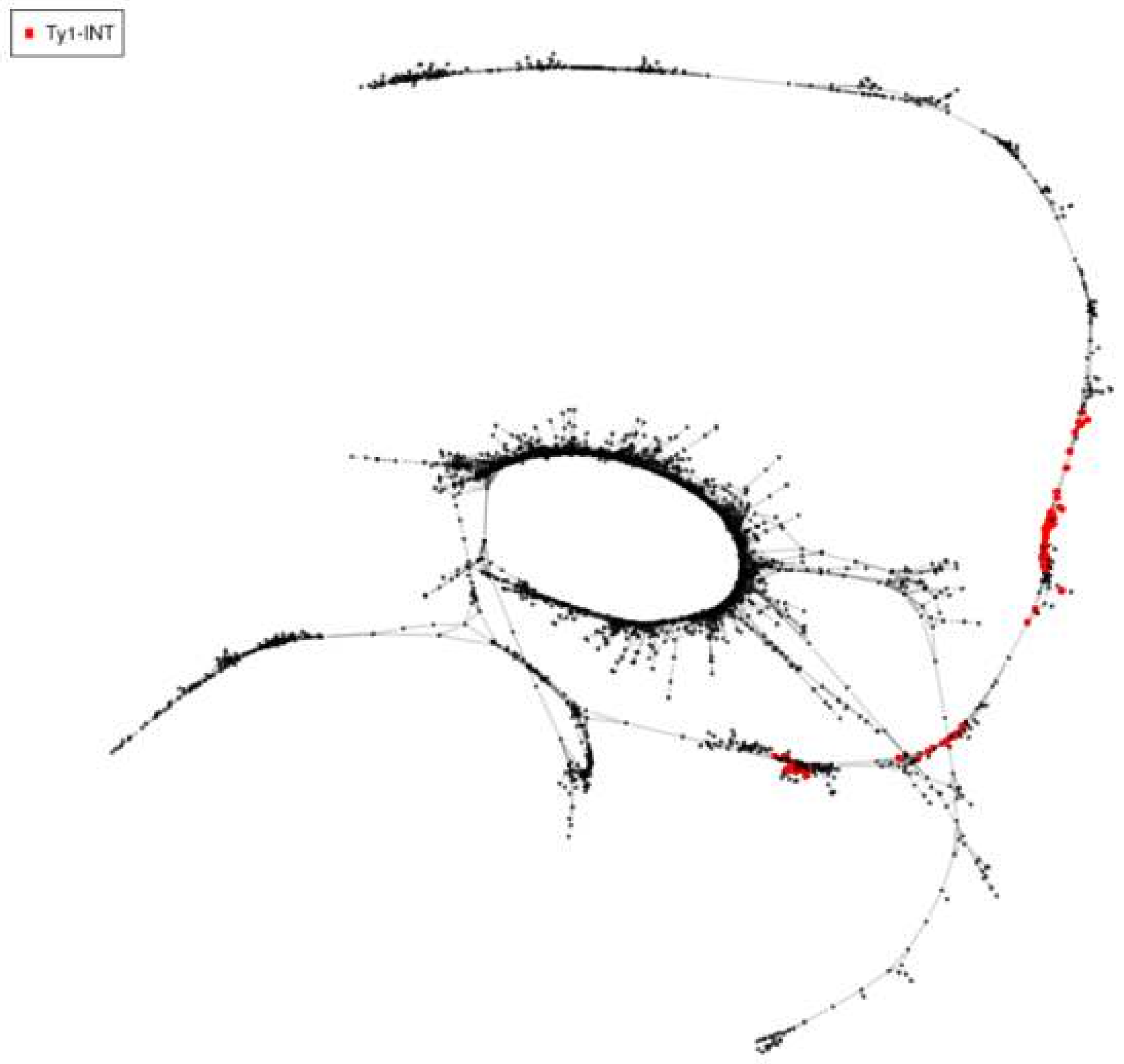

CL246
LTR_Copia
Length of Reads (GP):2462 (0.03%)

Tgrandiflorum

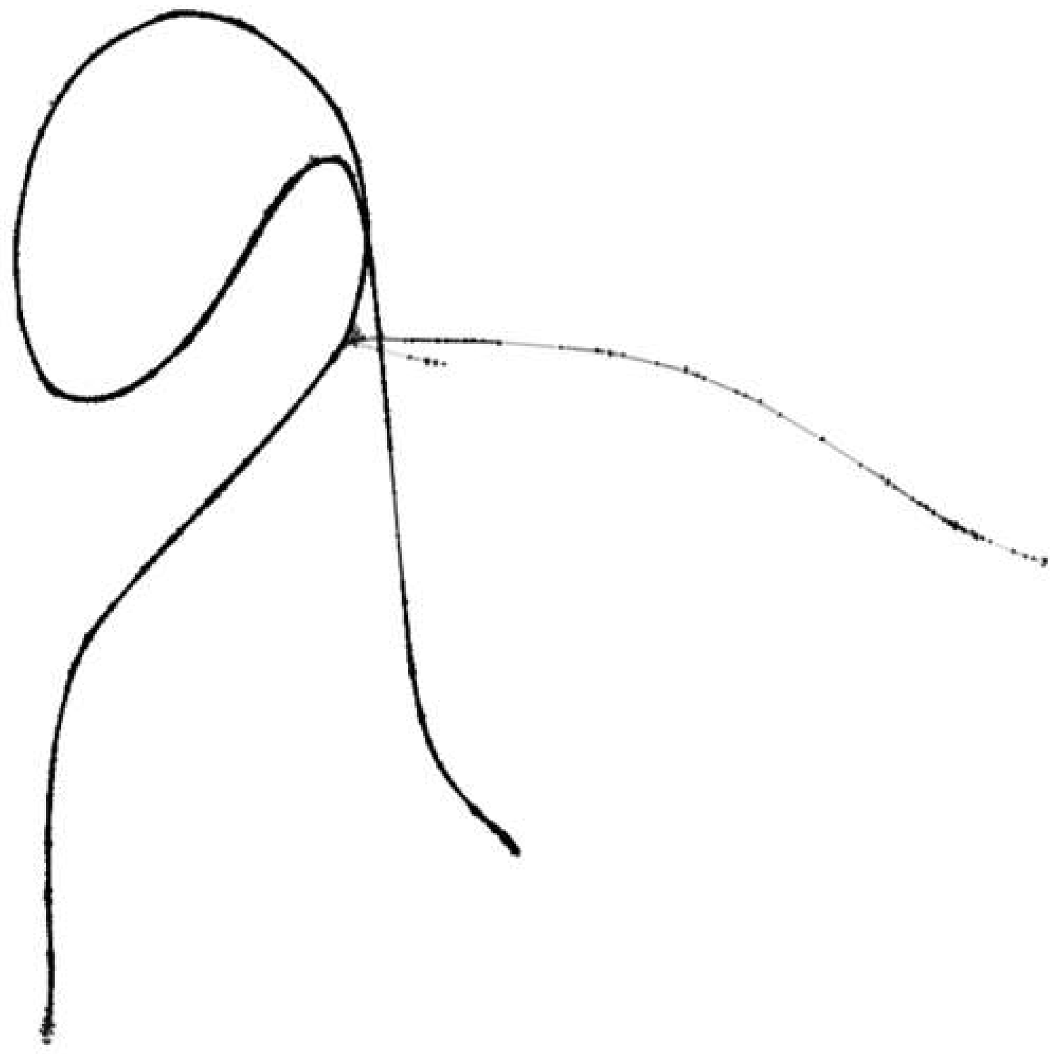

CL247
Low_complexity
Length of Reads (GP):2415 (0.03%)

Tgrandiflorum

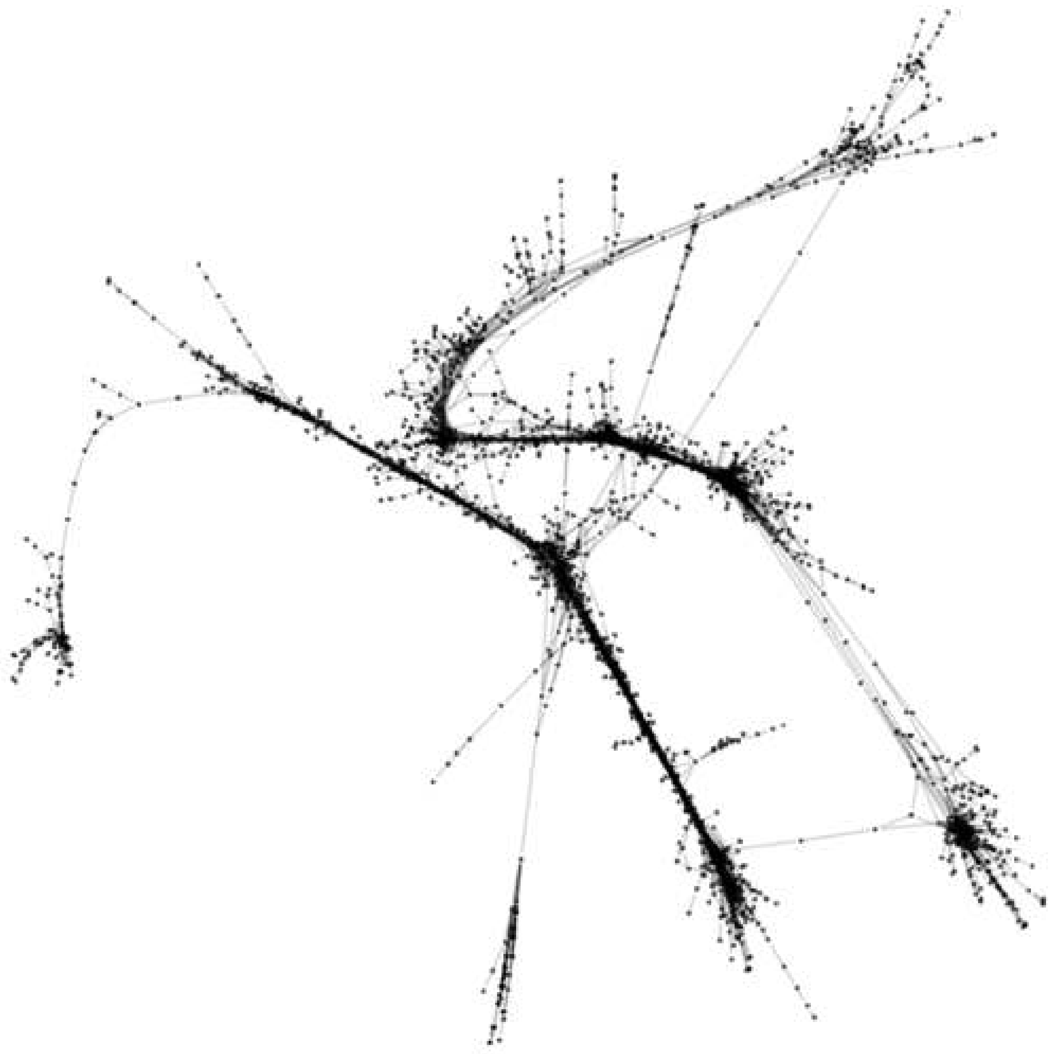

CL248
Low_complexity
Length of Reads (GP):2401 (0.03%)

Tgrandiflorum

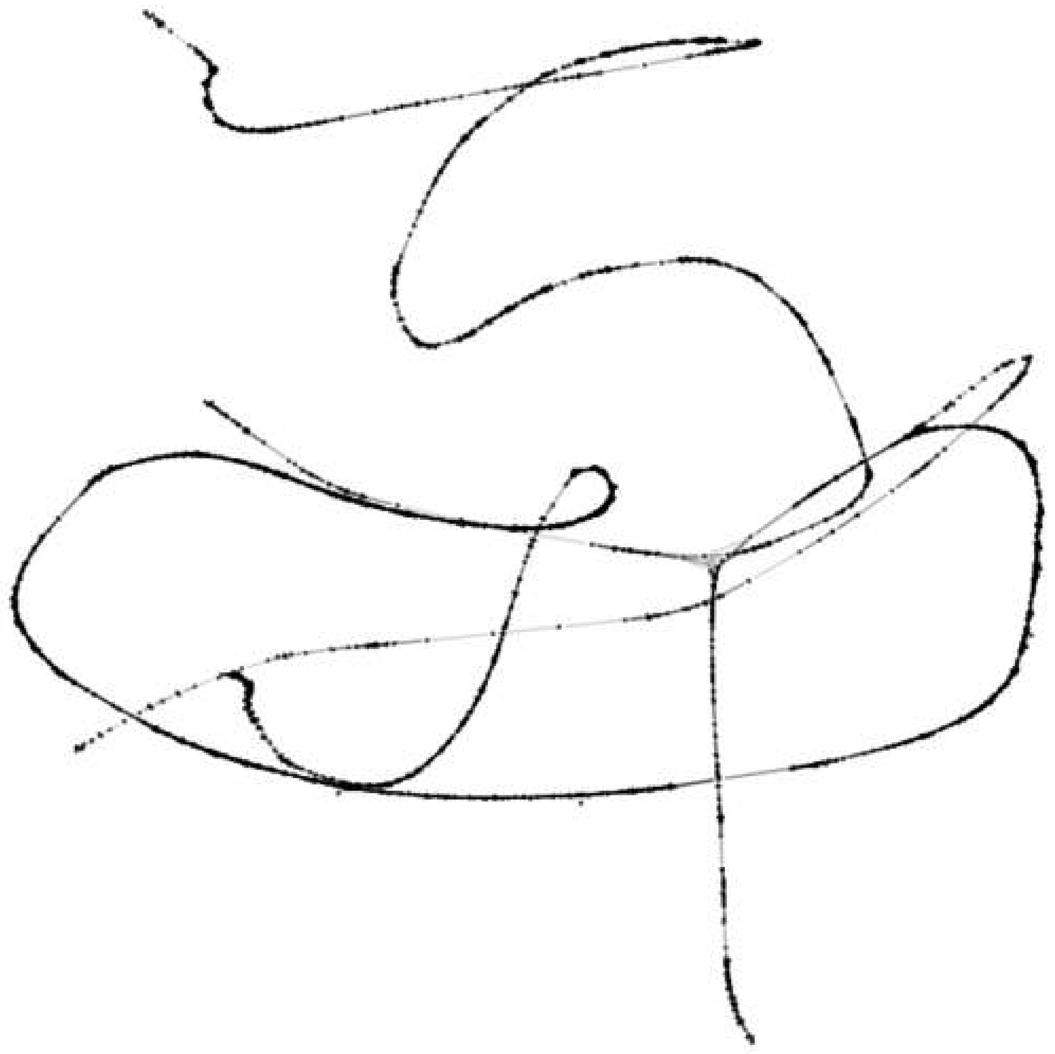

CL249
LTR_Gypsy
Length of Reads (GP):2388 (0.03%)

Tgrandiflorum

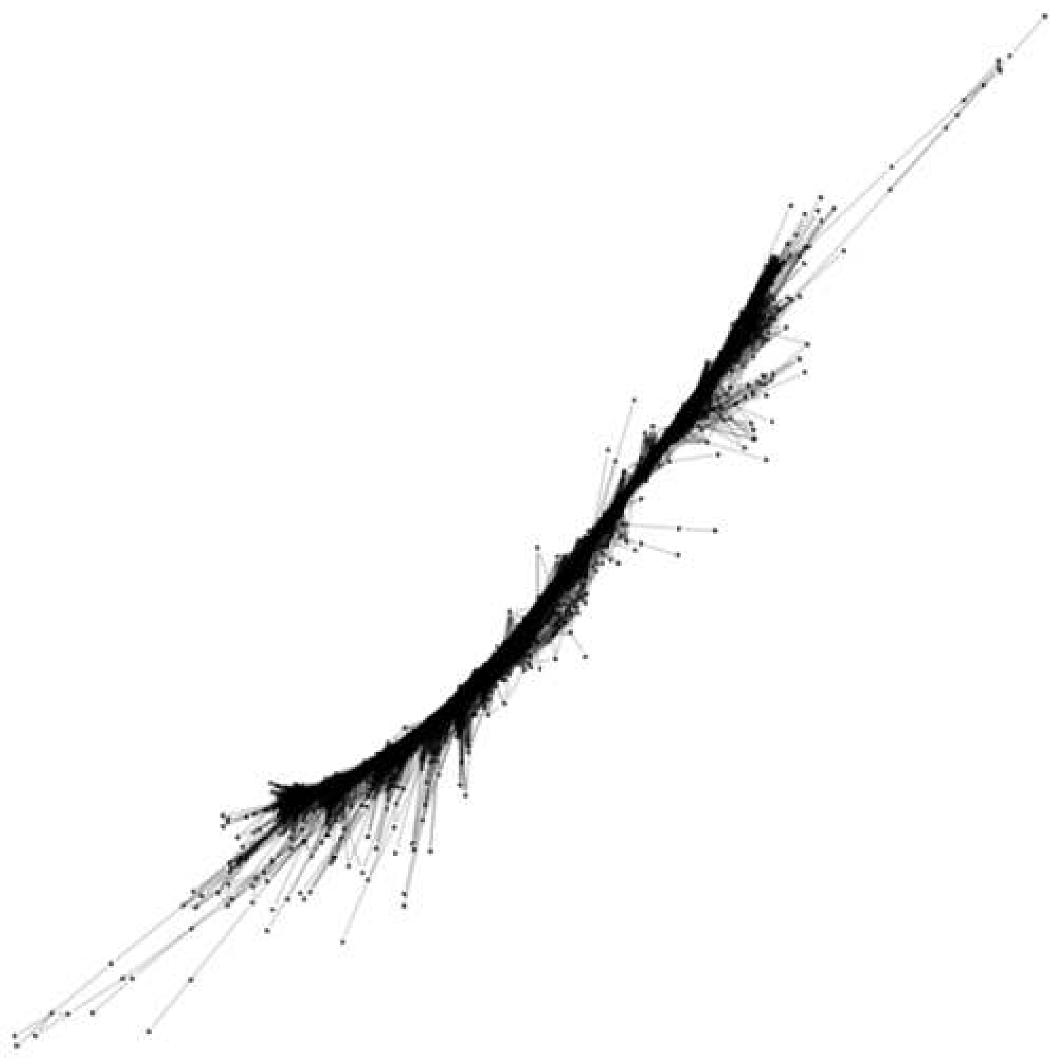

CL250
LTR_Copia
Length of Reads (GP):2283 (0.03%)

Tgrandiflorum

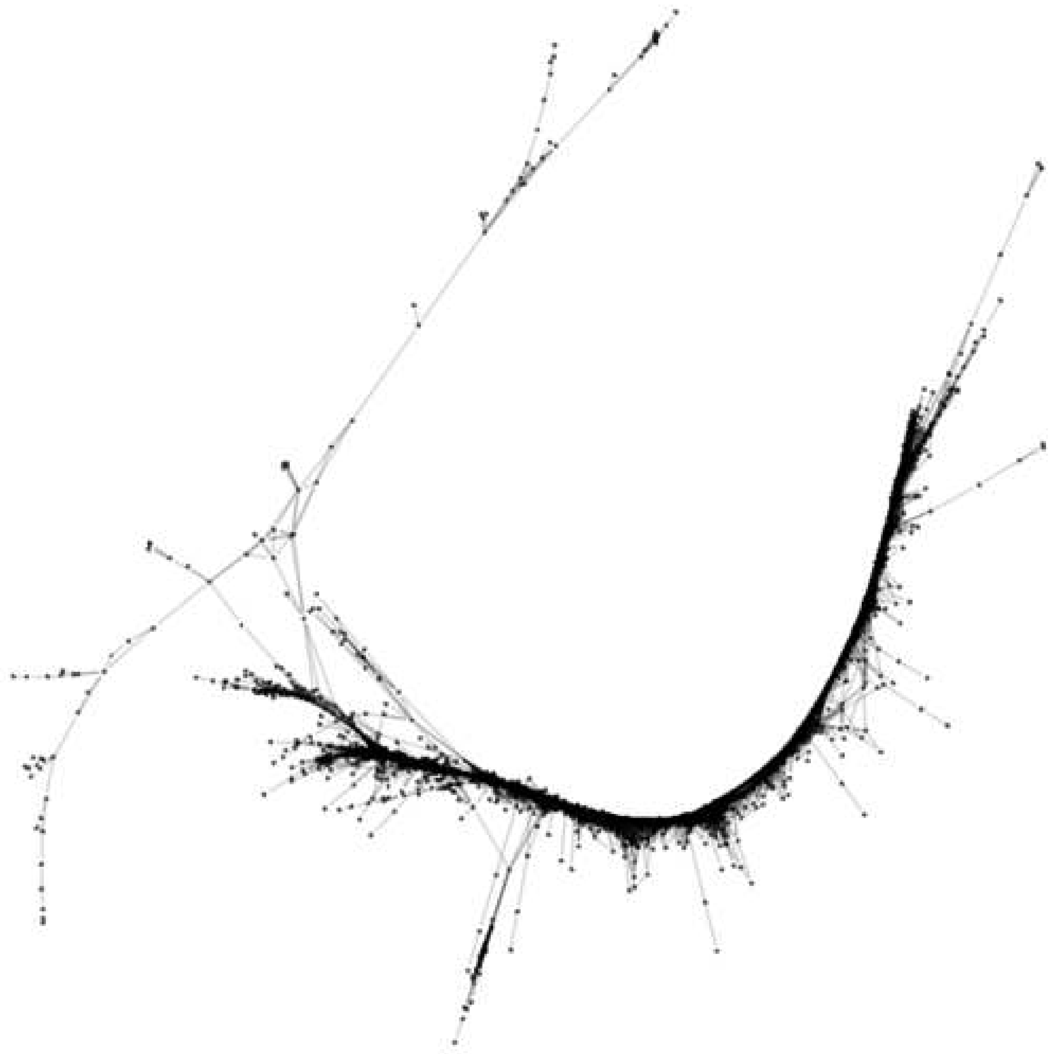

CL251
LTR_Copia
Length of Reads (GP):2241 (0.03%)

Tgrandiflorum

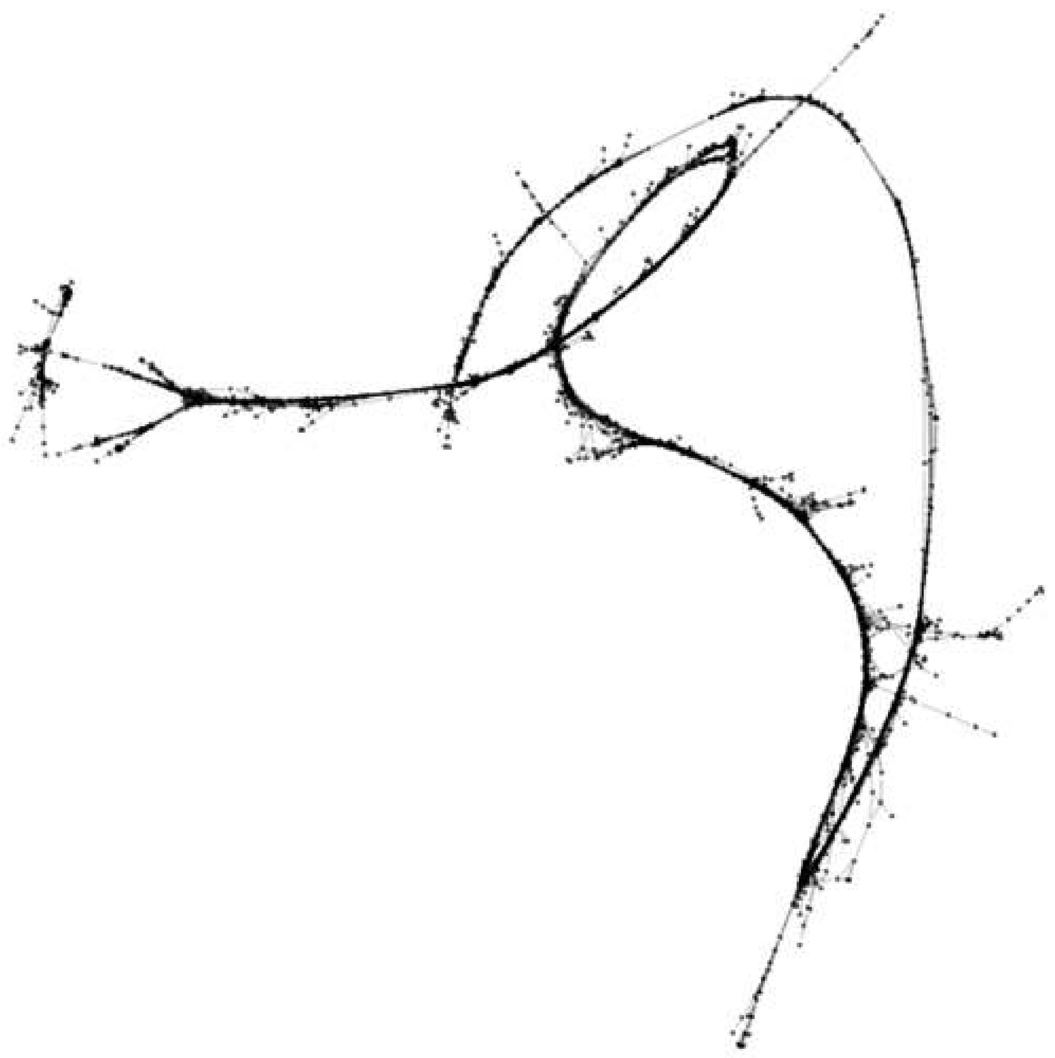

CL252
DNA_PIF_Harbinger
Length of Reads (GP):2202 (0.03%)

Tgrandiflorum

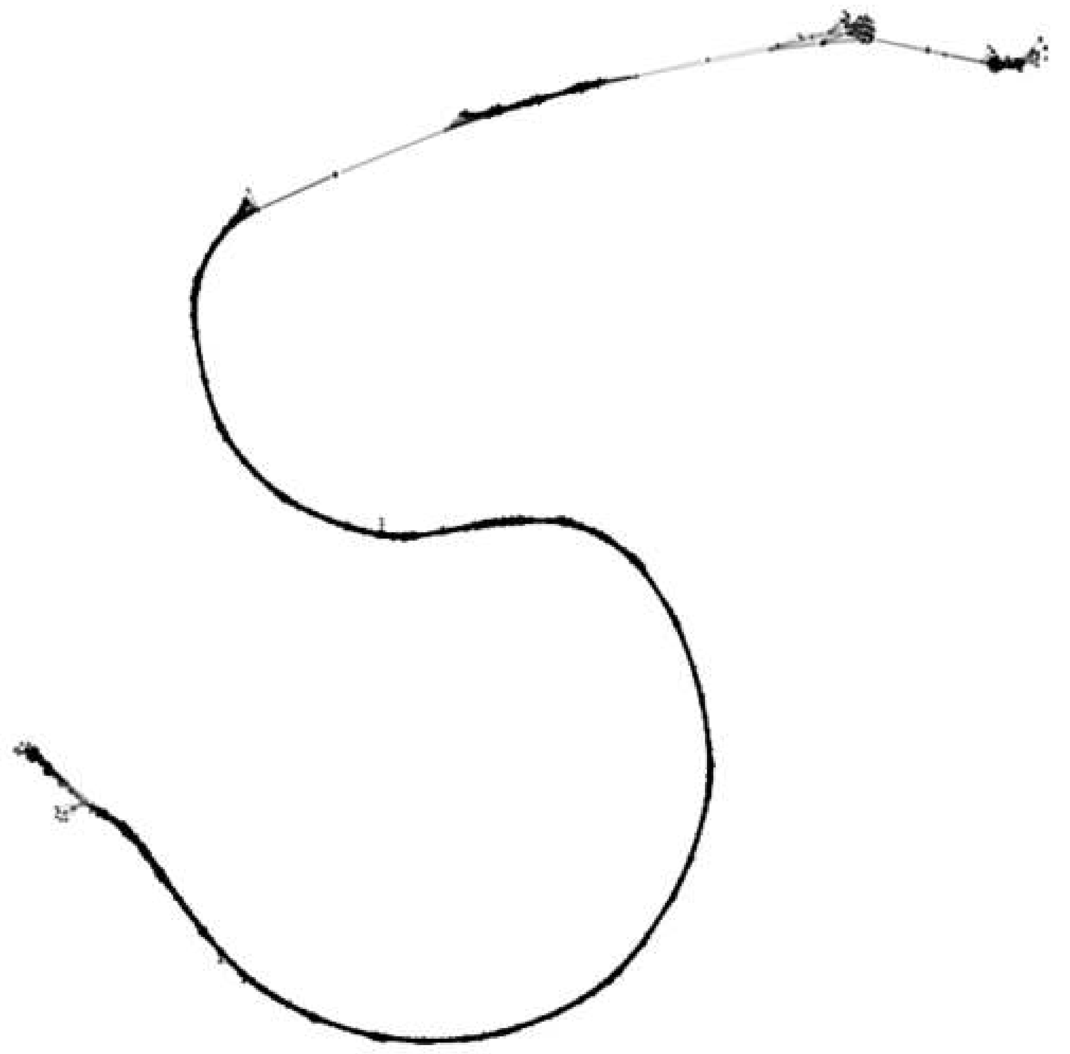

CL253
Low_complexity
Length of Reads (GP):2182 (0.03%)

Tgrandiflorum

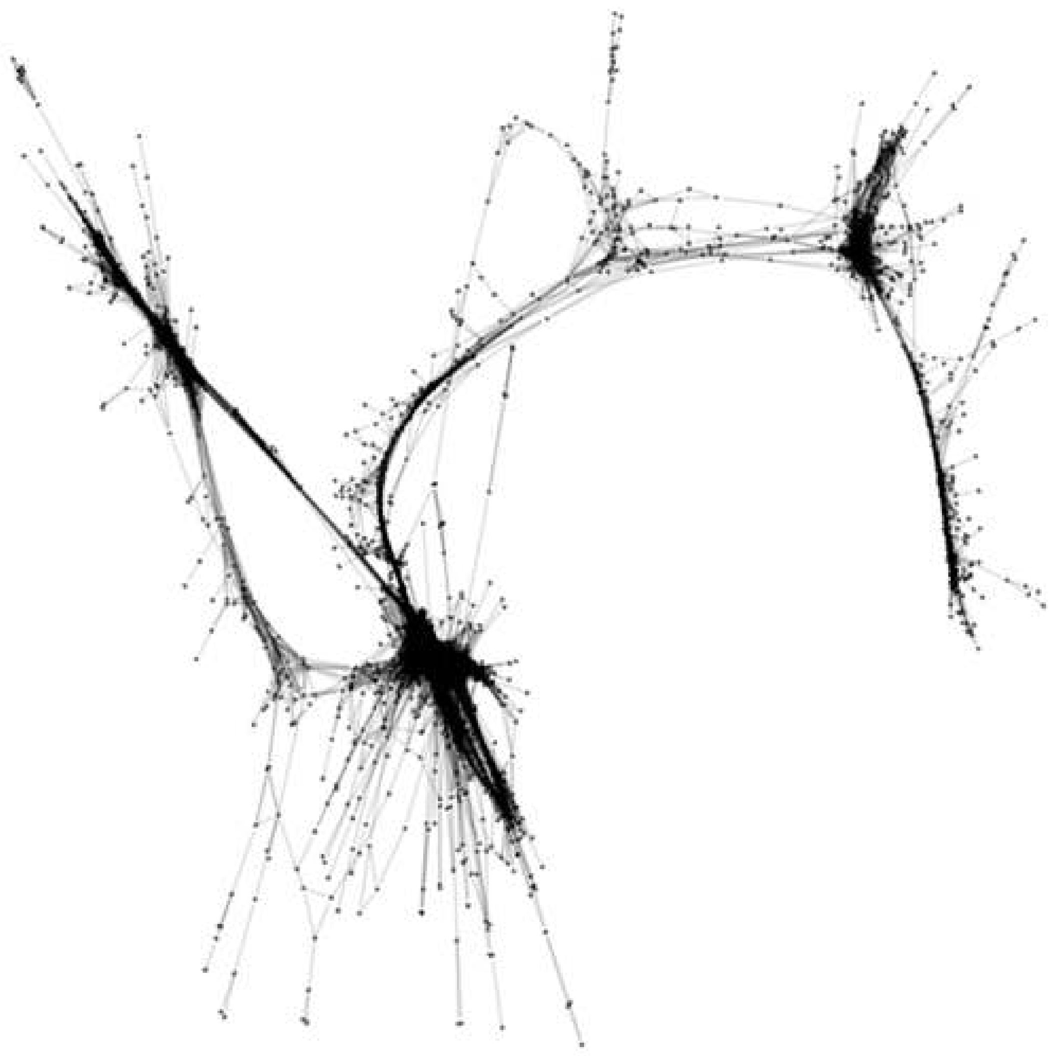

CL254
Low_complexity
Length of Reads (GP):2165 (0.03%)

Tgrandiflorum

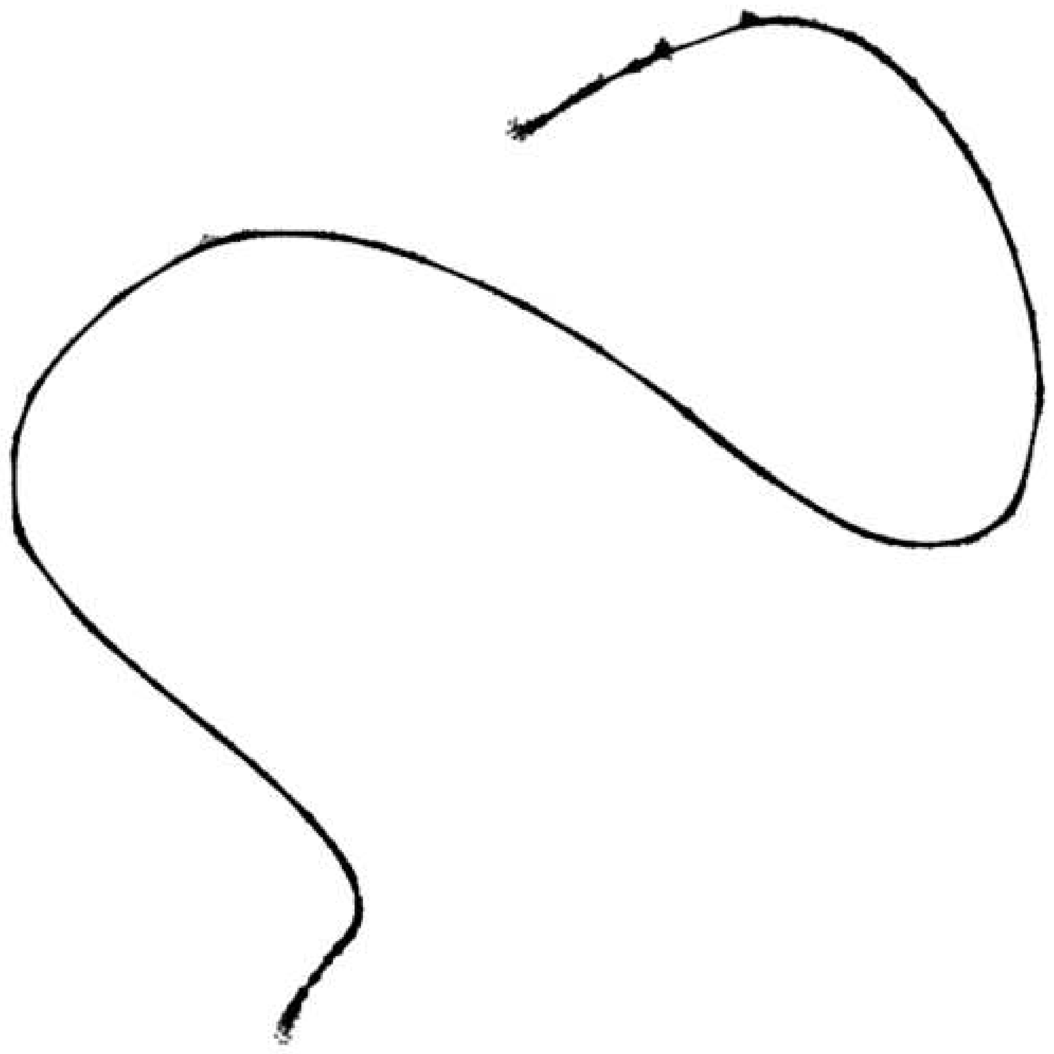

CL255
Low_complexity
Length of Reads (GP):2112 (0.03%)

Tgrandiflorum

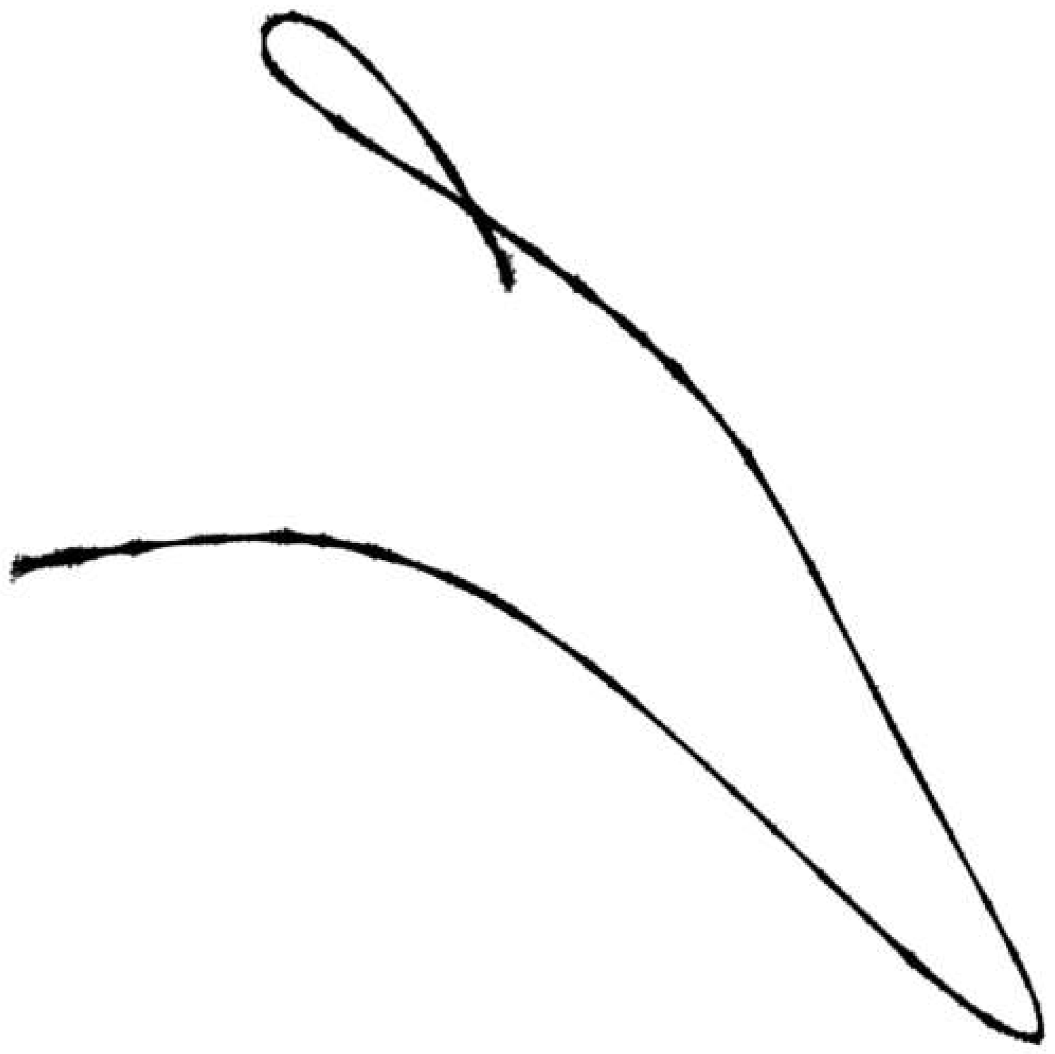

CL256
Low_complexity
Length of Reads (GP):2074 (0.03%)

Tgrandiflorum

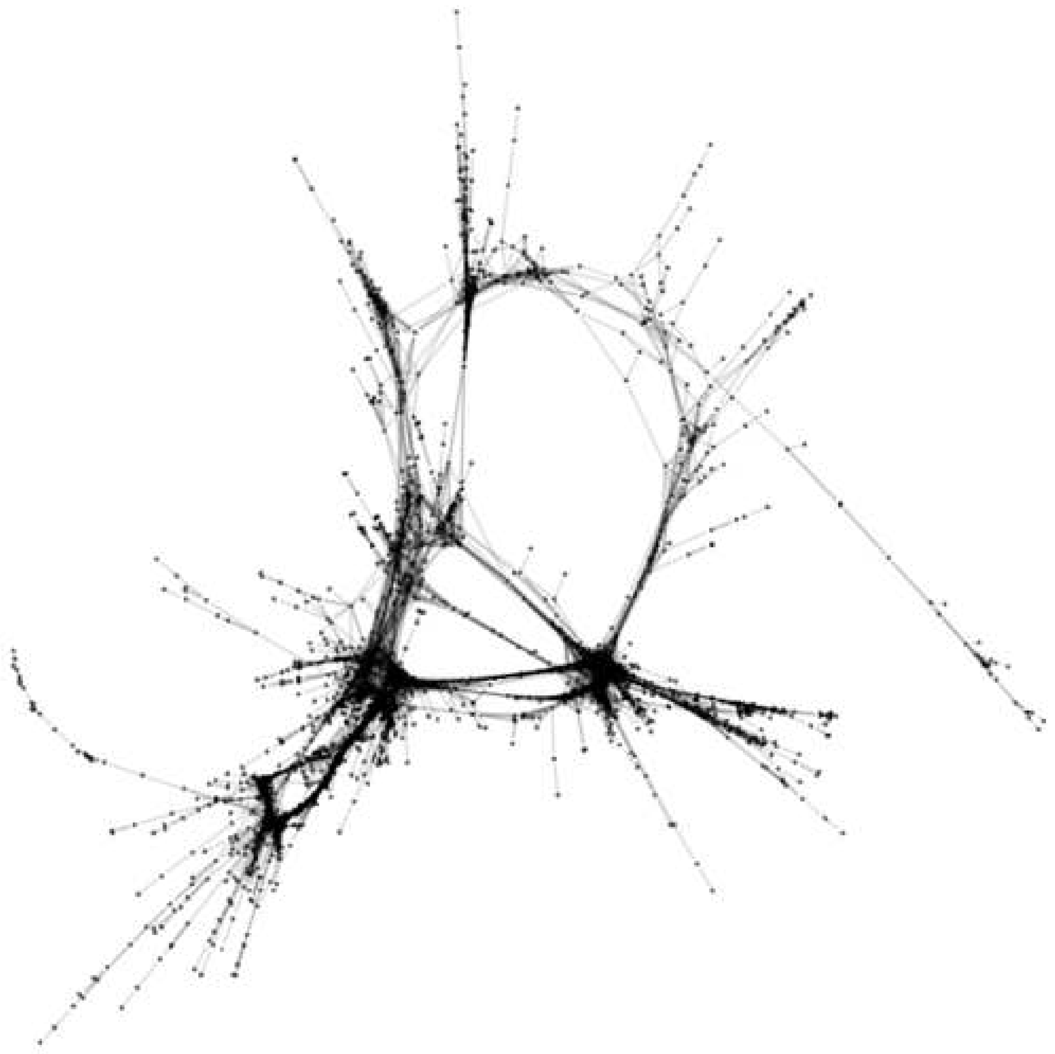

CL257
Low_complexity
Length of Reads (GP):2074 (0.03%)

Tgrandiflorum

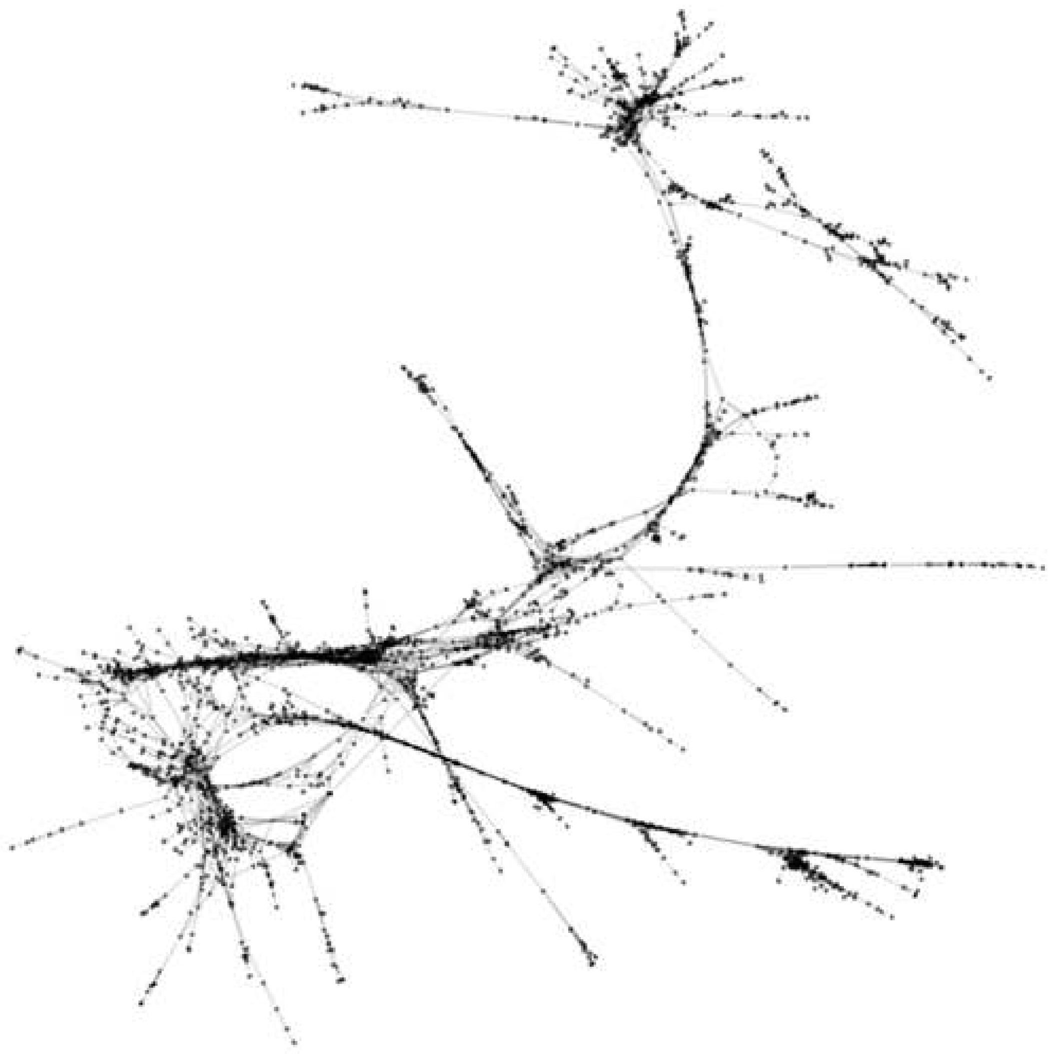

CL258
Low_complexity
Length of Reads (GP):2065 (0.03%)

Tgrandiflorum

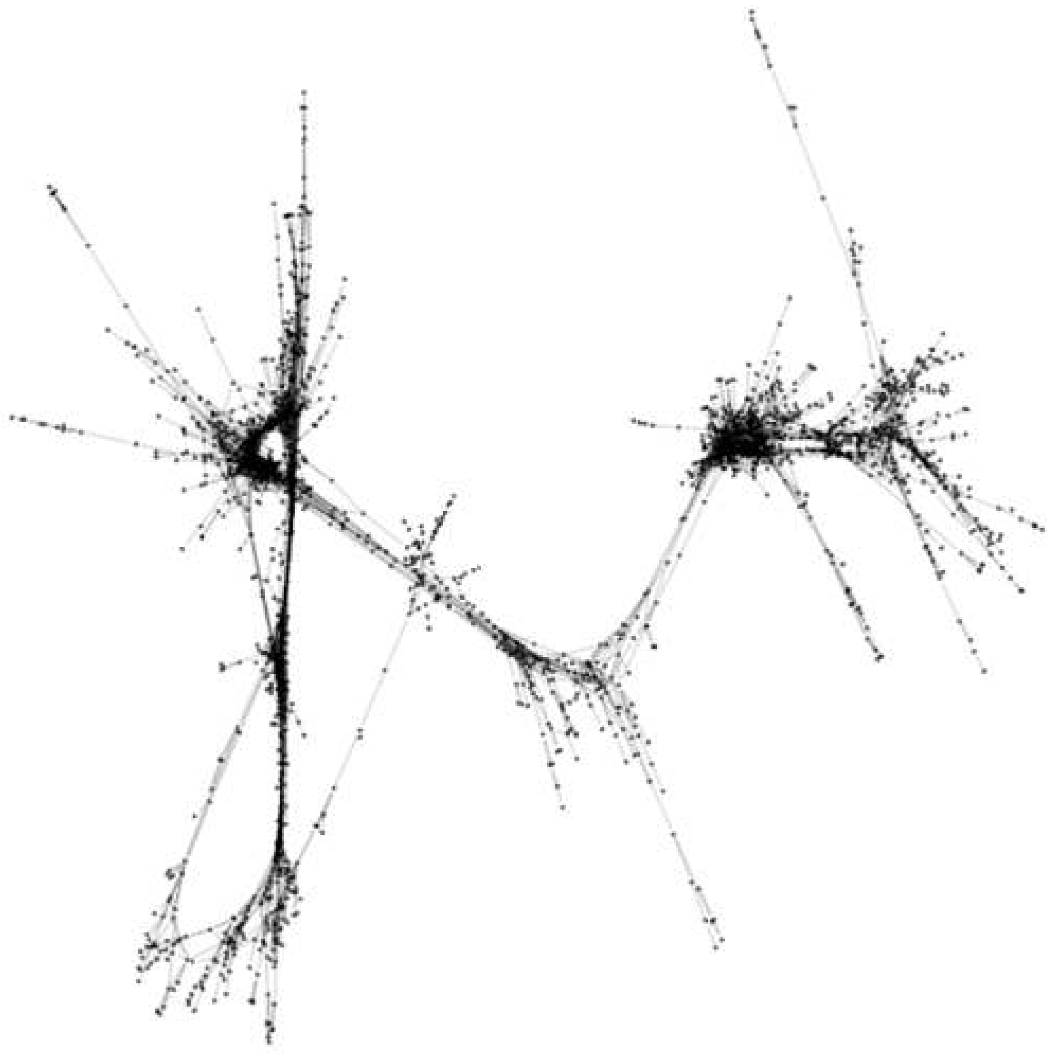

CL259
Low_complexity
Length of Reads (GP):2050 (0.03%)

Tgrandiflorum

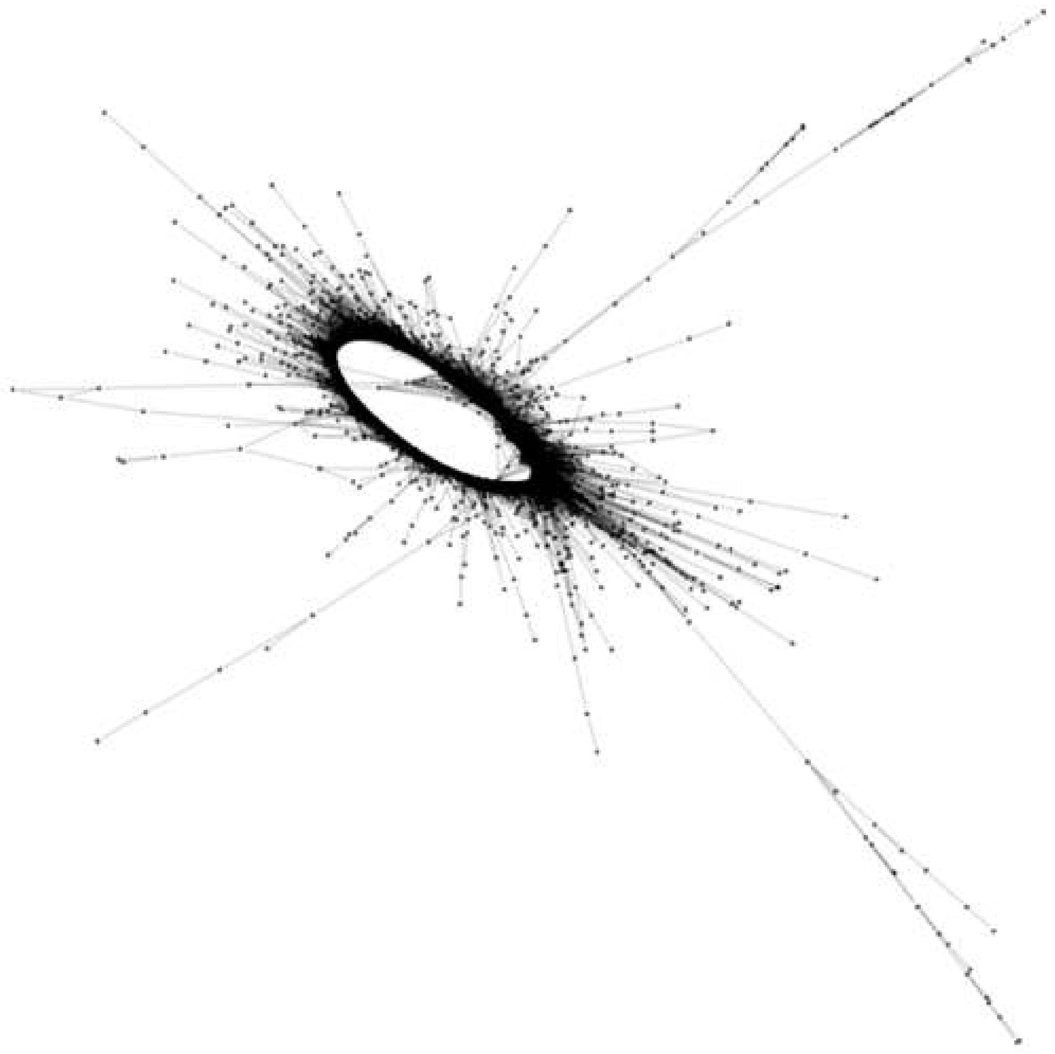

CL260
Low_complexity
Length of Reads (GP):2031 (0.03%)

Tgrandiflorum

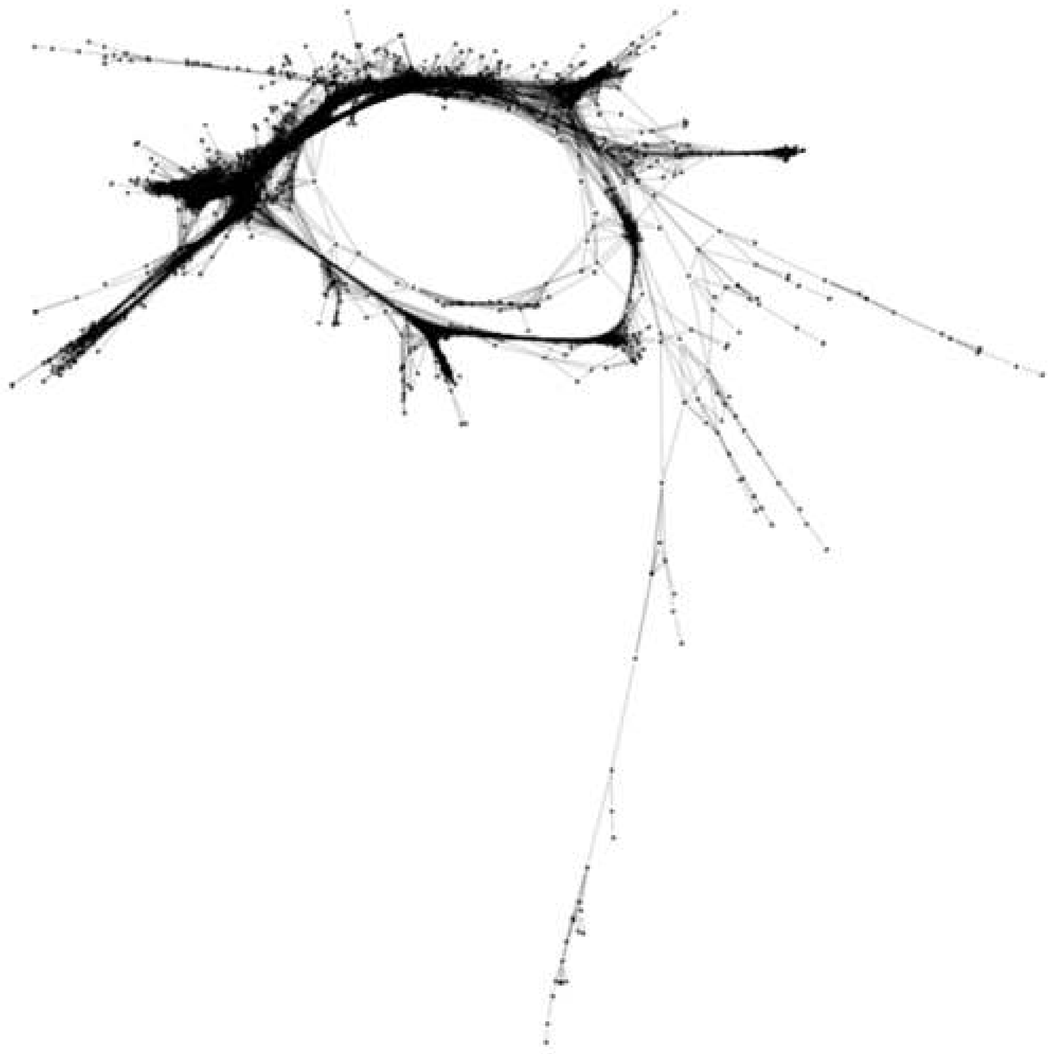

CL261
Low_complexity
Length of Reads (GP):2019 (0.03%)

Tgrandiflorum

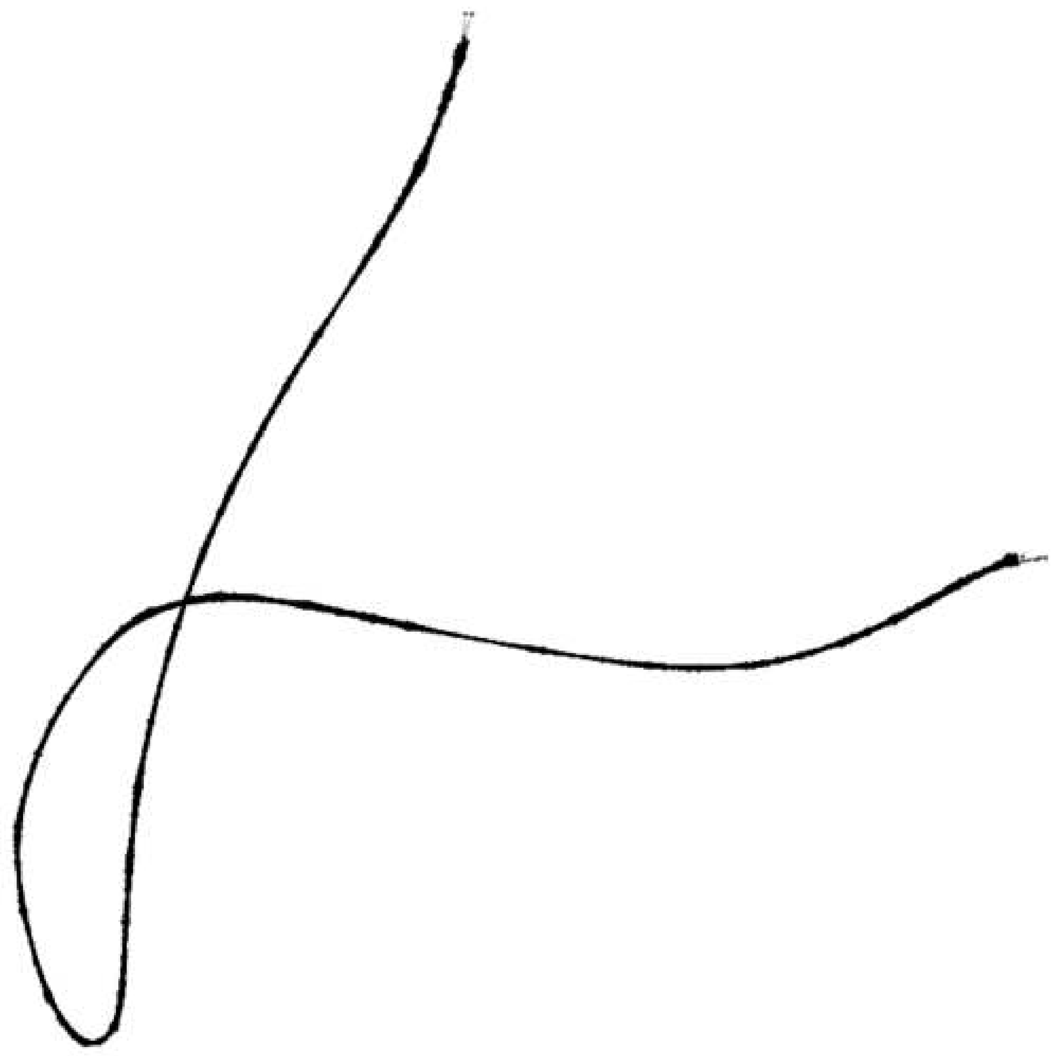

CL262
Low_complexity
Length of Reads (GP):2013 (0.03%)

Tgrandiflorum

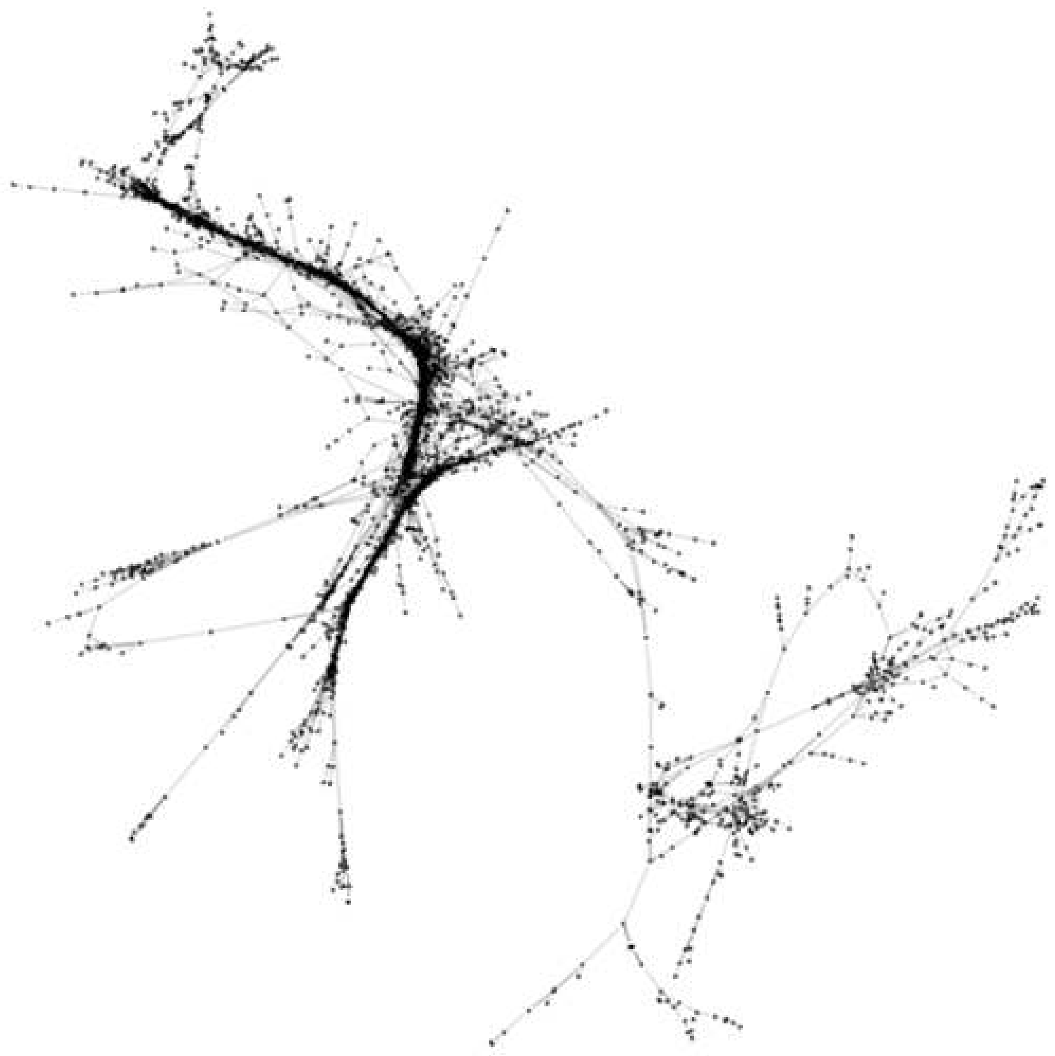

CL263
Low_complexity
Length of Reads (GP):1961 (0.02%)

Tgrandiflorum

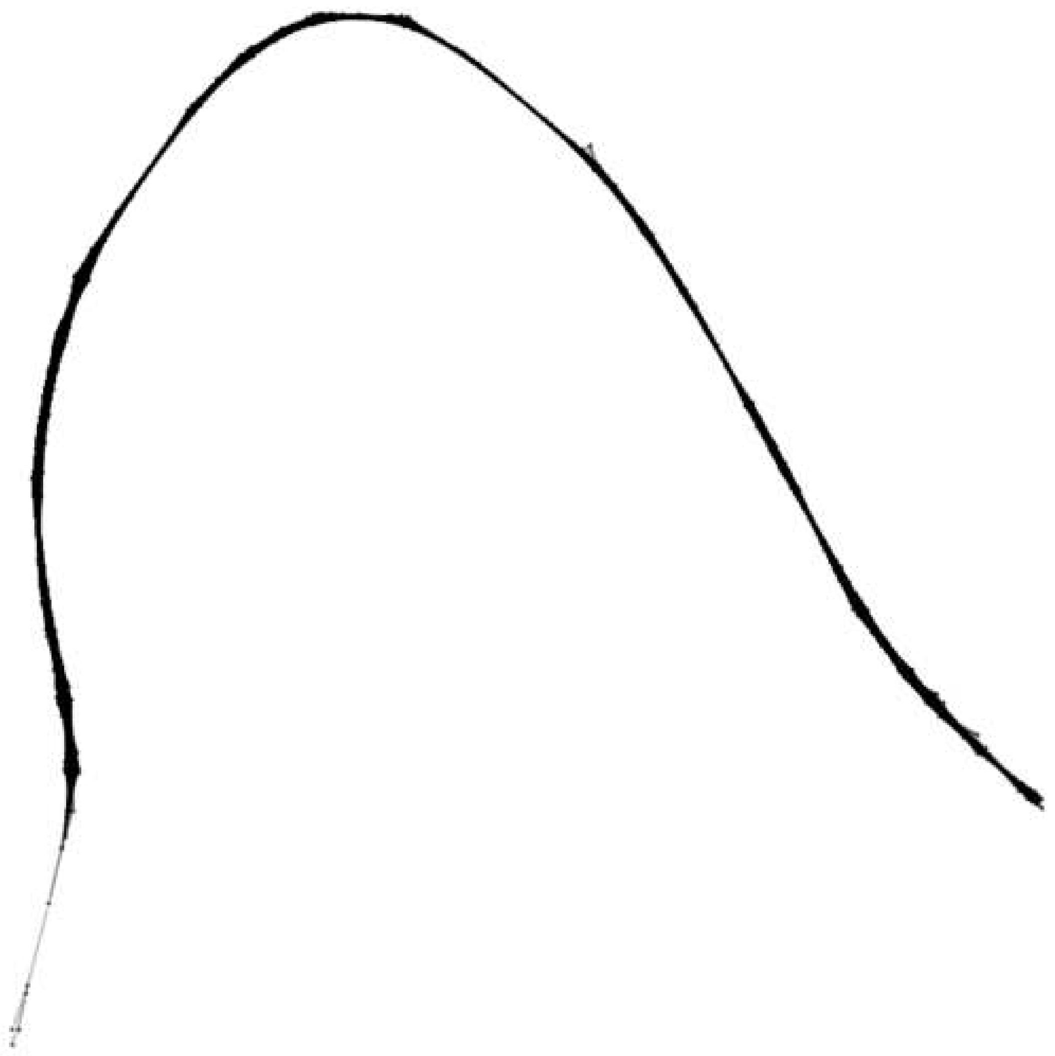

CL264
rRNA
Length of Reads (GP):1930 (0.02%)

Tgrandiflorum

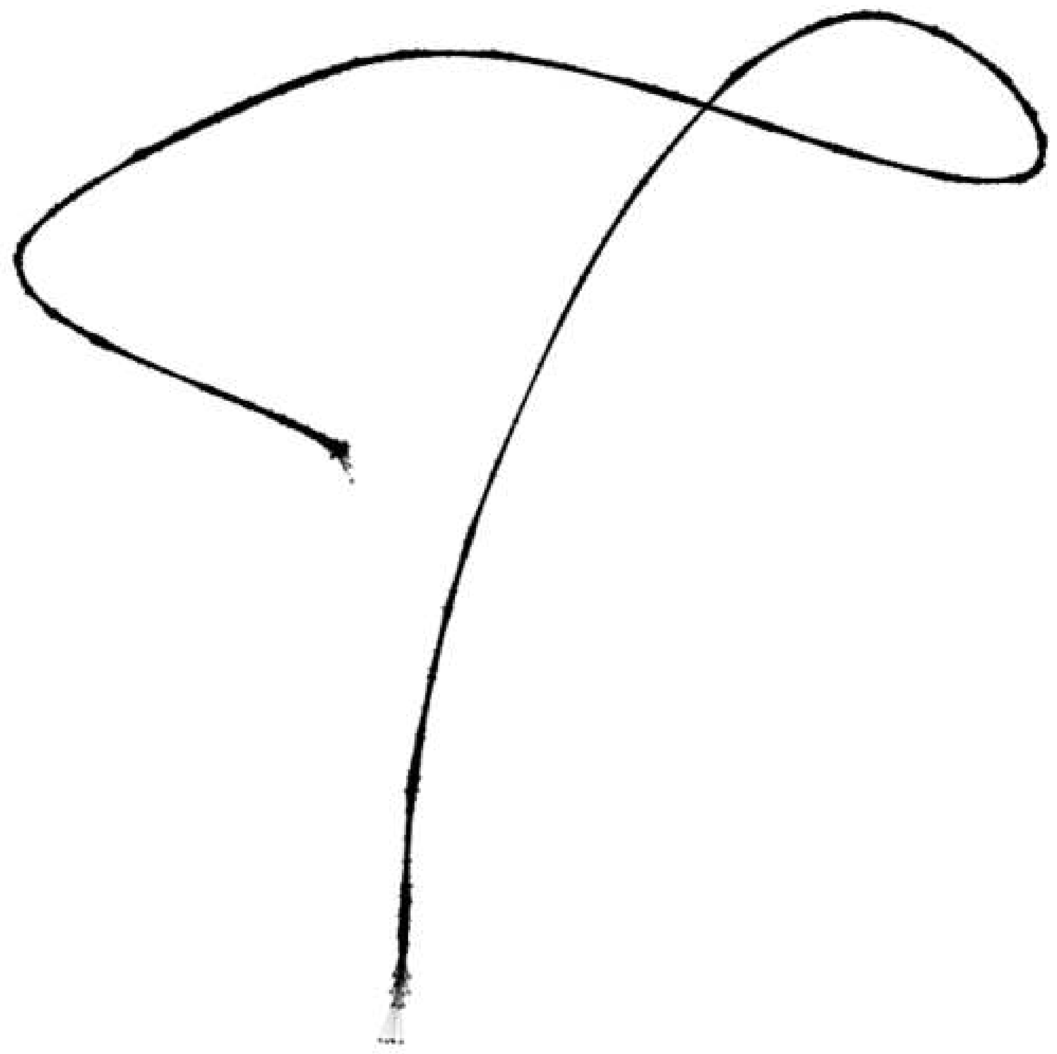

CL265
LTR_Copia
Length of Reads (GP):1923 (0.02%)

Tgrandiflorum

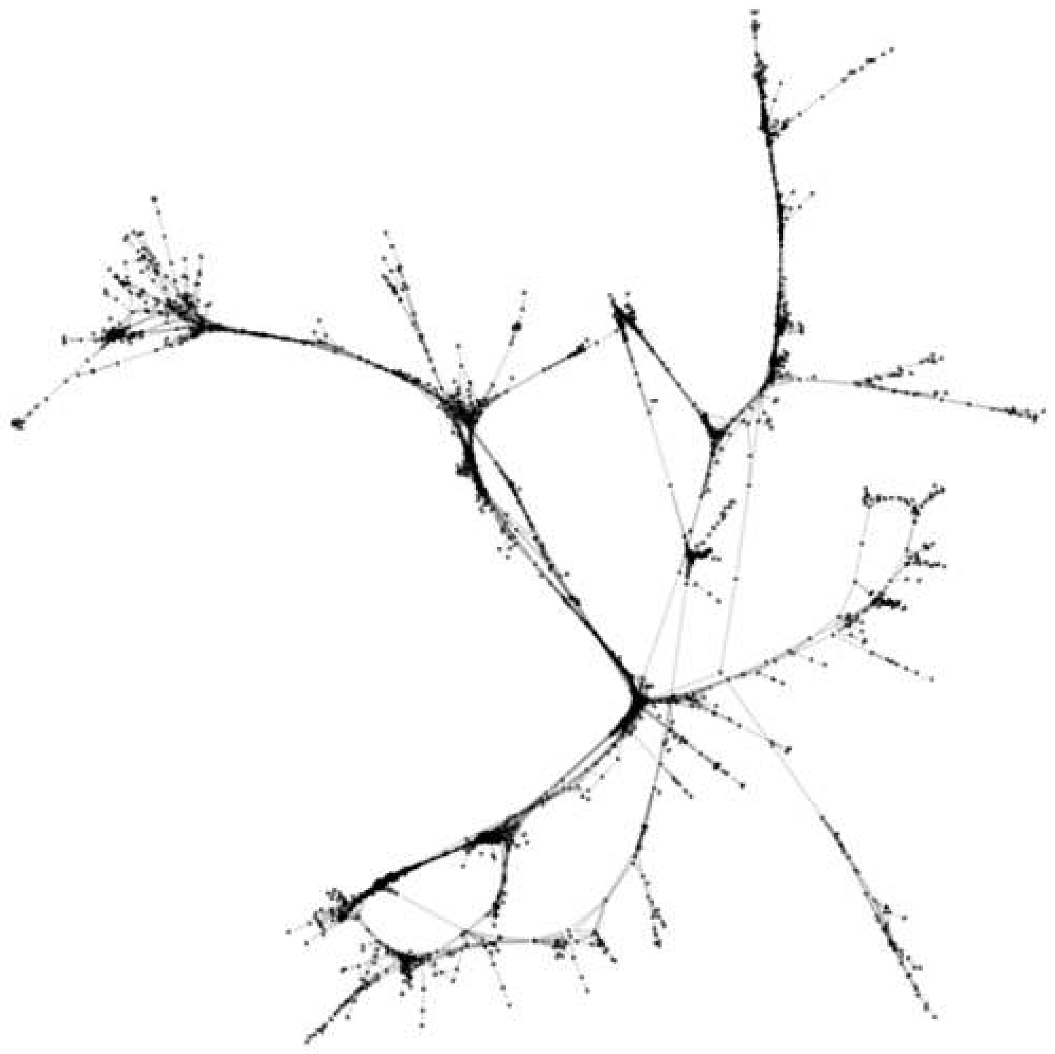

CL266
Low_complexity
Length of Reads (GP):1920 (0.02%)

Tgrandiflorum

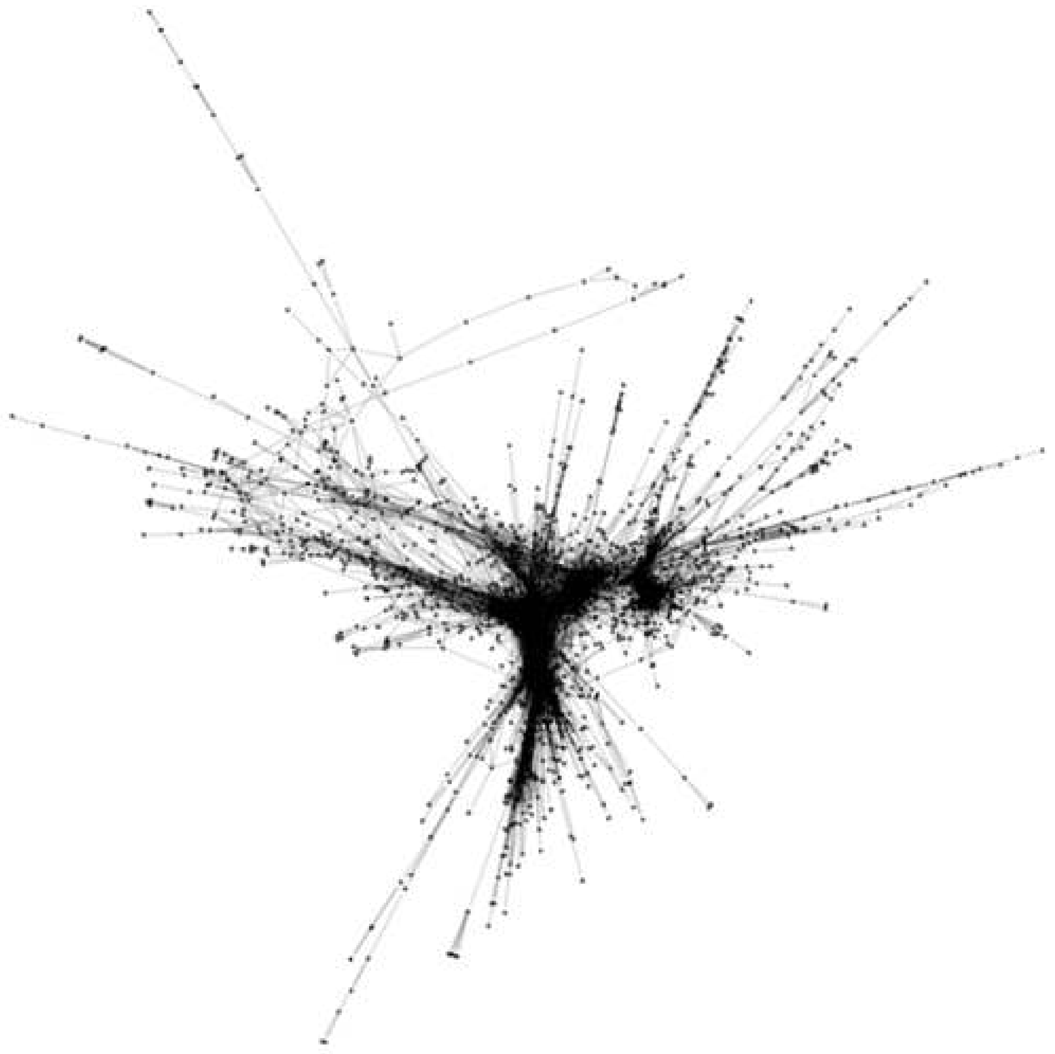

CL267
Low_complexity
Length of Reads (GP):1920 (0.02%)

Tgrandiflorum

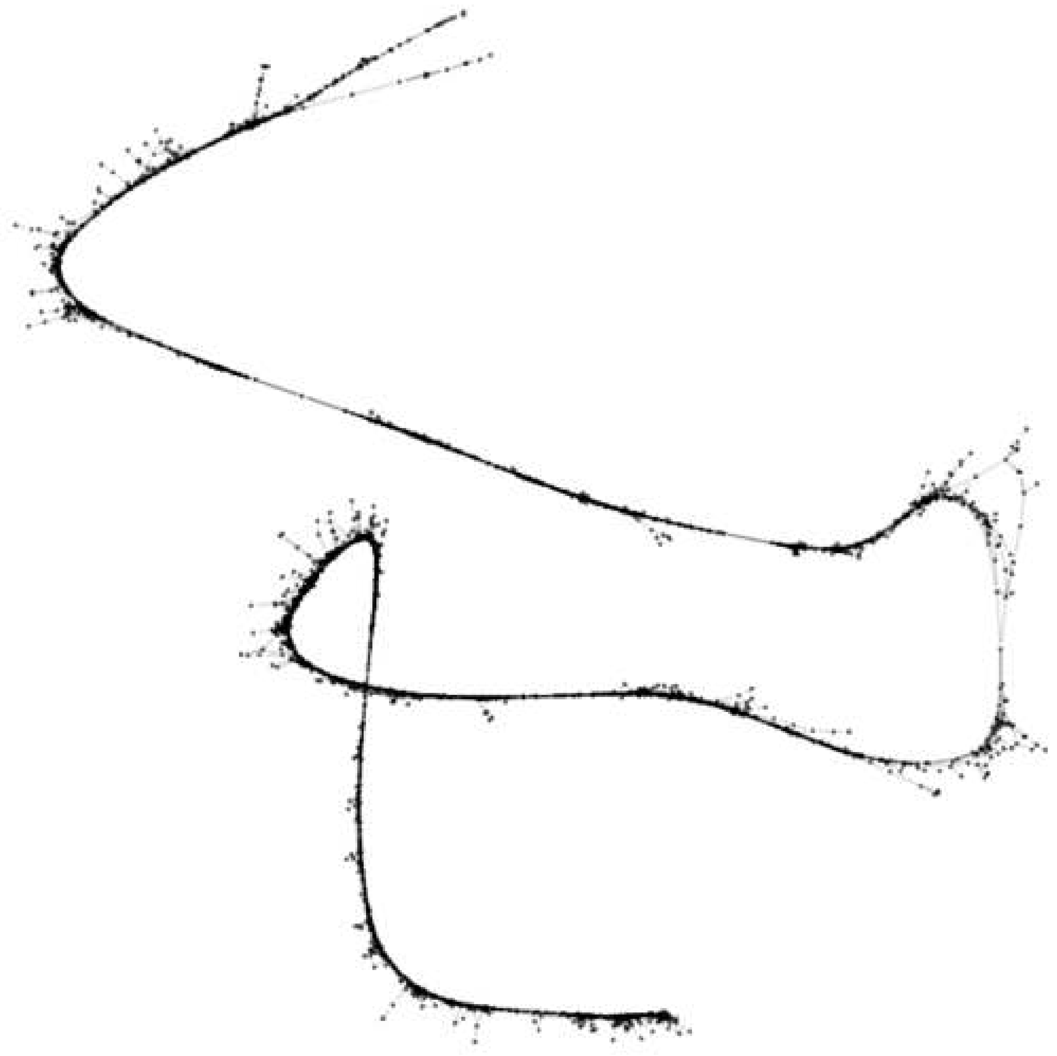

CL268
DNA_PIF_Harbinger
Length of Reads (GP):1876 (0.02%)

Tgrandiflorum

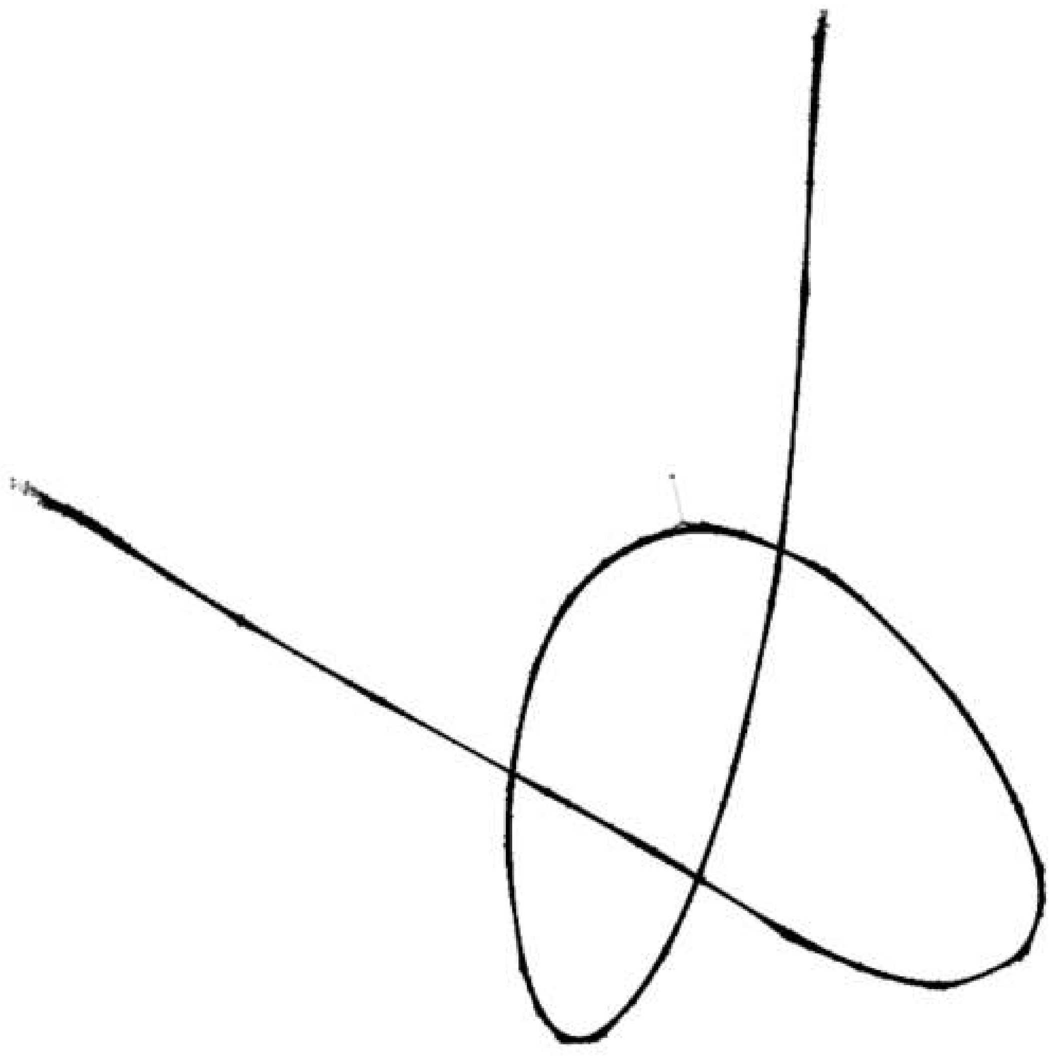

CL269
Low_complexity
Length of Reads (GP):1857 (0.02%)

Tgrandiflorum

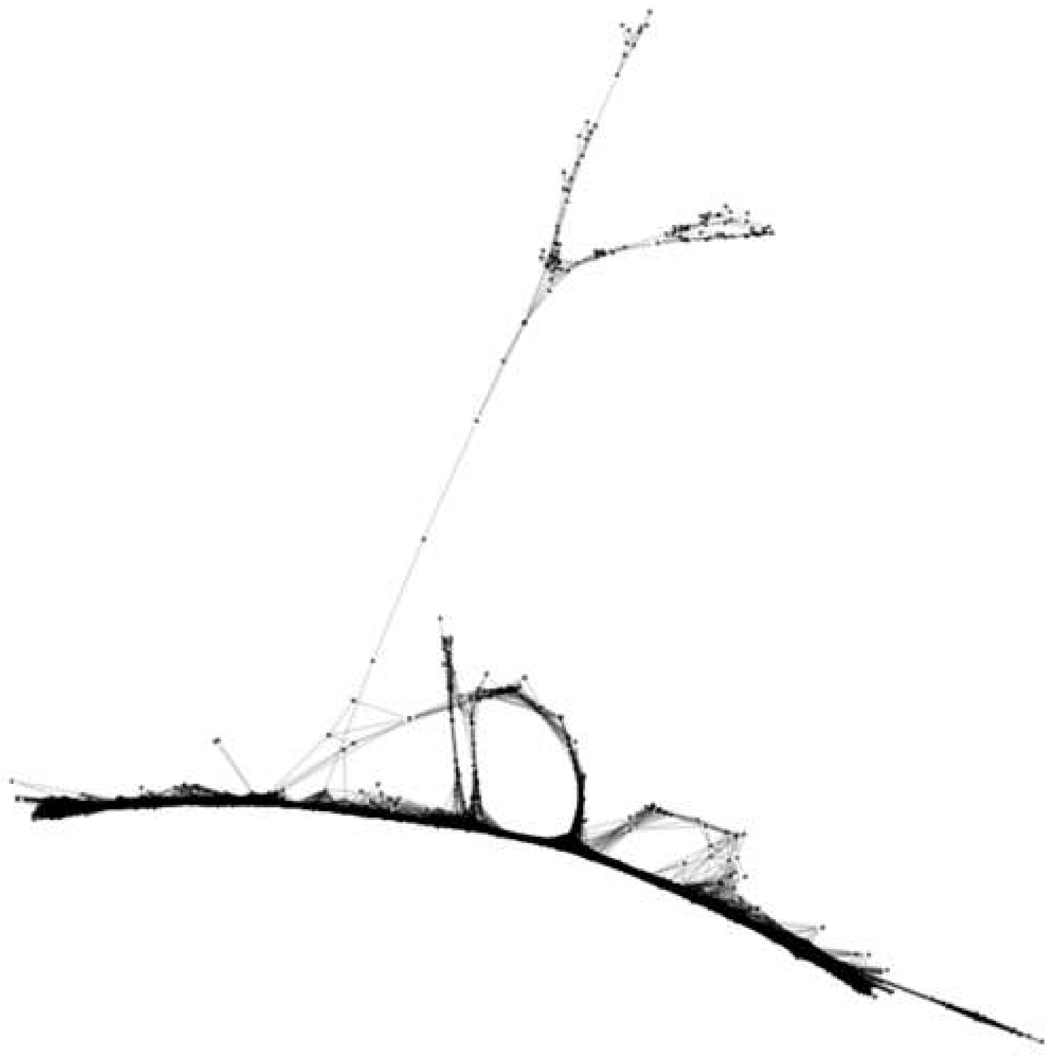

CL270
LTR_Copia
Length of Reads (GP):1809 (0.02%)

Tgrandiflorum

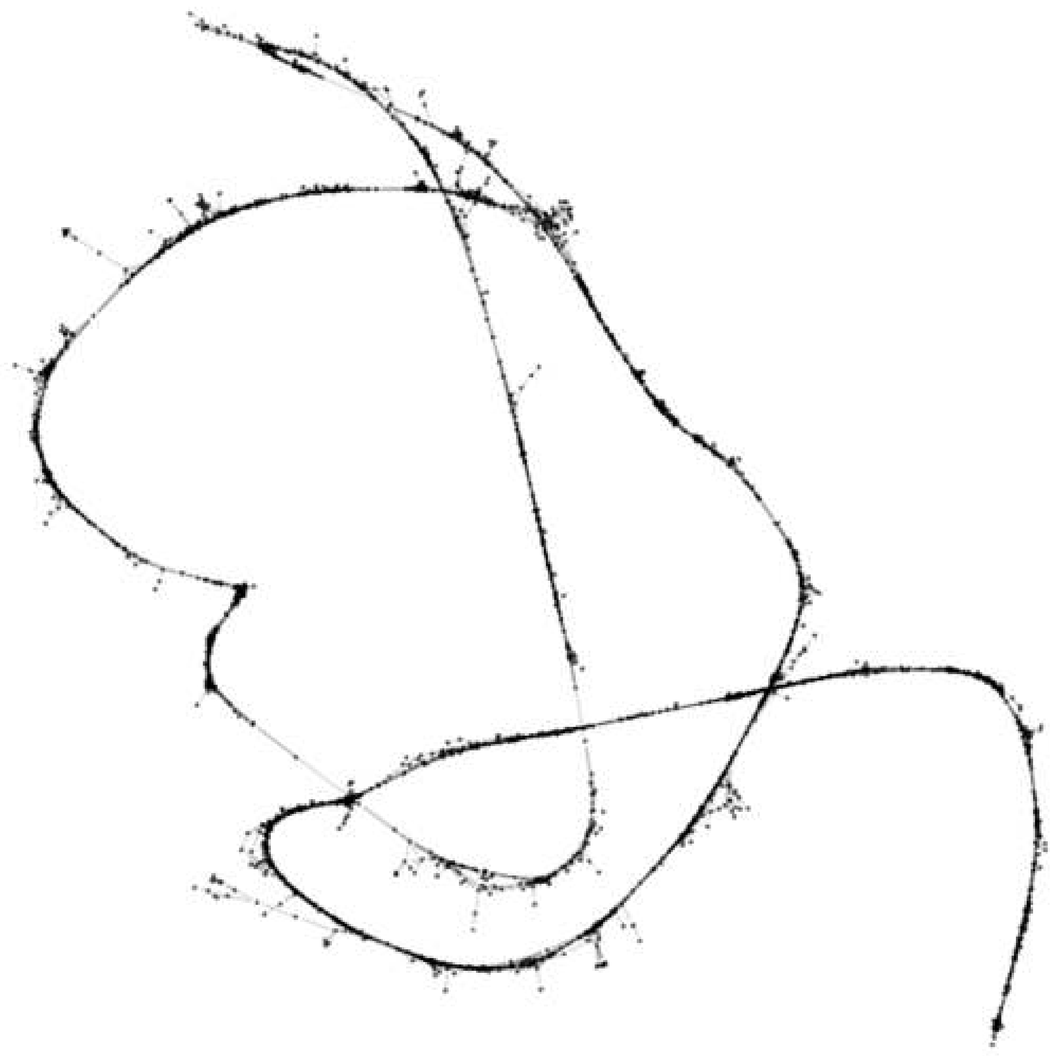

CL271
DNA_PIF_Harbinger
Length of Reads (GP):1796 (0.02%)

Tgrandiflorum

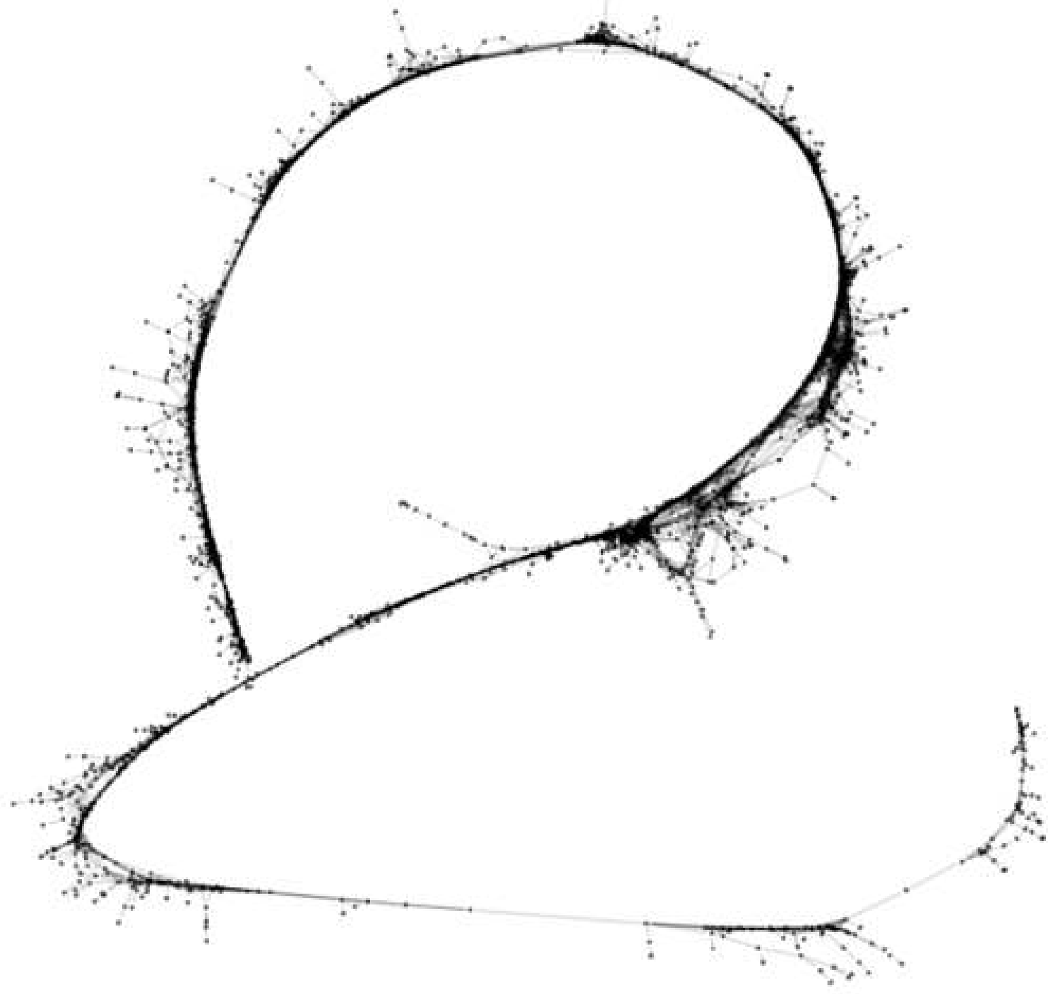

CL272
Low_complexity
Length of Reads (GP):1784 (0.02%)

Tgrandiflorum

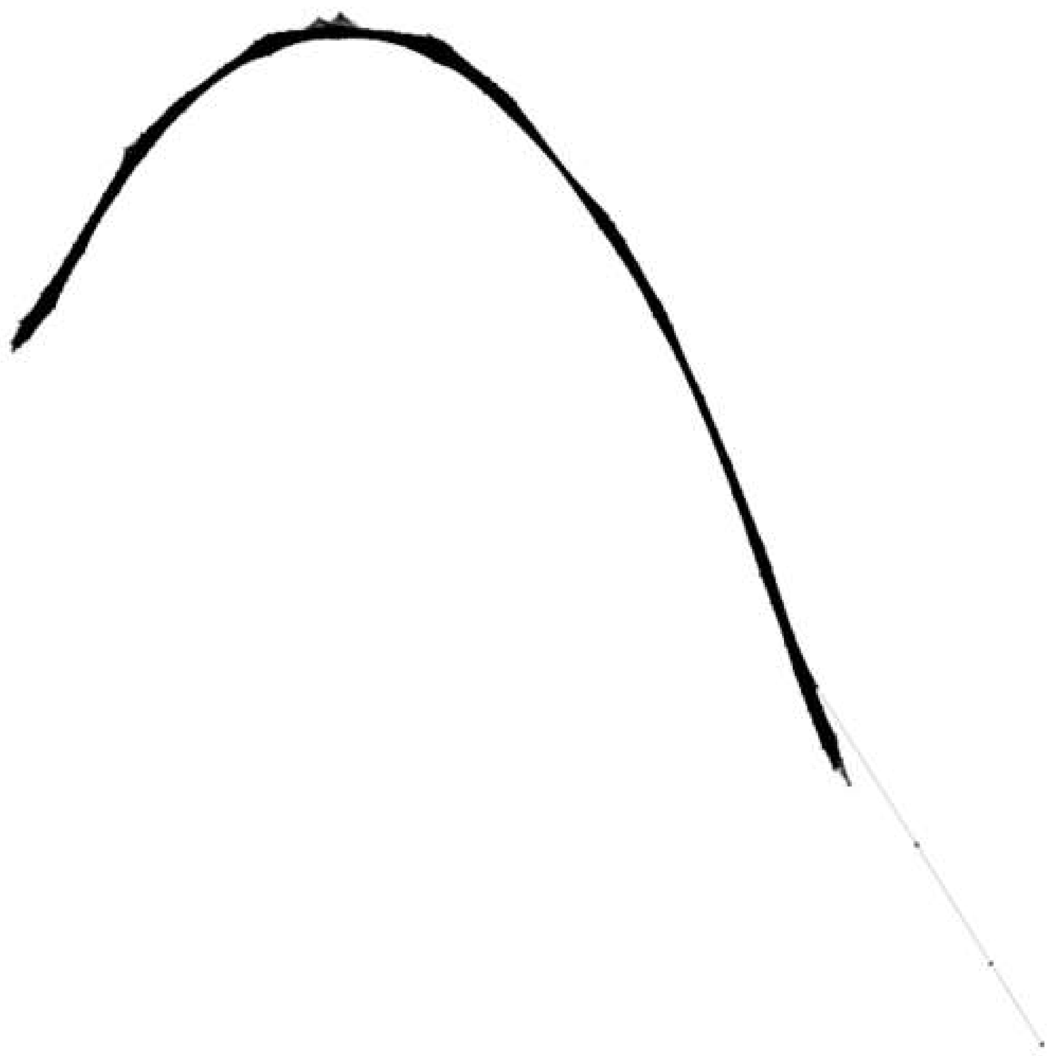

CL273
rRNA
Length of Reads (GP):1783 (0.02%)

Tgrandiflorum

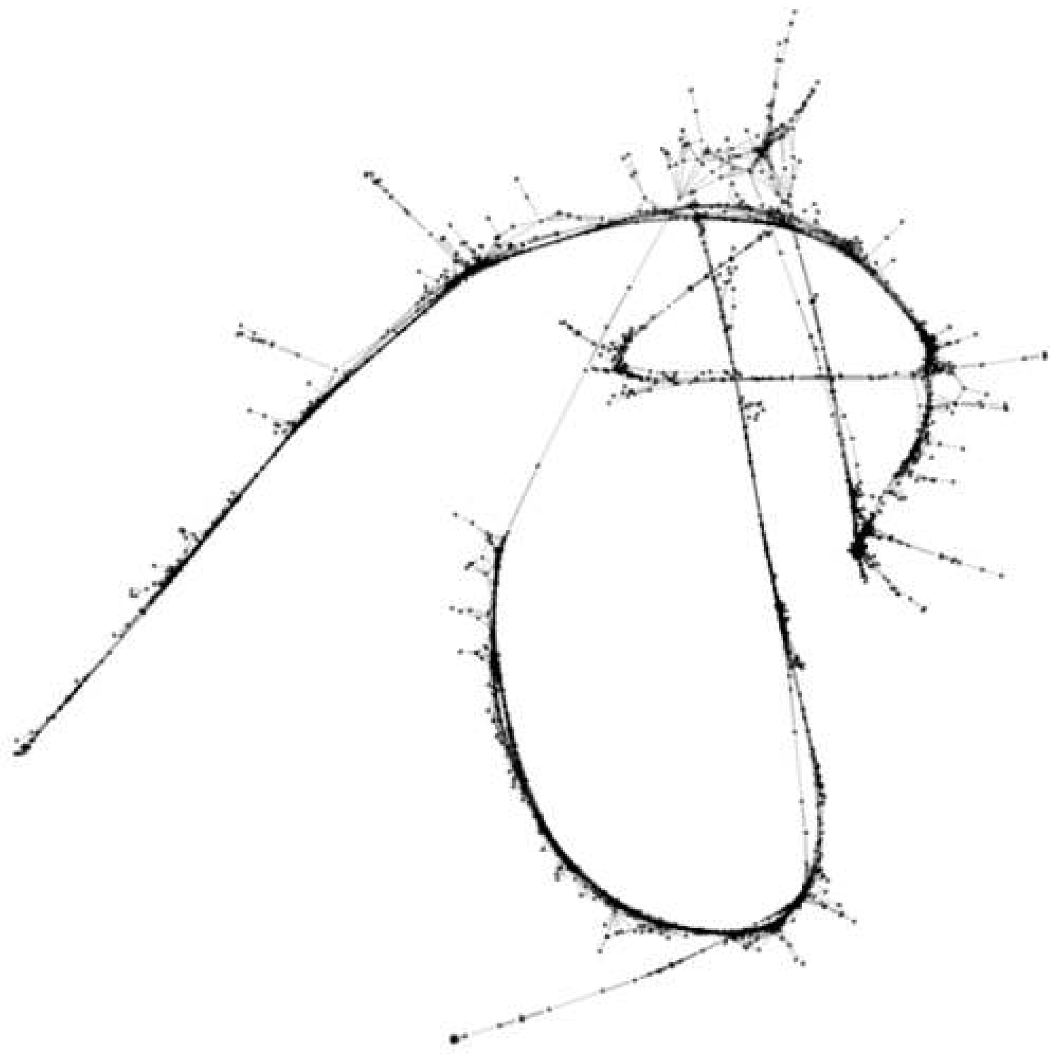

CL274
Low_complexity
Length of Reads (GP):1762 (0.02%)

Tgrandiflorum

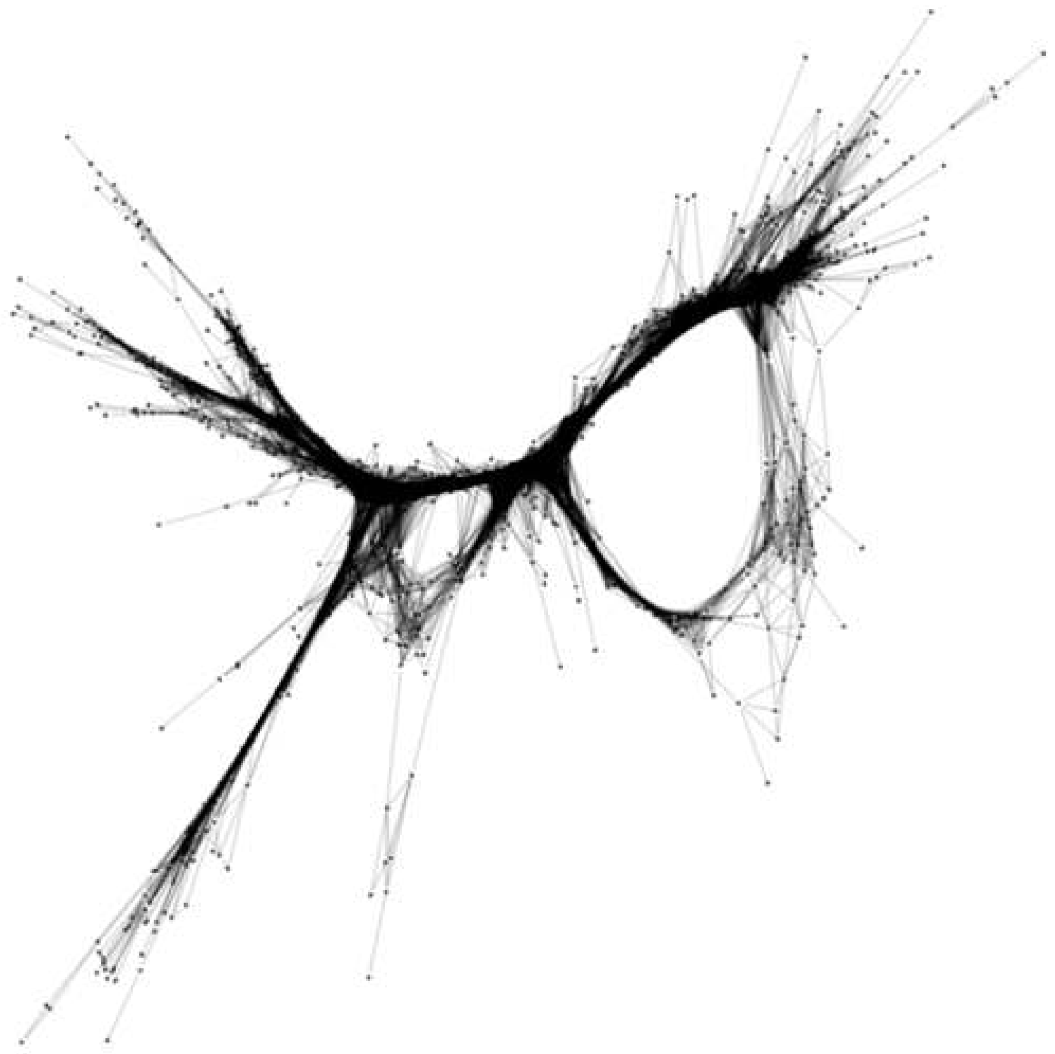

CL275
LTR_Gypsy
Length of Reads (GP):1761 (0.02%)

Tgrandiflorum

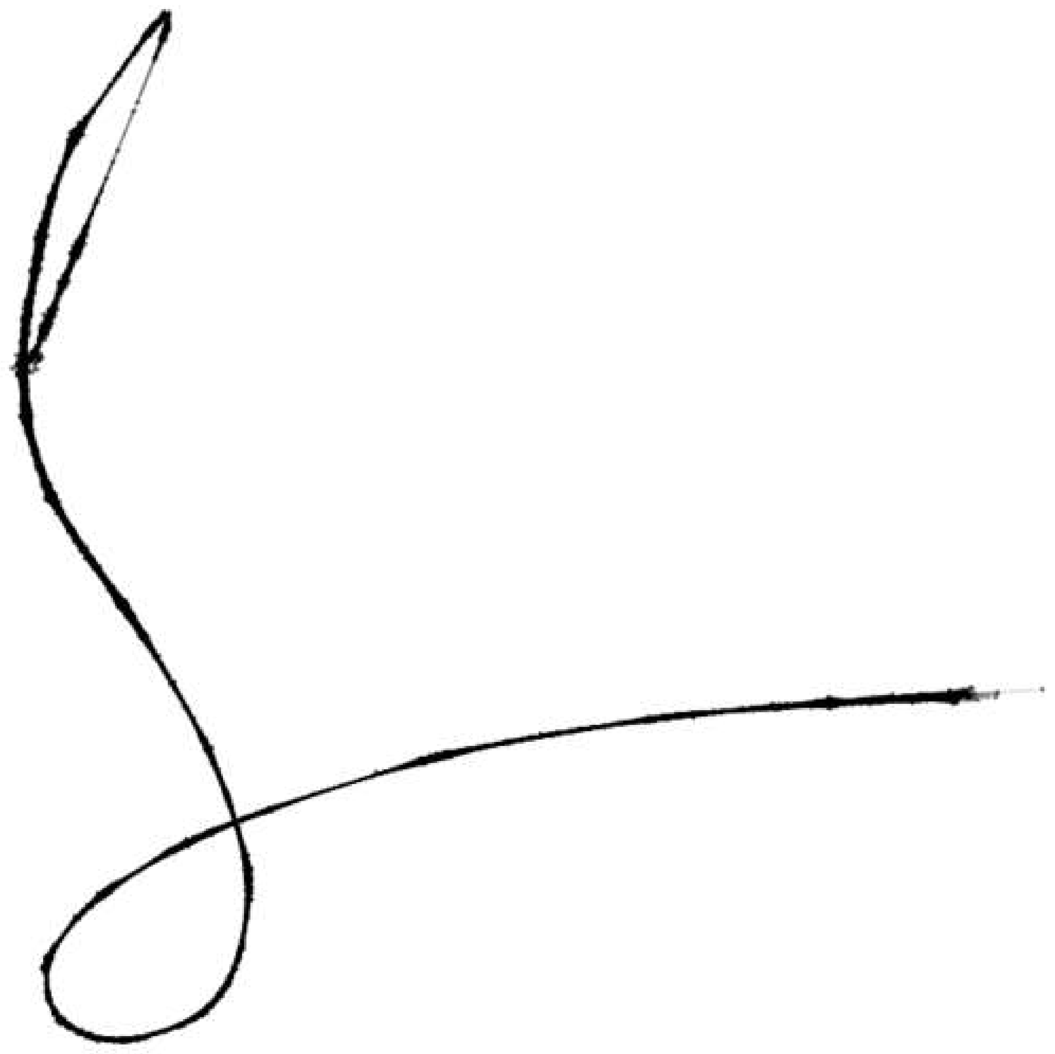

CL276
Low_complexity
Length of Reads (GP):1686 (0.02%)

Tgrandiflorum

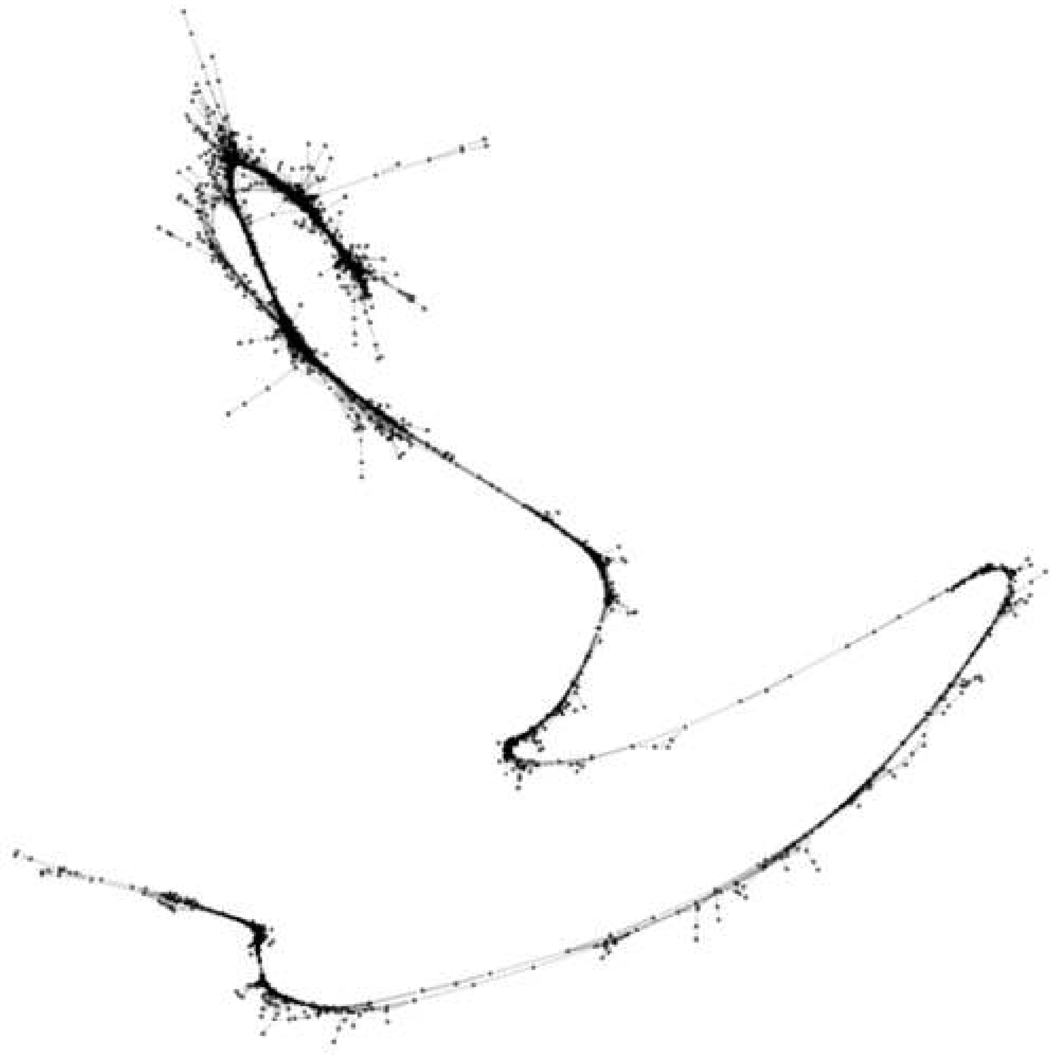

CL277
Low_complexity
Length of Reads (GP):1625 (0.02%)

Tgrandiflorum

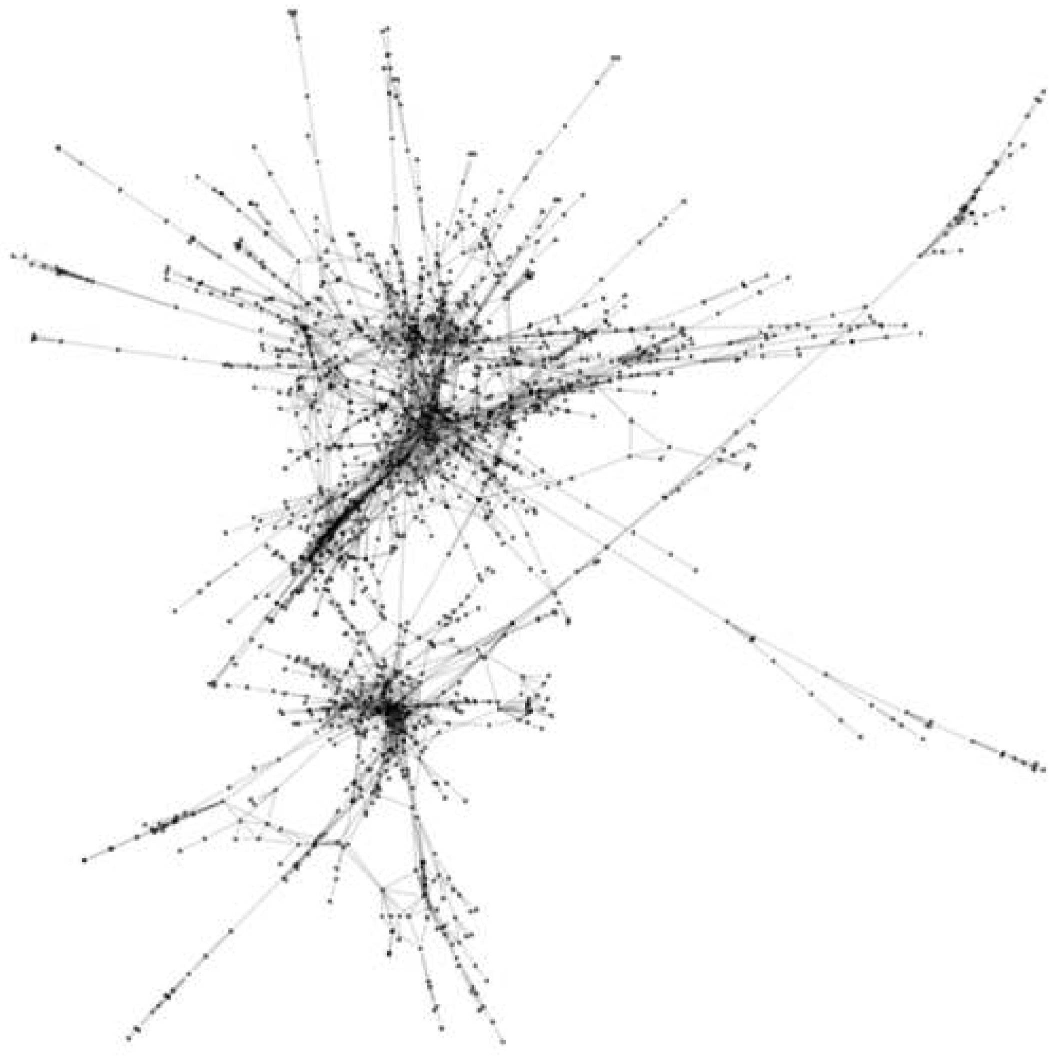

CL278
Low_complexity
Length of Reads (GP):1622 (0.02%)

Tgrandiflorum

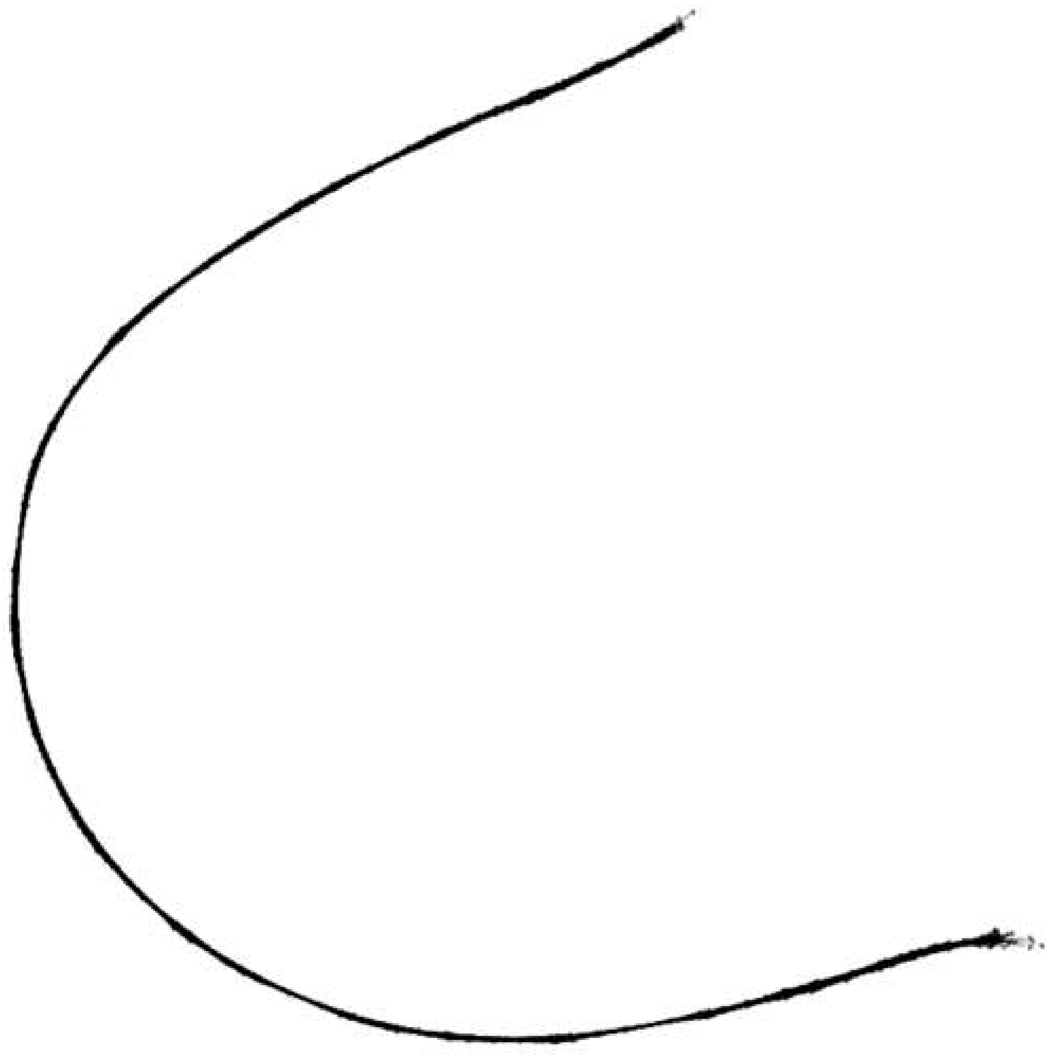

CL279
Low_complexity
Length of Reads (GP):1604 (0.02%)

Tgrandiflorum

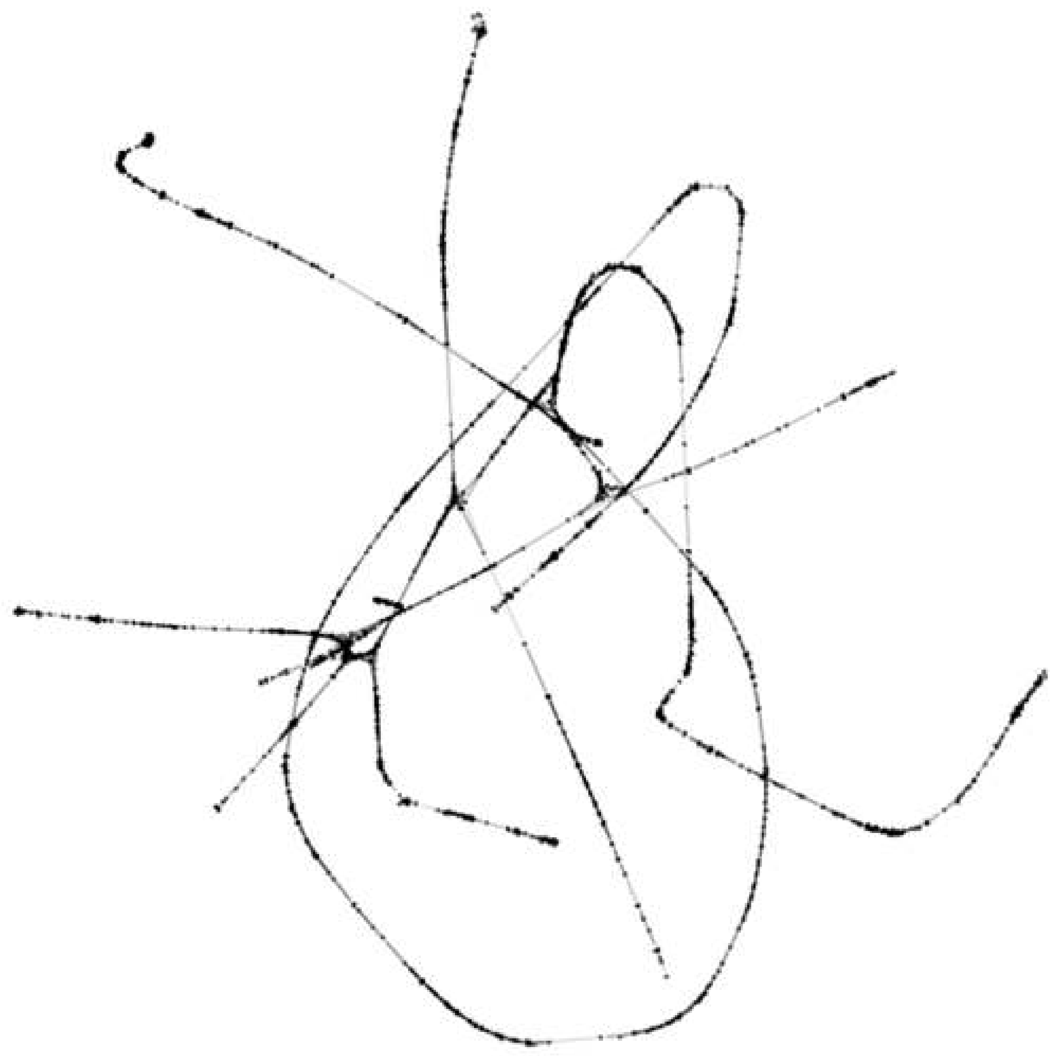

CL280
LTR_Copia
Length of Reads (GP):1588 (0.02%)

Tgrandiflorum

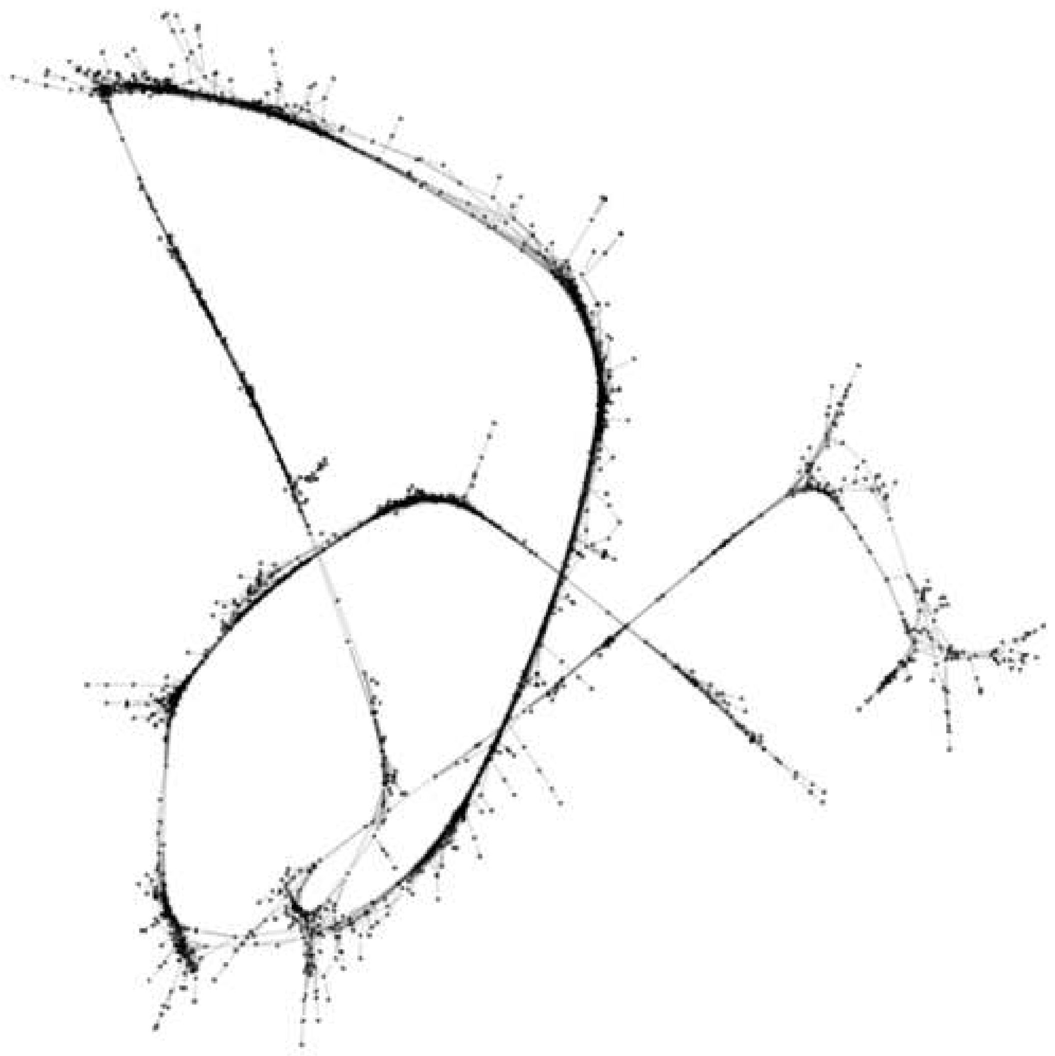

CL281
Low_complexity
Length of Reads (GP):1581 (0.02%)

Tgrandiflorum

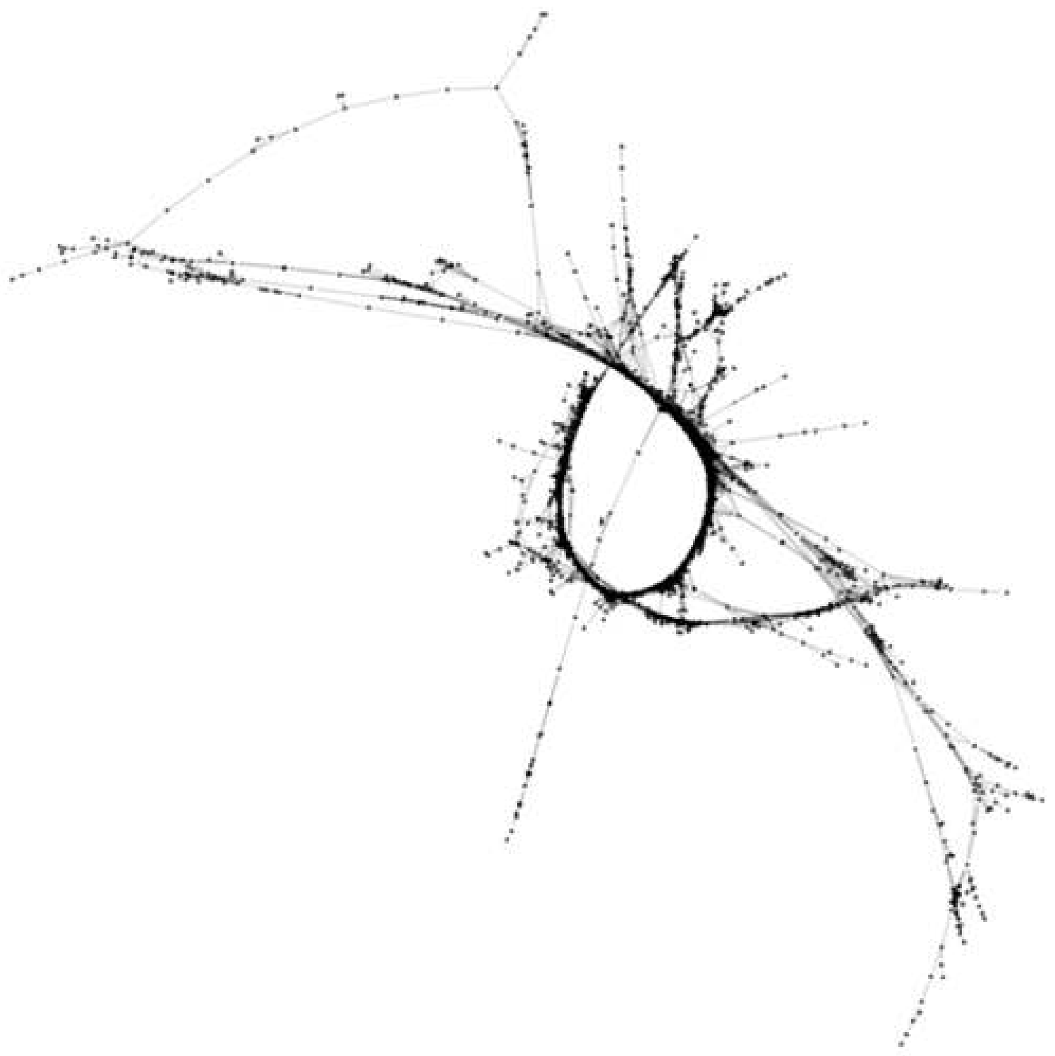

CL282
Low_complexity
Length of Reads (GP):1539 (0.02%)

Tgrandiflorum

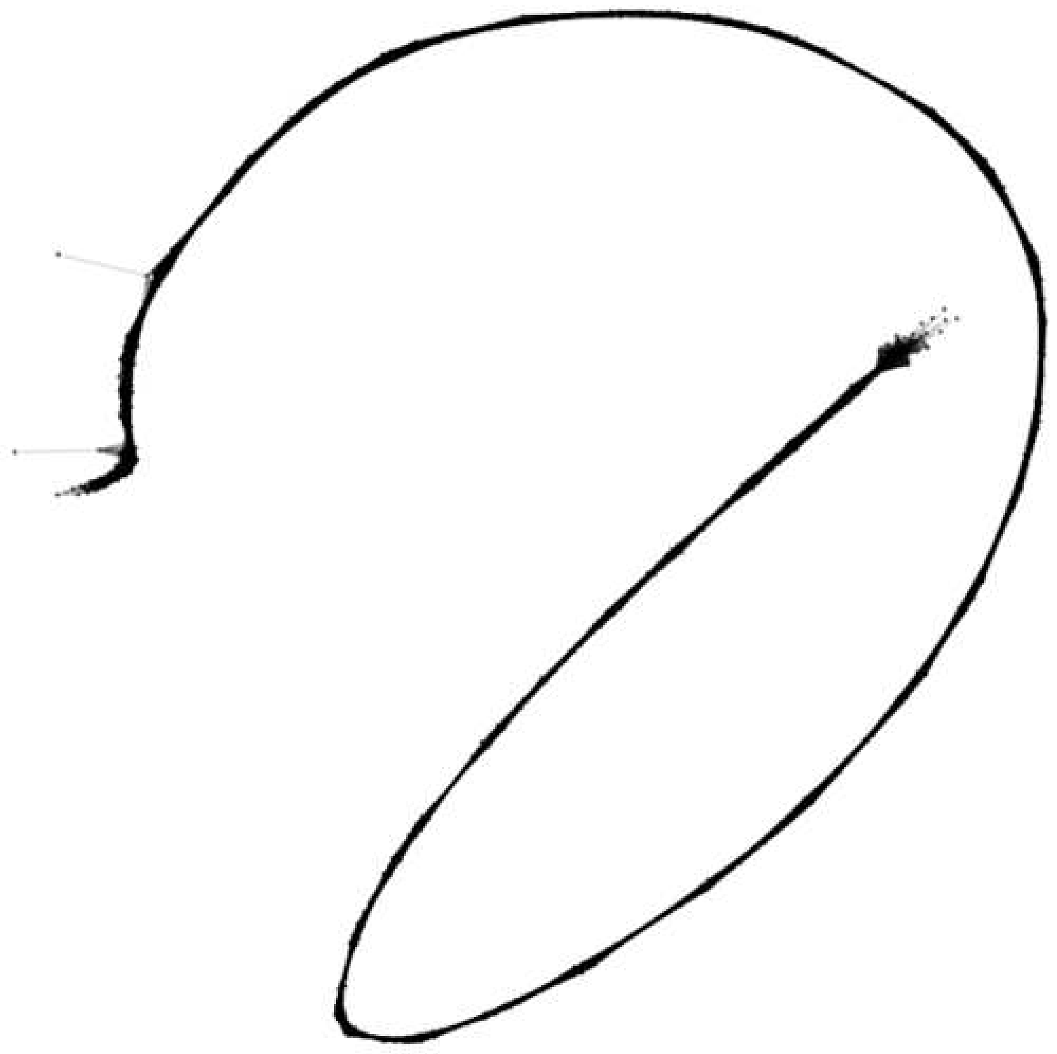

CL283
Low_complexity
Length of Reads (GP):1525 (0.02%)

Tgrandiflorum

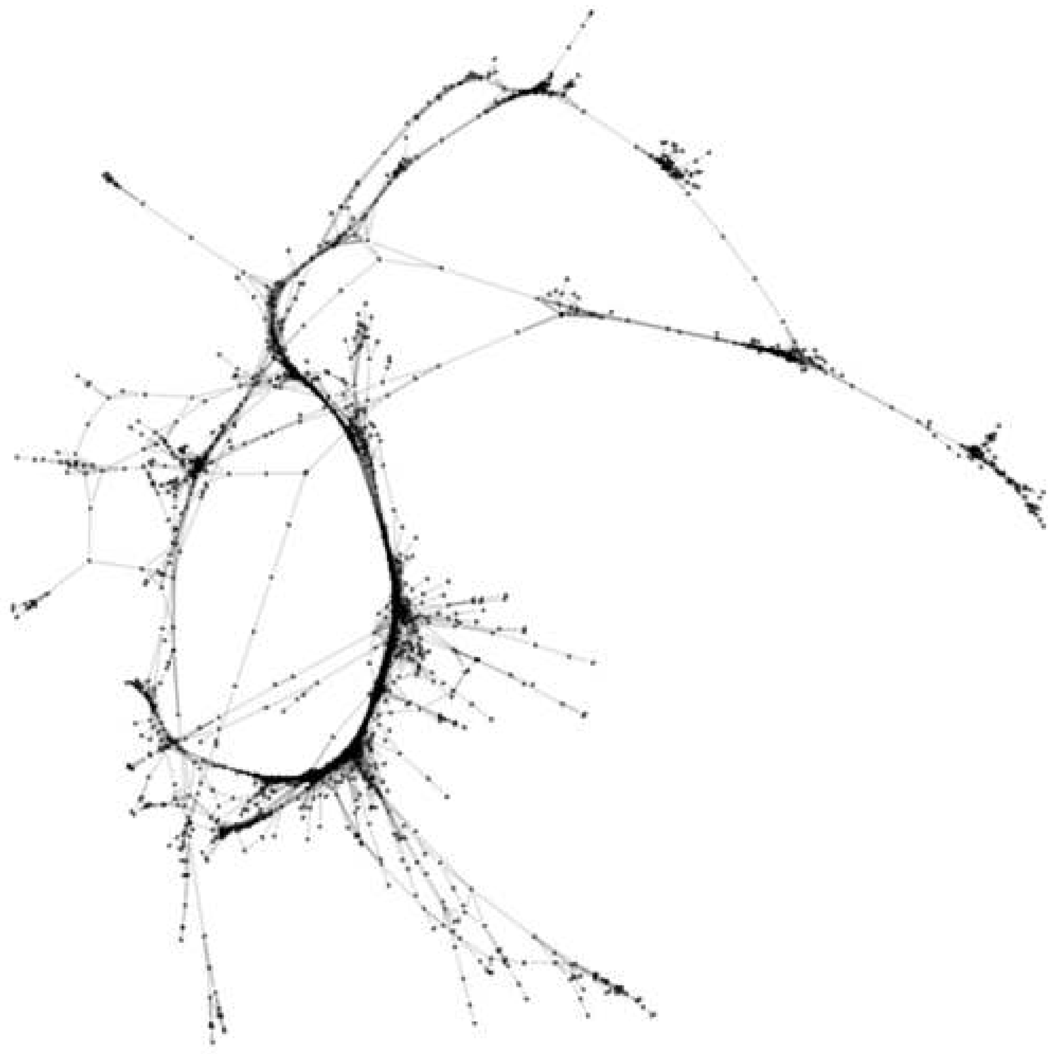

CL284
Low_complexity
Length of Reads (GP):1518 (0.02%)

Tgrandiflorum

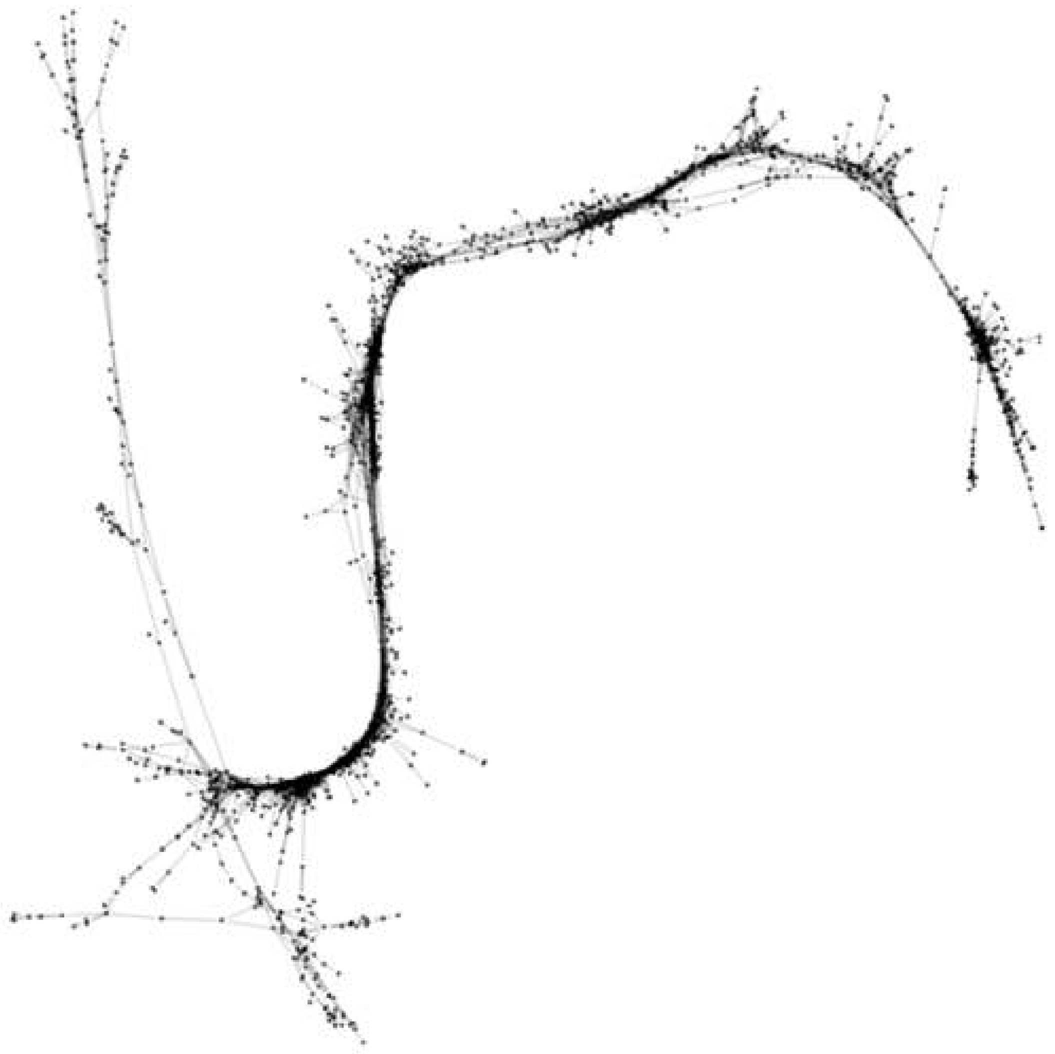

CL285
Low_complexity
Length of Reads (GP):1474 (0.02%)

Tgrandiflorum

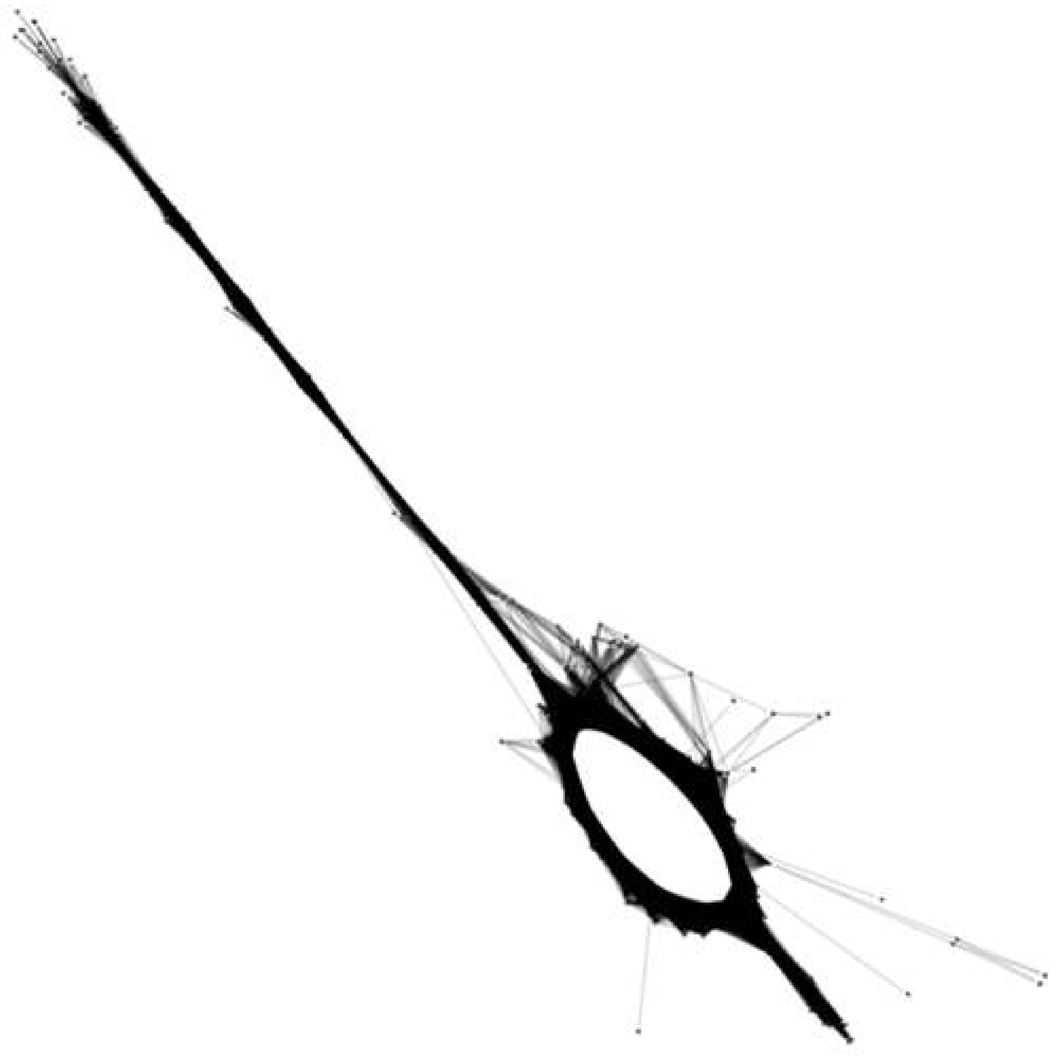

CL286
Low_complexity
Length of Reads (GP):1457 (0.02%)

Tgrandiflorum

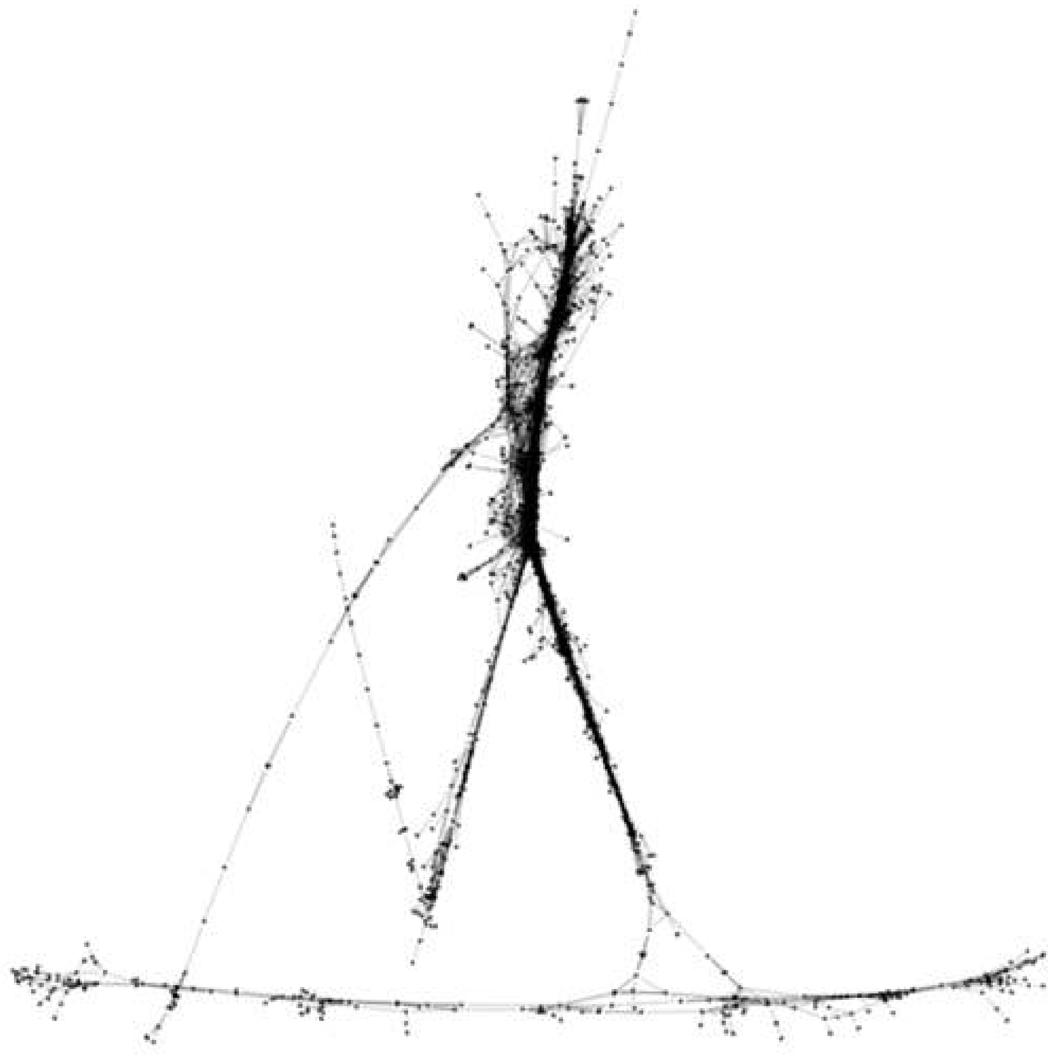

CL287
Low_complexity
Length of Reads (GP):1411 (0.02%)

Tgrandiflorum

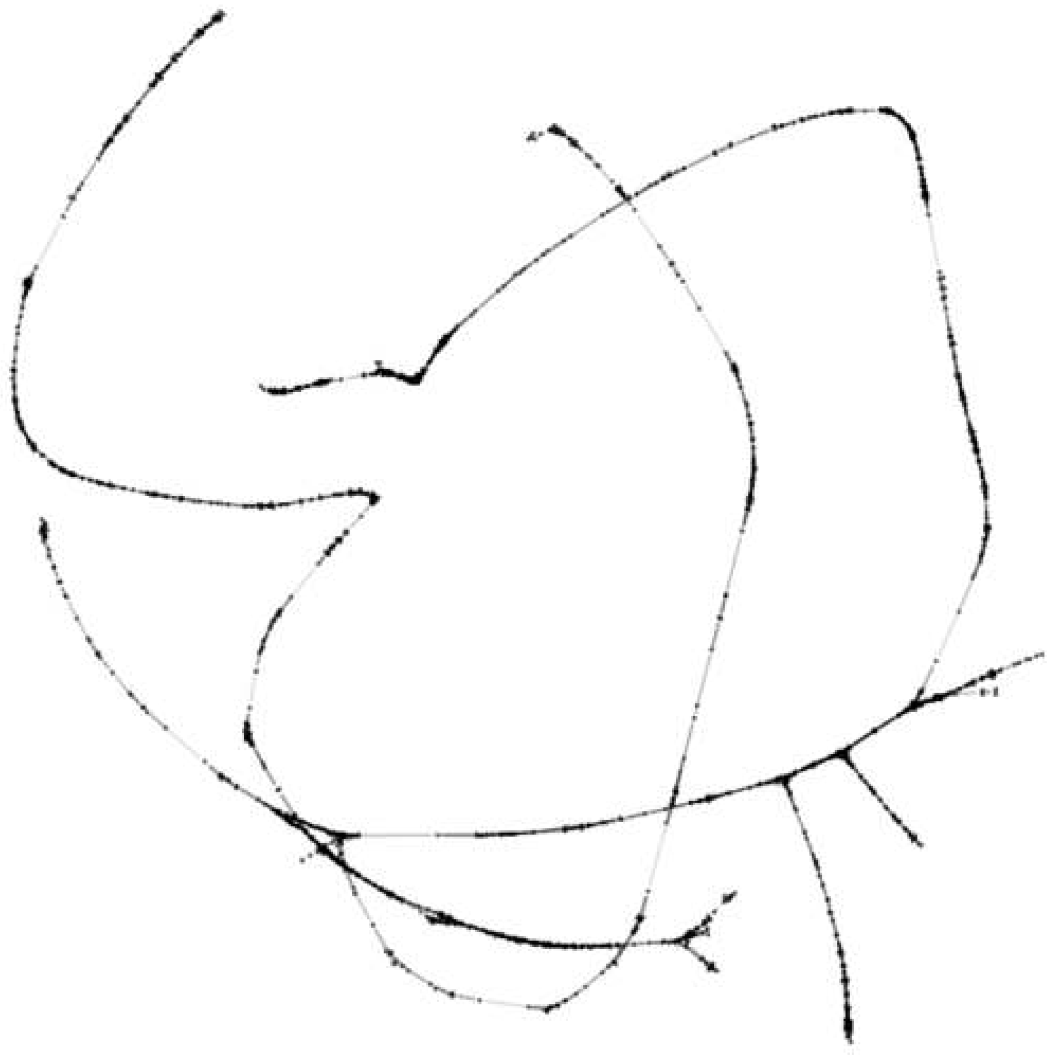

CL288
LTR_Copia
Length of Reads (GP):1395 (0.02%)

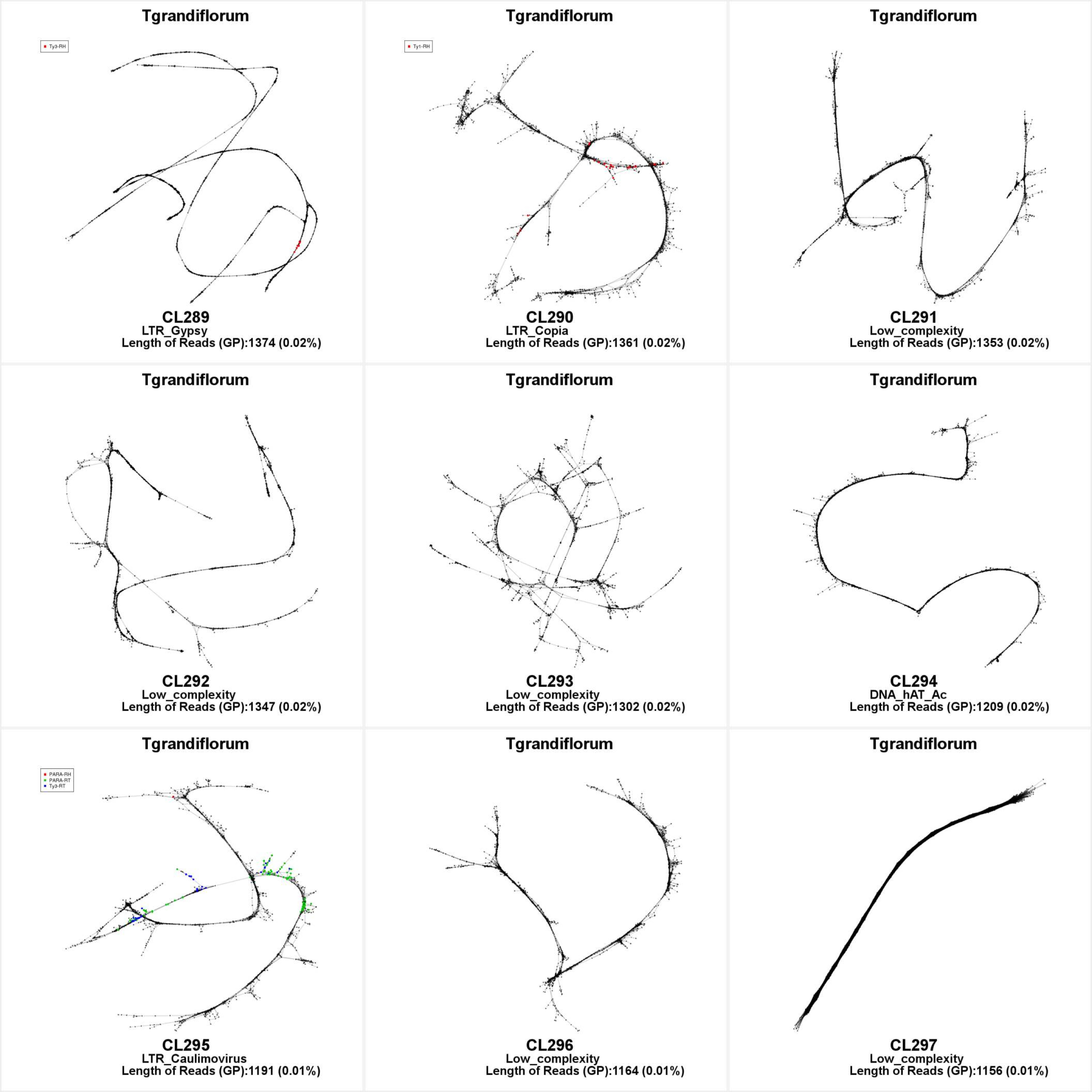
Tgrandiflorum
Ty3-RH
CL289
LTR_Gypsy
Length of Reads (GP):1374 (0.02%)
Tgrandiflorum
Ty1-RH
CL290
LTR_Copia
Length of Reads (GP):1361 (0.02%)
Tgrandiflorum
CL291
Low_complexity
Length of Reads (GP):1353 (0.02%)
Tgrandiflorum
CL292
Low_complexity
Length of Reads (GP):1347 (0.02%)
Tgrandiflorum
CL293
Low_complexity
Length of Reads (GP):1302 (0.02%)
Tgrandiflorum
CL294
DNA_hAT_Ac
Length of Reads (GP):1209 (0.02%)
Tgrandiflorum
PARA-RH
PARA-RT
Ty3-RT
CL295
LTR_Caulimovirus
Length of Reads (GP):1191 (0.01%)
Tgrandiflorum
CL296
Low_complexity
Length of Reads (GP):1164 (0.01%)
Tgrandiflorum
CL297
Low_complexity
Length of Reads (GP):1156 (0.01%)

Tgrandiflorum

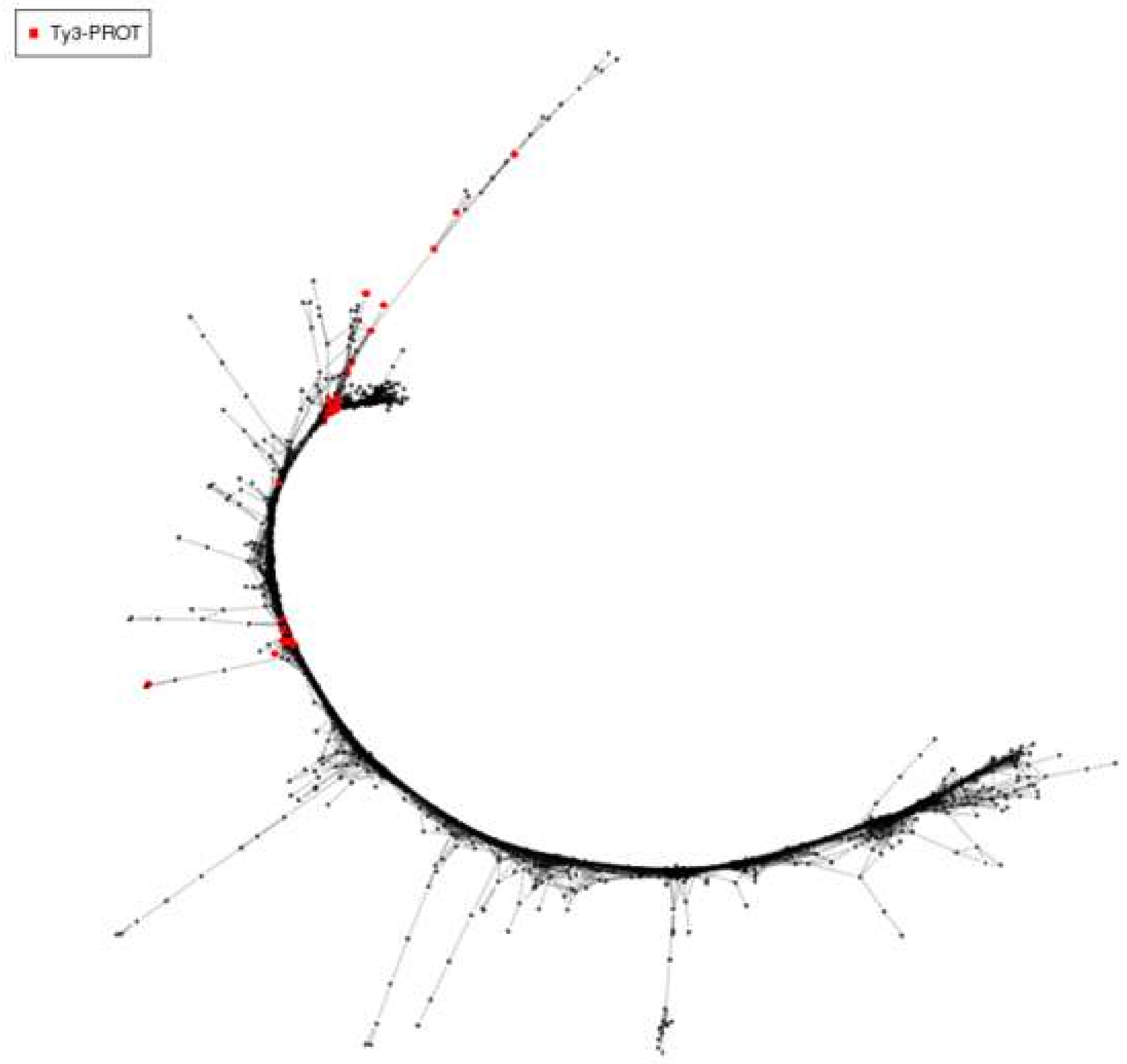

CL298
LTR_Gypsy
Length of Reads (GP):1121 (0.01%)

Tgrandiflorum

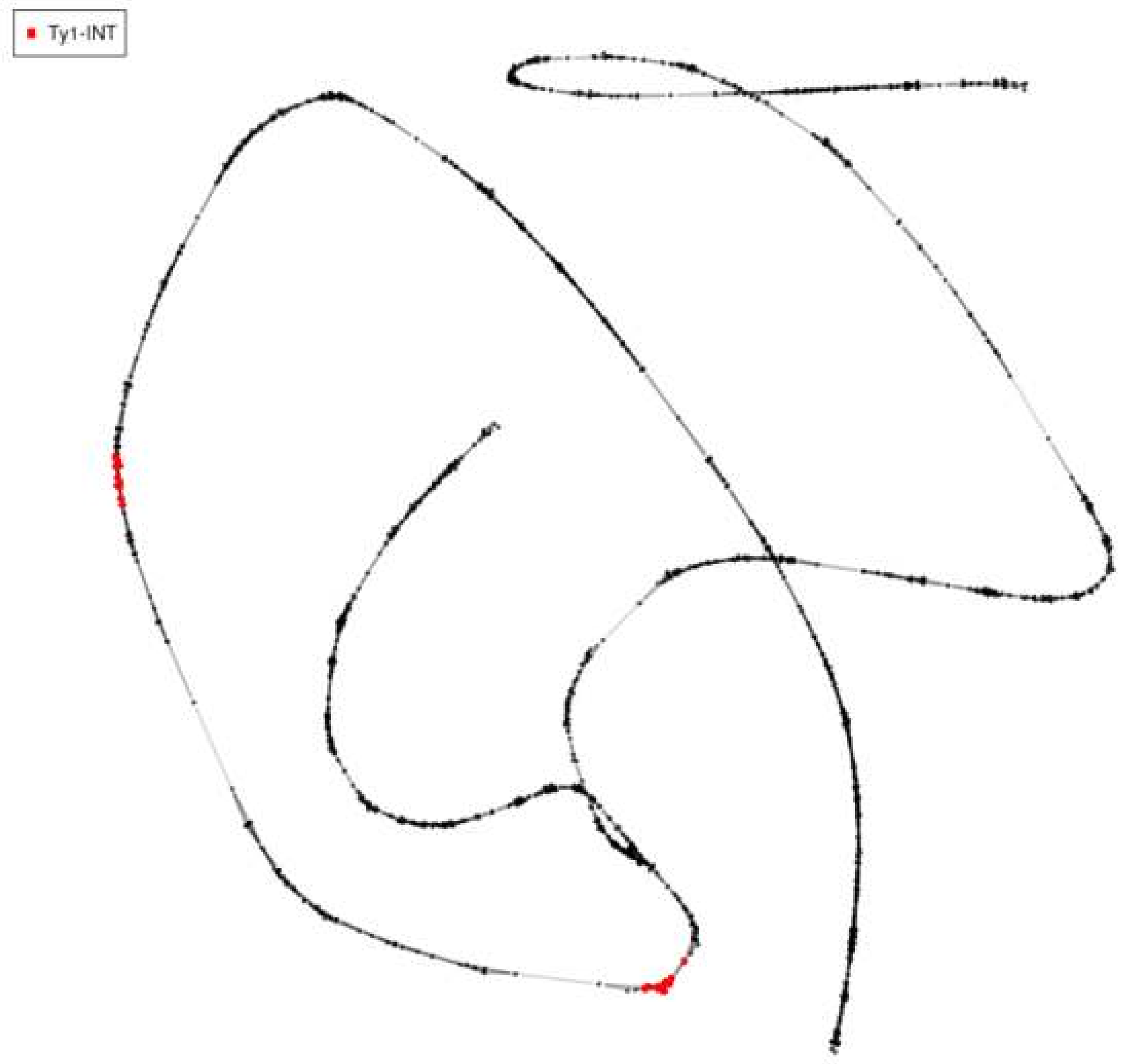

CL299
LTR_Copia
Length of Reads (GP):1117 (0.01%)

Tgrandiflorum

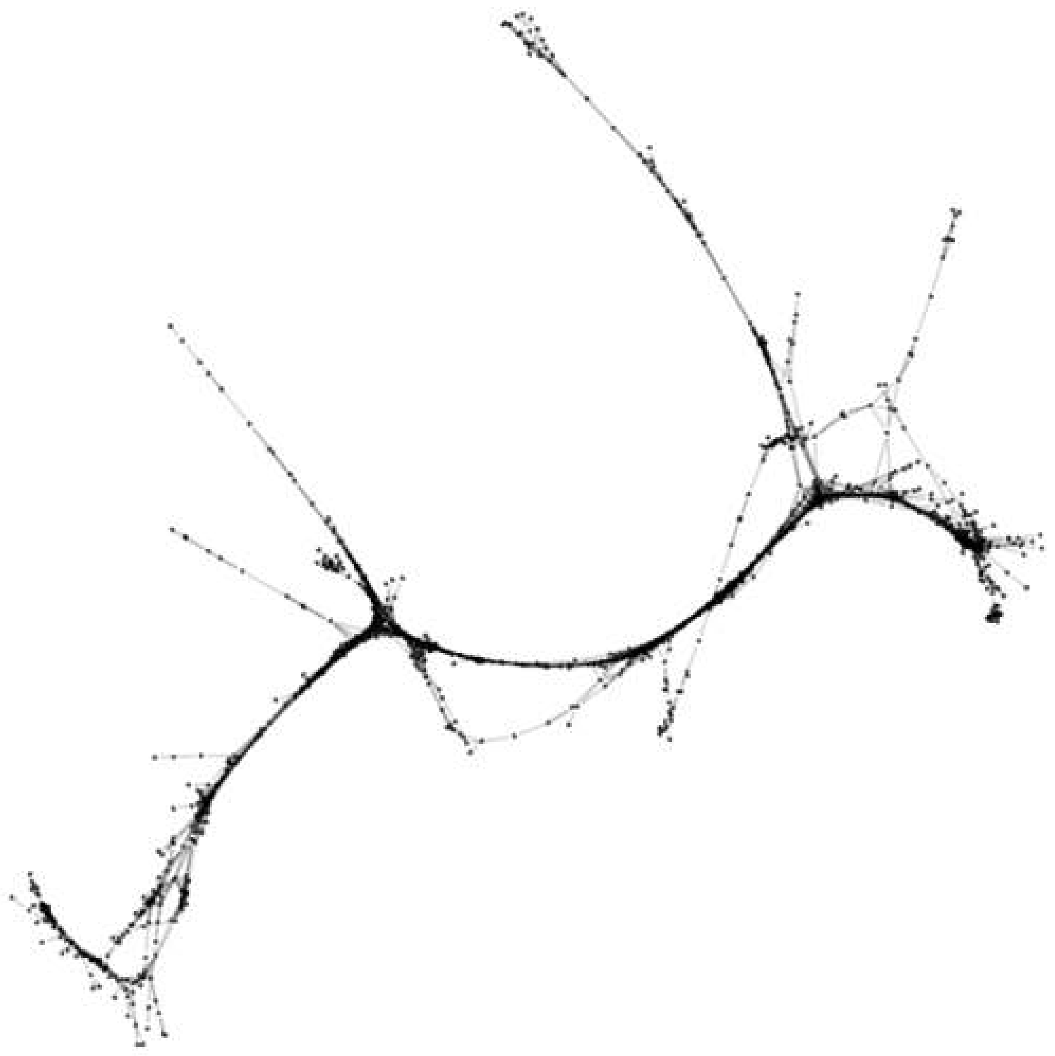

CL300
LTR_Copia
Length of Reads (GP):1111 (0.01%)

Tgrandiflorum

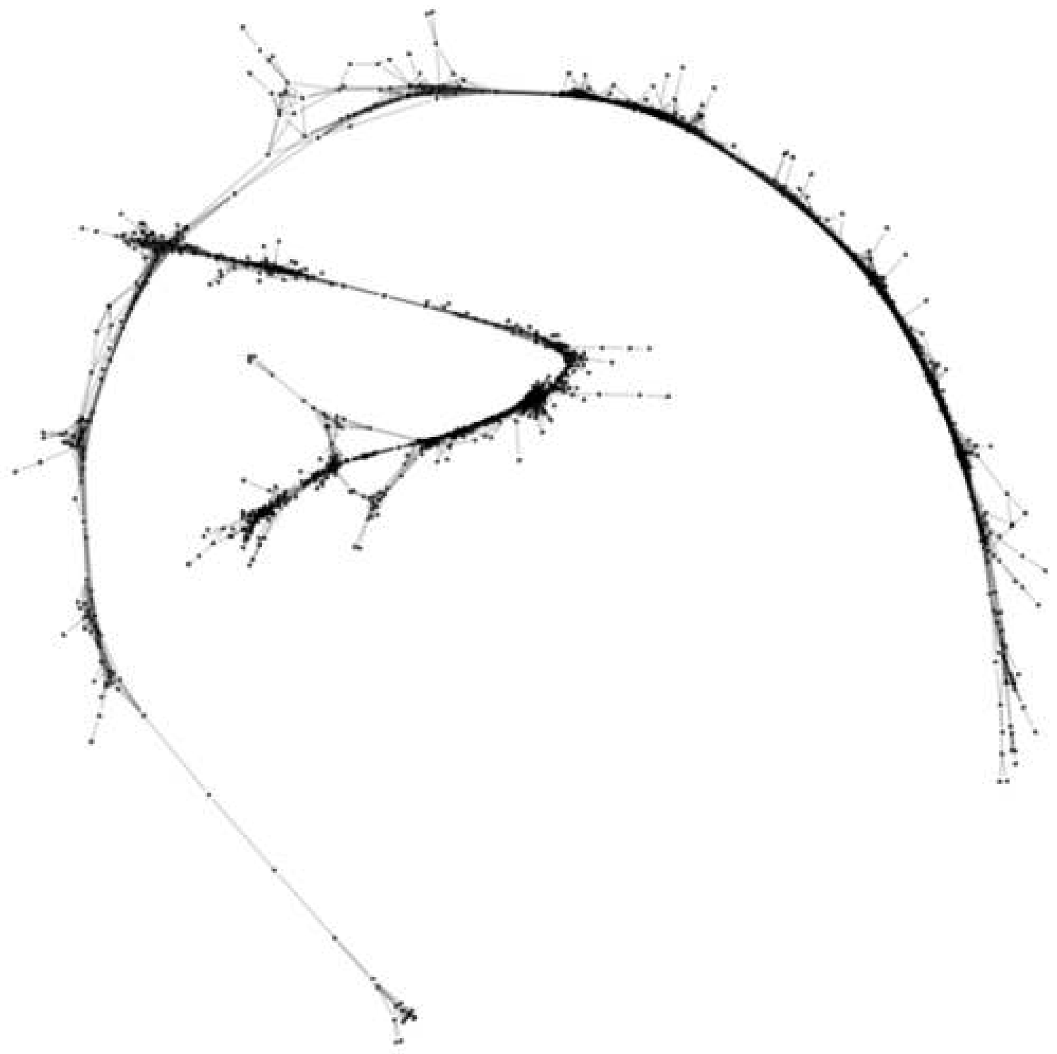

CL301
LTR_Copia
Length of Reads (GP):1069 (0.01%)

Tgrandiflorum

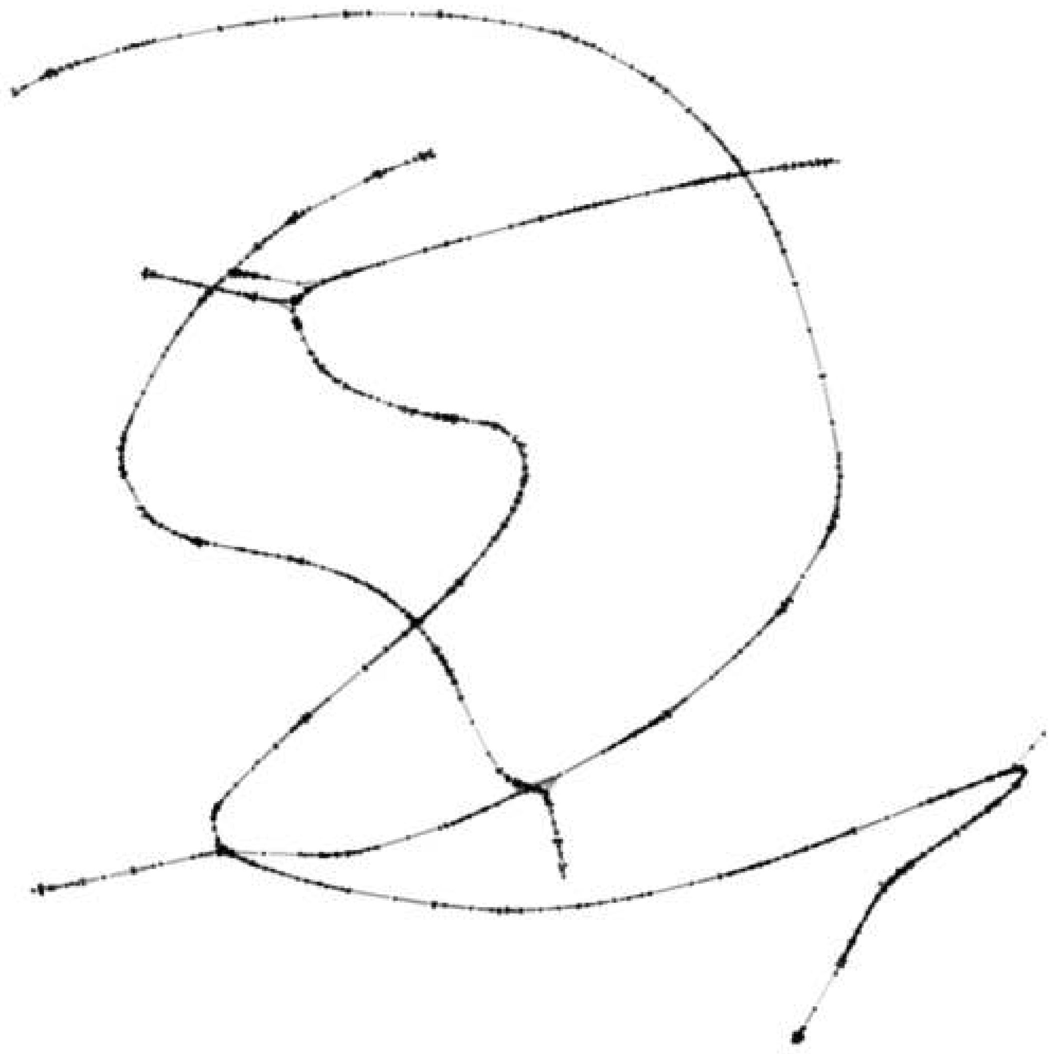

CL302
Low_complexity
Length of Reads (GP):1053 (0.01%)

Tgrandiflorum

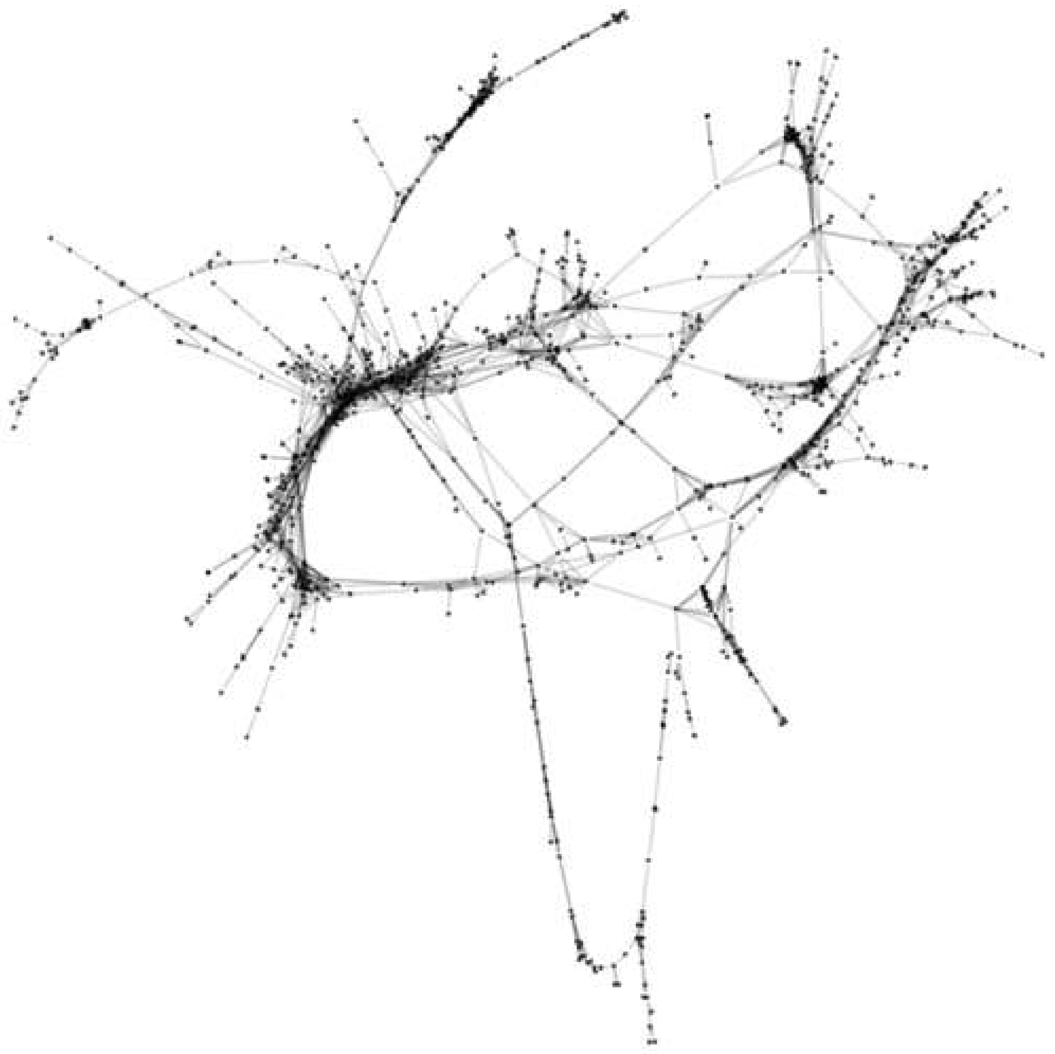

CL303
Low_complexity
Length of Reads (GP):1012 (0.01%)

Tgrandiflorum

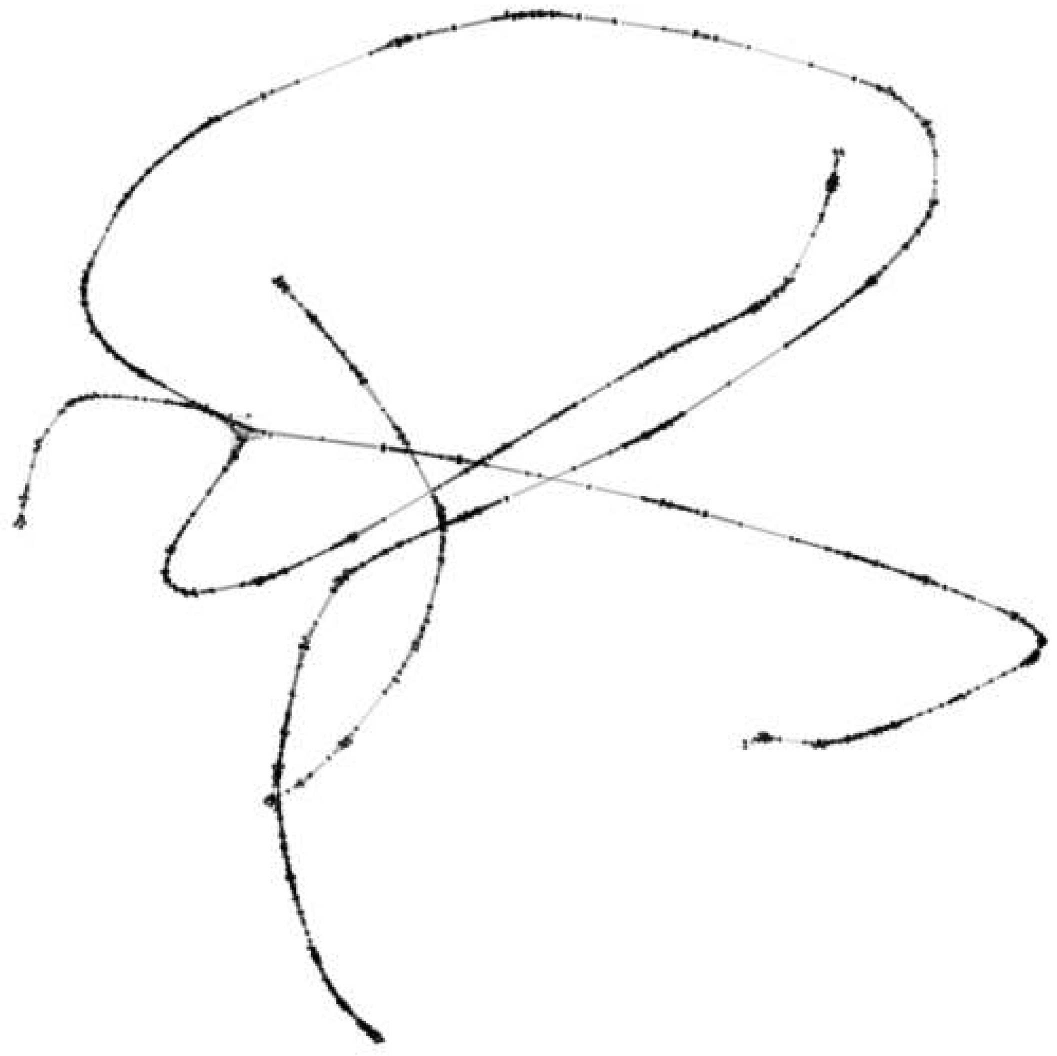

CL304
LTR_Copia
Length of Reads (GP):1009 (0.01%)

Tgrandiflorum

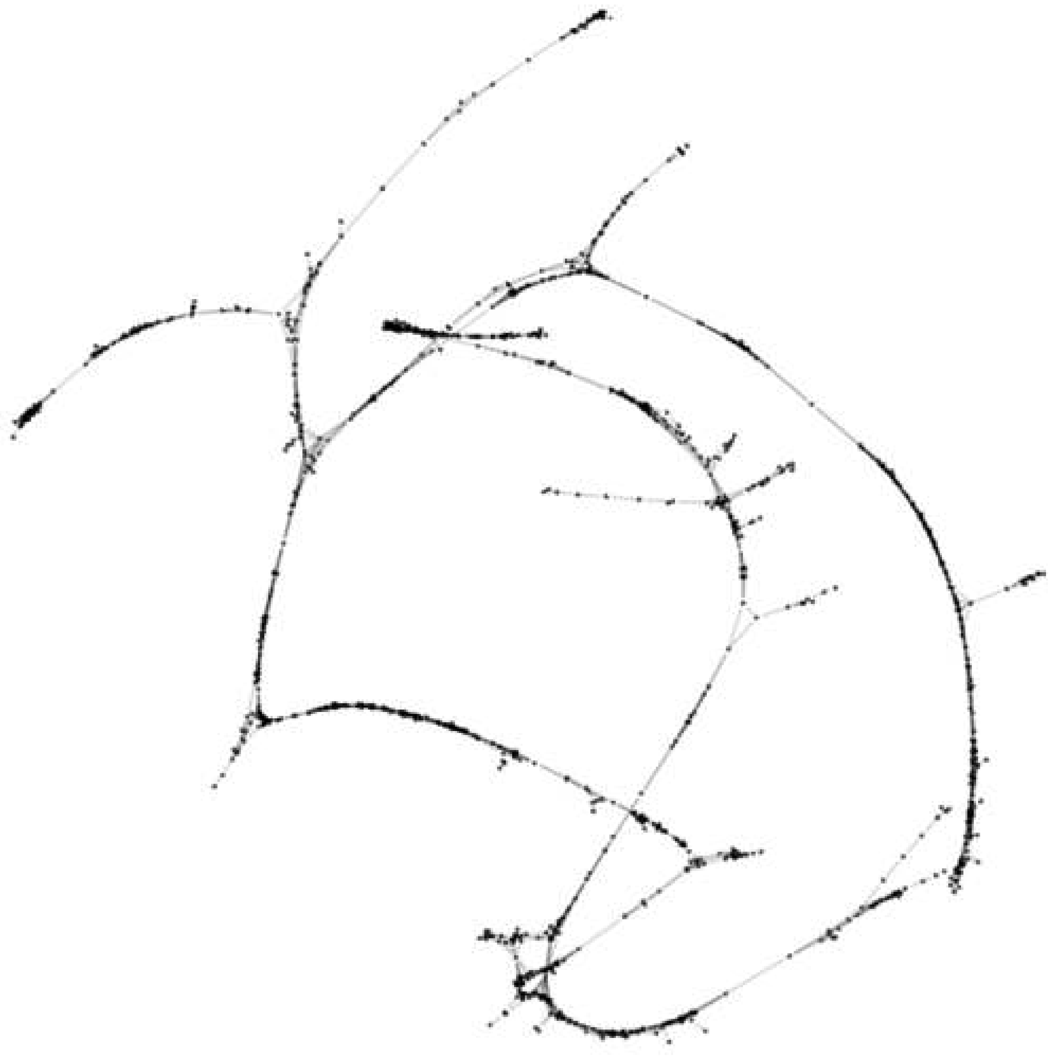

CL305
LTR_Copia
Length of Reads (GP):1003 (0.01%)

Tgrandiflorum

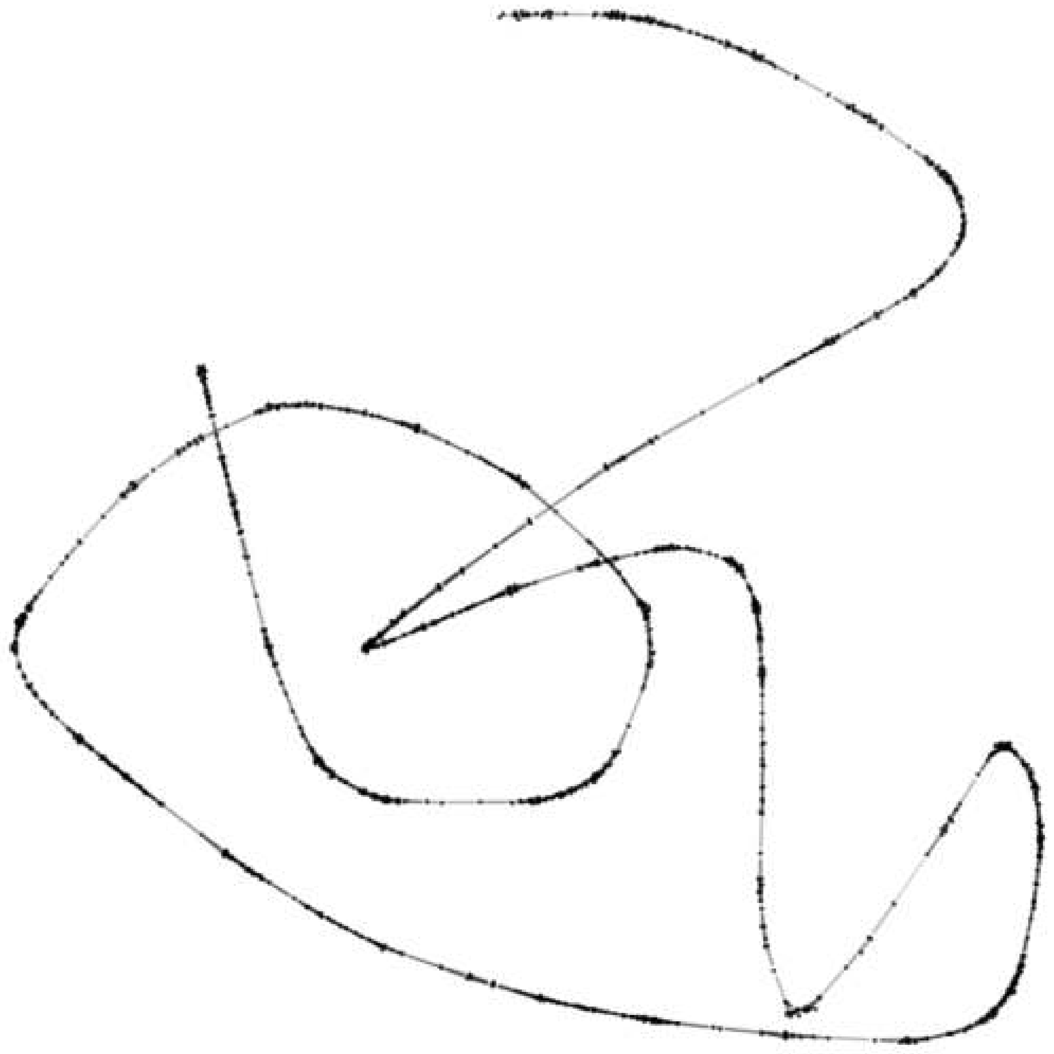

CL306
LTR_Copia
Length of Reads (GP):993 (0.01%)

Tgrandiflorum

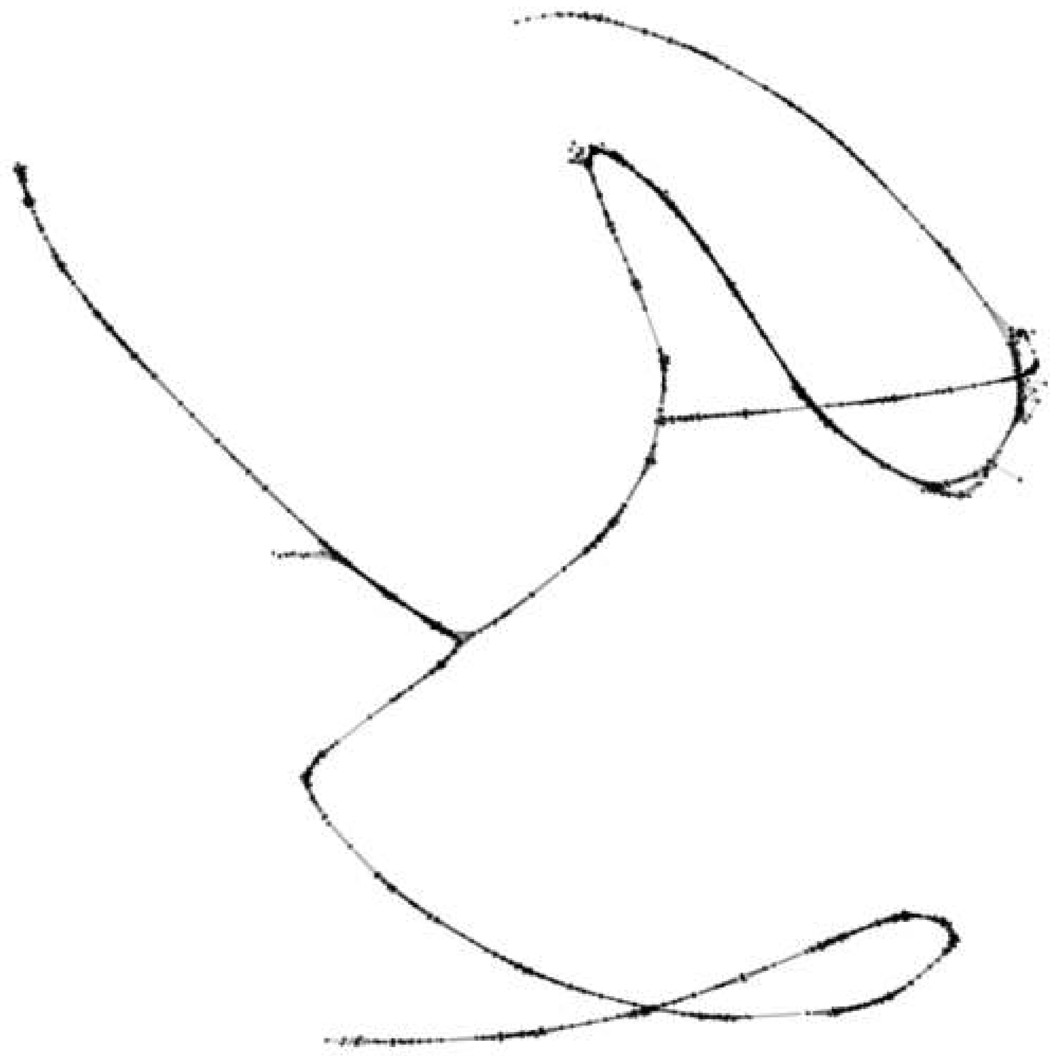

CL307
LTR_Copia
Length of Reads (GP):968 (0.01%)

Tgrandiflorum

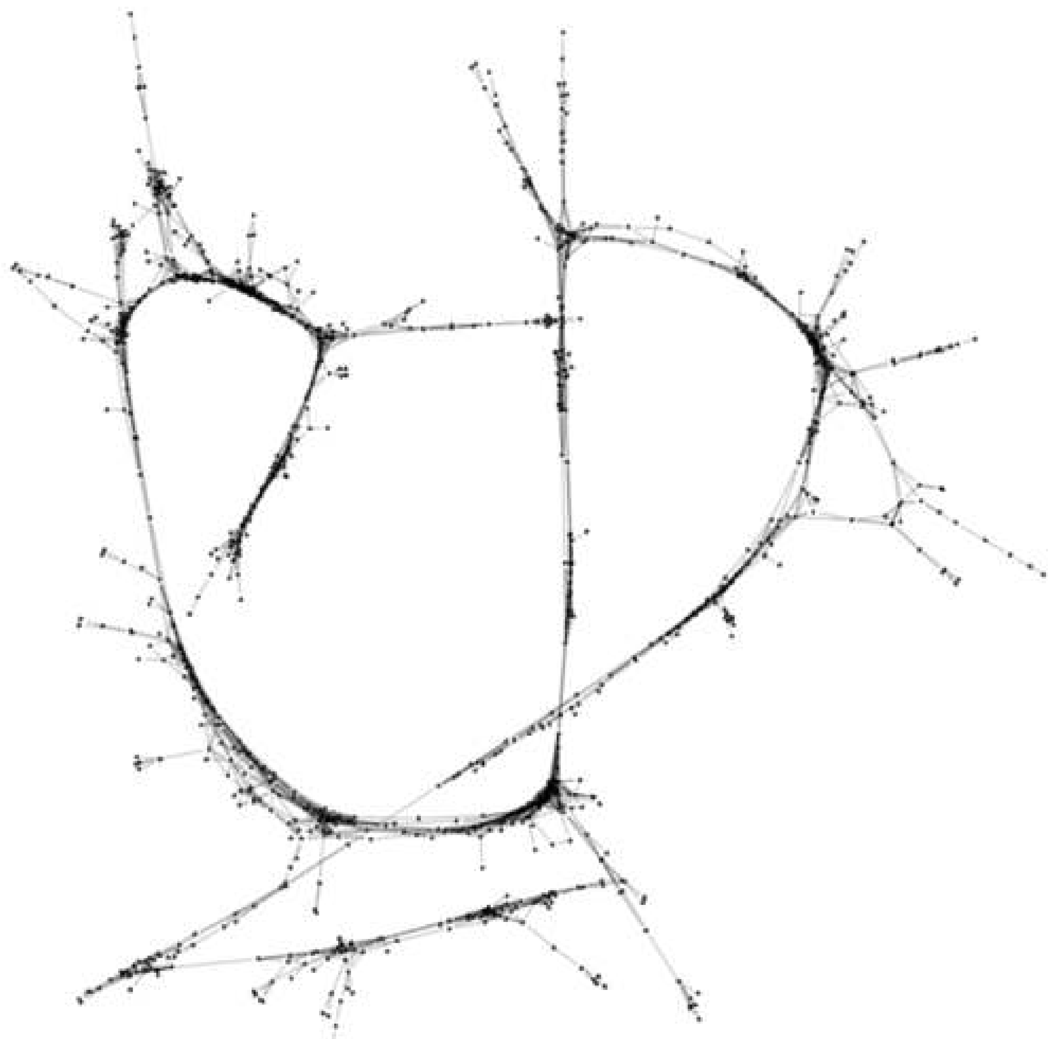

CL308
LTR_Caulimovirus
Length of Reads (GP):963 (0.01%)

Tgrandiflorum

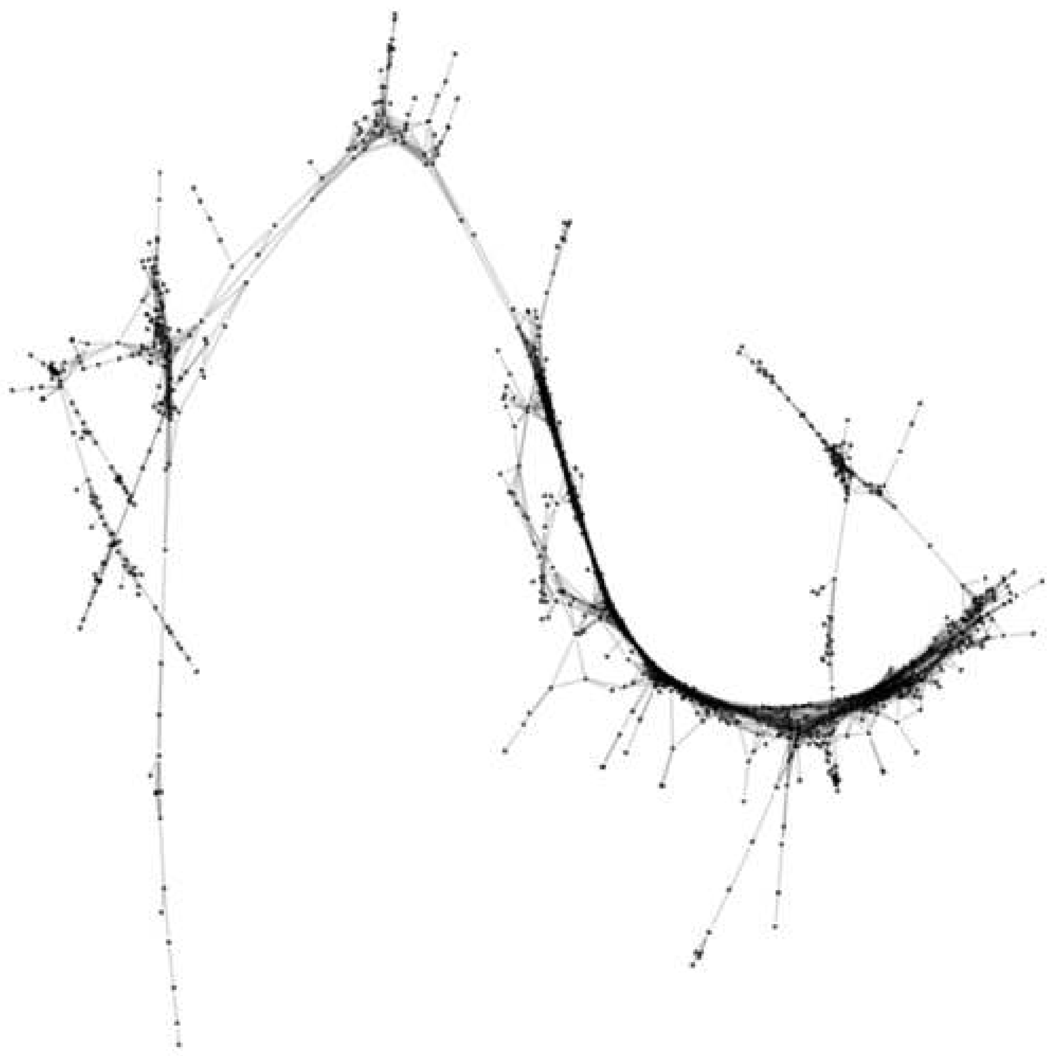

CL309
Low_complexity
Length of Reads (GP):929 (0.01%)

Tgrandiflorum

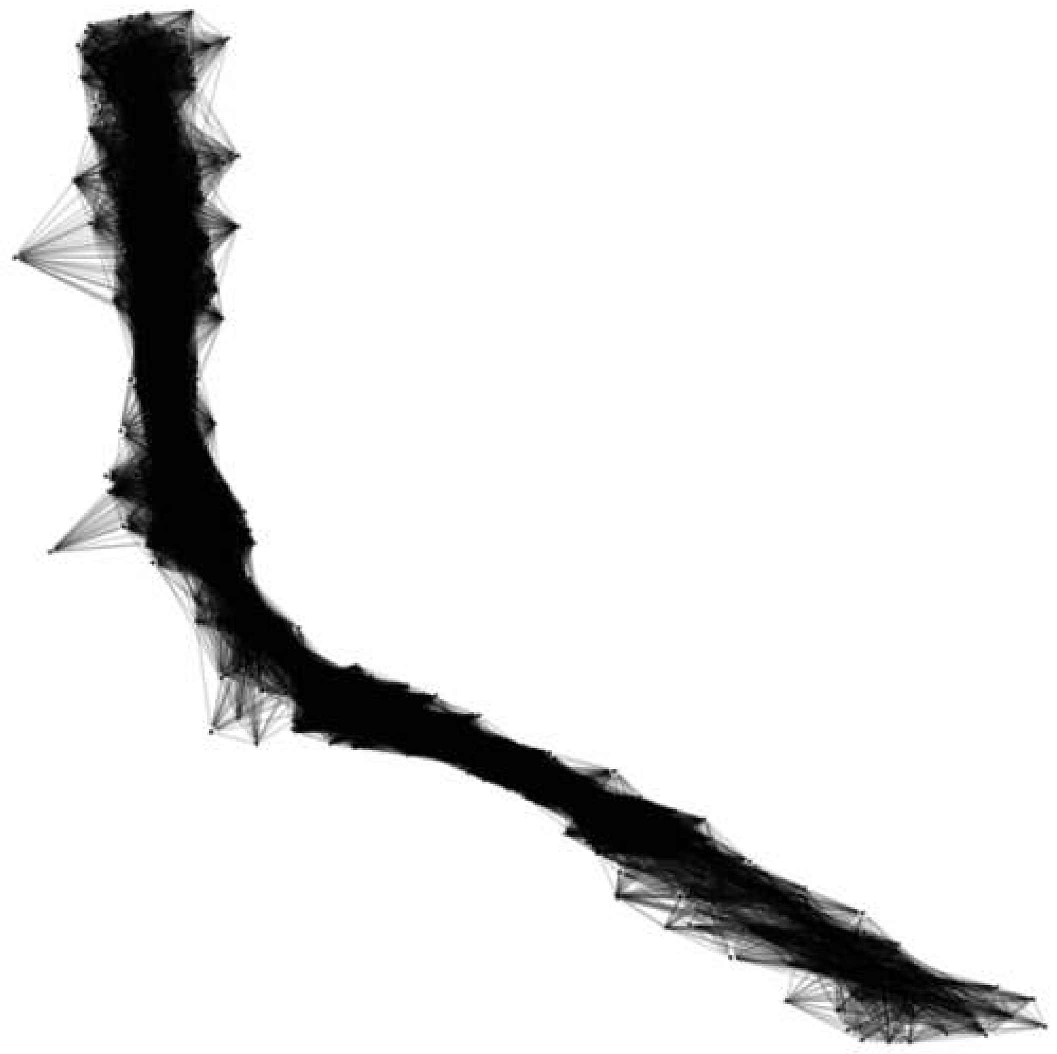

CL310
Low_complexity
Length of Reads (GP):928 (0.01%)

Tgrandiflorum

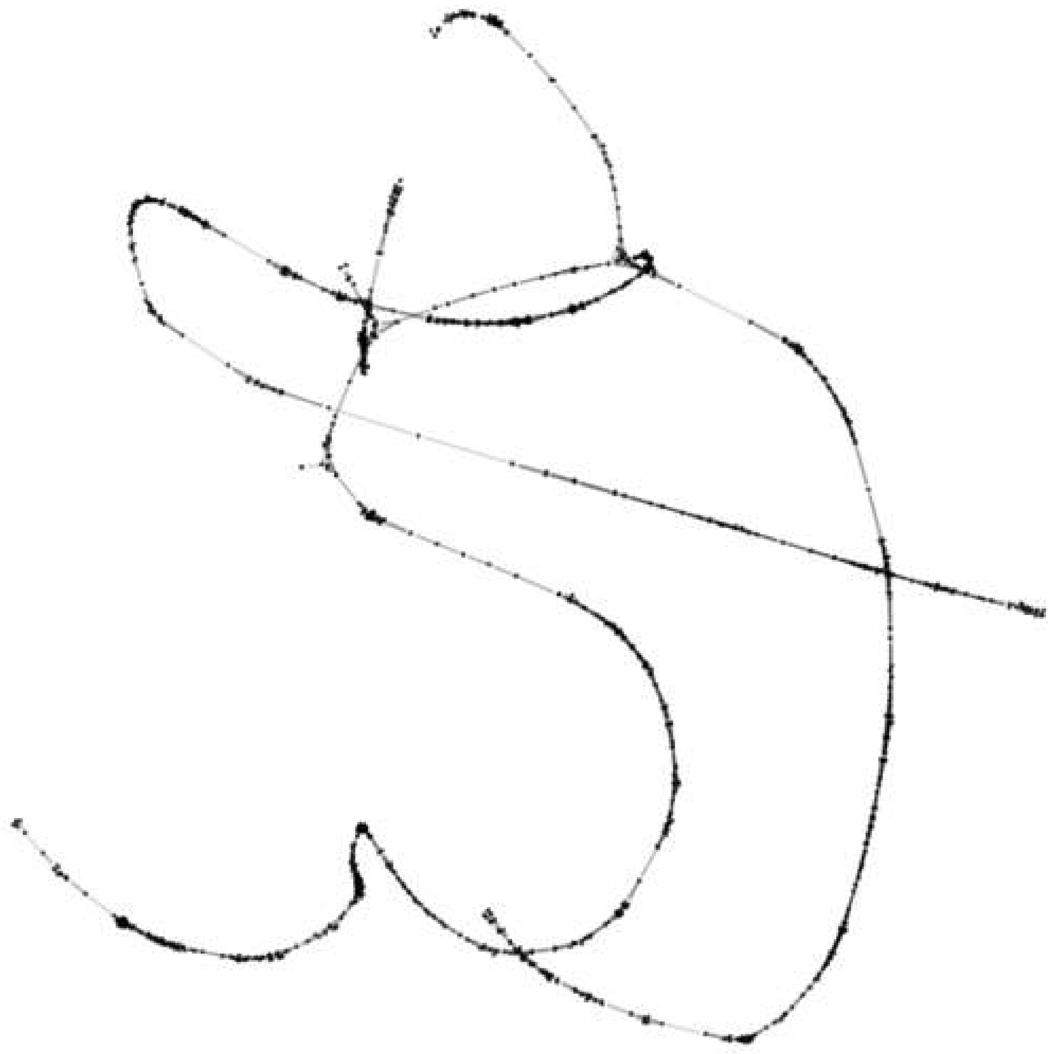

CL311
LTR_Copia
Length of Reads (GP):905 (0.01%)

Tgrandiflorum

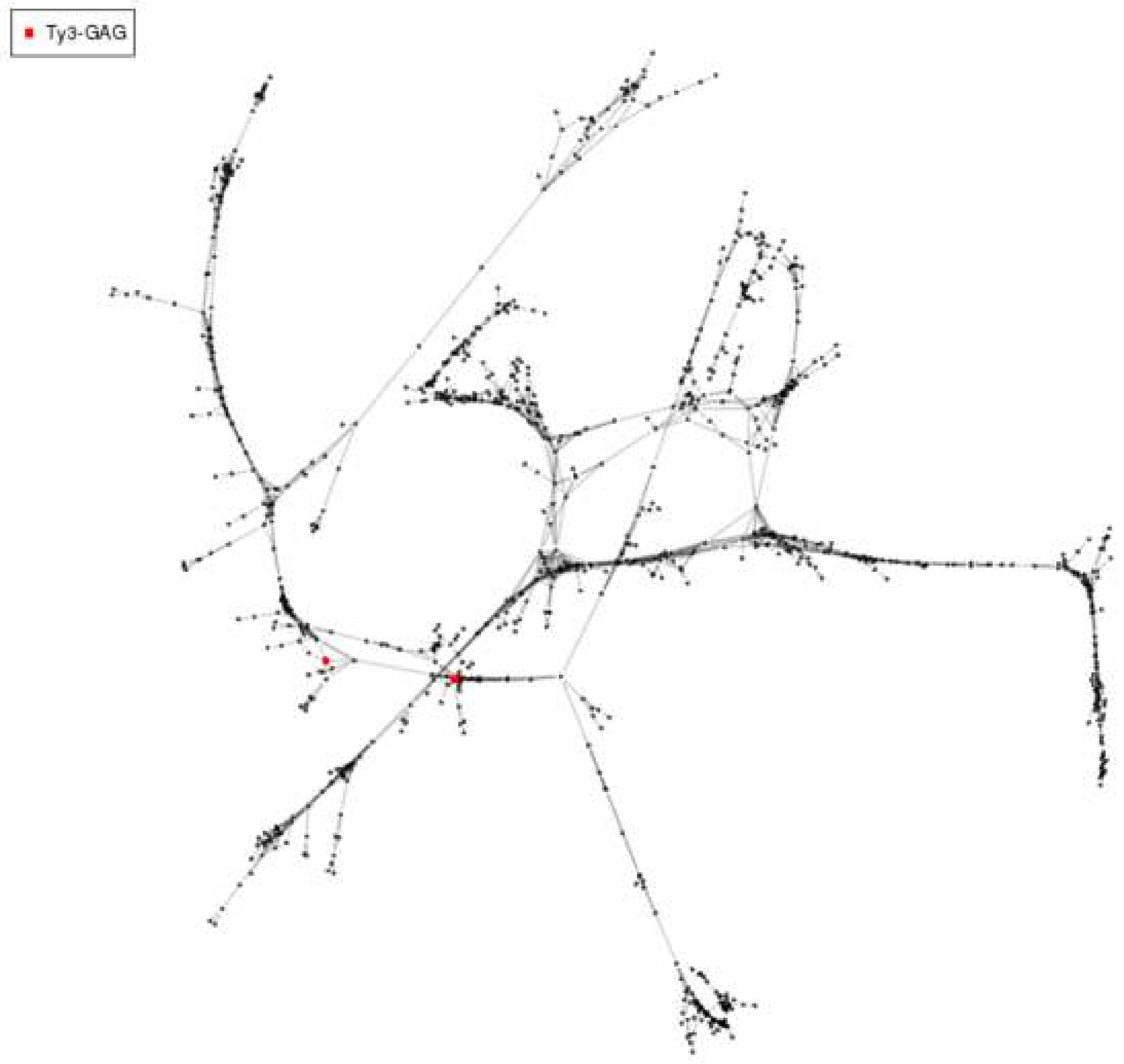

CL312
LTR_Copia
Length of Reads (GP):903 (0.01%)

Tgrandiflorum

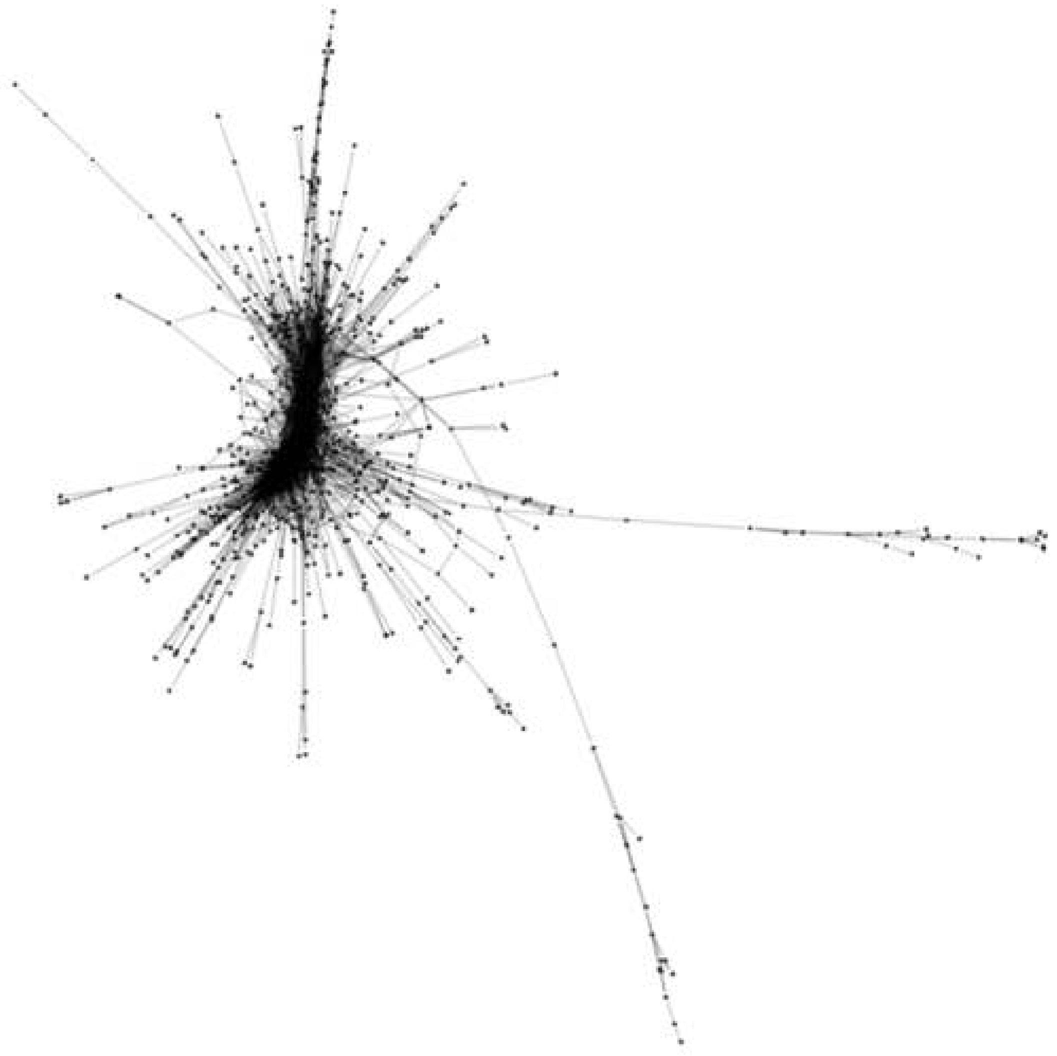

CL313
Low_complexity
Length of Reads (GP):888 (0.01%)

Tgrandiflorum

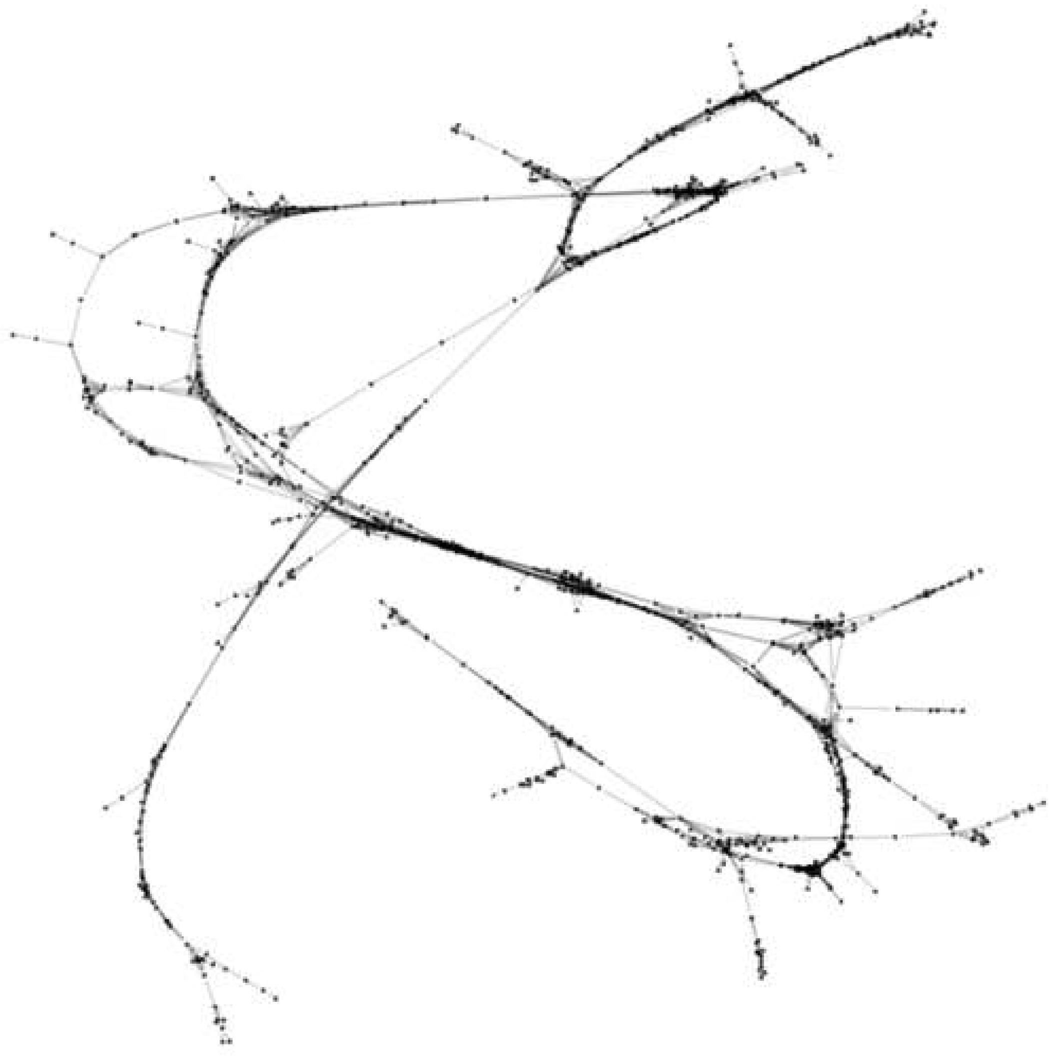

CL314
LTR_Copia
Length of Reads (GP):875 (0.01%)

Tgrandiflorum

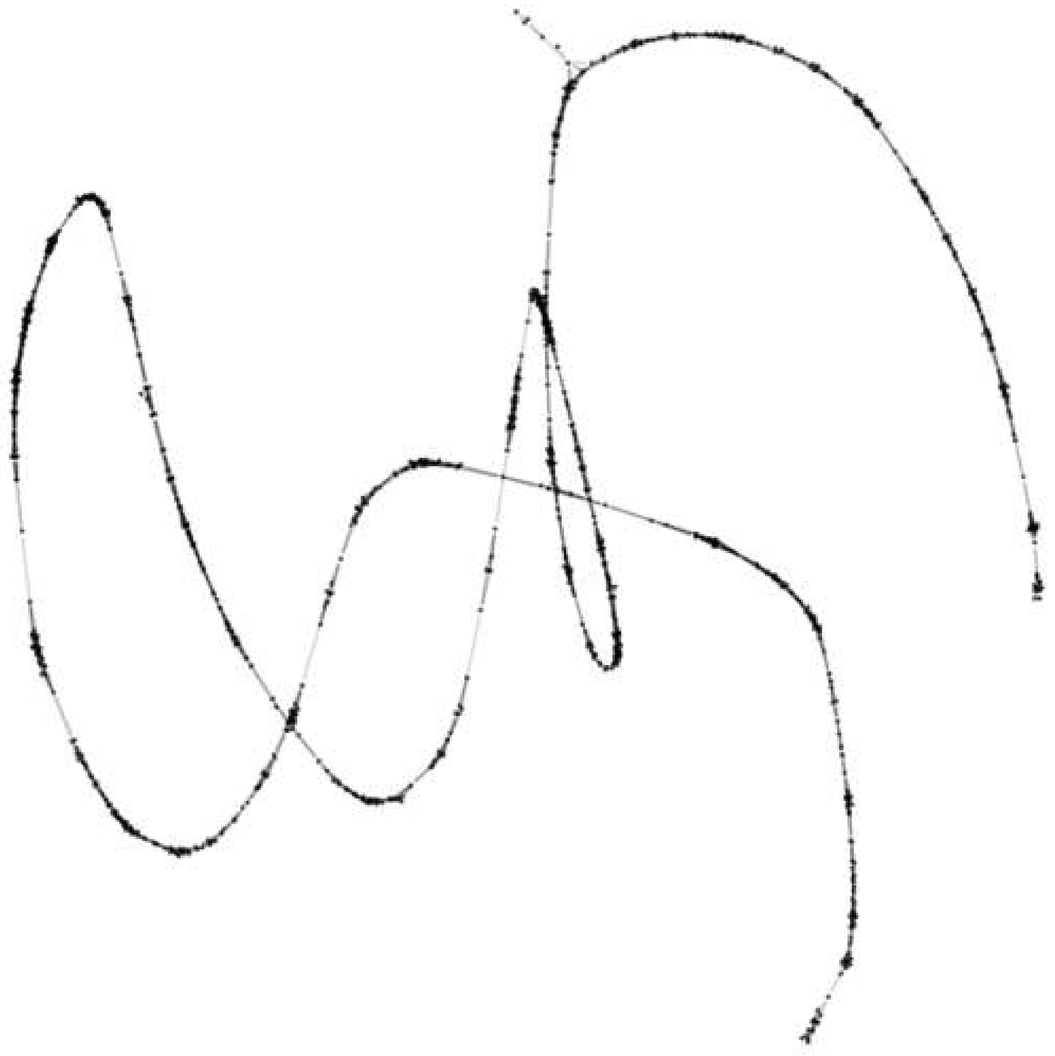

CL315
LTR_Copia
Length of Reads (GP):850 (0.01%)

Tgrandiflorum

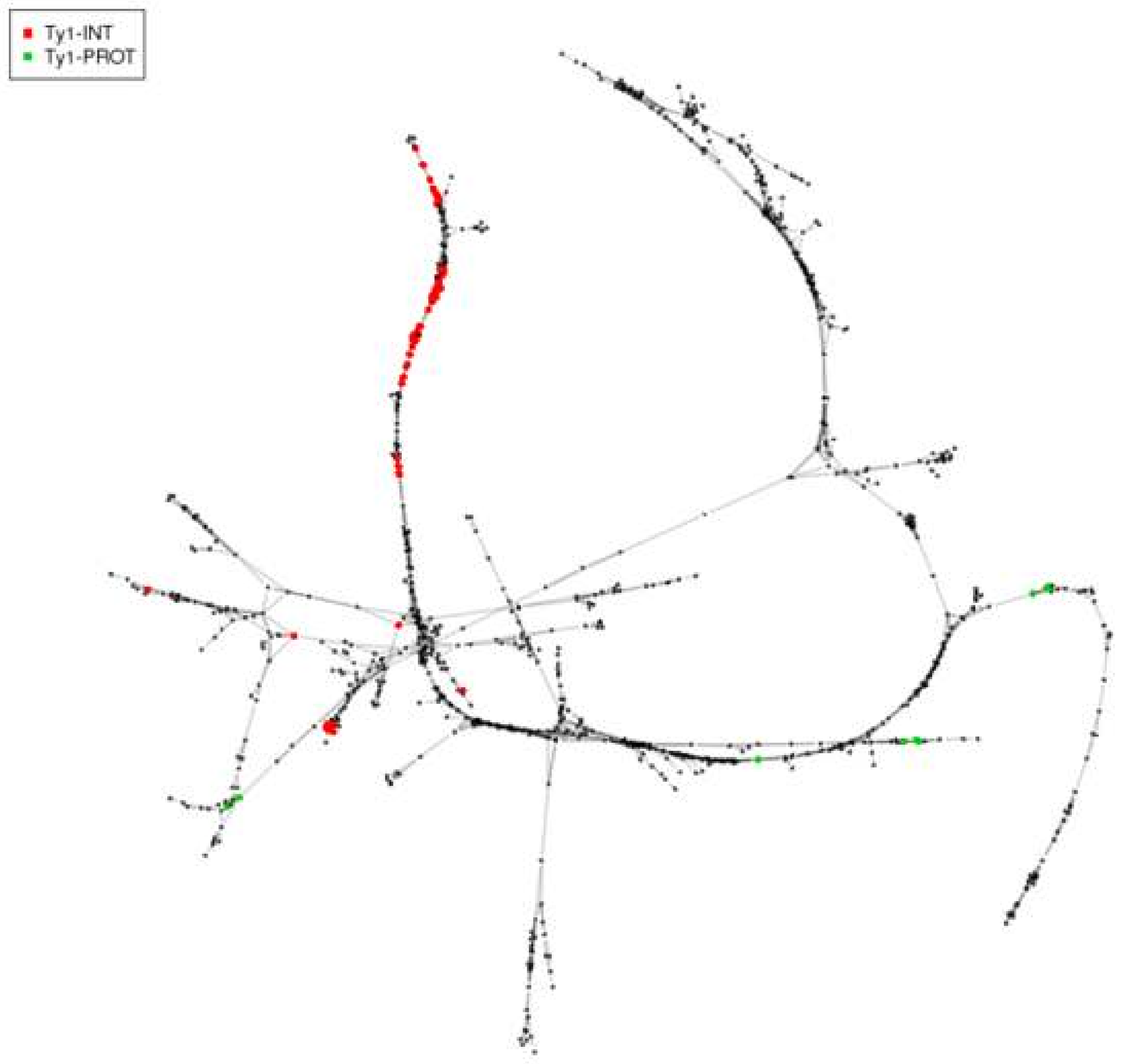

CL316
LTR_Copia
Length of Reads (GP):835 (0.01%)

Tgrandiflorum

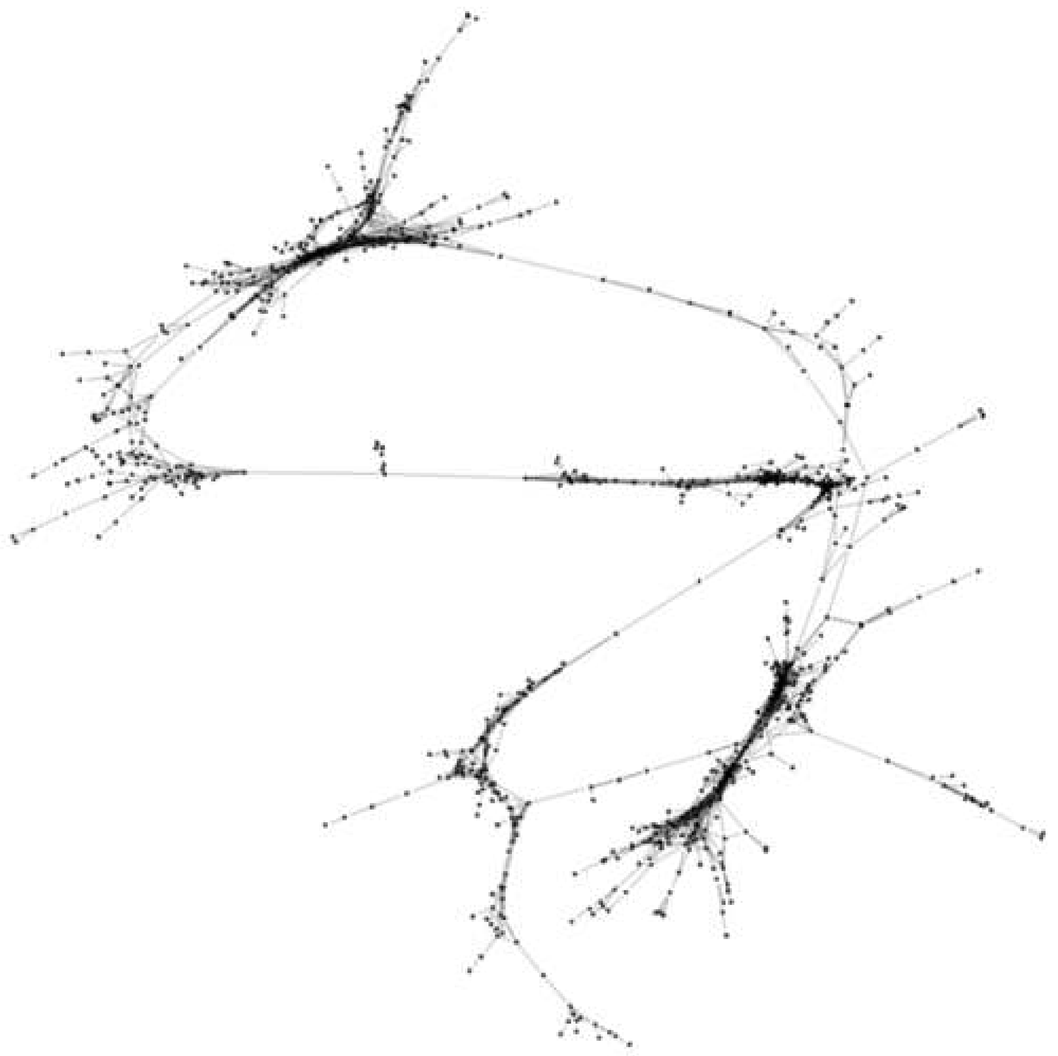

CL317
Simple_repeat
Length of Reads (GP):831 (0.01%)

Tgrandiflorum

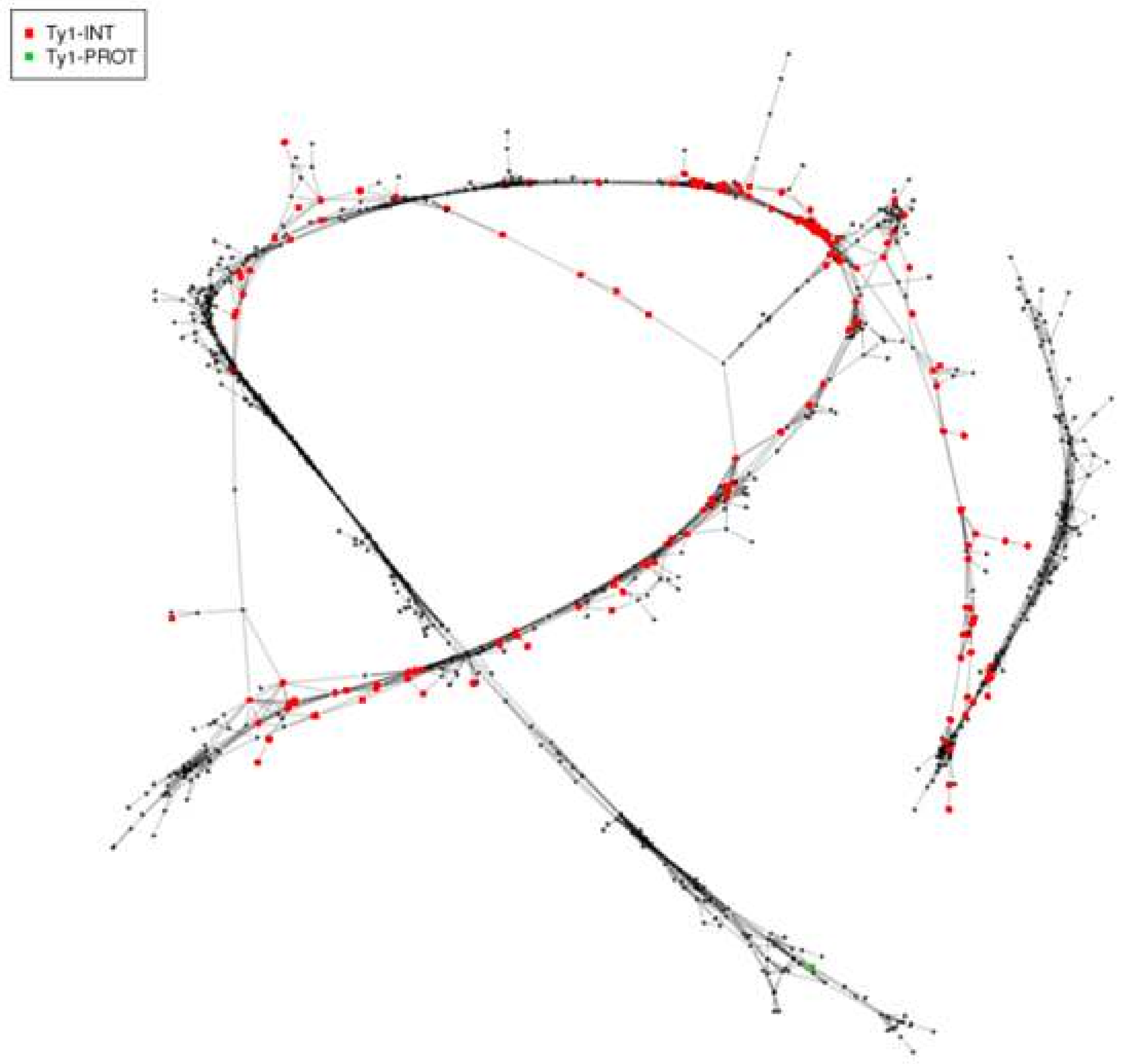

CL318
LTR_Copia
Length of Reads (GP):829 (0.01%)

Tgrandiflorum

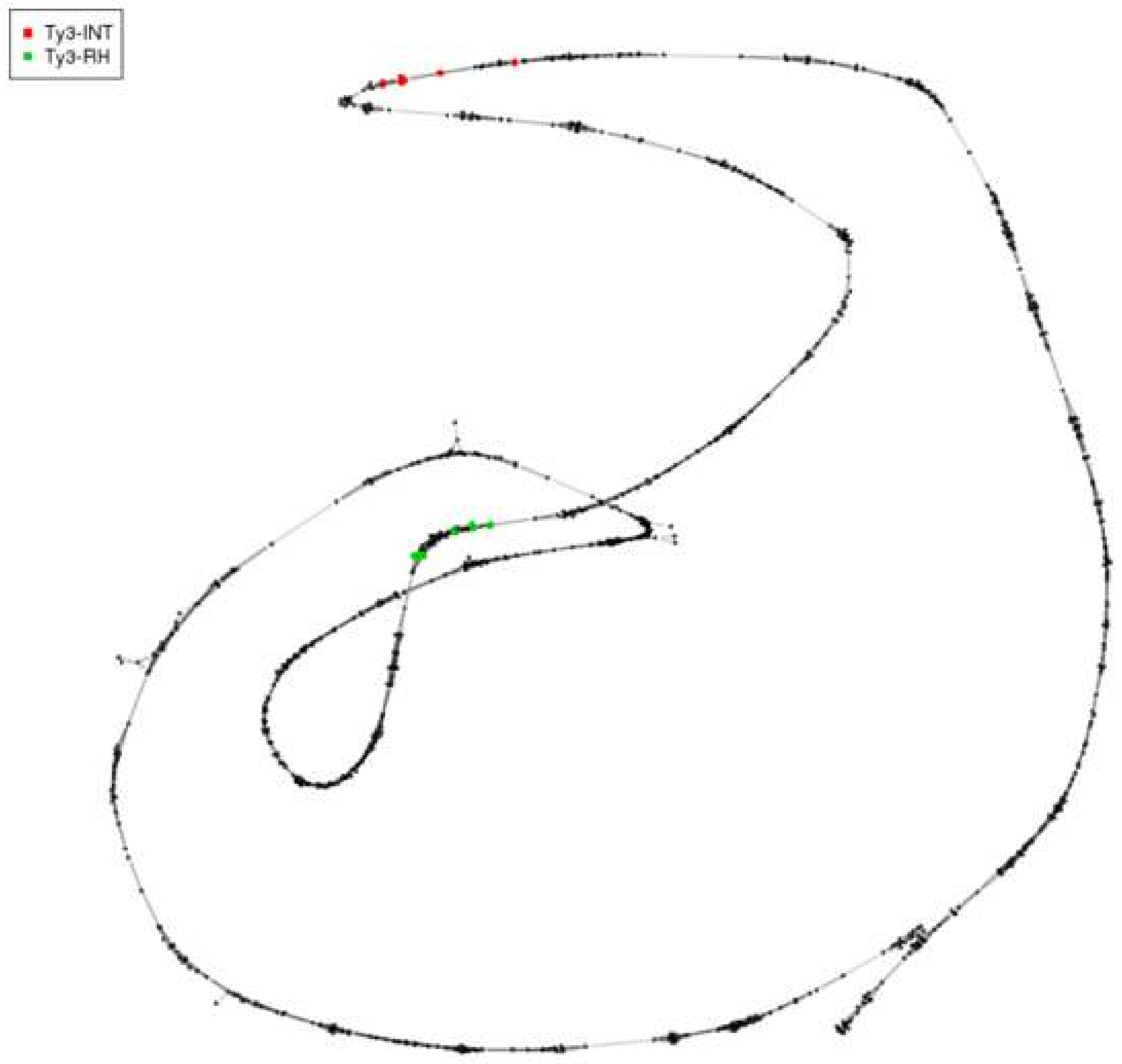

CL319
LTR_Gypsy
Length of Reads (GP):819 (0.01%)

Tgrandiflorum

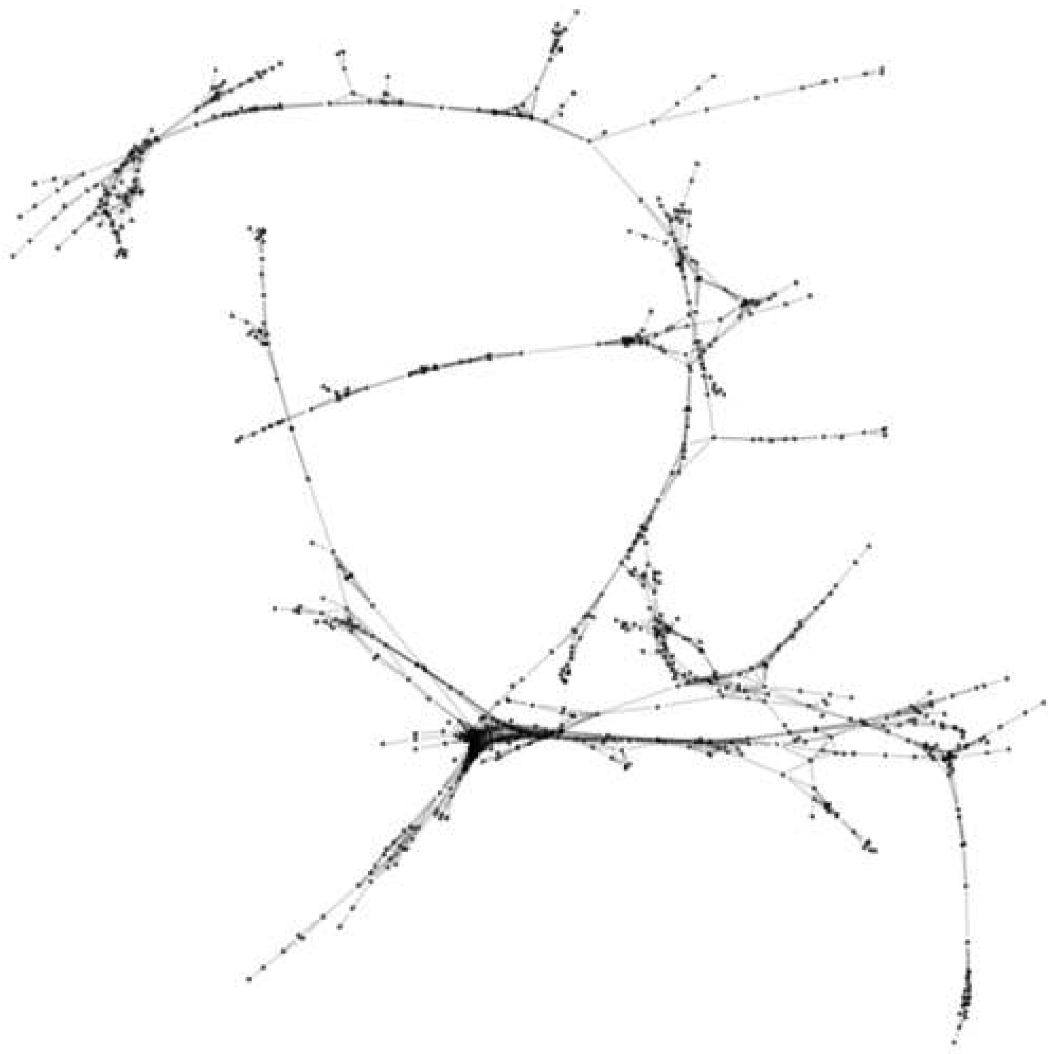

CL320
Low_complexity
Length of Reads (GP):816 (0.01%)
