# Supplementary material for: Transposon fingerprinting using low coverage whole genome shotgun sequencing in Cacao (Theobroma cacao L.) and related species
Source: BMC Genomics. 2013 Jul 24;14:502. doi: 10.1186/1471-2164-14-502 (PMC3726317; doi:10.1186/1471-2164-14-502)
Supplement: Additional file 3 — Biplots from PCAs on all of the sub-sampled datasets. Biplots from principal component analysis on every sub-sampled dataset generated, 49 in total. The abundance of each TE super-family was used as explanatory variables and the percentage of the explained variance is shown in parentheses in the legend of the x- and y-axis of the biplot. [file 1471-2164-14-502-S3.pdf]

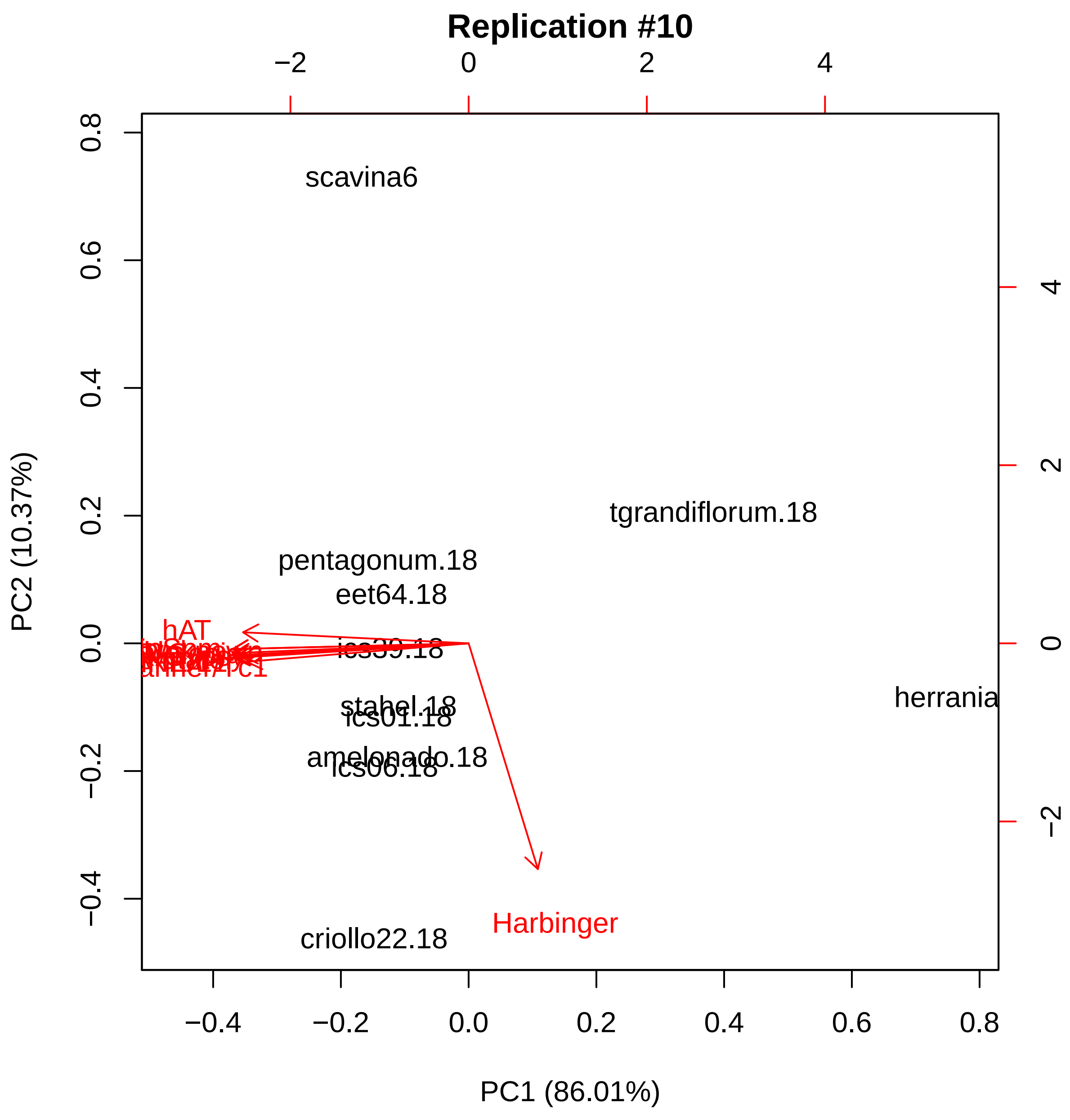

Replication #10
PC1 (86.01%)
PC2 (10.37%)
−0.4
−0.2
0.0
0.2
0.4
0.6
0.8
−2
0
2
4
scavina6
tgrandiflorum.18
pentagonum.18
eet64.18
ics39.18
stahel.18
ics01.18
herrania
amelonado.18
ics06.18
criollo22.18
hAT
Harbinger

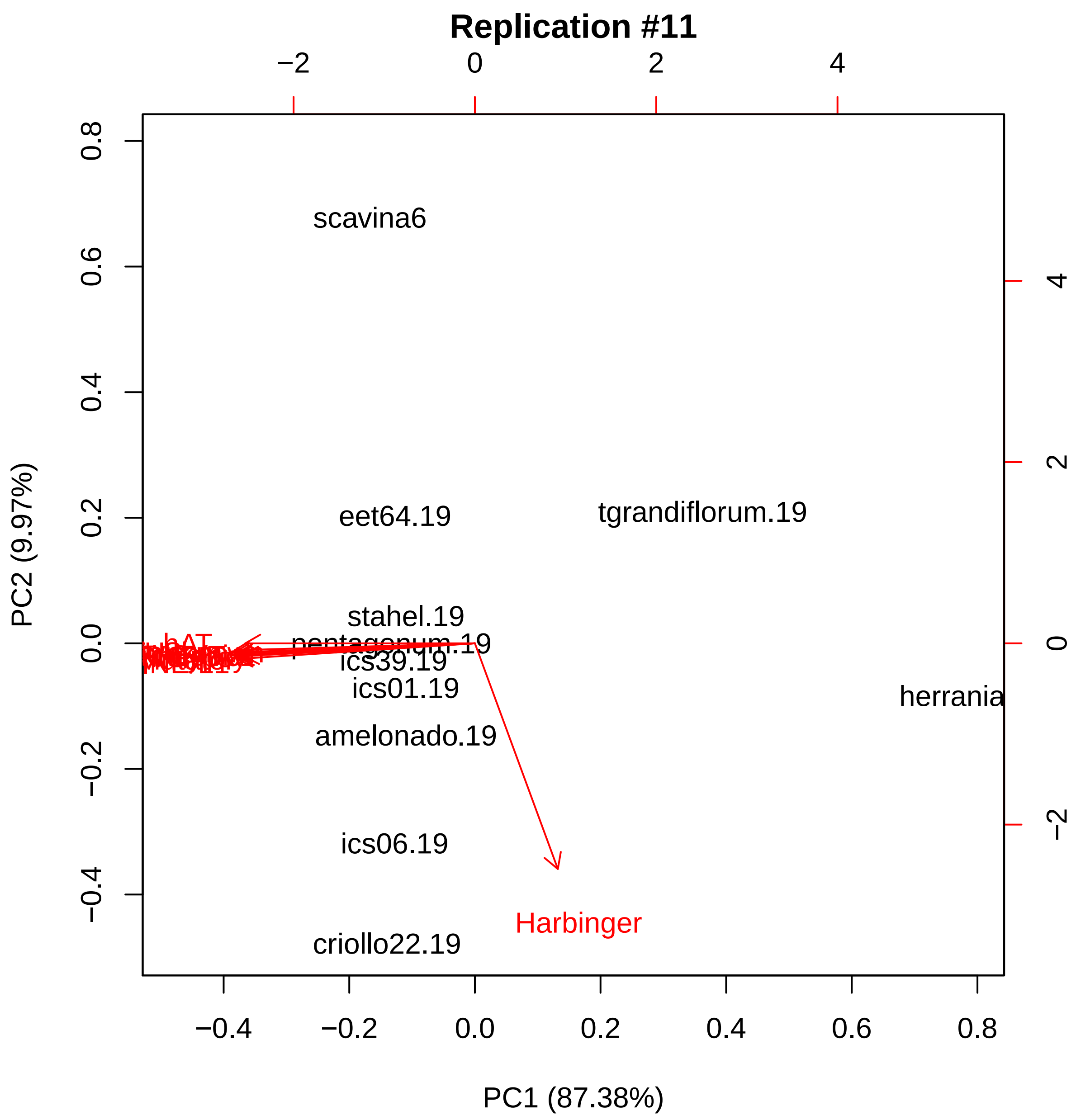
Replication #11
PC1 (87.38%)
PC2 (9.97%)
scavina6
eet64.19
tgrandiflorum.19
stahel.19
pentagonum.19
ics39.19
ics01.19
herrania
amelonado.19
ics06.19
criollo22.19
Harbinger
bAT

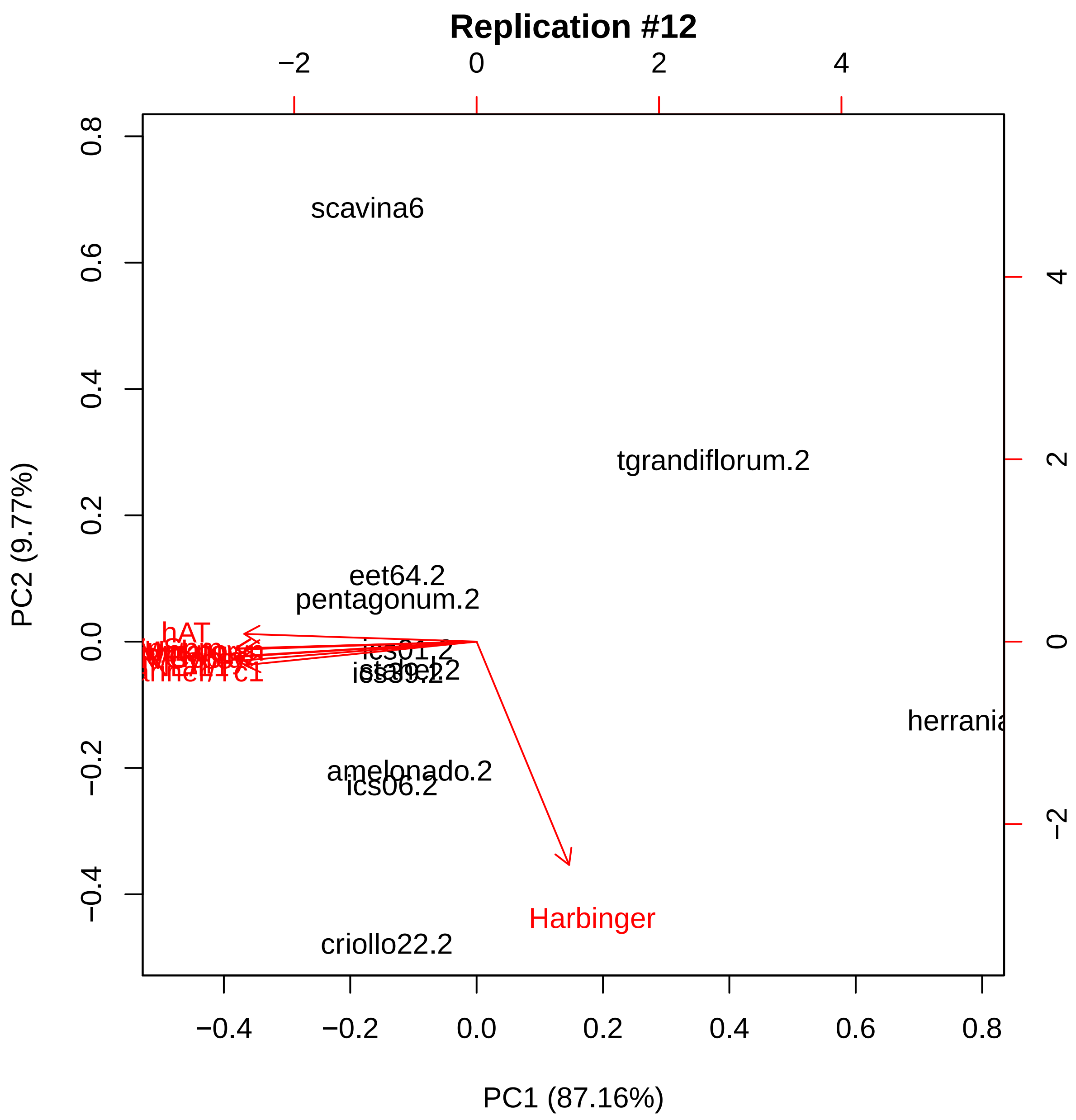
Replication #12
PC1 (87.16%)
PC2 (9.77%)
scavina6
tgrandiflorum.2
eet64.2
pentagonum.2
hAT
herrania
amelonado.2
ics06.2
criollo22.2
Harbinger

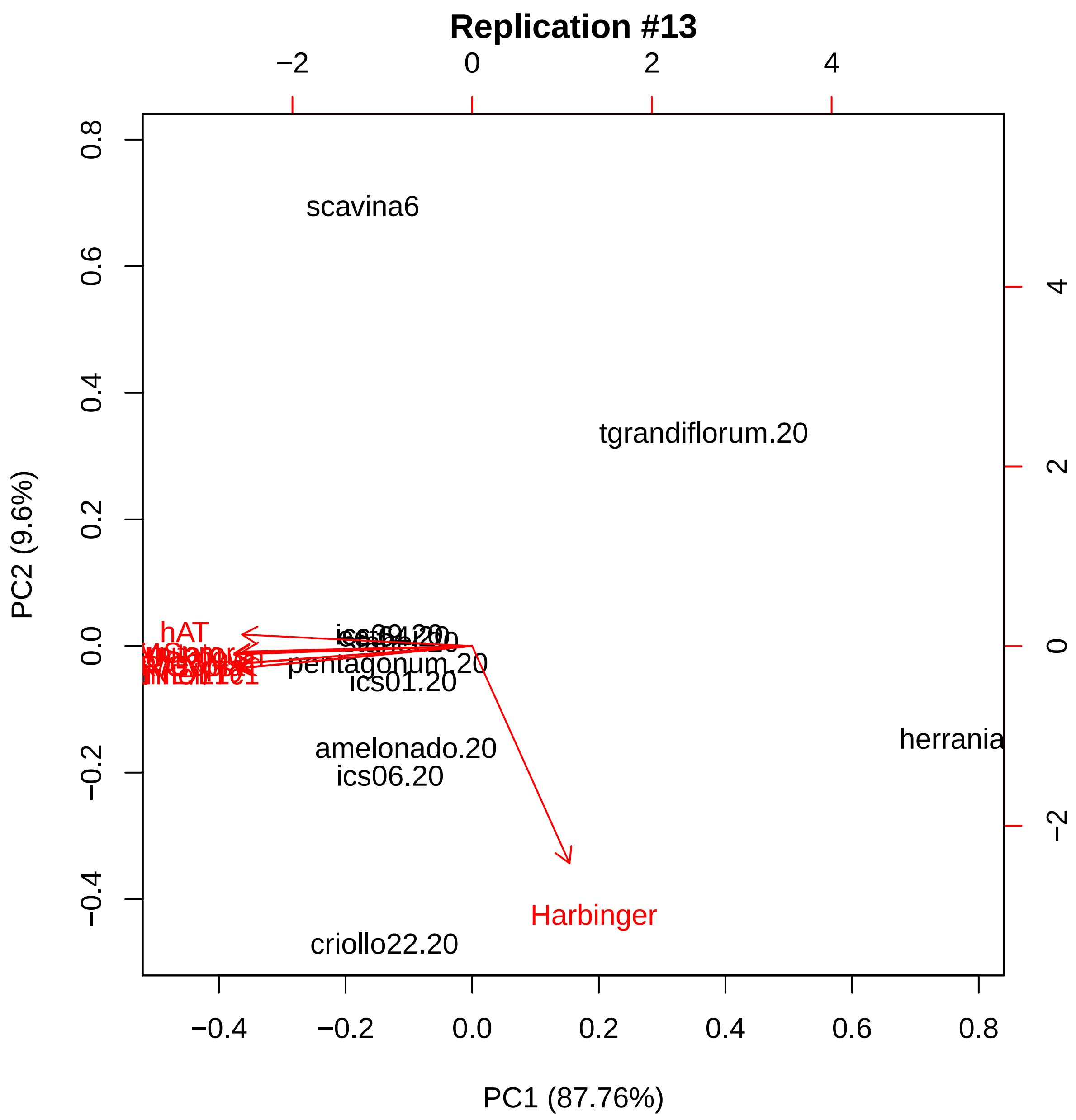
Replication #13
−2
0
2
4
0.8
0.6
0.4
0.2
0.0
−0.2
−0.4
PC2 (9.6%)
scavina6
tgrandiflorum.20
hAT
pentagonum.20
ics01.20
herrania
amelonado.20
ics06.20
Harbinger
criollo22.20
−0.4
−0.2
0.0
0.2
0.4
0.6
0.8
PC1 (87.76%)

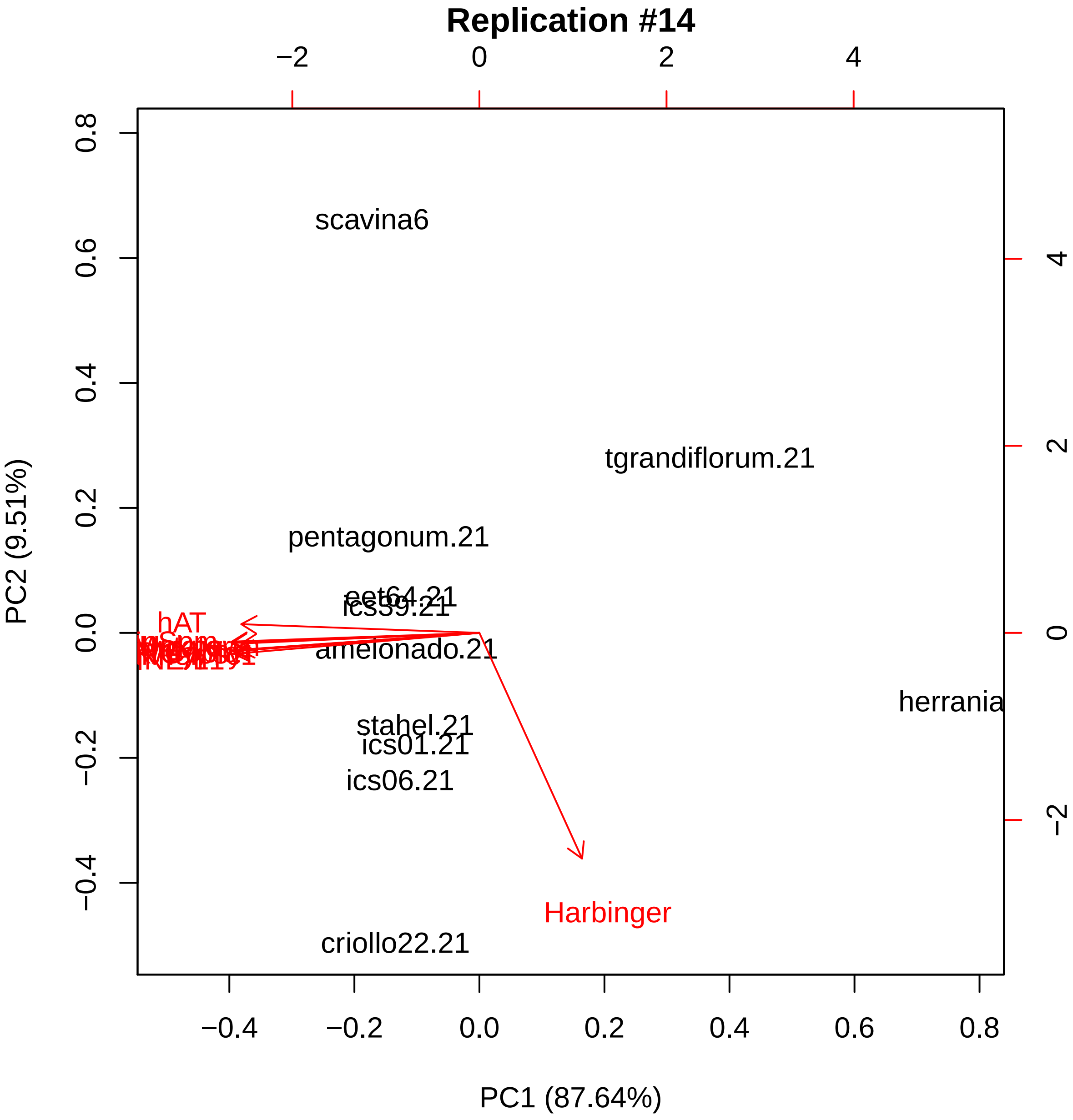
Replication #14
−2
0
2
4
0.8
0.6
0.4
0.2
0.0
−0.2
−0.4
PC2 (9.51%)
scavina6
tgrandiflorum.21
pentagonum.21
eet64.21
ics39.21
hAT
amelonado.21
herrania
stahel.21
ics01.21
ics06.21
Harbinger
criollo22.21
−0.4
−0.2
0.0
0.2
0.4
0.6
0.8
PC1 (87.64%)

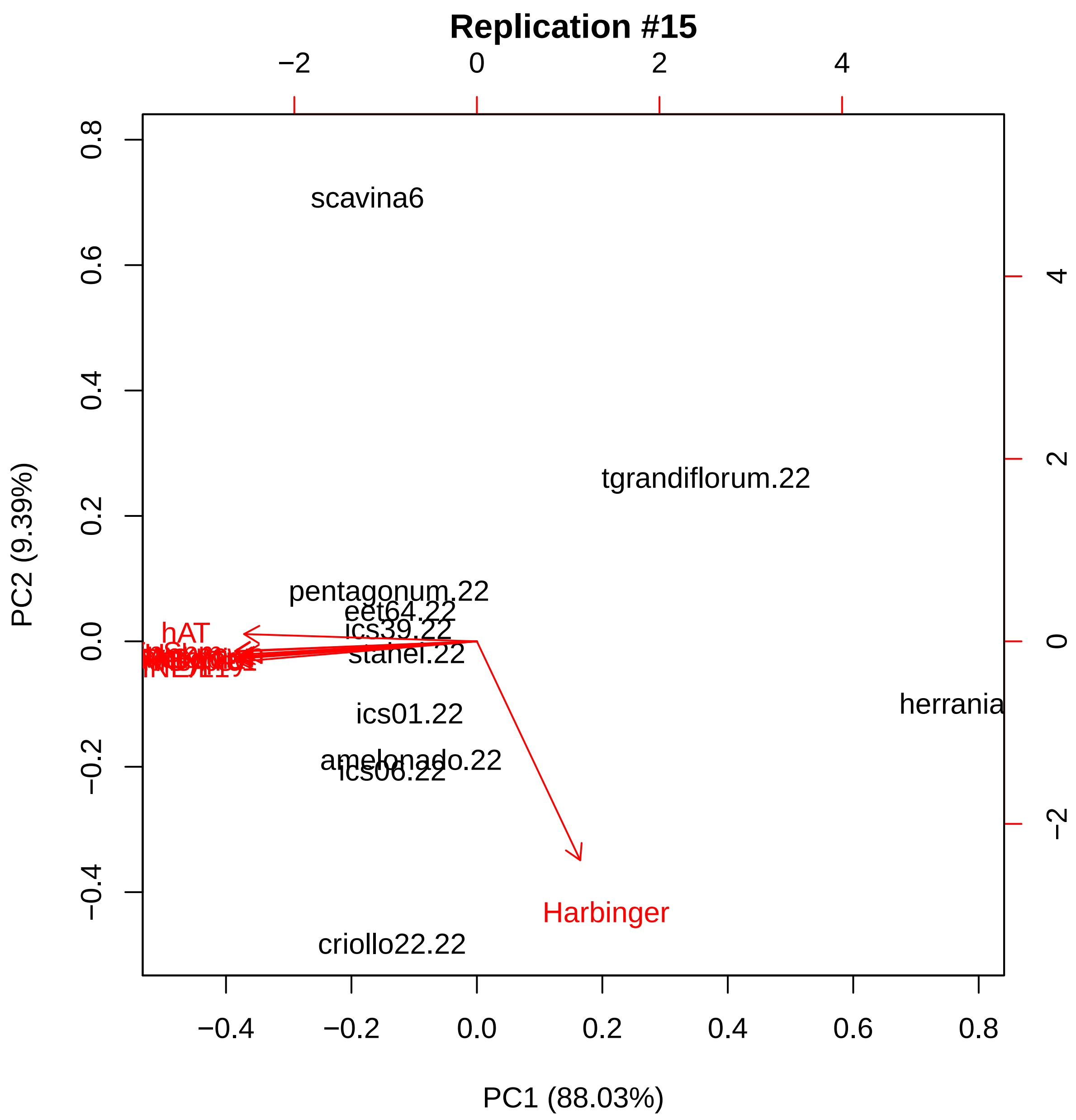
Replication #15
PC1 (88.03%)
PC2 (9.39%)
−0.4
−0.2
0.0
0.2
0.4
0.6
0.8
−2
0
2
4
scavina6
tgrandiflorum.22
pentagonum.22
eet64.22
ics39.22
stanel.22
herrania
ics01.22
amelonado.22
ics06.22
criollo22.22
hAT
Harbinger

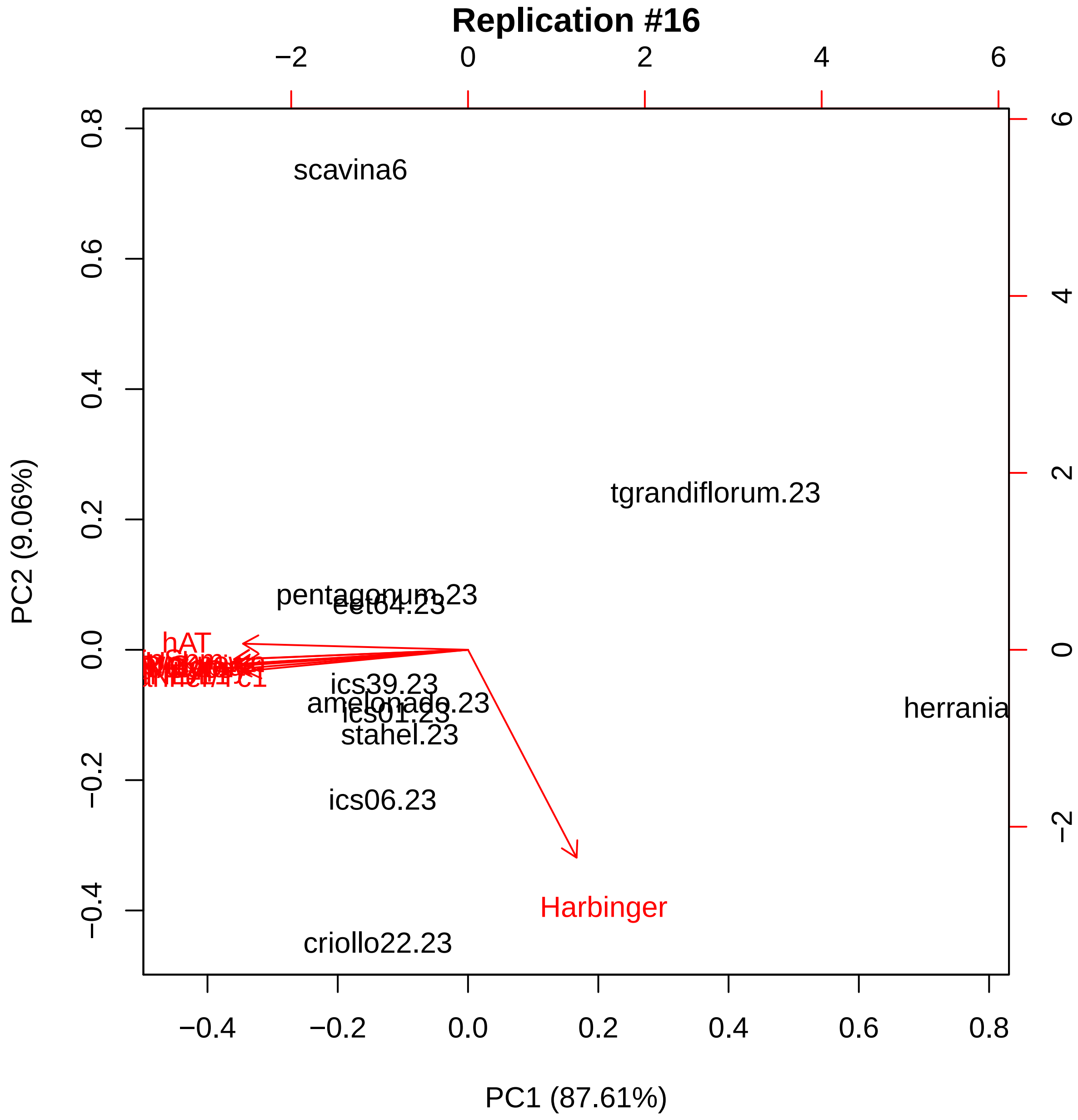
Replication #16
PC1 (87.61%)
PC2 (9.06%)
scavina6
tgrandiflorum.23
pentagonum.23
eet64.23
hAT
ics39.23
amelonado.23
ics01.23
stahel.23
ics06.23
herrania
Harbinger
criollo22.23
−0.4
−0.2
0.0
0.2
0.4
0.6
0.8
−2
0
2
4
6

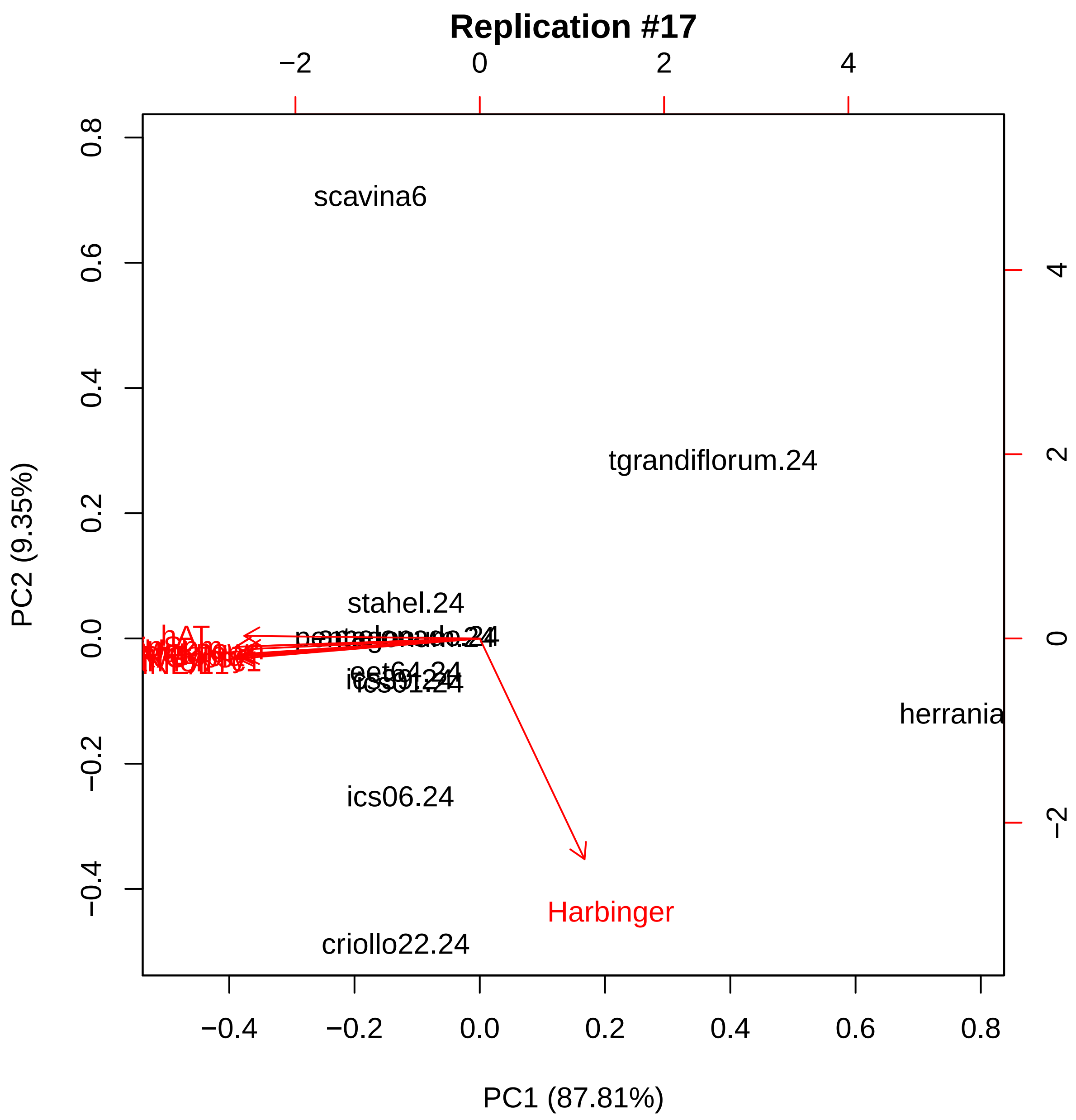
Replication #17
scavina6
tgrandiflorum.24
stahel.24
hAT
ics06.24
herrania
Harbinger
criollo22.24
PC1 (87.81%)
PC2 (9.35%)

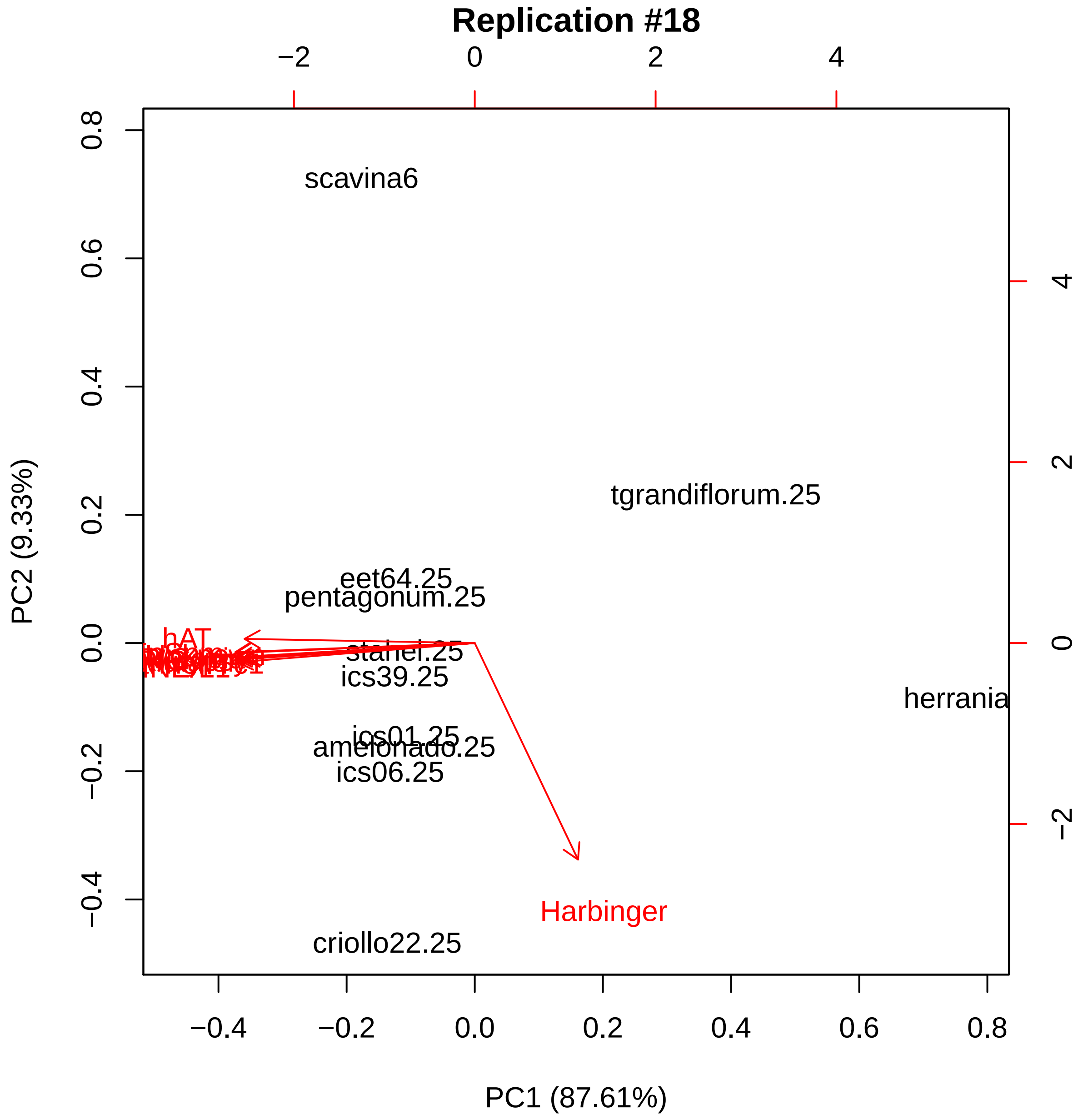
Replication #18
PC1 (87.61%)
PC2 (9.33%)
−0.4
−0.2
0.0
0.2
0.4
0.6
0.8
−2
0
2
4
scavina6
tgrandiflorum.25
eet64.25
pentagonum.25
stahel.25
ics39.25
herrania
ics01.25
amelonado.25
ics06.25
criollo22.25
hAT
Harbinger

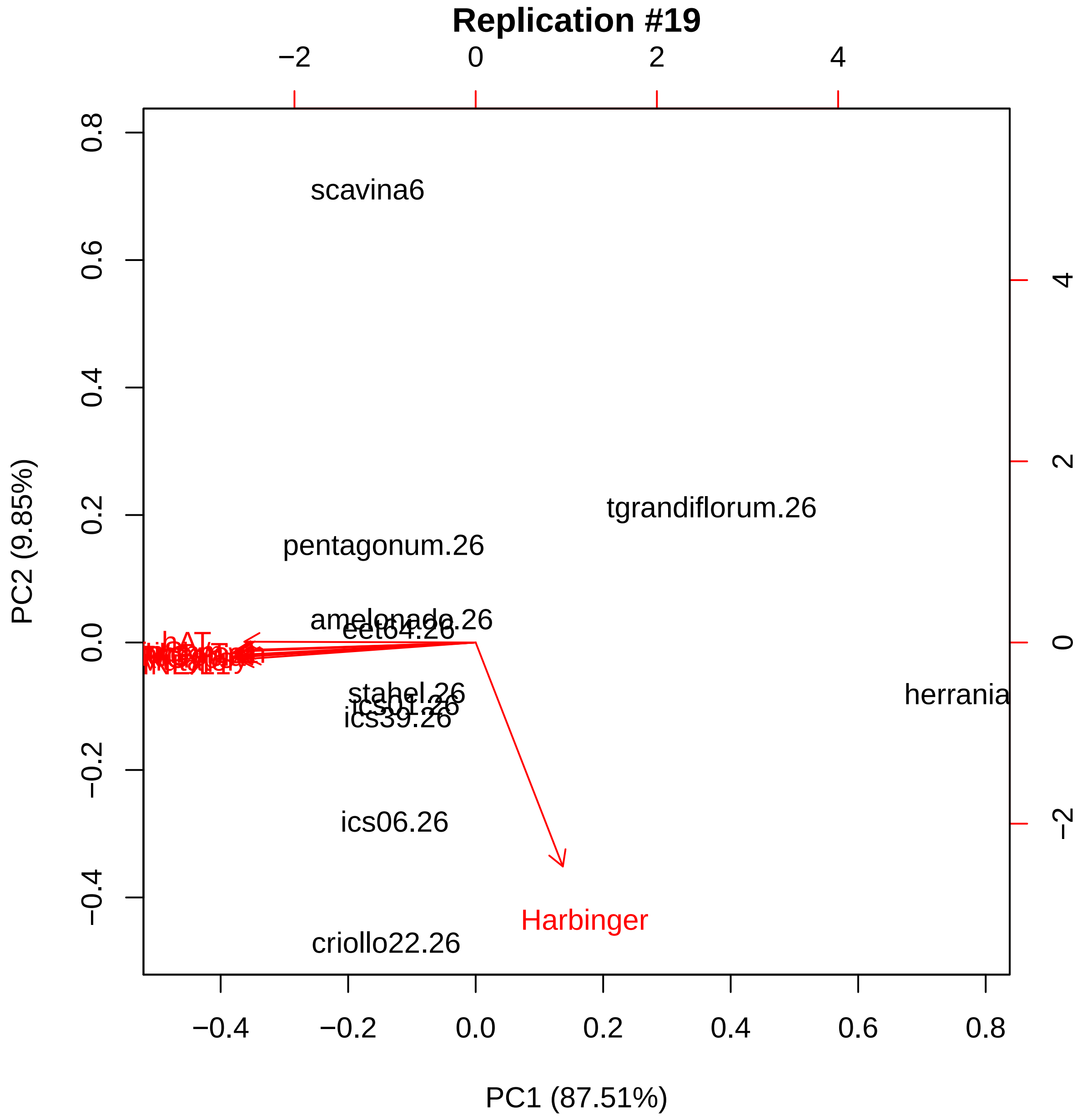
Replication #19
PC1 (87.51%)
PC2 (9.85%)
−0.4
−0.2
0.0
0.2
0.4
0.6
0.8
−2
0
2
4
scavina6
tgrandiflorum.26
pentagonum.26
amelonado.26
eet64.26
stahel.26
ics01.26
ics39.26
herrania
ics06.26
criollo22.26
Harbinger

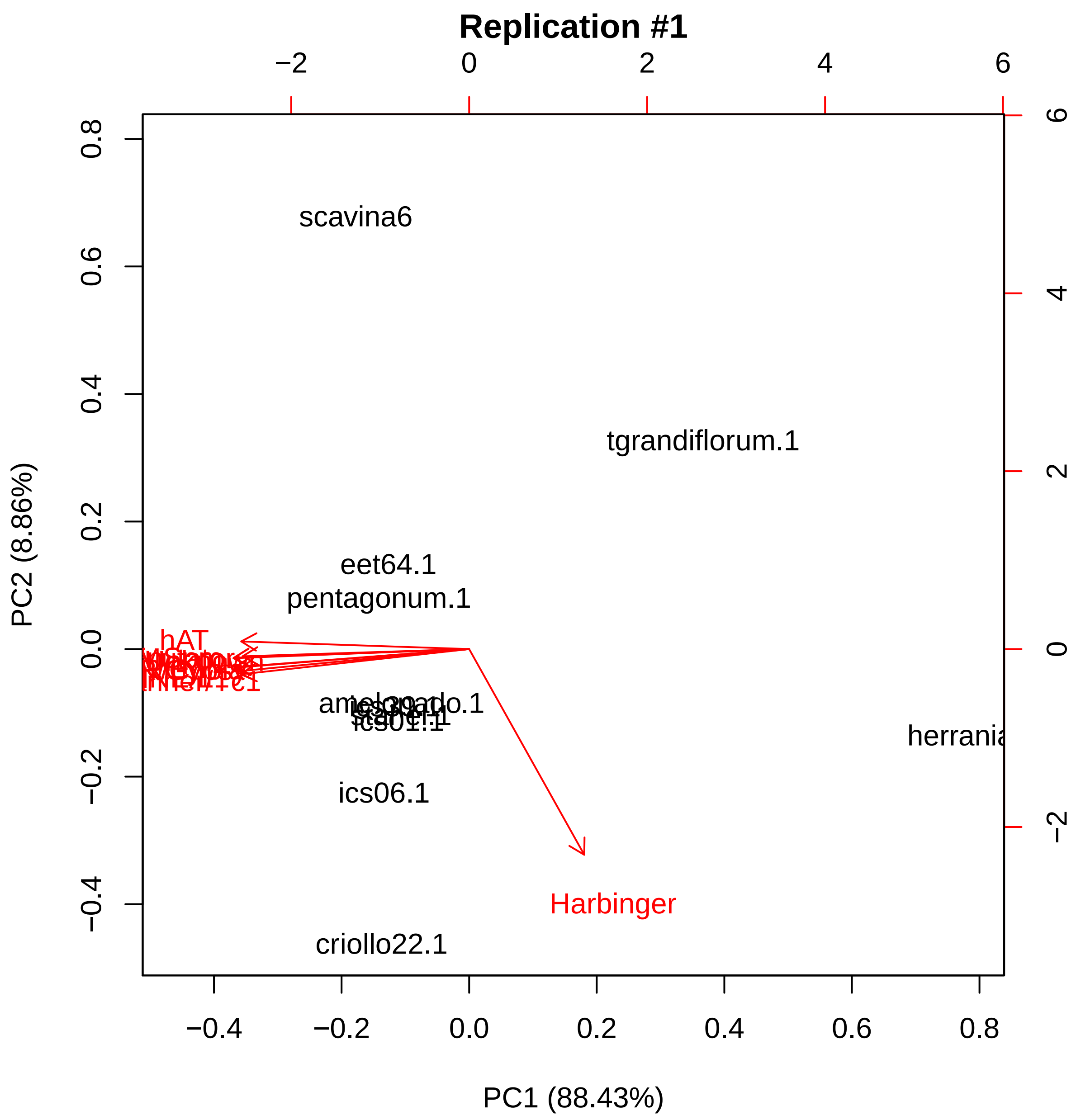

Replication #1
PC1 (88.43%)
PC2 (8.86%)
scavina6
tgrandiflorum.1
eet64.1
pentagonum.1
hAT
amelonado.1
ics06.1
herrania
Harbinger
criollo22.1
−0.4
−0.2
0.0
0.2
0.4
0.6
0.8
−2
0
2
4
6

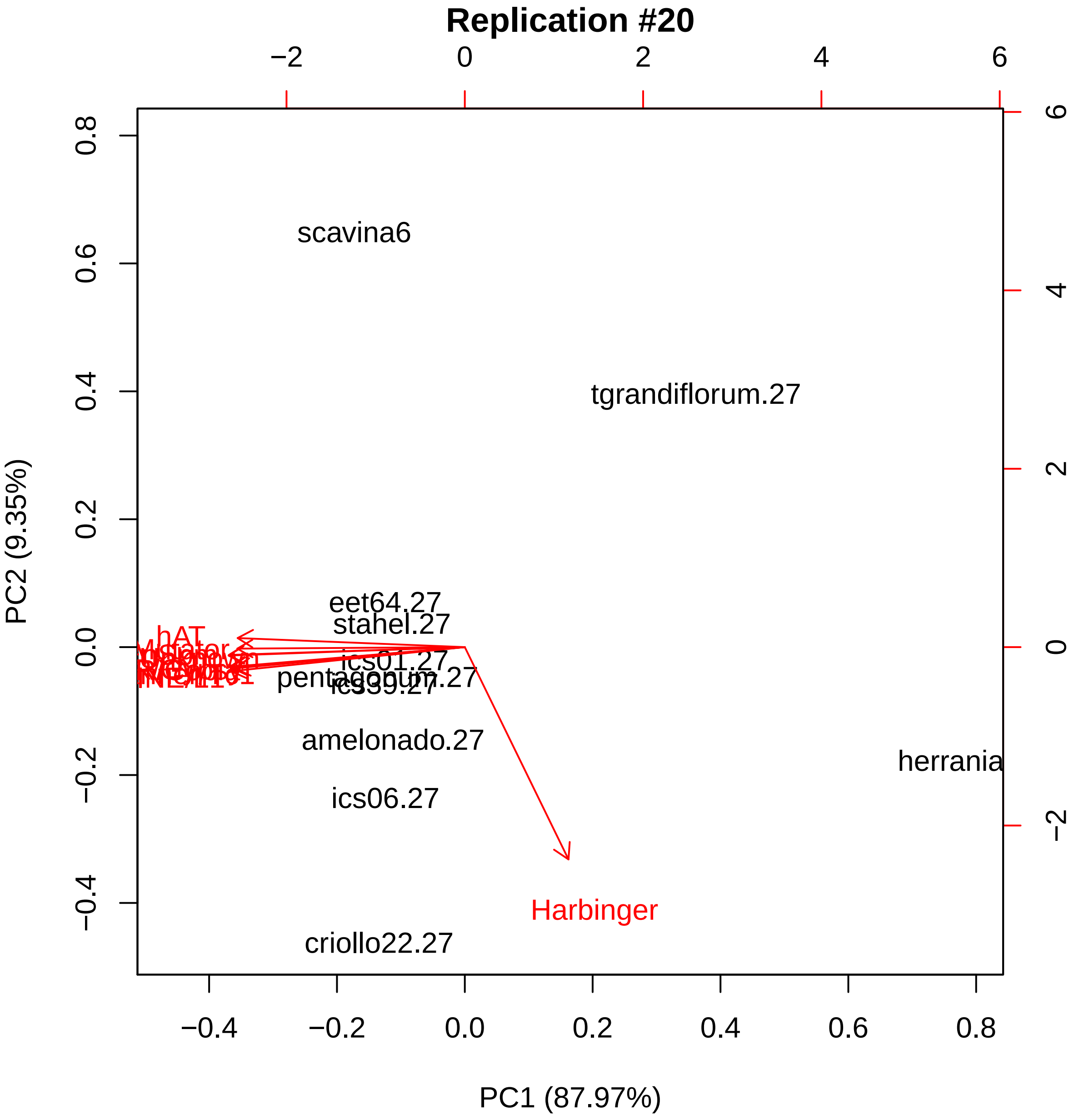

Replication #20
−2
0
2
4
6
0.8
0.6
0.4
0.2
0.0
−0.2
−0.4
PC2 (9.35%)
scavina6
tgrandiflorum.27
eet64.27
stahel.27
hAT
ics01.27
pentagonum.27
ics39.27
amelonado.27
herrania
ics06.27
Harbinger
criollo22.27
−0.4
−0.2
0.0
0.2
0.4
0.6
0.8
PC1 (87.97%)

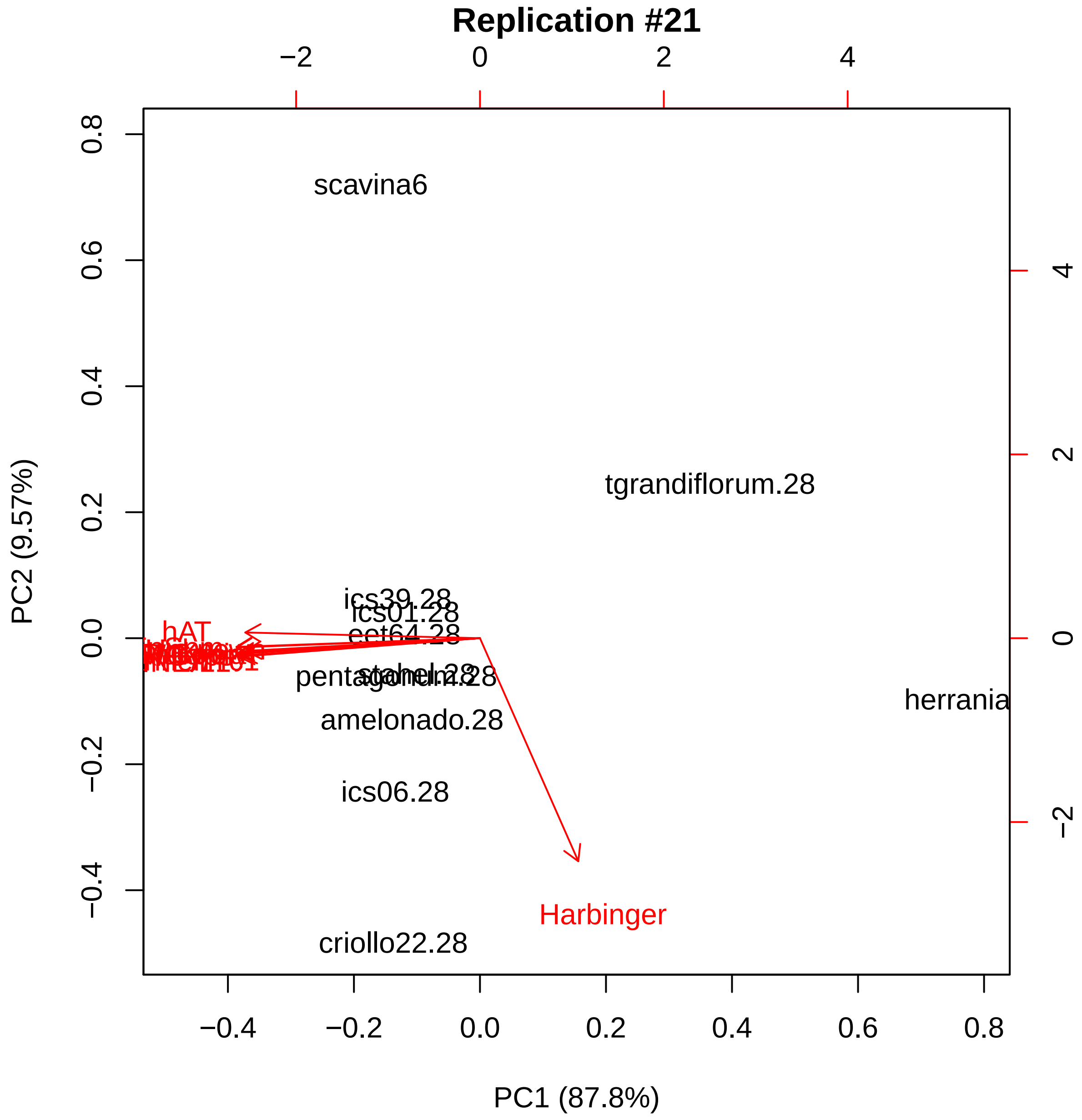
Replication #21
scavina6
tgrandiflorum.28
ics39.28
ics01.28
eet64.28
pentagonum.28
stahel.28
amelonado.28
herrania
ics06.28
criollo22.28
hAT
Harbinger
PC1 (87.8%)
PC2 (9.57%)

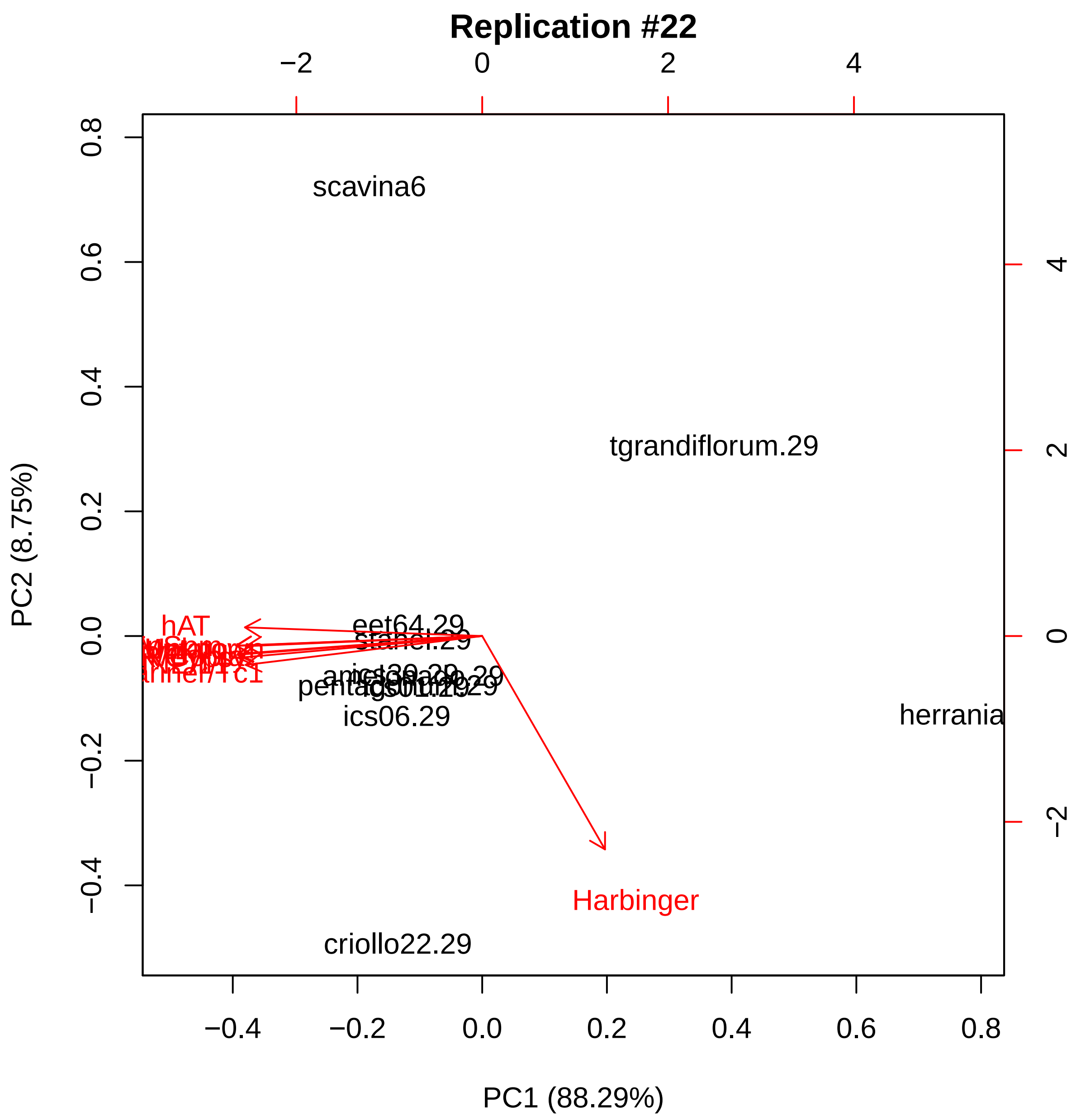

Replication #22
PC1 (88.29%)
PC2 (8.75%)
scavina6
tgrandiflorum.29
eet64.29
stahel.29
ics06.29
herrania
criollo22.29
hAT
Harbinger
−0.4
−0.2
0.0
0.2
0.4
0.6
0.8
−2
0
2
4

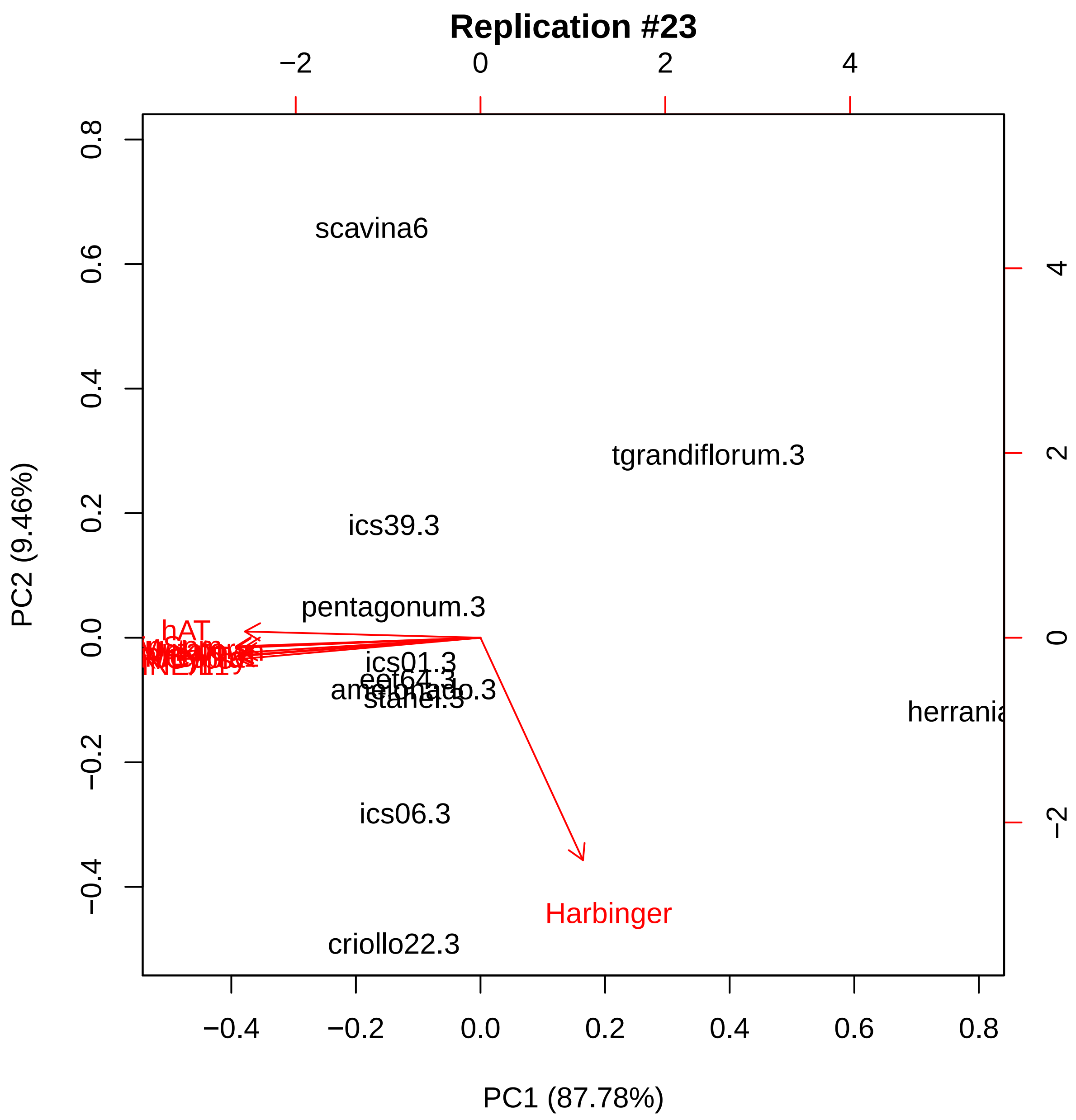

Replication #23
−2
0
2
4
0.8
0.6
0.4
0.2
0.0
−0.2
−0.4
PC2 (9.46%)
4
2
0
−2
scavina6
tgrandiflorum.3
ics39.3
pentagonum.3
hAT
ics01.3
eet64.3
amelonado.3
stahel.3
herrania
ics06.3
Harbinger
criollo22.3
−0.4
−0.2
0.0
0.2
0.4
0.6
0.8
PC1 (87.78%)

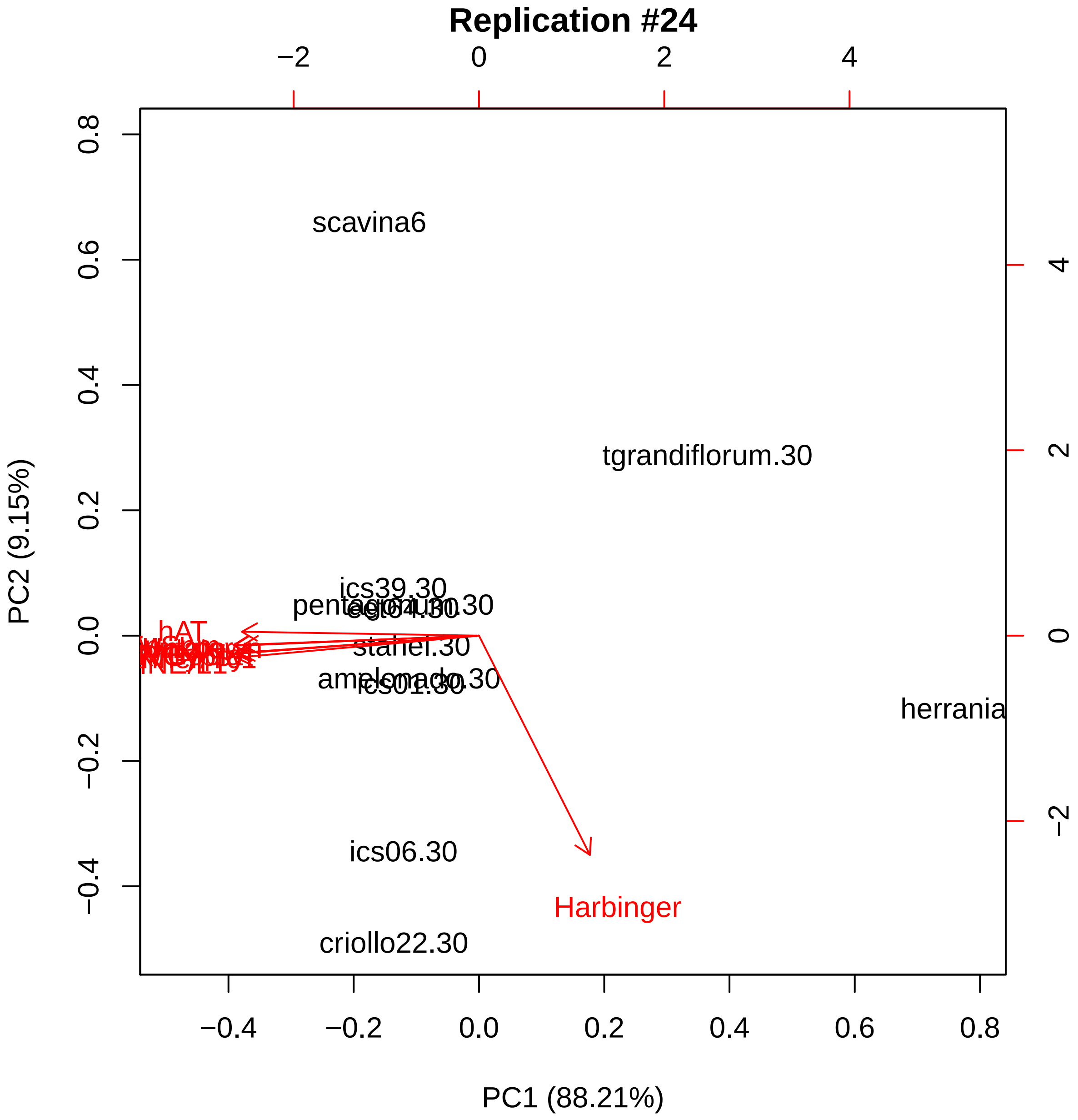
Replication #24
PC1 (88.21%)
PC2 (9.15%)
−0.4
−0.2
0.0
0.2
0.4
0.6
0.8
−2
0
2
4
scavina6
tgrandiflorum.30
ics39.30
stahel.30
herrania
ics06.30
Harbinger
criollo22.30
hAT

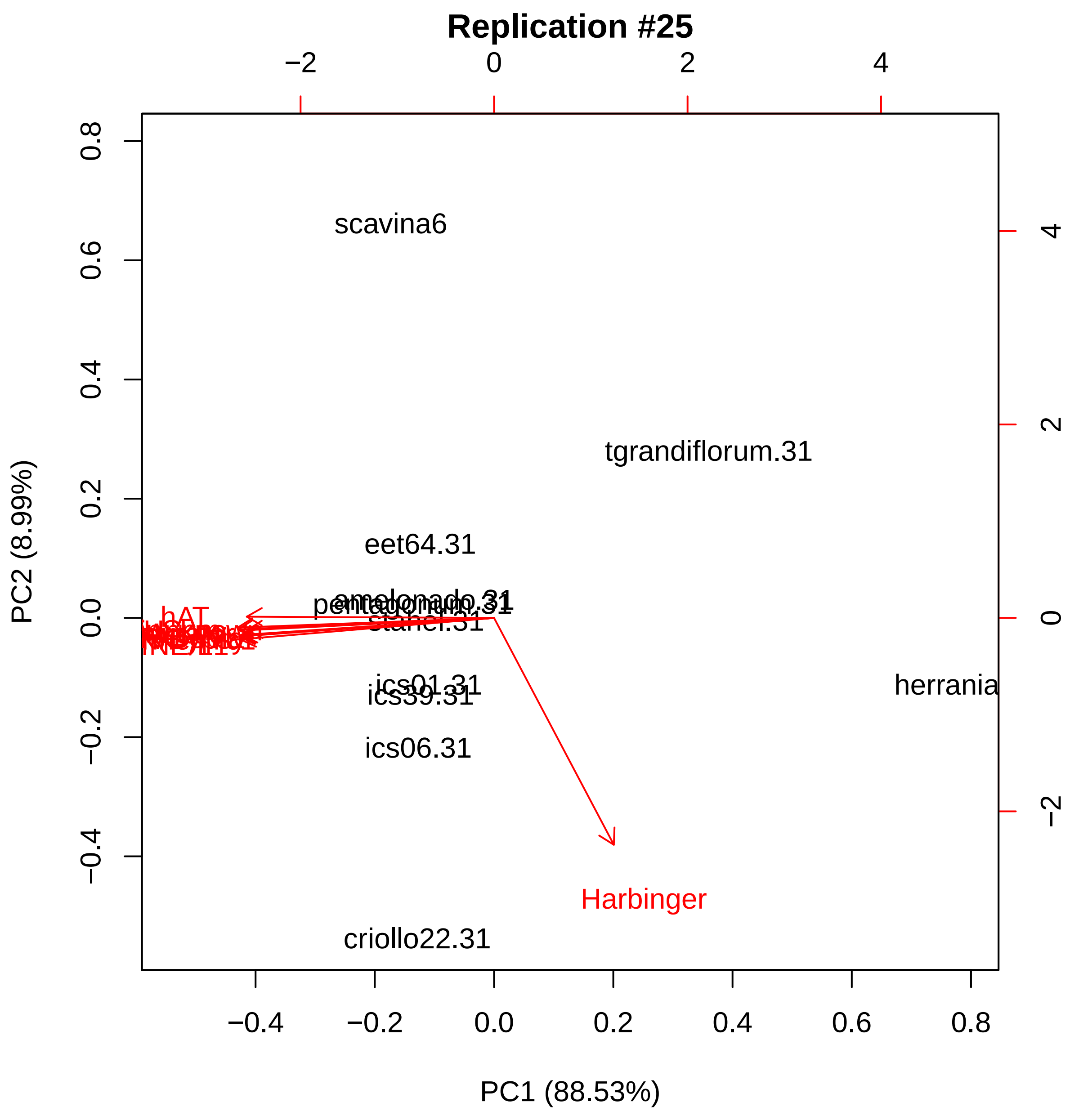

Replication #25
PC1 (88.53%)
PC2 (8.99%)
scavina6
tgrandiflorum.31
eet64.31
amelonado.31
pentagonum.31
stahel.31
ics01.31
ics39.31
ics06.31
herrania
criollo22.31
hAT
Harbinger
−2
0
2
4
−0.4
−0.2
0.0
0.2
0.4
0.6
0.8

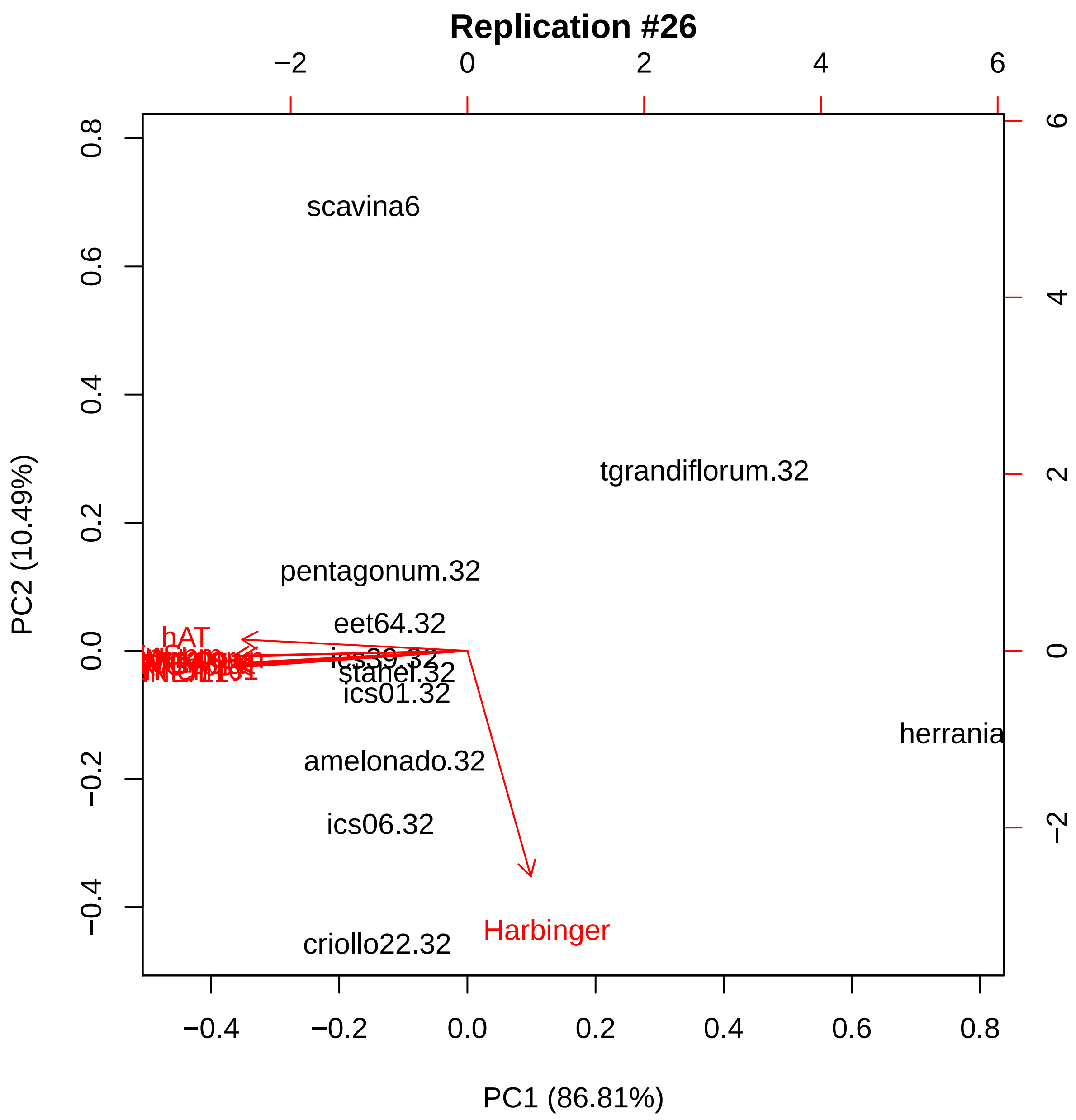
Replication #26
PC1 (86.81%)
PC2 (10.49%)
−0.4
−0.2
0.0
0.2
0.4
0.6
0.8
−2
0
2
4
6
scavina6
tgrandiflorum.32
pentagonum.32
eet64.32
stahel.32
ics01.32
herrania
amelonado.32
ics06.32
criollo22.32
hAT
Harbinger

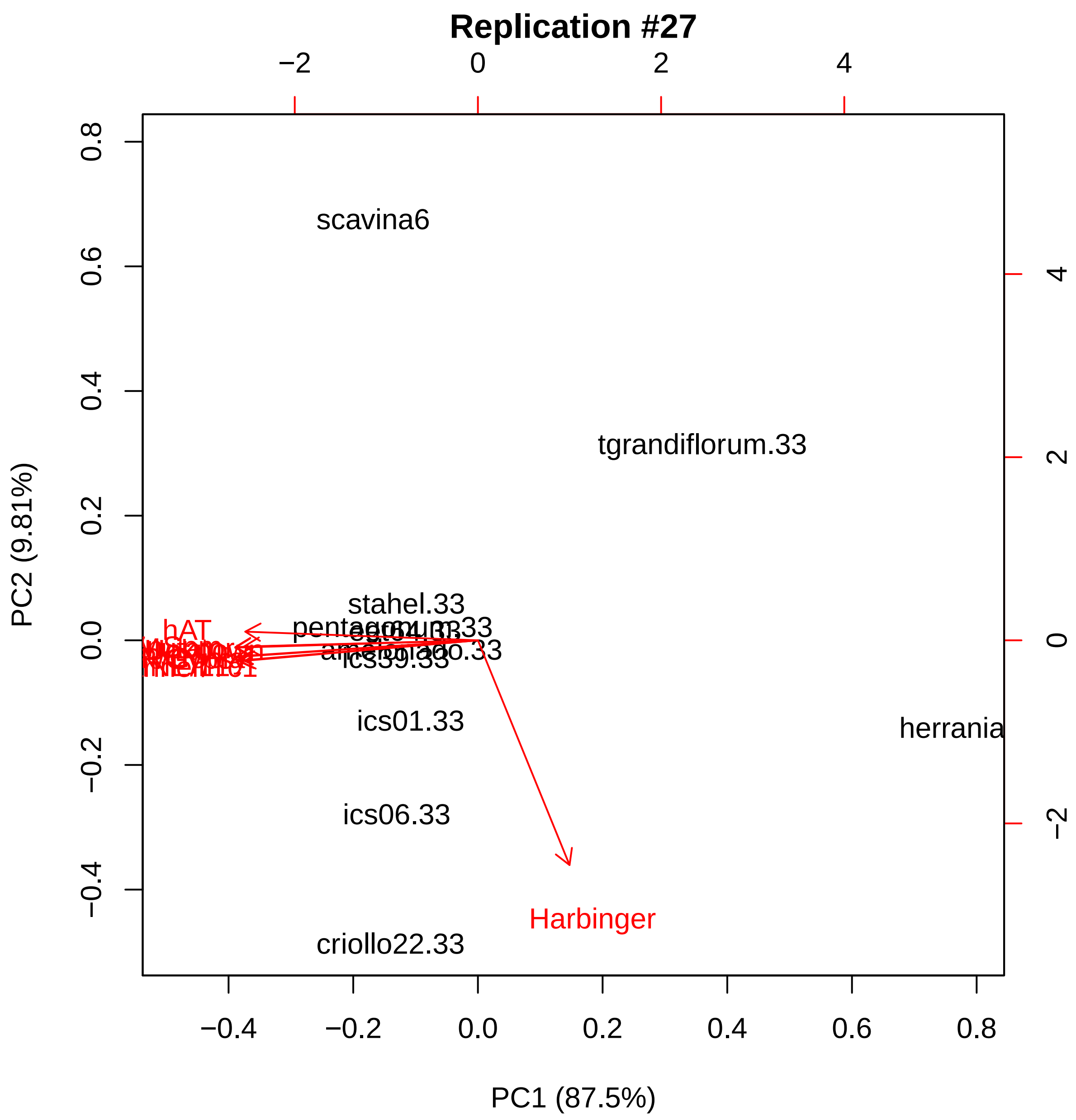

Replication #27
PC1 (87.5%)
PC2 (9.81%)
scavina6
tgrandiflorum.33
stahel.33
pentagonum.33
amelonado.33
ics39.33
ics01.33
ics06.33
criollo22.33
herrania
hAT
Harbinger
−0.4
−0.2
0.0
0.2
0.4
0.6
0.8
−2
0
2
4

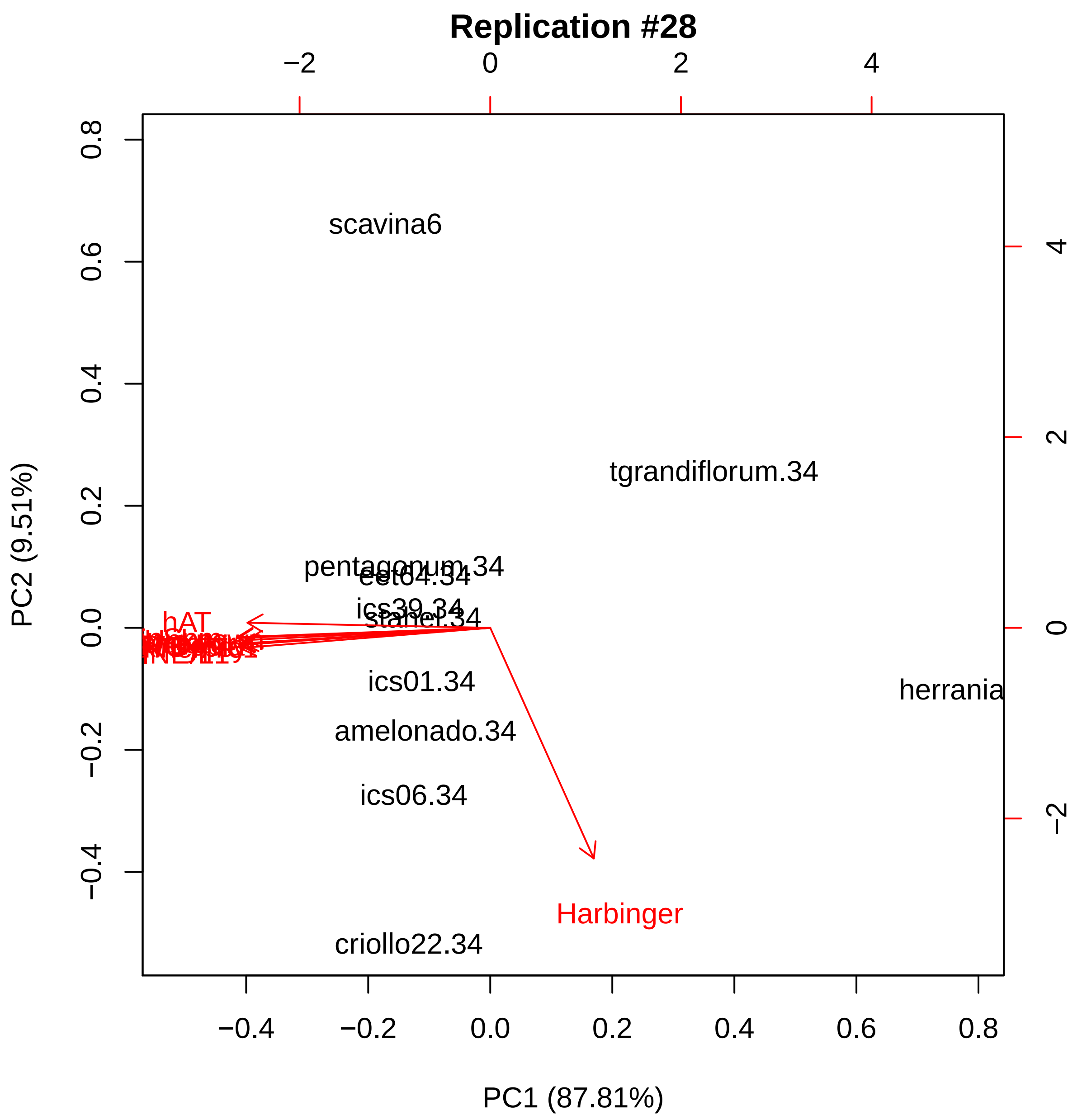
Replication #28
PC1 (87.81%)
PC2 (9.51%)
scavina6
tgrandiflorum.34
pentagonum.34
eet64.34
ics39.34
stahel.34
ics01.34
amelonado.34
ics06.34
criollo22.34
herrania
hAT
Harbinger

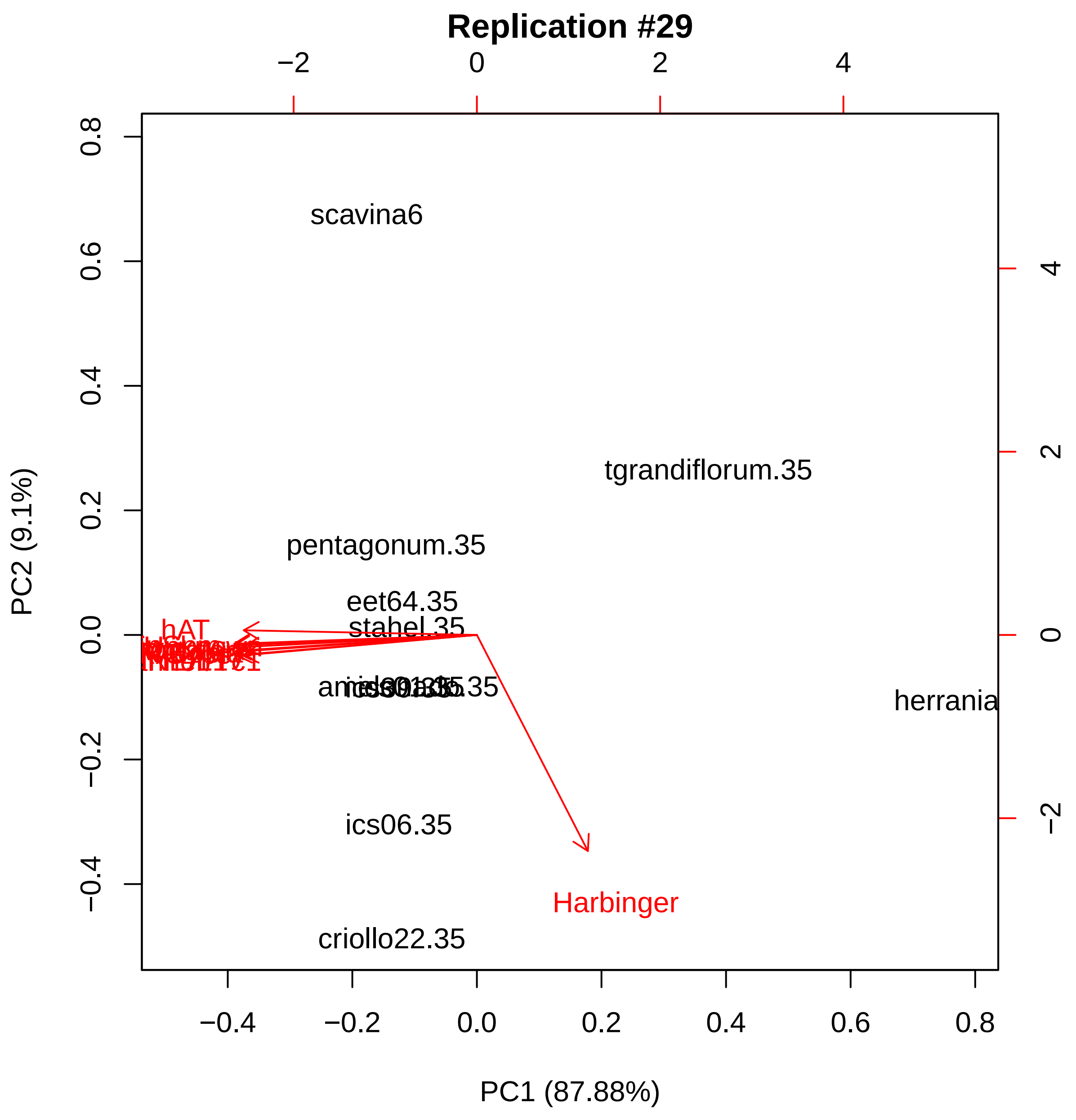
Replication #29
PC1 (87.88%)
PC2 (9.1%)
scavina6
tgrandiflorum.35
pentagonum.35
eet64.35
stahel.35
herrania
ics06.35
criollo22.35
hAT
Harbinger
−0.4
−0.2
0.0
0.2
0.4
0.6
0.8
−2
0
2
4

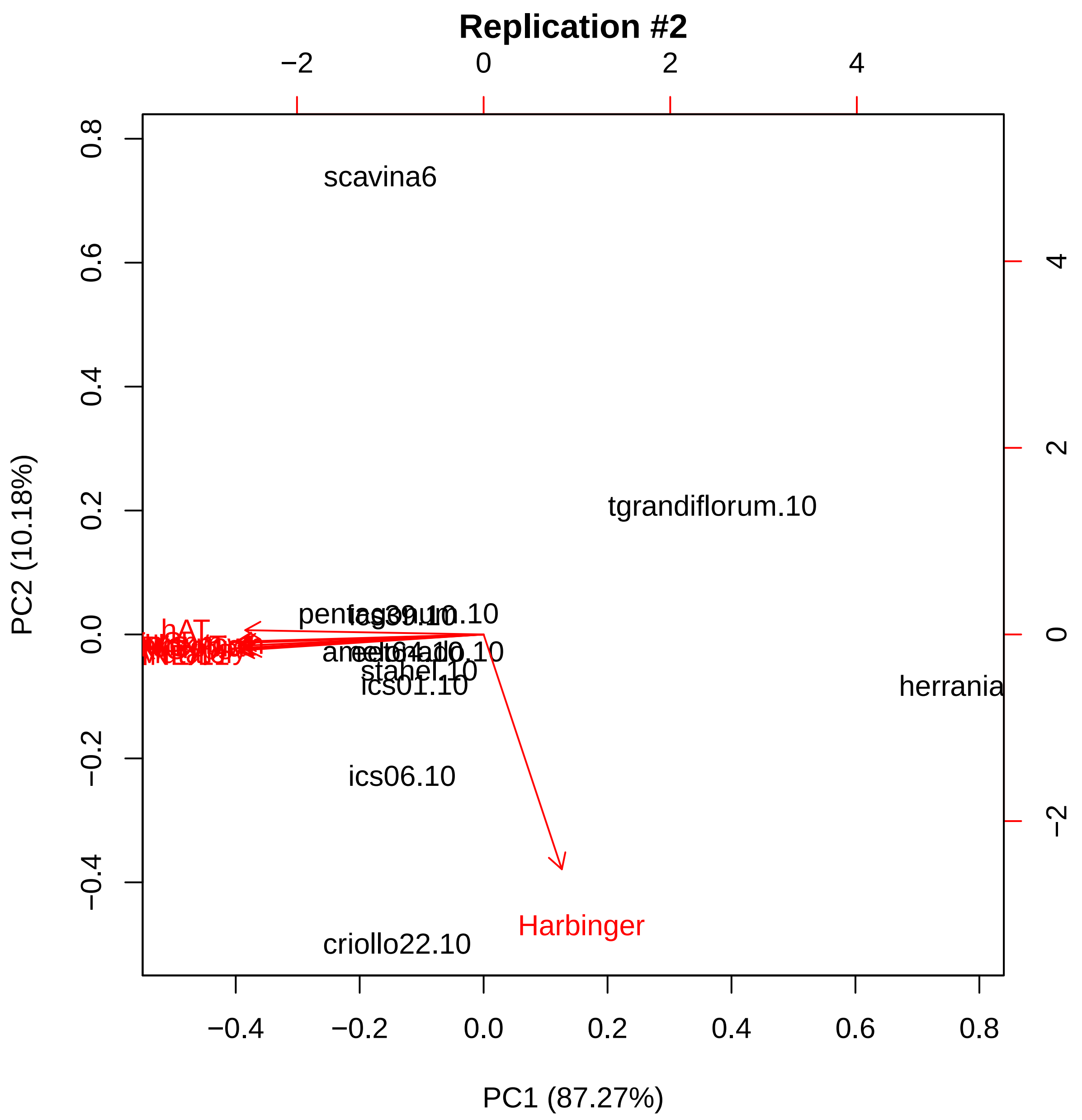

Replication #2
PC1 (87.27%)
PC2 (10.18%)
scavina6
tgrandiflorum.10
herrania
ics06.10
criollo22.10
stahel.10
ics01.10
Harbinger
hAT

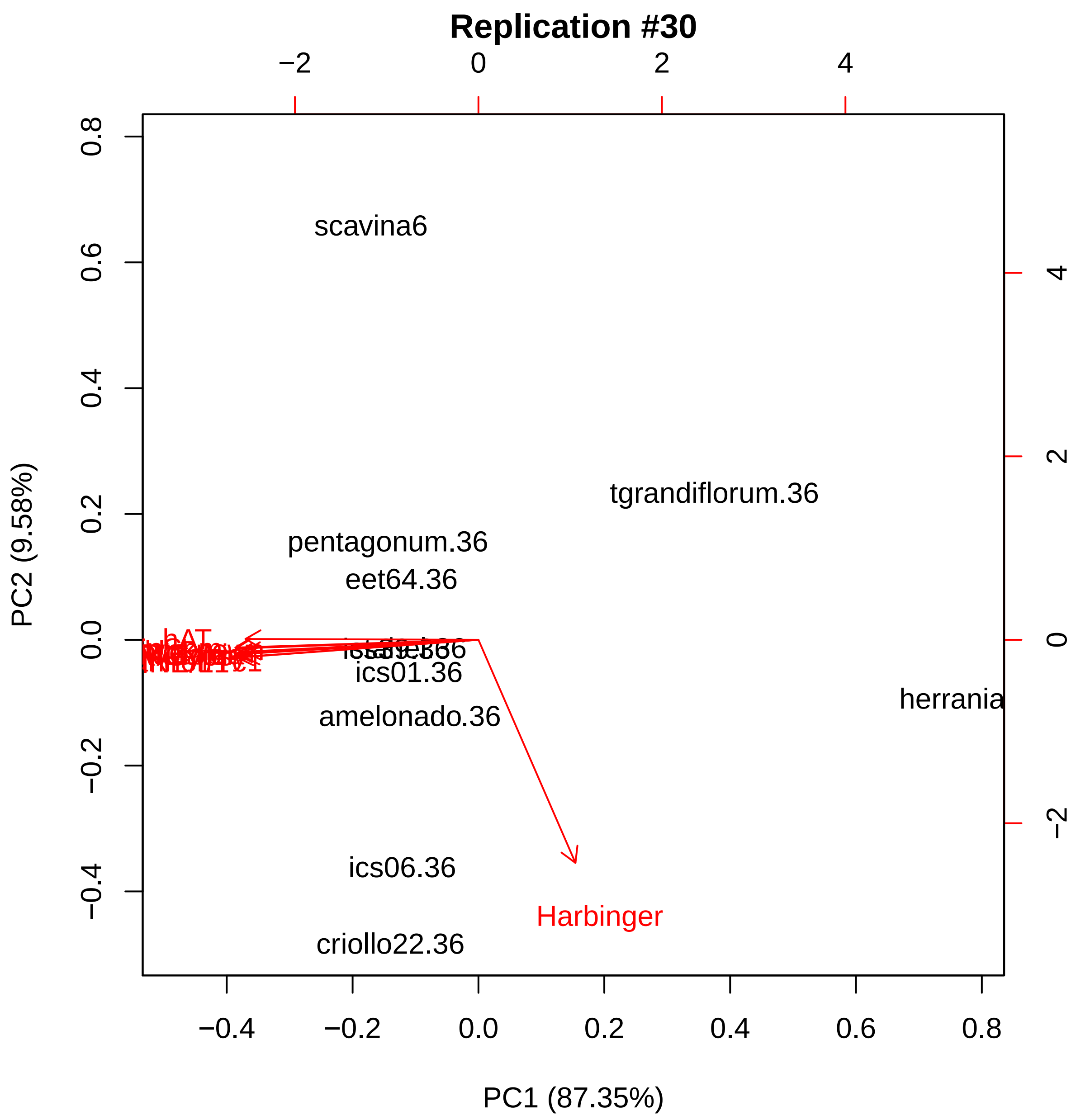
Replication #30
PC1 (87.35%)
PC2 (9.58%)
−2
0
2
4
−0.4
−0.2
0.0
0.2
0.4
0.6
0.8
scavina6
tgrandiflorum.36
pentagonum.36
eet64.36
ics01.36
amelonado.36
herrania
ics06.36
criollo22.36
Harbinger

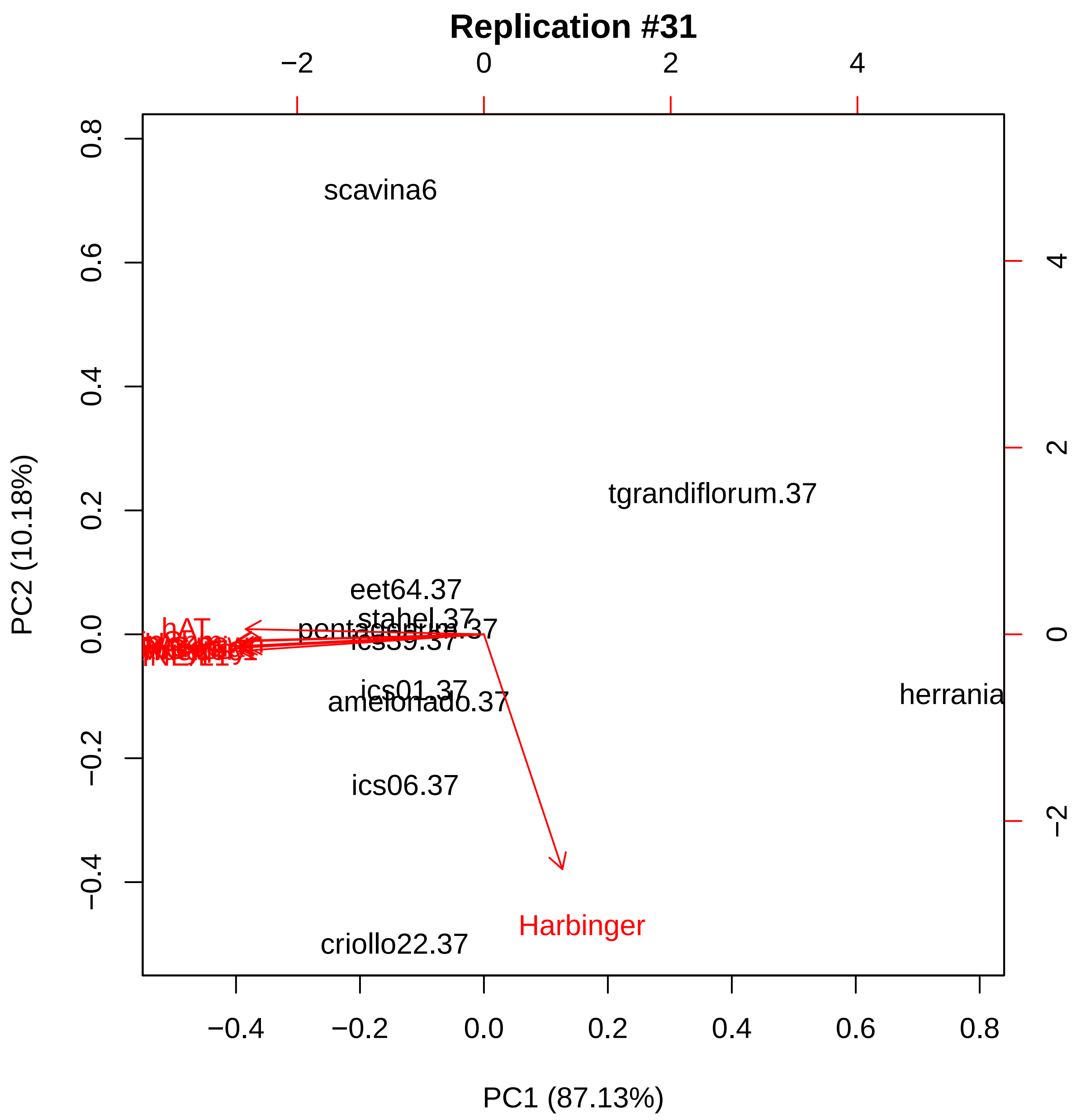

Replication #31
scavina6
tgrandiflorum.37
eet64.37
stahel.37
pentagonum.37
ics39.37
ics01.37
amelonado.37
herrania
ics06.37
criollo22.37
Harbinger
hAT
PC1 (87.13%)
PC2 (10.18%)
−0.4
−0.2
0.0
0.2
0.4
0.6
0.8
−2
0
2
4

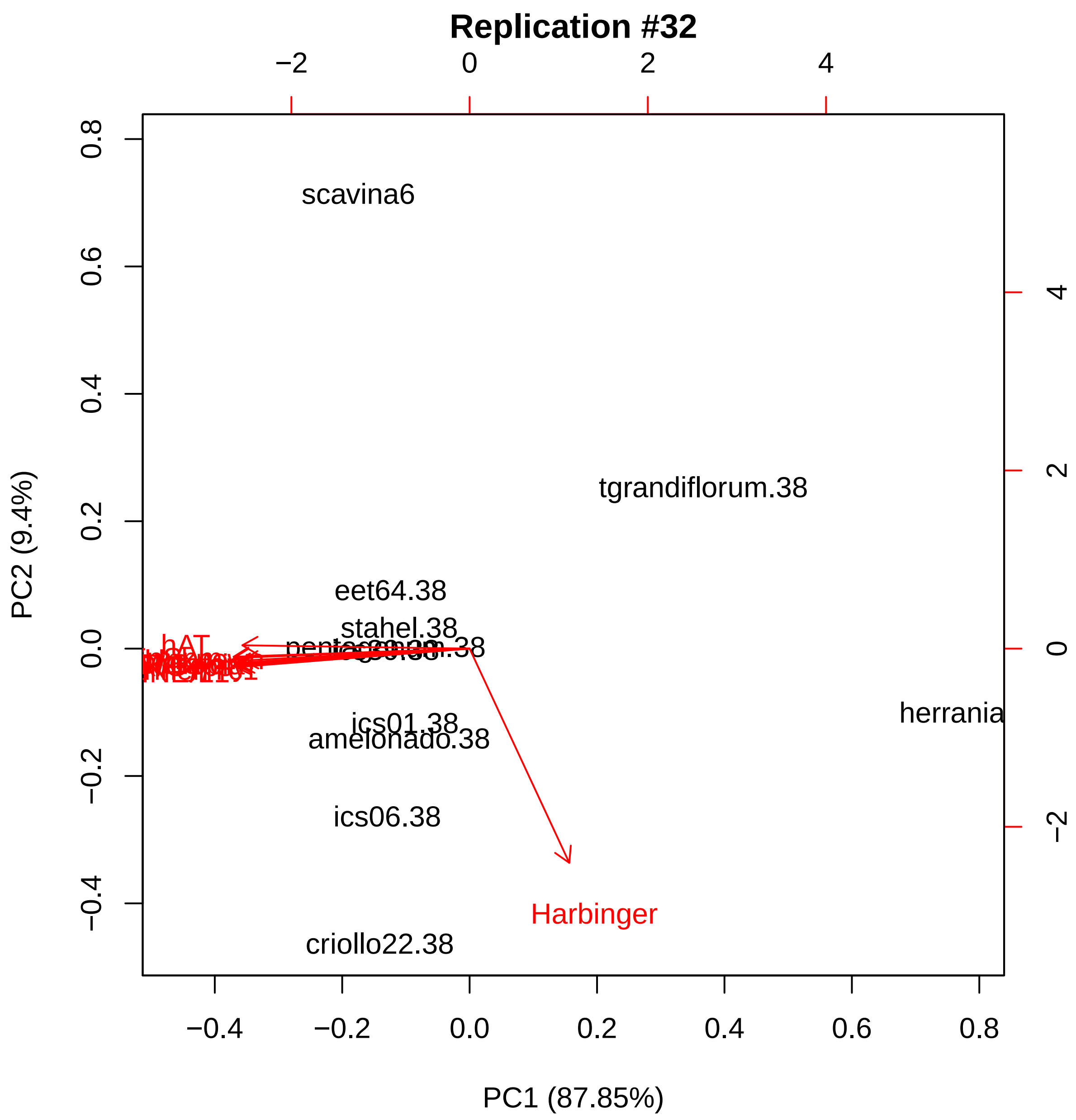
Replication #32
scavina6
tgrandiflorum.38
eet64.38
stahel.38
ics01.38
amelonado.38
ics06.38
herrania
criollo22.38
hAT
Harbinger
PC1 (87.85%)
PC2 (9.4%)

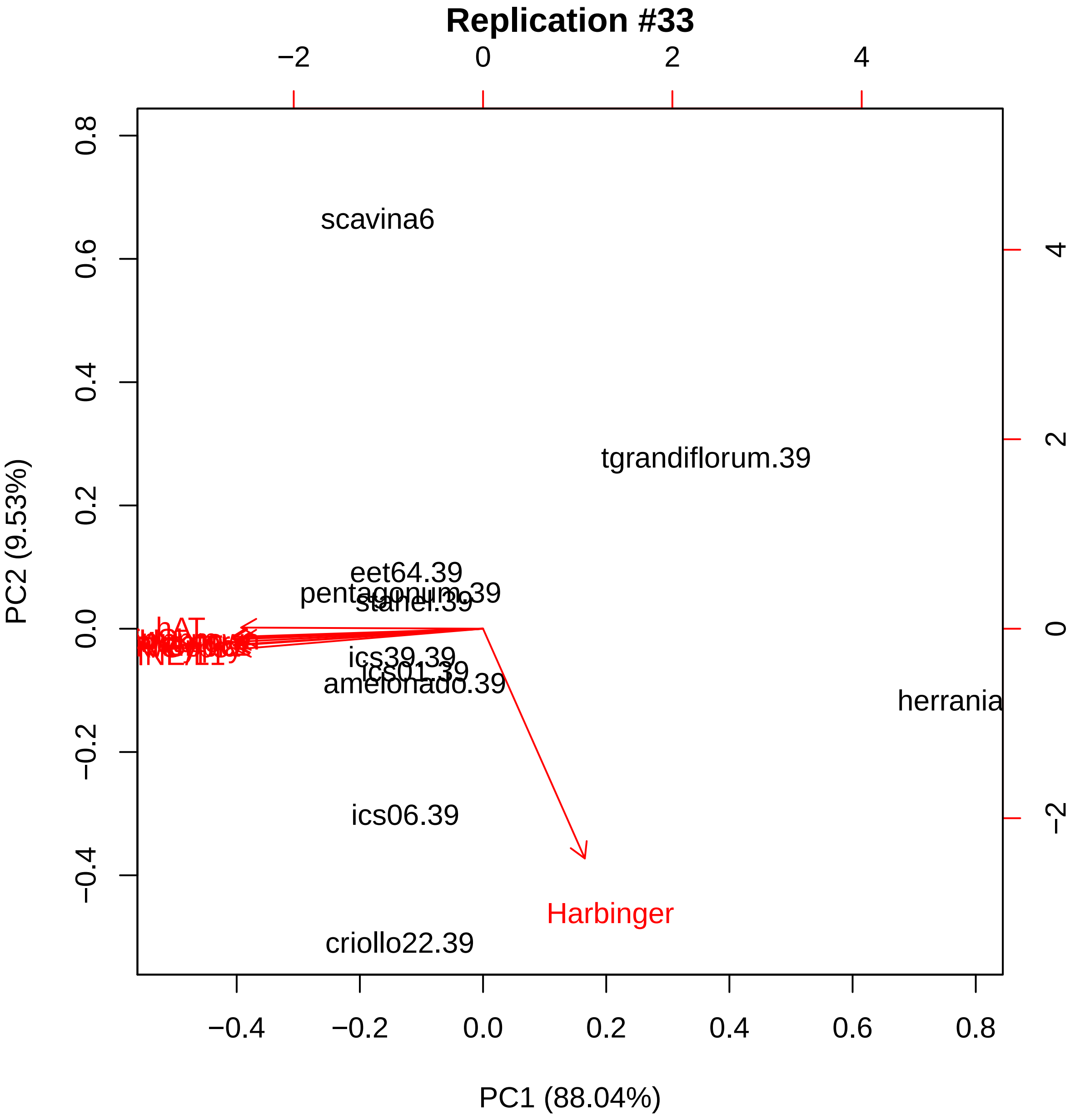

Replication #33
−2
0
2
4
scavina6
tgrandiflorum.39
eet64.39
pentagonum.39
stahel.39
ics39.39
ics01.39
amelonado.39
herrania
ics06.39
Harbinger
criollo22.39
hAT
0.8
0.6
0.4
0.2
0.0
−0.2
−0.4
PC2 (9.53%)
−0.4
−0.2
0.0
0.2
0.4
0.6
0.8
PC1 (88.04%)
4
2
0
−2

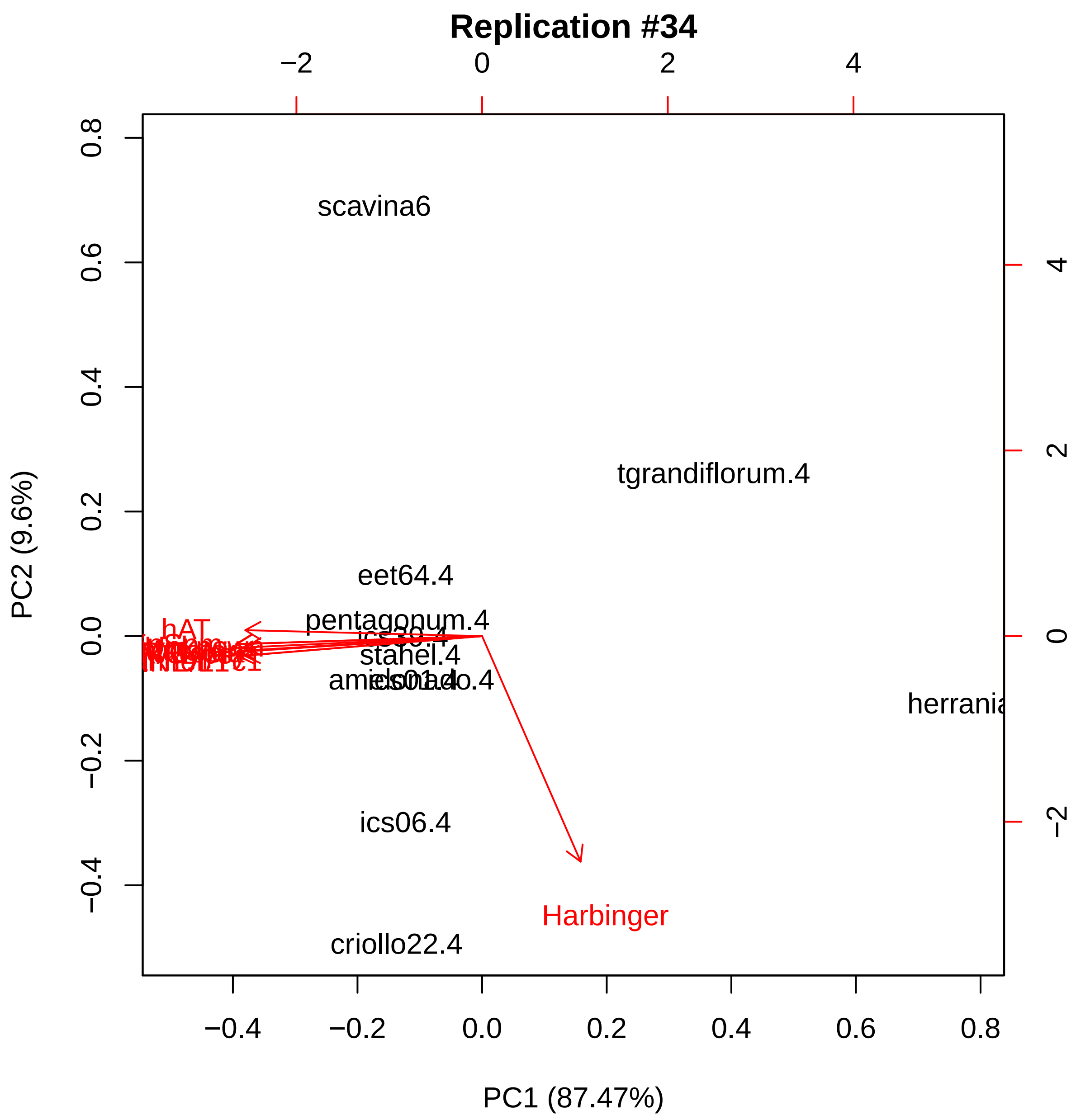

Replication #34
−2
0
2
4
0.8
0.6
0.4
0.2
0.0
−0.2
−0.4
PC2 (9.6%)
scavina6
tgrandiflorum.4
eet64.4
pentagonum.4
stahel.4
herrania
ics06.4
Harbinger
criollo22.4
hAT
−0.4
−0.2
0.0
0.2
0.4
0.6
0.8
PC1 (87.47%)

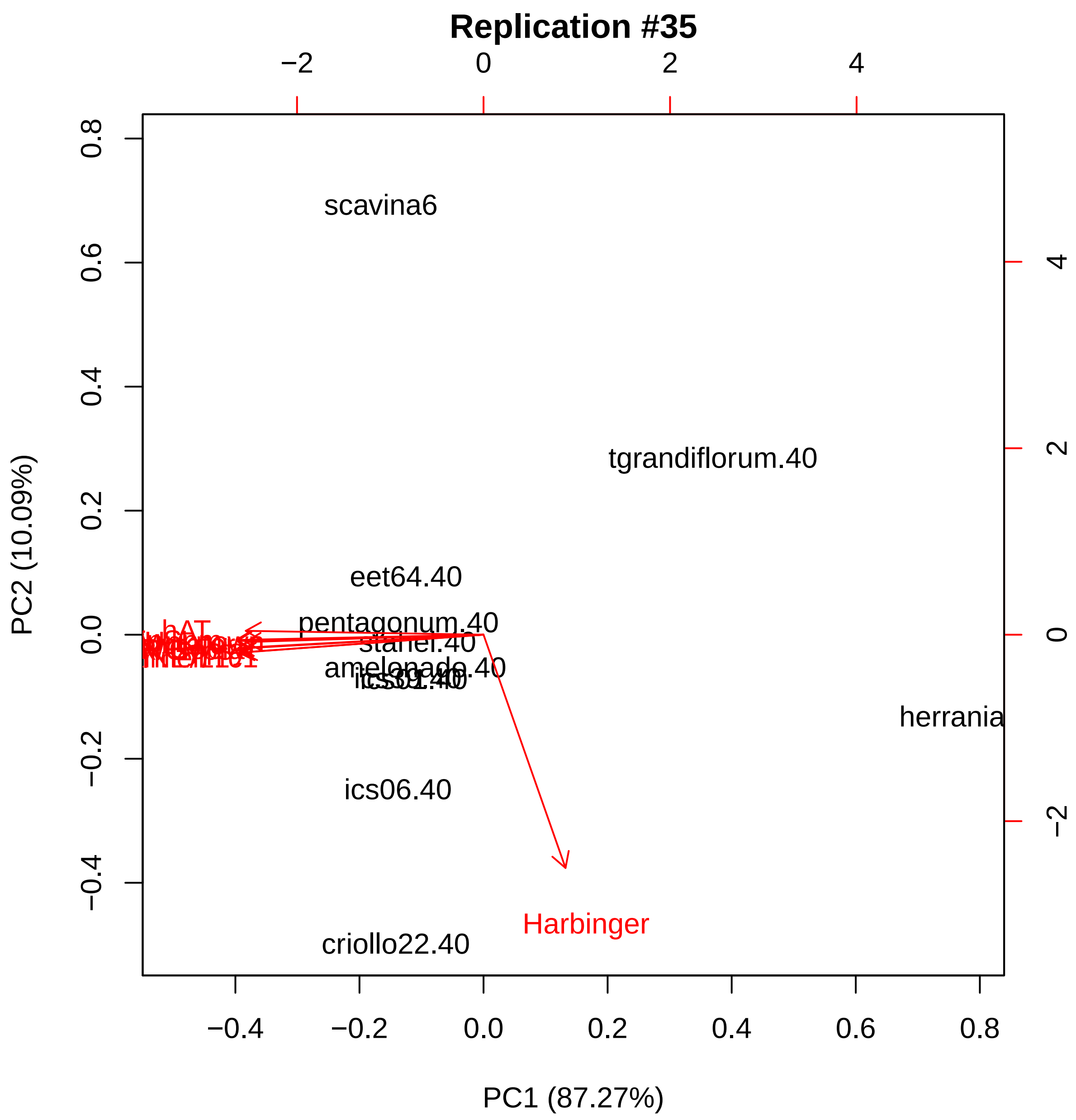

Replication #35
PC1 (87.27%)
PC2 (10.09%)
scavina6
tgrandiflorum.40
eet64.40
pentagonum.40
stahel.40
amelonado.40
herrania
ics06.40
criollo22.40
Harbinger
hAT

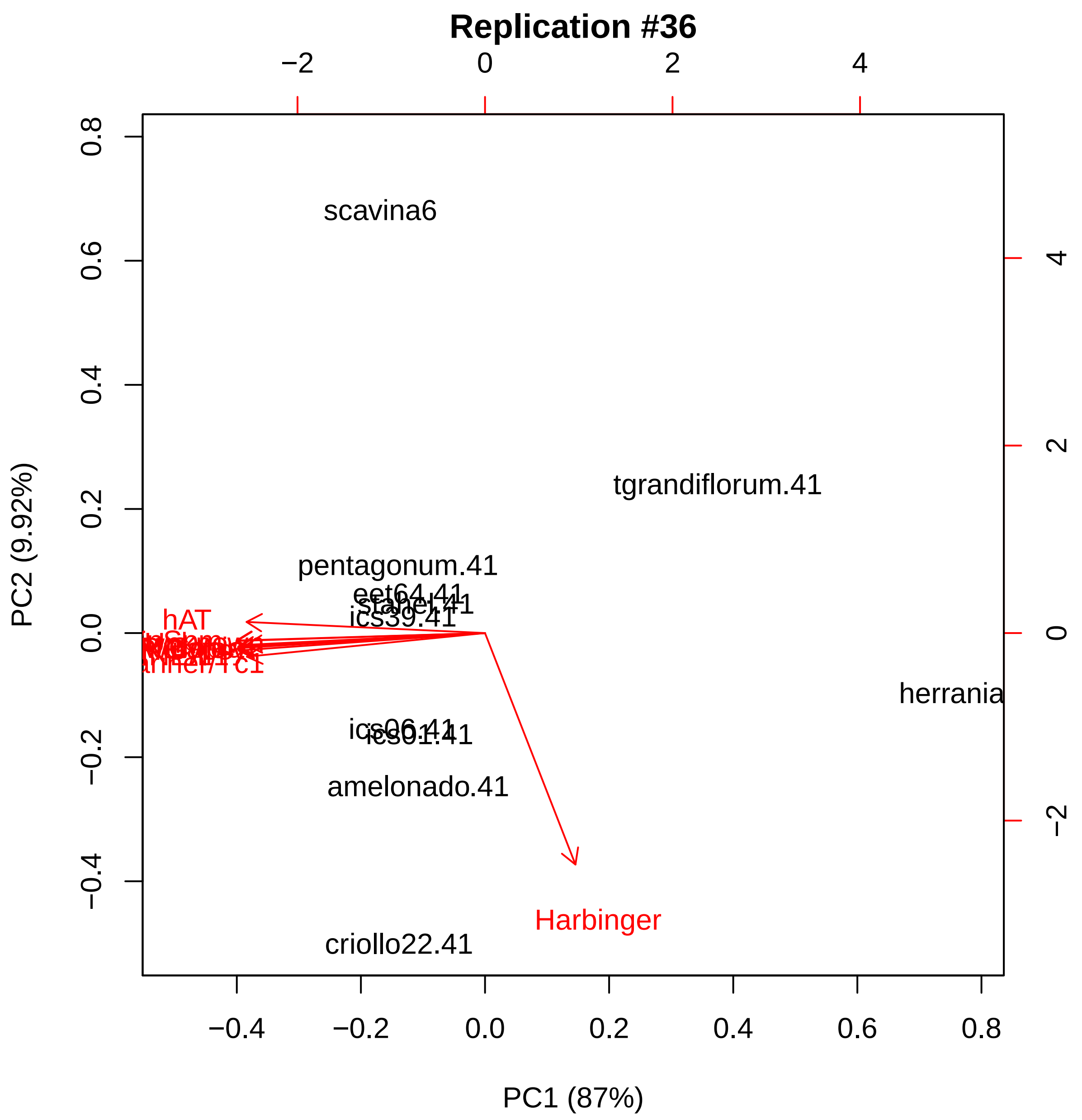

Replication #36
PC1 (87%)
PC2 (9.92%)
−0.4
−0.2
0.0
0.2
0.4
0.6
0.8
−2
0
2
4
scavina6
tgrandiflorum.41
pentagonum.41
eet64.41
stahel.41
ics39.41
hAT
herrania
ics06.41
ics01.41
amelonado.41
Harbinger
criollo22.41

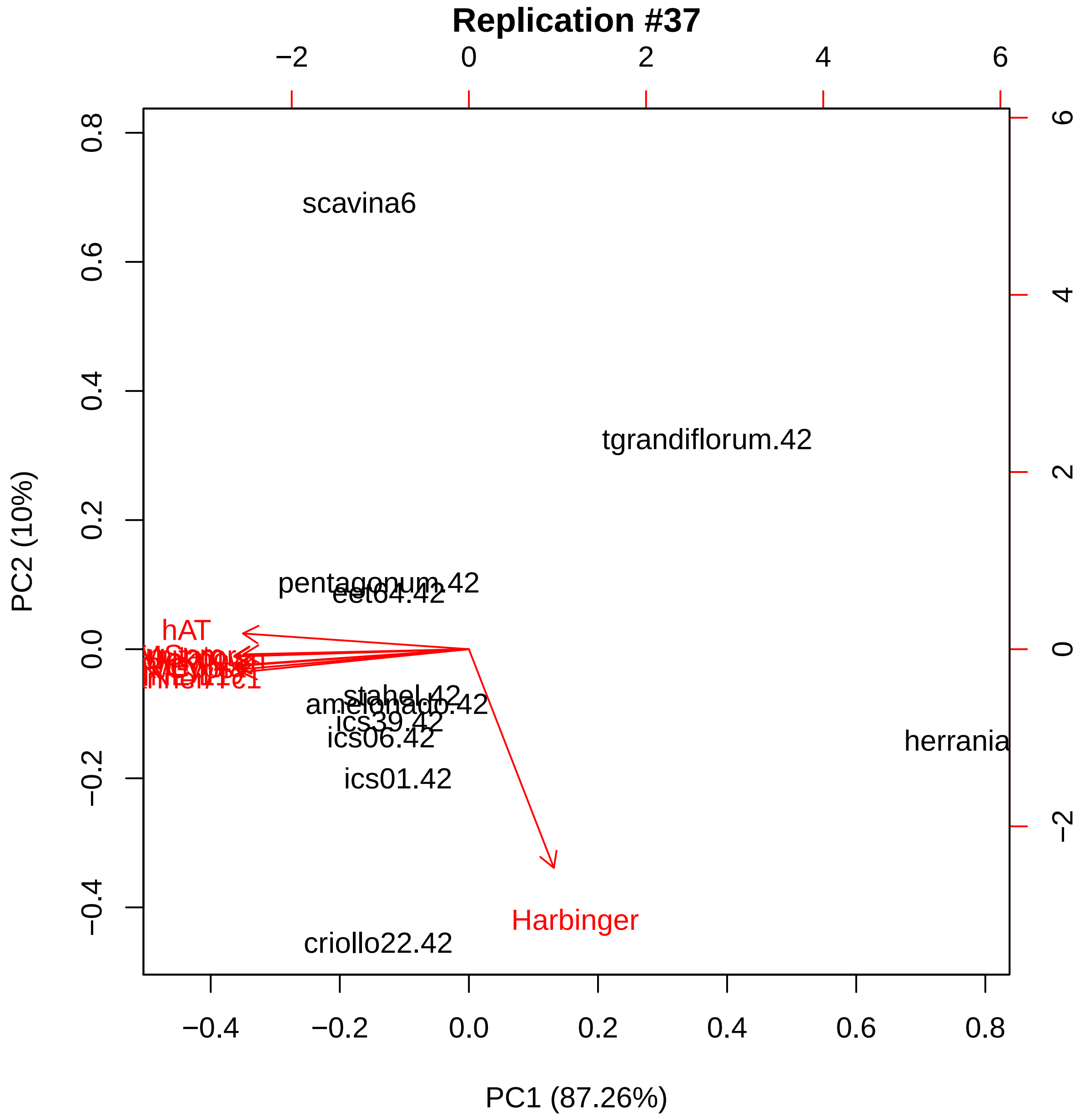

Replication #37
PC1 (87.26%)
PC2 (10%)
−0.4
−0.2
0.0
0.2
0.4
0.6
0.8
−2
0
2
4
6
scavina6
tgrandiflorum.42
pentagonum.42
eet64.42
stahel.42
amelonado.42
ics39.42
ics06.42
ics01.42
criollo22.42
herrania
hAT
Harbinger

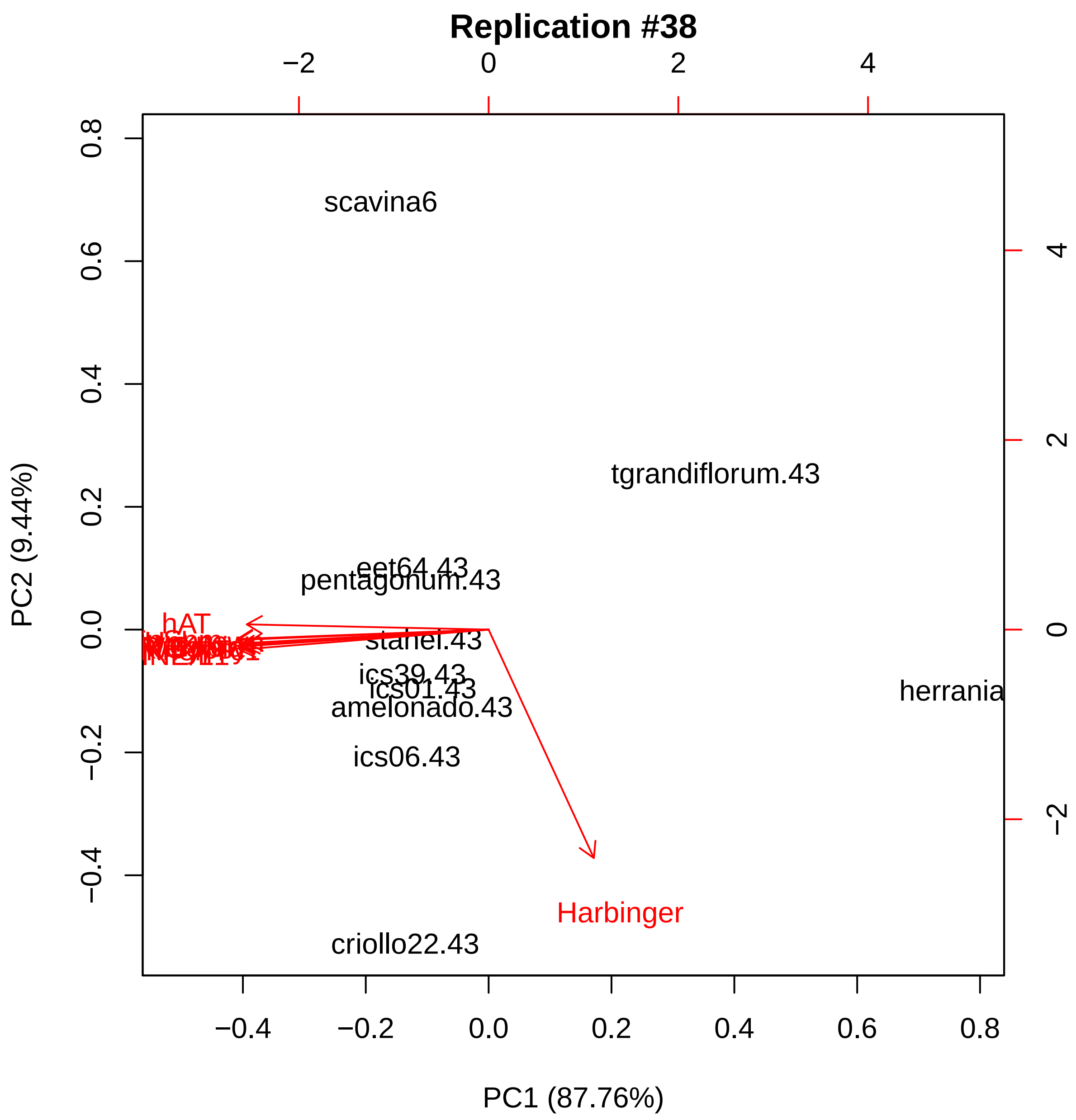

Replication #38
scavina6
tgrandiflorum.43
eet64.43
pentagonum.43
stahel.43
ics39.43
ics01.43
amelonado.43
ics06.43
herrania
criollo22.43
hAT
Harbinger
PC1 (87.76%)
PC2 (9.44%)

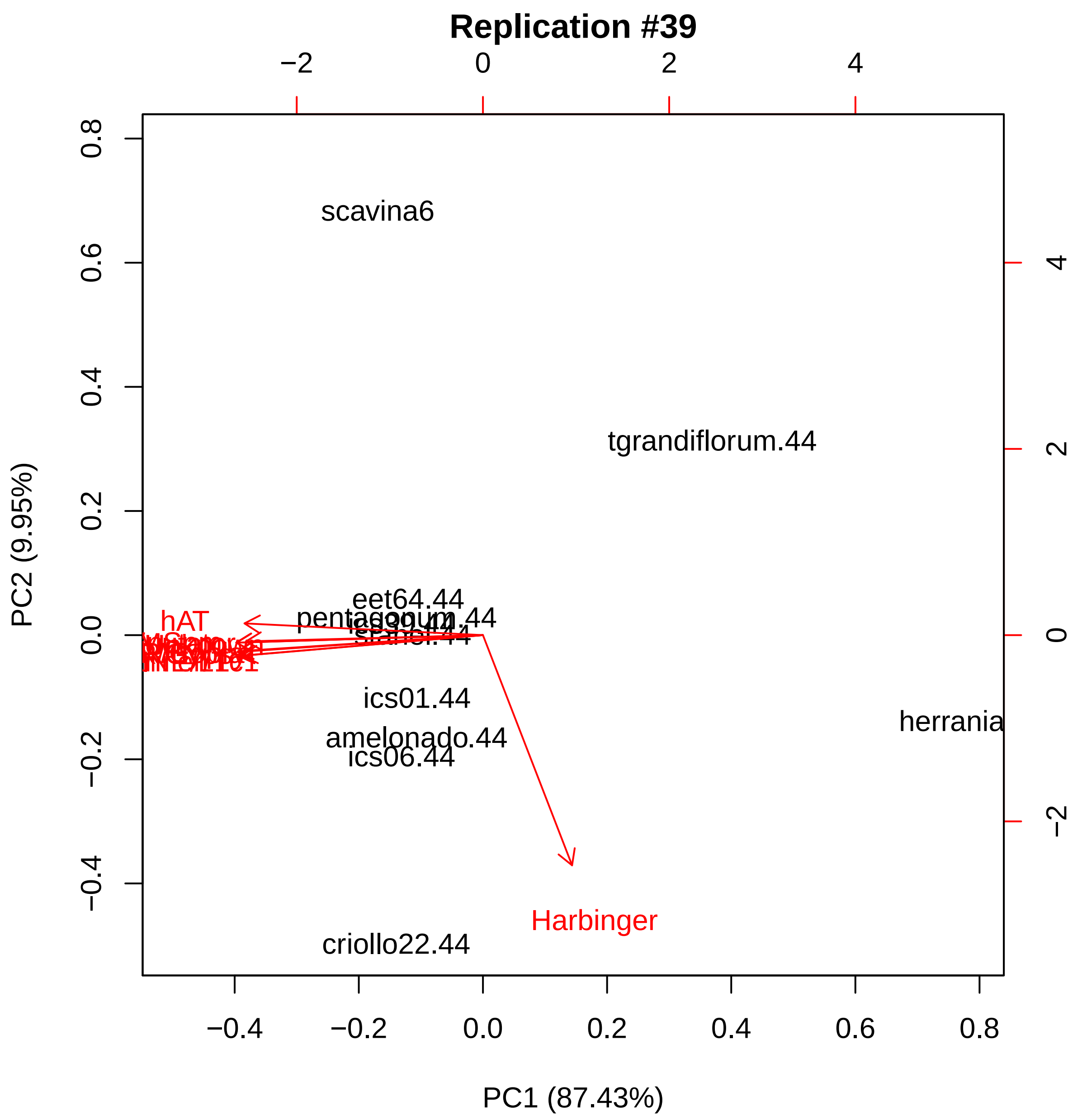

Replication #39
PC1 (87.43%)
PC2 (9.95%)
scavina6
tgrandiflorum.44
eet64.44
pentagonum.44
ics01.44
amelonado.44
ics06.44
herrania
criollo22.44
hAT
Harbinger
−0.4
−0.2
0.0
0.2
0.4
0.6
0.8
−2
0
2
4

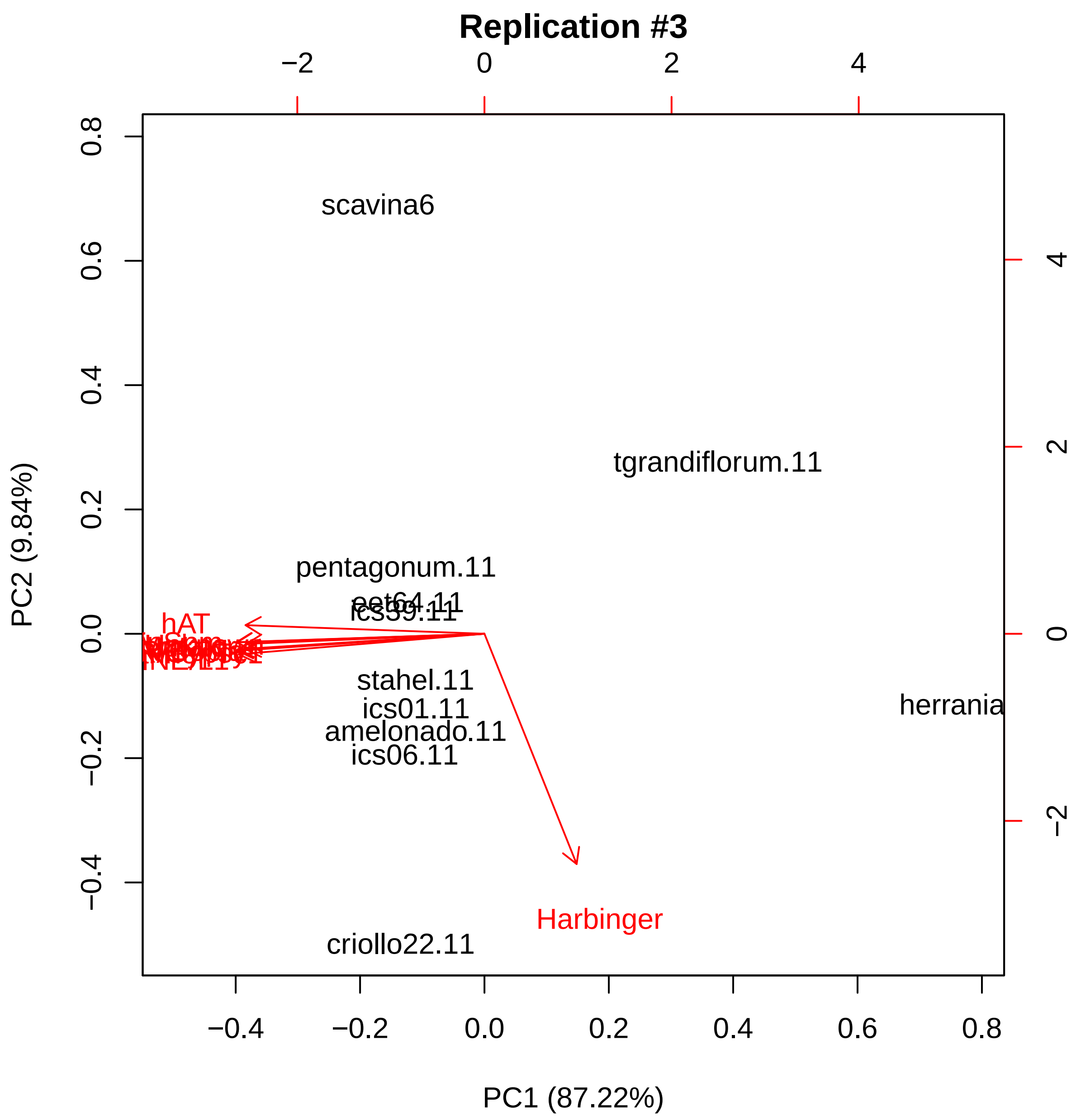
Replication #3
PC1 (87.22%)
PC2 (9.84%)
scavina6
tgrandiflorum.11
pentagonum.11
eet64.11
ics39.11
stahel.11
ics01.11
amelonado.11
ics06.11
criollo22.11
herrania
hAT
Harbinger
−0.4
−0.2
0.0
0.2
0.4
0.6
0.8
−2
0
2
4

Replication #40

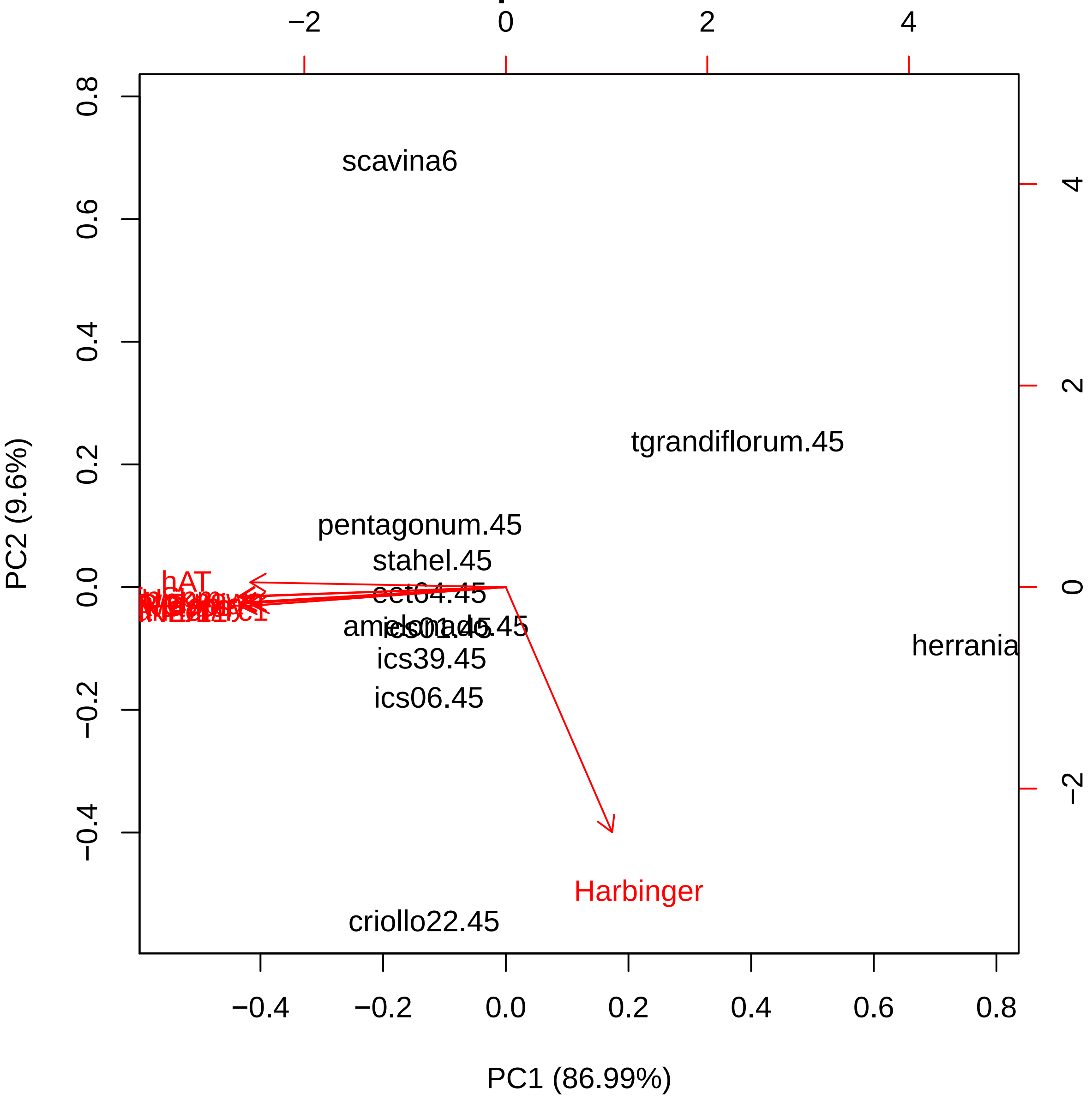

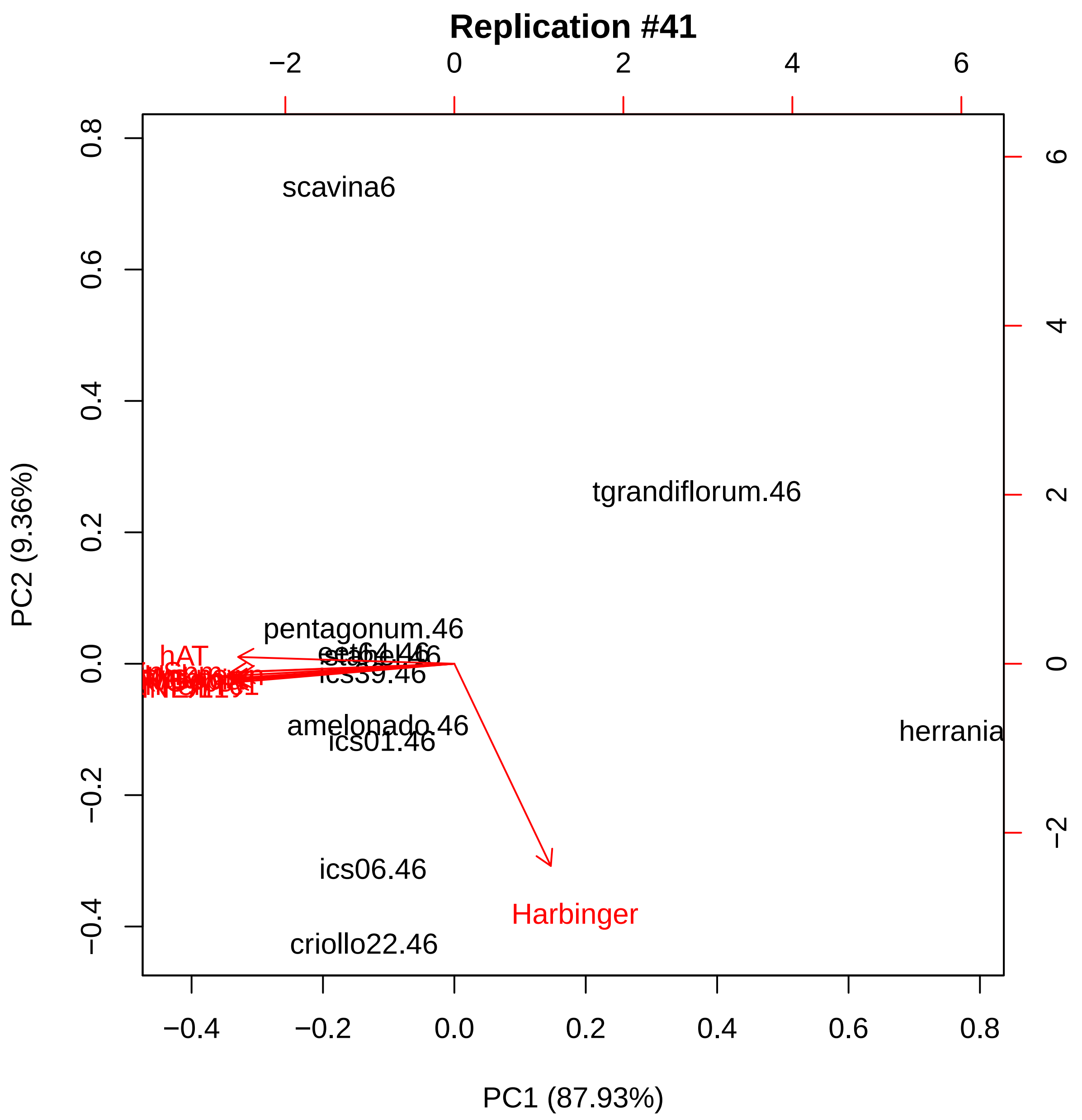
Replication #41
PC1 (87.93%)
PC2 (9.36%)
scavina6
tgrandiflorum.46
pentagonum.46
ics39.46
amelonado.46
ics01.46
herrania
ics06.46
criollo22.46
Harbinger
hAT
−2
0
2
4
6
−0.4
−0.2
0.0
0.2
0.4
0.6
0.8

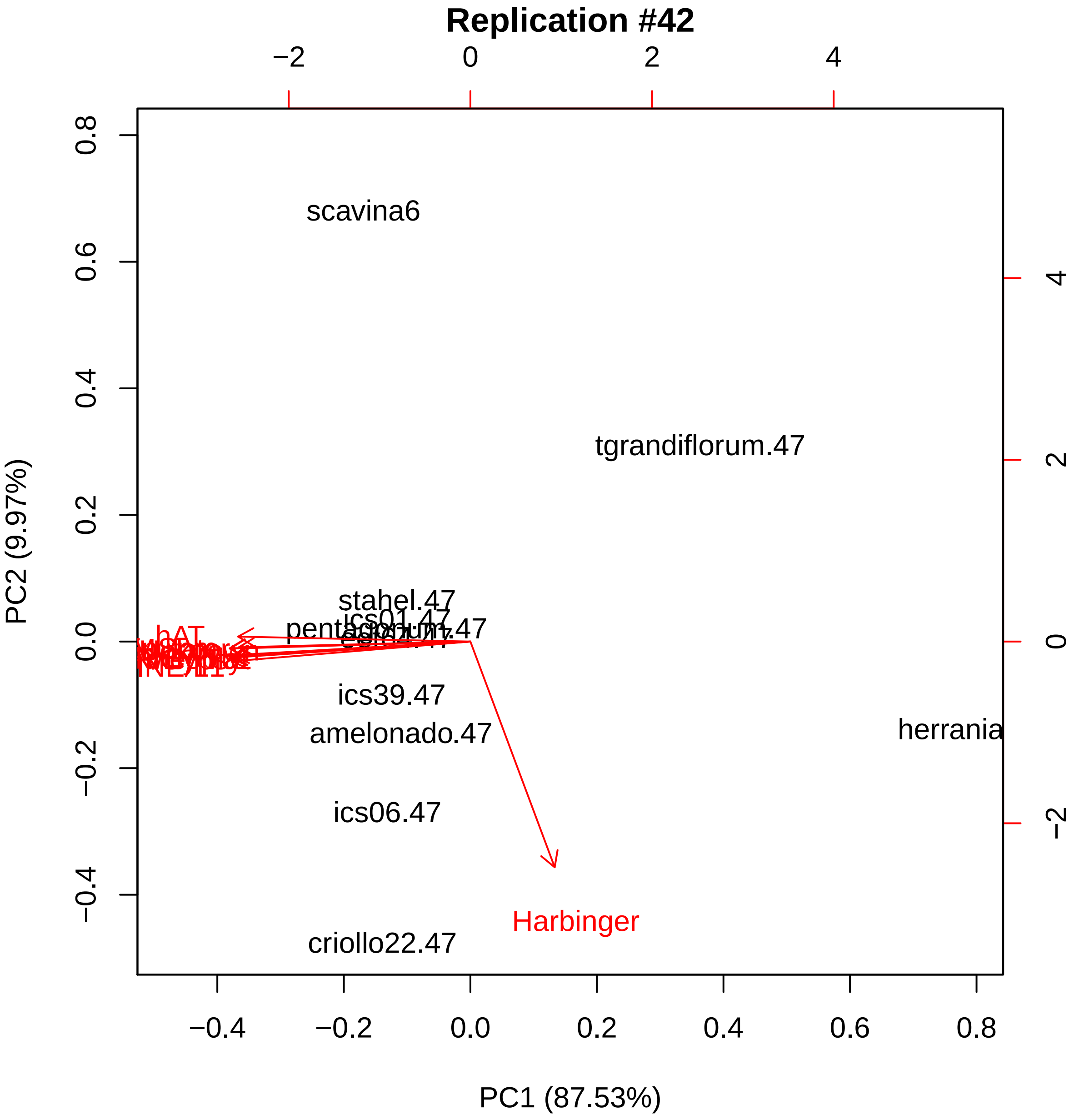

Replication #42
−2
0
2
4
PC2 (9.97%)
0.8
0.6
0.4
0.2
0.0
−0.2
−0.4
4
2
0
−2
scavina6
tgrandiflorum.47
stahel.47
ics01.47
pentagonum.47
hAT
ics39.47
amelonado.47
herrania
ics06.47
Harbinger
criollo22.47
−0.4
−0.2
0.0
0.2
0.4
0.6
0.8
PC1 (87.53%)

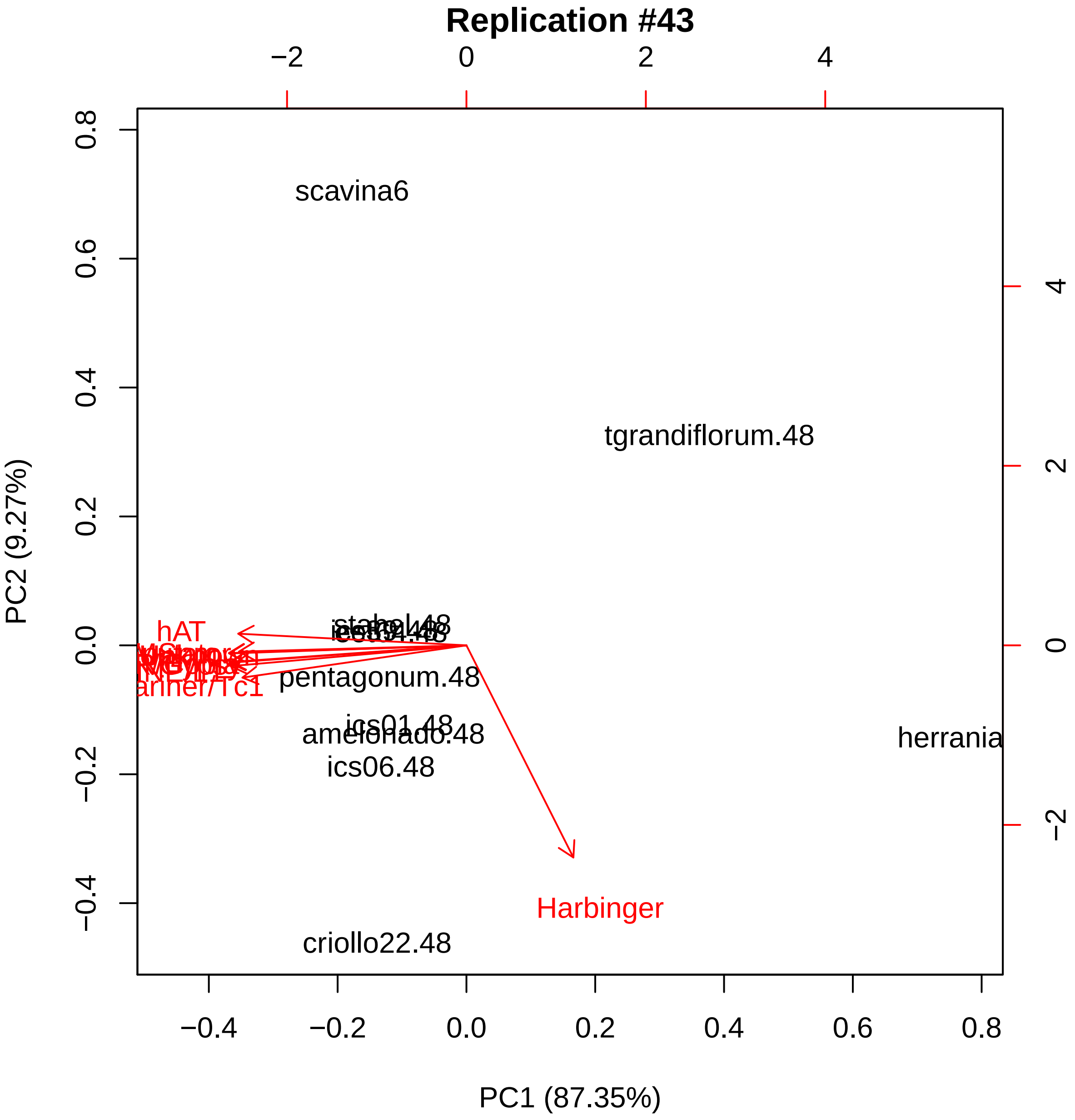
Replication #43
−2
0
2
4
0.8
0.6
0.4
0.2
0.0
−0.2
−0.4
PC2 (9.27%)
−0.4
−0.2
0.0
0.2
0.4
0.6
0.8
PC1 (87.35%)
4
2
0
−2
scavina6
tgrandiflorum.48
pentagonum.48
ics01.48
ics06.48
herrania
criollo22.48
hAT
Harbinger

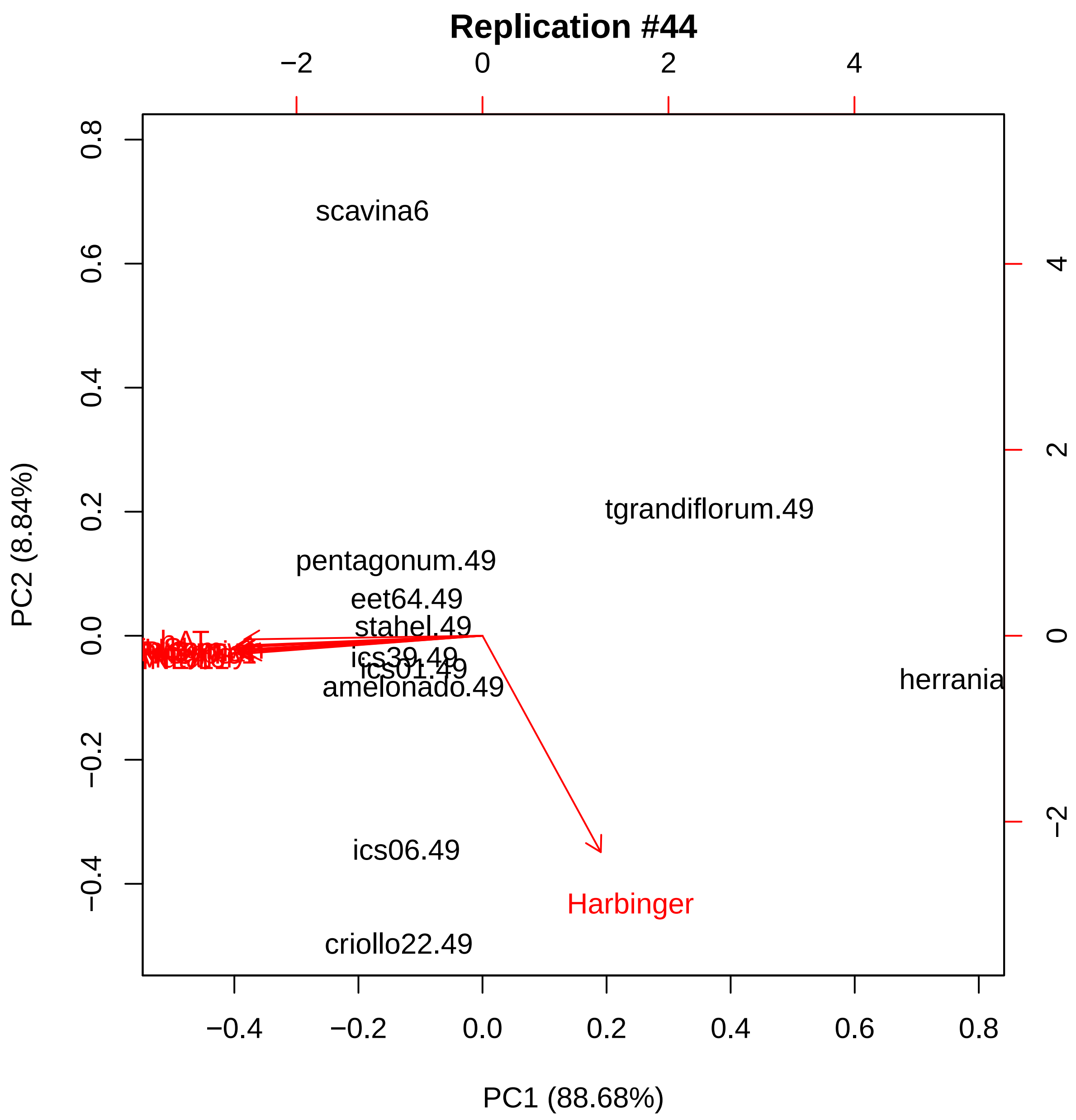

Replication #44
scavina6
tgrandiflorum.49
pentagonum.49
eet64.49
stahel.49
ics39.49
ics01.49
amelonado.49
herrania
ics06.49
Harbinger
criollo22.49
PC1 (88.68%)
PC2 (8.84%)

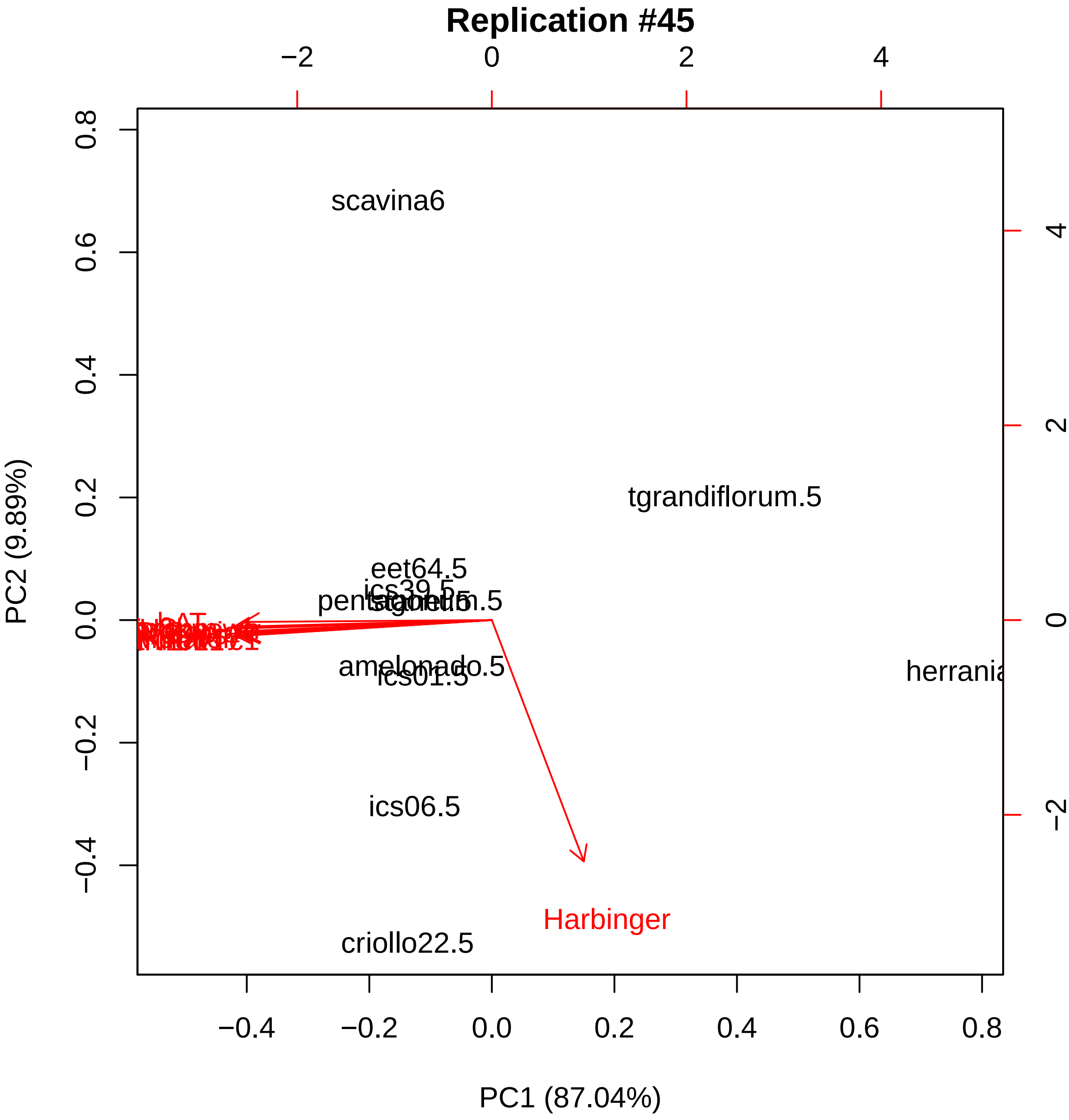
Replication #45
PC1 (87.04%)
PC2 (9.89%)
−2
0
2
4
0.8
0.6
0.4
0.2
0.0
−0.2
−0.4
−0.4
−0.2
0.0
0.2
0.4
0.6
0.8
4
2
0
−2
scavina6
tgrandiflorum.5
eet64.5
ics39.5
amelonado.5
ics01.5
herrania
ics06.5
criollo22.5
Harbinger

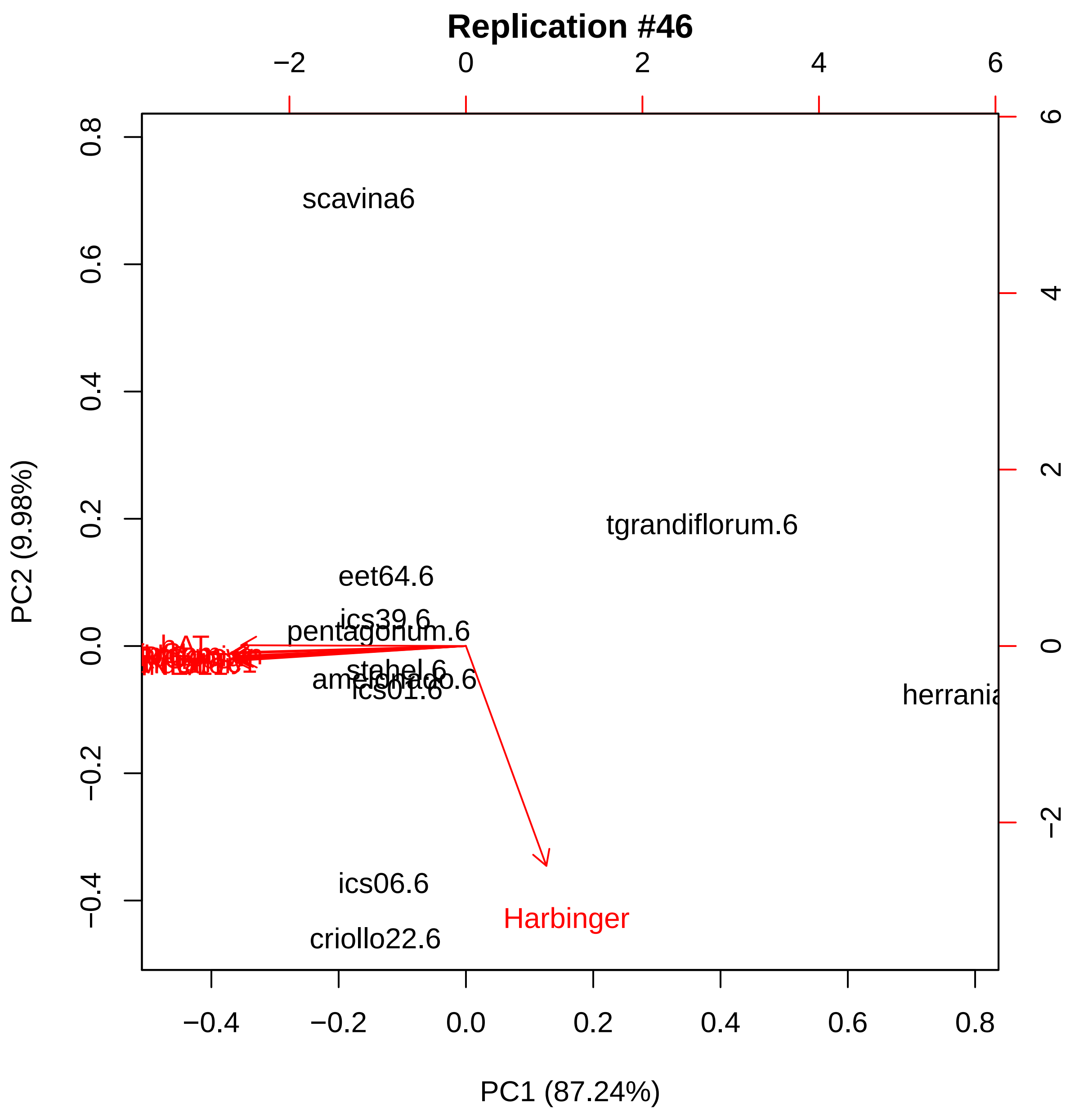

Replication #46
PC1 (87.24%)
PC2 (9.98%)
scavina6
tgrandiflorum.6
eet64.6
ics39.6
pentagonum.6
stahel.6
amelonado.6
ics01.6
herrania
ics06.6
criollo22.6
Harbinger
−0.4
−0.2
0.0
0.2
0.4
0.6
0.8
−2
0
2
4
6

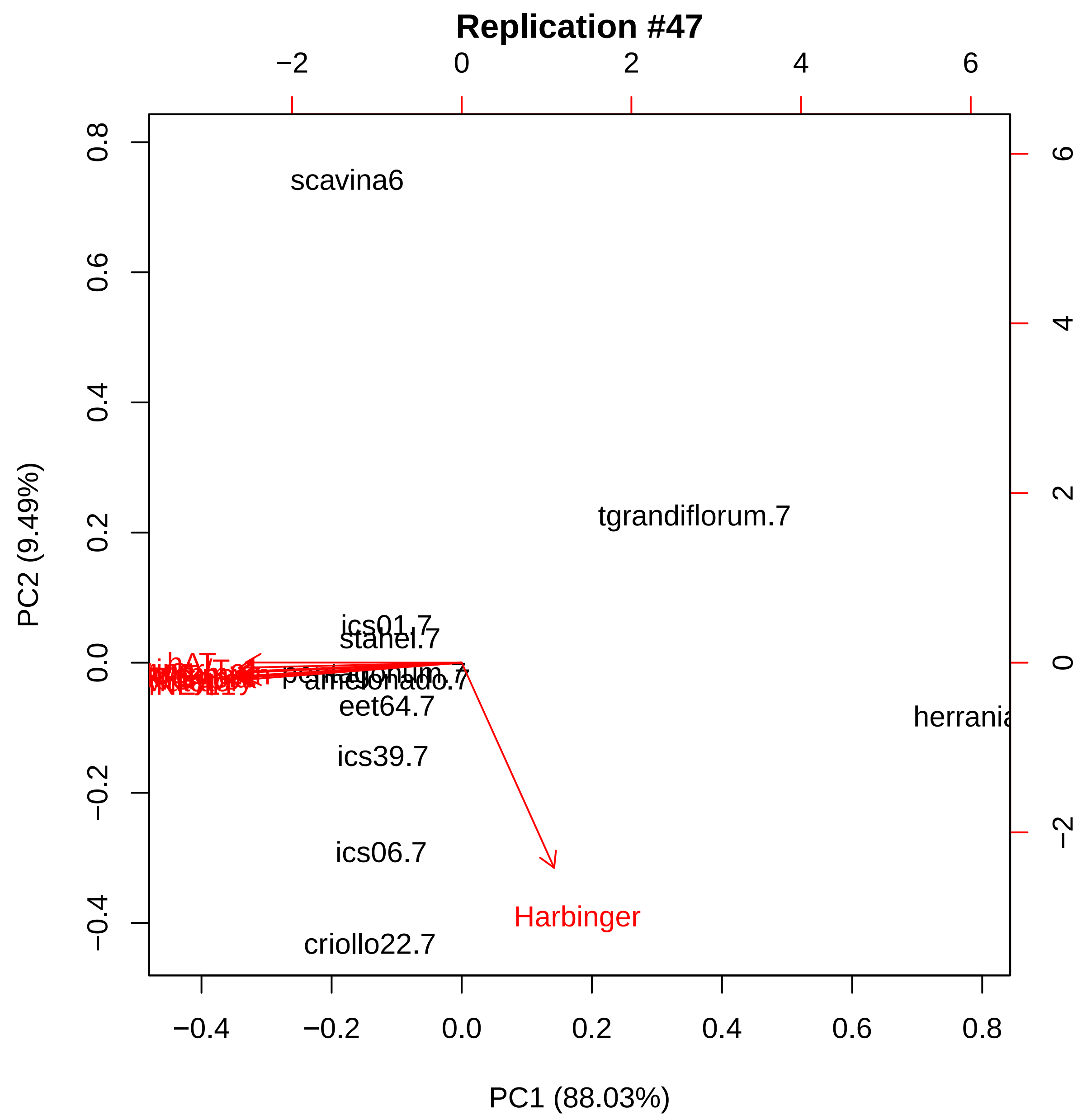

Replication #47
PC1 (88.03%)
PC2 (9.49%)
scavina6
tgrandiflorum.7
ics01.7
stahel.7
amelonado.7
eet64.7
ics39.7
ics06.7
criollo22.7
herrania
Harbinger

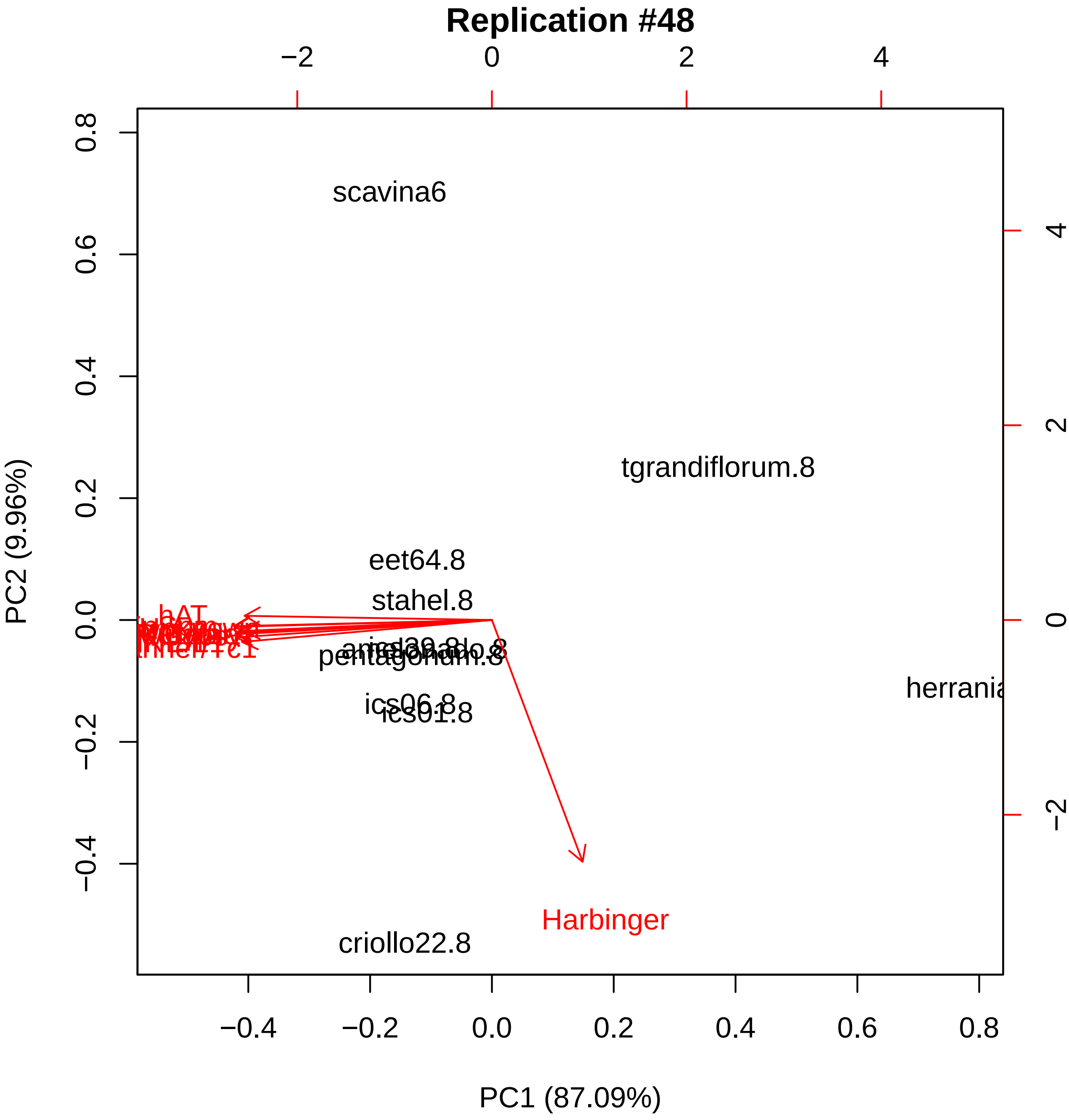

Replication #48
PC1 (87.09%)
PC2 (9.96%)
scavina6
tgrandiflorum.8
eet64.8
stahel.8
pentagonum.8
ics06.8
ics01.8
herrania
criollo22.8
Harbinger
hAT
−0.4
−0.2
0.0
0.2
0.4
0.6
0.8
−2
0
2
4

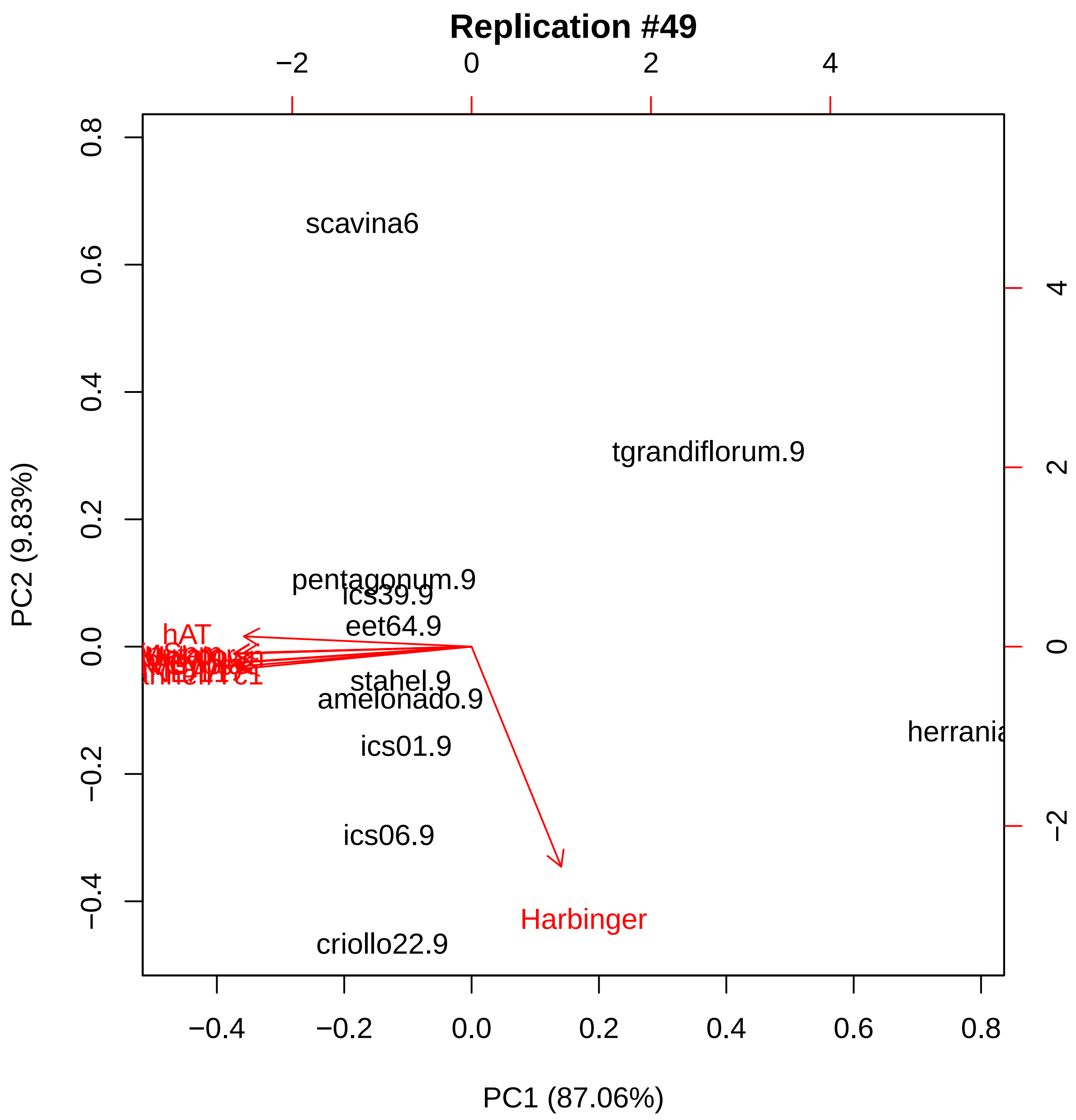

Replication #49
PC1 (87.06%)
PC2 (9.83%)
scavina6
tgrandiflorum.9
pentagonum.9
ics39.9
eet64.9
stahel.9
amelonado.9
ics01.9
ics06.9
criollo22.9
herrania
hAT
Harbinger
−2
0
2
4
−0.4
−0.2
0.0
0.2
0.4
0.6
0.8

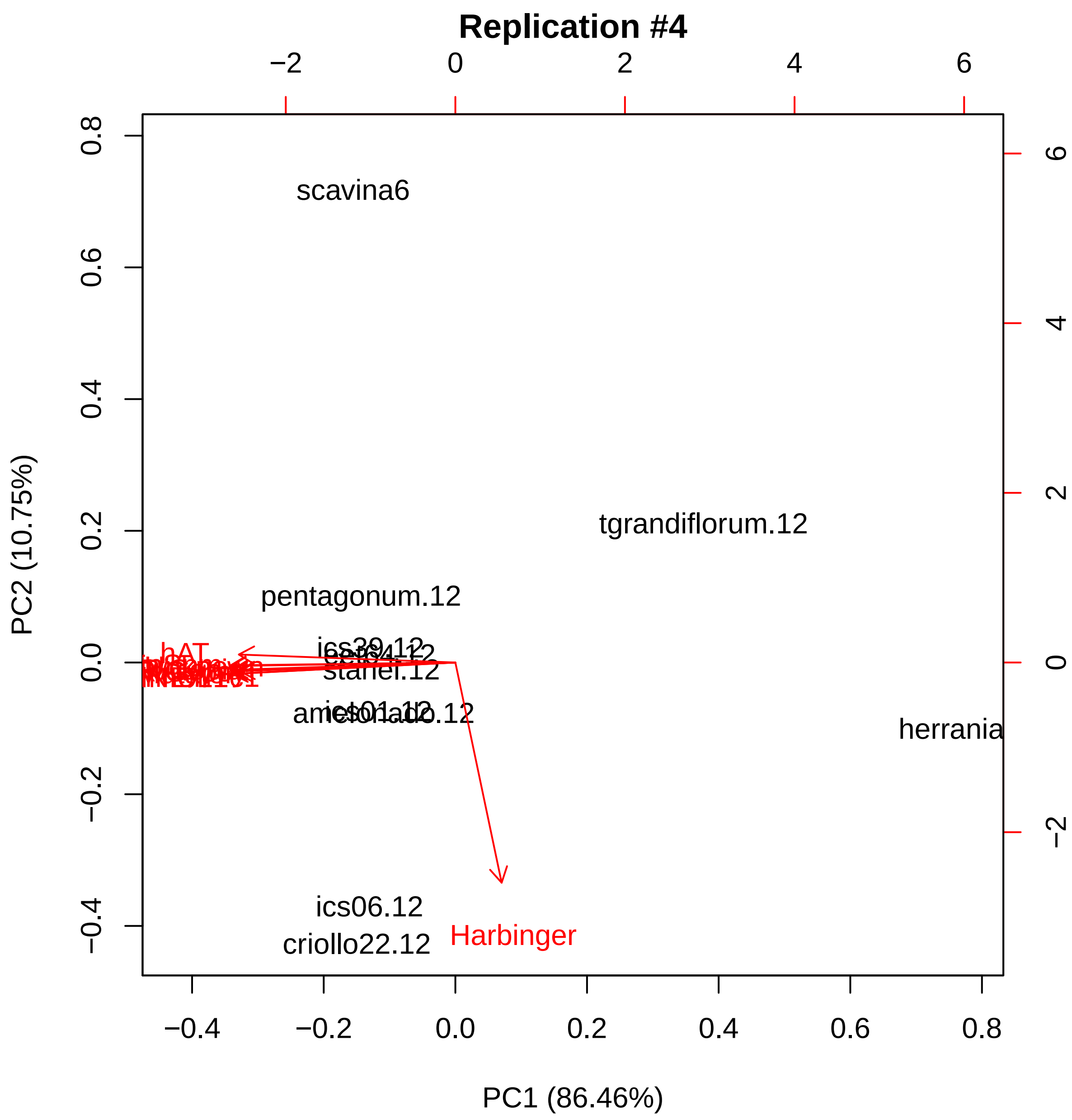
Replication #4
−2
0
2
4
6
0.8
0.6
0.4
0.2
0.0
−0.2
−0.4
PC2 (10.75%)
scavina6
tgrandiflorum.12
pentagonum.12
ics39.12
hAT
herrania
ics06.12
criollo22.12
Harbinger
−0.4
−0.2
0.0
0.2
0.4
0.6
0.8
PC1 (86.46%)

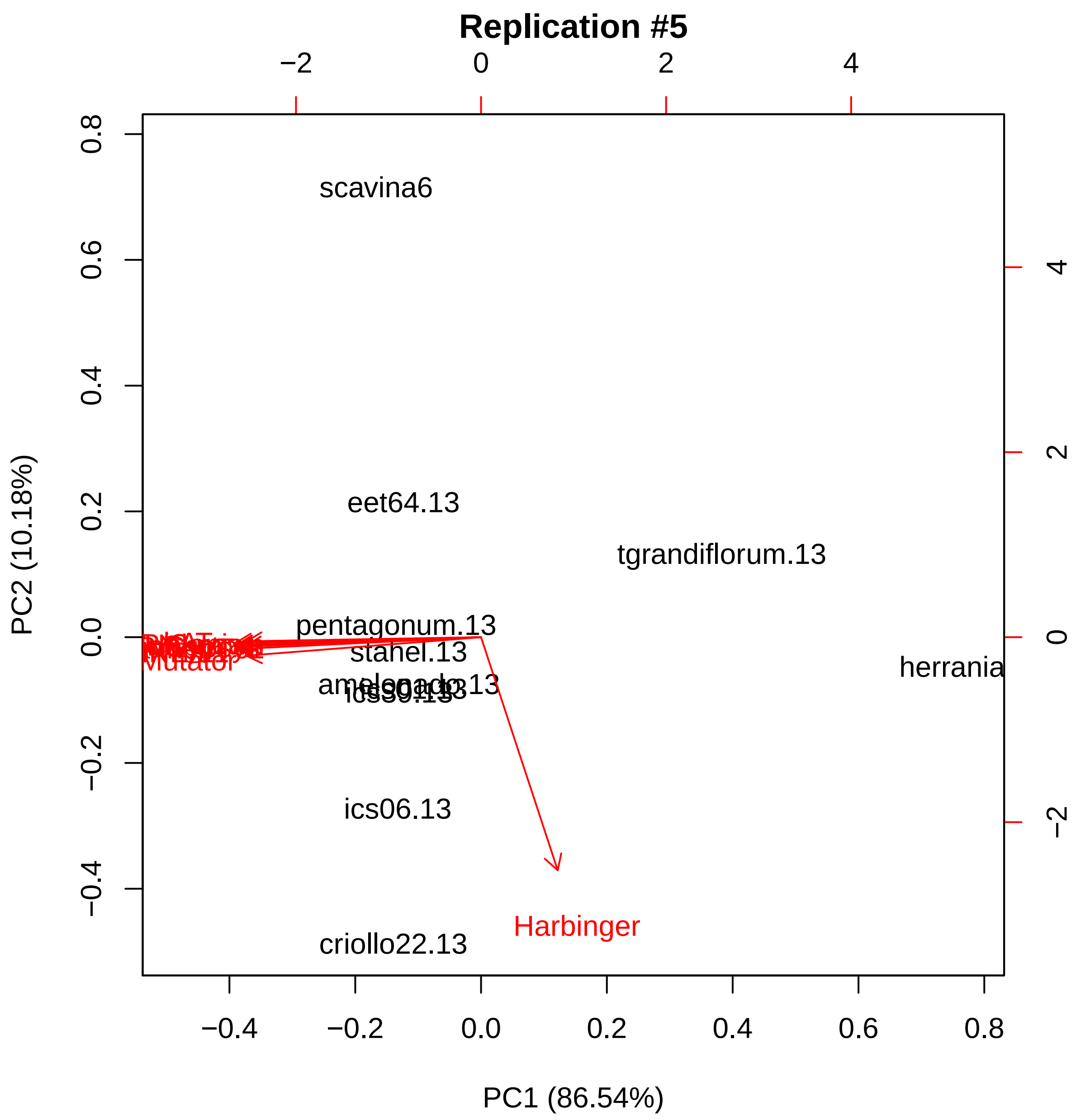

Replication #5
−2
0
2
4
0.8
0.6
0.4
0.2
0.0
−0.2
−0.4
PC2 (10.18%)
scavina6
eet64.13
tgrandiflorum.13
pentagonum.13
stahel.13
herrania
amelonado.13
ics39.13
ics06.13
Mutator
Harbinger
criollo22.13
−0.4
−0.2
0.0
0.2
0.4
0.6
0.8
PC1 (86.54%)

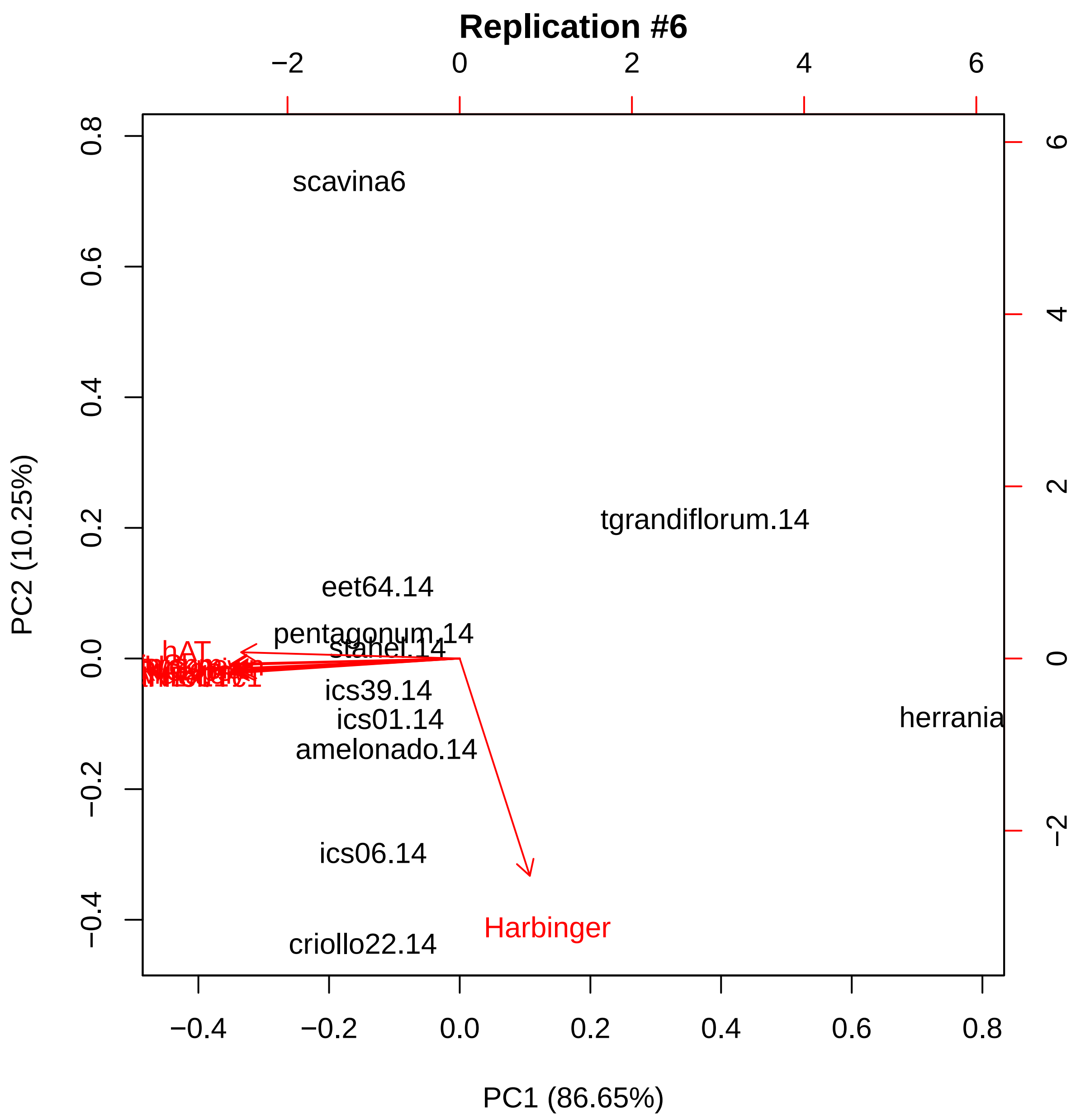

Replication #6
scavina6
tgrandiflorum.14
eet64.14
pentagonum.14
stahel.14
hAT
ics39.14
ics01.14
amelonado.14
herrania
ics06.14
criollo22.14
Harbinger
PC1 (86.65%)
PC2 (10.25%)
−0.4
−0.2
0.0
0.2
0.4
0.6
0.8
−2
0
2
4
6

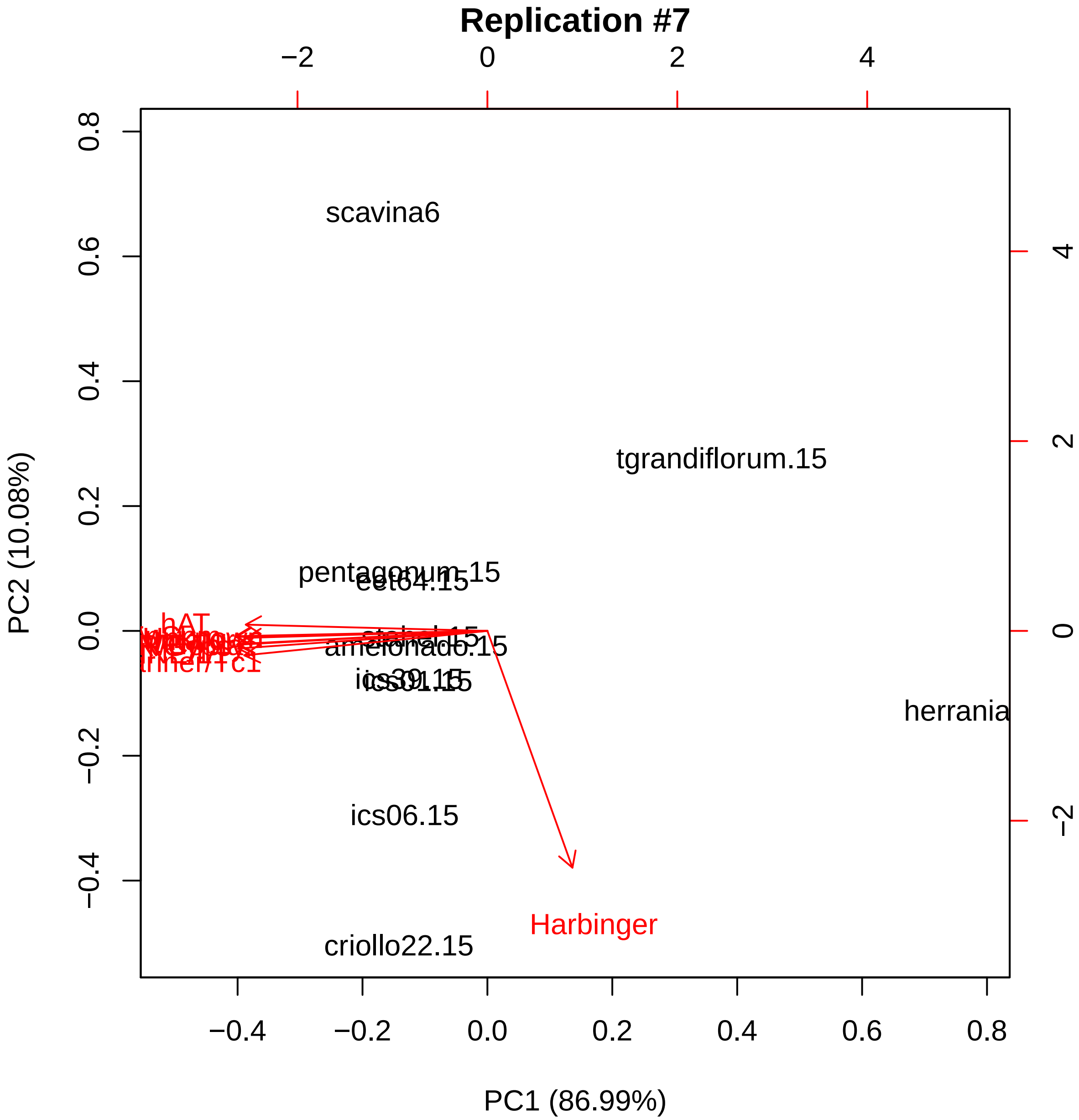
Replication #7
PC1 (86.99%)
PC2 (10.08%)
−0.4
−0.2
0.0
0.2
0.4
0.6
0.8
−2
0
2
4
scavina6
tgrandiflorum.15
pentagonum.15
amelonado.15
herrania
ics06.15
criollo22.15
hAT
Harbinger

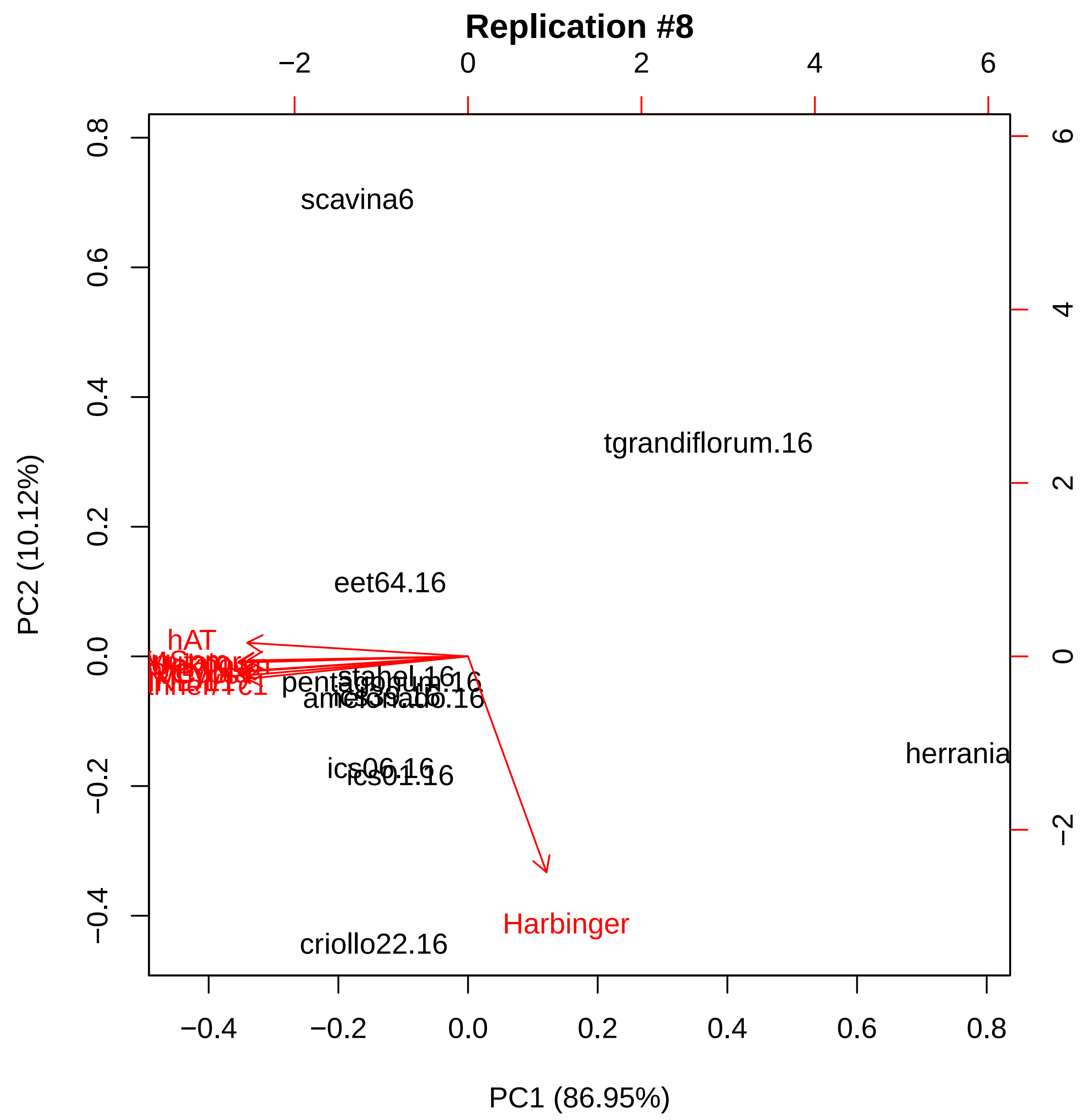

Replication #8
PC1 (86.95%)
PC2 (10.12%)
scavina6
tgrandiflorum.16
eet64.16
hAT
stahel.16
pentagonum.16
amelonado.16
ics06.16
ics01.16
herrania
Harbinger
criollo22.16

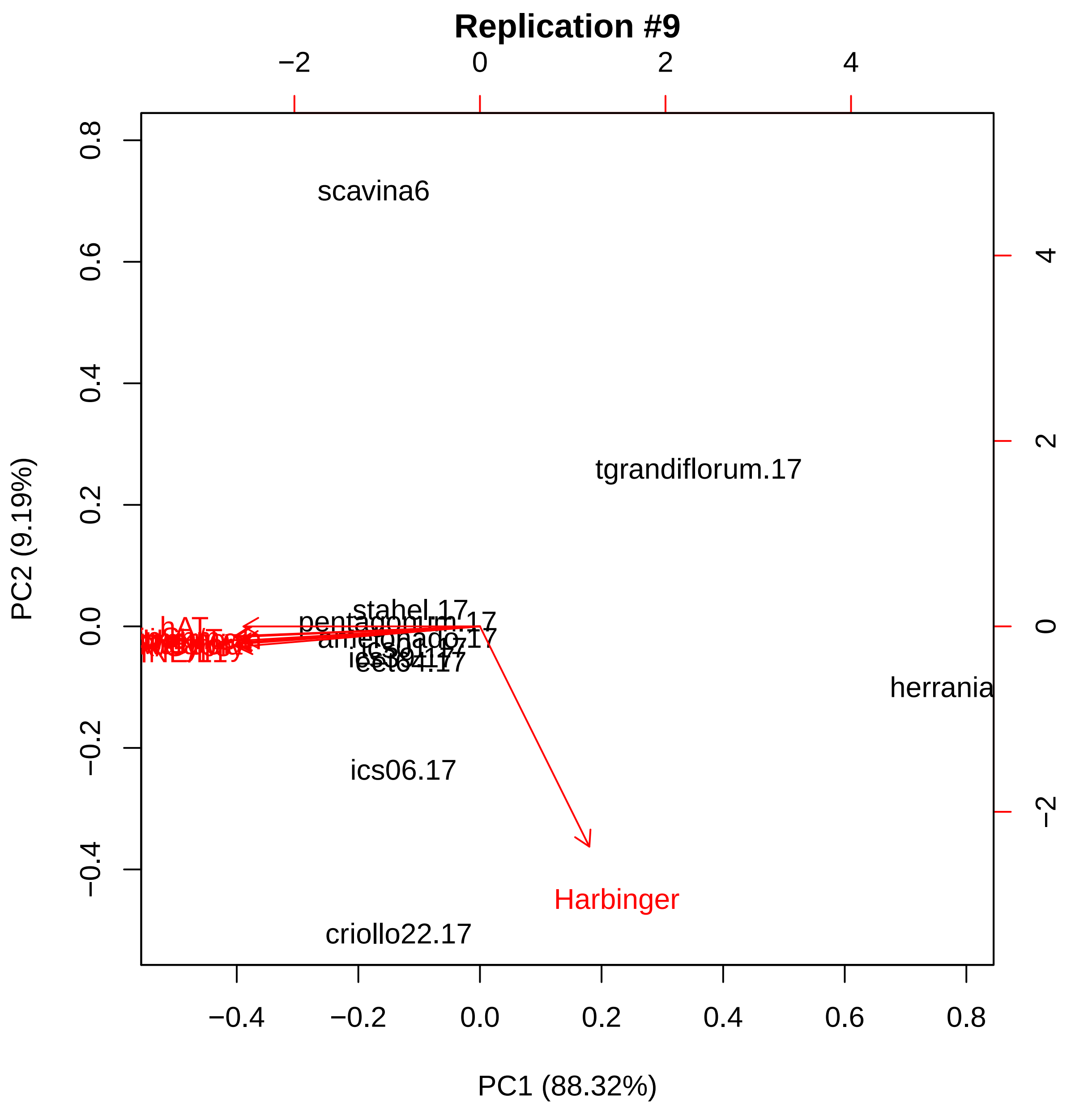

Replication #9
PC1 (88.32%)
PC2 (9.19%)
scavina6
tgrandiflorum.17
stahel.17
herrania
ics06.17
criollo22.17
Harbinger
hAT
